# DHX37 Impacts Prognosis of Hepatocellular Carcinoma and Lung Adenocarcinoma through Immune Infiltration

**DOI:** 10.1155/2020/8835393

**Published:** 2020-12-30

**Authors:** Yanni Xu, Qiongchao Jiang, Hejun Liu, Xiaoyun Xiao, Dinghong Yang, Phei Er Saw, Baoming Luo

**Affiliations:** ^1^Department of Ultrasound, Sun Yat-sen Memorial Hospital, Sun Yat-sen University, 107 Yanjiangxi Rd., Guangzhou, 510120 Guangdong Province, China; ^2^Guangdong Provincial Key Laboratory of Malignant Tumor Epigenetics and Gene Regulation, Sun Yat-sen Memorial Hospital, Sun Yat-sen University, China; ^3^Department of Hyperbaric Oxygen, Sun Yat-sen Memorial Hospital, Sun Yat-sen University, Guangzhou 510120, China; ^4^Guangdong Provincial Key Laboratory of Malignant Tumor Epigenetics and Gene Regulation, Medical Research Center, Sun Yat-sen Memorial Hospital, Sun Yat-sen University, Guangzhou, China 510120

## Abstract

**Background:**

RNA helicases have various essential functions in basically all aspects of RNA metabolism, not only unwinding RNA but also disturbing the interaction of RNA with proteins. Recently, RNA helicases have been considered potential targets in cancers. So far, there has been no detailed investigation of the biological functions of RNA helicase DHX37 in cancers.

**Objective:**

We aim to identify the prognostic value of DHX37 associated with tumor microenvironments in cancers.

**Methods:**

DHX37 expression was examined via the Oncomine database and Tumor Immune Estimation Resource (TIMER). We explored the prognostic role of DHX37 in cancers across various databases. Coexpression genes, Gene Ontology (GO) and Kyoto Encyclopedia of Genes and Genomes (KEGG), and fundamental regulators were performed via LinkedOmics. Confirming the prognostic value of DHX37 in liver hepatocellular carcinoma (LIHC) and lung adenocarcinoma (LUAD), we explored the role of DHX37 in infiltrated lymphocytes in cancers using the Gene Expression Profiling Interactive Analysis (GEPIA) and TIMER databases.

**Results:**

Through GO and KEGG analyses, expression of DHX37 was also correlated with complex function-specific networks involving the ribosome and RNA metabolic signaling pathways. In LIHC and LUAD, DHX37 expression showed significant positive correlations with markers of T_regs_, myeloid-derived suppressor cells (MDSCs), and T cell exhaustion, contributing to immune tolerance.

**Conclusion:**

These results indicate that DHX37 can serve as a prognostic biomarker in LIHC and LUAD while having an important role in immune tolerance by activating the function of T_regs_, MDSC, and T cell exhaustion.

## 1. Introduction

Cancer is now known to be a disease which involves multiple players, including its relationship with micro- and macroenvironments. Tumor microenvironment (TME) is crucial in cancer progression and therapeutic responses [1, 2] and consists of complex components, among which inflammatory cells make up for the majority proportion and are valuable for diagnostic and prognostic assessment of tumors. The widely used therapy of immune checkpoint modulators, including programmed cell death protein 1 (PD-1), cytotoxic T lymphocyte-associated antigen-4 (CTLA4), lymphocyte activation gene-3 (LAG-3), and T cell immunoglobulin mucin 3 (TIM-3) [3, 4], has become a promising anticancer therapy by reversing the suppressed immune status in tumors [5, 6]. However, immunotherapy failed to have satisfactory effects in that only 20% of cancer patients benefited with significantly increased survival rates [7, 8], due to a combination of different clinical and biological behaviors [9].

In recent years, cumulative evidence has revealed that RNA helicases can modulate physiological processes like innate immune reactions, carcinogenic disorders, and inflammatory disorders [10–13]. For example, RNA helicase A/DHX9, as a potential therapeutic target, is associated with cancer risk and inflammation [14]. The abnormally high expression of RNA helicases has been tested in a variety of cancers [15]. DHX15 is significantly upregulated in HCC, and its high expression was correlated with poor prognosis. DDX3X drives posttranscriptional programming that dictates melanoma phenotype and poor disease prognosis. RNA helicases p68 and p72 show increased expression during colon carcinogenesis.

DHX37 is a highly conserved DEAH box RNA helicase essential for development of the ribosome [16]. It conjugates to the U3 small nucleolar RNA and is significant for remodeling the U3 snoRNA-pre-18S rRNA structure during 40S maturation [17]; this ensures the formation of the central pseudoknot structure. The production of eukaryotic ribosomes is a massively complicated and energy-draining intracellular activity responsible for the translation of mRNAs into proteins [18].

Previous immunological characterization of CD8 T cells indicated that DHX37 suppressed effector functions, cytokine production, and T cell activation by modulating NF-*κ*B [19], which first showed that DHX37 was related to the progression of cancers. This suggests that DHX37 may be a potential biomarker for cancers and is involved in the immune response.

However, the intrinsic mechanisms of effects of DHX37 on malignant tumor development and immune regulation have not been investigated. In this research, we conducted a comprehensive bioinformatic analysis of DHX37 expression profiles in multiple cancers. This is the first finding that reveals the association between DHX37 and Pan-Cancer in multidimensional biological functions. Our study has identified DHX37 as a potential marker of lung and liver cancers, which may guide the development of novel anticancer therapies.

## 2. Methods

### 2.1. Oncomine Database Analysis

We examined the gene expression of DHX37 in Pan-Cancer using the Oncomine database [20]. The gene was assessed for differential expression with *t*-statistics using Total Access Statistics 2002 (FMS Inc., Vienna, VA). *t*-tests were conducted both as two-sided for differential expression analysis and one-sided for specific overexpression analysis. A *P* value of 0.001 and a fold change of 1.5 were set as significance thresholds.

### 2.2. PrognoScan Database Analysis

We utilized PrognoScan database analysis [21] to assess the correlations between DHX37 expression and survival time in multiple cancers. Survival analysis in PrognoScan employs the minimum *P* value approach to find the cut point in continuous gene expression measurement for grouping patients. An adjusted Cox *P* value < 0.05 was considered statistically significant.

### 2.3. Kaplan-Meier Plotter Database Analysis

The correlation between the gene level of DHX37 and overall survival as well as relapse-free survival in 21 types of tumors was performed by Kaplan-Meier Plotter [22]. The Kaplan-Meier Plotter was set up by searching the GEO, EGA, TCGA, and PubMed repositories to identify datasets with published gene expression and clinical data. The hazard ratio (HR) with 95% confidence intervals (CI) was analyzed. The correlation was significant at the 0.05 level.

### 2.4. UALCAN Analysis

UALCAN [23] was used to obtain the relative expression of DHX37 both in normal and neoplastic tissues and in several clinicopathological subgroups. UALCAN analysis use the TCGA-Assembler to download the TCGA level 3 RNA-seq data related to 31 cancer types and used TPM as the measure of expression. The *P* value cutoff was 0.05.

### 2.5. GEPIA

Gene Expression Profiling Interactive Analysis (GEPIA) was applied to confirm the correlational analyses from TIMER [24]. GEPIA analyzed the RNA sequencing expression data of both tumor samples and normal samples from TCGA and the Genotype Tissue Expression projects. To solve the imbalance between the tumor and normal data which can cause inefficiency in various differential analyses, GEPIA downloaded the TCGA and GTEx gene expression data that are recomputed from raw RNA-seq data by the UCSC Xena project based on a uniform pipeline. The *P* value cutoff was 0.05.

### 2.6. TIMER Database Analysis

The Tumor Immune Estimation Resource aims to evaluate relative proportions of various immune cell subsets based on data from TCGA with a deconvolution approach [25]. We assessed DHX37 expression and the correlation of DHX37 expression with the density of 6 types of infiltrating immune cells in diverse cancers by the TIMER algorithm database. In addition, the “differential expression module” was used to evaluate clinical prognosis affected by overexpressed DHX37.

### 2.7. TISIDB Database Analysis

Tumor and immune system interactions [26] were performed to explore the abundance of 28 types of tumor-infiltrating lymphocytes (TILs) and MHC markers by precalculating for 30 TCGA cancer types. The relational coefficient between DHX37 and TILs as well as MHC markers was measured by Spearman's test.

### 2.8. LinkedOmics Analysis

The LinkedOmics database is a publicly available web server for analyzing multidimensional datasets based on TCGA [27]. DHX37 coexpression was presented in heat maps with analysis of Pearson's correlation coefficient. GO (CC (cellular component), BP (biological process), and MF (molecular function)) and KEGG (Kyoto Encyclopedia of Genes and Genomes) pathways and enrichment of cellular regulators including kinase targets and miRNA targets and transcription factor target were analyzed by gene set enrichment analysis (GSEA). The rank criterion was an FDR (false discovery rate) < 0.05, a minimum number of genes of 3, and a simulation of 500, using the LIHC and LUAD compared datasets.

## 3. Results

### 3.1. DHX37 Expression in Various Cancers

**Figure 1:**
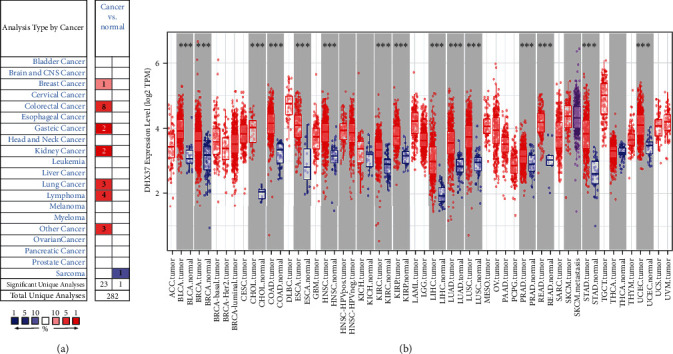
DHX37 expression levels in cancers. (a) Increased or decreased expression of DHX37 in different cancer tissues compared with normal tissues in the Oncomine database. Number in each cell is the number of datasets. Red indicates high expression and blue indicates low expression. (b) Human DHX37 expression levels in different cancer types from TCGA data in TIMER. ^∗^*P* < 0.05, ^∗∗^*P* < 0.01, ^∗∗∗^*P* < 0.001. Red indicates tumor and blue indicates normal tissue.

DHX37 gene expression was retrieved using the Oncomine database to determine differences between tumor and normal tissues over a cancer-wide range. As depicted in Figure 1(a) and Table S1, the DHX37 expression was elevated in breast (1.5, *P* = 5.45*e* − 9), colorectal (3.526, *P* = 5.14*e* − 5), gastric (3.866, *P* = 2.24*e* − 9), kidney (3.023, *P* = 5.81*e* − 4), and lung cancers (2.357, *P* = 6.95*e* − 5) as well as lymphoma (2.032, *P* = 3.03*e* − 7), whereas DHX37 was only observed significantly reduced in the sarcoma dataset (-1.632, *P* = 5.15*e* − 5).

To validate DHX37 high expression in other databases, the RNA-seq data in TCGA from TIMER were investigated. The aberrant expression of tumor masses compared with adjacent normal tissues in Pan-Cancer is shown in Figure 1(b). DHX37 expression was significantly upregulated in cancer groups, including BLCA (bladder urothelial carcinoma), BRCA (breast invasive carcinoma), CHOL (cholangiocarcinoma), COAD (colon adenocarcinoma), ESCA (esophageal carcinoma), HNSC (head and neck squamous cell carcinoma), KIRC (head and neck squamous cell carcinoma), KIRP (kidney renal papillary cell carcinoma), LIHC (liver hepatocellular carcinoma), LUAD (lung adenocarcinoma), LUSC (lung squamous cell carcinoma), PRAD (prostate adenocarcinoma), READ (rectum adenocarcinoma), STAD (stomach adenocarcinoma), and UCEC (uterine corpus endometrial carcinoma) as compared with the surrounding tissues.

### 3.2. Prognostic Value of DHX37 in Various Cancers

**Figure 2:**
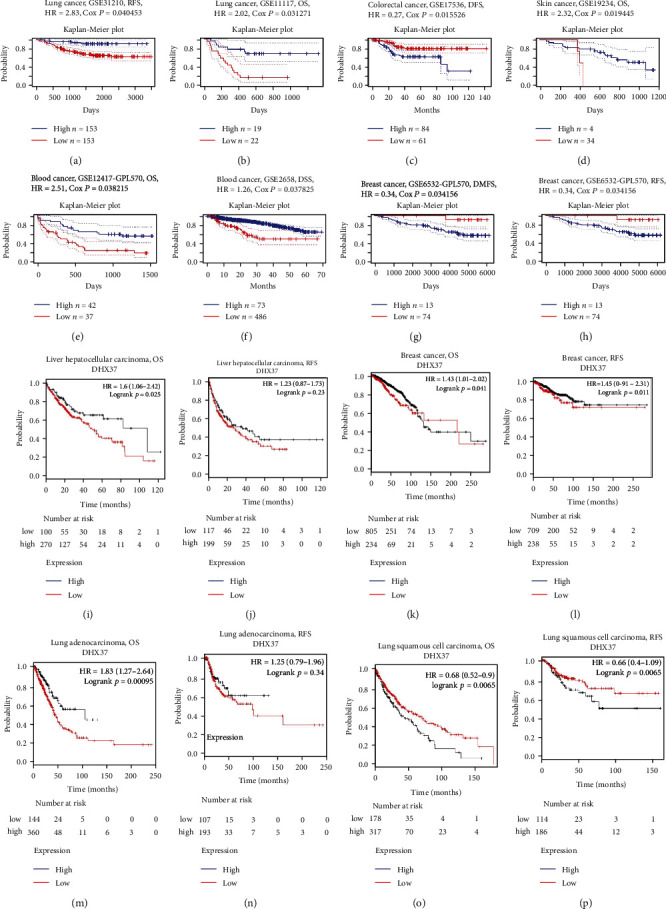
Kaplan-Meier survival curves comparing the high and low expression of DHX37 in different types of cancer in the PrognoScan (a–h) and Kaplan-Meier plotter databases (i–p). (a, b) Survival curves of RFS and OS in two lung cancer cohorts [GSE31210 (*n* = 204) and GSE11117 (*n* = 41)]. (c, d) Survival curves of DFS and OS in colorectal cancer cohort (GSE17536, *n* = 145) and skin cancer cohort (GSE19234, *n* = 38). (e, f) Survival curves of OS and DSS in two blood cancer cohorts [GSE12417-GPL570 (*n* = 79) and GSE2658 (*n* = 559)]. (g, h) Survival curves of DMSF and RFS in the breast cancer cohort (GSE6532-GPL570, *n* = 87). Kaplan-Meier survival curves comparing the high and low expression of DHX37 in Kaplan-Meier Plotter, OS, and RFS of (i, j) liver hepatocellular carcinoma (LIHC) (k, l) breast cancer (BRCA) (m, n) lung adenocarcinoma (LUAD) (o, p) lung squamous cell carcinoma (LUSC). Red curve represents patients with high expression of DHX37. OS: overall survival; DMSF: distant metastasis-free survival; DFS: disease-free survival; RFS: relapse-free survival; DSS: disease-specific survival.

The association between DHX37 and survival time from PrognoScan based on the Gene Expression Omnibus (GEO) database is summarized in Table S2. DHX37 expression significantly impacted prognosis in 5 types of cancers, including breast, colorectal, skin, blood, and lung cancers (Figures 2(a)–2(h)). Two cohorts (GSE31210 and GSE11117) comprising 204 samples and 41 samples, respectively, of lung cancer revealed that upregulated expression of DHX37 was related to poorer final outcome (RFS (relapse-free survival) HR (hazard ratio) = 2.83, 95%CI (confidence interval) = 1.05 to 7.66, Cox *P* = 0.04; OS HR = 2.02, 95%CI = 1.07 − 3.82, Cox *P* = 0.03) (Figures 2(a) and (b)). Therefore, increased DHX37predicted poor outcomes in lung cancer.

Unlike the findings from PrognoScan, we found a high expression of DHX37 reduced survival in BRCA (breast invasive carcinoma) (Figures 2(k) and 2(l)) by using the Kaplan-Meier Plotter database. Similarly, the poor outcome [28] in liver hepatocellular carcinoma (OS (overall survival) HR = 1.6, 95%CI = 1.06 to 2.42, *P* = 0.025; RFS HR = 1.23, 95%CI = 0.87 to 1.73, *P* = 0.23) and lung adenocarcinoma (OS HR = 1.83, 95%CI = 1.27 to 2.64, *P* = 0.00095; RFS HR = 1.25, 95%CI = 0.79 to 1.96, *P* = 0.34) was shown to be relevant to higher DHX37 expression (Figures 2(i), 2(j), 2(m), and 2(n)). However, DHX37 expression has less influence on head and neck squamous cell carcinoma (Figure S1C, D). For lung squamous cell carcinoma (Figures 2(o) and 2(p)), thyroid carcinoma (Figure S1E, F), rectum adenocarcinoma (Figure S1I, J), stomach adenocarcinoma (Figure S1M, N), and uterine corpus endometrial carcinoma (Figure S1O, P), DHX37 plays a protective role in their OS but not RFS. For esophageal adenocarcinoma, DHX37 was found to have a favorable effect on relapse-free survival while it worsened overall survival (Figure S1A, B). In addition, DHX37 only had significant correlation with RFS for pancreatic ductal adenocarcinoma and ovarian cancers (Figure S1G, H, S, T).

To further verify the value of differentially expressed DHX37 in the progression of different cancers, the RNA-seq data in TCGA were also exploited to confirm the prognostic implications of DHX37 in each cancer type via GEPIA (Gene Expression Profiling Interactive Analysis). We assessed correlations between DHX37 expression and clinical outcomes in 33 types of cancer (Figure S2). DHX37 overexpression was related to poor outcomes of OS (overall survival) and DFS (disease-free survival) in ACC (adrenocortical carcinoma), LGG (brain lower grade glioma), and LIHC (liver hepatocellular carcinoma); OS in LUAD (lung adenocarcinoma), MESO (mesothelioma), and THCA (thyroid carcinoma); and DFS (disease-free survival) in SKCM (Skin Cutaneous Melanoma). These results validated the predictive value of DHX37 in particular types of cancer, such as LIHC and LUAD.

### 3.3. DHX37 Expression Is Associated with Advanced Clinicopathological Characteristics in LIHC and LUAD

**Figure 3:**
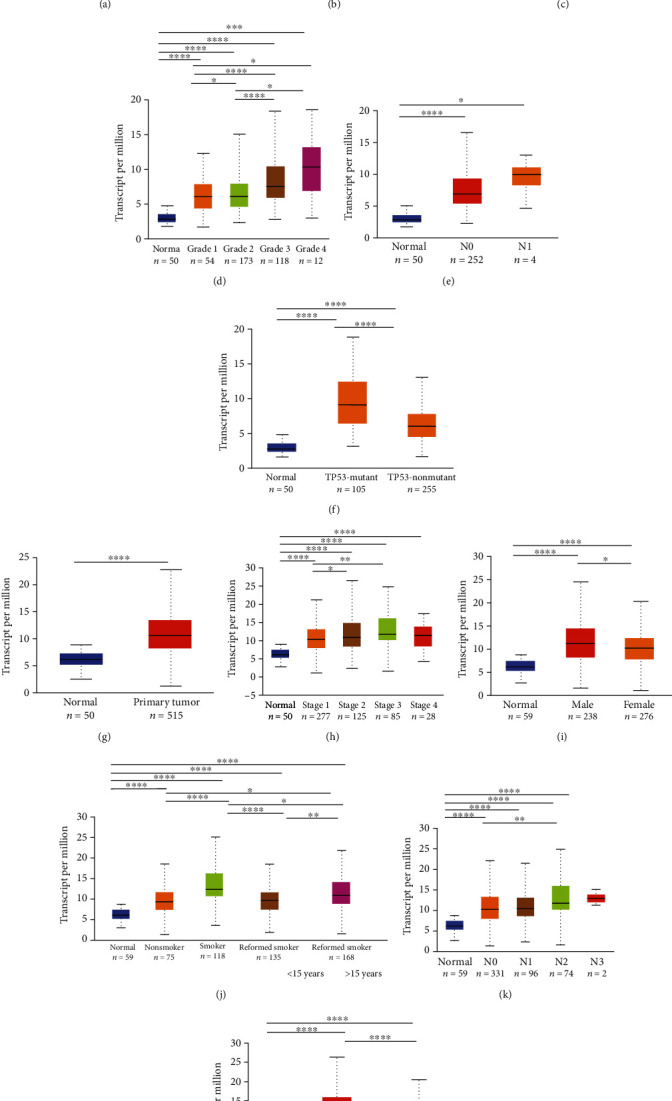
DHX37 transcription in subgroups of patients with hepatocellular carcinoma and lung adenocarcinoma, stratified based on gender, disease stages, pathological grade, tumor status, and TP53 mutation (UALCAN). (a, g) Boxplot showing relative expression of DHX37 in normal and LIHC or LUAD samples. (b, h) Boxplot showing relative expression of DHX37 in normal individuals and LIHC or LUAD patients in stages 1, 2, 3, or 4. (c, i) Boxplot showing relative expression of DHX37 in normal individuals of either gender and male or female LIHC and LUAD patients. (e, k) Boxplot showing relative expression of DHX37 in normal individuals and LIHC or LUAD patients with lymph node metastasis. (f, l) Boxplot showing relative expression of DHX37 in normal individuals and LIHC or LUAD patients with mutant TP53 or nonmutant TP53. (d) Boxplot showing relative expression of DHX37 in normal individuals or LIHC patients with grade 1, 2, 3, or 4 tumors. (j) Boxplot showing relative expression of DHX37 in normal individuals or LUAD patients with or without smoking habits. ^∗^*P* < 0.05, ^∗∗^*P* < 0.01, ^∗∗∗^*P* < 0.001, and ^∗∗∗∗^*P* < 0.0001.

To further reveal the potential relevance of DHX37 expression in cancers, we explored the relationship between DHX37 expression and several clinical features of LIHC and LUAD patients in TCGA cohorts by UALCAN. Subgroup analysis of several clinicopathological characteristics of 421 LIHC samples and 574 LUAD samples consistently showed significantly elevated DHX37 mRNA expression. As shown in Figure 3, the transcription level of DHX37 was significantly upregulated in LIHC and LUAD patients compared to the healthy group (with subgroup analysis based on gender, disease stages, pathological grade, cancer status, and TP53 mutation). Upregulated expression of DHX37 correlates with advanced stage and poor differentiation, particularly in older men and smokers.

Overall, these data analyses indicate that patients of LIHC and LUAD with high levels of DHX37 expression tend to have tumors with advanced clinicopathological parameters.

### 3.4. DHX37 Coexpression Networks in Patients with LIHC and LUAD

**Figure 4:**
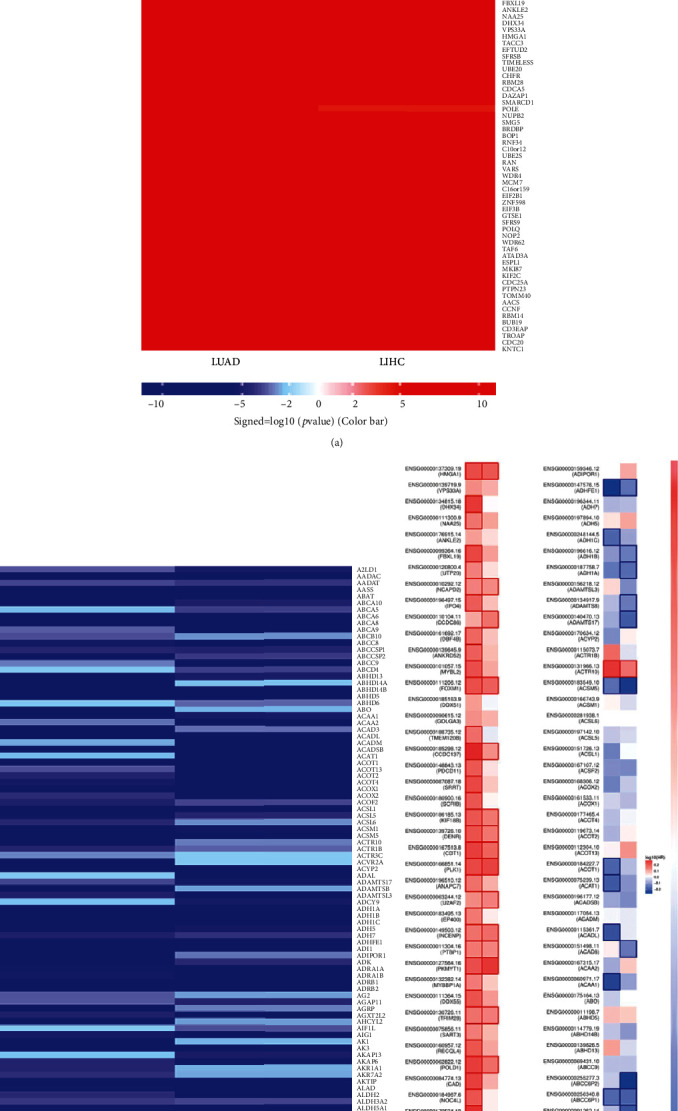
DHX37 coexpression genes in LIHC and LUAD (LinkedOmics). (a, b) Heat maps showing top 50 genes positively and negatively correlated with DHX37. (c) Survival map of the top 50 genes positively and negatively correlated with DHX37 in LIHC and LUAD.

Given the above prognostic findings from multiple databases, we chose LIHC and LUAD as representative cancers for further research. To elucidate the biological function of DHX37, LinkedOmics was applied to analyze DHX37 coexpression genes by comparing LIHC and LUAD cohorts. We found that 3682 overlap genes were positively correlated with DHX37, whereas 2002 overlap genes were negatively correlated (Table S4). This result suggests an extensive influence of DHX37 on the transcriptome. A heat map (Figures 4(a) and 4(b)) provides details about the top 50 significant (positively and negatively correlated) genes with DHX37. DHX37 expression had a strong positive association with expression of DDX54, GCN1L1, and PUS1, which reflect changes in mRNA modifications, transcriptional regulation, and DNA repair [29–31]. In line with the fact that DHX37 suppresses the immune system by modulating NF-*κ*B, DHX37 expression is positively correlated with the expression of PDCD11, which is known as the NF-*κ*B binding protein (Figure 4(a)). Notably, 43/50 and 18/50 genes in LIHC and LUAD, respectively, had high HR (*P* < 0.05) in the top 50 significantly positive genes. Instead, there were 12/50 and 13/50 genes with low HR (*P* < 0.05) in negatively significant ones (Figure 4(c)).

The functions of DHX37 were predicted by analyzing GO and KEGG by GSEA. The most highly enriched signaling pathway was determined by their normalized enrichment score (NES). As illustrated in Table 1 and Figure S3B, the biological processes, cellular components, and molecular functions strongly associated with DHX37 were cell cycle regulation, DNA replication, mRNA processing, and respiratory activity. Interestingly, GO analysis also uncovered that MHC (major histocompatibility complex), which plays a crucial role in antigen presenting in cancers, was one of the negatively correlated categories. KEGG analysis defined enrichment in DNA replication, cell cycle, ribosome biogenesis in eukaryotes, homologous recombination, Fanconi anemia pathway, notch signaling pathway, and microRNAs in cancers, while the activities like fatty acid degradation, drug metabolism, metabolism of xenobiotics by cytochrome, chemical carcinogenesis, complement and coagulation cascades, and activity of amino acid including histidine, arginine, and tyrosine were inhibited (Figure S3A). These results reveal that the functions involving cell circle modulation, amino acid metabolism, and immune activity were highly correlated with DHX37 expression.

### 3.5. Regulators of DHX37 in LIHC and LUAD

To delve further into the regulators of DHX37 in LIHC and LUAD, we analyzed the kinase, miRNA, and transcription factor derived from positively correlated gene sets. The top 5 most important target networks were the kinase-target ones related mainly to the polo-like kinase 1, checkpoint kinase 2, cyclin dependent kinase 2, ATR serine/threonine kinase, and cyclin dependent kinase 1 (Table 2). The miRNA-target network was associated with (TCCGTCC) MIR-184, (TGCACGA) MIR-517, (GTGGTGA) MIR-197, (CCAGGGG) MIR-331, and (CAGCAGG) MIR-370. The transcription factor-target network was related primarily to the E2F Transcription Factor family, including E2F1DP2_01, E2F1_Q6, E2F1DP1_01, E2F1DP2_01, and E2F4DP2_01. The gene set enriched for kinase is responsible mainly for regulating stability and integrality of the genome.

### 3.6. DHX37 Expression Impacts Immune Infiltration Level

**Figure 5:**
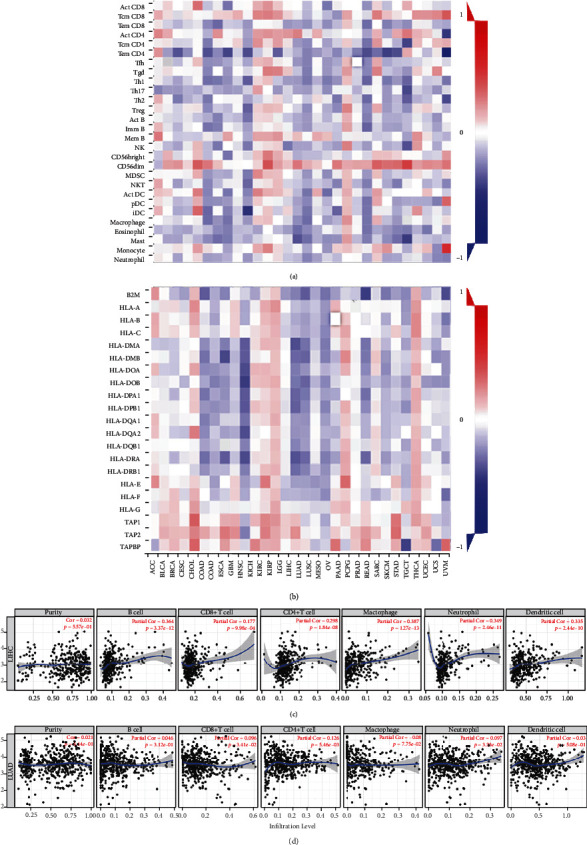
Correlations between DHX37 expression and TILs across human cancers. (a, b) Heat map showing relations between expression of DHX37 and 28 types of TILs as well as 21 types of MHC molecules. (c) DHX37 expression level has significant positive correlations with infiltrating levels of CD8+ T cells, CD4+ T cells, macrophages, neutrophils, B cells, and DCs in LIHC. (d) DHX37 expression level has significant positive correlations with infiltrating levels of CD4+ T cells and neutrophils and negative correlations with CD8+ T cells in LUAD. *P* < 0.05 is considered significant.

DHX37 is expressed in immune cells and TILs (tumor infiltrating lymphocytes). These cells serve as independent prognostic factors of clinicopathological parameters and outcome in cancers [32]. Hence, we analyzed the correlation between DHX37 expression and immunophenotypic characteristics in Pan-Cancer from TIMER and TISIDB (tumor and immune system interaction). The comprehensive analysis indicated that the DHX37 expression had significant correlations with tumor purity in 16 types of cancers. Moreover, DHX37 expression also significantly correlated with CD8 T cells, CD4 T cells, B cells, macrophages, neutrophils, and dendritic cells (DCs) in 16, 19, 12, 18, 16, and 14 types of cancer, respectively (Table S3 and Figure S4). Furthermore, we also found that DHX37 expression weakly to moderately negatively correlated with 28 types of TILs and MHC expression across all human heterogeneous cancers, except in KIRP (kidney renal papillary cell carcinoma), THCA (thyroid carcinoma), and LGG (brain lower grade glioma) (Figures 5(a) and 5(b)).

On the basis of the findings in immune infiltration landscapes, we further found that DHX37 expression level correlates with poorer clinical outcomes and specific immune cell infiltration in LIHC and LUAD (Figures 5(c) and 5(d)). The DHX37 expression level has significant positive correlations with infiltrating levels of CD8 T cells (*r* = 0.177, *P* = 9.98*e* − 04), CD4T cells (*r* = 0.298, *P* = 1.84*e* − 08), macrophages (*r* = 0.387, *P* = 1.27*e* − 13), neutrophils (*r* = 0.349, *P* = 2.46*e* − 11), B cells (*r* = 0.364, *P* = 3.37*e* − 12), and DCs (dendritic cells) (*r* = 0.335, *P* = 2.44*e* − 10) in LIHC. In LUAD, except for CD8 T cells (*r* = −0.096, *P* = 3.41*e* − 02), DHX37 expression positively correlated with infiltration levels of CD4 T cells (*r* = 0.126, *P* = 5.46*e* − 03) and neutrophils (*r* = 0.097, *P* = 3.33*e* − 02). Although DHX37 expression has no significant correlations with tumor purity in both LIHC (*r* = 0.032, *P* = 5.57*e* − 01) and LUAD (*r* = 0.021, *P* = 5.44*e* − 01), these findings strongly suggest that DHX37 may participate in immune response to affect patient survival in cancers like LIHC and LUAD.

### 3.7. Correlation Analysis between DHX37 Expression and Immune Markers

To broaden our understanding of DHX37 crosstalk with immune signatures, we assessed the correlations between DHX37 expression and immune marker genes of CD8+ T cells, T cells (general), B cells, monocytes, tumor associated macrophages (TAMs), M1 and M2 macrophages, neutrophils, NK cells, myeloid-derived suppressor cells (MDSCs), cancer-associated fibroblasts (CAFs), and DCs in LIHC and LUAD (Table 3). We also analyzed the different functional T cells, including Th1 cells, Th2 cells, T_regs_, and exhausted T cells. In TIMER, after adjustments for tumor purity, the DHX37 expression level was significantly correlated with 59 out of 72 immune cell markers in LIHC and 38 out of 72 in LUAD. In LIHC, B cells, macrophages, and various types of T cells were strongly correlated with DHX37 expression (Table 3) and less significant in LUAD. Moreover, we found that expression of DHX37 positively correlates with markers of CAFs including ACTA2 (*r* = 0.13, *P* = 0.0098), FAP (*r* = 0.32, *P* = 4.5*e* − 10), PDGFR (*r* = 0.22, *P* = 1.5*e* − 05), and S100A4 (*r* = 0.21, *P* = 3.4*e* − 05) in LIHC.

**Figure 6:**
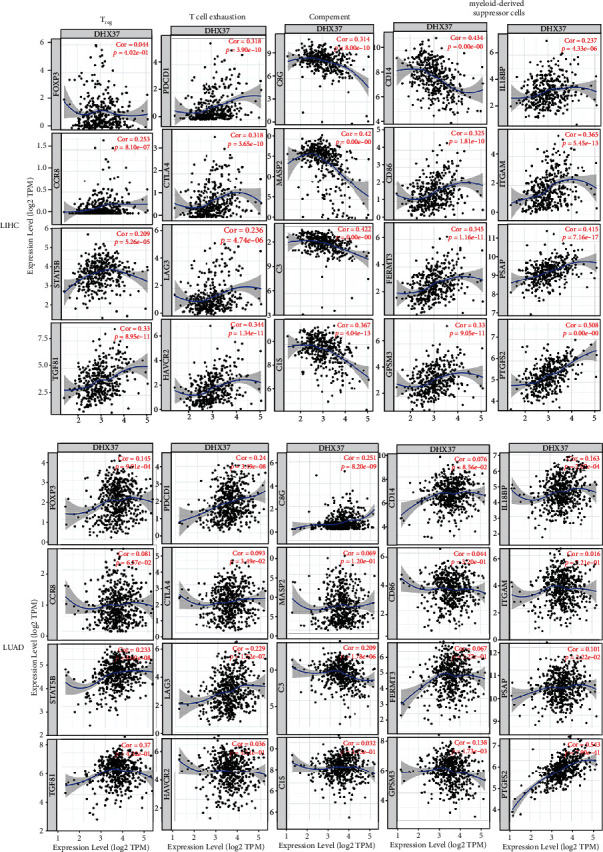
DHX37 expression correlates with T_regs_, T cell exhaustion, complement, and myeloid-derived suppressor cells in LIHC and LUAD. Markers include FOXP3, CCR8, STAT5B and TGFB1 of T_regs_, PDCD1, CTLA4, LAG3 of HAVCR2 of T cell exhaustion, CD14, CD86, FERMT3, GPSM3, IL18BP, ITGAM, PSAP and PTGES2 of myeloid-derived suppressor cells, C8G, MASP2, C3, and C1S of complement. *P* < 0.05 is considered significant.

The expression levels of most marker set of T_regs_ and exhausted T cells, such as forkhead box P3 (FOXP3), C-C chemokine receptor type 8 (CCR8), signal transducer and activator of transcription 5B (STAT5B), PD-1, CTLA4, and LAG3, have strong positive correlations with DHX37 expression in LIHC and LUAD (Figure 6). FOXP3 regulates the immune suppression and is a strong prognostic factor for distant metastases [33]. PD-1, a widely known marker related to T lymphocyte function, showed strong positive correlation with DHX37 expression, indicating that high DHX37 expression itself may be a novel predictor for immunotherapy response. In addition, DHX37 expression showed strong negative correlations with complement and strong positive correlations with markers of myeloid-derived suppressor cells. We further evaluated the correlation between DHX37 expression and the above strong significant markers in GEPIA (Table 3). Correlation results between DHX37 and the above markers are similar to this in TIMER. These findings further confirm that DHX37 plays a vital role in cancer immune escape.

## 4. Discussion

DHX37, a member of the DEAH box family of RNA helicases [34], plays indispensable roles in many aspects of gene expression [35]. Differential expression and dysfunction of RNA helicases have been reported in various cancers [36–39]. Although DHX37 functions have not been extensively elucidated, it was once discovered that it suppressed T cell activation in breast cancer [19]. As elucidated in other research, LAYN was identified as a prognostic biomarker and is highly correlated with immune infiltrates in gastric and colon cancers [40]. Therefore, it might reasonably be assumed that DHX37 expression may influence patients' clinical outcomes through immune infiltration. Here, we report that aberrant expression level of DHX37 correlated to prognosis in multiple cancers. Overexpression of DHX37 predicted higher rates of recurrence and shorter survival times in LIHC and LUAD. Interestingly, increased levels of DHX37 expression were related to advanced clinicopathological characteristics, indicating that DHX37 can be used as a prognostic indicator of cancer stages and metastasis. In addition, this study found that in liver hepatocellular carcinoma and lung adenocarcinoma, immune infiltration levels and various immune marker sets were correlated with the level of DHX37 expression. Therefore, this study revealed the potential usage of DHX37 expression as a novel predictive biomarker.

Notably, DHX37 expression is related to various immune infiltration levels in cancers, especially in liver cancer and lung adenocarcinoma (Tables 3 and 4 and Figures 5 and 6). Surprisingly, the expression level of DHX37 in LIHC and LUAD is not correlated with tumor purity, indicating that the expression of DHX37 in cancer cells and the tumor microenvironment is equally important. Our results suggest that there is weak to moderate positive correlations between DHX37 expression and infiltration levels of CD4+ T cells and neutrophils in LIHC and LUAD (Figures 5 and 6). Moreover, gene markers of T_regs_, T cell exhaustion, and MDSCs (myeloid-derived suppressor cells) showed significantly strong positive correlation with DHX37 expression, which gives clues about DHX37 function in modulating tumor immunology in LIHC and LUAD. Furthermore, significantly negative correlations can be found between DHX37 expression and several markers of complement. Immunotherapy based on checkpoint inhibitors yields significant clinical benefit for multiple cancers. However, PD-1 inhibition meets resistance partially resulting from the accumulation of MDSCs [41] or T_regs_ [42]. MDSCs, comprising macrophages, granulocytes, dendritic cells, and immature myeloid cells [43], were initially identified leading to tumor persistence and metastasis [44]. A set of microRNAs was associated with MDSCs and resistance to immunotherapy [45], which was consistent with our results that show expression of DHX37 induces microRNA in cancer (Figure S3A). C3 is an important component of the complement system [46]. Recent studies find that the complement activates and functions within cells [47], and that this takes effect in the induction of key metabolic pathways [48] and the regulation of cell death. Whether the complement fights cancer [49] or promotes the development of cancer [50] or both is yet undetermined raising the possibility that the function of complement depends on the type of cancer. A recent study [51] showed that C3d induced the antitumor immunological effect by increasing infiltrating CD8+ T cells, by decreasing T_regs_ and by suppressing expression of PD-1. Interestingly, other complement components, like anaphylatoxin C5a, contributed to cancer progression by promoting an immunosuppressive microenvironment in which MDSCs are involved [52, 53]. After adjustment for tumor purity, DHX37 in LIHC and LUAD showed negative correlations with C3 and other complement regulators but not significantly correlated with C5. Our results substantiate that DHX37 overexpression has far-reaching effects in the immune response and complement systems in cancers, which may ultimately affect patient clinical outcomes.

To probe regulators potentially responsible for DHX37, we performed enrichment analyses of target gene sets, which illustrated that DHX37 participated primarily in the spliceosome, ribosome, DNA replication, and cell cycle (Table 2). The aberrant expression of cell cycle regulatory factors in tumor cells leads to rapid multiplication and decreased apoptosis. The E2F family, always related to the progression of liver cancer [54], served as the main transcription factors for DHX37 dysregulation. In research into cancer biology and molecular pathways, we also found DHX37 expression was correlated with metabolic changes including inhibiting fatty acid degradation, amino acid (arginine, histidine valine, leucine, and isoleucine), metabolism, and mitochondrial function. These findings were in line with the molecular pathways illustrated in liver cancer oncogenes [55]. In addition, DHX37 is positively correlated with notch signaling that regulates cell proliferation, differentiation, and survival [56]. The notch pathway is an important target for many types of solid cancers [57]. The functional effects of DHX37 on cancer cell proliferation and survival of liver cancer and lung adenocarcinoma are probably partially modulated by notch signaling. Future studies may develop compounds targeting DHX37 for precision medicine in cancers.

However, even though we utilized online tools based on widely used bioinformatic theories from public databases, this study still had one major limitation. We only performed a bioinformatic analysis of DHX37 expression without further confirmation using *in vivo/in vitro* experiments. Future prospective studies focusing on these aspects in a comprehensive manner could help identify the function of DHX37 in cancers.

In conclusion, DHX37 can impact cancer prognosis by not only playing direct regulatory roles in cancer cells but also affecting the immune microenvironment. Based on multilevel evidence, DHX37 plays an oncogenic role and induces a suppressive tumor microenvironment in LIHC and LUAD. These findings for the first time offer evidence that DHX37 serves as an immunobased potential therapeutic target for cancer treatment.

## Figures and Tables

**Table 1:** Signaling pathways most significantly correlated with DHX37 expression based on their normalized enrichment score (NES) and *P* value.

	GO name (BP)	NES	*P* value	FDR
Positive	Protein localization to chromosome	1.672	≤0.001	0.008
	CENP-A containing chromatin organization	1.665	≤0.001	0.004
	rRNA metabolic process	1.640	≤0.001	0.006
	Chromosome localization	1.635	≤0.001	0.005
	Chromosome segregation	1.634	≤0.001	0.004
	DNA replication	1.627	≤0.001	0.004
Negative	Peroxisome organization	-1.916	≤0.001	0.107
	Benzene-containing compound metabolic process	-1.809	≤0.001	0.137
	Peroxisomal transport	-1.729	≤0.001	0.151
	Mitochondrial respiratory chain complex assembly	-1.721	≤0.001	0.119
	GO name (CC)	NES	*P* value	FDR
Positive	Preribosome	2.404	≤0.001	0.000
	Condensed chromosome	2.309	≤0.001	0.000
	Replication fork	2.209	≤0.001	0.000
	Chromosomal region	2.173	≤0.001	0.000
	Heterochromatin	2.162	≤0.001	0.000
Negative	NADH dehydrogenase complex	-2.478	≤0.001	0.000
	MHC protein complex	-2.371	≤0.001	0.000
	Respiratory chain	-2.251	≤0.001	0.000
	Platelet dense granule	-2.212	≤0.001	0.000
	Basal part of cell	-2.042	≤0.001	0.007
	GO name (MF)	NES	*P* value	FDR
Positive	snoRNA binding	2.247	≤0.001	0.000
	tRNA binding	2.233	≤0.001	0.000
	Helicase activity	2.204	≤0.001	0.000
	Catalytic activity, acting on DNA	2.161	≤0.001	0.000
	Methyl-CpG binding	2.038	≤0.001	0.000
Negative	Monooxygenase activity	-2.317	≤0.001	0.009
	Oxidoreductase activity, acting on NAD(P)H	-2.104	≤0.001	0.009
	Steroid dehydrogenase activity	-2.089	≤0.001	0.006
	Tetrapyrrole binding	-2.075	≤0.001	0.005
	Oxidoreductase activity, acting on peroxide as acceptor	-2.051	≤0.001	0.009

**Table 2:** The kinase, miRNA, and transcription factor-target networks of DHX37 in LIHC and LUAD (LinkedOmics).

Enriched category	Gene set	LeadingEdgeNum	FDR
Kinase target	Polo-like kinase 1	45	0.000
	Checkpoint kinase 2	17	0.000
	Cyclin dependent kinase 2	139	0.000
	ATR serine/threonine kinase	42	0.000
	Cyclin dependent kinase 1	108	0.000
miRNA target	TCCGTCC, MIR-184	4	0.164
	TGCACGA, MIR-517A/C	13	0.097
	GTGGTGA, MIR-197	34	0.104
	CCAGGGG, MIR-331	32	0.080
	CAGCAGG, MIR-370	51	0.078
Transcription factor target	SGCGSSAAA_V$E2F1DP2_01	74	0.000
	V$E2F1_Q6	95	0.000
	V$E2F1DP1_01	95	0.000
	V$E2F1DP2_01	95	0.000
	V$E2F4DP2_01	95	0.000

LeadingEdgeNum: the number of leading-edge genes; FDR: false discovery rate from Benjamini and Hochberg from gene set enrichment analysis (GSEA); V$: the annotation found in the Molecular Signatures Database for transcription factors.

**Table 3:** Correlation analysis between DHX37 and related genes and markers of immune cells in TIMER.

	Markers	LIHC				LUAD			
		None		Purity		None		Purity	
		Cor	*P*	Cor	*P*	Cor	*P*	Cor	*P*
CD8+ T cell	CD8A	0.138	∗∗	0.173	∗∗	0.063	0.160	0.081	0.074
	CD8B	0.140	∗∗	0.169	∗∗	0.056	0.212	0.068	0.133
T cell (general)	CD3D	0.263	∗∗∗∗	0.310	∗∗∗∗	-0.067	0.140	-0.065	0.148
	CD3E	0.174	∗∗	0.231	∗∗∗∗	-0.020	0.658	-0.010	0.817
	CD2	0.176	∗∗	0.225	∗∗∗∗	-0.058	0.199	-0.055	0.222
B cell	CD19	0.177	∗∗∗	0.199	∗∗∗	0.025	0.576	0.041	0.366
	CD79A	0.090	0.096	0.120	∗	-0.010	0.821	-0.001	0.986
Monocyte	CD86	0.314	∗∗∗∗	0.386	∗∗∗∗	-0.041	0.358	-0.036	0.427
	CSF1R	0.256	∗∗∗∗	0.322	∗∗∗∗	0.034	0.449	0.046	0.307
TAM	CCL2	0.078	0.145	0.112	∗	-0.009	0.845	-0.002	0.965
	CD68	0.291	∗∗∗∗	0.340	∗∗∗∗	0.075	0.097	0.088	0.051
	IL10	0.256	∗∗∗∗	0.307	∗∗∗∗	-0.029	0.527	-0.022	0.629
M1 macrophage	NOS2	-0.071	0.186	-0.069	0.202	0.173	∗∗∗	0.183	∗∗∗∗
	IRF5	0.393	∗∗∗∗	0.393	∗∗∗∗	0.124	∗∗	0.139	∗∗
	PTGS2	0.111	∗	0.147	∗∗	0.038	0.397	0.039	0.392
M2 macrophage	CD163	0.132	∗	0.168	∗∗	0.117	∗∗	0.136	∗∗
	VSIG4	0.162	∗∗	0.203	∗∗∗	-0.050	0.264	-0.046	0.308
	MS4A4A	0.140	∗∗	0.182	∗∗∗	-0.101	∗	-0.102	∗
Neutrophils	CEACAM8	0.044	0.414	0.048	0.377	-0.199	∗∗∗∗	-0.198	∗∗∗∗
	ITGAM	0.370	∗∗∗∗	0.403	∗∗∗∗	0.029	0.524	0.039	0.388
	CCR7	0.014	0.797	0.038	0.486	-0.082	0.070	-0.083	0.065
Natural killer cell	KIR2DL1	-0.036	0.508	-0.034	0.524	0.065	0.150	0.069	0.127
	KIR2DL3	0.150	∗∗	0.159	∗∗	0.121	∗∗	0.129	∗∗
	KIR2DL4	0.149	∗∗	0.157	∗∗	0.286	∗∗∗∗	0.300	∗∗∗∗
	KIR3DL1	0.050	0.357	0.053	0.324	0.057	0.205	0.062	0.166
	KIR3DL2	0.081	0.132	0.090	0.094	0.168	∗∗∗	0.178	∗∗∗∗
	KIR3DL3	0.024	0.652	0.026	0.631	0.119	∗∗	0.120	∗∗
	KIR2DS4	0.052	0.336	0.053	0.325	0.059	0.189	0.064	0.155
Dendritic cell	HLA-DPB1	0.154	∗∗	0.195	∗∗∗	-0.247	∗∗∗∗	-0.259	∗∗∗∗
	HLA-DQB1	0.141	∗∗	0.174	∗∗	-0.168	∗∗∗	-0.172	∗∗∗
	HLA-DRA	0.154	∗∗	0.193	∗∗∗	-0.299	∗∗∗∗	-0.316	∗∗∗∗
	HLA-DPA1	0.152	∗∗	0.191	∗∗∗	-0.234	∗∗∗∗	-0.244	∗∗∗∗
	CD1C	0.102	0.058	0.128	∗	-0.384	∗∗∗∗	-0.393	∗∗∗∗
	NRP1	0.239	∗∗∗∗	0.251	∗∗∗∗	0.032	0.472	0.035	0.442
	ITGAX	0.377	∗∗∗∗	0.442	∗∗∗∗	0.138	∗∗	0.164	∗∗∗
Th1	TBX21	0.073	0.176	0.096	0.074	0.090	∗	0.112	∗
	STAT4	0.165	∗∗	0.180	∗∗∗	-0.030	0.504	-0.023	0.608
	STAT1	0.278	∗∗∗∗	0.290	∗∗∗∗	0.301	∗∗∗∗	0.326	∗∗∗∗
	IFNG	0.255	∗∗∗∗	0.277	∗∗∗∗	0.158	∗∗∗	0.177	∗∗∗∗
	TNF	0.276	∗∗∗∗	0.322	∗∗∗∗	0.058	0.199	0.072	0.109
Th2	GATA3	0.137	∗	0.177	∗∗∗	0.095	∗	0.113	∗
	STAT6	0.185	∗∗∗	0.185	∗∗∗	0.091	∗	0.091	∗
	STAT5A	0.334	∗∗∗∗	0.353	∗∗∗∗	0.110	∗	0.130	∗∗
	IL13	-0.022	0.677	-0.022	0.683	0.011	0.804	0.016	0.724
Tfh	BCL6	0.209	∗∗∗∗	0.209	∗∗∗∗	0.078	0.084	0.079	0.081
Th17	STAT3	0.160	∗∗	0.172	∗∗	0.144	∗∗	0.143	∗∗
	IL17A	0.069	0.203	0.070	0.195	0.047	0.299	0.053	0.240
T_reg_	FOXP3	0.057	0.295	0.065	0.226	0.152	∗∗∗	0.184	∗∗∗∗
	CCR8	0.253	∗∗∗∗	0.278	∗∗∗∗	0.098	∗	0.117	∗∗
	STAT5B	0.208	∗∗∗∗	0.206	∗∗∗	0.246	∗∗∗∗	0.250	∗∗∗∗
	TGFB1	0.335	∗∗∗∗	0.381	∗∗∗∗	0.040	0.373	0.049	0.277
T cell exhaustion	PDCD1	0.304	∗∗∗∗	0.351	∗∗∗∗	0.242	∗∗∗∗	0.279	∗∗∗∗
	CTLA4	0.310	∗∗∗∗	0.355	∗∗∗∗	0.101	∗	0.129	∗∗
	LAG3	0.220	∗∗∗∗	0.234	∗∗∗∗	0.221	∗∗∗∗	0.247	∗∗∗∗
	HAVCR2	0.337	∗∗∗∗	0.411	∗∗∗∗	-0.033	0.470	-0.026	0.566
	GZMB	0.085	0.113	0.103	0.057	0.275	∗∗∗∗	0.308	∗∗∗∗
Myeloid-derived suppressor cells	CD14	-0.435	∗∗∗∗	-0.434	∗∗∗∗	0.078	0.084	0.093	∗
	CD86	0.314	∗∗∗∗	0.386	∗∗∗∗	-0.041	0.358	-0.036	0.427
	FERMT3	0.328	∗∗∗∗	0.413	∗∗∗∗	0.077	0.088	0.100	∗
	GPSM3	0.316	∗∗∗∗	0.408	∗∗∗∗	-0.127	∗∗	-0.127	∗∗∗
	IL18BP	0.215	∗∗∗∗	0.281	∗∗∗∗	0.160	∗∗∗	0.187	∗∗∗∗
	ITGAM	0.370	∗∗∗∗	0.403	∗∗∗∗	0.029	0.524	0.039	0.388
	PSAP	0.417	∗∗∗∗	0.439	∗∗∗∗	0.109	∗	0.114	∗
	PTGES2	0.496	∗∗∗∗	0.496	∗∗∗∗	0.538	∗∗∗∗	0.538	∗∗∗∗
Complement	CFD	0.227	∗∗∗∗	0.257	∗∗∗∗	-0.181	∗∗∗∗	-0.181	∗∗∗∗
	MBL2	-0.230	∗∗∗∗	-0.228	∗∗∗∗	-0.037	0.413	-0.036	0.429
	C2	-0.140	∗∗	-0.147	∗∗	-0.013	0.774	-0.011	0.813
	C5	-0.148	∗∗	-0.146	∗∗	0.028	0.537	0.027	0.552
	C8G	-0.309	∗∗∗∗	-0.307	∗∗∗∗	0.244	∗∗∗∗	0.244	∗∗∗∗
	MASP2	-0.424	∗∗∗∗	-0.424	∗∗∗∗	0.067	0.135	0.069	0.124
	C3	-0.425	∗∗∗∗	-0.426	∗∗∗∗	-0.202	∗∗∗∗	-0.202	∗∗∗∗
	C1S	-0.362	∗∗∗∗	-0.361	∗∗∗∗	0.039	0.388	0.055	0.225

^∗^ *P* < 0.05, ^∗∗^*P* < 0.01, ^∗∗∗^*P* < 0.001, and ^∗∗∗∗^*P* < 0.0001.

**Table 4:** Correlation between DHX37 and gene markers of complement, T_regs_, T cell exhaustion, and myeloid-derived suppressor cells.

Cell type	Gene marker	LIHC				LUAD			
		Tumor		Normal		Tumor		Normal	
		*R*	*P*	*R*	*P*	*R*	*P*	*R*	*P*
Complement	CFD	0.088	0.093	0.48	∗∗∗	-0.095	∗	0.044	0.74
	C2	-0.15	∗∗	0.32	∗	-0.069	0.13	0.29	∗
	C5	-0.067	0.2	0.28	0.052	0.061	0.18	0.23	0.076
	C8G	-0.28	∗∗∗∗	0.21	0.14	0.17	∗∗∗	0.12	0.38
	MASP2	-0.35	∗∗∗∗	0.079	0.59	0.12	∗∗	0.41	∗∗
	C4B	-0.24	∗∗∗∗	-0.017	0.91	-0.11	∗	0.25	0.056
	C3	-0.4	∗∗∗∗	-0.31	∗	-0.16	∗∗∗	0.17	0.2
	C1S	-0.25	∗∗∗∗	0.36	∗	0.042	0.36	0.31	∗
T_reg_	FOXP3	-0.017	0.75	0.27	0.055	0.12	∗	0.4	∗∗
	CCR8	0.21	∗∗∗∗	0.31	∗	0.12	∗∗	0.22	0.088
	STAT5B	0.23	∗∗∗∗	0.69	∗∗∗∗	0.32	∗∗∗∗	0.59	∗∗∗∗
	TGFB1	0.34	∗∗∗∗	0.61	∗∗∗∗	0.093	∗	0.64	∗∗∗∗
T cell exhaustion	PDCD1	0.18	∗∗∗	0.42	∗∗	0.26	∗∗∗∗	0.32	∗
	CTLA4	0.21	∗∗∗∗	0.3	∗	0.16	∗∗∗	0.26	∗
	LAG3	0.21	∗∗∗∗	0.2	0.17	0.19	∗∗∗∗	0.23	0.084
	HAVCR2	0.24	∗∗∗∗	0.43	∗∗	0.017	0.71	-0.039	0.77
	GZMB	0.15	∗∗	0.49	∗∗∗	0.25	∗∗∗∗	-0.069	0.6
Myeloid-derived suppressor cells	CD14	-0.28	∗∗∗∗	0.24	0.094	0.11	∗	-0.081	0.54
	CD86	0.38	∗∗∗∗	0.46	∗∗∗	0.0043	0.93	-0.066	0.62
	FERMT3	0.3	∗∗∗∗	0.45	∗∗	0.065	0.15	0.28	∗
	GPSM3	0.27	∗∗∗∗	0.48	∗∗∗	-0.098	∗	0.36	∗∗
	IL18BP	0.17	∗∗	0.57	∗∗∗∗	0.2	∗∗∗∗	0.042	0.75
	ITGAM	0.33	∗∗∗∗	0.63	∗∗∗∗	0.12	∗	0.44	∗∗∗
	PSAP	0.46	∗∗∗∗	0.62	∗∗∗∗	0.22	∗∗∗∗	0.22	0.1
	PTGES2	0.46	∗∗∗∗	0.67	∗∗∗∗	0.49	∗∗∗∗	0.59	∗∗∗∗
CAFs	ACTA2	0.13	∗∗	0.51	∗∗∗∗	0.007	0.88	0.058	0.66
	FAP	0.32	∗∗∗∗	0.45	∗∗∗	0.038	0.41	0.22	0.1
	PDGFR	0.22	∗∗∗∗	0.52	∗∗∗∗	0.087	0.055	0.37	∗∗
	S100A4	0.21	∗∗∗∗	0.35	∗	-0.15	∗∗∗	-0.33	∗

^∗^ *P* < 0.05, ^∗∗^*P* < 0.01, ^∗∗∗^*P* < 0.001, ^∗∗∗∗^*P* < 0.0001.

## Data Availability

The datasets analyzed for this study can be found in the Oncomine, PrognoScan, GEPIA, Kaplan-Meier Plotter, UALCAN, TIMER, TISIDB, and LinkedOmics web resources.

## Abbreviations

OS: Overall survival
DFS: Disease-free survival
RFS: Relapse-free survival
HR: Hazard ratio
MDSCs: Myeloid-derived suppressor cells
TCGA: The Cancer Genome Atlas
TIMER: Tumor Immune Estimation Resource
GEPIA: Gene Expression Profiling Interactive Analysis
TISIDB: Tumor and immune system interaction
GO: Gene Ontology
CC: Cellular component
BP: Biological process
MF: Molecular function
KEGG: Kyoto Encyclopedia of Genes and Genomes
GSEA: Gene set enrichment analysis
FDR: False discovery rate
ACC: Adrenocortical carcinoma
BLCA: Bladder urothelial carcinoma
BRCA: Breast invasive carcinoma
CESC: Cervical squamous cell carcinoma
CHOL: Cholangiocarcinoma
COAD: Colon adenocarcinoma
ESCA: Esophageal carcinoma
GBM: Glioblastoma multiforme
HNSC: Head and neck squamous cell carcinoma
KICH: Kidney chromophobe
KIRC: Kidney renal clear cell carcinoma
KIRP: Kidney renal papillary cell carcinoma
AML: Acute myeloid leukemia
LGG: Brain lower grade glioma
LIHC: Liver hepatocellular carcinoma
LUAD: Lung adenocarcinoma
LUSC: Lung squamous cell carcinoma
MESO: Mesothelioma
OV: Ovarian serous cystadenocarcinoma
PAAD: Pancreatic adenocarcinoma
PCPG: Pheochromocytoma and paraganglioma
PRAD: Prostate adenocarcinoma
READ: Rectum adenocarcinoma
SARC: Sarcoma
SKCM: Skin cutaneous melanoma
STAD: Stomach adenocarcinoma
STES: Stomach and esophageal carcinoma
THCA: Thyroid carcinoma
THYM: Thymoma
UCEC: Uterine corpus endometrial carcinoma
UCS: Uterine carcinosarcoma.

## Supplementary Materials

Supplementary Materials Per the publisher's request, the details of the supplementary materials are added here. However, the editable version of each table and figure is given in a separate folder, as requested. Table S1: DHX37 expression in cancers versus normal tissue in the Oncomine database. The DHX37 expression was elevated in breast, colorectal, gastric, kidney, lung cancers as well as lymphoma, whereas DHX37 was only observed significantly reduced in the sarcoma dataset. Table S2: relationship between DHX37 expression and patient prognosis of different cancers in the PrognoScan database. Table S3: DHX37 cooccurrence genes shown in the PDF file. We found that 3682 overlap genes were positively correlated with DHX37, whereas 2002 overlap genes were negatively correlated. Table S4: summarization of correlation between DHX37 expression and immune infiltration level in diverse type cancers via the TIMER database. N: not significantly; ^∗^*P* < 0.05, ^∗∗^*P* < 0.01, ^∗∗∗^*P* < 0.001, and ^∗∗∗∗^*P* < 0.0001. DHX37 expression showed significantly correlated with CD8 T cells, CD4 T cells, B cells, macrophages, neutrophils, and dendritic cells in 16, 19, 12, 18, 16, and 14 types of cancer, respectively. Figure S1: correlation of DHX37 expression with diverse types of cancer via Kaplan-Meier Plot. For esophageal adenocarcinoma, DHX37 was found to have a favorable effect on relapse-free survival while worsening overall survival. For head and neck squamous cell carcinoma, DHX37 expression has less influence. For thyroid carcinoma, rectum adenocarcinoma, stomach adenocarcinoma, and uterine corpus endometrial carcinoma, DHX37 plays a protective role in their OS but not RFS. DHX37 only had significant correlation with RFS for pancreatic ductal adenocarcinoma and ovarian cancer. Figure S2: correlation of DHX37 expression with diverse types of cancer via GEPIA. Overall survival and disease-free survival comparing the high and low expression of DHX37 in various cancers. DHX37 overexpression was related to worsening outcome of OS and DFS in ACC, LGG, and LIHC; OS in LUAD, MESO, and THCA; and DFS in SKCM. These results validated the predictive value of DHX37 in particular types of cancer, such as LIHC and LUAD. Figure S3: KEGG pathways and GO of DHX37 in the LIHC and LUAD cohort. These results reveal that the functions involving cell circle modulation, amino acid metabolism, and immune activity were highly correlated with DHX37 expression. Figure S4: correlation of the DHX37 expression with immune infiltration level in various cancers via the TIMER database. Diverse type of cancers via the TIMER database. DHX37 expression was shown as significantly correlated with CD8 T cells, CD4 T cells, B cells, macrophages, neutrophils, and dendritic cells in 16, 19, 12, 18, 16, and 14 types of cancer, respectively.
