# Supplementary material for: DHX37 Impacts Prognosis of Hepatocellular Carcinoma and Lung Adenocarcinoma through Immune Infiltration
Source: J Immunol Res. 2020 Dec 30;2020:8835393. doi: 10.1155/2020/8835393 (PMC7790560; doi:10.1155/2020/8835393)
Supplement: Supplementary Materials — Per the publisher's request, the details of the supplementary materials are added here. However, the editable version of each table and figure is given in a separate folder, as requested. Table S1: DHX37 expression in cancers versus normal tissue in the Oncomine database. The DHX37 expression was elevated in breast, colorectal, gastric, kidney, lung cancers as well as lymphoma, whereas DHX37 was only observed significantly reduced in the sarcoma dataset. Table S2: relationship between DHX37 expression and patient prognosis of different cancers in the PrognoScan database. Table S3: DHX37 cooccurrence genes shown in the PDF file. We found that 3682 overlap genes were positively correlated with DHX37, whereas 2002 overlap genes were negatively correlated. Table S4: summarization of correlation between DHX37 expression and immune infiltration level in diverse type cancers via the TIMER database. N: not significantly; ∗P < 0.05, ∗∗P < 0.01, ∗∗∗P < 0.001, and ∗∗∗∗P < 0.0001. DHX37 expression showed significantly correlated with CD8 T cells, CD4 T cells, B cells, macrophages, neutrophils, and dendritic cells in 16, 19, 12, 18, 16, and 14 types of cancer, respectively. Figure S1: correlation of DHX37 expression with diverse types of cancer via Kaplan-Meier Plot. For esophageal adenocarcinoma, DHX37 was found to have a favorable effect on relapse-free survival while worsening overall survival. For head and neck squamous cell carcinoma, DHX37 expression has less influence. For thyroid carcinoma, rectum adenocarcinoma, stomach adenocarcinoma, and uterine corpus endometrial carcinoma, DHX37 plays a protective role in their OS but not RFS. DHX37 only had significant correlation with RFS for pancreatic ductal adenocarcinoma and ovarian cancer. Figure S2: correlation of DHX37 expression with diverse types of cancer via GEPIA. Overall survival and disease-free survival comparing the high and low expression of DHX37 in various cancers. DHX37 overexpression was related to worsening o [file 8835393.f1.zip › Saw_Revised Supplementary Information (1).docx]

**Table S1.** DHX37 expression in cancers versus normal tissue in Oncomine database. The DHX37 expression was elevated in breast, colorectal, gastric, kidney, lung cancers as well as lymphoma, whereas DHX37 was only observed significantly reduced in sarcoma dataset.

**Table S2.** Relationship between DHX37 expression and patient prognosis of different cancer in Prognoscan database. DHX37 expression significantly impacted prognosis in 5 types of cancers, including breast, colorectal, skin, blood and lung cancers.


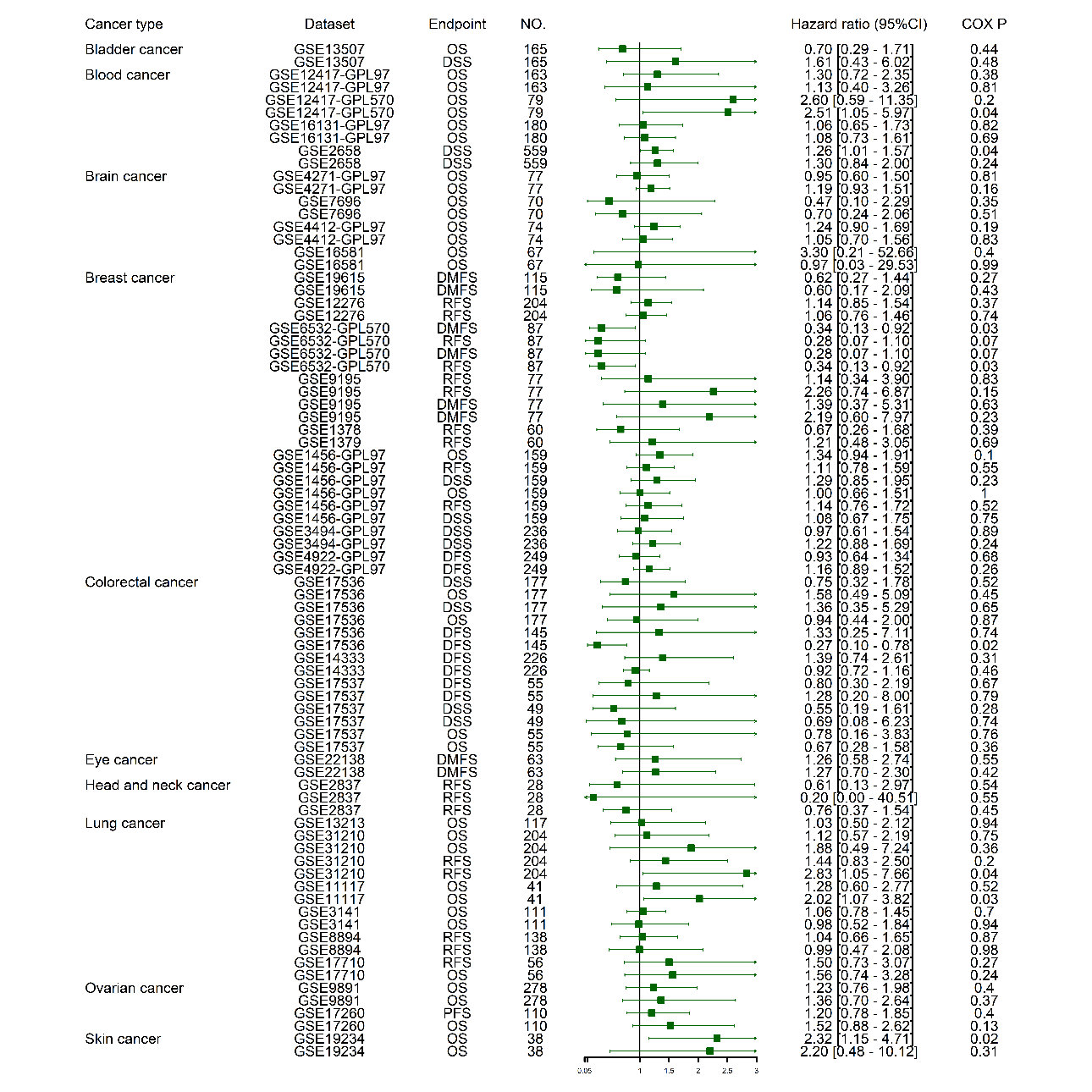


**Table S3.** DHX37 co-occurrence genes shown in PDF file (please see attachment). We found that 3682 overlap genes were positively correlated with DHX37, whereas 2002 overlap genes were negatively corelated.

**Table S4.** Summarization of correlation between DHX37 expression and immune infiltration level in diverse type cancers via TIMER database. DHX37 expression showed significant correlation with CD8 T cells, CD4 T cells, B cell, macrophages, neutrophils, and dendritic cells in 16, 19, 12, 18, 16 and 14 types of cancer, respectively.

*N, not significant; *P＜0.05；** P＜0.01；*** P＜0.001；**** P＜0.0001.*

**Figure S1.** Correlation of DHX37 expression with diverse types of cancer via Kaplan-Meier Plot.

For esophageal adenocarcinoma, DHX37 was found to have a favorable effect on relapse free survival while worsened overall survival. For head and neck squamous cell carcinoma, DHX37 expression has less influence. For thyroid carcinoma, rectum adenocarcinoma, stomach adenocarcinoma, and uterine corpus endometrial carcinoma, DHX37 plays a protective role in their OS but not RFS. DHX37 only had significant correlation with RFS for pancreatic ductal adenocarcinoma and ovarian cancer.

**Figure S2 (part 1)**

**Figure S2 (part 2)**

**Figure S2 (part 3)**

**Figure S2**. Correlation of DHX37 expression with diverse types of cancer via GEPIA. Overall survival and disease-free survival comparing the high and low expression of DHX37 in various cancers. DHX37 over-expression were related to worsen outcome of OS and DFS in ACC, LGG and LIHC; OS in LUAD, MESO and THCA; DFS in SKCM. These results validated the predictive value of DHX37 in particular types of cancer, such as LIHC and LUAD.

**
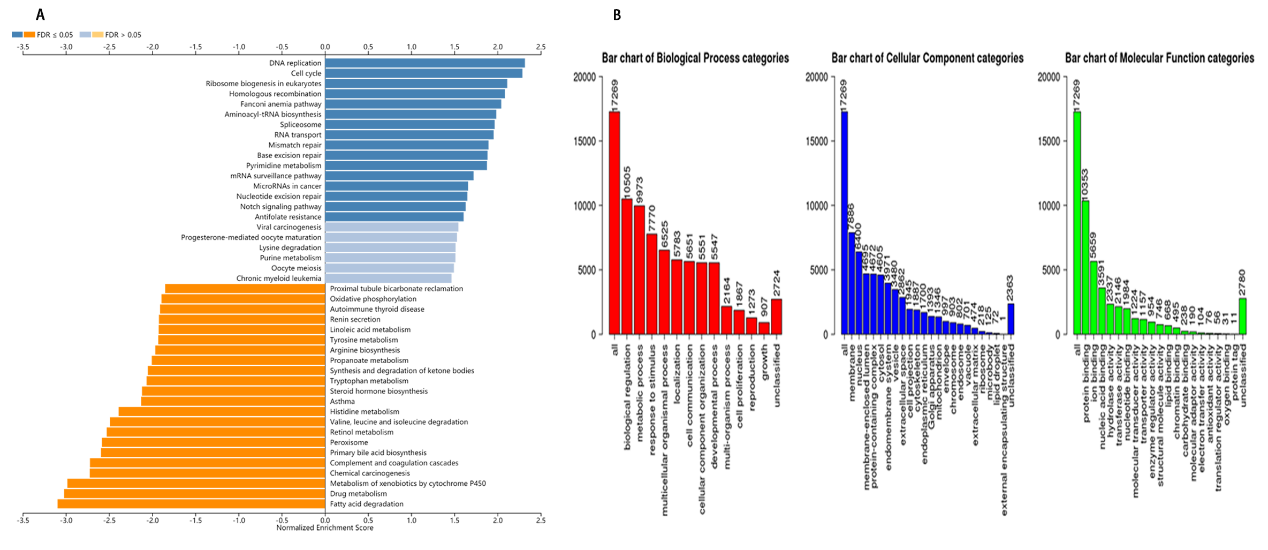
**

**Figure S3.** KEGG pathways and GO of DHX37 in LIHC and LUAD cohort. These results reveal that the functions involving cell circle modulation, amino acid metabolism, and immune activity were highly correlated with DHX37 expression.

**Figure S4 (part 1)**

**Figure S4 (part 2)**

**Figure S4 (part 3)**

**Figure S4 (part 4)**

**Figure S4.** Correlation of DHX37 expression with immune infiltration level in various cancers via TIMER database. DHX37 expression showed significantly correlated with CD8 T cells, CD4 T cells, B cell, macrophages, neutrophils, and dendritic cells in 16, 19, 12, 18, 16 and 14 types of cancer, respectively.
