# Supplementary material for: DHX37 Impacts Prognosis of Hepatocellular Carcinoma and Lung Adenocarcinoma through Immune Infiltration
Source: J Immunol Res. 2020 Dec 30;2020:8835393. doi: 10.1155/2020/8835393 (PMC7790560; doi:10.1155/2020/8835393)
Supplement: Supplementary Materials — Per the publisher's request, the details of the supplementary materials are added here. However, the editable version of each table and figure is given in a separate folder, as requested. Table S1: DHX37 expression in cancers versus normal tissue in the Oncomine database. The DHX37 expression was elevated in breast, colorectal, gastric, kidney, lung cancers as well as lymphoma, whereas DHX37 was only observed significantly reduced in the sarcoma dataset. Table S2: relationship between DHX37 expression and patient prognosis of different cancers in the PrognoScan database. Table S3: DHX37 cooccurrence genes shown in the PDF file. We found that 3682 overlap genes were positively correlated with DHX37, whereas 2002 overlap genes were negatively correlated. Table S4: summarization of correlation between DHX37 expression and immune infiltration level in diverse type cancers via the TIMER database. N: not significantly; ∗P < 0.05, ∗∗P < 0.01, ∗∗∗P < 0.001, and ∗∗∗∗P < 0.0001. DHX37 expression showed significantly correlated with CD8 T cells, CD4 T cells, B cells, macrophages, neutrophils, and dendritic cells in 16, 19, 12, 18, 16, and 14 types of cancer, respectively. Figure S1: correlation of DHX37 expression with diverse types of cancer via Kaplan-Meier Plot. For esophageal adenocarcinoma, DHX37 was found to have a favorable effect on relapse-free survival while worsening overall survival. For head and neck squamous cell carcinoma, DHX37 expression has less influence. For thyroid carcinoma, rectum adenocarcinoma, stomach adenocarcinoma, and uterine corpus endometrial carcinoma, DHX37 plays a protective role in their OS but not RFS. DHX37 only had significant correlation with RFS for pancreatic ductal adenocarcinoma and ovarian cancer. Figure S2: correlation of DHX37 expression with diverse types of cancer via GEPIA. Overall survival and disease-free survival comparing the high and low expression of DHX37 in various cancers. DHX37 overexpression was related to worsening o [file 8835393.f1.zip › Table S3_original data.docx]

Gene Sumz_

stat

Sumz_ P

Sumz_ FDR

| DHX37 | 2.59E+01 | 3.38E-148 | 6.71E-144 |
| --- | --- | --- | --- |
| DDX54 | 2.48E+01 | 8.71E-136 | 8.65E-132 |
| GCN1L1 | 2.46E+01 | 8.76E-134 | 5.80E-130 |
| PUS1 | 2.25E+01 | 9.05E-112 | 4.49E-108 |
| RBM19 | 2.14E+01 | 8.41E-102 | 3.34E-98 |
| NFKBIL2 | 1.94E+01 | 5.38E-84 | 1.78E-80 |
| RRP12 | 1.93E+01 | 8.71E-83 | 2.47E-79 |
| LMNB2 | 1.92E+01 | 4.42E-82 | 1.10E-78 |
| PGAM5 | 1.88E+01 | 9.30E-79 | 1.85E-75 |
| RAB35 | 1.88E+01 | 9.20E-79 | 1.85E-75 |
| TCOF1 | 1.86E+01 | 4.87E-77 | 8.79E-74 |
| SETD8 | 1.84E+01 | 5.95E-76 | 9.84E-73 |
| HCFC1 | 1.81E+01 | 2.05E-73 | 3.13E-70 |
| NOC4L | 1.80E+01 | 1.97E-72 | 2.80E-69 |
| CAD | 1.79E+01 | 1.71E-71 | 2.26E-68 |
| POLD1 | 1.77E+01 | 3.08E-70 | 3.82E-67 |
| RECQL4 | 1.74E+01 | 1.03E-67 | 1.20E-64 |
| SART3 | 1.74E+01 | 1.53E-67 | 1.69E-64 |
| TRIM28 | 1.72E+01 | 2.48E-66 | 2.59E-63 |
| DDX55 | 1.72E+01 | 3.60E-66 | 3.58E-63 |
| MYBBP1A | 1.71E+01 | 3.57E-65 | 3.37E-62 |
| PKMYT1 | 1.70E+01 | 6.52E-65 | 5.89E-62 |
| PTBP1 | 1.70E+01 | 9.90E-65 | 8.54E-62 |
| INCENP | 1.69E+01 | 3.45E-64 | 2.86E-61 |
| EP400 | 1.69E+01 | 5.58E-64 | 4.43E-61 |
| U2AF2 | 1.67E+01 | 8.29E-63 | 6.33E-60 |
| ANAPC7 | 1.66E+01 | 6.58E-62 | 4.84E-59 |
| PLK1 | 1.66E+01 | 7.04E-62 | 4.99E-59 |
| CDT1 | 1.65E+01 | 2.18E-61 | 1.49E-58 |
| DENR | 1.65E+01 | 3.23E-61 | 2.14E-58 |
| KIF18B | 1.65E+01 | 5.55E-61 | 3.56E-58 |
| SCRIB | 1.64E+01 | 1.96E-60 | 1.22E-57 |
| SRRT | 1.64E+01 | 2.72E-60 | 1.64E-57 |
| PDCD11 | 1.64E+01 | 2.92E-60 | 1.71E-57 |
| CCDC137 | 1.64E+01 | 3.27E-60 | 1.85E-57 |
| TMEM120B | 1.63E+01 | 1.23E-59 | 6.77E-57 |
| C15orf42 | 1.63E+01 | 1.71E-59 | 8.96E-57 |
| GOLGA3 | 1.63E+01 | 1.72E-59 | 8.96E-57 |
| DDX51 | 1.63E+01 | 2.03E-59 | 1.03E-56 |
| FOXM1 | 1.61E+01 | 2.13E-58 | 1.06E-55 |
| MYBL2 | 1.61E+01 | 3.75E-58 | 1.82E-55 |
| ANKRD52 | 1.61E+01 | 5.50E-58 | 2.60E-55 |
| DBF4B | 1.60E+01 | 7.87E-58 | 3.63E-55 |
| CCDC86 | 1.59E+01 | 3.45E-57 | 1.56E-54 |
| IPO4 | 1.59E+01 | 3.77E-57 | 1.66E-54 |
| NCAPD2 | 1.59E+01 | 7.63E-57 | 3.29E-54 |
| UTP20 | 1.58E+01 | 2.41E-56 | 1.02E-53 |
| FBXL19 | 1.58E+01 | 2.63E-56 | 1.09E-53 |
| ANKLE2 | 1.58E+01 | 4.44E-56 | 1.80E-53 |
| NAA25 | 1.58E+01 | 5.06E-56 | 2.01E-53 |
| DHX34 | 1.58E+01 | 6.73E-56 | 2.57E-53 |
| VPS33A | 1.58E+01 | 6.68E-56 | 2.57E-53 |
| HMGA1 | 1.57E+01 | 7.44E-56 | 2.79E-53 |
| TACC3 | 1.57E+01 | 1.95E-55 | 7.18E-53 |
| EFTUD2 | 1.57E+01 | 2.93E-55 | 1.06E-52 |
| SFRS8 | 1.56E+01 | 1.04E-54 | 3.68E-52 |

| TIMELESS | 1.56E+01 | 1.39E-54 | 4.83E-52 |
| --- | --- | --- | --- |
| UBE2O | 1.55E+01 | 2.67E-54 | 9.14E-52 |
| CHFR | 1.55E+01 | 3.00E-54 | 1.01E-51 |
| RBM28 | 1.55E+01 | 4.72E-54 | 1.56E-51 |
| CDCA5 | 1.55E+01 | 5.75E-54 | 1.87E-51 |
| DAZAP1 | 1.55E+01 | 5.83E-54 | 1.87E-51 |
| SMARCD1 | 1.54E+01 | 2.47E-53 | 7.77E-51 |
| POLE | 1.53E+01 | 4.94E-53 | 1.53E-50 |
| NUP62 | 1.53E+01 | 5.78E-53 | 1.76E-50 |
| SMG5 | 1.53E+01 | 1.06E-52 | 3.20E-50 |
| BRI3BP | 1.53E+01 | 1.66E-52 | 4.91E-50 |
| BOP1 | 1.52E+01 | 2.26E-52 | 6.61E-50 |
| RNF34 | 1.52E+01 | 4.21E-52 | 1.21E-49 |
| C10orf2 | 1.52E+01 | 6.43E-52 | 1.82E-49 |
| UBE2S | 1.52E+01 | 7.70E-52 | 2.15E-49 |
| RAN | 1.51E+01 | 8.18E-52 | 2.26E-49 |
| VARS | 1.51E+01 | 1.15E-51 | 3.12E-49 |
| WDR4 | 1.51E+01 | 1.53E-51 | 4.11E-49 |
| MCM7 | 1.51E+01 | 1.66E-51 | 4.38E-49 |
| C16orf59 | 1.51E+01 | 2.05E-51 | 5.34E-49 |
| EIF2B1 | 1.51E+01 | 2.12E-51 | 5.48E-49 |
| ZNF598 | 1.51E+01 | 2.84E-51 | 7.23E-49 |
| EIF3B | 1.50E+01 | 4.36E-51 | 1.10E-48 |
| GTSE1 | 1.50E+01 | 4.57E-51 | 1.14E-48 |
| SFRS9 | 1.50E+01 | 5.34E-51 | 1.31E-48 |
| POLQ | 1.50E+01 | 7.25E-51 | 1.75E-48 |
| NOP2 | 1.50E+01 | 8.19E-51 | 1.96E-48 |
| WDR62 | 1.50E+01 | 1.02E-50 | 2.40E-48 |
| TAF6 | 1.50E+01 | 1.25E-50 | 2.92E-48 |
| ATAD3A | 1.49E+01 | 1.71E-50 | 3.95E-48 |
| ESPL1 | 1.49E+01 | 3.99E-50 | 9.06E-48 |
| MKI67 | 1.49E+01 | 4.02E-50 | 9.06E-48 |
| KIF2C | 1.49E+01 | 5.68E-50 | 1.27E-47 |
| CDC25A | 1.49E+01 | 6.57E-50 | 1.45E-47 |
| PTPN23 | 1.48E+01 | 1.43E-49 | 3.12E-47 |
| TOMM40 | 1.48E+01 | 3.13E-49 | 6.76E-47 |
| AACS | 1.48E+01 | 3.21E-49 | 6.85E-47 |
| CCNF | 1.47E+01 | 3.86E-49 | 8.14E-47 |
| RBM14 | 1.47E+01 | 5.13E-49 | 1.07E-46 |
| BUB1B | 1.47E+01 | 5.48E-49 | 1.13E-46 |
| CD3EAP | 1.47E+01 | 6.40E-49 | 1.31E-46 |
| TROAP | 1.47E+01 | 7.92E-49 | 1.60E-46 |
| CDC20 | 1.47E+01 | 1.04E-48 | 2.09E-46 |
| KNTC1 | 1.47E+01 | 1.32E-48 | 2.62E-46 |
| KPNA2 | 1.46E+01 | 1.61E-48 | 3.16E-46 |
| KIF4A | 1.46E+01 | 1.86E-48 | 3.61E-46 |
| PCNXL3 | 1.46E+01 | 1.89E-48 | 3.65E-46 |
| INTS1 | 1.46E+01 | 5.24E-48 | 1.00E-45 |
| CENPO | 1.46E+01 | 5.55E-48 | 1.05E-45 |
| MCM2 | 1.45E+01 | 9.36E-48 | 1.75E-45 |
| KIFC1 | 1.45E+01 | 9.82E-48 | 1.82E-45 |
| RAD54L | 1.45E+01 | 1.32E-47 | 2.43E-45 |
| SMPD4 | 1.44E+01 | 3.54E-47 | 6.44E-45 |
| ORC1L | 1.44E+01 | 7.44E-47 | 1.34E-44 |
| DPH2 | 1.43E+01 | 1.25E-46 | 2.24E-44 |
| ATXN2L | 1.43E+01 | 2.00E-46 | 3.54E-44 |
| EXO1 | 1.43E+01 | 2.37E-46 | 4.16E-44 |
| DHX30 | 1.43E+01 | 2.87E-46 | 4.96E-44 |

| TCF3 | 1.43E+01 | 2.86E-46 | 4.96E-44 |
| --- | --- | --- | --- |
| SAPS1 | 1.43E+01 | 3.16E-46 | 5.41E-44 |
| NUP210 | 1.43E+01 | 4.37E-46 | 7.42E-44 |
| GATC | 1.43E+01 | 4.46E-46 | 7.50E-44 |
| CHAF1A | 1.42E+01 | 5.00E-46 | 8.35E-44 |
| XPO5 | 1.42E+01 | 5.12E-46 | 8.47E-44 |
| TPX2 | 1.42E+01 | 5.23E-46 | 8.58E-44 |
| HGS | 1.42E+01 | 7.18E-46 | 1.17E-43 |
| TRIP13 | 1.42E+01 | 7.85E-46 | 1.27E-43 |
| MARS | 1.42E+01 | 1.38E-45 | 2.20E-43 |
| NCOR2 | 1.42E+01 | 1.46E-45 | 2.32E-43 |
| ARHGAP39 | 1.42E+01 | 1.65E-45 | 2.61E-43 |
| CDCA3 | 1.42E+01 | 1.82E-45 | 2.82E-43 |
| POP1 | 1.42E+01 | 1.81E-45 | 2.82E-43 |
| FAM72D | 1.41E+01 | 2.07E-45 | 3.18E-43 |
| MCM10 | 1.41E+01 | 2.87E-45 | 4.38E-43 |
| DKC1 | 1.41E+01 | 3.59E-45 | 5.44E-43 |
| NCAPH | 1.41E+01 | 5.83E-45 | 8.77E-43 |
| UHRF1 | 1.40E+01 | 9.02E-45 | 1.35E-42 |
| POLR1A | 1.40E+01 | 1.20E-44 | 1.78E-42 |
| ANAPC5 | 1.40E+01 | 1.28E-44 | 1.88E-42 |
| AURKB | 1.40E+01 | 1.72E-44 | 2.51E-42 |
| MCM4 | 1.40E+01 | 2.04E-44 | 2.96E-42 |
| ILF3 | 1.40E+01 | 2.30E-44 | 3.31E-42 |
| ZC3H3 | 1.40E+01 | 2.97E-44 | 4.25E-42 |
| FOXK2 | 1.39E+01 | 3.67E-44 | 5.21E-42 |
| UBAP2 | 1.39E+01 | 4.01E-44 | 5.64E-42 |
| GSG2 | 1.39E+01 | 4.06E-44 | 5.68E-42 |
| FBRSL1 | 1.39E+01 | 4.57E-44 | 6.35E-42 |
| EHMT2 | 1.39E+01 | 7.32E-44 | 1.01E-41 |
| STIP1 | 1.39E+01 | 8.65E-44 | 1.18E-41 |
| CNOT3 | 1.38E+01 | 1.48E-43 | 2.01E-41 |
| KIAA1524 | 1.38E+01 | 1.62E-43 | 2.18E-41 |
| TMEM201 | 1.38E+01 | 2.11E-43 | 2.83E-41 |
| BAT2 | 1.38E+01 | 2.39E-43 | 3.18E-41 |
| MCRS1 | 1.38E+01 | 2.56E-43 | 3.39E-41 |
| PRKDC | 1.38E+01 | 2.72E-43 | 3.58E-41 |
| CENPE | 1.38E+01 | 4.01E-43 | 5.23E-41 |
| PRDM4 | 1.38E+01 | 4.17E-43 | 5.41E-41 |
| CKAP4 | 1.37E+01 | 7.50E-43 | 9.67E-41 |
| NAT10 | 1.37E+01 | 8.00E-43 | 1.03E-40 |
| REXO4 | 1.37E+01 | 9.26E-43 | 1.18E-40 |
| SKA1 | 1.37E+01 | 9.79E-43 | 1.24E-40 |
| PTCD1 | 1.37E+01 | 9.90E-43 | 1.24E-40 |
| KIAA0415 | 1.37E+01 | 1.01E-42 | 1.26E-40 |
| XRCC2 | 1.37E+01 | 1.06E-42 | 1.31E-40 |
| SPAG5 | 1.37E+01 | 1.19E-42 | 1.47E-40 |
| ZNF335 | 1.37E+01 | 1.77E-42 | 2.16E-40 |
| AZI1 | 1.37E+01 | 1.81E-42 | 2.20E-40 |
| DNMT1 | 1.36E+01 | 2.50E-42 | 3.03E-40 |
| FAM72B | 1.36E+01 | 2.61E-42 | 3.14E-40 |
| NLE1 | 1.36E+01 | 2.87E-42 | 3.43E-40 |
| ACLY | 1.36E+01 | 3.28E-42 | 3.90E-40 |
| E2F2 | 1.36E+01 | 3.40E-42 | 4.02E-40 |
| HNRNPM | 1.36E+01 | 3.54E-42 | 4.16E-40 |
| LIG1 | 1.36E+01 | 4.37E-42 | 5.11E-40 |
| CLSPN | 1.36E+01 | 4.82E-42 | 5.59E-40 |
| HJURP | 1.36E+01 | 5.65E-42 | 6.52E-40 |

| KLC2 | 1.36E+01 | 5.86E-42 | 6.72E-40 |
| --- | --- | --- | --- |
| CENPF | 1.36E+01 | 6.38E-42 | 7.28E-40 |
| H2AFX | 1.36E+01 | 8.33E-42 | 9.46E-40 |
| EME1 | 1.35E+01 | 9.04E-42 | 1.02E-39 |
| NOC2L | 1.35E+01 | 9.37E-42 | 1.05E-39 |
| GPR172A | 1.35E+01 | 1.75E-41 | 1.95E-39 |
| SYMPK | 1.35E+01 | 1.85E-41 | 2.05E-39 |
| XPO6 | 1.35E+01 | 1.97E-41 | 2.17E-39 |
| ARHGAP11A | 1.35E+01 | 2.01E-41 | 2.20E-39 |
| KIF23 | 1.35E+01 | 2.36E-41 | 2.57E-39 |
| STRN4 | 1.35E+01 | 2.61E-41 | 2.83E-39 |
| SHKBP1 | 1.35E+01 | 3.08E-41 | 3.33E-39 |
| C11orf84 | 1.35E+01 | 3.23E-41 | 3.46E-39 |
| RRP9 | 1.34E+01 | 7.32E-41 | 7.82E-39 |
| CDC45 | 1.34E+01 | 7.63E-41 | 8.10E-39 |
| PPRC1 | 1.34E+01 | 7.89E-41 | 8.33E-39 |
| CDCA8 | 1.34E+01 | 9.42E-41 | 9.89E-39 |
| ZNF282 | 1.34E+01 | 1.08E-40 | 1.13E-38 |
| DDX11 | 1.34E+01 | 1.12E-40 | 1.17E-38 |
| FANCD2 | 1.34E+01 | 1.24E-40 | 1.28E-38 |
| WHSC1 | 1.33E+01 | 1.45E-40 | 1.49E-38 |
| TRAIP | 1.33E+01 | 1.61E-40 | 1.65E-38 |
| CDC6 | 1.33E+01 | 1.68E-40 | 1.71E-38 |
| BUB1 | 1.33E+01 | 1.75E-40 | 1.77E-38 |
| HSF1 | 1.33E+01 | 2.07E-40 | 2.09E-38 |
| NOL6 | 1.33E+01 | 2.23E-40 | 2.23E-38 |
| C12orf43 | 1.33E+01 | 3.58E-40 | 3.57E-38 |
| RACGAP1 | 1.33E+01 | 3.69E-40 | 3.66E-38 |
| DSCC1 | 1.33E+01 | 3.90E-40 | 3.85E-38 |
| C12orf52 | 1.33E+01 | 4.16E-40 | 4.09E-38 |
| DDN | 1.33E+01 | 4.27E-40 | 4.15E-38 |
| NCL | 1.33E+01 | 4.26E-40 | 4.15E-38 |
| RPTOR | 1.33E+01 | 4.55E-40 | 4.41E-38 |
| NCAPG | 1.32E+01 | 4.91E-40 | 4.74E-38 |
| GMEB2 | 1.32E+01 | 5.31E-40 | 5.09E-38 |
| RRS1 | 1.32E+01 | 6.00E-40 | 5.73E-38 |
| SETD1A | 1.32E+01 | 7.43E-40 | 7.06E-38 |
| UCK2 | 1.32E+01 | 7.67E-40 | 7.25E-38 |
| KIF15 | 1.32E+01 | 7.83E-40 | 7.37E-38 |
| C17orf53 | 1.32E+01 | 7.93E-40 | 7.43E-38 |
| CENPA | 1.32E+01 | 1.03E-39 | 9.57E-38 |
| ULK1 | 1.32E+01 | 1.18E-39 | 1.10E-37 |
| PRC1 | 1.32E+01 | 1.46E-39 | 1.35E-37 |
| KIF11 | 1.32E+01 | 1.50E-39 | 1.38E-37 |
| CEP55 | 1.32E+01 | 1.71E-39 | 1.57E-37 |
| CCNB1 | 1.31E+01 | 1.88E-39 | 1.71E-37 |
| DVL2 | 1.31E+01 | 2.08E-39 | 1.89E-37 |
| KIF14 | 1.31E+01 | 2.22E-39 | 2.00E-37 |
| DGKZ | 1.31E+01 | 2.26E-39 | 2.03E-37 |
| LMNB1 | 1.31E+01 | 2.57E-39 | 2.30E-37 |
| LYAR | 1.31E+01 | 3.28E-39 | 2.92E-37 |
| YEATS2 | 1.31E+01 | 4.58E-39 | 4.06E-37 |
| PES1 | 1.31E+01 | 4.99E-39 | 4.40E-37 |
| SGOL1 | 1.31E+01 | 6.78E-39 | 5.96E-37 |
| CDCA2 | 1.30E+01 | 7.36E-39 | 6.44E-37 |
| SCAF1 | 1.30E+01 | 7.57E-39 | 6.59E-37 |
| ATAD3B | 1.30E+01 | 8.97E-39 | 7.78E-37 |
| DOT1L | 1.30E+01 | 9.05E-39 | 7.82E-37 |

| EZH2 | 1.30E+01 | 9.92E-39 | 8.52E-37 |
| --- | --- | --- | --- |
| CAMKK2 | 1.30E+01 | 1.01E-38 | 8.62E-37 |
| ARFGAP1 | 1.30E+01 | 1.12E-38 | 9.50E-37 |
| C9orf100 | 1.30E+01 | 1.20E-38 | 1.02E-36 |
| CPSF1 | 1.30E+01 | 1.35E-38 | 1.14E-36 |
| XRCC3 | 1.30E+01 | 1.43E-38 | 1.20E-36 |
| DNMT3A | 1.30E+01 | 1.55E-38 | 1.30E-36 |
| BIRC5 | 1.30E+01 | 1.56E-38 | 1.30E-36 |
| SSRP1 | 1.30E+01 | 1.62E-38 | 1.35E-36 |
| ORC6L | 1.30E+01 | 2.04E-38 | 1.69E-36 |
| GIT1 | 1.30E+01 | 2.21E-38 | 1.82E-36 |
| FANCA | 1.29E+01 | 2.87E-38 | 2.36E-36 |
| ABCF2 | 1.29E+01 | 3.74E-38 | 3.05E-36 |
| MAPKAPK5 | 1.29E+01 | 5.18E-38 | 4.22E-36 |
| KHSRP | 1.29E+01 | 6.73E-38 | 5.46E-36 |
| ATXN7L3 | 1.29E+01 | 8.02E-38 | 6.47E-36 |
| MTHFD1L | 1.28E+01 | 1.26E-37 | 1.01E-35 |
| UBTF | 1.28E+01 | 1.42E-37 | 1.14E-35 |
| RCC2 | 1.28E+01 | 1.60E-37 | 1.27E-35 |
| PPM1G | 1.28E+01 | 1.83E-37 | 1.45E-35 |
| BRCA1 | 1.28E+01 | 1.87E-37 | 1.48E-35 |
| CENPM | 1.28E+01 | 2.09E-37 | 1.65E-35 |
| MLL4 | 1.28E+01 | 2.21E-37 | 1.73E-35 |
| KPNB1 | 1.28E+01 | 2.61E-37 | 2.04E-35 |
| G6PD | 1.28E+01 | 2.73E-37 | 2.12E-35 |
| TTK | 1.28E+01 | 2.74E-37 | 2.12E-35 |
| FAM54A | 1.28E+01 | 2.76E-37 | 2.13E-35 |
| SUDS3 | 1.28E+01 | 2.80E-37 | 2.16E-35 |
| NOP56 | 1.27E+01 | 3.51E-37 | 2.69E-35 |
| IQGAP3 | 1.27E+01 | 5.29E-37 | 4.04E-35 |
| LARP1 | 1.27E+01 | 6.01E-37 | 4.57E-35 |
| CEP250 | 1.27E+01 | 6.81E-37 | 5.16E-35 |
| PFAS | 1.27E+01 | 8.46E-37 | 6.39E-35 |
| TIGD5 | 1.27E+01 | 1.03E-36 | 7.77E-35 |
| SKA3 | 1.27E+01 | 1.06E-36 | 7.97E-35 |
| DDX23 | 1.27E+01 | 1.13E-36 | 8.45E-35 |
| ANLN | 1.27E+01 | 1.17E-36 | 8.72E-35 |
| RAVER1 | 1.26E+01 | 1.25E-36 | 9.28E-35 |
| TTF2 | 1.26E+01 | 1.30E-36 | 9.58E-35 |
| FEN1 | 1.26E+01 | 1.37E-36 | 1.00E-34 |
| CEP164 | 1.26E+01 | 1.45E-36 | 1.07E-34 |
| ZNF668 | 1.26E+01 | 1.46E-36 | 1.07E-34 |
| ARHGAP11B | 1.26E+01 | 1.59E-36 | 1.15E-34 |
| BRAP | 1.26E+01 | 1.59E-36 | 1.15E-34 |
| ERCC6L | 1.26E+01 | 1.75E-36 | 1.26E-34 |
| EIF2C2 | 1.26E+01 | 2.34E-36 | 1.68E-34 |
| SF1 | 1.26E+01 | 2.82E-36 | 2.02E-34 |
| PAK4 | 1.26E+01 | 4.16E-36 | 2.97E-34 |
| C19orf48 | 1.25E+01 | 4.39E-36 | 3.12E-34 |
| DDX12 | 1.25E+01 | 6.64E-36 | 4.71E-34 |
| MELK | 1.25E+01 | 6.86E-36 | 4.85E-34 |
| PPAN | 1.25E+01 | 7.15E-36 | 5.03E-34 |
| RANGAP1 | 1.25E+01 | 7.50E-36 | 5.26E-34 |
| FAM72A | 1.25E+01 | 8.17E-36 | 5.71E-34 |
| URB2 | 1.25E+01 | 8.36E-36 | 5.82E-34 |
| RAD51 | 1.25E+01 | 8.51E-36 | 5.91E-34 |
| NCAPG2 | 1.25E+01 | 9.78E-36 | 6.77E-34 |
| KIF20A | 1.25E+01 | 1.01E-35 | 6.96E-34 |

| SMYD5 | 1.25E+01 | 1.06E-35 | 7.26E-34 |
| --- | --- | --- | --- |
| CHEK1 | 1.25E+01 | 1.30E-35 | 8.90E-34 |
| EWSR1 | 1.24E+01 | 1.49E-35 | 1.02E-33 |
| TUBA1B | 1.24E+01 | 1.68E-35 | 1.14E-33 |
| MTA2 | 1.24E+01 | 1.85E-35 | 1.25E-33 |
| C1orf135 | 1.24E+01 | 1.97E-35 | 1.33E-33 |
| RUVBL1 | 1.24E+01 | 1.99E-35 | 1.34E-33 |
| PA2G4 | 1.24E+01 | 2.57E-35 | 1.72E-33 |
| ARID3A | 1.24E+01 | 3.00E-35 | 2.00E-33 |
| UNG | 1.24E+01 | 3.13E-35 | 2.09E-33 |
| BLM | 1.24E+01 | 3.87E-35 | 2.57E-33 |
| ZNF142 | 1.24E+01 | 4.16E-35 | 2.75E-33 |
| CENPI | 1.24E+01 | 4.34E-35 | 2.87E-33 |
| KLF16 | 1.23E+01 | 6.05E-35 | 3.98E-33 |
| C11orf82 | 1.23E+01 | 6.14E-35 | 4.02E-33 |
| SART1 | 1.23E+01 | 6.16E-35 | 4.02E-33 |
| TUBA1C | 1.23E+01 | 6.67E-35 | 4.34E-33 |
| TUBG1 | 1.23E+01 | 6.80E-35 | 4.41E-33 |
| PSRC1 | 1.23E+01 | 8.13E-35 | 5.26E-33 |
| E2F4 | 1.23E+01 | 8.99E-35 | 5.80E-33 |
| BEND3 | 1.23E+01 | 1.07E-34 | 6.86E-33 |
| SRM | 1.23E+01 | 1.28E-34 | 8.19E-33 |
| ASPM | 1.23E+01 | 1.35E-34 | 8.60E-33 |
| NUP37 | 1.23E+01 | 1.62E-34 | 1.03E-32 |
| RCOR2 | 1.23E+01 | 1.66E-34 | 1.06E-32 |
| NACC1 | 1.22E+01 | 1.80E-34 | 1.14E-32 |
| CHTF18 | 1.22E+01 | 1.84E-34 | 1.16E-32 |
| ZBTB17 | 1.22E+01 | 1.90E-34 | 1.19E-32 |
| AATF | 1.22E+01 | 2.43E-34 | 1.53E-32 |
| TDG | 1.22E+01 | 4.07E-34 | 2.54E-32 |
| NCLN | 1.22E+01 | 4.15E-34 | 2.58E-32 |
| E2F1 | 1.22E+01 | 4.17E-34 | 2.58E-32 |
| MFSD2B | 1.22E+01 | 4.50E-34 | 2.78E-32 |
| SHCBP1 | 1.22E+01 | 4.72E-34 | 2.91E-32 |
| EDC4 | 1.22E+01 | 4.97E-34 | 3.05E-32 |
| TCHP | 1.22E+01 | 5.11E-34 | 3.13E-32 |
| GTF3C2 | 1.21E+01 | 6.40E-34 | 3.91E-32 |
| RRM2 | 1.21E+01 | 7.36E-34 | 4.48E-32 |
| WHSC2 | 1.21E+01 | 7.49E-34 | 4.55E-32 |
| NUP205 | 1.21E+01 | 7.96E-34 | 4.82E-32 |
| MCM5 | 1.21E+01 | 8.55E-34 | 5.16E-32 |
| C9orf140 | 1.21E+01 | 9.06E-34 | 5.45E-32 |
| YKT6 | 1.21E+01 | 9.32E-34 | 5.59E-32 |
| DLGAP5 | 1.21E+01 | 9.68E-34 | 5.77E-32 |
| PIF1 | 1.21E+01 | 9.68E-34 | 5.77E-32 |
| MYO19 | 1.21E+01 | 1.17E-33 | 6.92E-32 |
| THOC4 | 1.21E+01 | 1.17E-33 | 6.92E-32 |
| CSNK1G2 | 1.21E+01 | 1.51E-33 | 8.89E-32 |
| PLXNA1 | 1.21E+01 | 1.99E-33 | 1.17E-31 |
| TRRAP | 1.20E+01 | 2.27E-33 | 1.34E-31 |
| GTPBP4 | 1.20E+01 | 2.30E-33 | 1.34E-31 |
| PRR11 | 1.20E+01 | 2.44E-33 | 1.42E-31 |
| ZNF605 | 1.20E+01 | 2.65E-33 | 1.54E-31 |
| EPR1 | 1.20E+01 | 2.70E-33 | 1.57E-31 |
| AP2A1 | 1.20E+01 | 2.79E-33 | 1.62E-31 |
| TOP2A | 1.20E+01 | 2.94E-33 | 1.70E-31 |
| PRPF19 | 1.20E+01 | 3.28E-33 | 1.89E-31 |
| MARK2 | 1.20E+01 | 3.95E-33 | 2.27E-31 |

| MTBP | 1.20E+01 | 5.12E-33 | 2.93E-31 |
| --- | --- | --- | --- |
| GINS4 | 1.20E+01 | 5.13E-33 | 2.93E-31 |
| SLC4A2 | 1.20E+01 | 5.30E-33 | 3.01E-31 |
| NCAPD3 | 1.19E+01 | 7.68E-33 | 4.35E-31 |
| ZWINT | 1.19E+01 | 8.59E-33 | 4.86E-31 |
| FANCI | 1.19E+01 | 9.11E-33 | 5.14E-31 |
| PWP1 | 1.19E+01 | 9.18E-33 | 5.16E-31 |
| WDR67 | 1.19E+01 | 1.08E-32 | 6.03E-31 |
| RNFT2 | 1.19E+01 | 1.14E-32 | 6.35E-31 |
| RPAP1 | 1.19E+01 | 1.23E-32 | 6.86E-31 |
| HIP1R | 1.19E+01 | 1.23E-32 | 6.86E-31 |
| FBXO46 | 1.19E+01 | 1.31E-32 | 7.24E-31 |
| TBRG4 | 1.19E+01 | 1.36E-32 | 7.52E-31 |
| UBE2C | 1.19E+01 | 1.48E-32 | 8.16E-31 |
| DDX27 | 1.19E+01 | 1.52E-32 | 8.36E-31 |
| SPC25 | 1.19E+01 | 1.63E-32 | 8.95E-31 |
| MAFG | 1.19E+01 | 1.65E-32 | 9.05E-31 |
| STK11IP | 1.19E+01 | 1.74E-32 | 9.48E-31 |
| MED22 | 1.19E+01 | 1.76E-32 | 9.55E-31 |
| CKAP2L | 1.19E+01 | 1.86E-32 | 1.01E-30 |
| SAMD1 | 1.18E+01 | 3.08E-32 | 1.67E-30 |
| NUF2 | 1.18E+01 | 3.71E-32 | 2.00E-30 |
| FANCG | 1.18E+01 | 3.97E-32 | 2.14E-30 |
| ATXN2 | 1.18E+01 | 4.39E-32 | 2.36E-30 |
| ZNF628 | 1.18E+01 | 4.51E-32 | 2.41E-30 |
| QSOX2 | 1.18E+01 | 5.31E-32 | 2.84E-30 |
| SPATS2 | 1.18E+01 | 5.62E-32 | 2.99E-30 |
| FOXK1 | 1.18E+01 | 5.66E-32 | 3.00E-30 |
| MCM8 | 1.18E+01 | 5.79E-32 | 3.07E-30 |
| SGOL2 | 1.18E+01 | 6.35E-32 | 3.35E-30 |
| SFPQ | 1.18E+01 | 6.50E-32 | 3.42E-30 |
| ATAD5 | 1.17E+01 | 8.16E-32 | 4.29E-30 |
| CS | 1.17E+01 | 8.63E-32 | 4.52E-30 |
| DNAJC2 | 1.17E+01 | 9.76E-32 | 5.10E-30 |
| BMS1 | 1.17E+01 | 1.01E-31 | 5.25E-30 |
| BAZ2A | 1.17E+01 | 1.06E-31 | 5.50E-30 |
| NEK2 | 1.17E+01 | 1.06E-31 | 5.51E-30 |
| TK1 | 1.17E+01 | 1.12E-31 | 5.78E-30 |
| SETD1B | 1.17E+01 | 1.20E-31 | 6.19E-30 |
| NEIL3 | 1.17E+01 | 1.29E-31 | 6.62E-30 |
| DDX39 | 1.17E+01 | 1.32E-31 | 6.78E-30 |
| C12orf48 | 1.17E+01 | 1.33E-31 | 6.82E-30 |
| NDC80 | 1.17E+01 | 1.39E-31 | 7.10E-30 |
| EIF4G1 | 1.17E+01 | 1.43E-31 | 7.26E-30 |
| POLR3A | 1.17E+01 | 1.54E-31 | 7.78E-30 |
| SLC7A5 | 1.17E+01 | 1.54E-31 | 7.78E-30 |
| ZC3H18 | 1.17E+01 | 1.56E-31 | 7.89E-30 |
| ADRM1 | 1.17E+01 | 1.59E-31 | 8.01E-30 |
| ZNF775 | 1.17E+01 | 1.69E-31 | 8.48E-30 |
| DVL3 | 1.17E+01 | 1.70E-31 | 8.48E-30 |
| PLK4 | 1.17E+01 | 1.70E-31 | 8.48E-30 |
| NOLC1 | 1.17E+01 | 1.86E-31 | 9.30E-30 |
| SOLH | 1.17E+01 | 1.93E-31 | 9.58E-30 |
| TTLL4 | 1.17E+01 | 1.93E-31 | 9.58E-30 |
| CCNB2 | 1.17E+01 | 1.97E-31 | 9.75E-30 |
| CCT5 | 1.17E+01 | 1.97E-31 | 9.75E-30 |
| BRD4 | 1.16E+01 | 2.68E-31 | 1.32E-29 |
| ZBTB12 | 1.16E+01 | 2.73E-31 | 1.34E-29 |

| PWP2 | 1.16E+01 | 2.81E-31 | 1.38E-29 |
| --- | --- | --- | --- |
| PRMT1 | 1.16E+01 | 2.99E-31 | 1.46E-29 |
| PRR7 | 1.16E+01 | 4.10E-31 | 2.00E-29 |
| NSUN5 | 1.16E+01 | 4.58E-31 | 2.23E-29 |
| HELLS | 1.16E+01 | 4.87E-31 | 2.37E-29 |
| HEATR1 | 1.16E+01 | 5.61E-31 | 2.72E-29 |
| SLC7A1 | 1.16E+01 | 7.14E-31 | 3.45E-29 |
| C14orf80 | 1.15E+01 | 9.46E-31 | 4.56E-29 |
| IGF2BP3 | 1.15E+01 | 1.07E-30 | 5.16E-29 |
| TFAP4 | 1.15E+01 | 1.32E-30 | 6.31E-29 |
| AURKA | 1.15E+01 | 1.37E-30 | 6.51E-29 |
| MED24 | 1.15E+01 | 1.36E-30 | 6.51E-29 |
| DNMT3B | 1.15E+01 | 1.41E-30 | 6.72E-29 |
| CHD7 | 1.15E+01 | 1.42E-30 | 6.75E-29 |
| OGFOD2 | 1.15E+01 | 2.24E-30 | 1.06E-28 |
| LRWD1 | 1.14E+01 | 2.68E-30 | 1.27E-28 |
| DOHH | 1.14E+01 | 2.85E-30 | 1.34E-28 |
| OIP5 | 1.14E+01 | 2.89E-30 | 1.36E-28 |
| ASAP1 | 1.14E+01 | 3.79E-30 | 1.78E-28 |
| CCNE1 | 1.14E+01 | 3.90E-30 | 1.82E-28 |
| RNF216 | 1.14E+01 | 5.51E-30 | 2.58E-28 |
| PFKFB4 | 1.14E+01 | 6.27E-30 | 2.92E-28 |
| CBX2 | 1.14E+01 | 6.51E-30 | 3.03E-28 |
| STIL | 1.14E+01 | 6.74E-30 | 3.13E-28 |
| CCT6A | 1.14E+01 | 7.55E-30 | 3.49E-28 |
| CEP72 | 1.13E+01 | 8.26E-30 | 3.81E-28 |
| CHORDC1 | 1.13E+01 | 9.59E-30 | 4.42E-28 |
| SF3B4 | 1.13E+01 | 9.82E-30 | 4.51E-28 |
| BYSL | 1.13E+01 | 1.07E-29 | 4.91E-28 |
| GPATCH4 | 1.13E+01 | 1.10E-29 | 5.01E-28 |
| RFC5 | 1.13E+01 | 1.10E-29 | 5.03E-28 |
| FAM64A | 1.13E+01 | 1.13E-29 | 5.14E-28 |
| GPC2 | 1.13E+01 | 1.16E-29 | 5.28E-28 |
| KDM1A | 1.13E+01 | 1.19E-29 | 5.38E-28 |
| SLC25A22 | 1.13E+01 | 1.26E-29 | 5.71E-28 |
| SPNS1 | 1.13E+01 | 1.29E-29 | 5.81E-28 |
| TRMT61A | 1.13E+01 | 1.47E-29 | 6.62E-28 |
| MLXIP | 1.13E+01 | 1.90E-29 | 8.53E-28 |
| PUS7 | 1.13E+01 | 2.04E-29 | 9.14E-28 |
| PLBD2 | 1.13E+01 | 2.18E-29 | 9.75E-28 |
| C19orf40 | 1.12E+01 | 2.78E-29 | 1.24E-27 |
| DMWD | 1.12E+01 | 3.02E-29 | 1.34E-27 |
| FARSA | 1.12E+01 | 3.06E-29 | 1.36E-27 |
| CCDC99 | 1.12E+01 | 3.80E-29 | 1.68E-27 |
| PSMC3IP | 1.12E+01 | 3.93E-29 | 1.74E-27 |
| HEATR2 | 1.12E+01 | 4.08E-29 | 1.80E-27 |
| DEPDC1 | 1.12E+01 | 4.17E-29 | 1.84E-27 |
| FAM136A | 1.12E+01 | 5.23E-29 | 2.30E-27 |
| DBN1 | 1.12E+01 | 6.02E-29 | 2.64E-27 |
| E2F8 | 1.12E+01 | 6.52E-29 | 2.85E-27 |
| GINS1 | 1.12E+01 | 6.57E-29 | 2.87E-27 |
| VPS37C | 1.12E+01 | 7.11E-29 | 3.10E-27 |
| ASF1B | 1.11E+01 | 7.85E-29 | 3.41E-27 |
| DBF4 | 1.11E+01 | 9.01E-29 | 3.91E-27 |
| DEPDC1B | 1.11E+01 | 9.24E-29 | 4.00E-27 |
| ZNF707 | 1.11E+01 | 1.06E-28 | 4.56E-27 |
| PTGES2 | 1.11E+01 | 1.08E-28 | 4.67E-27 |
| SF3A2 | 1.11E+01 | 1.10E-28 | 4.71E-27 |

| NUP188 | 1.11E+01 | 1.52E-28 | 6.53E-27 |
| --- | --- | --- | --- |
| LONP1 | 1.11E+01 | 1.60E-28 | 6.85E-27 |
| RNF10 | 1.11E+01 | 1.73E-28 | 7.41E-27 |
| DTL | 1.11E+01 | 1.88E-28 | 7.99E-27 |
| CDC25C | 1.11E+01 | 1.98E-28 | 8.40E-27 |
| ZNF48 | 1.11E+01 | 2.07E-28 | 8.77E-27 |
| GRWD1 | 1.11E+01 | 2.08E-28 | 8.81E-27 |
| RPUSD1 | 1.11E+01 | 2.21E-28 | 9.32E-27 |
| RRP1 | 1.10E+01 | 2.58E-28 | 1.09E-26 |
| CPSF4 | 1.10E+01 | 2.79E-28 | 1.17E-26 |
| KIF20B | 1.10E+01 | 2.92E-28 | 1.23E-26 |
| LIMK1 | 1.10E+01 | 3.61E-28 | 1.51E-26 |
| FAM83D | 1.10E+01 | 3.62E-28 | 1.51E-26 |
| BRD9 | 1.10E+01 | 3.79E-28 | 1.58E-26 |
| STK25 | 1.10E+01 | 3.82E-28 | 1.59E-26 |
| CENPH | 1.10E+01 | 4.05E-28 | 1.68E-26 |
| THOP1 | 1.10E+01 | 4.61E-28 | 1.91E-26 |
| SOX12 | 1.10E+01 | 4.82E-28 | 1.99E-26 |
| JRK | 1.10E+01 | 4.92E-28 | 2.03E-26 |
| NUP93 | 1.10E+01 | 5.70E-28 | 2.35E-26 |
| PELP1 | 1.10E+01 | 5.74E-28 | 2.36E-26 |
| HPDL | 1.10E+01 | 6.06E-28 | 2.49E-26 |
| POLR3E | 1.10E+01 | 6.35E-28 | 2.60E-26 |
| CKAP5 | 1.10E+01 | 6.41E-28 | 2.62E-26 |
| IRAK1 | 1.10E+01 | 6.86E-28 | 2.79E-26 |
| PARP1 | 1.10E+01 | 6.85E-28 | 2.79E-26 |
| POLA2 | 1.10E+01 | 6.94E-28 | 2.82E-26 |
| CCDC9 | 1.09E+01 | 7.81E-28 | 3.17E-26 |
| PPP1CC | 1.09E+01 | 8.40E-28 | 3.40E-26 |
| C20orf27 | 1.09E+01 | 9.69E-28 | 3.91E-26 |
| CSTF2 | 1.09E+01 | 1.08E-27 | 4.36E-26 |
| NAA40 | 1.09E+01 | 1.11E-27 | 4.47E-26 |
| C1orf159 | 1.09E+01 | 1.12E-27 | 4.47E-26 |
| FTSJ3 | 1.09E+01 | 1.46E-27 | 5.82E-26 |
| SH2B2 | 1.09E+01 | 1.65E-27 | 6.60E-26 |
| PLOD3 | 1.09E+01 | 1.66E-27 | 6.62E-26 |
| WIZ | 1.09E+01 | 1.72E-27 | 6.84E-26 |
| GTF3C1 | 1.09E+01 | 1.80E-27 | 7.13E-26 |
| RFWD3 | 1.09E+01 | 1.88E-27 | 7.45E-26 |
| SNRPA | 1.09E+01 | 2.05E-27 | 8.13E-26 |
| RHBDF2 | 1.08E+01 | 2.14E-27 | 8.43E-26 |
| ADRBK1 | 1.08E+01 | 2.15E-27 | 8.48E-26 |
| LETM1 | 1.08E+01 | 2.21E-27 | 8.67E-26 |
| RFC4 | 1.08E+01 | 2.23E-27 | 8.73E-26 |
| KRI1 | 1.08E+01 | 2.27E-27 | 8.88E-26 |
| GLT25D1 | 1.08E+01 | 2.45E-27 | 9.56E-26 |
| FAM189B | 1.08E+01 | 2.61E-27 | 1.02E-25 |
| USP36 | 1.08E+01 | 2.97E-27 | 1.16E-25 |
| ATP13A1 | 1.08E+01 | 3.38E-27 | 1.32E-25 |
| LOC100128191 | 1.08E+01 | 3.58E-27 | 1.39E-25 |
| ABCD1 | 1.08E+01 | 4.12E-27 | 1.59E-25 |
| R3HDM1 | 1.08E+01 | 4.20E-27 | 1.62E-25 |
| XPOT | 1.08E+01 | 4.40E-27 | 1.70E-25 |
| CHERP | 1.08E+01 | 4.47E-27 | 1.72E-25 |
| CDC7 | 1.08E+01 | 4.66E-27 | 1.79E-25 |
| BCL2L12 | 1.08E+01 | 4.90E-27 | 1.88E-25 |
| MPHOSPH9 | 1.08E+01 | 5.09E-27 | 1.95E-25 |
| RAD51AP1 | 1.08E+01 | 5.34E-27 | 2.04E-25 |

| BRPF1 | 1.08E+01 | 6.02E-27 | 2.29E-25 |
| --- | --- | --- | --- |
| C7orf27 | 1.08E+01 | 6.02E-27 | 2.29E-25 |
| AAAS | 1.07E+01 | 6.71E-27 | 2.55E-25 |
| PSME3 | 1.07E+01 | 7.48E-27 | 2.84E-25 |
| CAPN10 | 1.07E+01 | 9.17E-27 | 3.47E-25 |
| PPAT | 1.07E+01 | 1.05E-26 | 3.95E-25 |
| HMGXB3 | 1.07E+01 | 1.11E-26 | 4.20E-25 |
| FBXW8 | 1.07E+01 | 1.20E-26 | 4.52E-25 |
| KIF18A | 1.07E+01 | 1.22E-26 | 4.59E-25 |
| SPC24 | 1.07E+01 | 1.23E-26 | 4.59E-25 |
| CSNK1D | 1.07E+01 | 1.23E-26 | 4.60E-25 |
| QTRTD1 | 1.07E+01 | 1.27E-26 | 4.74E-25 |
| LOC389333 | 1.07E+01 | 1.30E-26 | 4.85E-25 |
| SENP1 | 1.07E+01 | 1.34E-26 | 5.00E-25 |
| SUPT5H | 1.07E+01 | 1.58E-26 | 5.87E-25 |
| SPHK1 | 1.07E+01 | 1.63E-26 | 6.05E-25 |
| NPLOC4 | 1.07E+01 | 1.64E-26 | 6.07E-25 |
| CSE1L | 1.06E+01 | 1.88E-26 | 6.95E-25 |
| RBM15B | 1.06E+01 | 2.03E-26 | 7.47E-25 |
| TRMT1 | 1.06E+01 | 2.04E-26 | 7.49E-25 |
| FANCB | 1.06E+01 | 2.24E-26 | 8.23E-25 |
| CIZ1 | 1.06E+01 | 2.27E-26 | 8.31E-25 |
| LOC339674 | 1.06E+01 | 2.50E-26 | 9.14E-25 |
| TOPBP1 | 1.06E+01 | 2.62E-26 | 9.58E-25 |
| REXO1 | 1.06E+01 | 3.08E-26 | 1.12E-24 |
| C19orf29 | 1.06E+01 | 3.12E-26 | 1.13E-24 |
| ECT2 | 1.06E+01 | 3.15E-26 | 1.14E-24 |
| SGTA | 1.06E+01 | 3.20E-26 | 1.16E-24 |
| DIAPH3 | 1.06E+01 | 3.40E-26 | 1.23E-24 |
| NDOR1 | 1.06E+01 | 3.40E-26 | 1.23E-24 |
| MCM3 | 1.06E+01 | 3.45E-26 | 1.24E-24 |
| HIC2 | 1.06E+01 | 3.48E-26 | 1.25E-24 |
| DNAJC5 | 1.06E+01 | 3.61E-26 | 1.30E-24 |
| ATAD2 | 1.06E+01 | 3.77E-26 | 1.35E-24 |
| C20orf20 | 1.06E+01 | 4.30E-26 | 1.54E-24 |
| CDK16 | 1.06E+01 | 4.30E-26 | 1.54E-24 |
| MAZ | 1.06E+01 | 4.66E-26 | 1.66E-24 |
| COASY | 1.06E+01 | 5.04E-26 | 1.79E-24 |
| C19orf26 | 1.06E+01 | 5.06E-26 | 1.80E-24 |
| CCNA2 | 1.05E+01 | 5.90E-26 | 2.09E-24 |
| SAE1 | 1.05E+01 | 7.40E-26 | 2.62E-24 |
| PBK | 1.05E+01 | 8.88E-26 | 3.14E-24 |
| SAFB | 1.05E+01 | 1.11E-25 | 3.91E-24 |
| NUP85 | 1.05E+01 | 1.21E-25 | 4.24E-24 |
| TCERG1 | 1.05E+01 | 1.22E-25 | 4.27E-24 |
| GTPBP3 | 1.05E+01 | 1.36E-25 | 4.77E-24 |
| CHAF1B | 1.05E+01 | 1.38E-25 | 4.82E-24 |
| CARM1 | 1.05E+01 | 1.43E-25 | 5.00E-24 |
| WDR5 | 1.05E+01 | 1.46E-25 | 5.09E-24 |
| YDJC | 1.04E+01 | 1.64E-25 | 5.70E-24 |
| WDHD1 | 1.04E+01 | 1.72E-25 | 5.99E-24 |
| RBM10 | 1.04E+01 | 1.90E-25 | 6.61E-24 |
| LOC401010 | 1.04E+01 | 2.37E-25 | 8.19E-24 |
| SF3B2 | 1.04E+01 | 2.37E-25 | 8.21E-24 |
| SLC16A3 | 1.04E+01 | 2.67E-25 | 9.21E-24 |
| FAM71D | 1.04E+01 | 2.71E-25 | 9.34E-24 |
| FBXL18 | 1.04E+01 | 2.75E-25 | 9.45E-24 |
| TXNRD1 | 1.04E+01 | 2.91E-25 | 1.00E-23 |

| MPP6 | 1.04E+01 | 2.99E-25 | 1.03E-23 |
| --- | --- | --- | --- |
| DRP2 | 1.04E+01 | 3.34E-25 | 1.14E-23 |
| GARS | 1.04E+01 | 3.41E-25 | 1.16E-23 |
| SH3BP1 | 1.04E+01 | 4.10E-25 | 1.40E-23 |
| TATDN2 | 1.03E+01 | 4.51E-25 | 1.53E-23 |
| SMTN | 1.03E+01 | 4.57E-25 | 1.55E-23 |
| UBE3B | 1.03E+01 | 4.88E-25 | 1.66E-23 |
| SMARCC1 | 1.03E+01 | 4.92E-25 | 1.67E-23 |
| C12orf34 | 1.03E+01 | 5.00E-25 | 1.69E-23 |
| FNDC8 | 1.03E+01 | 5.35E-25 | 1.81E-23 |
| SBNO2 | 1.03E+01 | 5.61E-25 | 1.89E-23 |
| UBE2T | 1.03E+01 | 5.80E-25 | 1.95E-23 |
| MORC2 | 1.03E+01 | 5.84E-25 | 1.96E-23 |
| TELO2 | 1.03E+01 | 6.02E-25 | 2.02E-23 |
| HMMR | 1.03E+01 | 6.20E-25 | 2.08E-23 |
| SCARB1 | 1.03E+01 | 6.57E-25 | 2.20E-23 |
| MBD3 | 1.03E+01 | 7.42E-25 | 2.48E-23 |
| CDCA4 | 1.03E+01 | 7.55E-25 | 2.52E-23 |
| ZNF746 | 1.03E+01 | 7.87E-25 | 2.62E-23 |
| INTS8 | 1.03E+01 | 8.26E-25 | 2.74E-23 |
| SNHG4 | 1.03E+01 | 8.46E-25 | 2.81E-23 |
| MCM6 | 1.03E+01 | 9.00E-25 | 2.98E-23 |
| KAT2A | 1.03E+01 | 9.06E-25 | 2.99E-23 |
| NUSAP1 | 1.03E+01 | 9.75E-25 | 3.22E-23 |
| LRRC59 | 1.03E+01 | 9.87E-25 | 3.25E-23 |
| AP3D1 | 1.03E+01 | 1.11E-24 | 3.66E-23 |
| ACACA | 1.03E+01 | 1.12E-24 | 3.68E-23 |
| DHX38 | 1.03E+01 | 1.14E-24 | 3.73E-23 |
| E2F3 | 1.03E+01 | 1.21E-24 | 3.95E-23 |
| SMOX | 1.02E+01 | 1.25E-24 | 4.10E-23 |
| ANAPC1 | 1.02E+01 | 1.27E-24 | 4.13E-23 |
| ZNF384 | 1.02E+01 | 1.34E-24 | 4.37E-23 |
| COPS7B | 1.02E+01 | 1.43E-24 | 4.65E-23 |
| CIC | 1.02E+01 | 1.47E-24 | 4.77E-23 |
| NUDT1 | 1.02E+01 | 1.54E-24 | 4.98E-23 |
| DNAJC14 | 1.02E+01 | 1.56E-24 | 5.05E-23 |
| POLRMT | 1.02E+01 | 1.60E-24 | 5.17E-23 |
| TOP1MT | 1.02E+01 | 1.60E-24 | 5.17E-23 |
| TSSC1 | 1.02E+01 | 1.61E-24 | 5.18E-23 |
| HAUS6 | 1.02E+01 | 1.69E-24 | 5.44E-23 |
| PRPF31 | 1.02E+01 | 1.74E-24 | 5.59E-23 |
| DENND4B | 1.02E+01 | 1.79E-24 | 5.74E-23 |
| POC1A | 1.02E+01 | 2.00E-24 | 6.38E-23 |
| TERT | 1.02E+01 | 2.04E-24 | 6.52E-23 |
| MEX3A | 1.02E+01 | 2.08E-24 | 6.63E-23 |
| JMJD6 | 1.02E+01 | 2.61E-24 | 8.31E-23 |
| PHRF1 | 1.02E+01 | 2.85E-24 | 9.04E-23 |
| MAD2L1 | 1.02E+01 | 2.86E-24 | 9.07E-23 |
| EP400NL | 1.02E+01 | 2.91E-24 | 9.23E-23 |
| LRRC61 | 1.02E+01 | 2.97E-24 | 9.40E-23 |
| ZNF777 | 1.02E+01 | 3.38E-24 | 1.07E-22 |
| FER1L4 | 1.02E+01 | 3.41E-24 | 1.08E-22 |
| CDK1 | 1.02E+01 | 3.46E-24 | 1.09E-22 |
| PDAP1 | 1.01E+01 | 3.86E-24 | 1.21E-22 |
| STX1A | 1.01E+01 | 4.05E-24 | 1.27E-22 |
| FKBP4 | 1.01E+01 | 4.20E-24 | 1.32E-22 |
| TRIM11 | 1.01E+01 | 4.32E-24 | 1.35E-22 |
| NUP107 | 1.01E+01 | 4.46E-24 | 1.39E-22 |

| CDKN3 |  | 1.01E+01 | 5.08E-24 | 1.59E-22 |
| --- | --- | --- | --- | --- |
|  | 9-Sep | 1.01E+01 | 5.13E-24 | 1.60E-22 |
| REPIN1 |  | 1.01E+01 | 5.18E-24 | 1.61E-22 |
| SARS2 |  | 1.01E+01 | 5.81E-24 | 1.80E-22 |
| NEURL4 |  | 1.01E+01 | 6.04E-24 | 1.87E-22 |
| COBRA1 |  | 1.01E+01 | 6.48E-24 | 2.00E-22 |
| RANBP1 |  | 1.01E+01 | 6.63E-24 | 2.05E-22 |
| RTEL1 |  | 1.01E+01 | 7.34E-24 | 2.26E-22 |
| CTDP1 |  | 1.01E+01 | 7.47E-24 | 2.30E-22 |
| CCT3 |  | 1.01E+01 | 7.62E-24 | 2.34E-22 |
| IGF2BP1 |  | 1.01E+01 | 7.81E-24 | 2.40E-22 |
| MICALL1 |  | 1.01E+01 | 8.61E-24 | 2.64E-22 |
| RAD54B |  | 1.01E+01 | 8.81E-24 | 2.70E-22 |
| MED15 |  | 1.01E+01 | 8.86E-24 | 2.71E-22 |
| RNF31 |  | 1.01E+01 | 9.19E-24 | 2.80E-22 |
| CLCN2 |  | 1.01E+01 | 9.45E-24 | 2.88E-22 |
| SNRPF |  | 1.00E+01 | 9.85E-24 | 2.99E-22 |
| SASS6 |  | 1.00E+01 | 1.20E-23 | 3.64E-22 |
| TPCN1 |  | 1.00E+01 | 1.23E-23 | 3.74E-22 |
| MAP3K10 |  | 1.00E+01 | 1.34E-23 | 4.07E-22 |
| PHF19 |  | 1.00E+01 | 1.45E-23 | 4.38E-22 |
| GTF2IRD1 |  | 1.00E+01 | 1.54E-23 | 4.63E-22 |
| KIAA0195 |  | 1.00E+01 | 1.54E-23 | 4.65E-22 |
| C7orf29 |  | 1.00E+01 | 1.55E-23 | 4.67E-22 |
| SAFB2 |  | 9.99E+00 | 1.67E-23 | 5.03E-22 |
| ZFP64 |  | 9.98E+00 | 1.86E-23 | 5.59E-22 |
| PAXIP1 |  | 9.98E+00 | 1.92E-23 | 5.75E-22 |
| PACS1 |  | 9.97E+00 | 1.99E-23 | 5.96E-22 |
| SAMD4B |  | 9.97E+00 | 2.01E-23 | 5.99E-22 |
| GATSL1 |  | 9.96E+00 | 2.36E-23 | 7.03E-22 |
| NT5DC3 |  | 9.95E+00 | 2.58E-23 | 7.69E-22 |
| DTYMK |  | 9.95E+00 | 2.60E-23 | 7.72E-22 |
| TMEM194A |  | 9.95E+00 | 2.65E-23 | 7.85E-22 |
| OBFC2B |  | 9.94E+00 | 2.67E-23 | 7.92E-22 |
| ZNF512B |  | 9.93E+00 | 2.95E-23 | 8.74E-22 |
| TYMS |  | 9.93E+00 | 3.11E-23 | 9.18E-22 |
| CSNK1E |  | 9.93E+00 | 3.17E-23 | 9.35E-22 |
| TMEM48 |  | 9.93E+00 | 3.20E-23 | 9.42E-22 |
| FBXO5 |  | 9.93E+00 | 3.21E-23 | 9.44E-22 |
| GPRIN1 |  | 9.92E+00 | 3.52E-23 | 1.03E-21 |
| DLGAP4 |  | 9.91E+00 | 3.73E-23 | 1.09E-21 |
| ATIC |  | 9.91E+00 | 3.93E-23 | 1.15E-21 |
| ANKRD13D |  | 9.91E+00 | 3.97E-23 | 1.16E-21 |
| DLG5 |  | 9.90E+00 | 4.21E-23 | 1.23E-21 |
| ATP2A2 |  | 9.90E+00 | 4.23E-23 | 1.23E-21 |
| INTS4 |  | 9.89E+00 | 4.46E-23 | 1.30E-21 |
| CDK2 |  | 9.89E+00 | 4.48E-23 | 1.30E-21 |
| NCKAP5L |  | 9.89E+00 | 4.52E-23 | 1.31E-21 |
| PUF60 |  | 9.89E+00 | 4.61E-23 | 1.34E-21 |
| PIAS4 |  | 9.89E+00 | 4.67E-23 | 1.35E-21 |
| TRAF7 |  | 9.89E+00 | 4.76E-23 | 1.38E-21 |
| DPP3 |  | 9.87E+00 | 5.67E-23 | 1.64E-21 |
| ABCF1 |  | 9.87E+00 | 5.92E-23 | 1.71E-21 |
| ACIN1 |  | 9.86E+00 | 6.05E-23 | 1.74E-21 |
| TMEM104 |  | 9.86E+00 | 6.09E-23 | 1.75E-21 |
| RCE1 |  | 9.86E+00 | 6.33E-23 | 1.82E-21 |
| AURKAPS1 |  | 9.86E+00 | 6.34E-23 | 1.82E-21 |
| C7orf49 |  | 9.86E+00 | 6.40E-23 | 1.83E-21 |

| RNASEH1 | 9.85E+00 | 6.59E-23 | 1.88E-21 |
| --- | --- | --- | --- |
| TUBB | 9.85E+00 | 6.80E-23 | 1.94E-21 |
| MNT | 9.85E+00 | 6.85E-23 | 1.95E-21 |
| POM121 | 9.85E+00 | 7.24E-23 | 2.06E-21 |
| TBC1D10B | 9.84E+00 | 7.65E-23 | 2.17E-21 |
| FAM109A | 9.83E+00 | 8.10E-23 | 2.30E-21 |
| C19orf47 | 9.83E+00 | 8.22E-23 | 2.33E-21 |
| TMEM132A | 9.83E+00 | 8.34E-23 | 2.36E-21 |
| CCT2 | 9.82E+00 | 9.03E-23 | 2.55E-21 |
| DNAJC11 | 9.82E+00 | 9.59E-23 | 2.70E-21 |
| DUS3L | 9.82E+00 | 9.72E-23 | 2.74E-21 |
| PKP3 | 9.81E+00 | 1.05E-22 | 2.96E-21 |
| TRAF2 | 9.81E+00 | 1.07E-22 | 2.99E-21 |
| ALDOA | 9.80E+00 | 1.08E-22 | 3.04E-21 |
| CXXC1 | 9.80E+00 | 1.10E-22 | 3.08E-21 |
| C9orf86 | 9.80E+00 | 1.15E-22 | 3.23E-21 |
| MDC1 | 9.79E+00 | 1.20E-22 | 3.34E-21 |
| ZNF696 | 9.79E+00 | 1.23E-22 | 3.43E-21 |
| TECPR1 | 9.79E+00 | 1.26E-22 | 3.52E-21 |
| UBAP2L | 9.78E+00 | 1.35E-22 | 3.76E-21 |
| E2F7 | 9.78E+00 | 1.45E-22 | 4.02E-21 |
| RNF44 | 9.77E+00 | 1.52E-22 | 4.22E-21 |
| LOC399815 | 9.77E+00 | 1.53E-22 | 4.23E-21 |
| RILPL1 | 9.77E+00 | 1.60E-22 | 4.41E-21 |
| LSG1 | 9.76E+00 | 1.63E-22 | 4.51E-21 |
| PRAME | 9.76E+00 | 1.72E-22 | 4.74E-21 |
| IL4I1 | 9.75E+00 | 1.80E-22 | 4.94E-21 |
| ZNF26 | 9.75E+00 | 1.93E-22 | 5.32E-21 |
| C13orf34 | 9.74E+00 | 2.07E-22 | 5.69E-21 |
| MLL2 | 9.74E+00 | 2.09E-22 | 5.72E-21 |
| GGA3 | 9.74E+00 | 2.14E-22 | 5.86E-21 |
| PRR12 | 9.73E+00 | 2.32E-22 | 6.35E-21 |
| SBF1 | 9.71E+00 | 2.68E-22 | 7.32E-21 |
| PLXNA3 | 9.71E+00 | 2.77E-22 | 7.54E-21 |
| VGF | 9.69E+00 | 3.41E-22 | 9.29E-21 |
| NAT9 | 9.68E+00 | 3.61E-22 | 9.83E-21 |
| BRCA2 | 9.67E+00 | 3.90E-22 | 1.06E-20 |
| TNPO2 | 9.67E+00 | 4.12E-22 | 1.12E-20 |
| DCAF13 | 9.67E+00 | 4.15E-22 | 1.12E-20 |
| CDK4 | 9.66E+00 | 4.30E-22 | 1.16E-20 |
| NCOA5 | 9.66E+00 | 4.40E-22 | 1.19E-20 |
| PRICKLE3 | 9.66E+00 | 4.40E-22 | 1.19E-20 |
| DHX57 | 9.66E+00 | 4.52E-22 | 1.22E-20 |
| WDR43 | 9.66E+00 | 4.67E-22 | 1.26E-20 |
| PRR19 | 9.65E+00 | 5.12E-22 | 1.38E-20 |
| CPSF6 | 9.64E+00 | 5.20E-22 | 1.40E-20 |
| PLCB3 | 9.64E+00 | 5.25E-22 | 1.41E-20 |
| SPPL3 | 9.64E+00 | 5.25E-22 | 1.41E-20 |
| KIF4B | 9.64E+00 | 5.68E-22 | 1.52E-20 |
| SUPT6H | 9.63E+00 | 5.93E-22 | 1.58E-20 |
| PLEKHA9 | 9.62E+00 | 6.52E-22 | 1.74E-20 |
| C7orf43 | 9.62E+00 | 6.55E-22 | 1.74E-20 |
| LIG3 | 9.62E+00 | 6.62E-22 | 1.76E-20 |
| SMARCC2 | 9.61E+00 | 7.02E-22 | 1.86E-20 |
| GAPDH | 9.61E+00 | 7.13E-22 | 1.89E-20 |
| NOP58 | 9.61E+00 | 7.34E-22 | 1.94E-20 |
| PKN1 | 9.61E+00 | 7.64E-22 | 2.02E-20 |
| SEC61A1 | 9.60E+00 | 8.11E-22 | 2.14E-20 |

| HNRNPU | 9.60E+00 | 8.32E-22 | 2.19E-20 |
| --- | --- | --- | --- |
| PTPN11 | 9.60E+00 | 8.33E-22 | 2.19E-20 |
| C5orf34 | 9.59E+00 | 8.46E-22 | 2.22E-20 |
| PSMD2 | 9.59E+00 | 8.57E-22 | 2.25E-20 |
| CCDC77 | 9.59E+00 | 8.81E-22 | 2.31E-20 |
| DNA2 | 9.59E+00 | 9.14E-22 | 2.40E-20 |
| NKAIN1 | 9.58E+00 | 9.49E-22 | 2.48E-20 |
| MED12 | 9.58E+00 | 9.53E-22 | 2.49E-20 |
| ZMIZ2 | 9.57E+00 | 1.09E-21 | 2.84E-20 |
| CENPJ | 9.57E+00 | 1.09E-21 | 2.85E-20 |
| POM121C | 9.56E+00 | 1.18E-21 | 3.08E-20 |
| CENPW | 9.56E+00 | 1.21E-21 | 3.15E-20 |
| MRM1 | 9.55E+00 | 1.25E-21 | 3.24E-20 |
| ZNF296 | 9.55E+00 | 1.27E-21 | 3.29E-20 |
| SNRPB | 9.55E+00 | 1.28E-21 | 3.31E-20 |
| ATXN7L2 | 9.55E+00 | 1.34E-21 | 3.45E-20 |
| TRPM2 | 9.54E+00 | 1.37E-21 | 3.55E-20 |
| LLGL1 | 9.54E+00 | 1.41E-21 | 3.64E-20 |
| PRIM1 | 9.52E+00 | 1.74E-21 | 4.49E-20 |
| ZNF664 | 9.52E+00 | 1.75E-21 | 4.51E-20 |
| RDM1 | 9.50E+00 | 2.05E-21 | 5.27E-20 |
| C9orf172 | 9.50E+00 | 2.19E-21 | 5.62E-20 |
| PDSS1 | 9.49E+00 | 2.22E-21 | 5.70E-20 |
| IARS | 9.49E+00 | 2.37E-21 | 6.06E-20 |
| MTL5 | 9.49E+00 | 2.42E-21 | 6.19E-20 |
| UNK | 9.48E+00 | 2.51E-21 | 6.40E-20 |
| ZFP41 | 9.48E+00 | 2.54E-21 | 6.48E-20 |
| C17orf70 | 9.48E+00 | 2.59E-21 | 6.60E-20 |
| GRK6 | 9.46E+00 | 3.12E-21 | 7.92E-20 |
| PTTG1 | 9.45E+00 | 3.40E-21 | 8.62E-20 |
| BAT2L1 | 9.45E+00 | 3.42E-21 | 8.67E-20 |
| SNAPC4 | 9.44E+00 | 3.70E-21 | 9.38E-20 |
| EHMT1 | 9.44E+00 | 3.84E-21 | 9.70E-20 |
| ATP6V0A2 | 9.43E+00 | 4.17E-21 | 1.05E-19 |
| RNASEH2A | 9.43E+00 | 4.24E-21 | 1.07E-19 |
| CCT7 | 9.42E+00 | 4.32E-21 | 1.09E-19 |
| FARSB | 9.42E+00 | 4.56E-21 | 1.15E-19 |
| ZCCHC8 | 9.42E+00 | 4.68E-21 | 1.18E-19 |
| TMPO | 9.42E+00 | 4.72E-21 | 1.18E-19 |
| HSP90AB1 | 9.41E+00 | 5.05E-21 | 1.27E-19 |
| DAGLA | 9.40E+00 | 5.31E-21 | 1.33E-19 |
| KIAA0406 | 9.40E+00 | 5.33E-21 | 1.33E-19 |
| MYL6B | 9.40E+00 | 5.50E-21 | 1.37E-19 |
| HUWE1 | 9.39E+00 | 5.85E-21 | 1.46E-19 |
| CORO1C | 9.39E+00 | 6.20E-21 | 1.55E-19 |
| GNB1L | 9.38E+00 | 6.34E-21 | 1.58E-19 |
| ABCB9 | 9.38E+00 | 6.82E-21 | 1.69E-19 |
| TSR1 | 9.36E+00 | 7.81E-21 | 1.94E-19 |
| RELT | 9.35E+00 | 8.57E-21 | 2.12E-19 |
| CTU1 | 9.35E+00 | 8.91E-21 | 2.21E-19 |
| RNF126 | 9.34E+00 | 9.31E-21 | 2.30E-19 |
| DDX10 | 9.34E+00 | 9.78E-21 | 2.42E-19 |
| LRRC14 | 9.34E+00 | 9.89E-21 | 2.44E-19 |
| C13orf23 | 9.34E+00 | 9.93E-21 | 2.45E-19 |
| LOC100129637 | 9.34E+00 | 9.97E-21 | 2.45E-19 |
| ZBTB40 | 9.33E+00 | 1.07E-20 | 2.62E-19 |
| EIF4EBP1 | 9.33E+00 | 1.09E-20 | 2.68E-19 |
| YWHAG | 9.33E+00 | 1.11E-20 | 2.71E-19 |

| MRTO4 | 9.32E+00 | 1.14E-20 | 2.78E-19 |
| --- | --- | --- | --- |
| C20orf117 | 9.32E+00 | 1.22E-20 | 2.99E-19 |
| TOP3A | 9.31E+00 | 1.24E-20 | 3.04E-19 |
| ESCO2 | 9.31E+00 | 1.27E-20 | 3.11E-19 |
| TRABD | 9.30E+00 | 1.36E-20 | 3.31E-19 |
| GINS3 | 9.30E+00 | 1.43E-20 | 3.48E-19 |
| HSPD1 | 9.30E+00 | 1.44E-20 | 3.50E-19 |
| PPME1 | 9.29E+00 | 1.54E-20 | 3.73E-19 |
| SUV39H2 | 9.29E+00 | 1.58E-20 | 3.84E-19 |
| TBC1D16 | 9.28E+00 | 1.71E-20 | 4.14E-19 |
| TTLL12 | 9.28E+00 | 1.76E-20 | 4.25E-19 |
| SRC | 9.28E+00 | 1.78E-20 | 4.30E-19 |
| TARBP2 | 9.27E+00 | 1.85E-20 | 4.46E-19 |
| RAI1 | 9.26E+00 | 1.98E-20 | 4.76E-19 |
| ZNF74 | 9.26E+00 | 2.04E-20 | 4.91E-19 |
| TJAP1 | 9.26E+00 | 2.15E-20 | 5.17E-19 |
| PAFAH1B3 | 9.25E+00 | 2.16E-20 | 5.20E-19 |
| TTYH3 | 9.25E+00 | 2.20E-20 | 5.28E-19 |
| SOCS7 | 9.25E+00 | 2.29E-20 | 5.48E-19 |
| REEP4 | 9.25E+00 | 2.30E-20 | 5.50E-19 |
| C19orf55 | 9.25E+00 | 2.32E-20 | 5.55E-19 |
| HN1 | 9.25E+00 | 2.34E-20 | 5.59E-19 |
| NUDCD1 | 9.24E+00 | 2.37E-20 | 5.66E-19 |
| WDR77 | 9.24E+00 | 2.45E-20 | 5.84E-19 |
| ACD | 9.23E+00 | 2.68E-20 | 6.37E-19 |
| MEN1 | 9.23E+00 | 2.71E-20 | 6.44E-19 |
| ZNF280A | 9.22E+00 | 2.93E-20 | 6.95E-19 |
| C22orf9 | 9.22E+00 | 3.02E-20 | 7.16E-19 |
| MAMSTR | 9.21E+00 | 3.29E-20 | 7.79E-19 |
| DGCR8 | 9.21E+00 | 3.36E-20 | 7.95E-19 |
| MINK1 | 9.20E+00 | 3.64E-20 | 8.59E-19 |
| SNRNP200 | 9.20E+00 | 3.71E-20 | 8.76E-19 |
| FBXO41 | 9.19E+00 | 3.78E-20 | 8.90E-19 |
| TEX19 | 9.19E+00 | 3.78E-20 | 8.90E-19 |
| LOC388796 | 9.19E+00 | 3.81E-20 | 8.93E-19 |
| MAP1S | 9.19E+00 | 3.81E-20 | 8.93E-19 |
| WBSCR16 | 9.17E+00 | 4.60E-20 | 1.08E-18 |
| NOP14 | 9.17E+00 | 4.77E-20 | 1.12E-18 |
| NUP155 | 9.17E+00 | 4.82E-20 | 1.13E-18 |
| KIAA0101 | 9.16E+00 | 5.04E-20 | 1.18E-18 |
| RBM42 | 9.16E+00 | 5.18E-20 | 1.21E-18 |
| FBXO45 | 9.16E+00 | 5.28E-20 | 1.23E-18 |
| BCORL1 | 9.16E+00 | 5.37E-20 | 1.25E-18 |
| LIN28B | 9.15E+00 | 5.53E-20 | 1.29E-18 |
| SUV39H1 | 9.15E+00 | 5.56E-20 | 1.29E-18 |
| GSK3A | 9.15E+00 | 5.75E-20 | 1.33E-18 |
| NKIRAS2 | 9.15E+00 | 5.80E-20 | 1.34E-18 |
| RIPK2 | 9.15E+00 | 5.90E-20 | 1.36E-18 |
| KIF3C | 9.14E+00 | 6.13E-20 | 1.42E-18 |
| GATAD2A | 9.14E+00 | 6.16E-20 | 1.42E-18 |
| PLEC | 9.13E+00 | 6.67E-20 | 1.54E-18 |
| MYO9B | 9.13E+00 | 6.71E-20 | 1.55E-18 |
| ABCC10 | 9.13E+00 | 7.05E-20 | 1.62E-18 |
| RAP1GAP2 | 9.11E+00 | 8.37E-20 | 1.92E-18 |
| LEMD2 | 9.11E+00 | 8.65E-20 | 1.99E-18 |
| LRP8 | 9.11E+00 | 8.66E-20 | 1.99E-18 |
| CORO7 | 9.10E+00 | 9.04E-20 | 2.07E-18 |
| CCDC124 | 9.10E+00 | 9.10E-20 | 2.08E-18 |

| ODF2 | 9.10E+00 | 9.32E-20 | 2.13E-18 |
| --- | --- | --- | --- |
| TMEM184B | 9.10E+00 | 9.37E-20 | 2.14E-18 |
| CTAG1B | 9.09E+00 | 9.62E-20 | 2.19E-18 |
| OPA3 | 9.09E+00 | 9.96E-20 | 2.27E-18 |
| GAK | 9.09E+00 | 1.01E-19 | 2.29E-18 |
| C21orf45 | 9.09E+00 | 1.01E-19 | 2.30E-18 |
| USP5 | 9.08E+00 | 1.10E-19 | 2.50E-18 |
| C19orf61 | 9.08E+00 | 1.13E-19 | 2.57E-18 |
| ZBTB45 | 9.06E+00 | 1.27E-19 | 2.87E-18 |
| KIF24 | 9.06E+00 | 1.30E-19 | 2.94E-18 |
| SUPT16H | 9.06E+00 | 1.32E-19 | 2.98E-18 |
| PTDSS2 | 9.06E+00 | 1.33E-19 | 3.00E-18 |
| SLC36A1 | 9.05E+00 | 1.46E-19 | 3.30E-18 |
| SFRS16 | 9.05E+00 | 1.47E-19 | 3.32E-18 |
| WDR12 | 9.05E+00 | 1.48E-19 | 3.32E-18 |
| EIF4A3 | 9.04E+00 | 1.51E-19 | 3.40E-18 |
| ZNF740 | 9.04E+00 | 1.54E-19 | 3.45E-18 |
| MMS19 | 9.04E+00 | 1.57E-19 | 3.51E-18 |
| STK35 | 9.04E+00 | 1.57E-19 | 3.52E-18 |
| CHPF2 | 9.03E+00 | 1.70E-19 | 3.80E-18 |
| TPD52L2 | 9.03E+00 | 1.76E-19 | 3.92E-18 |
| MED25 | 9.02E+00 | 1.83E-19 | 4.07E-18 |
| SLC38A7 | 9.02E+00 | 1.85E-19 | 4.12E-18 |
| OTX1 | 9.02E+00 | 1.88E-19 | 4.18E-18 |
| ALG3 | 9.02E+00 | 1.92E-19 | 4.26E-18 |
| NUMBL | 9.01E+00 | 2.02E-19 | 4.48E-18 |
| SCML2 | 9.01E+00 | 2.02E-19 | 4.48E-18 |
| KCNJ14 | 9.01E+00 | 2.05E-19 | 4.54E-18 |
| NAT8L | 9.01E+00 | 2.06E-19 | 4.54E-18 |
| SAP130 | 9.01E+00 | 2.05E-19 | 4.54E-18 |
| PLEKHM2 | 9.01E+00 | 2.13E-19 | 4.71E-18 |
| USP19 | 9.00E+00 | 2.22E-19 | 4.90E-18 |
| APEX2 | 9.00E+00 | 2.29E-19 | 5.05E-18 |
| RUVBL2 | 9.00E+00 | 2.30E-19 | 5.06E-18 |
| TAF4 | 9.00E+00 | 2.37E-19 | 5.20E-18 |
| PRKCSH | 8.99E+00 | 2.43E-19 | 5.33E-18 |
| WDR76 | 8.99E+00 | 2.44E-19 | 5.35E-18 |
| RBL1 | 8.99E+00 | 2.44E-19 | 5.35E-18 |
| ADA | 8.99E+00 | 2.49E-19 | 5.46E-18 |
| FRMD8 | 8.99E+00 | 2.53E-19 | 5.54E-18 |
| SND1 | 8.98E+00 | 2.62E-19 | 5.72E-18 |
| ISL2 | 8.98E+00 | 2.70E-19 | 5.90E-18 |
| C12orf11 | 8.98E+00 | 2.76E-19 | 6.02E-18 |
| KIAA0664 | 8.97E+00 | 2.94E-19 | 6.41E-18 |
| TTC27 | 8.97E+00 | 3.04E-19 | 6.62E-18 |
| RNPS1 | 8.97E+00 | 3.05E-19 | 6.62E-18 |
| ZNF768 | 8.97E+00 | 3.05E-19 | 6.62E-18 |
| SF3B3 | 8.96E+00 | 3.15E-19 | 6.82E-18 |
| AKAP8L | 8.96E+00 | 3.30E-19 | 7.15E-18 |
| LOC642846 | 8.95E+00 | 3.43E-19 | 7.41E-18 |
| EHBP1L1 | 8.95E+00 | 3.43E-19 | 7.42E-18 |
| DCAF15 | 8.95E+00 | 3.46E-19 | 7.46E-18 |
| SHMT2 | 8.95E+00 | 3.64E-19 | 7.85E-18 |
| C12orf51 | 8.95E+00 | 3.71E-19 | 7.98E-18 |
| CASP2 | 8.94E+00 | 3.79E-19 | 8.16E-18 |
| HEATR7A | 8.94E+00 | 3.96E-19 | 8.51E-18 |
| FUS | 8.94E+00 | 4.04E-19 | 8.67E-18 |
| MSH2 | 8.94E+00 | 4.08E-19 | 8.75E-18 |

| TARS | 8.93E+00 | 4.09E-19 | 8.76E-18 |
| --- | --- | --- | --- |
| PXN | 8.92E+00 | 4.53E-19 | 9.69E-18 |
| C18orf54 | 8.92E+00 | 4.80E-19 | 1.03E-17 |
| TRMT6 | 8.92E+00 | 4.84E-19 | 1.03E-17 |
| ANKRD27 | 8.91E+00 | 5.02E-19 | 1.07E-17 |
| EID3 | 8.91E+00 | 5.08E-19 | 1.08E-17 |
| C15orf39 | 8.91E+00 | 5.24E-19 | 1.12E-17 |
| RELL2 | 8.91E+00 | 5.33E-19 | 1.13E-17 |
| C17orf86 | 8.90E+00 | 5.63E-19 | 1.20E-17 |
| C8orf30A | 8.90E+00 | 5.73E-19 | 1.22E-17 |
| BTBD12 | 8.89E+00 | 5.88E-19 | 1.25E-17 |
| ZMYND19 | 8.89E+00 | 5.92E-19 | 1.25E-17 |
| SLC3A2 | 8.89E+00 | 6.16E-19 | 1.30E-17 |
| GAGE4 | 8.89E+00 | 6.30E-19 | 1.33E-17 |
| RHOF | 8.89E+00 | 6.39E-19 | 1.35E-17 |
| ZBED4 | 8.88E+00 | 6.58E-19 | 1.39E-17 |
| SHOX2 | 8.88E+00 | 6.79E-19 | 1.43E-17 |
| FBRS | 8.87E+00 | 7.09E-19 | 1.49E-17 |
| TIMM44 | 8.87E+00 | 7.28E-19 | 1.53E-17 |
| MOGS | 8.87E+00 | 7.36E-19 | 1.55E-17 |
| STC2 | 8.87E+00 | 7.50E-19 | 1.57E-17 |
| HAUS5 | 8.87E+00 | 7.62E-19 | 1.60E-17 |
| KDM2B | 8.86E+00 | 7.80E-19 | 1.63E-17 |
| ZFPL1 | 8.86E+00 | 8.35E-19 | 1.74E-17 |
| ZWILCH | 8.85E+00 | 8.90E-19 | 1.86E-17 |
| SKIV2L | 8.85E+00 | 9.18E-19 | 1.91E-17 |
| LAMA5 | 8.84E+00 | 9.39E-19 | 1.96E-17 |
| RIOK1 | 8.84E+00 | 9.68E-19 | 2.02E-17 |
| ZNF787 | 8.84E+00 | 9.84E-19 | 2.05E-17 |
| SLC25A19 | 8.84E+00 | 1.00E-18 | 2.08E-17 |
| SLC38A1 | 8.83E+00 | 1.03E-18 | 2.13E-17 |
| CDK5R1 | 8.83E+00 | 1.08E-18 | 2.25E-17 |
| ANKLE1 | 8.82E+00 | 1.14E-18 | 2.36E-17 |
| FUBP1 | 8.82E+00 | 1.19E-18 | 2.47E-17 |
| ACRV1 | 8.81E+00 | 1.27E-18 | 2.63E-17 |
| TNRC18 | 8.81E+00 | 1.29E-18 | 2.65E-17 |
| RNF26 | 8.81E+00 | 1.30E-18 | 2.67E-17 |
| GAGE2D | 8.81E+00 | 1.30E-18 | 2.69E-17 |
| NRBP1 | 8.80E+00 | 1.33E-18 | 2.74E-17 |
| STARD3 | 8.79E+00 | 1.45E-18 | 2.97E-17 |
| HSPBP1 | 8.79E+00 | 1.54E-18 | 3.16E-17 |
| FASN | 8.77E+00 | 1.78E-18 | 3.65E-17 |
| HNRNPR | 8.77E+00 | 1.80E-18 | 3.69E-17 |
| CHD4 | 8.77E+00 | 1.83E-18 | 3.74E-17 |
| RPAP3 | 8.77E+00 | 1.86E-18 | 3.80E-17 |
| C3orf26 | 8.77E+00 | 1.87E-18 | 3.81E-17 |
| CDV3 | 8.76E+00 | 1.91E-18 | 3.90E-17 |
| ZNF408 | 8.76E+00 | 1.92E-18 | 3.90E-17 |
| ERCC2 | 8.76E+00 | 1.99E-18 | 4.06E-17 |
| ABCF3 | 8.75E+00 | 2.07E-18 | 4.21E-17 |
| ANKRD11 | 8.75E+00 | 2.15E-18 | 4.36E-17 |
| YIF1B | 8.75E+00 | 2.19E-18 | 4.45E-17 |
| TRAFD1 | 8.74E+00 | 2.24E-18 | 4.55E-17 |
| NFRKB | 8.74E+00 | 2.31E-18 | 4.67E-17 |
| LTV1 | 8.74E+00 | 2.31E-18 | 4.68E-17 |
| AP1B1 | 8.72E+00 | 2.71E-18 | 5.47E-17 |
| LRFN4 | 8.72E+00 | 2.71E-18 | 5.48E-17 |
| SRF | 8.72E+00 | 2.75E-18 | 5.55E-17 |

| HDAC2 | 8.72E+00 | 2.82E-18 | 5.68E-17 |
| --- | --- | --- | --- |
| KLHL18 | 8.72E+00 | 2.90E-18 | 5.84E-17 |
| CENPK | 8.71E+00 | 2.92E-18 | 5.87E-17 |
| DMPK | 8.71E+00 | 2.92E-18 | 5.87E-17 |
| RFC3 | 8.72E+00 | 2.92E-18 | 5.87E-17 |
| PRPF40B | 8.71E+00 | 3.06E-18 | 6.13E-17 |
| YARS2 | 8.71E+00 | 3.13E-18 | 6.28E-17 |
| NOL11 | 8.71E+00 | 3.14E-18 | 6.29E-17 |
| AMIGO3 | 8.70E+00 | 3.33E-18 | 6.65E-17 |
| SRPK1 | 8.70E+00 | 3.42E-18 | 6.82E-17 |
| MBD1 | 8.70E+00 | 3.47E-18 | 6.93E-17 |
| TUBB3 | 8.69E+00 | 3.50E-18 | 6.98E-17 |
| BRMS1 | 8.69E+00 | 3.59E-18 | 7.15E-17 |
| TET3 | 8.69E+00 | 3.61E-18 | 7.17E-17 |
| PPP1R9B | 8.69E+00 | 3.78E-18 | 7.51E-17 |
| GMPS | 8.67E+00 | 4.16E-18 | 8.27E-17 |
| RNASEN | 8.67E+00 | 4.26E-18 | 8.44E-17 |
| UBE2M | 8.67E+00 | 4.32E-18 | 8.56E-17 |
| AXIN1 | 8.66E+00 | 4.69E-18 | 9.27E-17 |
| ZDHHC18 | 8.66E+00 | 4.83E-18 | 9.55E-17 |
| WDR75 | 8.66E+00 | 4.88E-18 | 9.64E-17 |
| NONO | 8.66E+00 | 4.91E-18 | 9.68E-17 |
| ATP2B1 | 8.65E+00 | 4.97E-18 | 9.79E-17 |
| GEMIN5 | 8.65E+00 | 4.97E-18 | 9.80E-17 |
| LRDD | 8.65E+00 | 5.31E-18 | 1.05E-16 |
| EXOSC3 | 8.64E+00 | 5.68E-18 | 1.12E-16 |
| SR140 | 8.64E+00 | 5.89E-18 | 1.16E-16 |
| ZNF574 | 8.63E+00 | 6.02E-18 | 1.18E-16 |
| CCDC18 | 8.62E+00 | 6.61E-18 | 1.30E-16 |
| ECE2 | 8.62E+00 | 6.71E-18 | 1.31E-16 |
| HDGFRP2 | 8.62E+00 | 6.93E-18 | 1.36E-16 |
| MAST2 | 8.62E+00 | 6.97E-18 | 1.36E-16 |
| SIX5 | 8.61E+00 | 7.07E-18 | 1.38E-16 |
| PNPT1 | 8.61E+00 | 7.12E-18 | 1.39E-16 |
| GTF3C5 | 8.61E+00 | 7.66E-18 | 1.49E-16 |
| FAM83G | 8.60E+00 | 8.13E-18 | 1.58E-16 |
| CEP152 | 8.60E+00 | 8.23E-18 | 1.60E-16 |
| LSM14B | 8.59E+00 | 8.38E-18 | 1.63E-16 |
| KIAA0226 | 8.59E+00 | 9.08E-18 | 1.76E-16 |
| WRAP53 | 8.58E+00 | 9.23E-18 | 1.79E-16 |
| GTPBP5 | 8.58E+00 | 9.31E-18 | 1.80E-16 |
| YARS | 8.58E+00 | 9.80E-18 | 1.90E-16 |
| CBX4 | 8.58E+00 | 9.81E-18 | 1.90E-16 |
| NOL10 | 8.57E+00 | 1.01E-17 | 1.96E-16 |
| C15orf23 | 8.57E+00 | 1.02E-17 | 1.96E-16 |
| MAGEA2 | 8.56E+00 | 1.12E-17 | 2.15E-16 |
| DLL3 | 8.56E+00 | 1.12E-17 | 2.17E-16 |
| DIP2B | 8.56E+00 | 1.16E-17 | 2.23E-16 |
| METTL1 | 8.54E+00 | 1.30E-17 | 2.51E-16 |
| GRIN2D | 8.54E+00 | 1.32E-17 | 2.53E-16 |
| WASF1 | 8.54E+00 | 1.38E-17 | 2.65E-16 |
| HNRNPA2B1 | 8.53E+00 | 1.41E-17 | 2.71E-16 |
| DIABLO | 8.53E+00 | 1.44E-17 | 2.76E-16 |
| PA2G4P4 | 8.53E+00 | 1.50E-17 | 2.87E-16 |
| NAIF1 | 8.53E+00 | 1.50E-17 | 2.87E-16 |
| TMUB1 | 8.53E+00 | 1.53E-17 | 2.93E-16 |
| C19orf28 | 8.52E+00 | 1.57E-17 | 2.99E-16 |
| NSUN2 | 8.52E+00 | 1.59E-17 | 3.03E-16 |

| CTSL2 | 8.52E+00 | 1.64E-17 | 3.12E-16 |
| --- | --- | --- | --- |
| PITX1 | 8.52E+00 | 1.65E-17 | 3.13E-16 |
| PPP1R14B | 8.51E+00 | 1.77E-17 | 3.36E-16 |
| IMPDH1 | 8.51E+00 | 1.78E-17 | 3.37E-16 |
| PYCR1 | 8.50E+00 | 1.87E-17 | 3.55E-16 |
| PSMD3 | 8.50E+00 | 1.88E-17 | 3.56E-16 |
| RPS6KA4 | 8.50E+00 | 1.90E-17 | 3.59E-16 |
| MIER2 | 8.50E+00 | 1.91E-17 | 3.60E-16 |
| ATP2A1 | 8.49E+00 | 2.03E-17 | 3.84E-16 |
| KIAA0020 | 8.49E+00 | 2.08E-17 | 3.93E-16 |
| RRP1B | 8.49E+00 | 2.08E-17 | 3.93E-16 |
| FBXO10 | 8.49E+00 | 2.13E-17 | 4.01E-16 |
| INPPL1 | 8.49E+00 | 2.13E-17 | 4.01E-16 |
| TRIB3 | 8.49E+00 | 2.16E-17 | 4.07E-16 |
| TLR9 | 8.48E+00 | 2.19E-17 | 4.12E-16 |
| ATP13A2 | 8.48E+00 | 2.20E-17 | 4.14E-16 |
| TEAD4 | 8.48E+00 | 2.29E-17 | 4.30E-16 |
| WDR45L | 8.48E+00 | 2.35E-17 | 4.40E-16 |
| KCNK9 | 8.47E+00 | 2.39E-17 | 4.47E-16 |
| CTRL | 8.47E+00 | 2.45E-17 | 4.58E-16 |
| ZNF695 | 8.47E+00 | 2.50E-17 | 4.67E-16 |
| MSI1 | 8.47E+00 | 2.54E-17 | 4.74E-16 |
| SLC1A5 | 8.47E+00 | 2.56E-17 | 4.77E-16 |
| SACS | 8.46E+00 | 2.59E-17 | 4.83E-16 |
| PFKP | 8.46E+00 | 2.72E-17 | 5.06E-16 |
| TRMT2A | 8.46E+00 | 2.78E-17 | 5.17E-16 |
| SIN3B | 8.46E+00 | 2.79E-17 | 5.18E-16 |
| ZNF84 | 8.45E+00 | 2.97E-17 | 5.51E-16 |
| ARPC1B | 8.45E+00 | 3.03E-17 | 5.62E-16 |
| TOMM34 | 8.44E+00 | 3.25E-17 | 6.01E-16 |
| VCP | 8.44E+00 | 3.26E-17 | 6.03E-16 |
| CBX1 | 8.43E+00 | 3.46E-17 | 6.40E-16 |
| TEX10 | 8.43E+00 | 3.52E-17 | 6.49E-16 |
| KRBA1 | 8.42E+00 | 3.63E-17 | 6.69E-16 |
| SLC12A9 | 8.42E+00 | 3.68E-17 | 6.78E-16 |
| ZC3HAV1L | 8.42E+00 | 3.71E-17 | 6.83E-16 |
| AMPD2 | 8.42E+00 | 3.75E-17 | 6.90E-16 |
| EIF2S2 | 8.42E+00 | 3.78E-17 | 6.95E-16 |
| CARD14 | 8.42E+00 | 3.87E-17 | 7.11E-16 |
| AKT1S1 | 8.41E+00 | 4.08E-17 | 7.49E-16 |
| DYNC1H1 | 8.41E+00 | 4.22E-17 | 7.74E-16 |
| RNF220 | 8.41E+00 | 4.23E-17 | 7.75E-16 |
| LOC285033 | 8.41E+00 | 4.29E-17 | 7.85E-16 |
| EDC3 | 8.40E+00 | 4.30E-17 | 7.87E-16 |
| LOC349114 | 8.40E+00 | 4.58E-17 | 8.36E-16 |
| SNRPD1 | 8.40E+00 | 4.64E-17 | 8.47E-16 |
| SNHG1 | 8.39E+00 | 4.81E-17 | 8.77E-16 |
| LZTS2 | 8.39E+00 | 4.82E-17 | 8.78E-16 |
| DLX2 | 8.39E+00 | 4.91E-17 | 8.94E-16 |
| DNM1 | 8.39E+00 | 5.03E-17 | 9.15E-16 |
| FBL | 8.39E+00 | 5.08E-17 | 9.23E-16 |
| DHX33 | 8.38E+00 | 5.52E-17 | 1.00E-15 |
| ZNF646 | 8.37E+00 | 5.68E-17 | 1.03E-15 |
| CKAP2 | 8.37E+00 | 5.75E-17 | 1.04E-15 |
| WDR3 | 8.37E+00 | 5.79E-17 | 1.05E-15 |
| CTU2 | 8.37E+00 | 5.80E-17 | 1.05E-15 |
| CCDC150 | 8.37E+00 | 5.84E-17 | 1.05E-15 |
| SKP2 | 8.37E+00 | 5.84E-17 | 1.05E-15 |

| TOP3B | 8.36E+00 | 6.08E-17 | 1.10E-15 |
| --- | --- | --- | --- |
| FBXO43 | 8.36E+00 | 6.31E-17 | 1.14E-15 |
| ANKRD13B | 8.35E+00 | 6.60E-17 | 1.19E-15 |
| SNRPA1 | 8.35E+00 | 6.81E-17 | 1.23E-15 |
| NCBP2 | 8.35E+00 | 7.08E-17 | 1.27E-15 |
| SC65 | 8.35E+00 | 7.12E-17 | 1.28E-15 |
| NRF1 | 8.34E+00 | 7.35E-17 | 1.32E-15 |
| NEURL2 | 8.34E+00 | 7.45E-17 | 1.34E-15 |
| SH2D5 | 8.34E+00 | 7.76E-17 | 1.39E-15 |
| EXOSC4 | 8.33E+00 | 7.97E-17 | 1.43E-15 |
| C2orf44 | 8.33E+00 | 8.12E-17 | 1.45E-15 |
| GLTSCR1 | 8.33E+00 | 8.26E-17 | 1.48E-15 |
| ZFAT | 8.33E+00 | 8.28E-17 | 1.48E-15 |
| FAM171A2 | 8.33E+00 | 8.39E-17 | 1.50E-15 |
| MTA1 | 8.32E+00 | 8.47E-17 | 1.51E-15 |
| DHX16 | 8.32E+00 | 8.65E-17 | 1.54E-15 |
| C7orf61 | 8.32E+00 | 9.13E-17 | 1.62E-15 |
| UBE2Z | 8.31E+00 | 9.32E-17 | 1.66E-15 |
| B3GAT3 | 8.31E+00 | 9.53E-17 | 1.69E-15 |
| PLEKHG2 | 8.31E+00 | 9.80E-17 | 1.74E-15 |
| UBR5 | 8.30E+00 | 1.01E-16 | 1.78E-15 |
| CSK | 8.30E+00 | 1.01E-16 | 1.79E-15 |
| PAK1IP1 | 8.30E+00 | 1.06E-16 | 1.87E-15 |
| BAT3 | 8.29E+00 | 1.10E-16 | 1.94E-15 |
| CORO1B | 8.29E+00 | 1.13E-16 | 2.00E-15 |
| ZNF8 | 8.29E+00 | 1.13E-16 | 2.00E-15 |
| ZSCAN20 | 8.29E+00 | 1.16E-16 | 2.05E-15 |
| PPFIA4 | 8.28E+00 | 1.19E-16 | 2.10E-15 |
| ISY1 | 8.28E+00 | 1.27E-16 | 2.23E-15 |
| B3GNTL1 | 8.27E+00 | 1.29E-16 | 2.27E-15 |
| ENO1 | 8.27E+00 | 1.32E-16 | 2.31E-15 |
| BTBD2 | 8.27E+00 | 1.34E-16 | 2.36E-15 |
| DUSP13 | 8.27E+00 | 1.35E-16 | 2.36E-15 |
| ZNF473 | 8.27E+00 | 1.37E-16 | 2.40E-15 |
| SENP3 | 8.27E+00 | 1.38E-16 | 2.41E-15 |
| AARS2 | 8.27E+00 | 1.39E-16 | 2.42E-15 |
| CASC5 | 8.26E+00 | 1.42E-16 | 2.48E-15 |
| DDX49 | 8.26E+00 | 1.49E-16 | 2.59E-15 |
| CCDC21 | 8.25E+00 | 1.61E-16 | 2.81E-15 |
| SLC2A1 | 8.25E+00 | 1.62E-16 | 2.83E-15 |
| DDX56 | 8.25E+00 | 1.63E-16 | 2.84E-15 |
| DNAJC9 | 8.24E+00 | 1.66E-16 | 2.89E-15 |
| NME1 | 8.23E+00 | 1.81E-16 | 3.14E-15 |
| ARTN | 8.23E+00 | 1.92E-16 | 3.34E-15 |
| TMEM206 | 8.23E+00 | 1.95E-16 | 3.37E-15 |
| GRB2 | 8.22E+00 | 2.03E-16 | 3.51E-15 |
| CENPN | 8.22E+00 | 2.06E-16 | 3.56E-15 |
| SV2A | 8.22E+00 | 2.10E-16 | 3.63E-15 |
| SLC25A39 | 8.21E+00 | 2.17E-16 | 3.75E-15 |
| MYLK2 | 8.21E+00 | 2.19E-16 | 3.78E-15 |
| TRIM65 | 8.21E+00 | 2.28E-16 | 3.94E-15 |
| HDGF | 8.21E+00 | 2.31E-16 | 3.99E-15 |
| SLC26A11 | 8.20E+00 | 2.33E-16 | 4.02E-15 |
| WDR46 | 8.20E+00 | 2.33E-16 | 4.02E-15 |
| HNRNPA0 | 8.20E+00 | 2.51E-16 | 4.31E-15 |
| PNO1 | 8.20E+00 | 2.51E-16 | 4.32E-15 |
| CDK5R2 | 8.19E+00 | 2.56E-16 | 4.40E-15 |
| RACGAP1P | 8.19E+00 | 2.60E-16 | 4.46E-15 |

| PPFIA3 | 8.19E+00 | 2.62E-16 | 4.49E-15 |
| --- | --- | --- | --- |
| ADAMTS20 | 8.19E+00 | 2.65E-16 | 4.53E-15 |
| DTX2 | 8.19E+00 | 2.67E-16 | 4.57E-15 |
| MKL1 | 8.19E+00 | 2.72E-16 | 4.65E-15 |
| SBNO1 | 8.19E+00 | 2.73E-16 | 4.66E-15 |
| NOM1 | 8.18E+00 | 2.83E-16 | 4.83E-15 |
| TBKBP1 | 8.18E+00 | 2.84E-16 | 4.83E-15 |
| GNL3 | 8.18E+00 | 2.84E-16 | 4.84E-15 |
| BRIP1 | 8.18E+00 | 2.86E-16 | 4.86E-15 |
| C12orf56 | 8.18E+00 | 2.86E-16 | 4.87E-15 |
| NR0B1 | 8.17E+00 | 2.98E-16 | 5.05E-15 |
| TCF19 | 8.17E+00 | 2.99E-16 | 5.07E-15 |
| H2AFZ | 8.17E+00 | 3.18E-16 | 5.38E-15 |
| MGC87042 | 8.17E+00 | 3.20E-16 | 5.41E-15 |
| GAR1 | 8.16E+00 | 3.30E-16 | 5.58E-15 |
| DPF1 | 8.16E+00 | 3.38E-16 | 5.72E-15 |
| PNKP | 8.15E+00 | 3.52E-16 | 5.95E-15 |
| ERF | 8.15E+00 | 3.55E-16 | 5.99E-15 |
| KIF2A | 8.15E+00 | 3.58E-16 | 6.04E-15 |
| NEDD1 | 8.15E+00 | 3.58E-16 | 6.04E-15 |
| MAGEA6 | 8.15E+00 | 3.62E-16 | 6.09E-15 |
| PDLIM7 | 8.15E+00 | 3.69E-16 | 6.21E-15 |
| MLEC | 8.14E+00 | 3.98E-16 | 6.68E-15 |
| RELB | 8.14E+00 | 4.12E-16 | 6.91E-15 |
| SURF6 | 8.13E+00 | 4.16E-16 | 6.98E-15 |
| UBR4 | 8.13E+00 | 4.21E-16 | 7.05E-15 |
| FAM104A | 8.13E+00 | 4.42E-16 | 7.41E-15 |
| TARDBP | 8.12E+00 | 4.62E-16 | 7.73E-15 |
| KREMEN2 | 8.12E+00 | 4.76E-16 | 7.96E-15 |
| KIAA0090 | 8.12E+00 | 4.85E-16 | 8.11E-15 |
| PNCK | 8.12E+00 | 4.87E-16 | 8.14E-15 |
| NACC2 | 8.11E+00 | 4.95E-16 | 8.27E-15 |
| ACTL8 | 8.11E+00 | 5.18E-16 | 8.64E-15 |
| ZNF687 | 8.11E+00 | 5.27E-16 | 8.77E-15 |
| C17orf96 | 8.10E+00 | 5.50E-16 | 9.15E-15 |
| MAP3K11 | 8.10E+00 | 5.64E-16 | 9.37E-15 |
| CCDC123 | 8.10E+00 | 5.68E-16 | 9.44E-15 |
| ASXL1 | 8.10E+00 | 5.71E-16 | 9.47E-15 |
| MAGEA10 | 8.09E+00 | 5.76E-16 | 9.56E-15 |
| MASTL | 8.09E+00 | 5.79E-16 | 9.59E-15 |
| GNL3L | 8.09E+00 | 6.02E-16 | 9.98E-15 |
| B4GALT3 | 8.08E+00 | 6.51E-16 | 1.08E-14 |
| DHX9 | 8.08E+00 | 6.61E-16 | 1.09E-14 |
| MUTYH | 8.07E+00 | 7.02E-16 | 1.16E-14 |
| HNRNPL | 8.07E+00 | 7.11E-16 | 1.17E-14 |
| C17orf63 | 8.07E+00 | 7.19E-16 | 1.19E-14 |
| IPO9 | 8.07E+00 | 7.23E-16 | 1.19E-14 |
| E2F6 | 8.07E+00 | 7.32E-16 | 1.21E-14 |
| TRPC4AP | 8.06E+00 | 7.42E-16 | 1.22E-14 |
| MOV10 | 8.06E+00 | 7.55E-16 | 1.24E-14 |
| PTDSS1 | 8.06E+00 | 7.59E-16 | 1.25E-14 |
| PPIL2 | 8.06E+00 | 7.81E-16 | 1.28E-14 |
| RAPGEF1 | 8.06E+00 | 7.81E-16 | 1.28E-14 |
| CD276 | 8.06E+00 | 7.90E-16 | 1.29E-14 |
| FAM24B | 8.05E+00 | 8.15E-16 | 1.33E-14 |
| BRIX1 | 8.05E+00 | 8.45E-16 | 1.38E-14 |
| ACTG1 | 8.05E+00 | 8.53E-16 | 1.39E-14 |
| MAP2K2 | 8.05E+00 | 8.62E-16 | 1.41E-14 |

| SLC26A6 | 8.05E+00 | 8.65E-16 | 1.41E-14 |
| --- | --- | --- | --- |
| CLCN7 | 8.04E+00 | 8.68E-16 | 1.42E-14 |
| CLEC2L | 8.04E+00 | 8.75E-16 | 1.42E-14 |
| MARK4 | 8.03E+00 | 9.63E-16 | 1.57E-14 |
| C6orf167 | 8.03E+00 | 9.69E-16 | 1.58E-14 |
| PTPRN | 8.03E+00 | 9.85E-16 | 1.60E-14 |
| KCP | 8.03E+00 | 9.93E-16 | 1.61E-14 |
| CSNK2A1 | 8.03E+00 | 1.01E-15 | 1.64E-14 |
| PYCRL | 8.02E+00 | 1.04E-15 | 1.69E-14 |
| HIST2H3C | 8.02E+00 | 1.05E-15 | 1.70E-14 |
| GAGE12J | 8.02E+00 | 1.08E-15 | 1.74E-14 |
| GAS2L3 | 8.01E+00 | 1.13E-15 | 1.82E-14 |
| TGIF2 | 8.01E+00 | 1.13E-15 | 1.83E-14 |
| ALX1 | 8.01E+00 | 1.15E-15 | 1.85E-14 |
| GAGE12D | 8.01E+00 | 1.17E-15 | 1.88E-14 |
| RBM17 | 8.01E+00 | 1.17E-15 | 1.89E-14 |
| ARHGEF18 | 8.00E+00 | 1.22E-15 | 1.97E-14 |
| POPDC3 | 8.00E+00 | 1.25E-15 | 2.00E-14 |
| CDK12 | 8.00E+00 | 1.27E-15 | 2.05E-14 |
| SSB | 8.00E+00 | 1.29E-15 | 2.07E-14 |
| ORAI1 | 7.99E+00 | 1.33E-15 | 2.13E-14 |
| MYADML2 | 7.99E+00 | 1.36E-15 | 2.18E-14 |
| RAE1 | 7.99E+00 | 1.38E-15 | 2.21E-14 |
| SH3GL1 | 7.99E+00 | 1.39E-15 | 2.23E-14 |
| GINS2 | 7.98E+00 | 1.43E-15 | 2.28E-14 |
| ASB6 | 7.98E+00 | 1.49E-15 | 2.39E-14 |
| TSEN2 | 7.97E+00 | 1.54E-15 | 2.46E-14 |
| ERAL1 | 7.97E+00 | 1.54E-15 | 2.46E-14 |
| C2orf48 | 7.97E+00 | 1.54E-15 | 2.46E-14 |
| ISG20L2 | 7.97E+00 | 1.63E-15 | 2.60E-14 |
| NCOA6 | 7.97E+00 | 1.64E-15 | 2.61E-14 |
| RUNDC3A | 7.96E+00 | 1.66E-15 | 2.64E-14 |
| BAZ1B | 7.96E+00 | 1.66E-15 | 2.64E-14 |
| BCAR1 | 7.96E+00 | 1.72E-15 | 2.73E-14 |
| TSSC4 | 7.96E+00 | 1.76E-15 | 2.79E-14 |
| KDM5C | 7.96E+00 | 1.76E-15 | 2.79E-14 |
| SUV420H2 | 7.95E+00 | 1.82E-15 | 2.88E-14 |
| JARID2 | 7.95E+00 | 1.86E-15 | 2.94E-14 |
| SPATA2 | 7.94E+00 | 1.99E-15 | 3.14E-14 |
| UBA1 | 7.94E+00 | 2.07E-15 | 3.28E-14 |
| MCM3APAS | 7.94E+00 | 2.11E-15 | 3.33E-14 |
| ARID3B | 7.93E+00 | 2.17E-15 | 3.43E-14 |
| OAS3 | 7.93E+00 | 2.17E-15 | 3.43E-14 |
| NUP214 | 7.93E+00 | 2.22E-15 | 3.49E-14 |
| MYPOP | 7.93E+00 | 2.22E-15 | 3.49E-14 |
| ASNS | 7.93E+00 | 2.23E-15 | 3.50E-14 |
| TESK1 | 7.92E+00 | 2.29E-15 | 3.60E-14 |
| UTP6 | 7.92E+00 | 2.29E-15 | 3.60E-14 |
| NEURL | 7.91E+00 | 2.50E-15 | 3.93E-14 |
| POLE2 | 7.91E+00 | 2.59E-15 | 4.07E-14 |
| SMARCAL1 | 7.91E+00 | 2.62E-15 | 4.11E-14 |
| C16orf88 | 7.91E+00 | 2.65E-15 | 4.14E-14 |
| TULP1 | 7.90E+00 | 2.70E-15 | 4.22E-14 |
| BCL9L | 7.90E+00 | 2.74E-15 | 4.28E-14 |
| MAGEA3 | 7.90E+00 | 2.81E-15 | 4.38E-14 |
| KLHL17 | 7.90E+00 | 2.81E-15 | 4.39E-14 |
| PDCD2L | 7.90E+00 | 2.82E-15 | 4.40E-14 |
| PPP2R3B | 7.90E+00 | 2.91E-15 | 4.53E-14 |

| DCUN1D5 | 7.89E+00 | 3.12E-15 | 4.86E-14 |
| --- | --- | --- | --- |
| SCG3 | 7.88E+00 | 3.17E-15 | 4.93E-14 |
| FIP1L1 | 7.88E+00 | 3.22E-15 | 5.01E-14 |
| NFKB2 | 7.88E+00 | 3.24E-15 | 5.04E-14 |
| LHX4 | 7.88E+00 | 3.33E-15 | 5.17E-14 |
| DVL1 | 7.88E+00 | 3.34E-15 | 5.18E-14 |
| SMARCB1 | 7.87E+00 | 3.45E-15 | 5.34E-14 |
| RNF40 | 7.87E+00 | 3.46E-15 | 5.35E-14 |
| KCTD17 | 7.87E+00 | 3.61E-15 | 5.58E-14 |
| GDF1 | 7.87E+00 | 3.66E-15 | 5.66E-14 |
| ABL1 | 7.86E+00 | 3.81E-15 | 5.88E-14 |
| RELA | 7.86E+00 | 3.88E-15 | 5.99E-14 |
| TSEN54 | 7.86E+00 | 4.00E-15 | 6.17E-14 |
| ZMYM3 | 7.85E+00 | 4.05E-15 | 6.25E-14 |
| EPN1 | 7.85E+00 | 4.08E-15 | 6.28E-14 |
| SNRNP70 | 7.85E+00 | 4.23E-15 | 6.51E-14 |
| UCN2 | 7.84E+00 | 4.55E-15 | 7.00E-14 |
| CNP | 7.84E+00 | 4.57E-15 | 7.03E-14 |
| PRKD2 | 7.84E+00 | 4.68E-15 | 7.18E-14 |
| EXOC7 | 7.83E+00 | 4.81E-15 | 7.37E-14 |
| FCHO1 | 7.83E+00 | 4.81E-15 | 7.37E-14 |
| C9orf69 | 7.83E+00 | 4.85E-15 | 7.42E-14 |
| PGD | 7.83E+00 | 4.90E-15 | 7.49E-14 |
| TAF5 | 7.83E+00 | 4.96E-15 | 7.58E-14 |
| BANP | 7.83E+00 | 5.03E-15 | 7.68E-14 |
| FLNC | 7.83E+00 | 5.03E-15 | 7.68E-14 |
| DUSP9 | 7.83E+00 | 5.08E-15 | 7.76E-14 |
| NDRG1 | 7.82E+00 | 5.21E-15 | 7.95E-14 |
| ABCC1 | 7.82E+00 | 5.27E-15 | 8.03E-14 |
| TNPO3 | 7.82E+00 | 5.28E-15 | 8.05E-14 |
| CCDC142 | 7.82E+00 | 5.42E-15 | 8.25E-14 |
| APCDD1L | 7.81E+00 | 5.54E-15 | 8.42E-14 |
| IER5L | 7.81E+00 | 5.62E-15 | 8.54E-14 |
| FBF1 | 7.80E+00 | 6.05E-15 | 9.19E-14 |
| SAC3D1 | 7.79E+00 | 6.59E-15 | 9.99E-14 |
| GBF1 | 7.79E+00 | 6.60E-15 | 1.00E-13 |
| C7orf68 | 7.79E+00 | 6.67E-15 | 1.01E-13 |
| ESF1 | 7.79E+00 | 6.99E-15 | 1.06E-13 |
| BAX | 7.78E+00 | 7.11E-15 | 1.08E-13 |
| TKT | 7.78E+00 | 7.25E-15 | 1.10E-13 |
| HOXD11 | 7.77E+00 | 7.59E-15 | 1.15E-13 |
| C12orf73 | 7.77E+00 | 8.09E-15 | 1.22E-13 |
| RAD9A | 7.77E+00 | 8.17E-15 | 1.23E-13 |
| BOLA2 | 7.76E+00 | 8.48E-15 | 1.28E-13 |
| EPRS | 7.75E+00 | 9.38E-15 | 1.41E-13 |
| GTF2H3 | 7.75E+00 | 9.52E-15 | 1.43E-13 |
| MEGF8 | 7.74E+00 | 9.73E-15 | 1.46E-13 |
| MAPRE1 | 7.74E+00 | 9.84E-15 | 1.48E-13 |
| TET1 | 7.74E+00 | 1.02E-14 | 1.53E-13 |
| LIN28A | 7.73E+00 | 1.04E-14 | 1.56E-13 |
| ZNF643 | 7.73E+00 | 1.08E-14 | 1.61E-13 |
| UTP14A | 7.73E+00 | 1.11E-14 | 1.66E-13 |
| TM9SF4 | 7.72E+00 | 1.15E-14 | 1.72E-13 |
| CCDC22 | 7.72E+00 | 1.15E-14 | 1.72E-13 |
| ZNF205 | 7.72E+00 | 1.17E-14 | 1.75E-13 |
| AARS | 7.71E+00 | 1.24E-14 | 1.85E-13 |
| ZC3H4 | 7.71E+00 | 1.26E-14 | 1.87E-13 |
| C12orf24 | 7.71E+00 | 1.28E-14 | 1.91E-13 |

| NFXL1 | 7.71E+00 | 1.28E-14 | 1.91E-13 |
| --- | --- | --- | --- |
| MVD | 7.70E+00 | 1.35E-14 | 2.00E-13 |
| DND1 | 7.70E+00 | 1.39E-14 | 2.07E-13 |
| MMP12 | 7.70E+00 | 1.39E-14 | 2.07E-13 |
| RBM38 | 7.70E+00 | 1.39E-14 | 2.07E-13 |
| MND1 | 7.69E+00 | 1.49E-14 | 2.21E-13 |
| SLC5A11 | 7.69E+00 | 1.52E-14 | 2.25E-13 |
| DAPK3 | 7.68E+00 | 1.57E-14 | 2.33E-13 |
| ASB1 | 7.68E+00 | 1.59E-14 | 2.35E-13 |
| CHEK2 | 7.68E+00 | 1.60E-14 | 2.37E-13 |
| PLOD2 | 7.68E+00 | 1.60E-14 | 2.37E-13 |
| PGS1 | 7.68E+00 | 1.64E-14 | 2.42E-13 |
| C2CD3 | 7.67E+00 | 1.67E-14 | 2.46E-13 |
| GPS1 | 7.67E+00 | 1.68E-14 | 2.47E-13 |
| SLC29A4 | 7.67E+00 | 1.71E-14 | 2.52E-13 |
| WDR85 | 7.67E+00 | 1.74E-14 | 2.56E-13 |
| SIPA1L3 | 7.67E+00 | 1.76E-14 | 2.59E-13 |
| MFI2 | 7.67E+00 | 1.78E-14 | 2.62E-13 |
| CLIP1 | 7.66E+00 | 1.82E-14 | 2.67E-13 |
| FAM60A | 7.66E+00 | 1.83E-14 | 2.69E-13 |
| PSPH | 7.66E+00 | 1.84E-14 | 2.70E-13 |
| XPNPEP1 | 7.66E+00 | 1.89E-14 | 2.77E-13 |
| LRPPRC | 7.66E+00 | 1.94E-14 | 2.83E-13 |
| PRCC | 7.66E+00 | 1.93E-14 | 2.83E-13 |
| MRPL42 | 7.65E+00 | 1.95E-14 | 2.86E-13 |
| SLC7A11 | 7.65E+00 | 1.98E-14 | 2.90E-13 |
| C10orf91 | 7.65E+00 | 2.02E-14 | 2.95E-13 |
| DNAJC7 | 7.65E+00 | 2.03E-14 | 2.96E-13 |
| ZNF623 | 7.65E+00 | 2.07E-14 | 3.03E-13 |
| EIF4H | 7.64E+00 | 2.10E-14 | 3.06E-13 |
| CANT1 | 7.64E+00 | 2.26E-14 | 3.30E-13 |
| CECR7 | 7.63E+00 | 2.27E-14 | 3.30E-13 |
| AKT2 | 7.63E+00 | 2.28E-14 | 3.32E-13 |
| MIB2 | 7.63E+00 | 2.36E-14 | 3.43E-13 |
| CTAG2 | 7.63E+00 | 2.37E-14 | 3.44E-13 |
| OGFR | 7.62E+00 | 2.57E-14 | 3.74E-13 |
| BCL9 | 7.62E+00 | 2.61E-14 | 3.78E-13 |
| GNG4 | 7.61E+00 | 2.66E-14 | 3.85E-13 |
| TP53BP1 | 7.61E+00 | 2.83E-14 | 4.10E-13 |
| TRIM56 | 7.61E+00 | 2.84E-14 | 4.11E-13 |
| TCP1 | 7.61E+00 | 2.84E-14 | 4.11E-13 |
| HYOU1 | 7.61E+00 | 2.86E-14 | 4.13E-13 |
| ZNF697 | 7.60E+00 | 2.88E-14 | 4.16E-13 |
| BHLHA15 | 7.60E+00 | 2.91E-14 | 4.19E-13 |
| KIAA1967 | 7.60E+00 | 3.03E-14 | 4.37E-13 |
| IPPK | 7.59E+00 | 3.17E-14 | 4.57E-13 |
| SSH1 | 7.59E+00 | 3.24E-14 | 4.66E-13 |
| C12orf44 | 7.59E+00 | 3.28E-14 | 4.72E-13 |
| CCR10 | 7.59E+00 | 3.34E-14 | 4.80E-13 |
| MTHFD2 | 7.58E+00 | 3.34E-14 | 4.80E-13 |
| SYNGR3 | 7.58E+00 | 3.36E-14 | 4.82E-13 |
| C20orf72 | 7.58E+00 | 3.38E-14 | 4.85E-13 |
| MAGEA4 | 7.58E+00 | 3.40E-14 | 4.88E-13 |
| LOC645166 | 7.58E+00 | 3.47E-14 | 4.97E-13 |
| USP22 | 7.58E+00 | 3.47E-14 | 4.97E-13 |
| NUP153 | 7.58E+00 | 3.48E-14 | 4.98E-13 |
| RPIA | 7.58E+00 | 3.49E-14 | 4.99E-13 |
| MFSD10 | 7.58E+00 | 3.52E-14 | 5.02E-13 |

| VWA5B2 | 7.58E+00 | 3.55E-14 | 5.07E-13 |
| --- | --- | --- | --- |
| MINA | 7.58E+00 | 3.59E-14 | 5.12E-13 |
| HIST1H3B | 7.57E+00 | 3.62E-14 | 5.16E-13 |
| C1orf112 | 7.57E+00 | 3.66E-14 | 5.21E-13 |
| NME1-NME2 | 7.57E+00 | 3.71E-14 | 5.28E-13 |
| UPF1 | 7.57E+00 | 3.74E-14 | 5.33E-13 |
| E2F5 | 7.57E+00 | 3.77E-14 | 5.35E-13 |
| SLC12A8 | 7.57E+00 | 3.81E-14 | 5.42E-13 |
| MED1 | 7.56E+00 | 3.91E-14 | 5.55E-13 |
| CKS2 | 7.56E+00 | 4.10E-14 | 5.82E-13 |
| IGHMBP2 | 7.56E+00 | 4.20E-14 | 5.95E-13 |
| UCHL1 | 7.55E+00 | 4.24E-14 | 6.01E-13 |
| CFL1 | 7.55E+00 | 4.28E-14 | 6.06E-13 |
| COL7A1 | 7.55E+00 | 4.37E-14 | 6.17E-13 |
| SMC2 | 7.55E+00 | 4.37E-14 | 6.17E-13 |
| TEX15 | 7.55E+00 | 4.37E-14 | 6.17E-13 |
| ACTN4 | 7.55E+00 | 4.39E-14 | 6.19E-13 |
| C4orf21 | 7.55E+00 | 4.52E-14 | 6.37E-13 |
| RAB3B | 7.54E+00 | 4.63E-14 | 6.52E-13 |
| ADAMTS6 | 7.53E+00 | 5.01E-14 | 7.05E-13 |
| PRDM13 | 7.53E+00 | 5.02E-14 | 7.06E-13 |
| ATR | 7.53E+00 | 5.14E-14 | 7.22E-13 |
| CCDC41 | 7.53E+00 | 5.20E-14 | 7.30E-13 |
| EIF3A | 7.53E+00 | 5.22E-14 | 7.33E-13 |
| MPP2 | 7.52E+00 | 5.34E-14 | 7.50E-13 |
| PFN2 | 7.52E+00 | 5.41E-14 | 7.59E-13 |
| PAICS | 7.52E+00 | 5.58E-14 | 7.81E-13 |
| ZNF202 | 7.52E+00 | 5.69E-14 | 7.97E-13 |
| PSMD9 | 7.51E+00 | 5.93E-14 | 8.30E-13 |
| C9orf25 | 7.49E+00 | 6.70E-14 | 9.37E-13 |
| LASS1 | 7.49E+00 | 7.15E-14 | 9.99E-13 |
| CBX8 | 7.48E+00 | 7.19E-14 | 1.00E-12 |
| FAM53C | 7.48E+00 | 7.21E-14 | 1.01E-12 |
| ZYX | 7.48E+00 | 7.61E-14 | 1.06E-12 |
| TNIP2 | 7.47E+00 | 7.85E-14 | 1.09E-12 |
| PUSL1 | 7.47E+00 | 7.92E-14 | 1.10E-12 |
| CDH24 | 7.47E+00 | 8.02E-14 | 1.12E-12 |
| DDX21 | 7.47E+00 | 8.34E-14 | 1.16E-12 |
| IGF2BP2 | 7.46E+00 | 8.39E-14 | 1.17E-12 |
| PFKM | 7.46E+00 | 9.01E-14 | 1.25E-12 |
| DDX31 | 7.45E+00 | 9.15E-14 | 1.27E-12 |
| SLC5A6 | 7.45E+00 | 9.38E-14 | 1.30E-12 |
| SLMO1 | 7.45E+00 | 9.40E-14 | 1.30E-12 |
| ATG4B | 7.44E+00 | 1.01E-13 | 1.39E-12 |
| NASP | 7.44E+00 | 1.03E-13 | 1.43E-12 |
| SIRT7 | 7.43E+00 | 1.06E-13 | 1.47E-12 |
| HAUS8 | 7.43E+00 | 1.07E-13 | 1.48E-12 |
| GIT2 | 7.43E+00 | 1.07E-13 | 1.48E-12 |
| UBFD1 | 7.43E+00 | 1.08E-13 | 1.49E-12 |
| LBR | 7.43E+00 | 1.09E-13 | 1.50E-12 |
| ARHGAP4 | 7.43E+00 | 1.09E-13 | 1.50E-12 |
| ATN1 | 7.43E+00 | 1.10E-13 | 1.51E-12 |
| HNRNPD | 7.43E+00 | 1.10E-13 | 1.52E-12 |
| TRIM16 | 7.43E+00 | 1.12E-13 | 1.54E-12 |
| SLC6A15 | 7.42E+00 | 1.14E-13 | 1.57E-12 |
| PSMD11 | 7.42E+00 | 1.16E-13 | 1.60E-12 |
| ITGA5 | 7.41E+00 | 1.23E-13 | 1.69E-12 |
| PACSIN1 | 7.41E+00 | 1.27E-13 | 1.74E-12 |

| C12orf65 | 7.40E+00 | 1.34E-13 | 1.83E-12 |
| --- | --- | --- | --- |
| PLAC1 | 7.40E+00 | 1.40E-13 | 1.92E-12 |
| C1orf163 | 7.40E+00 | 1.42E-13 | 1.94E-12 |
| C17orf56 | 7.39E+00 | 1.48E-13 | 2.02E-12 |
| GAGE1 | 7.39E+00 | 1.48E-13 | 2.02E-12 |
| POLR3B | 7.39E+00 | 1.48E-13 | 2.03E-12 |
| PRMT5 | 7.39E+00 | 1.51E-13 | 2.06E-12 |
| VAC14 | 7.39E+00 | 1.52E-13 | 2.07E-12 |
| MGC72080 | 7.39E+00 | 1.52E-13 | 2.07E-12 |
| TMEM189 | 7.38E+00 | 1.54E-13 | 2.10E-12 |
| EEF1E1 | 7.38E+00 | 1.56E-13 | 2.12E-12 |
| SNHG3 | 7.38E+00 | 1.56E-13 | 2.12E-12 |
| HTT | 7.38E+00 | 1.62E-13 | 2.20E-12 |
| SMARCA4 | 7.38E+00 | 1.65E-13 | 2.23E-12 |
| SNORD1C | 7.37E+00 | 1.66E-13 | 2.26E-12 |
| WNK2 | 7.37E+00 | 1.67E-13 | 2.27E-12 |
| ZNF579 | 7.37E+00 | 1.68E-13 | 2.28E-12 |
| FAM157A | 7.37E+00 | 1.73E-13 | 2.34E-12 |
| BZW2 | 7.37E+00 | 1.73E-13 | 2.34E-12 |
| BSND | 7.37E+00 | 1.74E-13 | 2.36E-12 |
| SEC24C | 7.36E+00 | 1.85E-13 | 2.50E-12 |
| AP4M1 | 7.36E+00 | 1.86E-13 | 2.51E-12 |
| C17orf62 | 7.36E+00 | 1.87E-13 | 2.52E-12 |
| KHDRBS1 | 7.35E+00 | 1.95E-13 | 2.63E-12 |
| ELL | 7.35E+00 | 1.97E-13 | 2.66E-12 |
| RAD51L3 | 7.34E+00 | 2.07E-13 | 2.79E-12 |
| MRPL12 | 7.34E+00 | 2.09E-13 | 2.81E-12 |
| BLOC1S3 | 7.34E+00 | 2.09E-13 | 2.81E-12 |
| TIPIN | 7.34E+00 | 2.09E-13 | 2.81E-12 |
| GART | 7.34E+00 | 2.12E-13 | 2.84E-12 |
| SH2D2A | 7.34E+00 | 2.14E-13 | 2.87E-12 |
| ALDH18A1 | 7.34E+00 | 2.15E-13 | 2.88E-12 |
| CCT4 | 7.33E+00 | 2.27E-13 | 3.04E-12 |
| RTKN | 7.33E+00 | 2.31E-13 | 3.09E-12 |
| FLAD1 | 7.33E+00 | 2.37E-13 | 3.18E-12 |
| FLRT1 | 7.33E+00 | 2.38E-13 | 3.18E-12 |
| C10orf18 | 7.33E+00 | 2.40E-13 | 3.21E-12 |
| B3GNT4 | 7.32E+00 | 2.41E-13 | 3.22E-12 |
| IMP4 | 7.32E+00 | 2.43E-13 | 3.24E-12 |
| SPRN | 7.32E+00 | 2.45E-13 | 3.26E-12 |
| POLR2A | 7.32E+00 | 2.48E-13 | 3.31E-12 |
| GPR37L1 | 7.32E+00 | 2.49E-13 | 3.32E-12 |
| NARS | 7.32E+00 | 2.55E-13 | 3.39E-12 |
| TPI1 | 7.31E+00 | 2.61E-13 | 3.47E-12 |
| ADAMTS14 | 7.31E+00 | 2.64E-13 | 3.51E-12 |
| YJEFN3 | 7.31E+00 | 2.68E-13 | 3.57E-12 |
| SLC19A1 | 7.31E+00 | 2.73E-13 | 3.63E-12 |
| ZMYND8 | 7.31E+00 | 2.76E-13 | 3.67E-12 |
| HNRNPA3P1 | 7.30E+00 | 2.85E-13 | 3.77E-12 |
| ISM2 | 7.29E+00 | 3.05E-13 | 4.05E-12 |
| LAS1L | 7.29E+00 | 3.13E-13 | 4.14E-12 |
| KIAA0100 | 7.29E+00 | 3.19E-13 | 4.23E-12 |
| SETD5 | 7.28E+00 | 3.26E-13 | 4.32E-12 |
| PDRG1 | 7.28E+00 | 3.28E-13 | 4.33E-12 |
| FAM38A | 7.28E+00 | 3.37E-13 | 4.46E-12 |
| HOXA6 | 7.28E+00 | 3.47E-13 | 4.58E-12 |
| LOC648691 | 7.27E+00 | 3.52E-13 | 4.64E-12 |
| MSTO1 | 7.27E+00 | 3.56E-13 | 4.70E-12 |

| GABRA3 | 7.27E+00 | 3.65E-13 | 4.81E-12 |
| --- | --- | --- | --- |
| DARS2 | 7.27E+00 | 3.68E-13 | 4.84E-12 |
| HAUS7 | 7.26E+00 | 3.82E-13 | 5.02E-12 |
| STXBP5L | 7.26E+00 | 3.88E-13 | 5.11E-12 |
| CTTN | 7.26E+00 | 3.95E-13 | 5.20E-12 |
| DCAF16 | 7.26E+00 | 3.96E-13 | 5.20E-12 |
| GMEB1 | 7.26E+00 | 3.98E-13 | 5.22E-12 |
| PPP1R12C | 7.26E+00 | 3.99E-13 | 5.23E-12 |
| SPOCD1 | 7.25E+00 | 4.04E-13 | 5.29E-12 |
| LASP1 | 7.25E+00 | 4.10E-13 | 5.37E-12 |
| FIGN | 7.24E+00 | 4.49E-13 | 5.88E-12 |
| ZFC3H1 | 7.24E+00 | 4.58E-13 | 5.98E-12 |
| TRMU | 7.23E+00 | 4.81E-13 | 6.29E-12 |
| HMGB2 | 7.23E+00 | 4.91E-13 | 6.42E-12 |
| KISS1R | 7.23E+00 | 4.93E-13 | 6.43E-12 |
| HNRNPA3 | 7.22E+00 | 5.10E-13 | 6.66E-12 |
| FBXW9 | 7.22E+00 | 5.22E-13 | 6.80E-12 |
| RAB3IL1 | 7.22E+00 | 5.33E-13 | 6.94E-12 |
| URB1 | 7.22E+00 | 5.33E-13 | 6.94E-12 |
| FLJ45445 | 7.22E+00 | 5.37E-13 | 6.99E-12 |
| GPR19 | 7.21E+00 | 5.48E-13 | 7.13E-12 |
| MMP9 | 7.21E+00 | 5.52E-13 | 7.17E-12 |
| TLE3 | 7.21E+00 | 5.60E-13 | 7.28E-12 |
| MIIP | 7.20E+00 | 5.92E-13 | 7.68E-12 |
| FAM133A | 7.20E+00 | 5.98E-13 | 7.75E-12 |
| MUS81 | 7.20E+00 | 5.99E-13 | 7.76E-12 |
| REEP2 | 7.20E+00 | 6.25E-13 | 8.09E-12 |
| TRIM46 | 7.19E+00 | 6.46E-13 | 8.37E-12 |
| ARF3 | 7.19E+00 | 6.48E-13 | 8.38E-12 |
| UTP18 | 7.19E+00 | 6.67E-13 | 8.62E-12 |
| SLC7A5P1 | 7.19E+00 | 6.68E-13 | 8.63E-12 |
| NXPH4 | 7.19E+00 | 6.72E-13 | 8.68E-12 |
| FAM111B | 7.18E+00 | 6.95E-13 | 8.96E-12 |
| CD320 | 7.18E+00 | 7.05E-13 | 9.09E-12 |
| SMCR8 | 7.18E+00 | 7.06E-13 | 9.10E-12 |
| LOC286467 | 7.18E+00 | 7.11E-13 | 9.16E-12 |
| KCNV2 | 7.18E+00 | 7.16E-13 | 9.21E-12 |
| CELSR3 | 7.17E+00 | 7.44E-13 | 9.57E-12 |
| C1orf77 | 7.17E+00 | 7.46E-13 | 9.58E-12 |
| LRP12 | 7.17E+00 | 7.78E-13 | 9.99E-12 |
| CEP290 | 7.16E+00 | 7.89E-13 | 1.01E-11 |
| VANGL1 | 7.16E+00 | 7.89E-13 | 1.01E-11 |
| LOC127841 | 7.16E+00 | 7.96E-13 | 1.02E-11 |
| NT5C | 7.16E+00 | 7.99E-13 | 1.02E-11 |
| GANAB | 7.16E+00 | 8.01E-13 | 1.03E-11 |
| ACTR6 | 7.16E+00 | 8.25E-13 | 1.06E-11 |
| EPT1 | 7.16E+00 | 8.30E-13 | 1.06E-11 |
| KIAA1429 | 7.16E+00 | 8.31E-13 | 1.06E-11 |
| ZNF286A | 7.16E+00 | 8.34E-13 | 1.06E-11 |
| GPSM2 | 7.15E+00 | 8.42E-13 | 1.07E-11 |
| CENPL | 7.15E+00 | 8.49E-13 | 1.08E-11 |
| C6orf150 | 7.15E+00 | 8.67E-13 | 1.10E-11 |
| SET | 7.15E+00 | 8.76E-13 | 1.12E-11 |
| CARD10 | 7.15E+00 | 8.97E-13 | 1.14E-11 |
| AIMP2 | 7.14E+00 | 9.05E-13 | 1.15E-11 |
| ARHGDIA | 7.14E+00 | 9.09E-13 | 1.16E-11 |
| HNRNPUL2 | 7.14E+00 | 9.09E-13 | 1.16E-11 |
| POLR1B | 7.14E+00 | 9.23E-13 | 1.17E-11 |

| CDC25B | 7.13E+00 | 9.79E-13 | 1.24E-11 |
| --- | --- | --- | --- |
| MYC | 7.13E+00 | 9.87E-13 | 1.25E-11 |
| CHD8 | 7.13E+00 | 9.89E-13 | 1.25E-11 |
| NUFIP1 | 7.13E+00 | 9.94E-13 | 1.26E-11 |
| WASH3P | 7.13E+00 | 1.02E-12 | 1.29E-11 |
| NCDN | 7.13E+00 | 1.02E-12 | 1.29E-11 |
| BRSK1 | 7.12E+00 | 1.05E-12 | 1.33E-11 |
| SCARNA12 | 7.12E+00 | 1.05E-12 | 1.33E-11 |
| CCDC64 | 7.12E+00 | 1.07E-12 | 1.35E-11 |
| UBQLN4 | 7.12E+00 | 1.08E-12 | 1.36E-11 |
| CBL | 7.12E+00 | 1.10E-12 | 1.39E-11 |
| RECQL5 | 7.12E+00 | 1.11E-12 | 1.40E-11 |
| MAP4 | 7.11E+00 | 1.13E-12 | 1.42E-11 |
| MGC14436 | 7.11E+00 | 1.13E-12 | 1.42E-11 |
| TUBGCP2 | 7.11E+00 | 1.13E-12 | 1.42E-11 |
| LRRC45 | 7.11E+00 | 1.18E-12 | 1.48E-11 |
| SEMA6B | 7.11E+00 | 1.18E-12 | 1.48E-11 |
| CHST11 | 7.11E+00 | 1.19E-12 | 1.50E-11 |
| CALU | 7.11E+00 | 1.19E-12 | 1.50E-11 |
| FSCN1 | 7.11E+00 | 1.20E-12 | 1.50E-11 |
| SLC6A8 | 7.11E+00 | 1.20E-12 | 1.51E-11 |
| SIRT6 | 7.10E+00 | 1.23E-12 | 1.53E-11 |
| CDCP1 | 7.10E+00 | 1.23E-12 | 1.54E-11 |
| PGP | 7.10E+00 | 1.24E-12 | 1.55E-11 |
| MBOAT7 | 7.10E+00 | 1.26E-12 | 1.58E-11 |
| HIST1H1E | 7.10E+00 | 1.29E-12 | 1.61E-11 |
| ZBTB39 | 7.10E+00 | 1.29E-12 | 1.61E-11 |
| RBCK1 | 7.09E+00 | 1.35E-12 | 1.68E-11 |
| FKBP10 | 7.09E+00 | 1.35E-12 | 1.68E-11 |
| PPHLN1 | 7.09E+00 | 1.36E-12 | 1.69E-11 |
| AIFM2 | 7.09E+00 | 1.37E-12 | 1.70E-11 |
| KIFC3 | 7.09E+00 | 1.37E-12 | 1.71E-11 |
| LMTK2 | 7.09E+00 | 1.39E-12 | 1.73E-11 |
| TLL2 | 7.09E+00 | 1.40E-12 | 1.73E-11 |
| RPLP0P2 | 7.08E+00 | 1.40E-12 | 1.74E-11 |
| FXR1 | 7.08E+00 | 1.40E-12 | 1.74E-11 |
| TSSK6 | 7.08E+00 | 1.44E-12 | 1.78E-11 |
| CBX6 | 7.08E+00 | 1.47E-12 | 1.82E-11 |
| RGP1 | 7.08E+00 | 1.48E-12 | 1.83E-11 |
| TNNT1 | 7.08E+00 | 1.49E-12 | 1.84E-11 |
| FOXG1 | 7.07E+00 | 1.50E-12 | 1.86E-11 |
| FAM83H | 7.07E+00 | 1.52E-12 | 1.87E-11 |
| USP42 | 7.07E+00 | 1.53E-12 | 1.89E-11 |
| TRIO | 7.07E+00 | 1.54E-12 | 1.90E-11 |
| CNGB3 | 7.07E+00 | 1.56E-12 | 1.93E-11 |
| SOHLH1 | 7.07E+00 | 1.61E-12 | 1.98E-11 |
| PLCG1 | 7.06E+00 | 1.64E-12 | 2.02E-11 |
| NOP16 | 7.06E+00 | 1.67E-12 | 2.06E-11 |
| PPP5C | 7.06E+00 | 1.70E-12 | 2.09E-11 |
| MLF1IP | 7.06E+00 | 1.70E-12 | 2.09E-11 |
| CLPB | 7.05E+00 | 1.77E-12 | 2.17E-11 |
| BAGE | 7.05E+00 | 1.77E-12 | 2.17E-11 |
| CHRAC1 | 7.05E+00 | 1.78E-12 | 2.18E-11 |
| ZNF414 | 7.05E+00 | 1.83E-12 | 2.25E-11 |
| PNMA1 | 7.04E+00 | 1.92E-12 | 2.36E-11 |
| POLD2 | 7.04E+00 | 1.93E-12 | 2.36E-11 |
| CCNE2 | 7.04E+00 | 1.94E-12 | 2.37E-11 |
| EIF2B5 | 7.04E+00 | 1.97E-12 | 2.41E-11 |

| MIF | 7.04E+00 | 1.98E-12 | 2.42E-11 |
| --- | --- | --- | --- |
| ZBTB2 | 7.04E+00 | 1.99E-12 | 2.44E-11 |
| TLK2 | 7.03E+00 | 2.03E-12 | 2.48E-11 |
| CASC3 | 7.03E+00 | 2.06E-12 | 2.52E-11 |
| KPTN | 7.02E+00 | 2.15E-12 | 2.63E-11 |
| THOC5 | 7.02E+00 | 2.19E-12 | 2.67E-11 |
| CARS | 7.02E+00 | 2.31E-12 | 2.81E-11 |
| POLR3D | 7.01E+00 | 2.36E-12 | 2.88E-11 |
| LSM12 | 7.01E+00 | 2.41E-12 | 2.93E-11 |
| ZNF385A | 7.01E+00 | 2.46E-12 | 2.99E-11 |
| LOC100133161 | 7.01E+00 | 2.48E-12 | 3.01E-11 |
| PCBP4 | 7.00E+00 | 2.53E-12 | 3.07E-11 |
| NAA50 | 7.00E+00 | 2.53E-12 | 3.08E-11 |
| DKFZp761E198 | 7.00E+00 | 2.60E-12 | 3.15E-11 |
| SLC6A17 | 7.00E+00 | 2.60E-12 | 3.15E-11 |
| PKM2 | 7.00E+00 | 2.62E-12 | 3.18E-11 |
| NMT1 | 7.00E+00 | 2.66E-12 | 3.22E-11 |
| USP10 | 6.99E+00 | 2.73E-12 | 3.31E-11 |
| HNF1A | 6.99E+00 | 2.77E-12 | 3.35E-11 |
| KCTD5 | 6.99E+00 | 2.82E-12 | 3.41E-11 |
| AVL9 | 6.98E+00 | 2.90E-12 | 3.51E-11 |
| RBM12B | 6.98E+00 | 2.95E-12 | 3.56E-11 |
| NAA15 | 6.98E+00 | 3.00E-12 | 3.62E-11 |
| TAF2 | 6.98E+00 | 3.02E-12 | 3.64E-11 |
| SYT1 | 6.98E+00 | 3.05E-12 | 3.68E-11 |
| SYT5 | 6.98E+00 | 3.06E-12 | 3.69E-11 |
| SRXN1 | 6.98E+00 | 3.07E-12 | 3.69E-11 |
| CASKIN1 | 6.97E+00 | 3.07E-12 | 3.70E-11 |
| TAF1A | 6.97E+00 | 3.13E-12 | 3.77E-11 |
| LLPH | 6.97E+00 | 3.16E-12 | 3.80E-11 |
| LARS2 | 6.97E+00 | 3.18E-12 | 3.82E-11 |
| TMEM69 | 6.97E+00 | 3.20E-12 | 3.84E-11 |
| GAGE2A | 6.97E+00 | 3.26E-12 | 3.91E-11 |
| CCAR1 | 6.96E+00 | 3.36E-12 | 4.03E-11 |
| SYNCRIP | 6.96E+00 | 3.36E-12 | 4.03E-11 |
| IRF2BP1 | 6.96E+00 | 3.40E-12 | 4.08E-11 |
| GPI | 6.96E+00 | 3.42E-12 | 4.09E-11 |
| TH1L | 6.96E+00 | 3.42E-12 | 4.10E-11 |
| STK40 | 6.96E+00 | 3.44E-12 | 4.11E-11 |
| METTL8 | 6.96E+00 | 3.49E-12 | 4.17E-11 |
| FAM98B | 6.95E+00 | 3.54E-12 | 4.23E-11 |
| PRPF4 | 6.95E+00 | 3.61E-12 | 4.31E-11 |
| EGLN3 | 6.95E+00 | 3.62E-12 | 4.31E-11 |
| TPTE | 6.95E+00 | 3.67E-12 | 4.38E-11 |
| PRDM9 | 6.94E+00 | 3.81E-12 | 4.54E-11 |
| YWHAZ | 6.94E+00 | 3.91E-12 | 4.66E-11 |
| EXOSC2 | 6.94E+00 | 3.93E-12 | 4.67E-11 |
| HNRNPUL1 | 6.94E+00 | 3.93E-12 | 4.68E-11 |
| ZNF532 | 6.93E+00 | 4.08E-12 | 4.85E-11 |
| PIK3R2 | 6.93E+00 | 4.13E-12 | 4.90E-11 |
| MSH5 | 6.92E+00 | 4.42E-12 | 5.24E-11 |
| CDH18 | 6.92E+00 | 4.50E-12 | 5.34E-11 |
| KIAA1984 | 6.92E+00 | 4.56E-12 | 5.41E-11 |
| DNM1L | 6.91E+00 | 4.75E-12 | 5.63E-11 |
| ZNF526 | 6.91E+00 | 4.87E-12 | 5.77E-11 |
| CDC37 | 6.91E+00 | 5.01E-12 | 5.93E-11 |
| CCDC138 | 6.90E+00 | 5.10E-12 | 6.02E-11 |
| RAX | 6.90E+00 | 5.10E-12 | 6.02E-11 |

| GNB2 | 6.90E+00 | 5.27E-12 | 6.22E-11 |
| --- | --- | --- | --- |
| C8orf39 | 6.90E+00 | 5.27E-12 | 6.23E-11 |
| ATG9B | 6.90E+00 | 5.28E-12 | 6.23E-11 |
| MARCKSL1 | 6.90E+00 | 5.29E-12 | 6.24E-11 |
| MAP4K2 | 6.90E+00 | 5.30E-12 | 6.24E-11 |
| PARD3 | 6.90E+00 | 5.37E-12 | 6.32E-11 |
| RNF24 | 6.90E+00 | 5.39E-12 | 6.34E-11 |
| LIF | 6.90E+00 | 5.39E-12 | 6.34E-11 |
| VASP | 6.89E+00 | 5.42E-12 | 6.37E-11 |
| GGA1 | 6.89E+00 | 5.50E-12 | 6.46E-11 |
| CTXN1 | 6.89E+00 | 5.52E-12 | 6.48E-11 |
| ARHGAP19 | 6.89E+00 | 5.59E-12 | 6.56E-11 |
| LOC440905 | 6.89E+00 | 5.62E-12 | 6.59E-11 |
| E4F1 | 6.89E+00 | 5.71E-12 | 6.70E-11 |
| LTBR | 6.89E+00 | 5.73E-12 | 6.71E-11 |
| RHOT2 | 6.89E+00 | 5.76E-12 | 6.74E-11 |
| HOMER1 | 6.88E+00 | 5.83E-12 | 6.83E-11 |
| PABPC4 | 6.88E+00 | 5.93E-12 | 6.93E-11 |
| TPM3 | 6.88E+00 | 5.98E-12 | 6.99E-11 |
| SCYL1 | 6.88E+00 | 5.98E-12 | 6.99E-11 |
| TM4SF19 | 6.88E+00 | 6.04E-12 | 7.05E-11 |
| KCNF1 | 6.88E+00 | 6.05E-12 | 7.05E-11 |
| COPG | 6.88E+00 | 6.11E-12 | 7.12E-11 |
| ATP6V1C1 | 6.88E+00 | 6.12E-12 | 7.14E-11 |
| TNFRSF4 | 6.88E+00 | 6.16E-12 | 7.17E-11 |
| IMMT | 6.88E+00 | 6.20E-12 | 7.21E-11 |
| SLC12A7 | 6.87E+00 | 6.27E-12 | 7.29E-11 |
| IQCE | 6.87E+00 | 6.40E-12 | 7.43E-11 |
| POLA1 | 6.87E+00 | 6.40E-12 | 7.43E-11 |
| HOXA11AS | 6.87E+00 | 6.42E-12 | 7.45E-11 |
| SCAP | 6.87E+00 | 6.44E-12 | 7.47E-11 |
| C10orf12 | 6.87E+00 | 6.49E-12 | 7.52E-11 |
| IKBKG | 6.87E+00 | 6.55E-12 | 7.59E-11 |
| NOL7 | 6.87E+00 | 6.62E-12 | 7.67E-11 |
| FLVCR1 | 6.86E+00 | 6.68E-12 | 7.74E-11 |
| BAT2L2 | 6.86E+00 | 6.72E-12 | 7.77E-11 |
| C6orf195 | 6.86E+00 | 6.89E-12 | 7.97E-11 |
| PRKCG | 6.86E+00 | 6.93E-12 | 8.01E-11 |
| IKBKE | 6.86E+00 | 7.03E-12 | 8.12E-11 |
| ACAN | 6.86E+00 | 7.04E-12 | 8.12E-11 |
| EPS8L3 | 6.86E+00 | 7.09E-12 | 8.18E-11 |
| C19orf50 | 6.85E+00 | 7.29E-12 | 8.41E-11 |
| MYH6 | 6.85E+00 | 7.30E-12 | 8.41E-11 |
| PQLC2 | 6.85E+00 | 7.52E-12 | 8.66E-11 |
| ABCC5 | 6.85E+00 | 7.55E-12 | 8.70E-11 |
| KATNB1 | 6.84E+00 | 7.71E-12 | 8.87E-11 |
| SMC3 | 6.84E+00 | 7.71E-12 | 8.87E-11 |
| PACS2 | 6.84E+00 | 7.80E-12 | 8.96E-11 |
| PCIF1 | 6.84E+00 | 7.85E-12 | 9.01E-11 |
| TFE3 | 6.84E+00 | 7.93E-12 | 9.10E-11 |
| SHARPIN | 6.83E+00 | 8.27E-12 | 9.49E-11 |
| C1orf187 | 6.83E+00 | 8.36E-12 | 9.58E-11 |
| ZNF239 | 6.83E+00 | 8.57E-12 | 9.81E-11 |
| GAL | 6.83E+00 | 8.66E-12 | 9.92E-11 |
| THAP4 | 6.83E+00 | 8.67E-12 | 9.92E-11 |
| CCDC97 | 6.83E+00 | 8.72E-12 | 9.97E-11 |
| EXOSC5 | 6.83E+00 | 8.79E-12 | 1.00E-10 |
| DSCR9 | 6.82E+00 | 8.90E-12 | 1.02E-10 |

| FAM53B | 6.82E+00 | 8.98E-12 | 1.03E-10 |
| --- | --- | --- | --- |
| P2RY6 | 6.82E+00 | 9.00E-12 | 1.03E-10 |
| PSMC4 | 6.82E+00 | 9.30E-12 | 1.06E-10 |
| PNPLA6 | 6.81E+00 | 9.48E-12 | 1.08E-10 |
| HSP90B1 | 6.81E+00 | 9.76E-12 | 1.11E-10 |
| LGALS14 | 6.81E+00 | 9.76E-12 | 1.11E-10 |
| PRPF6 | 6.81E+00 | 9.90E-12 | 1.13E-10 |
| C16orf75 | 6.81E+00 | 9.94E-12 | 1.13E-10 |
| P2RX5 | 6.81E+00 | 9.94E-12 | 1.13E-10 |
| LOC100132287 | 6.80E+00 | 1.02E-11 | 1.16E-10 |
| MTA3 | 6.80E+00 | 1.07E-11 | 1.21E-10 |
| MLST8 | 6.80E+00 | 1.08E-11 | 1.23E-10 |
| RNF4 | 6.79E+00 | 1.10E-11 | 1.24E-10 |
| DONSON | 6.79E+00 | 1.10E-11 | 1.25E-10 |
| C8orf33 | 6.79E+00 | 1.11E-11 | 1.26E-10 |
| MRE11A | 6.79E+00 | 1.13E-11 | 1.27E-10 |
| GRAMD1A | 6.79E+00 | 1.14E-11 | 1.29E-10 |
| GET4 | 6.79E+00 | 1.16E-11 | 1.31E-10 |
| PATL1 | 6.78E+00 | 1.17E-11 | 1.33E-10 |
| GIGYF1 | 6.78E+00 | 1.18E-11 | 1.33E-10 |
| ZNF710 | 6.78E+00 | 1.21E-11 | 1.36E-10 |
| SLC37A3 | 6.78E+00 | 1.23E-11 | 1.39E-10 |
| EIF3C | 6.78E+00 | 1.24E-11 | 1.39E-10 |
| CD7 | 6.78E+00 | 1.24E-11 | 1.39E-10 |
| NFKBIB | 6.77E+00 | 1.28E-11 | 1.44E-10 |
| NRM | 6.77E+00 | 1.28E-11 | 1.44E-10 |
| RAD21 | 6.77E+00 | 1.28E-11 | 1.44E-10 |
| HSPA4 | 6.77E+00 | 1.30E-11 | 1.46E-10 |
| HMBS | 6.77E+00 | 1.30E-11 | 1.46E-10 |
| ELK1 | 6.77E+00 | 1.33E-11 | 1.49E-10 |
| CHD3 | 6.76E+00 | 1.34E-11 | 1.51E-10 |
| SH3BP5L | 6.76E+00 | 1.35E-11 | 1.52E-10 |
| XIRP1 | 6.76E+00 | 1.36E-11 | 1.52E-10 |
| TBC1D3 | 6.76E+00 | 1.41E-11 | 1.58E-10 |
| SH3PXD2B | 6.76E+00 | 1.42E-11 | 1.59E-10 |
| SLC29A2 | 6.76E+00 | 1.43E-11 | 1.60E-10 |
| TAF15 | 6.76E+00 | 1.43E-11 | 1.60E-10 |
| ZKSCAN5 | 6.75E+00 | 1.47E-11 | 1.64E-10 |
| COL11A1 | 6.75E+00 | 1.48E-11 | 1.65E-10 |
| PLAGL2 | 6.75E+00 | 1.48E-11 | 1.66E-10 |
| SFRS1 | 6.75E+00 | 1.48E-11 | 1.66E-10 |
| NBEAL2 | 6.75E+00 | 1.49E-11 | 1.66E-10 |
| VPS72 | 6.75E+00 | 1.54E-11 | 1.71E-10 |
| LEPRE1 | 6.74E+00 | 1.55E-11 | 1.73E-10 |
| HOXD13 | 6.74E+00 | 1.56E-11 | 1.74E-10 |
| UGGT1 | 6.74E+00 | 1.57E-11 | 1.75E-10 |
| NBPF4 | 6.74E+00 | 1.62E-11 | 1.80E-10 |
| RCCD1 | 6.73E+00 | 1.66E-11 | 1.84E-10 |
| NEFM | 6.73E+00 | 1.68E-11 | 1.87E-10 |
| CBFA2T2 | 6.73E+00 | 1.75E-11 | 1.94E-10 |
| HOXB9 | 6.72E+00 | 1.81E-11 | 2.01E-10 |
| SAMD10 | 6.72E+00 | 1.83E-11 | 2.03E-10 |
| POTEG | 6.72E+00 | 1.85E-11 | 2.05E-10 |
| NF2 | 6.72E+00 | 1.87E-11 | 2.07E-10 |
| ZYG11A | 6.72E+00 | 1.88E-11 | 2.09E-10 |
| CENPB | 6.71E+00 | 1.93E-11 | 2.14E-10 |
| BICD1 | 6.71E+00 | 1.94E-11 | 2.15E-10 |
| TUBB2B | 6.71E+00 | 1.96E-11 | 2.17E-10 |

| RNF214 | 6.71E+00 | 1.96E-11 | 2.17E-10 |
| --- | --- | --- | --- |
| VRK1 | 6.71E+00 | 1.99E-11 | 2.20E-10 |
| SNX8 | 6.71E+00 | 2.01E-11 | 2.22E-10 |
| TBC1D13 | 6.71E+00 | 2.01E-11 | 2.22E-10 |
| MXD3 | 6.70E+00 | 2.02E-11 | 2.23E-10 |
| HEATR3 | 6.70E+00 | 2.03E-11 | 2.24E-10 |
| GNA12 | 6.69E+00 | 2.21E-11 | 2.44E-10 |
| UBN1 | 6.69E+00 | 2.23E-11 | 2.45E-10 |
| ADAT3 | 6.69E+00 | 2.25E-11 | 2.48E-10 |
| C2orf29 | 6.69E+00 | 2.28E-11 | 2.50E-10 |
| GPR63 | 6.69E+00 | 2.31E-11 | 2.54E-10 |
| CSAG3 | 6.68E+00 | 2.32E-11 | 2.55E-10 |
| TRIM16L | 6.68E+00 | 2.32E-11 | 2.55E-10 |
| MURC | 6.68E+00 | 2.36E-11 | 2.59E-10 |
| ANKS3 | 6.68E+00 | 2.39E-11 | 2.62E-10 |
| CAGE1 | 6.68E+00 | 2.42E-11 | 2.65E-10 |
| SNHG6 | 6.68E+00 | 2.48E-11 | 2.72E-10 |
| RHEBL1 | 6.67E+00 | 2.49E-11 | 2.72E-10 |
| LOC441666 | 6.67E+00 | 2.51E-11 | 2.74E-10 |
| C22orf30 | 6.67E+00 | 2.52E-11 | 2.75E-10 |
| DGCR14 | 6.67E+00 | 2.53E-11 | 2.76E-10 |
| SUPV3L1 | 6.67E+00 | 2.54E-11 | 2.78E-10 |
| VDAC1 | 6.67E+00 | 2.55E-11 | 2.78E-10 |
| ZGPAT | 6.67E+00 | 2.56E-11 | 2.79E-10 |
| TSC22D4 | 6.67E+00 | 2.58E-11 | 2.81E-10 |
| TBL3 | 6.67E+00 | 2.61E-11 | 2.84E-10 |
| RLTPR | 6.66E+00 | 2.66E-11 | 2.90E-10 |
| DHX35 | 6.66E+00 | 2.71E-11 | 2.95E-10 |
| C1QTNF6 | 6.66E+00 | 2.72E-11 | 2.96E-10 |
| TNFRSF18 | 6.66E+00 | 2.77E-11 | 3.02E-10 |
| NPRL3 | 6.66E+00 | 2.80E-11 | 3.04E-10 |
| METTL13 | 6.65E+00 | 2.86E-11 | 3.10E-10 |
| HOMER3 | 6.65E+00 | 2.87E-11 | 3.12E-10 |
| AIM1L | 6.65E+00 | 2.92E-11 | 3.17E-10 |
| PI4KAP2 | 6.65E+00 | 2.92E-11 | 3.17E-10 |
| OTOF | 6.65E+00 | 3.01E-11 | 3.26E-10 |
| PKIB | 6.65E+00 | 3.02E-11 | 3.27E-10 |
| ADAM12 | 6.64E+00 | 3.06E-11 | 3.31E-10 |
| KSR1 | 6.64E+00 | 3.06E-11 | 3.32E-10 |
| HTR2C | 6.64E+00 | 3.09E-11 | 3.34E-10 |
| PPM1F | 6.64E+00 | 3.11E-11 | 3.36E-10 |
| ZNF498 | 6.64E+00 | 3.12E-11 | 3.37E-10 |
| SLC35A2 | 6.64E+00 | 3.12E-11 | 3.37E-10 |
| ATG9A | 6.64E+00 | 3.12E-11 | 3.37E-10 |
| KIAA1549 | 6.64E+00 | 3.15E-11 | 3.40E-10 |
| WSB2 | 6.64E+00 | 3.20E-11 | 3.45E-10 |
| CTBP1 | 6.64E+00 | 3.25E-11 | 3.50E-10 |
| FBXO18 | 6.63E+00 | 3.28E-11 | 3.53E-10 |
| IFRD1 | 6.63E+00 | 3.34E-11 | 3.59E-10 |
| MDH2 | 6.63E+00 | 3.43E-11 | 3.69E-10 |
| LOC147804 | 6.62E+00 | 3.49E-11 | 3.75E-10 |
| CCDC34 | 6.62E+00 | 3.50E-11 | 3.76E-10 |
| SFRS2 | 6.62E+00 | 3.53E-11 | 3.79E-10 |
| GTF3C3 | 6.62E+00 | 3.60E-11 | 3.86E-10 |
| LOC407835 | 6.62E+00 | 3.61E-11 | 3.87E-10 |
| C18orf2 | 6.62E+00 | 3.68E-11 | 3.95E-10 |
| CCDC85C | 6.62E+00 | 3.69E-11 | 3.96E-10 |
| MSI2 | 6.62E+00 | 3.71E-11 | 3.97E-10 |

| GPAA1 | 6.61E+00 | 3.73E-11 | 4.00E-10 |
| --- | --- | --- | --- |
| RTL1 | 6.61E+00 | 3.76E-11 | 4.02E-10 |
| ORAI2 | 6.61E+00 | 3.82E-11 | 4.08E-10 |
| LCN15 | 6.61E+00 | 3.83E-11 | 4.09E-10 |
| C12orf49 | 6.61E+00 | 3.86E-11 | 4.12E-10 |
| SEMA4C | 6.61E+00 | 3.87E-11 | 4.13E-10 |
| NKPD1 | 6.61E+00 | 3.88E-11 | 4.13E-10 |
| RALY | 6.61E+00 | 3.89E-11 | 4.14E-10 |
| TNK2 | 6.61E+00 | 3.91E-11 | 4.17E-10 |
| UHRF1BP1 | 6.61E+00 | 3.95E-11 | 4.21E-10 |
| SFRS13B | 6.60E+00 | 4.06E-11 | 4.33E-10 |
| MAPK7 | 6.60E+00 | 4.12E-11 | 4.38E-10 |
| RRP7B | 6.60E+00 | 4.12E-11 | 4.38E-10 |
| TGS1 | 6.60E+00 | 4.18E-11 | 4.44E-10 |
| FANCF | 6.60E+00 | 4.24E-11 | 4.51E-10 |
| LOC731789 | 6.59E+00 | 4.28E-11 | 4.54E-10 |
| C7orf47 | 6.59E+00 | 4.30E-11 | 4.57E-10 |
| MAF1 | 6.59E+00 | 4.41E-11 | 4.68E-10 |
| POLG2 | 6.59E+00 | 4.41E-11 | 4.68E-10 |
| HOXA11 | 6.59E+00 | 4.43E-11 | 4.69E-10 |
| C13orf29 | 6.59E+00 | 4.46E-11 | 4.72E-10 |
| WDR18 | 6.59E+00 | 4.47E-11 | 4.73E-10 |
| NEUROG3 | 6.59E+00 | 4.50E-11 | 4.76E-10 |
| GAGE2B | 6.58E+00 | 4.61E-11 | 4.87E-10 |
| RIBC2 | 6.58E+00 | 4.64E-11 | 4.90E-10 |
| RFFL | 6.58E+00 | 4.72E-11 | 4.99E-10 |
| ROBO1 | 6.58E+00 | 4.73E-11 | 5.00E-10 |
| UPF3B | 6.58E+00 | 4.84E-11 | 5.10E-10 |
| CAMKV | 6.57E+00 | 4.94E-11 | 5.21E-10 |
| DRAP1 | 6.57E+00 | 5.06E-11 | 5.33E-10 |
| HLTF | 6.57E+00 | 5.08E-11 | 5.35E-10 |
| PDCD1 | 6.57E+00 | 5.12E-11 | 5.39E-10 |
| PABPC1 | 6.57E+00 | 5.17E-11 | 5.44E-10 |
| ARHGAP33 | 6.56E+00 | 5.25E-11 | 5.52E-10 |
| NOB1 | 6.56E+00 | 5.28E-11 | 5.55E-10 |
| YTHDF1 | 6.56E+00 | 5.38E-11 | 5.65E-10 |
| IDI2 | 6.56E+00 | 5.43E-11 | 5.70E-10 |
| MARCKS | 6.56E+00 | 5.46E-11 | 5.73E-10 |
| PLIN3 | 6.56E+00 | 5.46E-11 | 5.73E-10 |
| PYY2 | 6.56E+00 | 5.46E-11 | 5.73E-10 |
| CD2BP2 | 6.56E+00 | 5.53E-11 | 5.80E-10 |
| POTEH | 6.56E+00 | 5.54E-11 | 5.80E-10 |
| STMN1 | 6.56E+00 | 5.54E-11 | 5.80E-10 |
| TMEM161A | 6.55E+00 | 5.86E-11 | 6.13E-10 |
| TRIM25 | 6.55E+00 | 5.87E-11 | 6.13E-10 |
| LOC338799 | 6.55E+00 | 5.93E-11 | 6.20E-10 |
| SSBP4 | 6.55E+00 | 5.96E-11 | 6.22E-10 |
| KIAA0196 | 6.54E+00 | 6.14E-11 | 6.41E-10 |
| RPL36A | 6.54E+00 | 6.18E-11 | 6.45E-10 |
| RFC2 | 6.54E+00 | 6.27E-11 | 6.54E-10 |
| SEMA5B | 6.54E+00 | 6.32E-11 | 6.58E-10 |
| PTTG3P | 6.53E+00 | 6.41E-11 | 6.68E-10 |
| ADSL | 6.53E+00 | 6.56E-11 | 6.83E-10 |
| MDGA2 | 6.53E+00 | 6.56E-11 | 6.83E-10 |
| PVT1 | 6.53E+00 | 6.60E-11 | 6.87E-10 |
| SLC25A3 | 6.53E+00 | 6.65E-11 | 6.91E-10 |
| ZNF496 | 6.52E+00 | 6.95E-11 | 7.22E-10 |
| PNPLA1 | 6.52E+00 | 7.18E-11 | 7.45E-10 |

| UBE2MP1 | 6.51E+00 | 7.53E-11 | 7.82E-10 |
| --- | --- | --- | --- |
| MKI67IP | 6.51E+00 | 7.55E-11 | 7.83E-10 |
| KIAA0556 | 6.51E+00 | 7.73E-11 | 8.02E-10 |
| AKAP8 | 6.51E+00 | 7.76E-11 | 8.04E-10 |
| BIN1 | 6.50E+00 | 7.86E-11 | 8.14E-10 |
| DDX52 | 6.50E+00 | 7.86E-11 | 8.14E-10 |
| HOXC6 | 6.50E+00 | 7.91E-11 | 8.19E-10 |
| KIF5A | 6.50E+00 | 8.06E-11 | 8.33E-10 |
| PRDM15 | 6.50E+00 | 8.10E-11 | 8.37E-10 |
| C8orf51 | 6.50E+00 | 8.21E-11 | 8.48E-10 |
| SUZ12 | 6.50E+00 | 8.24E-11 | 8.51E-10 |
| SLC6A10P | 6.50E+00 | 8.26E-11 | 8.53E-10 |
| ELAC2 | 6.49E+00 | 8.33E-11 | 8.59E-10 |
| FAM128A | 6.49E+00 | 8.46E-11 | 8.72E-10 |
| PHLDA2 | 6.49E+00 | 8.52E-11 | 8.78E-10 |
| SEMA3F | 6.49E+00 | 8.54E-11 | 8.79E-10 |
| SMC1B | 6.49E+00 | 8.60E-11 | 8.85E-10 |
| HIST1H2AH | 6.49E+00 | 8.62E-11 | 8.86E-10 |
| ACAD9 | 6.49E+00 | 8.80E-11 | 9.05E-10 |
| CSAG2 | 6.49E+00 | 8.82E-11 | 9.06E-10 |
| ADCK5 | 6.48E+00 | 9.14E-11 | 9.39E-10 |
| DNAJC10 | 6.48E+00 | 9.19E-11 | 9.44E-10 |
| FTSJ1 | 6.48E+00 | 9.20E-11 | 9.44E-10 |
| C9orf114 | 6.48E+00 | 9.25E-11 | 9.49E-10 |
| ARPC1A | 6.48E+00 | 9.35E-11 | 9.59E-10 |
| TMEM79 | 6.48E+00 | 9.40E-11 | 9.63E-10 |
| PSMD1 | 6.47E+00 | 9.60E-11 | 9.83E-10 |
| TXLNA | 6.47E+00 | 9.88E-11 | 1.01E-09 |
| HMGA2 | 6.47E+00 | 9.91E-11 | 1.01E-09 |
| MAST1 | 6.47E+00 | 9.91E-11 | 1.01E-09 |
| PIGU | 6.47E+00 | 1.00E-10 | 1.02E-09 |
| SLC7A6OS | 6.47E+00 | 1.01E-10 | 1.03E-09 |
| SELV | 6.47E+00 | 1.02E-10 | 1.04E-09 |
| ANKRD33 | 6.46E+00 | 1.02E-10 | 1.04E-09 |
| FHL3 | 6.46E+00 | 1.02E-10 | 1.04E-09 |
| MLLT6 | 6.46E+00 | 1.03E-10 | 1.05E-09 |
| SLC7A5P2 | 6.46E+00 | 1.03E-10 | 1.05E-09 |
| PWWP2B | 6.46E+00 | 1.05E-10 | 1.07E-09 |
| CDK11B | 6.46E+00 | 1.07E-10 | 1.09E-09 |
| HEATR6 | 6.46E+00 | 1.08E-10 | 1.10E-09 |
| MSH6 | 6.45E+00 | 1.13E-10 | 1.15E-09 |
| WRNIP1 | 6.45E+00 | 1.14E-10 | 1.16E-09 |
| LOC388152 | 6.45E+00 | 1.15E-10 | 1.17E-09 |
| NCRNA00095 | 6.45E+00 | 1.16E-10 | 1.17E-09 |
| ZNF259 | 6.45E+00 | 1.16E-10 | 1.18E-09 |
| MYBL1 | 6.44E+00 | 1.22E-10 | 1.24E-09 |
| CYC1 | 6.43E+00 | 1.24E-10 | 1.26E-09 |
| CAMSAP1 | 6.43E+00 | 1.26E-10 | 1.28E-09 |
| PIP5K1C | 6.43E+00 | 1.27E-10 | 1.29E-09 |
| MMD | 6.43E+00 | 1.29E-10 | 1.30E-09 |
| MAD1L1 | 6.43E+00 | 1.30E-10 | 1.31E-09 |
| NEFH | 6.43E+00 | 1.30E-10 | 1.31E-09 |
| EXOSC10 | 6.42E+00 | 1.34E-10 | 1.35E-09 |
| PLEKHG4 | 6.42E+00 | 1.35E-10 | 1.36E-09 |
| CHRNA9 | 6.42E+00 | 1.36E-10 | 1.37E-09 |
| HHIPL2 | 6.42E+00 | 1.36E-10 | 1.37E-09 |
| PPY2 | 6.42E+00 | 1.36E-10 | 1.38E-09 |
| CACNA1E | 6.42E+00 | 1.37E-10 | 1.38E-09 |

| TDRD5 | 6.42E+00 | 1.38E-10 | 1.39E-09 |
| --- | --- | --- | --- |
| EPO | 6.42E+00 | 1.39E-10 | 1.40E-09 |
| PABPN1 | 6.42E+00 | 1.40E-10 | 1.41E-09 |
| DCAF7 | 6.42E+00 | 1.40E-10 | 1.41E-09 |
| LOC92659 | 6.42E+00 | 1.40E-10 | 1.41E-09 |
| CCDC93 | 6.42E+00 | 1.41E-10 | 1.41E-09 |
| ELAVL1 | 6.42E+00 | 1.41E-10 | 1.42E-09 |
| C12orf32 | 6.42E+00 | 1.41E-10 | 1.42E-09 |
| B4GALT2 | 6.41E+00 | 1.43E-10 | 1.43E-09 |
| SLC35E4 | 6.41E+00 | 1.43E-10 | 1.44E-09 |
| ACOT7 | 6.41E+00 | 1.45E-10 | 1.45E-09 |
| MAGEA9B | 6.41E+00 | 1.47E-10 | 1.47E-09 |
| AFP | 6.41E+00 | 1.47E-10 | 1.47E-09 |
| LOC441208 | 6.41E+00 | 1.47E-10 | 1.47E-09 |
| MAP1D | 6.41E+00 | 1.49E-10 | 1.49E-09 |
| YBX2 | 6.41E+00 | 1.50E-10 | 1.50E-09 |
| CIRH1A | 6.41E+00 | 1.50E-10 | 1.50E-09 |
| NUP43 | 6.41E+00 | 1.51E-10 | 1.50E-09 |
| C1QL4 | 6.40E+00 | 1.51E-10 | 1.51E-09 |
| TFRC | 6.40E+00 | 1.53E-10 | 1.52E-09 |
| FLJ25363 | 6.40E+00 | 1.55E-10 | 1.55E-09 |
| IGLON5 | 6.40E+00 | 1.56E-10 | 1.56E-09 |
| C20orf165 | 6.40E+00 | 1.57E-10 | 1.57E-09 |
| ZNF488 | 6.40E+00 | 1.57E-10 | 1.57E-09 |
| TFCP2 | 6.39E+00 | 1.62E-10 | 1.62E-09 |
| MAGEA12 | 6.39E+00 | 1.63E-10 | 1.62E-09 |
| USP21 | 6.39E+00 | 1.65E-10 | 1.64E-09 |
| SETDB1 | 6.39E+00 | 1.66E-10 | 1.65E-09 |
| ASCL2 | 6.39E+00 | 1.69E-10 | 1.68E-09 |
| RRP7A | 6.38E+00 | 1.74E-10 | 1.73E-09 |
| CBX5 | 6.38E+00 | 1.75E-10 | 1.74E-09 |
| ZNF343 | 6.38E+00 | 1.76E-10 | 1.74E-09 |
| HMX2 | 6.38E+00 | 1.76E-10 | 1.75E-09 |
| METTL11A | 6.38E+00 | 1.79E-10 | 1.77E-09 |
| FLII | 6.38E+00 | 1.80E-10 | 1.79E-09 |
| CSTF3 | 6.37E+00 | 1.85E-10 | 1.83E-09 |
| CHML | 6.37E+00 | 1.87E-10 | 1.85E-09 |
| WDR8 | 6.37E+00 | 1.89E-10 | 1.87E-09 |
| CA5BP | 6.37E+00 | 1.90E-10 | 1.88E-09 |
| FAM131C | 6.37E+00 | 1.90E-10 | 1.88E-09 |
| DVWA | 6.37E+00 | 1.92E-10 | 1.90E-09 |
| LPCAT4 | 6.37E+00 | 1.92E-10 | 1.90E-09 |
| RPN2 | 6.37E+00 | 1.93E-10 | 1.90E-09 |
| CDC123 | 6.37E+00 | 1.95E-10 | 1.92E-09 |
| TREML3 | 6.37E+00 | 1.95E-10 | 1.93E-09 |
| SFRS14 | 6.36E+00 | 1.97E-10 | 1.94E-09 |
| PITX2 | 6.36E+00 | 1.97E-10 | 1.94E-09 |
| TRIP12 | 6.36E+00 | 2.00E-10 | 1.97E-09 |
| ZNF786 | 6.36E+00 | 2.01E-10 | 1.97E-09 |
| SRCAP | 6.36E+00 | 2.03E-10 | 1.99E-09 |
| PARG | 6.36E+00 | 2.04E-10 | 2.01E-09 |
| UNC119B | 6.36E+00 | 2.06E-10 | 2.02E-09 |
| DNAH14 | 6.36E+00 | 2.07E-10 | 2.03E-09 |
| ACTR5 | 6.36E+00 | 2.08E-10 | 2.04E-09 |
| MKRN3 | 6.36E+00 | 2.09E-10 | 2.05E-09 |
| HELB | 6.35E+00 | 2.13E-10 | 2.09E-09 |
| PASK | 6.35E+00 | 2.14E-10 | 2.10E-09 |
| FXR2 | 6.35E+00 | 2.15E-10 | 2.10E-09 |

| RFT1 | 6.35E+00 | 2.15E-10 | 2.10E-09 |
| --- | --- | --- | --- |
| ADAMTS7 | 6.35E+00 | 2.16E-10 | 2.11E-09 |
| GTPBP2 | 6.35E+00 | 2.20E-10 | 2.15E-09 |
| NLN | 6.35E+00 | 2.21E-10 | 2.16E-09 |
| GLI4 | 6.35E+00 | 2.22E-10 | 2.16E-09 |
| TUBB2C | 6.34E+00 | 2.24E-10 | 2.18E-09 |
| DUSP5P | 6.34E+00 | 2.27E-10 | 2.22E-09 |
| CEP78 | 6.34E+00 | 2.28E-10 | 2.22E-09 |
| NTNG2 | 6.34E+00 | 2.33E-10 | 2.27E-09 |
| ZNF146 | 6.34E+00 | 2.34E-10 | 2.28E-09 |
| FAM49B | 6.34E+00 | 2.35E-10 | 2.29E-09 |
| ADAM17 | 6.33E+00 | 2.39E-10 | 2.32E-09 |
| P2RY11 | 6.33E+00 | 2.54E-10 | 2.47E-09 |
| MAGEA1 | 6.32E+00 | 2.55E-10 | 2.48E-09 |
| PPP2R1A | 6.32E+00 | 2.62E-10 | 2.54E-09 |
| VWDE | 6.32E+00 | 2.63E-10 | 2.55E-09 |
| YBX1 | 6.32E+00 | 2.69E-10 | 2.61E-09 |
| RPLP0 | 6.32E+00 | 2.69E-10 | 2.61E-09 |
| VPS16 | 6.32E+00 | 2.70E-10 | 2.62E-09 |
| AGRN | 6.31E+00 | 2.76E-10 | 2.67E-09 |
| CLSTN1 | 6.31E+00 | 2.76E-10 | 2.68E-09 |
| KRR1 | 6.31E+00 | 2.77E-10 | 2.68E-09 |
| SSTR3 | 6.31E+00 | 2.80E-10 | 2.71E-09 |
| BUB3 | 6.31E+00 | 2.82E-10 | 2.73E-09 |
| LRCH3 | 6.31E+00 | 2.83E-10 | 2.73E-09 |
| LIN9 | 6.31E+00 | 2.84E-10 | 2.74E-09 |
| CKS1B | 6.31E+00 | 2.87E-10 | 2.77E-09 |
| ACAP3 | 6.31E+00 | 2.88E-10 | 2.78E-09 |
| SNF8 | 6.30E+00 | 2.90E-10 | 2.80E-09 |
| VRK2 | 6.30E+00 | 2.92E-10 | 2.82E-09 |
| TRAP1 | 6.30E+00 | 2.94E-10 | 2.84E-09 |
| SPANXC | 6.30E+00 | 2.97E-10 | 2.87E-09 |
| FUCA2 | 6.29E+00 | 3.11E-10 | 3.00E-09 |
| WASF2 | 6.29E+00 | 3.13E-10 | 3.01E-09 |
| HIRA | 6.29E+00 | 3.16E-10 | 3.04E-09 |
| MBD6 | 6.29E+00 | 3.20E-10 | 3.08E-09 |
| ATG2A | 6.29E+00 | 3.23E-10 | 3.10E-09 |
| PKN3 | 6.29E+00 | 3.23E-10 | 3.10E-09 |
| PLCB1 | 6.29E+00 | 3.23E-10 | 3.10E-09 |
| GAGE2C | 6.29E+00 | 3.28E-10 | 3.14E-09 |
| IL17RA | 6.28E+00 | 3.29E-10 | 3.16E-09 |
| SMC1A | 6.28E+00 | 3.34E-10 | 3.20E-09 |
| PDCL3 | 6.28E+00 | 3.47E-10 | 3.32E-09 |
| DPYSL4 | 6.28E+00 | 3.50E-10 | 3.35E-09 |
| MAVS | 6.27E+00 | 3.57E-10 | 3.42E-09 |
| SSX4 | 6.27E+00 | 3.58E-10 | 3.43E-09 |
| FLJ22536 | 6.27E+00 | 3.64E-10 | 3.49E-09 |
| ARAP1 | 6.27E+00 | 3.67E-10 | 3.51E-09 |
| GAL3ST2 | 6.27E+00 | 3.69E-10 | 3.52E-09 |
| FBXL6 | 6.27E+00 | 3.72E-10 | 3.55E-09 |
| IRF3 | 6.26E+00 | 3.74E-10 | 3.57E-09 |
| ZNF692 | 6.26E+00 | 3.75E-10 | 3.58E-09 |
| SREBF1 | 6.26E+00 | 3.77E-10 | 3.60E-09 |
| SLC39A7 | 6.26E+00 | 3.92E-10 | 3.74E-09 |
| OPRD1 | 6.26E+00 | 3.93E-10 | 3.75E-09 |
| ZC3HC1 | 6.26E+00 | 3.94E-10 | 3.75E-09 |
| TMEM44 | 6.25E+00 | 4.00E-10 | 3.81E-09 |
| MTIF2 | 6.25E+00 | 4.00E-10 | 3.81E-09 |

| FAM193A | 6.25E+00 | 4.02E-10 | 3.83E-09 |
| --- | --- | --- | --- |
| EPS15L1 | 6.25E+00 | 4.09E-10 | 3.89E-09 |
| TPRN | 6.25E+00 | 4.11E-10 | 3.91E-09 |
| CUL7 | 6.25E+00 | 4.14E-10 | 3.93E-09 |
| CDX2 | 6.25E+00 | 4.15E-10 | 3.94E-09 |
| NSD1 | 6.25E+00 | 4.18E-10 | 3.97E-09 |
| IQCD | 6.25E+00 | 4.19E-10 | 3.97E-09 |
| IGF2R | 6.25E+00 | 4.23E-10 | 4.01E-09 |
| RBM45 | 6.24E+00 | 4.33E-10 | 4.11E-09 |
| ABL2 | 6.24E+00 | 4.40E-10 | 4.17E-09 |
| RUSC2 | 6.24E+00 | 4.43E-10 | 4.20E-09 |
| GPR35 | 6.23E+00 | 4.54E-10 | 4.29E-09 |
| RASAL1 | 6.23E+00 | 4.59E-10 | 4.34E-09 |
| NHLH1 | 6.23E+00 | 4.81E-10 | 4.54E-09 |
| XRCC1 | 6.22E+00 | 4.87E-10 | 4.60E-09 |
| LOC729991-MEF2B | 6.22E+00 | 4.91E-10 | 4.64E-09 |
| INGX | 6.22E+00 | 4.97E-10 | 4.70E-09 |
| MAML1 | 6.22E+00 | 5.04E-10 | 4.76E-09 |
| CCDC43 | 6.22E+00 | 5.10E-10 | 4.81E-09 |
| PIGS | 6.22E+00 | 5.12E-10 | 4.82E-09 |
| ANKRD7 | 6.21E+00 | 5.26E-10 | 4.96E-09 |
| DDAH2 | 6.21E+00 | 5.32E-10 | 5.02E-09 |
| CNTNAP4 | 6.21E+00 | 5.39E-10 | 5.07E-09 |
| RABEP2 | 6.21E+00 | 5.39E-10 | 5.08E-09 |
| XAB2 | 6.21E+00 | 5.48E-10 | 5.15E-09 |
| CUZD1 | 6.21E+00 | 5.48E-10 | 5.15E-09 |
| RHOV | 6.20E+00 | 5.52E-10 | 5.19E-09 |
| DPP9 | 6.20E+00 | 5.61E-10 | 5.27E-09 |
| PAGE1 | 6.20E+00 | 5.63E-10 | 5.29E-09 |
| CNO | 6.20E+00 | 5.77E-10 | 5.41E-09 |
| PCDHA2 | 6.20E+00 | 5.78E-10 | 5.42E-09 |
| PSME4 | 6.20E+00 | 5.80E-10 | 5.44E-09 |
| RPS6KB2 | 6.19E+00 | 5.88E-10 | 5.51E-09 |
| CT45A5 | 6.19E+00 | 5.90E-10 | 5.52E-09 |
| DSC2 | 6.19E+00 | 5.95E-10 | 5.57E-09 |
| UCKL1 | 6.19E+00 | 5.99E-10 | 5.61E-09 |
| C7orf51 | 6.19E+00 | 6.09E-10 | 5.69E-09 |
| TGFBRAP1 | 6.19E+00 | 6.13E-10 | 5.73E-09 |
| DNASE1 | 6.19E+00 | 6.16E-10 | 5.76E-09 |
| SCAMP3 | 6.19E+00 | 6.20E-10 | 5.79E-09 |
| GPR119 | 6.18E+00 | 6.32E-10 | 5.90E-09 |
| NOL9 | 6.18E+00 | 6.38E-10 | 5.95E-09 |
| TRIM59 | 6.18E+00 | 6.40E-10 | 5.97E-09 |
| EIF4A1 | 6.18E+00 | 6.42E-10 | 5.99E-09 |
| ZDHHC23 | 6.18E+00 | 6.48E-10 | 6.04E-09 |
| BCL7A | 6.18E+00 | 6.57E-10 | 6.12E-09 |
| PSAT1 | 6.18E+00 | 6.58E-10 | 6.12E-09 |
| PYGO2 | 6.18E+00 | 6.60E-10 | 6.14E-09 |
| POMT2 | 6.17E+00 | 6.73E-10 | 6.26E-09 |
| SGK494 | 6.17E+00 | 6.88E-10 | 6.40E-09 |
| CELF1 | 6.17E+00 | 6.90E-10 | 6.41E-09 |
| CUEDC1 | 6.17E+00 | 6.90E-10 | 6.41E-09 |
| TAOK2 | 6.17E+00 | 6.96E-10 | 6.46E-09 |
| DDX42 | 6.17E+00 | 6.98E-10 | 6.48E-09 |
| SPTAN1 | 6.16E+00 | 7.17E-10 | 6.65E-09 |
| HUS1B | 6.16E+00 | 7.27E-10 | 6.74E-09 |
| GUCA1A | 6.16E+00 | 7.34E-10 | 6.80E-09 |
| STOML2 | 6.16E+00 | 7.38E-10 | 6.83E-09 |

| LOC647946 | 6.16E+00 | 7.45E-10 | 6.89E-09 |
| --- | --- | --- | --- |
| MAP2K7 | 6.15E+00 | 7.55E-10 | 6.99E-09 |
| CXorf48 | 6.15E+00 | 7.58E-10 | 7.01E-09 |
| ALKBH2 | 6.15E+00 | 7.58E-10 | 7.01E-09 |
| SAP30BP | 6.15E+00 | 7.59E-10 | 7.02E-09 |
| PIP4K2C | 6.15E+00 | 7.67E-10 | 7.08E-09 |
| CDCA7 | 6.15E+00 | 7.84E-10 | 7.23E-09 |
| ZNF341 | 6.15E+00 | 7.87E-10 | 7.26E-09 |
| FZR1 | 6.15E+00 | 7.92E-10 | 7.30E-09 |
| MGC57346 | 6.15E+00 | 8.01E-10 | 7.39E-09 |
| RNF213 | 6.14E+00 | 8.05E-10 | 7.42E-09 |
| DNAJB11 | 6.14E+00 | 8.22E-10 | 7.57E-09 |
| GPR157 | 6.14E+00 | 8.31E-10 | 7.65E-09 |
| CABIN1 | 6.14E+00 | 8.34E-10 | 7.67E-09 |
| HDAC7 | 6.14E+00 | 8.47E-10 | 7.79E-09 |
| NAA11 | 6.14E+00 | 8.49E-10 | 7.81E-09 |
| CRTC2 | 6.14E+00 | 8.52E-10 | 7.83E-09 |
| CSNK2A1P | 6.13E+00 | 8.87E-10 | 8.14E-09 |
| PTGES3 | 6.13E+00 | 9.04E-10 | 8.30E-09 |
| TBP | 6.13E+00 | 9.05E-10 | 8.31E-09 |
| KLK2 | 6.12E+00 | 9.20E-10 | 8.43E-09 |
| CABLES2 | 6.12E+00 | 9.30E-10 | 8.53E-09 |
| HAVCR1 | 6.12E+00 | 9.31E-10 | 8.53E-09 |
| RCC1 | 6.12E+00 | 9.49E-10 | 8.69E-09 |
| SLC1A6 | 6.12E+00 | 9.63E-10 | 8.81E-09 |
| CCHCR1 | 6.12E+00 | 9.64E-10 | 8.82E-09 |
| DDA1 | 6.12E+00 | 9.68E-10 | 8.85E-09 |
| PHGDH | 6.11E+00 | 9.70E-10 | 8.86E-09 |
| TRPA1 | 6.11E+00 | 9.81E-10 | 8.97E-09 |
| GMIP | 6.11E+00 | 9.83E-10 | 8.98E-09 |
| ZNF485 | 6.11E+00 | 9.85E-10 | 8.99E-09 |
| RHBDD3 | 6.11E+00 | 9.88E-10 | 9.01E-09 |
| POP7 | 6.11E+00 | 9.95E-10 | 9.07E-09 |
| EIF5A | 6.11E+00 | 1.00E-09 | 9.13E-09 |
| RP9P | 6.11E+00 | 1.01E-09 | 9.16E-09 |
| FMNL1 | 6.11E+00 | 1.01E-09 | 9.17E-09 |
| WBP11 | 6.11E+00 | 1.02E-09 | 9.32E-09 |
| CSAG1 | 6.11E+00 | 1.03E-09 | 9.33E-09 |
| RTCD1 | 6.10E+00 | 1.04E-09 | 9.47E-09 |
| SYNJ2 | 6.10E+00 | 1.04E-09 | 9.47E-09 |
| DOCK6 | 6.10E+00 | 1.06E-09 | 9.62E-09 |
| PVR | 6.10E+00 | 1.06E-09 | 9.62E-09 |
| DYNLL1 | 6.10E+00 | 1.08E-09 | 9.79E-09 |
| EXTL3 | 6.09E+00 | 1.11E-09 | 1.01E-08 |
| KIF21A | 6.09E+00 | 1.14E-09 | 1.03E-08 |
| C17orf104 | 6.09E+00 | 1.16E-09 | 1.05E-08 |
| NDST4 | 6.08E+00 | 1.18E-09 | 1.07E-08 |
| PTOV1 | 6.08E+00 | 1.18E-09 | 1.07E-08 |
| L1CAM | 6.08E+00 | 1.19E-09 | 1.08E-08 |
| UBA2 | 6.08E+00 | 1.20E-09 | 1.09E-08 |
| KIF21B | 6.08E+00 | 1.22E-09 | 1.10E-08 |
| CYP24A1 | 6.08E+00 | 1.22E-09 | 1.10E-08 |
| CCDC58 | 6.08E+00 | 1.23E-09 | 1.11E-08 |
| MSC | 6.07E+00 | 1.26E-09 | 1.13E-08 |
| PI4KB | 6.07E+00 | 1.27E-09 | 1.15E-08 |
| IL11 | 6.07E+00 | 1.29E-09 | 1.16E-08 |
| GAPDHS | 6.07E+00 | 1.29E-09 | 1.16E-08 |
| C1orf182 | 6.07E+00 | 1.31E-09 | 1.18E-08 |

| CPLX2 | 6.07E+00 | 1.31E-09 | 1.18E-08 |
| --- | --- | --- | --- |
| MLLT11 | 6.07E+00 | 1.32E-09 | 1.19E-08 |
| ARIH2 | 6.07E+00 | 1.32E-09 | 1.19E-08 |
| NUDC | 6.06E+00 | 1.33E-09 | 1.20E-08 |
| GLA | 6.06E+00 | 1.33E-09 | 1.20E-08 |
| RRP15 | 6.06E+00 | 1.34E-09 | 1.21E-08 |
| C9orf40 | 6.06E+00 | 1.36E-09 | 1.22E-08 |
| CCT8 | 6.06E+00 | 1.36E-09 | 1.22E-08 |
| KLC3 | 6.06E+00 | 1.38E-09 | 1.24E-08 |
| SLC22A20 | 6.05E+00 | 1.44E-09 | 1.29E-08 |
| DENND1A | 6.05E+00 | 1.44E-09 | 1.30E-08 |
| ERCC3 | 6.05E+00 | 1.48E-09 | 1.32E-08 |
| HOXC13 | 6.04E+00 | 1.51E-09 | 1.36E-08 |
| CDK11A | 6.04E+00 | 1.52E-09 | 1.36E-08 |
| LOC652276 | 6.04E+00 | 1.52E-09 | 1.36E-08 |
| ITPA | 6.04E+00 | 1.52E-09 | 1.36E-08 |
| UBE2CBP | 6.04E+00 | 1.53E-09 | 1.37E-08 |
| TP53TG3B | 6.04E+00 | 1.54E-09 | 1.38E-08 |
| PPP1R16A | 6.04E+00 | 1.59E-09 | 1.42E-08 |
| KDM2A | 6.03E+00 | 1.60E-09 | 1.43E-08 |
| C16orf57 | 6.03E+00 | 1.62E-09 | 1.45E-08 |
| CPNE1 | 6.03E+00 | 1.63E-09 | 1.46E-08 |
| PRLHR | 6.03E+00 | 1.64E-09 | 1.46E-08 |
| KDM4B | 6.03E+00 | 1.69E-09 | 1.51E-08 |
| NOL12 | 6.02E+00 | 1.71E-09 | 1.52E-08 |
| GALNS | 6.02E+00 | 1.73E-09 | 1.54E-08 |
| REPS1 | 6.02E+00 | 1.75E-09 | 1.56E-08 |
| RIMS2 | 6.02E+00 | 1.78E-09 | 1.58E-08 |
| SRRM5 | 6.02E+00 | 1.79E-09 | 1.59E-08 |
| FAM168B | 6.02E+00 | 1.79E-09 | 1.59E-08 |
| ORAOV1 | 6.02E+00 | 1.79E-09 | 1.60E-08 |
| GAGE8 | 6.01E+00 | 1.83E-09 | 1.63E-08 |
| C4orf46 | 6.01E+00 | 1.84E-09 | 1.63E-08 |
| SLC15A4 | 6.01E+00 | 1.84E-09 | 1.63E-08 |
| CLSTN3 | 6.01E+00 | 1.85E-09 | 1.64E-08 |
| KIAA1731 | 6.01E+00 | 1.86E-09 | 1.65E-08 |
| HAPLN3 | 6.01E+00 | 1.86E-09 | 1.65E-08 |
| FAM101A | 6.01E+00 | 1.86E-09 | 1.65E-08 |
| SEH1L | 6.01E+00 | 1.87E-09 | 1.66E-08 |
| RIF1 | 6.01E+00 | 1.89E-09 | 1.67E-08 |
| HARS2 | 6.01E+00 | 1.91E-09 | 1.69E-08 |
| PCNT | 6.01E+00 | 1.91E-09 | 1.69E-08 |
| CTHRC1 | 6.00E+00 | 1.94E-09 | 1.71E-08 |
| HDLBP | 6.00E+00 | 1.97E-09 | 1.74E-08 |
| MEST | 6.00E+00 | 2.01E-09 | 1.78E-08 |
| KIAA0284 | 6.00E+00 | 2.04E-09 | 1.80E-08 |
| RNF157 | 5.99E+00 | 2.06E-09 | 1.82E-08 |
| AK3L1 | 5.99E+00 | 2.08E-09 | 1.84E-08 |
| SP2 | 5.99E+00 | 2.11E-09 | 1.86E-08 |
| STK32C | 5.99E+00 | 2.14E-09 | 1.89E-08 |
| OR1F1 | 5.99E+00 | 2.15E-09 | 1.90E-08 |
| C9orf129 | 5.98E+00 | 2.17E-09 | 1.91E-08 |
| NUDT15 | 5.98E+00 | 2.17E-09 | 1.91E-08 |
| NKX1-2 | 5.98E+00 | 2.18E-09 | 1.92E-08 |
| SP9 | 5.98E+00 | 2.19E-09 | 1.93E-08 |
| VPS37B | 5.98E+00 | 2.24E-09 | 1.97E-08 |
| CREG2 | 5.98E+00 | 2.28E-09 | 2.00E-08 |
| DLK2 | 5.97E+00 | 2.31E-09 | 2.03E-08 |

| HSN2 | 5.97E+00 | 2.34E-09 | 2.05E-08 |
| --- | --- | --- | --- |
| RPL7L1 | 5.97E+00 | 2.35E-09 | 2.06E-08 |
| POLR3G | 5.97E+00 | 2.36E-09 | 2.07E-08 |
| CN5H6.4 | 5.97E+00 | 2.36E-09 | 2.07E-08 |
| C10orf137 | 5.97E+00 | 2.39E-09 | 2.10E-08 |
| LOC399744 | 5.97E+00 | 2.39E-09 | 2.10E-08 |
| HIST1H1B | 5.97E+00 | 2.40E-09 | 2.10E-08 |
| VPS33B | 5.97E+00 | 2.40E-09 | 2.10E-08 |
| ST14 | 5.97E+00 | 2.41E-09 | 2.11E-08 |
| DHX8 | 5.97E+00 | 2.42E-09 | 2.11E-08 |
| RGS20 | 5.97E+00 | 2.44E-09 | 2.13E-08 |
| CARD9 | 5.97E+00 | 2.45E-09 | 2.14E-08 |
| RPL13P5 | 5.96E+00 | 2.46E-09 | 2.14E-08 |
| CPSF3 | 5.96E+00 | 2.49E-09 | 2.17E-08 |
| C1orf35 | 5.96E+00 | 2.55E-09 | 2.23E-08 |
| MFSD9 | 5.96E+00 | 2.55E-09 | 2.23E-08 |
| PHF21A | 5.95E+00 | 2.64E-09 | 2.30E-08 |
| C21orf70 | 5.95E+00 | 2.67E-09 | 2.33E-08 |
| MGC12982 | 5.94E+00 | 2.78E-09 | 2.42E-08 |
| CIT | 5.94E+00 | 2.80E-09 | 2.44E-08 |
| BAI2 | 5.94E+00 | 2.81E-09 | 2.45E-08 |
| CCDC88A | 5.94E+00 | 2.87E-09 | 2.49E-08 |
| INSM1 | 5.94E+00 | 2.87E-09 | 2.50E-08 |
| C8orf77 | 5.94E+00 | 2.88E-09 | 2.50E-08 |
| RFX6 | 5.94E+00 | 2.91E-09 | 2.53E-08 |
| FIGNL1 | 5.93E+00 | 3.00E-09 | 2.60E-08 |
| FANCC | 5.93E+00 | 3.02E-09 | 2.62E-08 |
| AP2M1 | 5.93E+00 | 3.02E-09 | 2.62E-08 |
| EME2 | 5.93E+00 | 3.10E-09 | 2.69E-08 |
| ZNF212 | 5.93E+00 | 3.10E-09 | 2.69E-08 |
| KRT8 | 5.93E+00 | 3.12E-09 | 2.70E-08 |
| GEN1 | 5.92E+00 | 3.14E-09 | 2.72E-08 |
| GIP | 5.92E+00 | 3.14E-09 | 2.72E-08 |
| PTP4A3 | 5.92E+00 | 3.16E-09 | 2.73E-08 |
| TCTN2 | 5.92E+00 | 3.17E-09 | 2.74E-08 |
| VAX1 | 5.92E+00 | 3.18E-09 | 2.75E-08 |
| C17orf93 | 5.92E+00 | 3.19E-09 | 2.76E-08 |
| TRA2B | 5.92E+00 | 3.26E-09 | 2.82E-08 |
| CACNG7 | 5.92E+00 | 3.28E-09 | 2.84E-08 |
| DDX41 | 5.92E+00 | 3.29E-09 | 2.84E-08 |
| TIMM13 | 5.92E+00 | 3.31E-09 | 2.85E-08 |
| FLJ36000 | 5.91E+00 | 3.39E-09 | 2.93E-08 |
| PKD1 | 5.91E+00 | 3.42E-09 | 2.95E-08 |
| KIR2DL4 | 5.91E+00 | 3.43E-09 | 2.96E-08 |
| NEB | 5.91E+00 | 3.45E-09 | 2.97E-08 |
| SERBP1 | 5.91E+00 | 3.45E-09 | 2.97E-08 |
| IL1R2 | 5.91E+00 | 3.47E-09 | 2.98E-08 |
| TIMM50 | 5.91E+00 | 3.49E-09 | 3.00E-08 |
| SNCB | 5.91E+00 | 3.50E-09 | 3.01E-08 |
| UNC13A | 5.90E+00 | 3.56E-09 | 3.06E-08 |
| HOXB13 | 5.90E+00 | 3.56E-09 | 3.06E-08 |
| HIST1H3D | 5.90E+00 | 3.59E-09 | 3.08E-08 |
| MDN1 | 5.90E+00 | 3.59E-09 | 3.08E-08 |
| TCL6 | 5.90E+00 | 3.59E-09 | 3.08E-08 |
| TCF20 | 5.90E+00 | 3.64E-09 | 3.12E-08 |
| FAM110A | 5.90E+00 | 3.65E-09 | 3.13E-08 |
| GON4L | 5.90E+00 | 3.68E-09 | 3.15E-08 |
| GABRG2 | 5.90E+00 | 3.68E-09 | 3.16E-08 |

| RGS19 | 5.90E+00 | 3.69E-09 | 3.16E-08 |
| --- | --- | --- | --- |
| RPP25 | 5.90E+00 | 3.74E-09 | 3.20E-08 |
| COX7B2 | 5.89E+00 | 3.79E-09 | 3.24E-08 |
| TH | 5.89E+00 | 3.83E-09 | 3.28E-08 |
| LOC222699 | 5.89E+00 | 3.84E-09 | 3.28E-08 |
| SHC1 | 5.89E+00 | 3.90E-09 | 3.33E-08 |
| UNC13D | 5.89E+00 | 3.91E-09 | 3.34E-08 |
| TAS1R3 | 5.89E+00 | 3.95E-09 | 3.37E-08 |
| KCNMB3 | 5.89E+00 | 3.96E-09 | 3.38E-08 |
| EPHA8 | 5.89E+00 | 3.99E-09 | 3.40E-08 |
| CC2D1B | 5.88E+00 | 4.08E-09 | 3.48E-08 |
| NR2C2AP | 5.88E+00 | 4.13E-09 | 3.52E-08 |
| TDP1 | 5.88E+00 | 4.17E-09 | 3.56E-08 |
| LOC116437 | 5.88E+00 | 4.21E-09 | 3.58E-08 |
| ZSCAN5A | 5.88E+00 | 4.21E-09 | 3.58E-08 |
| STT3A | 5.88E+00 | 4.22E-09 | 3.59E-08 |
| HIF1AN | 5.88E+00 | 4.24E-09 | 3.60E-08 |
| HK2 | 5.87E+00 | 4.30E-09 | 3.66E-08 |
| PPP1R12A | 5.87E+00 | 4.31E-09 | 3.66E-08 |
| TBCB | 5.87E+00 | 4.34E-09 | 3.68E-08 |
| PLEKHA8 | 5.87E+00 | 4.35E-09 | 3.69E-08 |
| MRPL9 | 5.87E+00 | 4.35E-09 | 3.70E-08 |
| NOMO1 | 5.87E+00 | 4.48E-09 | 3.80E-08 |
| SLC39A6 | 5.86E+00 | 4.52E-09 | 3.83E-08 |
| HOXD12 | 5.86E+00 | 4.54E-09 | 3.85E-08 |
| IP6K1 | 5.86E+00 | 4.59E-09 | 3.89E-08 |
| GPATCH1 | 5.86E+00 | 4.60E-09 | 3.89E-08 |
| SMCR7L | 5.86E+00 | 4.60E-09 | 3.89E-08 |
| CSRNP2 | 5.86E+00 | 4.60E-09 | 3.89E-08 |
| RFX1 | 5.86E+00 | 4.66E-09 | 3.94E-08 |
| HDAC4 | 5.86E+00 | 4.69E-09 | 3.96E-08 |
| UBE2N | 5.86E+00 | 4.69E-09 | 3.96E-08 |
| ADAM19 | 5.86E+00 | 4.70E-09 | 3.97E-08 |
| TMEM5 | 5.85E+00 | 4.85E-09 | 4.09E-08 |
| LOC389791 | 5.85E+00 | 4.89E-09 | 4.13E-08 |
| DCLRE1B | 5.85E+00 | 4.92E-09 | 4.15E-08 |
| HRAS | 5.85E+00 | 4.93E-09 | 4.16E-08 |
| LAMC1 | 5.85E+00 | 4.94E-09 | 4.17E-08 |
| OTUB1 | 5.85E+00 | 4.97E-09 | 4.19E-08 |
| UNKL | 5.85E+00 | 4.97E-09 | 4.19E-08 |
| TRAF3 | 5.85E+00 | 4.98E-09 | 4.19E-08 |
| GPR115 | 5.85E+00 | 5.00E-09 | 4.20E-08 |
| CHGA | 5.85E+00 | 5.06E-09 | 4.25E-08 |
| NCAPH2 | 5.84E+00 | 5.13E-09 | 4.31E-08 |
| MAGEC2 | 5.84E+00 | 5.23E-09 | 4.39E-08 |
| CT45A3 | 5.84E+00 | 5.23E-09 | 4.39E-08 |
| PPT2 | 5.84E+00 | 5.31E-09 | 4.46E-08 |
| ESX1 | 5.84E+00 | 5.33E-09 | 4.48E-08 |
| SNHG7 | 5.84E+00 | 5.34E-09 | 4.48E-08 |
| TUFT1 | 5.84E+00 | 5.35E-09 | 4.48E-08 |
| HES6 | 5.84E+00 | 5.40E-09 | 4.52E-08 |
| SMC4 | 5.83E+00 | 5.41E-09 | 4.53E-08 |
| PSMD12 | 5.83E+00 | 5.42E-09 | 4.54E-08 |
| DUS1L | 5.83E+00 | 5.47E-09 | 4.58E-08 |
| PLOD1 | 5.83E+00 | 5.52E-09 | 4.62E-08 |
| ARHGEF11 | 5.83E+00 | 5.59E-09 | 4.67E-08 |
| ING5 | 5.83E+00 | 5.59E-09 | 4.67E-08 |
| KCNV1 | 5.83E+00 | 5.63E-09 | 4.71E-08 |

| SLC27A4 | 5.83E+00 | 5.65E-09 | 4.72E-08 |
| --- | --- | --- | --- |
| PRKRIP1 | 5.82E+00 | 5.73E-09 | 4.78E-08 |
| SLC24A6 | 5.82E+00 | 5.77E-09 | 4.82E-08 |
| KLHL31 | 5.82E+00 | 5.80E-09 | 4.84E-08 |
| TRIM6 | 5.82E+00 | 5.81E-09 | 4.84E-08 |
| PAQR9 | 5.82E+00 | 5.85E-09 | 4.88E-08 |
| EHD1 | 5.82E+00 | 5.87E-09 | 4.89E-08 |
| LOXL2 | 5.82E+00 | 5.87E-09 | 4.89E-08 |
| PTHLH | 5.82E+00 | 5.87E-09 | 4.89E-08 |
| NRD1 | 5.82E+00 | 5.96E-09 | 4.96E-08 |
| KLF14 | 5.82E+00 | 6.03E-09 | 5.02E-08 |
| GAS2L1 | 5.82E+00 | 6.04E-09 | 5.02E-08 |
| TMEM185B | 5.82E+00 | 6.05E-09 | 5.03E-08 |
| TACR3 | 5.82E+00 | 6.07E-09 | 5.04E-08 |
| HTR1D | 5.82E+00 | 6.08E-09 | 5.05E-08 |
| POLR2E | 5.81E+00 | 6.14E-09 | 5.10E-08 |
| RBM15 | 5.81E+00 | 6.25E-09 | 5.19E-08 |
| B4GALNT1 | 5.81E+00 | 6.27E-09 | 5.20E-08 |
| LRRC69 | 5.81E+00 | 6.28E-09 | 5.20E-08 |
| ILF2 | 5.81E+00 | 6.31E-09 | 5.23E-08 |
| TMEM65 | 5.81E+00 | 6.33E-09 | 5.24E-08 |
| AGBL5 | 5.81E+00 | 6.34E-09 | 5.25E-08 |
| CC2D1A | 5.81E+00 | 6.39E-09 | 5.29E-08 |
| UCP1 | 5.80E+00 | 6.52E-09 | 5.40E-08 |
| SSX6 | 5.80E+00 | 6.62E-09 | 5.48E-08 |
| COL5A2 | 5.80E+00 | 6.75E-09 | 5.58E-08 |
| CNIH2 | 5.80E+00 | 6.75E-09 | 5.58E-08 |
| STEAP1 | 5.80E+00 | 6.76E-09 | 5.58E-08 |
| EMR2 | 5.80E+00 | 6.78E-09 | 5.60E-08 |
| CCDC144NL | 5.80E+00 | 6.83E-09 | 5.63E-08 |
| KCTD10 | 5.79E+00 | 6.86E-09 | 5.66E-08 |
| RPL6 | 5.79E+00 | 6.92E-09 | 5.70E-08 |
| RBM12 | 5.79E+00 | 7.00E-09 | 5.77E-08 |
| NAGPA | 5.79E+00 | 7.04E-09 | 5.80E-08 |
| PITX3 | 5.79E+00 | 7.04E-09 | 5.80E-08 |
| DCTPP1 | 5.79E+00 | 7.06E-09 | 5.81E-08 |
| PDIA4 | 5.79E+00 | 7.08E-09 | 5.82E-08 |
| RAB42 | 5.79E+00 | 7.13E-09 | 5.87E-08 |
| DMRTC2 | 5.79E+00 | 7.19E-09 | 5.92E-08 |
| HCN2 | 5.79E+00 | 7.26E-09 | 5.97E-08 |
| MAGEB2 | 5.78E+00 | 7.27E-09 | 5.98E-08 |
| MSL1 | 5.78E+00 | 7.28E-09 | 5.98E-08 |
| C12orf10 | 5.78E+00 | 7.30E-09 | 5.99E-08 |
| SPRED3 | 5.78E+00 | 7.32E-09 | 6.01E-08 |
| PITPNM1 | 5.78E+00 | 7.34E-09 | 6.02E-08 |
| NCBP1 | 5.78E+00 | 7.34E-09 | 6.02E-08 |
| ARID1A | 5.78E+00 | 7.45E-09 | 6.11E-08 |
| RNF216L | 5.78E+00 | 7.45E-09 | 6.11E-08 |
| LRRC1 | 5.78E+00 | 7.48E-09 | 6.13E-08 |
| SCD | 5.78E+00 | 7.50E-09 | 6.14E-08 |
| HOXA10 | 5.78E+00 | 7.50E-09 | 6.14E-08 |
| SCAMP4 | 5.78E+00 | 7.54E-09 | 6.17E-08 |
| AMDHD2 | 5.78E+00 | 7.70E-09 | 6.29E-08 |
| PARP12 | 5.77E+00 | 8.11E-09 | 6.63E-08 |
| DCLK3 | 5.76E+00 | 8.26E-09 | 6.74E-08 |
| MYO18B | 5.76E+00 | 8.31E-09 | 6.79E-08 |
| BCCIP | 5.76E+00 | 8.34E-09 | 6.81E-08 |
| COX19 | 5.76E+00 | 8.42E-09 | 6.87E-08 |

| ACTN3 | 5.76E+00 | 8.57E-09 | 6.99E-08 |
| --- | --- | --- | --- |
| FSD1L | 5.76E+00 | 8.62E-09 | 7.02E-08 |
| CIB2 | 5.76E+00 | 8.62E-09 | 7.03E-08 |
| ELOVL4 | 5.76E+00 | 8.65E-09 | 7.04E-08 |
| FAM75A3 | 5.75E+00 | 8.81E-09 | 7.18E-08 |
| SNORD116-4 | 5.75E+00 | 8.94E-09 | 7.27E-08 |
| GALNT2 | 5.75E+00 | 8.99E-09 | 7.31E-08 |
| NCOA3 | 5.74E+00 | 9.25E-09 | 7.52E-08 |
| GPN2 | 5.74E+00 | 9.30E-09 | 7.56E-08 |
| SAAL1 | 5.74E+00 | 9.36E-09 | 7.61E-08 |
| COCH | 5.74E+00 | 9.48E-09 | 7.70E-08 |
| NBPF6 | 5.74E+00 | 9.54E-09 | 7.74E-08 |
| ADAR | 5.74E+00 | 9.58E-09 | 7.77E-08 |
| HSPA1A | 5.74E+00 | 9.59E-09 | 7.78E-08 |
| EXOC3 | 5.74E+00 | 9.63E-09 | 7.81E-08 |
| C21orf58 | 5.74E+00 | 9.66E-09 | 7.83E-08 |
| NPW | 5.73E+00 | 9.79E-09 | 7.93E-08 |
| NAA38 | 5.73E+00 | 9.81E-09 | 7.94E-08 |
| HIST1H1D | 5.73E+00 | 9.82E-09 | 7.95E-08 |
| HNRNPAB | 5.73E+00 | 9.82E-09 | 7.95E-08 |
| NCS1 | 5.73E+00 | 1.00E-08 | 8.09E-08 |
| POP5 | 5.73E+00 | 1.00E-08 | 8.11E-08 |
| ZNF71 | 5.73E+00 | 1.01E-08 | 8.15E-08 |
| HOXC8 | 5.73E+00 | 1.02E-08 | 8.22E-08 |
| CHST1 | 5.73E+00 | 1.02E-08 | 8.22E-08 |
| RARS | 5.73E+00 | 1.02E-08 | 8.23E-08 |
| BRD3 | 5.73E+00 | 1.02E-08 | 8.24E-08 |
| LRRC14B | 5.73E+00 | 1.02E-08 | 8.25E-08 |
| ACTL6A | 5.73E+00 | 1.03E-08 | 8.26E-08 |
| TAP2 | 5.73E+00 | 1.03E-08 | 8.31E-08 |
| UGDH | 5.73E+00 | 1.03E-08 | 8.32E-08 |
| VAT1 | 5.73E+00 | 1.03E-08 | 8.32E-08 |
| MRPS12 | 5.73E+00 | 1.04E-08 | 8.33E-08 |
| PHB | 5.72E+00 | 1.07E-08 | 8.60E-08 |
| UNC45A | 5.72E+00 | 1.08E-08 | 8.67E-08 |
| MYO7A | 5.72E+00 | 1.08E-08 | 8.68E-08 |
| LOC727896 | 5.72E+00 | 1.10E-08 | 8.82E-08 |
| PSPC1 | 5.71E+00 | 1.11E-08 | 8.87E-08 |
| TFPT | 5.71E+00 | 1.11E-08 | 8.92E-08 |
| DPH3B | 5.71E+00 | 1.11E-08 | 8.94E-08 |
| SMS | 5.71E+00 | 1.13E-08 | 9.06E-08 |
| AHRR | 5.71E+00 | 1.13E-08 | 9.07E-08 |
| MRPL10 | 5.71E+00 | 1.14E-08 | 9.11E-08 |
| MMP14 | 5.70E+00 | 1.17E-08 | 9.38E-08 |
| SLC43A2 | 5.70E+00 | 1.17E-08 | 9.39E-08 |
| GRIPAP1 | 5.70E+00 | 1.18E-08 | 9.46E-08 |
| SRGAP1 | 5.70E+00 | 1.20E-08 | 9.61E-08 |
| DCAF8L2 | 5.70E+00 | 1.21E-08 | 9.70E-08 |
| C19orf22 | 5.70E+00 | 1.22E-08 | 9.77E-08 |
| TCIRG1 | 5.70E+00 | 1.22E-08 | 9.77E-08 |
| PCNA | 5.70E+00 | 1.22E-08 | 9.77E-08 |
| RPP40 | 5.70E+00 | 1.24E-08 | 9.88E-08 |
| UNC119 | 5.69E+00 | 1.24E-08 | 9.88E-08 |
| AQPEP | 5.69E+00 | 1.25E-08 | 9.98E-08 |
| SRP68 | 5.69E+00 | 1.25E-08 | 9.99E-08 |
| CCDC88C | 5.69E+00 | 1.26E-08 | 1.01E-07 |
| CLGN | 5.69E+00 | 1.29E-08 | 1.03E-07 |
| PFDN2 | 5.68E+00 | 1.33E-08 | 1.06E-07 |

| FICD |  | 5.68E+00 | 1.34E-08 | 1.06E-07 |
| --- | --- | --- | --- | --- |
| SMUG1 |  | 5.68E+00 | 1.34E-08 | 1.06E-07 |
| RANBP3 |  | 5.68E+00 | 1.36E-08 | 1.08E-07 |
| ZNF367 |  | 5.68E+00 | 1.36E-08 | 1.08E-07 |
| FAM86C |  | 5.68E+00 | 1.38E-08 | 1.10E-07 |
| C8ORFK29 |  | 5.68E+00 | 1.39E-08 | 1.10E-07 |
| SF4 |  | 5.67E+00 | 1.40E-08 | 1.11E-07 |
| MVK |  | 5.67E+00 | 1.41E-08 | 1.12E-07 |
| MEPCE |  | 5.67E+00 | 1.42E-08 | 1.13E-07 |
| VHL |  | 5.67E+00 | 1.42E-08 | 1.13E-07 |
| HOXA1 |  | 5.67E+00 | 1.43E-08 | 1.14E-07 |
| LY6G5B |  | 5.67E+00 | 1.45E-08 | 1.15E-07 |
| CHMP1A |  | 5.66E+00 | 1.48E-08 | 1.17E-07 |
| WDR81 |  | 5.66E+00 | 1.50E-08 | 1.19E-07 |
| HIST2H3D |  | 5.65E+00 | 1.58E-08 | 1.25E-07 |
| MFSD5 |  | 5.65E+00 | 1.58E-08 | 1.25E-07 |
| SLC39A1 |  | 5.65E+00 | 1.58E-08 | 1.25E-07 |
| TTC26 |  | 5.65E+00 | 1.58E-08 | 1.25E-07 |
| NOTCH3 |  | 5.65E+00 | 1.60E-08 | 1.27E-07 |
| IGDCC4 |  | 5.65E+00 | 1.61E-08 | 1.27E-07 |
| PDCD2 |  | 5.64E+00 | 1.67E-08 | 1.32E-07 |
| MED13L |  | 5.64E+00 | 1.68E-08 | 1.32E-07 |
| HOXD10 |  | 5.64E+00 | 1.69E-08 | 1.33E-07 |
| MRPS30 |  | 5.64E+00 | 1.71E-08 | 1.35E-07 |
| C19orf23 |  | 5.64E+00 | 1.73E-08 | 1.36E-07 |
| GH2 |  | 5.64E+00 | 1.74E-08 | 1.37E-07 |
| LARP4B |  | 5.63E+00 | 1.76E-08 | 1.39E-07 |
| KLHDC5 |  | 5.63E+00 | 1.78E-08 | 1.40E-07 |
| DUSP12 |  | 5.63E+00 | 1.81E-08 | 1.42E-07 |
| GAP43 |  | 5.63E+00 | 1.81E-08 | 1.43E-07 |
| GABRQ |  | 5.63E+00 | 1.81E-08 | 1.43E-07 |
| ZNF7 |  | 5.63E+00 | 1.82E-08 | 1.43E-07 |
| DKFZp686A1627 |  | 5.63E+00 | 1.83E-08 | 1.44E-07 |
| LRFN1 |  | 5.63E+00 | 1.84E-08 | 1.45E-07 |
| C9orf110 |  | 5.63E+00 | 1.85E-08 | 1.45E-07 |
| CSPP1 |  | 5.62E+00 | 1.86E-08 | 1.46E-07 |
| MCHR2 |  | 5.62E+00 | 1.86E-08 | 1.46E-07 |
| APBA3 |  | 5.62E+00 | 1.87E-08 | 1.47E-07 |
| SPERT |  | 5.62E+00 | 1.89E-08 | 1.49E-07 |
| MAP3K4 |  | 5.62E+00 | 1.90E-08 | 1.49E-07 |
| ZBTB9 |  | 5.62E+00 | 1.91E-08 | 1.50E-07 |
| CDC27 |  | 5.62E+00 | 1.91E-08 | 1.50E-07 |
| ACTR3B |  | 5.62E+00 | 1.96E-08 | 1.54E-07 |
| MAGEA8 |  | 5.62E+00 | 1.97E-08 | 1.54E-07 |
| MAPK12 |  | 5.61E+00 | 1.98E-08 | 1.55E-07 |
|  | 14-Sep | 5.61E+00 | 1.99E-08 | 1.56E-07 |
| C12orf39 |  | 5.61E+00 | 2.00E-08 | 1.57E-07 |
| KIFC2 |  | 5.61E+00 | 2.00E-08 | 1.57E-07 |
| HSF2BP |  | 5.61E+00 | 2.02E-08 | 1.58E-07 |
| CABYR |  | 5.61E+00 | 2.04E-08 | 1.60E-07 |
| SIPA1 |  | 5.61E+00 | 2.08E-08 | 1.62E-07 |
| DCTN1 |  | 5.60E+00 | 2.10E-08 | 1.64E-07 |
| SAGE1 |  | 5.60E+00 | 2.11E-08 | 1.65E-07 |
| TMED2 |  | 5.60E+00 | 2.11E-08 | 1.65E-07 |
| STRAP |  | 5.60E+00 | 2.12E-08 | 1.65E-07 |
| NADK |  | 5.60E+00 | 2.12E-08 | 1.65E-07 |
| OPN1SW |  | 5.60E+00 | 2.13E-08 | 1.66E-07 |
| C1orf61 |  | 5.60E+00 | 2.14E-08 | 1.67E-07 |

| METAP2 | 5.60E+00 | 2.15E-08 | 1.67E-07 |
| --- | --- | --- | --- |
| SQLE | 5.60E+00 | 2.15E-08 | 1.67E-07 |
| DCAF4L1 | 5.60E+00 | 2.18E-08 | 1.69E-07 |
| PRDM10 | 5.60E+00 | 2.20E-08 | 1.71E-07 |
| MTMR2 | 5.60E+00 | 2.20E-08 | 1.71E-07 |
| HSPH1 | 5.59E+00 | 2.22E-08 | 1.73E-07 |
| IQCB1 | 5.59E+00 | 2.26E-08 | 1.75E-07 |
| HOXC9 | 5.59E+00 | 2.27E-08 | 1.76E-07 |
| SLC5A10 | 5.59E+00 | 2.29E-08 | 1.78E-07 |
| ALDH16A1 | 5.59E+00 | 2.29E-08 | 1.78E-07 |
| CORO6 | 5.59E+00 | 2.30E-08 | 1.78E-07 |
| PLEKHG5 | 5.59E+00 | 2.31E-08 | 1.79E-07 |
| LIMD2 | 5.59E+00 | 2.32E-08 | 1.80E-07 |
| MAP7D1 | 5.58E+00 | 2.35E-08 | 1.82E-07 |
| HES4 | 5.58E+00 | 2.38E-08 | 1.84E-07 |
| CLDN19 | 5.58E+00 | 2.41E-08 | 1.87E-07 |
| DSP | 5.58E+00 | 2.44E-08 | 1.89E-07 |
| VCX3A | 5.58E+00 | 2.47E-08 | 1.91E-07 |
| BCL3 | 5.58E+00 | 2.48E-08 | 1.92E-07 |
| TMEM184A | 5.57E+00 | 2.49E-08 | 1.93E-07 |
| CACYBP | 5.57E+00 | 2.51E-08 | 1.94E-07 |
| TRERF1 | 5.57E+00 | 2.54E-08 | 1.96E-07 |
| VCX | 5.57E+00 | 2.54E-08 | 1.96E-07 |
| C4orf48 | 5.57E+00 | 2.62E-08 | 2.02E-07 |
| LOC80154 | 5.57E+00 | 2.62E-08 | 2.02E-07 |
| TULP3 | 5.56E+00 | 2.69E-08 | 2.08E-07 |
| PRIM2 | 5.56E+00 | 2.76E-08 | 2.12E-07 |
| AGPS | 5.56E+00 | 2.78E-08 | 2.14E-07 |
| USP14 | 5.55E+00 | 2.78E-08 | 2.14E-07 |
| ALX3 | 5.55E+00 | 2.80E-08 | 2.15E-07 |
| SLC35C2 | 5.55E+00 | 2.82E-08 | 2.17E-07 |
| TRIM15 | 5.55E+00 | 2.84E-08 | 2.18E-07 |
| GAL3ST3 | 5.55E+00 | 2.86E-08 | 2.20E-07 |
| SLC2A5 | 5.55E+00 | 2.88E-08 | 2.21E-07 |
| B4GALNT4 | 5.55E+00 | 2.89E-08 | 2.22E-07 |
| BAGE2 | 5.55E+00 | 2.89E-08 | 2.22E-07 |
| PMS2L3 | 5.55E+00 | 2.92E-08 | 2.24E-07 |
| C1orf113 | 5.55E+00 | 2.94E-08 | 2.25E-07 |
| C14orf23 | 5.54E+00 | 2.95E-08 | 2.27E-07 |
| MAP6D1 | 5.54E+00 | 2.96E-08 | 2.27E-07 |
| FOXRED2 | 5.54E+00 | 2.98E-08 | 2.28E-07 |
| CSNK1G1 | 5.54E+00 | 2.99E-08 | 2.29E-07 |
| NBN | 5.54E+00 | 3.00E-08 | 2.30E-07 |
| EED | 5.54E+00 | 3.01E-08 | 2.30E-07 |
| ERMP1 | 5.54E+00 | 3.01E-08 | 2.30E-07 |
| LOC642852 | 5.54E+00 | 3.03E-08 | 2.31E-07 |
| LOC100286793 | 5.54E+00 | 3.03E-08 | 2.32E-07 |
| FUBP3 | 5.54E+00 | 3.04E-08 | 2.33E-07 |
| GPKOW | 5.54E+00 | 3.05E-08 | 2.33E-07 |
| GOSR2 | 5.54E+00 | 3.06E-08 | 2.33E-07 |
| LAGE3 | 5.54E+00 | 3.09E-08 | 2.36E-07 |
| C2orf18 | 5.54E+00 | 3.12E-08 | 2.38E-07 |
| C6orf218 | 5.54E+00 | 3.12E-08 | 2.38E-07 |
| FAM90A1 | 5.53E+00 | 3.12E-08 | 2.38E-07 |
| OLFML2B | 5.53E+00 | 3.13E-08 | 2.38E-07 |
| ADAT2 | 5.53E+00 | 3.16E-08 | 2.41E-07 |
| ATXN7L3B | 5.53E+00 | 3.17E-08 | 2.42E-07 |
| PI4KA | 5.53E+00 | 3.21E-08 | 2.44E-07 |

| ATP5SL | 5.53E+00 | 3.23E-08 | 2.45E-07 |
| --- | --- | --- | --- |
| RNF183 | 5.53E+00 | 3.23E-08 | 2.46E-07 |
| EPPK1 | 5.53E+00 | 3.24E-08 | 2.46E-07 |
| FAM132A | 5.53E+00 | 3.28E-08 | 2.49E-07 |
| RRM1 | 5.53E+00 | 3.29E-08 | 2.50E-07 |
| TUBB4 | 5.53E+00 | 3.29E-08 | 2.50E-07 |
| RNMT | 5.52E+00 | 3.31E-08 | 2.51E-07 |
| HNRNPH3 | 5.52E+00 | 3.31E-08 | 2.51E-07 |
| KIF26B | 5.52E+00 | 3.33E-08 | 2.53E-07 |
| RNF123 | 5.52E+00 | 3.40E-08 | 2.58E-07 |
| CEP135 | 5.52E+00 | 3.46E-08 | 2.63E-07 |
| KC6 | 5.52E+00 | 3.47E-08 | 2.63E-07 |
| MSMP | 5.52E+00 | 3.48E-08 | 2.63E-07 |
| THOC6 | 5.52E+00 | 3.48E-08 | 2.64E-07 |
| MAGEF1 | 5.52E+00 | 3.49E-08 | 2.64E-07 |
| SPHK2 | 5.51E+00 | 3.50E-08 | 2.65E-07 |
| MYH16 | 5.51E+00 | 3.54E-08 | 2.68E-07 |
| IGDCC3 | 5.51E+00 | 3.56E-08 | 2.69E-07 |
| MAEA | 5.51E+00 | 3.58E-08 | 2.71E-07 |
| WDR74 | 5.51E+00 | 3.59E-08 | 2.71E-07 |
| FGD1 | 5.51E+00 | 3.67E-08 | 2.77E-07 |
| POLR2D | 5.51E+00 | 3.68E-08 | 2.78E-07 |
| ZNF513 | 5.50E+00 | 3.71E-08 | 2.80E-07 |
| MCF2L2 | 5.50E+00 | 3.74E-08 | 2.83E-07 |
| B4GALT4 | 5.50E+00 | 3.81E-08 | 2.88E-07 |
| SLC2A6 | 5.50E+00 | 3.85E-08 | 2.90E-07 |
| NSF | 5.49E+00 | 3.92E-08 | 2.95E-07 |
| C2orf27A | 5.49E+00 | 3.94E-08 | 2.97E-07 |
| PRR14 | 5.49E+00 | 3.95E-08 | 2.97E-07 |
| CNTNAP1 | 5.49E+00 | 3.97E-08 | 2.99E-07 |
| C16orf68 | 5.49E+00 | 4.11E-08 | 3.09E-07 |
| SBK2 | 5.48E+00 | 4.26E-08 | 3.20E-07 |
| PLEKHH1 | 5.48E+00 | 4.26E-08 | 3.21E-07 |
| EFHD2 | 5.48E+00 | 4.27E-08 | 3.21E-07 |
| DLX1 | 5.48E+00 | 4.31E-08 | 3.24E-07 |
| KDM6B | 5.48E+00 | 4.35E-08 | 3.27E-07 |
| LOC440173 | 5.47E+00 | 4.42E-08 | 3.32E-07 |
| SUZ12P | 5.47E+00 | 4.47E-08 | 3.36E-07 |
| HPS4 | 5.47E+00 | 4.49E-08 | 3.37E-07 |
| STRA6 | 5.47E+00 | 4.49E-08 | 3.37E-07 |
| CCNK | 5.47E+00 | 4.50E-08 | 3.37E-07 |
| EXOC2 | 5.47E+00 | 4.53E-08 | 3.39E-07 |
| UBE2J2 | 5.47E+00 | 4.55E-08 | 3.40E-07 |
| KIAA0947 | 5.47E+00 | 4.57E-08 | 3.42E-07 |
| DPF2 | 5.46E+00 | 4.67E-08 | 3.50E-07 |
| NPAS1 | 5.46E+00 | 4.68E-08 | 3.50E-07 |
| H1FX | 5.46E+00 | 4.69E-08 | 3.51E-07 |
| GPATCH8 | 5.46E+00 | 4.80E-08 | 3.59E-07 |
| SPANXB2 | 5.46E+00 | 4.87E-08 | 3.64E-07 |
| TTYH1 | 5.46E+00 | 4.87E-08 | 3.64E-07 |
| NKAIN4 | 5.45E+00 | 4.93E-08 | 3.68E-07 |
| RSRC2 | 5.45E+00 | 4.93E-08 | 3.68E-07 |
| C14orf21 | 5.45E+00 | 4.93E-08 | 3.68E-07 |
| LOC100134713 | 5.45E+00 | 4.94E-08 | 3.69E-07 |
| PPIL1 | 5.45E+00 | 4.97E-08 | 3.71E-07 |
| CDK6 | 5.45E+00 | 4.99E-08 | 3.72E-07 |
| H19 | 5.45E+00 | 5.00E-08 | 3.73E-07 |
| PFDN4 | 5.45E+00 | 5.01E-08 | 3.73E-07 |

| FAM157B | 5.45E+00 | 5.02E-08 | 3.74E-07 |
| --- | --- | --- | --- |
| GPR107 | 5.45E+00 | 5.03E-08 | 3.74E-07 |
| TUBB2A | 5.45E+00 | 5.03E-08 | 3.74E-07 |
| NME6 | 5.45E+00 | 5.04E-08 | 3.75E-07 |
| CGREF1 | 5.45E+00 | 5.05E-08 | 3.76E-07 |
| TMEM39B | 5.45E+00 | 5.10E-08 | 3.79E-07 |
| CASKIN2 | 5.45E+00 | 5.16E-08 | 3.84E-07 |
| ZP3 | 5.45E+00 | 5.19E-08 | 3.85E-07 |
| FAM57B | 5.44E+00 | 5.21E-08 | 3.87E-07 |
| PDE6A | 5.44E+00 | 5.21E-08 | 3.87E-07 |
| LAMB1 | 5.44E+00 | 5.30E-08 | 3.93E-07 |
| NCRNA00189 | 5.44E+00 | 5.30E-08 | 3.93E-07 |
| ZIC5 | 5.44E+00 | 5.39E-08 | 3.99E-07 |
| SRPK2 | 5.44E+00 | 5.44E-08 | 4.03E-07 |
| GABRA2 | 5.44E+00 | 5.46E-08 | 4.04E-07 |
| FASTK | 5.43E+00 | 5.54E-08 | 4.10E-07 |
| MED27 | 5.43E+00 | 5.56E-08 | 4.12E-07 |
| FGF5 | 5.43E+00 | 5.57E-08 | 4.12E-07 |
| CHD1L | 5.43E+00 | 5.69E-08 | 4.21E-07 |
| CDC34 | 5.43E+00 | 5.77E-08 | 4.27E-07 |
| MTF2 | 5.42E+00 | 5.88E-08 | 4.35E-07 |
| CYP26B1 | 5.42E+00 | 5.91E-08 | 4.37E-07 |
| CEP110 | 5.42E+00 | 5.94E-08 | 4.39E-07 |
| CHST12 | 5.42E+00 | 5.96E-08 | 4.40E-07 |
| MON1B | 5.42E+00 | 6.04E-08 | 4.45E-07 |
| HIST1H2BH | 5.42E+00 | 6.04E-08 | 4.46E-07 |
| C6orf147 | 5.42E+00 | 6.10E-08 | 4.50E-07 |
| MAGEA11 | 5.41E+00 | 6.15E-08 | 4.53E-07 |
| ZNF703 | 5.41E+00 | 6.16E-08 | 4.54E-07 |
| HSPB1 | 5.41E+00 | 6.19E-08 | 4.56E-07 |
| POGK | 5.41E+00 | 6.24E-08 | 4.59E-07 |
| GHRLOS | 5.41E+00 | 6.26E-08 | 4.60E-07 |
| VPRBP | 5.41E+00 | 6.27E-08 | 4.61E-07 |
| CIDECP | 5.41E+00 | 6.40E-08 | 4.71E-07 |
| CPSF3L | 5.41E+00 | 6.47E-08 | 4.76E-07 |
| ATRIP | 5.41E+00 | 6.47E-08 | 4.76E-07 |
| MYEOV | 5.41E+00 | 6.49E-08 | 4.77E-07 |
| DSG2 | 5.40E+00 | 6.50E-08 | 4.78E-07 |
| SP8 | 5.40E+00 | 6.60E-08 | 4.84E-07 |
| PAWR | 5.40E+00 | 6.63E-08 | 4.87E-07 |
| MIAT | 5.40E+00 | 6.65E-08 | 4.88E-07 |
| POLR2H | 5.40E+00 | 6.74E-08 | 4.94E-07 |
| TMC6 | 5.40E+00 | 6.76E-08 | 4.96E-07 |
| HOXA9 | 5.40E+00 | 6.78E-08 | 4.97E-07 |
| SOX4 | 5.39E+00 | 6.88E-08 | 5.04E-07 |
| POLD3 | 5.39E+00 | 6.92E-08 | 5.07E-07 |
| FAM47A | 5.39E+00 | 6.95E-08 | 5.09E-07 |
| GNL1 | 5.39E+00 | 6.96E-08 | 5.09E-07 |
| MAFK | 5.39E+00 | 7.07E-08 | 5.17E-07 |
| BEST3 | 5.39E+00 | 7.14E-08 | 5.22E-07 |
| COMTD1 | 5.39E+00 | 7.17E-08 | 5.24E-07 |
| GPRC5D | 5.39E+00 | 7.22E-08 | 5.28E-07 |
| ADNP2 | 5.39E+00 | 7.26E-08 | 5.30E-07 |
| NOMO2 | 5.38E+00 | 7.34E-08 | 5.36E-07 |
| DLX6 | 5.38E+00 | 7.38E-08 | 5.38E-07 |
| ALKBH4 | 5.38E+00 | 7.41E-08 | 5.40E-07 |
| COL1A1 | 5.38E+00 | 7.41E-08 | 5.40E-07 |
| MAGEC1 | 5.38E+00 | 7.43E-08 | 5.41E-07 |

| DCTN2 | 5.38E+00 | 7.44E-08 | 5.42E-07 |
| --- | --- | --- | --- |
| LOC100190939 | 5.38E+00 | 7.45E-08 | 5.43E-07 |
| NELF | 5.38E+00 | 7.50E-08 | 5.46E-07 |
| ACCN2 | 5.38E+00 | 7.55E-08 | 5.50E-07 |
| MEF2B | 5.38E+00 | 7.55E-08 | 5.50E-07 |
| OR56A3 | 5.38E+00 | 7.59E-08 | 5.52E-07 |
| GJD4 | 5.38E+00 | 7.64E-08 | 5.56E-07 |
| PARP10 | 5.37E+00 | 7.76E-08 | 5.64E-07 |
| HBE1 | 5.37E+00 | 7.78E-08 | 5.65E-07 |
| PHF12 | 5.37E+00 | 7.87E-08 | 5.71E-07 |
| LOC100130274 | 5.37E+00 | 7.89E-08 | 5.73E-07 |
| QSER1 | 5.37E+00 | 7.90E-08 | 5.73E-07 |
| KIAA1522 | 5.37E+00 | 7.96E-08 | 5.77E-07 |
| ZSCAN2 | 5.37E+00 | 7.96E-08 | 5.77E-07 |
| FGFR4 | 5.36E+00 | 8.16E-08 | 5.91E-07 |
| TAB1 | 5.36E+00 | 8.21E-08 | 5.95E-07 |
| TBCD | 5.36E+00 | 8.42E-08 | 6.10E-07 |
| PDZD7 | 5.36E+00 | 8.43E-08 | 6.10E-07 |
| SH3TC1 | 5.36E+00 | 8.44E-08 | 6.11E-07 |
| SPP1 | 5.36E+00 | 8.47E-08 | 6.13E-07 |
| HOXD9 | 5.36E+00 | 8.50E-08 | 6.15E-07 |
| METTL2B | 5.35E+00 | 8.65E-08 | 6.26E-07 |
| NPM3 | 5.35E+00 | 8.68E-08 | 6.27E-07 |
| FBXO21 | 5.35E+00 | 8.69E-08 | 6.28E-07 |
| HERC2 | 5.35E+00 | 8.76E-08 | 6.33E-07 |
| MT1H | 5.35E+00 | 8.81E-08 | 6.36E-07 |
| GTF2H4 | 5.35E+00 | 8.94E-08 | 6.45E-07 |
| FAM119A | 5.35E+00 | 9.00E-08 | 6.49E-07 |
| DHCR7 | 5.35E+00 | 9.03E-08 | 6.51E-07 |
| MIXL1 | 5.35E+00 | 9.05E-08 | 6.52E-07 |
| SGSH | 5.34E+00 | 9.07E-08 | 6.54E-07 |
| DNAH17 | 5.34E+00 | 9.09E-08 | 6.55E-07 |
| SNHG3-RCC1 | 5.34E+00 | 9.15E-08 | 6.59E-07 |
| LSM4 | 5.34E+00 | 9.17E-08 | 6.60E-07 |
| ZBTB32 | 5.34E+00 | 9.18E-08 | 6.60E-07 |
| TRAPPC9 | 5.34E+00 | 9.34E-08 | 6.72E-07 |
| TAF1C | 5.34E+00 | 9.41E-08 | 6.77E-07 |
| MTNR1B | 5.34E+00 | 9.54E-08 | 6.86E-07 |
| SPATA5 | 5.34E+00 | 9.55E-08 | 6.87E-07 |
| LOC220729 | 5.33E+00 | 9.64E-08 | 6.92E-07 |
| DYRK2 | 5.33E+00 | 9.67E-08 | 6.94E-07 |
| PSMC5 | 5.33E+00 | 9.72E-08 | 6.98E-07 |
| OSGIN2 | 5.33E+00 | 9.74E-08 | 6.98E-07 |
| PRPF40A | 5.33E+00 | 9.73E-08 | 6.98E-07 |
| KCNJ10 | 5.33E+00 | 9.81E-08 | 7.04E-07 |
| HBQ1 | 5.33E+00 | 9.81E-08 | 7.04E-07 |
| GMPPA | 5.33E+00 | 9.85E-08 | 7.06E-07 |
| CALM3 | 5.33E+00 | 9.95E-08 | 7.13E-07 |
| TGIF2LY | 5.33E+00 | 1.00E-07 | 7.19E-07 |
| SSTR2 | 5.33E+00 | 1.01E-07 | 7.20E-07 |
| PCNXL2 | 5.33E+00 | 1.01E-07 | 7.23E-07 |
| ANKZF1 | 5.32E+00 | 1.01E-07 | 7.24E-07 |
| DUSP4 | 5.32E+00 | 1.03E-07 | 7.33E-07 |
| NPHP4 | 5.32E+00 | 1.03E-07 | 7.33E-07 |
| RHPN1 | 5.32E+00 | 1.03E-07 | 7.37E-07 |
| ZFYVE27 | 5.32E+00 | 1.04E-07 | 7.41E-07 |
| ZACN | 5.32E+00 | 1.05E-07 | 7.48E-07 |
| NUP98 | 5.32E+00 | 1.06E-07 | 7.56E-07 |

| SALL4 | 5.32E+00 | 1.07E-07 | 7.61E-07 |
| --- | --- | --- | --- |
| ADAM2 | 5.31E+00 | 1.07E-07 | 7.65E-07 |
| DDB1 | 5.31E+00 | 1.07E-07 | 7.66E-07 |
| KIAA0652 | 5.31E+00 | 1.09E-07 | 7.74E-07 |
| AAMP | 5.31E+00 | 1.09E-07 | 7.75E-07 |
| NCRNA00176 | 5.31E+00 | 1.09E-07 | 7.75E-07 |
| ANP32E | 5.31E+00 | 1.09E-07 | 7.77E-07 |
| LRRC24 | 5.31E+00 | 1.10E-07 | 7.80E-07 |
| PIP4K2B | 5.31E+00 | 1.10E-07 | 7.85E-07 |
| GALR2 | 5.31E+00 | 1.11E-07 | 7.92E-07 |
| HMHA1 | 5.31E+00 | 1.11E-07 | 7.92E-07 |
| C11orf2 | 5.31E+00 | 1.12E-07 | 7.95E-07 |
| TNFRSF8 | 5.30E+00 | 1.15E-07 | 8.17E-07 |
| PRR5 | 5.30E+00 | 1.16E-07 | 8.23E-07 |
| GJA3 | 5.30E+00 | 1.16E-07 | 8.24E-07 |
| TBC1D3B | 5.30E+00 | 1.18E-07 | 8.38E-07 |
| ZNF469 | 5.30E+00 | 1.19E-07 | 8.42E-07 |
| CBX3 | 5.30E+00 | 1.19E-07 | 8.45E-07 |
| FANCE | 5.29E+00 | 1.22E-07 | 8.68E-07 |
| CASP5 | 5.29E+00 | 1.23E-07 | 8.69E-07 |
| CER1 | 5.29E+00 | 1.23E-07 | 8.69E-07 |
| KLK8 | 5.29E+00 | 1.25E-07 | 8.86E-07 |
| COPZ1 | 5.28E+00 | 1.26E-07 | 8.94E-07 |
| OR2C3 | 5.28E+00 | 1.29E-07 | 9.11E-07 |
| LOC81691 | 5.28E+00 | 1.30E-07 | 9.20E-07 |
| UPF2 | 5.28E+00 | 1.30E-07 | 9.20E-07 |
| MPP3 | 5.28E+00 | 1.31E-07 | 9.26E-07 |
| MARS2 | 5.28E+00 | 1.31E-07 | 9.28E-07 |
| RSPH6A | 5.28E+00 | 1.32E-07 | 9.30E-07 |
| PDCL2 | 5.28E+00 | 1.32E-07 | 9.32E-07 |
| BOLL | 5.28E+00 | 1.33E-07 | 9.37E-07 |
| SBSN | 5.28E+00 | 1.33E-07 | 9.37E-07 |
| PLTP | 5.27E+00 | 1.33E-07 | 9.39E-07 |
| HIST1H3F | 5.27E+00 | 1.35E-07 | 9.50E-07 |
| JAG2 | 5.27E+00 | 1.35E-07 | 9.51E-07 |
| LOC90784 | 5.27E+00 | 1.36E-07 | 9.57E-07 |
| RAD18 | 5.27E+00 | 1.36E-07 | 9.59E-07 |
| COMMD5 | 5.27E+00 | 1.36E-07 | 9.60E-07 |
| VIL1 | 5.27E+00 | 1.37E-07 | 9.67E-07 |
| FXYD2 | 5.27E+00 | 1.40E-07 | 9.83E-07 |
| MOBKL2A | 5.27E+00 | 1.40E-07 | 9.83E-07 |
| FAM65A | 5.27E+00 | 1.40E-07 | 9.85E-07 |
| LHFPL2 | 5.26E+00 | 1.43E-07 | 1.01E-06 |
| MTMR7 | 5.26E+00 | 1.44E-07 | 1.01E-06 |
| C20orf141 | 5.26E+00 | 1.45E-07 | 1.02E-06 |
| GPR84 | 5.26E+00 | 1.46E-07 | 1.02E-06 |
| H2AFB1 | 5.26E+00 | 1.46E-07 | 1.02E-06 |
| GBX2 | 5.26E+00 | 1.48E-07 | 1.04E-06 |
| MED16 | 5.26E+00 | 1.48E-07 | 1.04E-06 |
| CCNJ | 5.26E+00 | 1.48E-07 | 1.04E-06 |
| MTOR | 5.25E+00 | 1.48E-07 | 1.04E-06 |
| TBR1 | 5.25E+00 | 1.49E-07 | 1.04E-06 |
| WASH7P | 5.25E+00 | 1.50E-07 | 1.05E-06 |
| FAM124A | 5.25E+00 | 1.51E-07 | 1.06E-06 |
| TKTL2 | 5.25E+00 | 1.53E-07 | 1.07E-06 |
| NDRG3 | 5.25E+00 | 1.53E-07 | 1.07E-06 |
| PARP2 | 5.25E+00 | 1.53E-07 | 1.07E-06 |
| TMPPE | 5.25E+00 | 1.54E-07 | 1.08E-06 |

| WISP3 | 5.25E+00 | 1.57E-07 | 1.09E-06 |
| --- | --- | --- | --- |
| MGC16025 | 5.24E+00 | 1.59E-07 | 1.11E-06 |
| NRCAM | 5.24E+00 | 1.59E-07 | 1.11E-06 |
| LOC153910 | 5.24E+00 | 1.59E-07 | 1.11E-06 |
| FLJ39739 | 5.24E+00 | 1.59E-07 | 1.11E-06 |
| ATF4 | 5.24E+00 | 1.60E-07 | 1.12E-06 |
| ANO9 | 5.24E+00 | 1.60E-07 | 1.12E-06 |
| C8orf76 | 5.24E+00 | 1.62E-07 | 1.13E-06 |
| LYPLA2 | 5.24E+00 | 1.63E-07 | 1.13E-06 |
| NOTCH1 | 5.24E+00 | 1.64E-07 | 1.14E-06 |
| RTTN | 5.24E+00 | 1.64E-07 | 1.15E-06 |
| ZNF280B | 5.24E+00 | 1.65E-07 | 1.15E-06 |
| DGKG | 5.23E+00 | 1.66E-07 | 1.16E-06 |
| ABCB8 | 5.23E+00 | 1.67E-07 | 1.16E-06 |
| ACAD10 | 5.23E+00 | 1.67E-07 | 1.16E-06 |
| MRPL37 | 5.23E+00 | 1.72E-07 | 1.20E-06 |
| HDAC1 | 5.23E+00 | 1.73E-07 | 1.20E-06 |
| C19orf76 | 5.23E+00 | 1.73E-07 | 1.20E-06 |
| KTI12 | 5.22E+00 | 1.75E-07 | 1.21E-06 |
| COL22A1 | 5.22E+00 | 1.75E-07 | 1.21E-06 |
| TMEM86B | 5.22E+00 | 1.76E-07 | 1.22E-06 |
| GRM4 | 5.22E+00 | 1.76E-07 | 1.22E-06 |
| NSUN5P1 | 5.22E+00 | 1.77E-07 | 1.23E-06 |
| TAF6L | 5.22E+00 | 1.79E-07 | 1.24E-06 |
| AGAP3 | 5.22E+00 | 1.80E-07 | 1.25E-06 |
| MYB | 5.22E+00 | 1.81E-07 | 1.25E-06 |
| C22orf27 | 5.22E+00 | 1.83E-07 | 1.27E-06 |
| NRAS | 5.22E+00 | 1.83E-07 | 1.27E-06 |
| C21orf56 | 5.21E+00 | 1.85E-07 | 1.28E-06 |
| LCTL | 5.21E+00 | 1.87E-07 | 1.30E-06 |
| FAM98A | 5.21E+00 | 1.88E-07 | 1.30E-06 |
| PMS2 | 5.21E+00 | 1.89E-07 | 1.31E-06 |
| SLC4A1AP | 5.21E+00 | 1.90E-07 | 1.32E-06 |
| METTL3 | 5.21E+00 | 1.91E-07 | 1.32E-06 |
| ZNF407 | 5.21E+00 | 1.91E-07 | 1.32E-06 |
| CNOT1 | 5.21E+00 | 1.93E-07 | 1.33E-06 |
| NAP1L4 | 5.21E+00 | 1.93E-07 | 1.33E-06 |
| HSPC072 | 5.21E+00 | 1.93E-07 | 1.33E-06 |
| ZNF556 | 5.21E+00 | 1.93E-07 | 1.33E-06 |
| IGSF1 | 5.21E+00 | 1.94E-07 | 1.34E-06 |
| ATP5B | 5.20E+00 | 1.95E-07 | 1.35E-06 |
| SEMA3A | 5.20E+00 | 2.01E-07 | 1.39E-06 |
| JMJD4 | 5.20E+00 | 2.03E-07 | 1.40E-06 |
| TRAF4 | 5.20E+00 | 2.05E-07 | 1.41E-06 |
| C10orf82 | 5.20E+00 | 2.05E-07 | 1.41E-06 |
| CCL26 | 5.20E+00 | 2.05E-07 | 1.41E-06 |
| GMNN | 5.20E+00 | 2.05E-07 | 1.41E-06 |
| MAGED4B | 5.20E+00 | 2.05E-07 | 1.41E-06 |
| FJX1 | 5.19E+00 | 2.06E-07 | 1.42E-06 |
| DUS2L | 5.19E+00 | 2.08E-07 | 1.43E-06 |
| CPXM1 | 5.19E+00 | 2.10E-07 | 1.44E-06 |
| APAF1 | 5.19E+00 | 2.12E-07 | 1.46E-06 |
| RUSC1 | 5.19E+00 | 2.12E-07 | 1.46E-06 |
| HAP1 | 5.19E+00 | 2.14E-07 | 1.47E-06 |
| COX4NB | 5.19E+00 | 2.15E-07 | 1.48E-06 |
| SLCO5A1 | 5.19E+00 | 2.16E-07 | 1.48E-06 |
| CHRNA5 | 5.19E+00 | 2.16E-07 | 1.48E-06 |
| COL5A3 | 5.19E+00 | 2.16E-07 | 1.48E-06 |

| PCDHB17 | 5.18E+00 | 2.19E-07 | 1.50E-06 |
| --- | --- | --- | --- |
| C20orf96 | 5.18E+00 | 2.19E-07 | 1.50E-06 |
| ARID5A | 5.18E+00 | 2.20E-07 | 1.50E-06 |
| RPF2 | 5.18E+00 | 2.20E-07 | 1.50E-06 |
| HTRA3 | 5.18E+00 | 2.23E-07 | 1.53E-06 |
| VOPP1 | 5.18E+00 | 2.24E-07 | 1.53E-06 |
| PANX3 | 5.18E+00 | 2.24E-07 | 1.53E-06 |
| CHCHD3 | 5.18E+00 | 2.25E-07 | 1.54E-06 |
| LOC646762 | 5.18E+00 | 2.26E-07 | 1.55E-06 |
| HINFP | 5.18E+00 | 2.28E-07 | 1.56E-06 |
| BARD1 | 5.17E+00 | 2.29E-07 | 1.56E-06 |
| USP35 | 5.17E+00 | 2.30E-07 | 1.57E-06 |
| TLN1 | 5.17E+00 | 2.31E-07 | 1.57E-06 |
| ERO1L | 5.17E+00 | 2.35E-07 | 1.60E-06 |
| ZC3HAV1 | 5.17E+00 | 2.35E-07 | 1.60E-06 |
| GNB3 | 5.17E+00 | 2.38E-07 | 1.62E-06 |
| OR8A1 | 5.17E+00 | 2.39E-07 | 1.62E-06 |
| HERC2P4 | 5.17E+00 | 2.40E-07 | 1.63E-06 |
| NKX6-1 | 5.16E+00 | 2.45E-07 | 1.67E-06 |
| ARHGAP22 | 5.16E+00 | 2.47E-07 | 1.68E-06 |
| C2orf82 | 5.16E+00 | 2.48E-07 | 1.69E-06 |
| PSMG3 | 5.16E+00 | 2.49E-07 | 1.70E-06 |
| BRWD3 | 5.16E+00 | 2.50E-07 | 1.70E-06 |
| SLC45A4 | 5.16E+00 | 2.50E-07 | 1.70E-06 |
| KHDC1L | 5.16E+00 | 2.51E-07 | 1.71E-06 |
| CNOT10 | 5.16E+00 | 2.52E-07 | 1.71E-06 |
| KCNH7 | 5.16E+00 | 2.52E-07 | 1.71E-06 |
| DHRS13 | 5.16E+00 | 2.53E-07 | 1.72E-06 |
| TRIP10 | 5.15E+00 | 2.55E-07 | 1.73E-06 |
| NFKBIL1 | 5.15E+00 | 2.57E-07 | 1.74E-06 |
| ACTN1 | 5.15E+00 | 2.60E-07 | 1.76E-06 |
| C20orf191 | 5.15E+00 | 2.64E-07 | 1.79E-06 |
| EIF3CL | 5.15E+00 | 2.66E-07 | 1.80E-06 |
| PRKAA2 | 5.14E+00 | 2.74E-07 | 1.86E-06 |
| LUC7L2 | 5.14E+00 | 2.78E-07 | 1.88E-06 |
| STEAP2 | 5.14E+00 | 2.82E-07 | 1.91E-06 |
| IMPDH2 | 5.14E+00 | 2.83E-07 | 1.91E-06 |
| FKBP9 | 5.13E+00 | 2.84E-07 | 1.92E-06 |
| RPUSD4 | 5.13E+00 | 2.85E-07 | 1.93E-06 |
| C14orf145 | 5.13E+00 | 2.88E-07 | 1.95E-06 |
| ENTPD7 | 5.13E+00 | 2.95E-07 | 1.99E-06 |
| INTS4L1 | 5.12E+00 | 2.99E-07 | 2.02E-06 |
| BPTF | 5.12E+00 | 3.00E-07 | 2.02E-06 |
| WDR34 | 5.12E+00 | 3.08E-07 | 2.08E-06 |
| CHRNB2 | 5.12E+00 | 3.08E-07 | 2.08E-06 |
| WARS | 5.12E+00 | 3.09E-07 | 2.08E-06 |
| LOC100272146 | 5.12E+00 | 3.11E-07 | 2.09E-06 |
| SNRNP35 | 5.12E+00 | 3.11E-07 | 2.10E-06 |
| MYH9 | 5.12E+00 | 3.13E-07 | 2.11E-06 |
| GRAMD1B | 5.11E+00 | 3.15E-07 | 2.12E-06 |
| TRAF5 | 5.11E+00 | 3.15E-07 | 2.12E-06 |
| SMG7 | 5.11E+00 | 3.16E-07 | 2.13E-06 |
| SPEN | 5.11E+00 | 3.21E-07 | 2.16E-06 |
| GEMIN4 | 5.11E+00 | 3.23E-07 | 2.18E-06 |
| AGFG1 | 5.11E+00 | 3.30E-07 | 2.22E-06 |
| PRMT3 | 5.11E+00 | 3.30E-07 | 2.22E-06 |
| DGKQ | 5.10E+00 | 3.33E-07 | 2.24E-06 |
| SST | 5.10E+00 | 3.35E-07 | 2.25E-06 |

| CAND1 | 5.10E+00 | 3.37E-07 | 2.26E-06 |
| --- | --- | --- | --- |
| PCDHB2 | 5.10E+00 | 3.38E-07 | 2.27E-06 |
| AFG3L1 | 5.10E+00 | 3.38E-07 | 2.27E-06 |
| FAM40B | 5.10E+00 | 3.38E-07 | 2.27E-06 |
| DQX1 | 5.10E+00 | 3.43E-07 | 2.30E-06 |
| DSN1 | 5.10E+00 | 3.46E-07 | 2.32E-06 |
| MDK | 5.10E+00 | 3.46E-07 | 2.32E-06 |
| BARHL2 | 5.10E+00 | 3.48E-07 | 2.33E-06 |
| HEPACAM2 | 5.09E+00 | 3.50E-07 | 2.34E-06 |
| B4GALT5 | 5.09E+00 | 3.50E-07 | 2.34E-06 |
| RHBG | 5.09E+00 | 3.55E-07 | 2.37E-06 |
| FIZ1 | 5.09E+00 | 3.55E-07 | 2.38E-06 |
| RAB39 | 5.09E+00 | 3.61E-07 | 2.42E-06 |
| PLAC8L1 | 5.09E+00 | 3.63E-07 | 2.43E-06 |
| TMEM158 | 5.09E+00 | 3.65E-07 | 2.44E-06 |
| NPM1 | 5.09E+00 | 3.67E-07 | 2.45E-06 |
| GAST | 5.08E+00 | 3.69E-07 | 2.46E-06 |
| ZBTB24 | 5.08E+00 | 3.72E-07 | 2.48E-06 |
| PIGO | 5.08E+00 | 3.73E-07 | 2.49E-06 |
| PEX26 | 5.08E+00 | 3.73E-07 | 2.49E-06 |
| LPAR2 | 5.08E+00 | 3.75E-07 | 2.50E-06 |
| FGF11 | 5.08E+00 | 3.78E-07 | 2.52E-06 |
| MRPL4 | 5.08E+00 | 3.79E-07 | 2.52E-06 |
| FAM50A | 5.08E+00 | 3.79E-07 | 2.53E-06 |
| KIF7 | 5.08E+00 | 3.81E-07 | 2.54E-06 |
| SOX15 | 5.08E+00 | 3.81E-07 | 2.54E-06 |
| RALA | 5.08E+00 | 3.83E-07 | 2.55E-06 |
| UMPS | 5.07E+00 | 4.00E-07 | 2.66E-06 |
| FZD2 | 5.07E+00 | 4.02E-07 | 2.67E-06 |
| KIF22 | 5.07E+00 | 4.02E-07 | 2.67E-06 |
| CDHR2 | 5.07E+00 | 4.05E-07 | 2.69E-06 |
| MRPS35 | 5.07E+00 | 4.06E-07 | 2.70E-06 |
| NPC1 | 5.07E+00 | 4.06E-07 | 2.70E-06 |
| GGNBP1 | 5.07E+00 | 4.06E-07 | 2.70E-06 |
| CWC27 | 5.06E+00 | 4.16E-07 | 2.76E-06 |
| ARHGAP17 | 5.06E+00 | 4.17E-07 | 2.76E-06 |
| CDAN1 | 5.06E+00 | 4.19E-07 | 2.78E-06 |
| LAG3 | 5.06E+00 | 4.22E-07 | 2.80E-06 |
| FTHL17 | 5.06E+00 | 4.26E-07 | 2.82E-06 |
| CCDC51 | 5.06E+00 | 4.27E-07 | 2.83E-06 |
| GPR158 | 5.06E+00 | 4.27E-07 | 2.83E-06 |
| DCAF8L1 | 5.06E+00 | 4.29E-07 | 2.84E-06 |
| TWF2 | 5.06E+00 | 4.29E-07 | 2.84E-06 |
| PPARGC1B | 5.05E+00 | 4.33E-07 | 2.86E-06 |
| SFI1 | 5.05E+00 | 4.33E-07 | 2.86E-06 |
| TBPL2 | 5.05E+00 | 4.33E-07 | 2.86E-06 |
| NKAIN2 | 5.05E+00 | 4.34E-07 | 2.87E-06 |
| KLHL25 | 5.05E+00 | 4.38E-07 | 2.89E-06 |
| PRR13 | 5.05E+00 | 4.43E-07 | 2.93E-06 |
| C17orf90 | 5.05E+00 | 4.47E-07 | 2.96E-06 |
| PLAUR | 5.05E+00 | 4.49E-07 | 2.96E-06 |
| NOC3L | 5.05E+00 | 4.50E-07 | 2.97E-06 |
| ZNHIT6 | 5.05E+00 | 4.50E-07 | 2.97E-06 |
| MRPS2 | 5.04E+00 | 4.56E-07 | 3.01E-06 |
| LILRA3 | 5.03E+00 | 4.79E-07 | 3.16E-06 |
| FABP5 | 5.03E+00 | 4.82E-07 | 3.18E-06 |
| TCL1B | 5.03E+00 | 4.83E-07 | 3.18E-06 |
| USP1 | 5.03E+00 | 4.85E-07 | 3.19E-06 |

| BRF1 | 5.03E+00 | 4.88E-07 | 3.21E-06 |
| --- | --- | --- | --- |
| SLC20A1 | 5.03E+00 | 4.91E-07 | 3.23E-06 |
| SSX2 | 5.03E+00 | 4.94E-07 | 3.25E-06 |
| RGS9BP | 5.03E+00 | 4.95E-07 | 3.26E-06 |
| ADNP | 5.03E+00 | 4.98E-07 | 3.27E-06 |
| IWS1 | 5.03E+00 | 5.01E-07 | 3.29E-06 |
| NT5DC2 | 5.03E+00 | 5.01E-07 | 3.29E-06 |
| PARS2 | 5.03E+00 | 5.03E-07 | 3.31E-06 |
| CPA2 | 5.02E+00 | 5.07E-07 | 3.33E-06 |
| DDX19A | 5.02E+00 | 5.14E-07 | 3.37E-06 |
| GAGE2E | 5.02E+00 | 5.18E-07 | 3.40E-06 |
| PAGE2 | 5.02E+00 | 5.21E-07 | 3.42E-06 |
| STYK1 | 5.02E+00 | 5.22E-07 | 3.42E-06 |
| OR2B6 | 5.01E+00 | 5.37E-07 | 3.52E-06 |
| UBE3C | 5.01E+00 | 5.38E-07 | 3.53E-06 |
| CYP27B1 | 5.01E+00 | 5.39E-07 | 3.53E-06 |
| HIST1H2BO | 5.01E+00 | 5.41E-07 | 3.54E-06 |
| QRICH2 | 5.01E+00 | 5.55E-07 | 3.63E-06 |
| HYAL4 | 5.01E+00 | 5.55E-07 | 3.63E-06 |
| ANKS1A | 5.01E+00 | 5.57E-07 | 3.64E-06 |
| MGC45800 | 5.00E+00 | 5.60E-07 | 3.67E-06 |
| MYO18A | 5.00E+00 | 5.63E-07 | 3.68E-06 |
| POFUT2 | 5.00E+00 | 5.64E-07 | 3.69E-06 |
| PPP2R5D | 5.00E+00 | 5.66E-07 | 3.70E-06 |
| FNDC3B | 5.00E+00 | 5.71E-07 | 3.73E-06 |
| PRIC285 | 5.00E+00 | 5.74E-07 | 3.75E-06 |
| KIAA0182 | 5.00E+00 | 5.77E-07 | 3.77E-06 |
| DLAT | 5.00E+00 | 5.78E-07 | 3.77E-06 |
| KIAA0892 | 5.00E+00 | 5.78E-07 | 3.77E-06 |
| HIST1H3I | 5.00E+00 | 5.86E-07 | 3.82E-06 |
| BSG | 5.00E+00 | 5.89E-07 | 3.84E-06 |
| HIST2H2BF | 4.99E+00 | 5.93E-07 | 3.86E-06 |
| BAIAP2L2 | 4.99E+00 | 5.94E-07 | 3.87E-06 |
| MGA | 4.99E+00 | 5.95E-07 | 3.88E-06 |
| C1orf220 | 4.99E+00 | 5.99E-07 | 3.90E-06 |
| INHBE | 4.99E+00 | 6.01E-07 | 3.91E-06 |
| HIST1H2BL | 4.99E+00 | 6.06E-07 | 3.94E-06 |
| PIGW | 4.99E+00 | 6.10E-07 | 3.97E-06 |
| C20orf135 | 4.99E+00 | 6.12E-07 | 3.98E-06 |
| COPE | 4.99E+00 | 6.19E-07 | 4.02E-06 |
| FAM75C1 | 4.99E+00 | 6.19E-07 | 4.02E-06 |
| CNOT6 | 4.98E+00 | 6.23E-07 | 4.05E-06 |
| IPO13 | 4.98E+00 | 6.29E-07 | 4.09E-06 |
| CT45A2 | 4.98E+00 | 6.32E-07 | 4.10E-06 |
| PRKAR1B | 4.98E+00 | 6.33E-07 | 4.11E-06 |
| KRTAP4-1 | 4.98E+00 | 6.35E-07 | 4.12E-06 |
| LZTR1 | 4.98E+00 | 6.35E-07 | 4.12E-06 |
| FLJ25758 | 4.98E+00 | 6.39E-07 | 4.14E-06 |
| NOSIP | 4.98E+00 | 6.39E-07 | 4.14E-06 |
| LOC100131551 | 4.98E+00 | 6.44E-07 | 4.17E-06 |
| PNMA3 | 4.98E+00 | 6.46E-07 | 4.19E-06 |
| C17orf71 | 4.98E+00 | 6.53E-07 | 4.23E-06 |
| B3GALNT2 | 4.97E+00 | 6.56E-07 | 4.25E-06 |
| EVC2 | 4.97E+00 | 6.61E-07 | 4.28E-06 |
| ACVR2B | 4.97E+00 | 6.62E-07 | 4.28E-06 |
| JAK3 | 4.97E+00 | 6.61E-07 | 4.28E-06 |
| GDI1 | 4.97E+00 | 6.63E-07 | 4.28E-06 |
| NT5C3 | 4.97E+00 | 6.66E-07 | 4.30E-06 |

| CKM | 4.97E+00 | 6.68E-07 | 4.32E-06 |
| --- | --- | --- | --- |
| MGC2752 | 4.97E+00 | 6.74E-07 | 4.35E-06 |
| COPS6 | 4.97E+00 | 6.82E-07 | 4.40E-06 |
| C2orf78 | 4.97E+00 | 6.83E-07 | 4.41E-06 |
| EPHB2 | 4.97E+00 | 6.83E-07 | 4.41E-06 |
| RAB10 | 4.97E+00 | 6.86E-07 | 4.42E-06 |
| RBM8A | 4.96E+00 | 6.95E-07 | 4.48E-06 |
| DGKK | 4.96E+00 | 6.98E-07 | 4.50E-06 |
| THOC3 | 4.96E+00 | 7.01E-07 | 4.51E-06 |
| LRP4 | 4.96E+00 | 7.11E-07 | 4.58E-06 |
| OTX2 | 4.96E+00 | 7.12E-07 | 4.59E-06 |
| DKFZP434L187 | 4.96E+00 | 7.14E-07 | 4.60E-06 |
| TIMM17B | 4.96E+00 | 7.20E-07 | 4.64E-06 |
| SDCCAG3 | 4.96E+00 | 7.23E-07 | 4.65E-06 |
| WIPF2 | 4.95E+00 | 7.34E-07 | 4.72E-06 |
| ABCB6 | 4.95E+00 | 7.35E-07 | 4.72E-06 |
| LZTS1 | 4.95E+00 | 7.35E-07 | 4.72E-06 |
| SPPL2B | 4.95E+00 | 7.36E-07 | 4.73E-06 |
| DEFB126 | 4.95E+00 | 7.40E-07 | 4.76E-06 |
| GSS | 4.95E+00 | 7.42E-07 | 4.76E-06 |
| HIST1H2BF | 4.95E+00 | 7.51E-07 | 4.82E-06 |
| CYP2W1 | 4.95E+00 | 7.58E-07 | 4.86E-06 |
| HIST1H3C | 4.94E+00 | 7.67E-07 | 4.92E-06 |
| CT45A1 | 4.94E+00 | 7.75E-07 | 4.97E-06 |
| GRIP2 | 4.94E+00 | 7.77E-07 | 4.98E-06 |
| HNRNPA1L2 | 4.94E+00 | 7.80E-07 | 5.00E-06 |
| LOC100288778 | 4.94E+00 | 7.80E-07 | 5.00E-06 |
| ARL6IP4 | 4.94E+00 | 7.82E-07 | 5.01E-06 |
| ZNF93 | 4.94E+00 | 7.82E-07 | 5.01E-06 |
| GPR97 | 4.94E+00 | 7.87E-07 | 5.04E-06 |
| ZFP42 | 4.94E+00 | 7.99E-07 | 5.11E-06 |
| MCM3AP | 4.93E+00 | 8.05E-07 | 5.15E-06 |
| TSNARE1 | 4.93E+00 | 8.06E-07 | 5.15E-06 |
| AGMAT | 4.93E+00 | 8.16E-07 | 5.22E-06 |
| ZP1 | 4.93E+00 | 8.23E-07 | 5.26E-06 |
| ZC3H8 | 4.93E+00 | 8.24E-07 | 5.26E-06 |
| LENG8 | 4.93E+00 | 8.26E-07 | 5.27E-06 |
| PRL | 4.93E+00 | 8.33E-07 | 5.32E-06 |
| UAP1L1 | 4.92E+00 | 8.46E-07 | 5.40E-06 |
| FSTL5 | 4.92E+00 | 8.50E-07 | 5.42E-06 |
| GPR109B | 4.92E+00 | 8.63E-07 | 5.51E-06 |
| HNRNPH1 | 4.92E+00 | 8.68E-07 | 5.54E-06 |
| ENAH | 4.92E+00 | 8.85E-07 | 5.64E-06 |
| NUAK1 | 4.92E+00 | 8.88E-07 | 5.66E-06 |
| TBC1D22A | 4.92E+00 | 8.88E-07 | 5.66E-06 |
| PSMB2 | 4.91E+00 | 8.93E-07 | 5.68E-06 |
| FADS2 | 4.91E+00 | 8.96E-07 | 5.70E-06 |
| DDOST | 4.91E+00 | 8.97E-07 | 5.71E-06 |
| SCAMP5 | 4.91E+00 | 8.99E-07 | 5.72E-06 |
| GRIK5 | 4.91E+00 | 9.01E-07 | 5.73E-06 |
| CCDC163P | 4.91E+00 | 9.04E-07 | 5.75E-06 |
| MDFI | 4.91E+00 | 9.10E-07 | 5.78E-06 |
| SPATA5L1 | 4.91E+00 | 9.11E-07 | 5.79E-06 |
| SNPH | 4.91E+00 | 9.16E-07 | 5.82E-06 |
| CCDC59 | 4.91E+00 | 9.17E-07 | 5.82E-06 |
| P2RX4 | 4.91E+00 | 9.22E-07 | 5.86E-06 |
| MRPL3 | 4.91E+00 | 9.33E-07 | 5.92E-06 |
| FAM128B | 4.90E+00 | 9.37E-07 | 5.95E-06 |

| TMEM106C | 4.90E+00 | 9.53E-07 | 6.04E-06 |
| --- | --- | --- | --- |
| FAM168A | 4.90E+00 | 9.61E-07 | 6.09E-06 |
| ZNF300 | 4.90E+00 | 9.61E-07 | 6.09E-06 |
| MAP4K4 | 4.90E+00 | 9.73E-07 | 6.16E-06 |
| ADAMTS4 | 4.90E+00 | 9.76E-07 | 6.18E-06 |
| KIAA1614 | 4.90E+00 | 9.76E-07 | 6.18E-06 |
| SLC6A9 | 4.90E+00 | 9.84E-07 | 6.23E-06 |
| CDYL | 4.89E+00 | 9.88E-07 | 6.25E-06 |
| HOXC5 | 4.89E+00 | 9.92E-07 | 6.28E-06 |
| ALOXE3 | 4.89E+00 | 1.00E-06 | 6.32E-06 |
| ATP6V0A4 | 4.89E+00 | 1.00E-06 | 6.32E-06 |
| CALY | 4.89E+00 | 1.00E-06 | 6.33E-06 |
| DDX46 | 4.89E+00 | 1.00E-06 | 6.33E-06 |
| HK3 | 4.89E+00 | 1.00E-06 | 6.33E-06 |
| TPR | 4.89E+00 | 1.00E-06 | 6.34E-06 |
| DNAJC13 | 4.89E+00 | 1.01E-06 | 6.40E-06 |
| ARNTL2 | 4.89E+00 | 1.02E-06 | 6.44E-06 |
| VENTXP1 | 4.89E+00 | 1.04E-06 | 6.54E-06 |
| SF3A1 | 4.88E+00 | 1.04E-06 | 6.54E-06 |
| LOC339535 | 4.88E+00 | 1.04E-06 | 6.56E-06 |
| ELP2P | 4.88E+00 | 1.07E-06 | 6.71E-06 |
| ZNF716 | 4.88E+00 | 1.07E-06 | 6.76E-06 |
| CDC42EP2 | 4.88E+00 | 1.07E-06 | 6.76E-06 |
| MAGEB1 | 4.88E+00 | 1.08E-06 | 6.77E-06 |
| ANP32A | 4.88E+00 | 1.08E-06 | 6.79E-06 |
| ALDH1L2 | 4.88E+00 | 1.08E-06 | 6.81E-06 |
| MLL | 4.87E+00 | 1.10E-06 | 6.90E-06 |
| HIST1H4I | 4.87E+00 | 1.10E-06 | 6.93E-06 |
| ABCC2 | 4.87E+00 | 1.11E-06 | 6.95E-06 |
| KCNJ9 | 4.87E+00 | 1.12E-06 | 7.01E-06 |
| PABPC1L | 4.87E+00 | 1.12E-06 | 7.03E-06 |
| GTF2F1 | 4.87E+00 | 1.12E-06 | 7.03E-06 |
| OR7E5P | 4.87E+00 | 1.12E-06 | 7.04E-06 |
| ZNF827 | 4.87E+00 | 1.13E-06 | 7.08E-06 |
| C11orf83 | 4.87E+00 | 1.13E-06 | 7.10E-06 |
| FAM160B2 | 4.87E+00 | 1.14E-06 | 7.18E-06 |
| USP20 | 4.86E+00 | 1.16E-06 | 7.27E-06 |
| FOXD1 | 4.86E+00 | 1.16E-06 | 7.27E-06 |
| INTS2 | 4.86E+00 | 1.17E-06 | 7.31E-06 |
| NPEPPS | 4.86E+00 | 1.17E-06 | 7.31E-06 |
| BAP1 | 4.86E+00 | 1.17E-06 | 7.32E-06 |
| OBSCN | 4.86E+00 | 1.17E-06 | 7.34E-06 |
| ARL9 | 4.86E+00 | 1.18E-06 | 7.36E-06 |
| KCNH4 | 4.86E+00 | 1.18E-06 | 7.37E-06 |
| STXBP5 | 4.86E+00 | 1.18E-06 | 7.37E-06 |
| TRPM5 | 4.86E+00 | 1.19E-06 | 7.43E-06 |
| IL20RB | 4.86E+00 | 1.19E-06 | 7.45E-06 |
| FLJ16779 | 4.86E+00 | 1.19E-06 | 7.46E-06 |
| ALG11 | 4.86E+00 | 1.20E-06 | 7.47E-06 |
| SLITRK1 | 4.86E+00 | 1.20E-06 | 7.51E-06 |
| DGKD | 4.85E+00 | 1.21E-06 | 7.54E-06 |
| FRAT2 | 4.85E+00 | 1.21E-06 | 7.54E-06 |
| C12orf75 | 4.85E+00 | 1.22E-06 | 7.61E-06 |
| HOXC10 | 4.85E+00 | 1.23E-06 | 7.68E-06 |
| RIC8B | 4.85E+00 | 1.23E-06 | 7.68E-06 |
| SMEK3P | 4.85E+00 | 1.23E-06 | 7.68E-06 |
| LOC100133331 | 4.85E+00 | 1.24E-06 | 7.71E-06 |
| ADAMDEC1 | 4.85E+00 | 1.25E-06 | 7.80E-06 |

| PSMB5 | 4.85E+00 | 1.26E-06 | 7.81E-06 |
| --- | --- | --- | --- |
| ZNF492 | 4.85E+00 | 1.26E-06 | 7.83E-06 |
| RDBP | 4.85E+00 | 1.26E-06 | 7.84E-06 |
| LOC100132354 | 4.85E+00 | 1.26E-06 | 7.85E-06 |
| TCEB3B | 4.85E+00 | 1.26E-06 | 7.85E-06 |
| SPANXE | 4.85E+00 | 1.27E-06 | 7.88E-06 |
| OR2H2 | 4.84E+00 | 1.27E-06 | 7.91E-06 |
| KCNMB4 | 4.84E+00 | 1.28E-06 | 7.92E-06 |
| AEN | 4.84E+00 | 1.30E-06 | 8.10E-06 |
| TNFRSF25 | 4.84E+00 | 1.31E-06 | 8.13E-06 |
| ARPC3 | 4.84E+00 | 1.31E-06 | 8.13E-06 |
| NR6A1 | 4.84E+00 | 1.33E-06 | 8.27E-06 |
| MFN1 | 4.83E+00 | 1.34E-06 | 8.31E-06 |
| KCNA7 | 4.83E+00 | 1.35E-06 | 8.39E-06 |
| FAM176B | 4.83E+00 | 1.36E-06 | 8.43E-06 |
| WDR6 | 4.83E+00 | 1.36E-06 | 8.44E-06 |
| MTX1 | 4.83E+00 | 1.36E-06 | 8.45E-06 |
| C6orf134 | 4.83E+00 | 1.38E-06 | 8.55E-06 |
| LY6K | 4.83E+00 | 1.38E-06 | 8.56E-06 |
| PNP | 4.82E+00 | 1.41E-06 | 8.70E-06 |
| OR1J2 | 4.82E+00 | 1.41E-06 | 8.70E-06 |
| C20orf46 | 4.82E+00 | 1.41E-06 | 8.71E-06 |
| MAGEB6 | 4.82E+00 | 1.41E-06 | 8.71E-06 |
| ANP32B | 4.82E+00 | 1.43E-06 | 8.85E-06 |
| GAGE13 | 4.82E+00 | 1.44E-06 | 8.88E-06 |
| LOC440356 | 4.82E+00 | 1.45E-06 | 8.96E-06 |
| FEV | 4.82E+00 | 1.46E-06 | 8.98E-06 |
| ZNF589 | 4.82E+00 | 1.46E-06 | 9.01E-06 |
| RNF166 | 4.82E+00 | 1.47E-06 | 9.05E-06 |
| ZCCHC3 | 4.82E+00 | 1.47E-06 | 9.07E-06 |
| SNRPC | 4.82E+00 | 1.47E-06 | 9.08E-06 |
| NLRC5 | 4.81E+00 | 1.49E-06 | 9.17E-06 |
| GPR156 | 4.81E+00 | 1.49E-06 | 9.18E-06 |
| ZNF445 | 4.81E+00 | 1.50E-06 | 9.27E-06 |
| SPAG4 | 4.81E+00 | 1.55E-06 | 9.54E-06 |
| ANKRD26P1 | 4.81E+00 | 1.55E-06 | 9.55E-06 |
| NBPF22P | 4.80E+00 | 1.56E-06 | 9.59E-06 |
| ZNF98 | 4.80E+00 | 1.56E-06 | 9.60E-06 |
| ZNF653 | 4.80E+00 | 1.57E-06 | 9.65E-06 |
| HDAC10 | 4.80E+00 | 1.59E-06 | 9.78E-06 |
| PDCD5 | 4.80E+00 | 1.60E-06 | 9.84E-06 |
| OR7E156P | 4.80E+00 | 1.61E-06 | 9.90E-06 |
| RQCD1 | 4.80E+00 | 1.62E-06 | 9.92E-06 |
| GLIS2 | 4.80E+00 | 1.62E-06 | 9.93E-06 |
| GALNT6 | 4.80E+00 | 1.62E-06 | 9.98E-06 |
| TFG | 4.80E+00 | 1.63E-06 | 1.00E-05 |
| BATF2 | 4.79E+00 | 1.63E-06 | 1.00E-05 |
| ARL2 | 4.79E+00 | 1.64E-06 | 1.00E-05 |
| PMM2 | 4.79E+00 | 1.65E-06 | 1.01E-05 |
| SPTBN2 | 4.79E+00 | 1.65E-06 | 1.01E-05 |
| SLC25A32 | 4.79E+00 | 1.66E-06 | 1.02E-05 |
| TAL2 | 4.79E+00 | 1.66E-06 | 1.02E-05 |
| EPN3 | 4.79E+00 | 1.67E-06 | 1.03E-05 |
| DPYSL5 | 4.79E+00 | 1.67E-06 | 1.03E-05 |
| AHSA1 | 4.79E+00 | 1.68E-06 | 1.03E-05 |
| PLEKHJ1 | 4.79E+00 | 1.70E-06 | 1.04E-05 |
| PPP4C | 4.79E+00 | 1.70E-06 | 1.04E-05 |
| PAX8 | 4.79E+00 | 1.71E-06 | 1.04E-05 |

| VAV2 | 4.78E+00 | 1.75E-06 | 1.07E-05 |
| --- | --- | --- | --- |
| CSH2 | 4.78E+00 | 1.75E-06 | 1.07E-05 |
| EFCAB3 | 4.78E+00 | 1.75E-06 | 1.07E-05 |
| HRNBP3 | 4.78E+00 | 1.75E-06 | 1.07E-05 |
| FCHSD1 | 4.78E+00 | 1.76E-06 | 1.08E-05 |
| TSGA14 | 4.78E+00 | 1.78E-06 | 1.09E-05 |
| C2orf14 | 4.78E+00 | 1.78E-06 | 1.09E-05 |
| TMEM8A | 4.78E+00 | 1.79E-06 | 1.09E-05 |
| GALNT8 | 4.78E+00 | 1.79E-06 | 1.09E-05 |
| LOC728643 | 4.78E+00 | 1.80E-06 | 1.10E-05 |
| IL12RB2 | 4.77E+00 | 1.80E-06 | 1.10E-05 |
| TRPM6 | 4.77E+00 | 1.83E-06 | 1.11E-05 |
| SLC10A3 | 4.77E+00 | 1.84E-06 | 1.12E-05 |
| SNHG12 | 4.77E+00 | 1.84E-06 | 1.12E-05 |
| WNT10B | 4.77E+00 | 1.85E-06 | 1.13E-05 |
| C4orf50 | 4.77E+00 | 1.85E-06 | 1.13E-05 |
| ENO2 | 4.77E+00 | 1.87E-06 | 1.14E-05 |
| EIF5AL1 | 4.77E+00 | 1.89E-06 | 1.15E-05 |
| ANAPC2 | 4.76E+00 | 1.91E-06 | 1.16E-05 |
| ADAMTS5 | 4.76E+00 | 1.92E-06 | 1.17E-05 |
| NAA35 | 4.76E+00 | 1.94E-06 | 1.18E-05 |
| ZNF213 | 4.76E+00 | 1.95E-06 | 1.18E-05 |
| CENPP | 4.76E+00 | 1.95E-06 | 1.18E-05 |
| HES7 | 4.76E+00 | 1.95E-06 | 1.18E-05 |
| C1orf85 | 4.76E+00 | 1.96E-06 | 1.19E-05 |
| FAM46D | 4.76E+00 | 1.98E-06 | 1.20E-05 |
| BEST1 | 4.76E+00 | 1.99E-06 | 1.21E-05 |
| SENP5 | 4.75E+00 | 2.01E-06 | 1.22E-05 |
| RNF186 | 4.75E+00 | 2.03E-06 | 1.23E-05 |
| FAM195B | 4.75E+00 | 2.04E-06 | 1.24E-05 |
| TMEM145 | 4.75E+00 | 2.04E-06 | 1.24E-05 |
| FAM40A | 4.75E+00 | 2.05E-06 | 1.24E-05 |
| KCNC1 | 4.75E+00 | 2.06E-06 | 1.25E-05 |
| TRIM31 | 4.75E+00 | 2.06E-06 | 1.25E-05 |
| MRPS5 | 4.75E+00 | 2.07E-06 | 1.25E-05 |
| ADM2 | 4.75E+00 | 2.08E-06 | 1.26E-05 |
| GTPBP1 | 4.75E+00 | 2.09E-06 | 1.26E-05 |
| GBX1 | 4.75E+00 | 2.09E-06 | 1.26E-05 |
| HIST1H2AJ | 4.75E+00 | 2.09E-06 | 1.26E-05 |
| OR1J1 | 4.74E+00 | 2.09E-06 | 1.26E-05 |
| HRNR | 4.74E+00 | 2.10E-06 | 1.27E-05 |
| UBXN7 | 4.74E+00 | 2.13E-06 | 1.29E-05 |
| GOLGA8C | 4.74E+00 | 2.14E-06 | 1.29E-05 |
| DCAF4L2 | 4.74E+00 | 2.15E-06 | 1.30E-05 |
| DDIT3 | 4.74E+00 | 2.15E-06 | 1.30E-05 |
| SLC39A4 | 4.74E+00 | 2.16E-06 | 1.30E-05 |
| SMN2 | 4.74E+00 | 2.17E-06 | 1.31E-05 |
| LOC441089 | 4.73E+00 | 2.23E-06 | 1.34E-05 |
| ENTPD6 | 4.73E+00 | 2.24E-06 | 1.35E-05 |
| SF3A3 | 4.73E+00 | 2.26E-06 | 1.36E-05 |
| DLGAP3 | 4.73E+00 | 2.27E-06 | 1.37E-05 |
| OLA1 | 4.73E+00 | 2.29E-06 | 1.38E-05 |
| KIRREL2 | 4.73E+00 | 2.31E-06 | 1.39E-05 |
| MYOD1 | 4.72E+00 | 2.31E-06 | 1.39E-05 |
| LOC100128842 | 4.72E+00 | 2.31E-06 | 1.39E-05 |
| GOLGA2 | 4.72E+00 | 2.31E-06 | 1.39E-05 |
| GNL2 | 4.72E+00 | 2.32E-06 | 1.40E-05 |
| ACSL3 | 4.72E+00 | 2.33E-06 | 1.40E-05 |

| PAX3 | 4.72E+00 | 2.33E-06 | 1.40E-05 |
| --- | --- | --- | --- |
| ZIC2 | 4.72E+00 | 2.35E-06 | 1.41E-05 |
| B3GALT6 | 4.72E+00 | 2.35E-06 | 1.41E-05 |
| DOK3 | 4.72E+00 | 2.36E-06 | 1.42E-05 |
| PFKL | 4.72E+00 | 2.37E-06 | 1.42E-05 |
| FAM127B | 4.72E+00 | 2.37E-06 | 1.42E-05 |
| VCX2 | 4.72E+00 | 2.39E-06 | 1.43E-05 |
| EFNA3 | 4.72E+00 | 2.40E-06 | 1.44E-05 |
| C1orf144 | 4.72E+00 | 2.42E-06 | 1.45E-05 |
| SIP1 | 4.72E+00 | 2.42E-06 | 1.45E-05 |
| GABPB1 | 4.72E+00 | 2.42E-06 | 1.45E-05 |
| LOC400696 | 4.72E+00 | 2.42E-06 | 1.45E-05 |
| SLC7A3 | 4.71E+00 | 2.43E-06 | 1.45E-05 |
| GRK7 | 4.71E+00 | 2.46E-06 | 1.47E-05 |
| CALB1 | 4.71E+00 | 2.47E-06 | 1.48E-05 |
| PGAM1 | 4.71E+00 | 2.48E-06 | 1.48E-05 |
| OR8D2 | 4.71E+00 | 2.48E-06 | 1.48E-05 |
| SPATA8 | 4.71E+00 | 2.49E-06 | 1.49E-05 |
| MC1R | 4.71E+00 | 2.49E-06 | 1.49E-05 |
| QRFP | 4.71E+00 | 2.49E-06 | 1.49E-05 |
| SULF1 | 4.71E+00 | 2.50E-06 | 1.49E-05 |
| USH1C | 4.71E+00 | 2.50E-06 | 1.49E-05 |
| RPA1 | 4.71E+00 | 2.51E-06 | 1.50E-05 |
| FFAR2 | 4.71E+00 | 2.53E-06 | 1.51E-05 |
| C19orf21 | 4.70E+00 | 2.55E-06 | 1.52E-05 |
| HIST1H4D | 4.70E+00 | 2.57E-06 | 1.53E-05 |
| KCTD2 | 4.70E+00 | 2.57E-06 | 1.53E-05 |
| C9orf109 | 4.70E+00 | 2.58E-06 | 1.54E-05 |
| BAK1 | 4.70E+00 | 2.60E-06 | 1.55E-05 |
| USP11 | 4.70E+00 | 2.61E-06 | 1.55E-05 |
| FADD | 4.70E+00 | 2.64E-06 | 1.57E-05 |
| TMEM81 | 4.70E+00 | 2.64E-06 | 1.57E-05 |
| NPPB | 4.69E+00 | 2.68E-06 | 1.59E-05 |
| MAPK8IP2 | 4.69E+00 | 2.69E-06 | 1.60E-05 |
| GYS1 | 4.69E+00 | 2.70E-06 | 1.61E-05 |
| CENPT | 4.69E+00 | 2.73E-06 | 1.62E-05 |
| LUZP4 | 4.69E+00 | 2.76E-06 | 1.64E-05 |
| PHF20L1 | 4.69E+00 | 2.76E-06 | 1.64E-05 |
| YY2 | 4.69E+00 | 2.78E-06 | 1.65E-05 |
| STAG1 | 4.69E+00 | 2.79E-06 | 1.66E-05 |
| POU4F2 | 4.69E+00 | 2.80E-06 | 1.66E-05 |
| CELF3 | 4.69E+00 | 2.80E-06 | 1.66E-05 |
| G3BP1 | 4.68E+00 | 2.82E-06 | 1.68E-05 |
| HIST2H2BA | 4.68E+00 | 2.93E-06 | 1.74E-05 |
| SLIT1 | 4.68E+00 | 2.93E-06 | 1.74E-05 |
| LOC100131193 | 4.67E+00 | 2.96E-06 | 1.75E-05 |
| TNFRSF21 | 4.67E+00 | 2.96E-06 | 1.76E-05 |
| CDK5RAP2 | 4.67E+00 | 3.00E-06 | 1.78E-05 |
| FOXH1 | 4.67E+00 | 3.02E-06 | 1.79E-05 |
| SLC7A6 | 4.67E+00 | 3.04E-06 | 1.80E-05 |
| FBN2 | 4.67E+00 | 3.04E-06 | 1.80E-05 |
| CD177 | 4.67E+00 | 3.04E-06 | 1.80E-05 |
| PLEKHH3 | 4.67E+00 | 3.09E-06 | 1.83E-05 |
| PITPNM2 | 4.66E+00 | 3.12E-06 | 1.85E-05 |
| HIST1H2BG | 4.66E+00 | 3.14E-06 | 1.85E-05 |
| FAM113B | 4.66E+00 | 3.14E-06 | 1.86E-05 |
| C12orf70 | 4.66E+00 | 3.15E-06 | 1.86E-05 |
| PSMD14 | 4.66E+00 | 3.17E-06 | 1.87E-05 |

| LYG2 | 4.66E+00 | 3.18E-06 | 1.88E-05 |
| --- | --- | --- | --- |
| ABCE1 | 4.66E+00 | 3.21E-06 | 1.90E-05 |
| RALBP1 | 4.66E+00 | 3.24E-06 | 1.91E-05 |
| NEUROD4 | 4.65E+00 | 3.26E-06 | 1.92E-05 |
| OTUD6B | 4.65E+00 | 3.26E-06 | 1.92E-05 |
| PDIA2 | 4.65E+00 | 3.26E-06 | 1.92E-05 |
| CA9 | 4.65E+00 | 3.27E-06 | 1.93E-05 |
| AGPAT6 | 4.65E+00 | 3.31E-06 | 1.95E-05 |
| RPS6KC1 | 4.65E+00 | 3.31E-06 | 1.95E-05 |
| TMEM88B | 4.65E+00 | 3.33E-06 | 1.96E-05 |
| MFSD3 | 4.65E+00 | 3.34E-06 | 1.96E-05 |
| NDUFA4L2 | 4.65E+00 | 3.36E-06 | 1.98E-05 |
| TREML4 | 4.65E+00 | 3.36E-06 | 1.98E-05 |
| TTC22 | 4.65E+00 | 3.37E-06 | 1.98E-05 |
| LRRC47 | 4.65E+00 | 3.38E-06 | 1.99E-05 |
| PLCD3 | 4.65E+00 | 3.38E-06 | 1.99E-05 |
| UBE2R2 | 4.65E+00 | 3.38E-06 | 1.99E-05 |
| ZNF789 | 4.65E+00 | 3.41E-06 | 2.00E-05 |
| NES | 4.64E+00 | 3.41E-06 | 2.00E-05 |
| SSH2 | 4.64E+00 | 3.43E-06 | 2.01E-05 |
| FADS1 | 4.64E+00 | 3.44E-06 | 2.02E-05 |
| CPSF7 | 4.64E+00 | 3.45E-06 | 2.02E-05 |
| PGAM4 | 4.64E+00 | 3.46E-06 | 2.03E-05 |
| RIN1 | 4.64E+00 | 3.47E-06 | 2.03E-05 |
| TAS2R43 | 4.64E+00 | 3.47E-06 | 2.03E-05 |
| LILRB4 | 4.64E+00 | 3.47E-06 | 2.03E-05 |
| SPANXA2 | 4.64E+00 | 3.50E-06 | 2.05E-05 |
| QPRT | 4.64E+00 | 3.52E-06 | 2.06E-05 |
| CDK2AP1 | 4.64E+00 | 3.56E-06 | 2.09E-05 |
| DIDO1 | 4.64E+00 | 3.56E-06 | 2.09E-05 |
| KCNH5 | 4.64E+00 | 3.57E-06 | 2.09E-05 |
| AANAT | 4.64E+00 | 3.57E-06 | 2.09E-05 |
| PAGE2B | 4.63E+00 | 3.60E-06 | 2.10E-05 |
| SLC26A10 | 4.63E+00 | 3.61E-06 | 2.11E-05 |
| BLMH | 4.63E+00 | 3.61E-06 | 2.11E-05 |
| RALGAPB | 4.63E+00 | 3.65E-06 | 2.13E-05 |
| HOXB8 | 4.63E+00 | 3.67E-06 | 2.14E-05 |
| DMAP1 | 4.63E+00 | 3.67E-06 | 2.14E-05 |
| CLIP2 | 4.63E+00 | 3.67E-06 | 2.14E-05 |
| CDC42BPG | 4.63E+00 | 3.70E-06 | 2.16E-05 |
| MGC70857 | 4.63E+00 | 3.73E-06 | 2.18E-05 |
| RECQL | 4.63E+00 | 3.75E-06 | 2.19E-05 |
| SMTNL1 | 4.63E+00 | 3.75E-06 | 2.19E-05 |
| ZNF280C | 4.63E+00 | 3.75E-06 | 2.19E-05 |
| PHF14 | 4.62E+00 | 3.77E-06 | 2.20E-05 |
| FABP6 | 4.62E+00 | 3.78E-06 | 2.20E-05 |
| ZNF764 | 4.62E+00 | 3.78E-06 | 2.20E-05 |
| QTRT1 | 4.62E+00 | 3.81E-06 | 2.22E-05 |
| RHPN2 | 4.62E+00 | 3.83E-06 | 2.23E-05 |
| RPL10L | 4.62E+00 | 3.84E-06 | 2.24E-05 |
| LOC100271836 | 4.62E+00 | 3.86E-06 | 2.25E-05 |
| WDR1 | 4.62E+00 | 3.87E-06 | 2.25E-05 |
| USP24 | 4.62E+00 | 3.88E-06 | 2.26E-05 |
| ANKRD32 | 4.62E+00 | 3.89E-06 | 2.26E-05 |
| OR4C6 | 4.62E+00 | 3.89E-06 | 2.26E-05 |
| THOC1 | 4.62E+00 | 3.89E-06 | 2.26E-05 |
| ZNF517 | 4.62E+00 | 3.89E-06 | 2.26E-05 |
| SSX2IP | 4.62E+00 | 3.89E-06 | 2.26E-05 |

| NAP1L1 | 4.62E+00 | 3.91E-06 | 2.27E-05 |
| --- | --- | --- | --- |
| SLC5A12 | 4.62E+00 | 3.93E-06 | 2.28E-05 |
| QRICH1 | 4.62E+00 | 3.93E-06 | 2.28E-05 |
| LOC100132247 | 4.62E+00 | 3.94E-06 | 2.28E-05 |
| GRIN1 | 4.61E+00 | 3.97E-06 | 2.30E-05 |
| QPCT | 4.61E+00 | 3.99E-06 | 2.31E-05 |
| CLPTM1 | 4.61E+00 | 4.02E-06 | 2.33E-05 |
| TCAM1P | 4.61E+00 | 4.02E-06 | 2.33E-05 |
| TGIF2LX | 4.61E+00 | 4.02E-06 | 2.33E-05 |
| TAS2R5 | 4.61E+00 | 4.04E-06 | 2.34E-05 |
| C16orf11 | 4.61E+00 | 4.04E-06 | 2.34E-05 |
| TINAG | 4.61E+00 | 4.07E-06 | 2.35E-05 |
| EBNA1BP2 | 4.61E+00 | 4.08E-06 | 2.36E-05 |
| G6PC3 | 4.61E+00 | 4.08E-06 | 2.36E-05 |
| HCG18 | 4.61E+00 | 4.08E-06 | 2.36E-05 |
| PAK2 | 4.61E+00 | 4.08E-06 | 2.36E-05 |
| C19orf24 | 4.61E+00 | 4.09E-06 | 2.36E-05 |
| HOXA3 | 4.61E+00 | 4.10E-06 | 2.37E-05 |
| RAD21L1 | 4.61E+00 | 4.11E-06 | 2.37E-05 |
| STRBP | 4.61E+00 | 4.12E-06 | 2.38E-05 |
| LOC100133985 | 4.61E+00 | 4.12E-06 | 2.38E-05 |
| NKRF | 4.61E+00 | 4.13E-06 | 2.38E-05 |
| PSIMCT-1 | 4.60E+00 | 4.17E-06 | 2.41E-05 |
| FAM119B | 4.60E+00 | 4.20E-06 | 2.42E-05 |
| LOC91149 | 4.60E+00 | 4.22E-06 | 2.43E-05 |
| STC1 | 4.60E+00 | 4.23E-06 | 2.43E-05 |
| RPS6KB1 | 4.60E+00 | 4.23E-06 | 2.44E-05 |
| SCYL2 | 4.60E+00 | 4.27E-06 | 2.46E-05 |
| MAP3K7 | 4.60E+00 | 4.29E-06 | 2.47E-05 |
| CCDC120 | 4.60E+00 | 4.33E-06 | 2.49E-05 |
| MED12L | 4.59E+00 | 4.34E-06 | 2.50E-05 |
| KLHDC4 | 4.59E+00 | 4.38E-06 | 2.52E-05 |
| FOSL1 | 4.59E+00 | 4.40E-06 | 2.53E-05 |
| KARS | 4.59E+00 | 4.42E-06 | 2.54E-05 |
| LOC152024 | 4.59E+00 | 4.45E-06 | 2.56E-05 |
| LSM7 | 4.59E+00 | 4.49E-06 | 2.58E-05 |
| MRPL38 | 4.59E+00 | 4.50E-06 | 2.59E-05 |
| COL4A2 | 4.59E+00 | 4.51E-06 | 2.59E-05 |
| FAM71E2 | 4.59E+00 | 4.51E-06 | 2.59E-05 |
| AKR1B15 | 4.59E+00 | 4.54E-06 | 2.60E-05 |
| HSPA9 | 4.59E+00 | 4.54E-06 | 2.60E-05 |
| LDLRAD3 | 4.59E+00 | 4.55E-06 | 2.61E-05 |
| NKX2-2 | 4.59E+00 | 4.55E-06 | 2.61E-05 |
| TRIM37 | 4.58E+00 | 4.56E-06 | 2.61E-05 |
| EIF3D | 4.58E+00 | 4.58E-06 | 2.62E-05 |
| UMOD | 4.58E+00 | 4.59E-06 | 2.63E-05 |
| DDX47 | 4.58E+00 | 4.61E-06 | 2.64E-05 |
| EIF2AK2 | 4.58E+00 | 4.70E-06 | 2.69E-05 |
| CT45A4 | 4.58E+00 | 4.74E-06 | 2.71E-05 |
| SPRYD3 | 4.58E+00 | 4.75E-06 | 2.72E-05 |
| C7orf26 | 4.58E+00 | 4.75E-06 | 2.72E-05 |
| C11orf24 | 4.57E+00 | 4.83E-06 | 2.76E-05 |
| LRRC42 | 4.57E+00 | 4.83E-06 | 2.76E-05 |
| SGK196 | 4.57E+00 | 4.84E-06 | 2.76E-05 |
| MTPAP | 4.57E+00 | 4.85E-06 | 2.77E-05 |
| UBL4A | 4.57E+00 | 4.85E-06 | 2.77E-05 |
| FAM122B | 4.57E+00 | 4.88E-06 | 2.79E-05 |
| HSPA1B | 4.57E+00 | 4.93E-06 | 2.81E-05 |

| TAGLN3 | 4.57E+00 | 4.99E-06 | 2.85E-05 |
| --- | --- | --- | --- |
| IL17B | 4.56E+00 | 5.01E-06 | 2.86E-05 |
| AACSL | 4.56E+00 | 5.03E-06 | 2.87E-05 |
| CDK5RAP1 | 4.56E+00 | 5.03E-06 | 2.87E-05 |
| OR52A4 | 4.56E+00 | 5.04E-06 | 2.87E-05 |
| KRT80 | 4.56E+00 | 5.06E-06 | 2.88E-05 |
| POLL | 4.56E+00 | 5.09E-06 | 2.90E-05 |
| OR8G5 | 4.56E+00 | 5.12E-06 | 2.92E-05 |
| C12orf47 | 4.56E+00 | 5.14E-06 | 2.93E-05 |
| CDH10 | 4.56E+00 | 5.15E-06 | 2.93E-05 |
| TDRG1 | 4.56E+00 | 5.16E-06 | 2.93E-05 |
| GNA11 | 4.55E+00 | 5.36E-06 | 3.05E-05 |
| TSEN34 | 4.55E+00 | 5.40E-06 | 3.07E-05 |
| NKX2-5 | 4.55E+00 | 5.42E-06 | 3.08E-05 |
| DRG2 | 4.55E+00 | 5.47E-06 | 3.11E-05 |
| CTXN2 | 4.55E+00 | 5.50E-06 | 3.13E-05 |
| ZDHHC12 | 4.55E+00 | 5.50E-06 | 3.13E-05 |
| RCAN3 | 4.55E+00 | 5.51E-06 | 3.13E-05 |
| SYNGR4 | 4.54E+00 | 5.51E-06 | 3.13E-05 |
| AQP12B | 4.54E+00 | 5.51E-06 | 3.13E-05 |
| ARMC6 | 4.54E+00 | 5.53E-06 | 3.14E-05 |
| ACTL6B | 4.54E+00 | 5.56E-06 | 3.15E-05 |
| CNTNAP5 | 4.54E+00 | 5.56E-06 | 3.15E-05 |
| PABPC3 | 4.54E+00 | 5.56E-06 | 3.15E-05 |
| CHD5 | 4.54E+00 | 5.57E-06 | 3.16E-05 |
| DNTTIP1 | 4.54E+00 | 5.57E-06 | 3.16E-05 |
| DMRTB1 | 4.54E+00 | 5.57E-06 | 3.16E-05 |
| ZZEF1 | 4.54E+00 | 5.58E-06 | 3.16E-05 |
| TSKS | 4.54E+00 | 5.60E-06 | 3.17E-05 |
| LOXL3 | 4.54E+00 | 5.65E-06 | 3.20E-05 |
| CXorf61 | 4.54E+00 | 5.72E-06 | 3.23E-05 |
| BCAN | 4.54E+00 | 5.72E-06 | 3.24E-05 |
| IFT81 | 4.53E+00 | 5.80E-06 | 3.28E-05 |
| ZIC3 | 4.53E+00 | 5.86E-06 | 3.31E-05 |
| HM13 | 4.53E+00 | 5.87E-06 | 3.32E-05 |
| MEX3D | 4.53E+00 | 5.89E-06 | 3.33E-05 |
| CYB561D1 | 4.53E+00 | 5.92E-06 | 3.35E-05 |
| FBLL1 | 4.53E+00 | 5.96E-06 | 3.37E-05 |
| ST3GAL2 | 4.53E+00 | 6.04E-06 | 3.41E-05 |
| SREBF2 | 4.52E+00 | 6.11E-06 | 3.45E-05 |
| TMCC1 | 4.52E+00 | 6.15E-06 | 3.47E-05 |
| DPH1 | 4.52E+00 | 6.15E-06 | 3.47E-05 |
| PI4KAP1 | 4.52E+00 | 6.17E-06 | 3.48E-05 |
| PPIF | 4.52E+00 | 6.20E-06 | 3.49E-05 |
| FOXJ3 | 4.52E+00 | 6.20E-06 | 3.49E-05 |
| BARX1 | 4.52E+00 | 6.21E-06 | 3.50E-05 |
| LOC100240734 | 4.52E+00 | 6.30E-06 | 3.55E-05 |
| CTPS | 4.52E+00 | 6.32E-06 | 3.56E-05 |
| HAPLN1 | 4.52E+00 | 6.33E-06 | 3.57E-05 |
| LOC286359 | 4.52E+00 | 6.35E-06 | 3.57E-05 |
| FKBP9L | 4.51E+00 | 6.36E-06 | 3.58E-05 |
| KIAA0907 | 4.51E+00 | 6.37E-06 | 3.58E-05 |
| SCLT1 | 4.51E+00 | 6.39E-06 | 3.59E-05 |
| SPEM1 | 4.51E+00 | 6.41E-06 | 3.60E-05 |
| SCN5A | 4.51E+00 | 6.42E-06 | 3.61E-05 |
| LRIT1 | 4.51E+00 | 6.43E-06 | 3.61E-05 |
| AARSD1 | 4.51E+00 | 6.44E-06 | 3.62E-05 |
| PEX5 | 4.51E+00 | 6.49E-06 | 3.64E-05 |

| PRR3 |  | 4.51E+00 | 6.50E-06 | 3.65E-05 |
| --- | --- | --- | --- | --- |
| SFRS17A |  | 4.51E+00 | 6.50E-06 | 3.65E-05 |
| FERMT1 |  | 4.51E+00 | 6.51E-06 | 3.65E-05 |
| KCNN1 |  | 4.51E+00 | 6.51E-06 | 3.65E-05 |
| ZNF346 |  | 4.51E+00 | 6.52E-06 | 3.65E-05 |
| AMMECR1 |  | 4.51E+00 | 6.53E-06 | 3.66E-05 |
| PPM1H |  | 4.51E+00 | 6.55E-06 | 3.67E-05 |
| RBM33 |  | 4.51E+00 | 6.58E-06 | 3.68E-05 |
| LOC145783 |  | 4.51E+00 | 6.60E-06 | 3.70E-05 |
| TTL |  | 4.50E+00 | 6.67E-06 | 3.74E-05 |
| MED10 |  | 4.50E+00 | 6.69E-06 | 3.75E-05 |
| SEMA4D |  | 4.50E+00 | 6.73E-06 | 3.77E-05 |
| ADAM23 |  | 4.50E+00 | 6.81E-06 | 3.81E-05 |
| FTSJD2 |  | 4.50E+00 | 6.81E-06 | 3.81E-05 |
| PEX10 |  | 4.50E+00 | 6.87E-06 | 3.84E-05 |
| NOL8 |  | 4.50E+00 | 6.93E-06 | 3.88E-05 |
| MGAT5B |  | 4.49E+00 | 7.00E-06 | 3.91E-05 |
| CCDC144C |  | 4.49E+00 | 7.03E-06 | 3.93E-05 |
| FTMT |  | 4.49E+00 | 7.07E-06 | 3.95E-05 |
| ZNF107 |  | 4.49E+00 | 7.07E-06 | 3.95E-05 |
| OR4F29 |  | 4.49E+00 | 7.09E-06 | 3.96E-05 |
| OXGR1 |  | 4.49E+00 | 7.14E-06 | 3.98E-05 |
| POLR2B |  | 4.49E+00 | 7.17E-06 | 4.00E-05 |
| C9orf45 |  | 4.49E+00 | 7.24E-06 | 4.04E-05 |
|  | 5-Sep | 4.49E+00 | 7.26E-06 | 4.05E-05 |
| RIPPLY2 |  | 4.49E+00 | 7.31E-06 | 4.07E-05 |
| ST18 |  | 4.48E+00 | 7.42E-06 | 4.14E-05 |
| UGT8 |  | 4.48E+00 | 7.43E-06 | 4.14E-05 |
| LOC100133469 |  | 4.48E+00 | 7.44E-06 | 4.14E-05 |
| RFNG |  | 4.48E+00 | 7.51E-06 | 4.18E-05 |
| RREB1 |  | 4.48E+00 | 7.61E-06 | 4.24E-05 |
| GPATCH3 |  | 4.48E+00 | 7.65E-06 | 4.26E-05 |
| CYHR1 |  | 4.47E+00 | 7.69E-06 | 4.28E-05 |
| SRRM2 |  | 4.47E+00 | 7.72E-06 | 4.29E-05 |
| TADA2A |  | 4.47E+00 | 7.74E-06 | 4.31E-05 |
| SAPS3 |  | 4.47E+00 | 7.78E-06 | 4.33E-05 |
| GCLM |  | 4.47E+00 | 7.81E-06 | 4.34E-05 |
| MAN1B1 |  | 4.47E+00 | 7.83E-06 | 4.35E-05 |
| FOXE3 |  | 4.47E+00 | 7.84E-06 | 4.36E-05 |
| RNPEPL1 |  | 4.47E+00 | 7.85E-06 | 4.36E-05 |
| C1orf180 |  | 4.47E+00 | 7.89E-06 | 4.38E-05 |
| IL21R |  | 4.47E+00 | 7.95E-06 | 4.41E-05 |
| ARL14 |  | 4.47E+00 | 7.96E-06 | 4.41E-05 |
| NLRP4 |  | 4.47E+00 | 7.98E-06 | 4.43E-05 |
| CDH9 |  | 4.47E+00 | 7.99E-06 | 4.43E-05 |
| KLHDC7B |  | 4.47E+00 | 8.02E-06 | 4.44E-05 |
| PLCD4 |  | 4.46E+00 | 8.06E-06 | 4.47E-05 |
| C2CD2L |  | 4.46E+00 | 8.09E-06 | 4.48E-05 |
| LMTK3 |  | 4.46E+00 | 8.18E-06 | 4.53E-05 |
| SSSCA1 |  | 4.46E+00 | 8.24E-06 | 4.56E-05 |
| MTP18 |  | 4.46E+00 | 8.26E-06 | 4.57E-05 |
| NFKBID |  | 4.46E+00 | 8.29E-06 | 4.59E-05 |
| A2LD1 |  | -4.37E+00 | 1.24E-05 | 4.60E-05 |
| AADAC |  | -4.37E+00 | 1.24E-05 | 4.60E-05 |
| AADAT |  | -4.37E+00 | 1.24E-05 | 4.60E-05 |
| AASS |  | -4.37E+00 | 1.24E-05 | 4.60E-05 |
| ABAT |  | -4.37E+00 | 1.24E-05 | 4.60E-05 |
| ABCA10 |  | -4.37E+00 | 1.24E-05 | 4.60E-05 |

| ABCA5 | -4.37E+00 | 1.24E-05 | 4.60E-05 |
| --- | --- | --- | --- |
| ABCA6 | -4.37E+00 | 1.24E-05 | 4.60E-05 |
| ABCA8 | -4.37E+00 | 1.24E-05 | 4.60E-05 |
| ABCA9 | -4.37E+00 | 1.24E-05 | 4.60E-05 |
| ABCB10 | -4.37E+00 | 1.24E-05 | 4.60E-05 |
| ABCC6 | -4.37E+00 | 1.24E-05 | 4.60E-05 |
| ABCC6P1 | -4.37E+00 | 1.24E-05 | 4.60E-05 |
| ABCC6P2 | -4.37E+00 | 1.24E-05 | 4.60E-05 |
| ABCC9 | -4.37E+00 | 1.24E-05 | 4.60E-05 |
| ABCD4 | -4.37E+00 | 1.24E-05 | 4.60E-05 |
| ABHD13 | -4.37E+00 | 1.24E-05 | 4.60E-05 |
| ABHD14A | -4.37E+00 | 1.24E-05 | 4.60E-05 |
| ABHD14B | -4.37E+00 | 1.24E-05 | 4.60E-05 |
| ABHD5 | -4.37E+00 | 1.24E-05 | 4.60E-05 |
| ABHD6 | -4.37E+00 | 1.24E-05 | 4.60E-05 |
| ABO | -4.37E+00 | 1.24E-05 | 4.60E-05 |
| ACAA1 | -4.37E+00 | 1.24E-05 | 4.60E-05 |
| ACAA2 | -4.37E+00 | 1.24E-05 | 4.60E-05 |
| ACAD8 | -4.37E+00 | 1.24E-05 | 4.60E-05 |
| ACADL | -4.37E+00 | 1.24E-05 | 4.60E-05 |
| ACADM | -4.37E+00 | 1.24E-05 | 4.60E-05 |
| ACADSB | -4.37E+00 | 1.24E-05 | 4.60E-05 |
| ACAT1 | -4.37E+00 | 1.24E-05 | 4.60E-05 |
| ACOT1 | -4.37E+00 | 1.24E-05 | 4.60E-05 |
| ACOT13 | -4.37E+00 | 1.24E-05 | 4.60E-05 |
| ACOT2 | -4.37E+00 | 1.24E-05 | 4.60E-05 |
| ACOT4 | -4.37E+00 | 1.24E-05 | 4.60E-05 |
| ACOX1 | -4.37E+00 | 1.24E-05 | 4.60E-05 |
| ACOX2 | -4.37E+00 | 1.24E-05 | 4.60E-05 |
| ACSF2 | -4.37E+00 | 1.24E-05 | 4.60E-05 |
| ACSL1 | -4.37E+00 | 1.24E-05 | 4.60E-05 |
| ACSL5 | -4.37E+00 | 1.24E-05 | 4.60E-05 |
| ACSL6 | -4.37E+00 | 1.24E-05 | 4.60E-05 |
| ACSM1 | -4.37E+00 | 1.24E-05 | 4.60E-05 |
| ACSM5 | -4.37E+00 | 1.24E-05 | 4.60E-05 |
| ACTR10 | -4.37E+00 | 1.24E-05 | 4.60E-05 |
| ACTR1B | -4.37E+00 | 1.24E-05 | 4.60E-05 |
| ACTR3C | -4.37E+00 | 1.24E-05 | 4.60E-05 |
| ACVR2A | -4.37E+00 | 1.24E-05 | 4.60E-05 |
| ACYP2 | -4.37E+00 | 1.24E-05 | 4.60E-05 |
| ADAL | -4.37E+00 | 1.24E-05 | 4.60E-05 |
| ADAMTS17 | -4.37E+00 | 1.24E-05 | 4.60E-05 |
| ADAMTS8 | -4.37E+00 | 1.24E-05 | 4.60E-05 |
| ADAMTSL3 | -4.37E+00 | 1.24E-05 | 4.60E-05 |
| ADCY9 | -4.37E+00 | 1.24E-05 | 4.60E-05 |
| ADH1A | -4.37E+00 | 1.24E-05 | 4.60E-05 |
| ADH1B | -4.37E+00 | 1.24E-05 | 4.60E-05 |
| ADH1C | -4.37E+00 | 1.24E-05 | 4.60E-05 |
| ADH5 | -4.37E+00 | 1.24E-05 | 4.60E-05 |
| ADH7 | -4.37E+00 | 1.24E-05 | 4.60E-05 |
| ADHFE1 | -4.37E+00 | 1.24E-05 | 4.60E-05 |
| ADI1 | -4.37E+00 | 1.24E-05 | 4.60E-05 |
| ADIPOR1 | -4.37E+00 | 1.24E-05 | 4.60E-05 |
| ADK | -4.37E+00 | 1.24E-05 | 4.60E-05 |
| ADRA1A | -4.37E+00 | 1.24E-05 | 4.60E-05 |
| ADRA1B | -4.37E+00 | 1.24E-05 | 4.60E-05 |
| ADRB1 | -4.37E+00 | 1.24E-05 | 4.60E-05 |
| ADRB2 | -4.37E+00 | 1.24E-05 | 4.60E-05 |

| AG2 | -4.37E+00 | 1.24E-05 | 4.60E-05 |
| --- | --- | --- | --- |
| AGAP11 | -4.37E+00 | 1.24E-05 | 4.60E-05 |
| AGRP | -4.37E+00 | 1.24E-05 | 4.60E-05 |
| AGXT2L2 | -4.37E+00 | 1.24E-05 | 4.60E-05 |
| AHCTF1 | 4.43E+00 | 9.62E-06 | 4.60E-05 |
| AHCYL2 | -4.37E+00 | 1.24E-05 | 4.60E-05 |
| AIF1L | -4.37E+00 | 1.24E-05 | 4.60E-05 |
| AIG1 | -4.37E+00 | 1.24E-05 | 4.60E-05 |
| AK1 | -4.37E+00 | 1.24E-05 | 4.60E-05 |
| AK3 | -4.37E+00 | 1.24E-05 | 4.60E-05 |
| AKAP13 | -4.37E+00 | 1.24E-05 | 4.60E-05 |
| AKAP6 | -4.37E+00 | 1.24E-05 | 4.60E-05 |
| AKR1A1 | -4.37E+00 | 1.24E-05 | 4.60E-05 |
| AKR7A2 | -4.37E+00 | 1.24E-05 | 4.60E-05 |
| AKTIP | -4.37E+00 | 1.24E-05 | 4.60E-05 |
| ALAD | -4.37E+00 | 1.24E-05 | 4.60E-05 |
| ALDH2 | -4.37E+00 | 1.24E-05 | 4.60E-05 |
| ALDH3A2 | -4.37E+00 | 1.24E-05 | 4.60E-05 |
| ALDH5A1 | -4.37E+00 | 1.24E-05 | 4.60E-05 |
| ALDH6A1 | -4.37E+00 | 1.24E-05 | 4.60E-05 |
| ALDH7A1 | -4.37E+00 | 1.24E-05 | 4.60E-05 |
| ALDH9A1 | -4.37E+00 | 1.24E-05 | 4.60E-05 |
| ALDOB | -4.37E+00 | 1.24E-05 | 4.60E-05 |
| ALG13 | -4.37E+00 | 1.24E-05 | 4.60E-05 |
| ALG14 | -4.37E+00 | 1.24E-05 | 4.60E-05 |
| ALKBH1 | -4.37E+00 | 1.24E-05 | 4.60E-05 |
| ALOX15 | -4.37E+00 | 1.24E-05 | 4.60E-05 |
| ALPL | -4.37E+00 | 1.24E-05 | 4.60E-05 |
| ALS2CR8 | -4.37E+00 | 1.24E-05 | 4.60E-05 |
| AMACR | -4.37E+00 | 1.24E-05 | 4.60E-05 |
| AMY1A | -4.37E+00 | 1.24E-05 | 4.60E-05 |
| AMY2A | -4.37E+00 | 1.24E-05 | 4.60E-05 |
| AMY2B | -4.37E+00 | 1.24E-05 | 4.60E-05 |
| ANAPC10 | -4.37E+00 | 1.24E-05 | 4.60E-05 |
| ANAPC13 | -4.37E+00 | 1.24E-05 | 4.60E-05 |
| ANAPC16 | -4.37E+00 | 1.24E-05 | 4.60E-05 |
| ANG | -4.37E+00 | 1.24E-05 | 4.60E-05 |
| ANGPT4 | -4.37E+00 | 1.24E-05 | 4.60E-05 |
| ANGPTL1 | -4.37E+00 | 1.24E-05 | 4.60E-05 |
| ANGPTL7 | -4.37E+00 | 1.24E-05 | 4.60E-05 |
| ANKRA2 | -4.37E+00 | 1.24E-05 | 4.60E-05 |
| ANKRD29 | -4.37E+00 | 1.24E-05 | 4.60E-05 |
| ANKRD50 | -4.37E+00 | 1.24E-05 | 4.60E-05 |
| ANXA7 | -4.37E+00 | 1.24E-05 | 4.60E-05 |
| AOC3 | -4.37E+00 | 1.24E-05 | 4.60E-05 |
| AP3S2 | -4.37E+00 | 1.24E-05 | 4.60E-05 |
| AP4S1 | -4.37E+00 | 1.24E-05 | 4.60E-05 |
| APBA1 | -4.37E+00 | 1.24E-05 | 4.60E-05 |
| API5 | -4.37E+00 | 1.24E-05 | 4.60E-05 |
| APOC1 | -4.37E+00 | 1.24E-05 | 4.60E-05 |
| APOC2 | -4.37E+00 | 1.24E-05 | 4.60E-05 |
| APOH | -4.37E+00 | 1.24E-05 | 4.60E-05 |
| AQP1 | -4.37E+00 | 1.24E-05 | 4.60E-05 |
| AQP4 | -4.37E+00 | 1.24E-05 | 4.60E-05 |
| AQP7 | -4.37E+00 | 1.24E-05 | 4.60E-05 |
| AQP8 | -4.37E+00 | 1.24E-05 | 4.60E-05 |
| AR | -4.37E+00 | 1.24E-05 | 4.60E-05 |
| ARFIP1 | -4.37E+00 | 1.24E-05 | 4.60E-05 |

| ARFRP1 | 4.45E+00 | 8.54E-06 | 4.60E-05 |
| --- | --- | --- | --- |
| ARHGAP24 | -4.37E+00 | 1.24E-05 | 4.60E-05 |
| ARHGAP29 | -4.37E+00 | 1.24E-05 | 4.60E-05 |
| ARHGAP42 | -4.37E+00 | 1.24E-05 | 4.60E-05 |
| ARHGAP6 | -4.37E+00 | 1.24E-05 | 4.60E-05 |
| ARHGEF4 | 4.44E+00 | 8.97E-06 | 4.60E-05 |
| ARHGEF6 | -4.37E+00 | 1.24E-05 | 4.60E-05 |
| ARID4A | -4.37E+00 | 1.24E-05 | 4.60E-05 |
| ARL15 | -4.37E+00 | 1.24E-05 | 4.60E-05 |
| ARL2BP | -4.37E+00 | 1.24E-05 | 4.60E-05 |
| ARL6IP5 | -4.37E+00 | 1.24E-05 | 4.60E-05 |
| ARRDC4 | -4.37E+00 | 1.24E-05 | 4.60E-05 |
| ARSD | -4.37E+00 | 1.24E-05 | 4.60E-05 |
| ARSK | -4.37E+00 | 1.24E-05 | 4.60E-05 |
| ART4 | -4.37E+00 | 1.24E-05 | 4.60E-05 |
| ARV1 | -4.37E+00 | 1.24E-05 | 4.60E-05 |
| ASNSD1 | -4.37E+00 | 1.24E-05 | 4.60E-05 |
| ASPA | -4.37E+00 | 1.24E-05 | 4.60E-05 |
| ASPN | -4.37E+00 | 1.24E-05 | 4.60E-05 |
| ASS1 | -4.37E+00 | 1.24E-05 | 4.60E-05 |
| ATAD1 | -4.37E+00 | 1.24E-05 | 4.60E-05 |
| ATF7IP2 | -4.37E+00 | 1.24E-05 | 4.60E-05 |
| ATG4A | -4.37E+00 | 1.24E-05 | 4.60E-05 |
| ATL1 | -4.37E+00 | 1.24E-05 | 4.60E-05 |
| ATL2 | -4.37E+00 | 1.24E-05 | 4.60E-05 |
| ATOH8 | -4.37E+00 | 1.24E-05 | 4.60E-05 |
| ATP5J | -4.37E+00 | 1.24E-05 | 4.60E-05 |
| ATP5O | -4.37E+00 | 1.24E-05 | 4.60E-05 |
| ATP5S | -4.37E+00 | 1.24E-05 | 4.60E-05 |
| ATP6V1D | -4.37E+00 | 1.24E-05 | 4.60E-05 |
| ATP8B4 | -4.37E+00 | 1.24E-05 | 4.60E-05 |
| ATPAF1 | -4.37E+00 | 1.24E-05 | 4.60E-05 |
| ATPIF1 | -4.37E+00 | 1.24E-05 | 4.60E-05 |
| ATXN3 | -4.37E+00 | 1.24E-05 | 4.60E-05 |
| AUH | -4.37E+00 | 1.24E-05 | 4.60E-05 |
| AUTS2 | -4.37E+00 | 1.24E-05 | 4.60E-05 |
| B2M | -4.37E+00 | 1.24E-05 | 4.60E-05 |
| B3GNT2 | -4.37E+00 | 1.24E-05 | 4.60E-05 |
| BAAT | -4.37E+00 | 1.24E-05 | 4.60E-05 |
| BACE1 | -4.37E+00 | 1.24E-05 | 4.60E-05 |
| BAG2 | 4.39E+00 | 1.12E-05 | 4.60E-05 |
| BAIAP2 | -4.37E+00 | 1.24E-05 | 4.60E-05 |
| BBS1 | -4.37E+00 | 1.24E-05 | 4.60E-05 |
| BCAP29 | -4.37E+00 | 1.24E-05 | 4.60E-05 |
| BCKDHB | -4.37E+00 | 1.24E-05 | 4.60E-05 |
| BDH2 | -4.37E+00 | 1.24E-05 | 4.60E-05 |
| BDNFOS | -4.37E+00 | 1.24E-05 | 4.60E-05 |
| BHLHE22 | -4.37E+00 | 1.24E-05 | 4.60E-05 |
| BIVM | -4.37E+00 | 1.24E-05 | 4.60E-05 |
| BLNK | -4.37E+00 | 1.24E-05 | 4.60E-05 |
| BLOC1S1 | -4.37E+00 | 1.24E-05 | 4.60E-05 |
| BMPER | -4.37E+00 | 1.24E-05 | 4.60E-05 |
| BMPR2 | -4.37E+00 | 1.24E-05 | 4.60E-05 |
| BMX | -4.37E+00 | 1.24E-05 | 4.60E-05 |
| BOK | -4.37E+00 | 1.24E-05 | 4.60E-05 |
| BREA2 | 4.43E+00 | 9.25E-06 | 4.60E-05 |
| BRP44L | -4.37E+00 | 1.24E-05 | 4.60E-05 |
| BRWD1 | -4.37E+00 | 1.24E-05 | 4.60E-05 |

| BTD | -4.37E+00 | 1.24E-05 | 4.60E-05 |
| --- | --- | --- | --- |
| BTN3A1 | -4.37E+00 | 1.24E-05 | 4.60E-05 |
| BTN3A3 | -4.37E+00 | 1.24E-05 | 4.60E-05 |
| BTNL9 | -4.37E+00 | 1.24E-05 | 4.60E-05 |
| C10orf105 | -4.37E+00 | 1.24E-05 | 4.60E-05 |
| C10orf107 | -4.37E+00 | 1.24E-05 | 4.60E-05 |
| C10orf11 | -4.37E+00 | 1.24E-05 | 4.60E-05 |
| C10orf116 | -4.37E+00 | 1.24E-05 | 4.60E-05 |
| C10orf128 | -4.37E+00 | 1.24E-05 | 4.60E-05 |
| C10orf26 | -4.37E+00 | 1.24E-05 | 4.60E-05 |
| C10orf32 | -4.37E+00 | 1.24E-05 | 4.60E-05 |
| C10orf46 | 4.38E+00 | 1.17E-05 | 4.60E-05 |
| C10orf72 | -4.37E+00 | 1.24E-05 | 4.60E-05 |
| C10orf88 | 4.43E+00 | 9.56E-06 | 4.60E-05 |
| C11orf1 | -4.37E+00 | 1.24E-05 | 4.60E-05 |
| C11orf46 | -4.37E+00 | 1.24E-05 | 4.60E-05 |
| C11orf54 | -4.37E+00 | 1.24E-05 | 4.60E-05 |
| C11orf66 | -4.37E+00 | 1.24E-05 | 4.60E-05 |
| C11orf67 | -4.37E+00 | 1.24E-05 | 4.60E-05 |
| C12orf60 | -4.37E+00 | 1.24E-05 | 4.60E-05 |
| C12orf69 | -4.37E+00 | 1.24E-05 | 4.60E-05 |
| C12orf72 | -4.37E+00 | 1.24E-05 | 4.60E-05 |
| C13orf15 | -4.37E+00 | 1.24E-05 | 4.60E-05 |
| C14orf1 | -4.37E+00 | 1.24E-05 | 4.60E-05 |
| C14orf138 | -4.37E+00 | 1.24E-05 | 4.60E-05 |
| C14orf142 | -4.37E+00 | 1.24E-05 | 4.60E-05 |
| C14orf159 | -4.37E+00 | 1.24E-05 | 4.60E-05 |
| C14orf167 | -4.37E+00 | 1.24E-05 | 4.60E-05 |
| C14orf179 | -4.37E+00 | 1.24E-05 | 4.60E-05 |
| C14orf180 | -4.37E+00 | 1.24E-05 | 4.60E-05 |
| C14orf28 | -4.37E+00 | 1.24E-05 | 4.60E-05 |
| C14orf45 | -4.37E+00 | 1.24E-05 | 4.60E-05 |
| C15orf24 | -4.37E+00 | 1.24E-05 | 4.60E-05 |
| C15orf29 | -4.37E+00 | 1.24E-05 | 4.60E-05 |
| C15orf33 | -4.37E+00 | 1.24E-05 | 4.60E-05 |
| C16orf52 | -4.37E+00 | 1.24E-05 | 4.60E-05 |
| C16orf62 | -4.37E+00 | 1.24E-05 | 4.60E-05 |
| C16orf71 | -4.37E+00 | 1.24E-05 | 4.60E-05 |
| C17orf108 | -4.37E+00 | 1.24E-05 | 4.60E-05 |
| C17orf44 | -4.37E+00 | 1.24E-05 | 4.60E-05 |
| C17orf48 | -4.37E+00 | 1.24E-05 | 4.60E-05 |
| C17orf61 | -4.37E+00 | 1.24E-05 | 4.60E-05 |
| C17orf91 | -4.37E+00 | 1.24E-05 | 4.60E-05 |
| C18orf1 | -4.37E+00 | 1.24E-05 | 4.60E-05 |
| C18orf18 | -4.37E+00 | 1.24E-05 | 4.60E-05 |
| C18orf32 | -4.37E+00 | 1.24E-05 | 4.60E-05 |
| C19orf30 | 4.38E+00 | 1.17E-05 | 4.60E-05 |
| C19orf42 | -4.37E+00 | 1.24E-05 | 4.60E-05 |
| C1D | -4.37E+00 | 1.24E-05 | 4.60E-05 |
| C1GALT1C1 | -4.37E+00 | 1.24E-05 | 4.60E-05 |
| C1orf101 | -4.37E+00 | 1.24E-05 | 4.60E-05 |
| C1orf107 | 4.44E+00 | 8.86E-06 | 4.60E-05 |
| C1orf168 | -4.37E+00 | 1.24E-05 | 4.60E-05 |
| C1orf21 | -4.37E+00 | 1.24E-05 | 4.60E-05 |
| C1orf212 | -4.37E+00 | 1.24E-05 | 4.60E-05 |
| C1orf25 | -4.37E+00 | 1.24E-05 | 4.60E-05 |
| C1orf27 | -4.37E+00 | 1.24E-05 | 4.60E-05 |

| C1orf53 | -4.37E+00 | 1.24E-05 | 4.60E-05 |
| --- | --- | --- | --- |
| C20orf108 | -4.37E+00 | 1.24E-05 | 4.60E-05 |
| C20orf132 | -4.37E+00 | 1.24E-05 | 4.60E-05 |
| C20orf56 | -4.37E+00 | 1.24E-05 | 4.60E-05 |
| C21orf33 | -4.37E+00 | 1.24E-05 | 4.60E-05 |
| C21orf34 | -4.37E+00 | 1.24E-05 | 4.60E-05 |
| C21orf63 | -4.37E+00 | 1.24E-05 | 4.60E-05 |
| C21orf7 | -4.37E+00 | 1.24E-05 | 4.60E-05 |
| C22orf13 | -4.37E+00 | 1.24E-05 | 4.60E-05 |
| C22orf32 | -4.37E+00 | 1.24E-05 | 4.60E-05 |
| C22orf33 | -4.37E+00 | 1.24E-05 | 4.60E-05 |
| C2orf40 | -4.37E+00 | 1.24E-05 | 4.60E-05 |
| C2orf55 | -4.37E+00 | 1.24E-05 | 4.60E-05 |
| C2orf58 | -4.37E+00 | 1.24E-05 | 4.60E-05 |
| C2orf64 | -4.37E+00 | 1.24E-05 | 4.60E-05 |
| C2orf67 | -4.37E+00 | 1.24E-05 | 4.60E-05 |
| C2orf7 | -4.37E+00 | 1.24E-05 | 4.60E-05 |
| C2orf74 | -4.37E+00 | 1.24E-05 | 4.60E-05 |
| C2orf86 | -4.37E+00 | 1.24E-05 | 4.60E-05 |
| C3 | -4.37E+00 | 1.24E-05 | 4.60E-05 |
| C3orf23 | -4.37E+00 | 1.24E-05 | 4.60E-05 |
| C3orf58 | -4.37E+00 | 1.24E-05 | 4.60E-05 |
| C4A | -4.37E+00 | 1.24E-05 | 4.60E-05 |
| C4BPA | -4.37E+00 | 1.24E-05 | 4.60E-05 |
| C4orf19 | -4.37E+00 | 1.24E-05 | 4.60E-05 |
| C4orf3 | -4.37E+00 | 1.24E-05 | 4.60E-05 |
| C4orf33 | -4.37E+00 | 1.24E-05 | 4.60E-05 |
| C4orf34 | -4.37E+00 | 1.24E-05 | 4.60E-05 |
| C5orf23 | -4.37E+00 | 1.24E-05 | 4.60E-05 |
| C5orf35 | -4.37E+00 | 1.24E-05 | 4.60E-05 |
| C5orf36 | -4.37E+00 | 1.24E-05 | 4.60E-05 |
| C5orf4 | -4.37E+00 | 1.24E-05 | 4.60E-05 |
| C5orf41 | -4.37E+00 | 1.24E-05 | 4.60E-05 |
| C5orf44 | -4.37E+00 | 1.24E-05 | 4.60E-05 |
| C5orf53 | -4.37E+00 | 1.24E-05 | 4.60E-05 |
| C6 | -4.37E+00 | 1.24E-05 | 4.60E-05 |
| C6orf123 | -4.37E+00 | 1.24E-05 | 4.60E-05 |
| C6orf138 | -4.37E+00 | 1.24E-05 | 4.60E-05 |
| C6orf145 | -4.37E+00 | 1.24E-05 | 4.60E-05 |
| C6orf203 | -4.37E+00 | 1.24E-05 | 4.60E-05 |
| C6orf217 | -4.37E+00 | 1.24E-05 | 4.60E-05 |
| C6orf41 | -4.37E+00 | 1.24E-05 | 4.60E-05 |
| C6orf70 | -4.37E+00 | 1.24E-05 | 4.60E-05 |
| C6orf72 | -4.37E+00 | 1.24E-05 | 4.60E-05 |
| C6orf97 | -4.37E+00 | 1.24E-05 | 4.60E-05 |
| C7 | -4.37E+00 | 1.24E-05 | 4.60E-05 |
| C7orf41 | -4.37E+00 | 1.24E-05 | 4.60E-05 |
| C7orf58 | -4.37E+00 | 1.24E-05 | 4.60E-05 |
| C8B | -4.37E+00 | 1.24E-05 | 4.60E-05 |
| C8orf4 | -4.37E+00 | 1.24E-05 | 4.60E-05 |
| C8orf40 | -4.37E+00 | 1.24E-05 | 4.60E-05 |
| C8orf46 | -4.37E+00 | 1.24E-05 | 4.60E-05 |
| C8orf79 | -4.37E+00 | 1.24E-05 | 4.60E-05 |
| C8orf83 | -4.37E+00 | 1.24E-05 | 4.60E-05 |
| C9orf103 | -4.37E+00 | 1.24E-05 | 4.60E-05 |
| C9orf142 | 4.38E+00 | 1.17E-05 | 4.60E-05 |
| C9orf150 | -4.37E+00 | 1.24E-05 | 4.60E-05 |

| C9orf44 | -4.37E+00 | 1.24E-05 | 4.60E-05 |
| --- | --- | --- | --- |
| C9orf46 | -4.37E+00 | 1.24E-05 | 4.60E-05 |
| C9orf5 | -4.37E+00 | 1.24E-05 | 4.60E-05 |
| C9orf72 | -4.37E+00 | 1.24E-05 | 4.60E-05 |
| C9orf95 | -4.37E+00 | 1.24E-05 | 4.60E-05 |
| CA10 | -4.37E+00 | 1.24E-05 | 4.60E-05 |
| CA2 | -4.37E+00 | 1.24E-05 | 4.60E-05 |
| CA4 | -4.37E+00 | 1.24E-05 | 4.60E-05 |
| CADM1 | -4.37E+00 | 1.24E-05 | 4.60E-05 |
| CALCB | 4.45E+00 | 8.80E-06 | 4.60E-05 |
| CALCOCO1 | -4.37E+00 | 1.24E-05 | 4.60E-05 |
| CALCOCO2 | -4.37E+00 | 1.24E-05 | 4.60E-05 |
| CALCRL | -4.37E+00 | 1.24E-05 | 4.60E-05 |
| CALM1 | -4.37E+00 | 1.24E-05 | 4.60E-05 |
| CALML4 | -4.37E+00 | 1.24E-05 | 4.60E-05 |
| CAMK2D | -4.37E+00 | 1.24E-05 | 4.60E-05 |
| CAMTA1 | -4.37E+00 | 1.24E-05 | 4.60E-05 |
| CAPN3 | -4.37E+00 | 1.24E-05 | 4.60E-05 |
| CASC4 | -4.37E+00 | 1.24E-05 | 4.60E-05 |
| CASP12 | -4.37E+00 | 1.24E-05 | 4.60E-05 |
| CASQ2 | -4.37E+00 | 1.24E-05 | 4.60E-05 |
| CAST | -4.37E+00 | 1.24E-05 | 4.60E-05 |
| CAT | -4.37E+00 | 1.24E-05 | 4.60E-05 |
| CAV2 | -4.37E+00 | 1.24E-05 | 4.60E-05 |
| CBARA1 | -4.37E+00 | 1.24E-05 | 4.60E-05 |
| CBR4 | -4.37E+00 | 1.24E-05 | 4.60E-05 |
| CBX7 | -4.37E+00 | 1.24E-05 | 4.60E-05 |
| CC2D2B | -4.37E+00 | 1.24E-05 | 4.60E-05 |
| CCBL2 | -4.37E+00 | 1.24E-05 | 4.60E-05 |
| CCDC111 | -4.37E+00 | 1.24E-05 | 4.60E-05 |
| CCDC121 | -4.37E+00 | 1.24E-05 | 4.60E-05 |
| CCDC126 | -4.37E+00 | 1.24E-05 | 4.60E-05 |
| CCDC134 | 4.45E+00 | 8.68E-06 | 4.60E-05 |
| CCDC146 | -4.37E+00 | 1.24E-05 | 4.60E-05 |
| CCDC147 | -4.37E+00 | 1.24E-05 | 4.60E-05 |
| CCDC152 | -4.37E+00 | 1.24E-05 | 4.60E-05 |
| CCDC159 | -4.37E+00 | 1.24E-05 | 4.60E-05 |
| CCDC25 | -4.37E+00 | 1.24E-05 | 4.60E-05 |
| CCDC28A | -4.37E+00 | 1.24E-05 | 4.60E-05 |
| CCDC3 | -4.37E+00 | 1.24E-05 | 4.60E-05 |
| CCDC48 | -4.37E+00 | 1.24E-05 | 4.60E-05 |
| CCDC52 | 4.40E+00 | 1.06E-05 | 4.60E-05 |
| CCDC53 | -4.37E+00 | 1.24E-05 | 4.60E-05 |
| CCDC85A | -4.37E+00 | 1.24E-05 | 4.60E-05 |
| CCDC85B | 4.37E+00 | 1.22E-05 | 4.60E-05 |
| CCDC88B | 4.42E+00 | 9.70E-06 | 4.60E-05 |
| CCL14 | -4.37E+00 | 1.24E-05 | 4.60E-05 |
| CCL16 | -4.37E+00 | 1.24E-05 | 4.60E-05 |
| CCL23 | -4.37E+00 | 1.24E-05 | 4.60E-05 |
| CCNDBP1 | -4.37E+00 | 1.24E-05 | 4.60E-05 |
| CCNG1 | -4.37E+00 | 1.24E-05 | 4.60E-05 |
| CCNH | -4.37E+00 | 1.24E-05 | 4.60E-05 |
| CCPG1 | -4.37E+00 | 1.24E-05 | 4.60E-05 |
| CCT6B | -4.37E+00 | 1.24E-05 | 4.60E-05 |
| CD1D | -4.37E+00 | 1.24E-05 | 4.60E-05 |
| CD300LG | -4.37E+00 | 1.24E-05 | 4.60E-05 |
| CD302 | -4.37E+00 | 1.24E-05 | 4.60E-05 |

| CD55 | -4.37E+00 | 1.24E-05 | 4.60E-05 |
| --- | --- | --- | --- |
| CD59 | -4.37E+00 | 1.24E-05 | 4.60E-05 |
| CD5L | -4.37E+00 | 1.24E-05 | 4.60E-05 |
| CD81 | -4.37E+00 | 1.24E-05 | 4.60E-05 |
| CD9 | -4.37E+00 | 1.24E-05 | 4.60E-05 |
| CDADC1 | -4.37E+00 | 1.24E-05 | 4.60E-05 |
| CDC37L1 | -4.37E+00 | 1.24E-05 | 4.60E-05 |
| CDC42EP3 | -4.37E+00 | 1.24E-05 | 4.60E-05 |
| CDH23 | -4.37E+00 | 1.24E-05 | 4.60E-05 |
| CDKN1B | -4.37E+00 | 1.24E-05 | 4.60E-05 |
| CDKN2A | 4.40E+00 | 1.10E-05 | 4.60E-05 |
| CDKN2AIP | -4.37E+00 | 1.24E-05 | 4.60E-05 |
| CDNF | -4.37E+00 | 1.24E-05 | 4.60E-05 |
| CDO1 | -4.37E+00 | 1.24E-05 | 4.60E-05 |
| CEACAM1 | -4.37E+00 | 1.24E-05 | 4.60E-05 |
| CECR6 | 4.42E+00 | 1.01E-05 | 4.60E-05 |
| CENPC1 | -4.37E+00 | 1.24E-05 | 4.60E-05 |
| CEP120 | -4.37E+00 | 1.24E-05 | 4.60E-05 |
| CEP57 | -4.37E+00 | 1.24E-05 | 4.60E-05 |
| CEP70 | -4.37E+00 | 1.24E-05 | 4.60E-05 |
| CEPT1 | -4.37E+00 | 1.24E-05 | 4.60E-05 |
| CES1 | -4.37E+00 | 1.24E-05 | 4.60E-05 |
| CES2 | -4.37E+00 | 1.24E-05 | 4.60E-05 |
| CFH | -4.37E+00 | 1.24E-05 | 4.60E-05 |
| CFI | -4.37E+00 | 1.24E-05 | 4.60E-05 |
| CGNL1 | -4.37E+00 | 1.24E-05 | 4.60E-05 |
| CGRRF1 | -4.37E+00 | 1.24E-05 | 4.60E-05 |
| CHADL | -4.37E+00 | 1.24E-05 | 4.60E-05 |
| CHD9 | -4.37E+00 | 1.24E-05 | 4.60E-05 |
| CHIC2 | -4.37E+00 | 1.24E-05 | 4.60E-05 |
| CHN2 | -4.37E+00 | 1.24E-05 | 4.60E-05 |
| CHP | -4.37E+00 | 1.24E-05 | 4.60E-05 |
| CHPT1 | -4.37E+00 | 1.24E-05 | 4.60E-05 |
| CHRM2 | -4.37E+00 | 1.24E-05 | 4.60E-05 |
| CHST7 | -4.37E+00 | 1.24E-05 | 4.60E-05 |
| CHST9 | -4.37E+00 | 1.24E-05 | 4.60E-05 |
| CHURC1 | -4.37E+00 | 1.24E-05 | 4.60E-05 |
| CIR1 | -4.37E+00 | 1.24E-05 | 4.60E-05 |
| CIRBP | -4.37E+00 | 1.24E-05 | 4.60E-05 |
| CISH | -4.37E+00 | 1.24E-05 | 4.60E-05 |
| CKMT2 | -4.37E+00 | 1.24E-05 | 4.60E-05 |
| CLDN23 | -4.37E+00 | 1.24E-05 | 4.60E-05 |
| CLEC14A | -4.37E+00 | 1.24E-05 | 4.60E-05 |
| CLEC1A | -4.37E+00 | 1.24E-05 | 4.60E-05 |
| CLEC3B | -4.37E+00 | 1.24E-05 | 4.60E-05 |
| CLIC5 | -4.37E+00 | 1.24E-05 | 4.60E-05 |
| CLK1 | -4.37E+00 | 1.24E-05 | 4.60E-05 |
| CLK4 | -4.37E+00 | 1.24E-05 | 4.60E-05 |
| CLN5 | -4.37E+00 | 1.24E-05 | 4.60E-05 |
| CLU | -4.37E+00 | 1.24E-05 | 4.60E-05 |
| CLYBL | -4.37E+00 | 1.24E-05 | 4.60E-05 |
| CMAH | -4.37E+00 | 1.24E-05 | 4.60E-05 |
| CMPK1 | -4.37E+00 | 1.24E-05 | 4.60E-05 |
| CNGA1 | -4.37E+00 | 1.24E-05 | 4.60E-05 |
| CNIH | -4.37E+00 | 1.24E-05 | 4.60E-05 |
| CNOT6L | -4.37E+00 | 1.24E-05 | 4.60E-05 |
| CNRIP1 | -4.37E+00 | 1.24E-05 | 4.60E-05 |

| CNTN3 | -4.37E+00 | 1.24E-05 | 4.60E-05 |
| --- | --- | --- | --- |
| CNTN4 | -4.37E+00 | 1.24E-05 | 4.60E-05 |
| COBLL1 | -4.37E+00 | 1.24E-05 | 4.60E-05 |
| COG1 | 4.43E+00 | 9.60E-06 | 4.60E-05 |
| COL4A3 | -4.37E+00 | 1.24E-05 | 4.60E-05 |
| COL4A4 | -4.37E+00 | 1.24E-05 | 4.60E-05 |
| COMMD1 | -4.37E+00 | 1.24E-05 | 4.60E-05 |
| COPS2 | -4.37E+00 | 1.24E-05 | 4.60E-05 |
| COPZ2 | -4.37E+00 | 1.24E-05 | 4.60E-05 |
| COQ10B | -4.37E+00 | 1.24E-05 | 4.60E-05 |
| COQ4 | -4.37E+00 | 1.24E-05 | 4.60E-05 |
| COQ6 | -4.37E+00 | 1.24E-05 | 4.60E-05 |
| COQ7 | -4.37E+00 | 1.24E-05 | 4.60E-05 |
| COX16 | -4.37E+00 | 1.24E-05 | 4.60E-05 |
| COX18 | -4.37E+00 | 1.24E-05 | 4.60E-05 |
| COX7A1 | -4.37E+00 | 1.24E-05 | 4.60E-05 |
| COX7B | -4.37E+00 | 1.24E-05 | 4.60E-05 |
| CPB2 | -4.37E+00 | 1.24E-05 | 4.60E-05 |
| CPEB2 | -4.37E+00 | 1.24E-05 | 4.60E-05 |
| CPEB3 | -4.37E+00 | 1.24E-05 | 4.60E-05 |
| CPEB4 | -4.37E+00 | 1.24E-05 | 4.60E-05 |
| CPT2 | -4.37E+00 | 1.24E-05 | 4.60E-05 |
| CR1L | 4.39E+00 | 1.12E-05 | 4.60E-05 |
| CRADD | -4.37E+00 | 1.24E-05 | 4.60E-05 |
| CRBN | -4.37E+00 | 1.24E-05 | 4.60E-05 |
| CREBL2 | -4.37E+00 | 1.24E-05 | 4.60E-05 |
| CREG1 | -4.37E+00 | 1.24E-05 | 4.60E-05 |
| CRHBP | -4.37E+00 | 1.24E-05 | 4.60E-05 |
| CRIM1 | -4.37E+00 | 1.24E-05 | 4.60E-05 |
| CRIPT | -4.37E+00 | 1.24E-05 | 4.60E-05 |
| CRLS1 | -4.37E+00 | 1.24E-05 | 4.60E-05 |
| CRTAC1 | -4.37E+00 | 1.24E-05 | 4.60E-05 |
| CRTC1 | 4.38E+00 | 1.18E-05 | 4.60E-05 |
| CRY2 | -4.37E+00 | 1.24E-05 | 4.60E-05 |
| CRYL1 | -4.37E+00 | 1.24E-05 | 4.60E-05 |
| CRYM | -4.37E+00 | 1.24E-05 | 4.60E-05 |
| CRYZL1 | -4.37E+00 | 1.24E-05 | 4.60E-05 |
| CSRNP1 | -4.37E+00 | 1.24E-05 | 4.60E-05 |
| CSRP1 | -4.37E+00 | 1.24E-05 | 4.60E-05 |
| CTAGE5 | -4.37E+00 | 1.24E-05 | 4.60E-05 |
| CTBS | -4.37E+00 | 1.24E-05 | 4.60E-05 |
| CTSF | -4.37E+00 | 1.24E-05 | 4.60E-05 |
| CTSH | -4.37E+00 | 1.24E-05 | 4.60E-05 |
| CTSO | -4.37E+00 | 1.24E-05 | 4.60E-05 |
| CWH43 | -4.37E+00 | 1.24E-05 | 4.60E-05 |
| CXCL2 | -4.37E+00 | 1.24E-05 | 4.60E-05 |
| CYB5A | -4.37E+00 | 1.24E-05 | 4.60E-05 |
| CYB5D2 | -4.37E+00 | 1.24E-05 | 4.60E-05 |
| CYBRD1 | -4.37E+00 | 1.24E-05 | 4.60E-05 |
| CYLD | -4.37E+00 | 1.24E-05 | 4.60E-05 |
| CYP17A1 | -4.37E+00 | 1.24E-05 | 4.60E-05 |
| CYP19A1 | 4.43E+00 | 9.27E-06 | 4.60E-05 |
| CYP1B1 | -4.37E+00 | 1.24E-05 | 4.60E-05 |
| CYP27A1 | -4.37E+00 | 1.24E-05 | 4.60E-05 |
| CYP2A13 | -4.37E+00 | 1.24E-05 | 4.60E-05 |
| CYP2A6 | -4.37E+00 | 1.24E-05 | 4.60E-05 |
| CYP2A7 | -4.37E+00 | 1.24E-05 | 4.60E-05 |

| CYP2B7P1 | -4.37E+00 | 1.24E-05 | 4.60E-05 |
| --- | --- | --- | --- |
| CYP2U1 | -4.37E+00 | 1.24E-05 | 4.60E-05 |
| CYP39A1 | -4.37E+00 | 1.24E-05 | 4.60E-05 |
| CYP4A11 | -4.37E+00 | 1.24E-05 | 4.60E-05 |
| CYP4A22 | -4.37E+00 | 1.24E-05 | 4.60E-05 |
| CYP4B1 | -4.37E+00 | 1.24E-05 | 4.60E-05 |
| CYP4V2 | -4.37E+00 | 1.24E-05 | 4.60E-05 |
| CYP4X1 | -4.37E+00 | 1.24E-05 | 4.60E-05 |
| CYP4Z1 | -4.37E+00 | 1.24E-05 | 4.60E-05 |
| CYP4Z2P | -4.37E+00 | 1.24E-05 | 4.60E-05 |
| CYP7A1 | -4.37E+00 | 1.24E-05 | 4.60E-05 |
| CYP7B1 | -4.37E+00 | 1.24E-05 | 4.60E-05 |
| CYYR1 | -4.37E+00 | 1.24E-05 | 4.60E-05 |
| DAAM2 | -4.37E+00 | 1.24E-05 | 4.60E-05 |
| DAPK1 | -4.37E+00 | 1.24E-05 | 4.60E-05 |
| DAPK2 | -4.37E+00 | 1.24E-05 | 4.60E-05 |
| DAXX | 4.40E+00 | 1.09E-05 | 4.60E-05 |
| DBP | -4.37E+00 | 1.24E-05 | 4.60E-05 |
| DBT | -4.37E+00 | 1.24E-05 | 4.60E-05 |
| DCAF6 | -4.37E+00 | 1.24E-05 | 4.60E-05 |
| DCAF8 | -4.37E+00 | 1.24E-05 | 4.60E-05 |
| DCN | -4.37E+00 | 1.24E-05 | 4.60E-05 |
| DCTN6 | -4.37E+00 | 1.24E-05 | 4.60E-05 |
| DCUN1D3 | -4.37E+00 | 1.24E-05 | 4.60E-05 |
| DCUN1D4 | -4.37E+00 | 1.24E-05 | 4.60E-05 |
| DCXR | -4.37E+00 | 1.24E-05 | 4.60E-05 |
| DDAH1 | -4.37E+00 | 1.24E-05 | 4.60E-05 |
| DDB2 | -4.37E+00 | 1.24E-05 | 4.60E-05 |
| DET1 | -4.37E+00 | 1.24E-05 | 4.60E-05 |
| DEXI | -4.37E+00 | 1.24E-05 | 4.60E-05 |
| DGCR6 | -4.37E+00 | 1.24E-05 | 4.60E-05 |
| DHRS1 | -4.37E+00 | 1.24E-05 | 4.60E-05 |
| DHRS12 | -4.37E+00 | 1.24E-05 | 4.60E-05 |
| DHRS3 | -4.37E+00 | 1.24E-05 | 4.60E-05 |
| DHRS7 | -4.37E+00 | 1.24E-05 | 4.60E-05 |
| DHRS7B | -4.37E+00 | 1.24E-05 | 4.60E-05 |
| DHX29 | -4.37E+00 | 1.24E-05 | 4.60E-05 |
| DIO1 | -4.37E+00 | 1.24E-05 | 4.60E-05 |
| DIO3OS | -4.37E+00 | 1.24E-05 | 4.60E-05 |
| DIRAS3 | -4.37E+00 | 1.24E-05 | 4.60E-05 |
| DIRC2 | -4.37E+00 | 1.24E-05 | 4.60E-05 |
| DIS3L | -4.37E+00 | 1.24E-05 | 4.60E-05 |
| DISP1 | -4.37E+00 | 1.24E-05 | 4.60E-05 |
| DKFZP586I1420 | -4.37E+00 | 1.24E-05 | 4.60E-05 |
| DKFZp779M0652 | -4.37E+00 | 1.24E-05 | 4.60E-05 |
| DLC1 | -4.37E+00 | 1.24E-05 | 4.60E-05 |
| DLEC1 | -4.37E+00 | 1.24E-05 | 4.60E-05 |
| DMD | -4.37E+00 | 1.24E-05 | 4.60E-05 |
| DMXL1 | -4.37E+00 | 1.24E-05 | 4.60E-05 |
| DNAH6 | -4.37E+00 | 1.24E-05 | 4.60E-05 |
| DNAJA2 | -4.37E+00 | 1.24E-05 | 4.60E-05 |
| DNAJB9 | -4.37E+00 | 1.24E-05 | 4.60E-05 |
| DNAJC19 | -4.37E+00 | 1.24E-05 | 4.60E-05 |
| DNAJC27 | -4.37E+00 | 1.24E-05 | 4.60E-05 |
| DNAJC28 | -4.37E+00 | 1.24E-05 | 4.60E-05 |
| DNAJC3 | -4.37E+00 | 1.24E-05 | 4.60E-05 |
| DNASE1L3 | -4.37E+00 | 1.24E-05 | 4.60E-05 |

| DOCK9 | -4.37E+00 | 1.24E-05 | 4.60E-05 |
| --- | --- | --- | --- |
| DPP4 | -4.37E+00 | 1.24E-05 | 4.60E-05 |
| DPT | -4.37E+00 | 1.24E-05 | 4.60E-05 |
| DPYS | -4.37E+00 | 1.24E-05 | 4.60E-05 |
| DRD1 | -4.37E+00 | 1.24E-05 | 4.60E-05 |
| DSCR3 | -4.37E+00 | 1.24E-05 | 4.60E-05 |
| DSTN | -4.37E+00 | 1.24E-05 | 4.60E-05 |
| DTWD2 | -4.37E+00 | 1.24E-05 | 4.60E-05 |
| DUSP1 | -4.37E+00 | 1.24E-05 | 4.60E-05 |
| DUSP10 | -4.37E+00 | 1.24E-05 | 4.60E-05 |
| DYNLRB2 | -4.37E+00 | 1.24E-05 | 4.60E-05 |
| DYNLT3 | -4.37E+00 | 1.24E-05 | 4.60E-05 |
| DYRK1A | -4.37E+00 | 1.24E-05 | 4.60E-05 |
| DYRK3 | -4.37E+00 | 1.24E-05 | 4.60E-05 |
| EAPP | -4.37E+00 | 1.24E-05 | 4.60E-05 |
| EBPL | -4.37E+00 | 1.24E-05 | 4.60E-05 |
| ECHDC2 | -4.37E+00 | 1.24E-05 | 4.60E-05 |
| ECHDC3 | -4.37E+00 | 1.24E-05 | 4.60E-05 |
| ECM2 | -4.37E+00 | 1.24E-05 | 4.60E-05 |
| ECSCR | -4.37E+00 | 1.24E-05 | 4.60E-05 |
| EDA2R | -4.37E+00 | 1.24E-05 | 4.60E-05 |
| EDEM1 | -4.37E+00 | 1.24E-05 | 4.60E-05 |
| EDNRB | -4.37E+00 | 1.24E-05 | 4.60E-05 |
| EFCAB1 | -4.37E+00 | 1.24E-05 | 4.60E-05 |
| EFCAB6 | -4.37E+00 | 1.24E-05 | 4.60E-05 |
| EFHA2 | -4.37E+00 | 1.24E-05 | 4.60E-05 |
| EFNA1 | -4.37E+00 | 1.24E-05 | 4.60E-05 |
| EFNB2 | -4.37E+00 | 1.24E-05 | 4.60E-05 |
| EID1 | -4.37E+00 | 1.24E-05 | 4.60E-05 |
| EIF1AD | 4.38E+00 | 1.17E-05 | 4.60E-05 |
| EIF1AX | -4.37E+00 | 1.24E-05 | 4.60E-05 |
| EIF2C4 | -4.37E+00 | 1.24E-05 | 4.60E-05 |
| EIF4E3 | -4.37E+00 | 1.24E-05 | 4.60E-05 |
| EIF4EBP2 | -4.37E+00 | 1.24E-05 | 4.60E-05 |
| EIF5 | -4.37E+00 | 1.24E-05 | 4.60E-05 |
| ELF2 | -4.37E+00 | 1.24E-05 | 4.60E-05 |
| ELL2 | -4.37E+00 | 1.24E-05 | 4.60E-05 |
| ELL3 | -4.37E+00 | 1.24E-05 | 4.60E-05 |
| ELMOD2 | -4.37E+00 | 1.24E-05 | 4.60E-05 |
| ELN | -4.37E+00 | 1.24E-05 | 4.60E-05 |
| ELTD1 | -4.37E+00 | 1.24E-05 | 4.60E-05 |
| EMCN | -4.37E+00 | 1.24E-05 | 4.60E-05 |
| EML1 | -4.37E+00 | 1.24E-05 | 4.60E-05 |
| EML5 | -4.37E+00 | 1.24E-05 | 4.60E-05 |
| EMP1 | -4.37E+00 | 1.24E-05 | 4.60E-05 |
| EMP2 | -4.37E+00 | 1.24E-05 | 4.60E-05 |
| ENOSF1 | -4.37E+00 | 1.24E-05 | 4.60E-05 |
| EPB41L4A | -4.37E+00 | 1.24E-05 | 4.60E-05 |
| EPHA4 | -4.37E+00 | 1.24E-05 | 4.60E-05 |
| EPHX1 | -4.37E+00 | 1.24E-05 | 4.60E-05 |
| EPHX2 | -4.37E+00 | 1.24E-05 | 4.60E-05 |
| EPM2A | -4.37E+00 | 1.24E-05 | 4.60E-05 |
| EPM2AIP1 | -4.37E+00 | 1.24E-05 | 4.60E-05 |
| EPS15 | -4.37E+00 | 1.24E-05 | 4.60E-05 |
| ERC1 | 4.39E+00 | 1.12E-05 | 4.60E-05 |
| ERCC5 | -4.37E+00 | 1.24E-05 | 4.60E-05 |
| ERG | -4.37E+00 | 1.24E-05 | 4.60E-05 |

| ERMAP | -4.37E+00 | 1.24E-05 | 4.60E-05 |
| --- | --- | --- | --- |
| ERO1LB | -4.37E+00 | 1.24E-05 | 4.60E-05 |
| ERP44 | -4.37E+00 | 1.24E-05 | 4.60E-05 |
| ESAM | -4.37E+00 | 1.24E-05 | 4.60E-05 |
| ESD | -4.37E+00 | 1.24E-05 | 4.60E-05 |
| ESR1 | -4.37E+00 | 1.24E-05 | 4.60E-05 |
| ETAA1 | -4.37E+00 | 1.24E-05 | 4.60E-05 |
| ETFDH | -4.37E+00 | 1.24E-05 | 4.60E-05 |
| EVPLL | -4.37E+00 | 1.24E-05 | 4.60E-05 |
| EXOC8 | -4.37E+00 | 1.24E-05 | 4.60E-05 |
| EXPH5 | -4.37E+00 | 1.24E-05 | 4.60E-05 |
| F11 | -4.37E+00 | 1.24E-05 | 4.60E-05 |
| F11R | -4.37E+00 | 1.24E-05 | 4.60E-05 |
| F8 | -4.37E+00 | 1.24E-05 | 4.60E-05 |
| FAAH | -4.37E+00 | 1.24E-05 | 4.60E-05 |
| FAAH2 | -4.37E+00 | 1.24E-05 | 4.60E-05 |
| FABP4 | -4.37E+00 | 1.24E-05 | 4.60E-05 |
| FAH | -4.37E+00 | 1.24E-05 | 4.60E-05 |
| FAM107A | -4.37E+00 | 1.24E-05 | 4.60E-05 |
| FAM107B | -4.37E+00 | 1.24E-05 | 4.60E-05 |
| FAM108B1 | -4.37E+00 | 1.24E-05 | 4.60E-05 |
| FAM122A | -4.37E+00 | 1.24E-05 | 4.60E-05 |
| FAM124B | -4.37E+00 | 1.24E-05 | 4.60E-05 |
| FAM126B | -4.37E+00 | 1.24E-05 | 4.60E-05 |
| FAM134B | -4.37E+00 | 1.24E-05 | 4.60E-05 |
| FAM13A | -4.37E+00 | 1.24E-05 | 4.60E-05 |
| FAM149A | -4.37E+00 | 1.24E-05 | 4.60E-05 |
| FAM149B1 | -4.37E+00 | 1.24E-05 | 4.60E-05 |
| FAM150B | -4.37E+00 | 1.24E-05 | 4.60E-05 |
| FAM151B | -4.37E+00 | 1.24E-05 | 4.60E-05 |
| FAM160B1 | -4.37E+00 | 1.24E-05 | 4.60E-05 |
| FAM162B | -4.37E+00 | 1.24E-05 | 4.60E-05 |
| FAM163A | 4.37E+00 | 1.23E-05 | 4.60E-05 |
| FAM164C | -4.37E+00 | 1.24E-05 | 4.60E-05 |
| FAM172A | -4.37E+00 | 1.24E-05 | 4.60E-05 |
| FAM174A | -4.37E+00 | 1.24E-05 | 4.60E-05 |
| FAM176A | -4.37E+00 | 1.24E-05 | 4.60E-05 |
| FAM177A1 | -4.37E+00 | 1.24E-05 | 4.60E-05 |
| FAM180A | -4.37E+00 | 1.24E-05 | 4.60E-05 |
| FAM185A | -4.37E+00 | 1.24E-05 | 4.60E-05 |
| FAM188A | -4.37E+00 | 1.24E-05 | 4.60E-05 |
| FAM189A2 | -4.37E+00 | 1.24E-05 | 4.60E-05 |
| FAM190B | -4.37E+00 | 1.24E-05 | 4.60E-05 |
| FAM198A | -4.37E+00 | 1.24E-05 | 4.60E-05 |
| FAM200B | -4.37E+00 | 1.24E-05 | 4.60E-05 |
| FAM20A | -4.37E+00 | 1.24E-05 | 4.60E-05 |
| FAM35B | -4.37E+00 | 1.24E-05 | 4.60E-05 |
| FAM47E | -4.37E+00 | 1.24E-05 | 4.60E-05 |
| FAM54B | -4.37E+00 | 1.24E-05 | 4.60E-05 |
| FAM76A | -4.37E+00 | 1.24E-05 | 4.60E-05 |
| FAM82A1 | -4.37E+00 | 1.24E-05 | 4.60E-05 |
| FAM82A2 | -4.37E+00 | 1.24E-05 | 4.60E-05 |
| FAM8A1 | -4.37E+00 | 1.24E-05 | 4.60E-05 |
| FAM96A | -4.37E+00 | 1.24E-05 | 4.60E-05 |
| FARS2 | -4.37E+00 | 1.24E-05 | 4.60E-05 |
| FAS | -4.37E+00 | 1.24E-05 | 4.60E-05 |
| FAT4 | -4.37E+00 | 1.24E-05 | 4.60E-05 |

| FBP1 | -4.37E+00 | 1.24E-05 | 4.60E-05 |
| --- | --- | --- | --- |
| FBXL17 | -4.37E+00 | 1.24E-05 | 4.60E-05 |
| FBXL3 | -4.37E+00 | 1.24E-05 | 4.60E-05 |
| FBXL5 | -4.37E+00 | 1.24E-05 | 4.60E-05 |
| FBXO15 | -4.37E+00 | 1.24E-05 | 4.60E-05 |
| FBXO2 | -4.37E+00 | 1.24E-05 | 4.60E-05 |
| FBXO25 | -4.37E+00 | 1.24E-05 | 4.60E-05 |
| FBXO28 | -4.37E+00 | 1.24E-05 | 4.60E-05 |
| FBXO3 | -4.37E+00 | 1.24E-05 | 4.60E-05 |
| FBXO34 | -4.37E+00 | 1.24E-05 | 4.60E-05 |
| FBXO38 | -4.37E+00 | 1.24E-05 | 4.60E-05 |
| FBXO4 | -4.37E+00 | 1.24E-05 | 4.60E-05 |
| FBXO7 | -4.37E+00 | 1.24E-05 | 4.60E-05 |
| FBXO8 | -4.37E+00 | 1.24E-05 | 4.60E-05 |
| FBXO9 | -4.37E+00 | 1.24E-05 | 4.60E-05 |
| FCER1A | -4.37E+00 | 1.24E-05 | 4.60E-05 |
| FCGRT | -4.37E+00 | 1.24E-05 | 4.60E-05 |
| FCN3 | -4.37E+00 | 1.24E-05 | 4.60E-05 |
| FDX1 | -4.37E+00 | 1.24E-05 | 4.60E-05 |
| FEM1B | -4.37E+00 | 1.24E-05 | 4.60E-05 |
| FEM1C | -4.37E+00 | 1.24E-05 | 4.60E-05 |
| FERMT2 | -4.37E+00 | 1.24E-05 | 4.60E-05 |
| FGD4 | -4.37E+00 | 1.24E-05 | 4.60E-05 |
| FGF1 | -4.37E+00 | 1.24E-05 | 4.60E-05 |
| FGF14 | -4.37E+00 | 1.24E-05 | 4.60E-05 |
| FGF7 | -4.37E+00 | 1.24E-05 | 4.60E-05 |
| FGGY | -4.37E+00 | 1.24E-05 | 4.60E-05 |
| FHL1 | -4.37E+00 | 1.24E-05 | 4.60E-05 |
| FHL5 | -4.37E+00 | 1.24E-05 | 4.60E-05 |
| FILIP1 | -4.37E+00 | 1.24E-05 | 4.60E-05 |
| FILIP1L | -4.37E+00 | 1.24E-05 | 4.60E-05 |
| FKBP7 | -4.37E+00 | 1.24E-05 | 4.60E-05 |
| FLI1 | -4.37E+00 | 1.24E-05 | 4.60E-05 |
| FLJ10038 | -4.37E+00 | 1.24E-05 | 4.60E-05 |
| FLJ11235 | -4.37E+00 | 1.24E-05 | 4.60E-05 |
| FLJ13197 | -4.37E+00 | 1.24E-05 | 4.60E-05 |
| FLJ36777 | -4.37E+00 | 1.24E-05 | 4.60E-05 |
| FLJ40852 | -4.37E+00 | 1.24E-05 | 4.60E-05 |
| FLJ42289 | -4.37E+00 | 1.24E-05 | 4.60E-05 |
| FLJ44635 | -4.37E+00 | 1.24E-05 | 4.60E-05 |
| FLJ45244 | -4.37E+00 | 1.24E-05 | 4.60E-05 |
| FMO2 | -4.37E+00 | 1.24E-05 | 4.60E-05 |
| FMO3 | -4.37E+00 | 1.24E-05 | 4.60E-05 |
| FMO4 | -4.37E+00 | 1.24E-05 | 4.60E-05 |
| FMO5 | -4.37E+00 | 1.24E-05 | 4.60E-05 |
| FNDC5 | -4.37E+00 | 1.24E-05 | 4.60E-05 |
| FNIP2 | -4.37E+00 | 1.24E-05 | 4.60E-05 |
| FOS | -4.37E+00 | 1.24E-05 | 4.60E-05 |
| FOSB | -4.37E+00 | 1.24E-05 | 4.60E-05 |
| FOXF1 | -4.37E+00 | 1.24E-05 | 4.60E-05 |
| FOXN3 | -4.37E+00 | 1.24E-05 | 4.60E-05 |
| FOXO1 | -4.37E+00 | 1.24E-05 | 4.60E-05 |
| FRG1 | -4.37E+00 | 1.24E-05 | 4.60E-05 |
| FRMD4B | -4.37E+00 | 1.24E-05 | 4.60E-05 |
| FRY | -4.37E+00 | 1.24E-05 | 4.60E-05 |
| FTO | -4.37E+00 | 1.24E-05 | 4.60E-05 |
| FUCA1 | -4.37E+00 | 1.24E-05 | 4.60E-05 |

| FXYD6 | -4.37E+00 | 1.24E-05 | 4.60E-05 |
| --- | --- | --- | --- |
| FZD8 | -4.37E+00 | 1.24E-05 | 4.60E-05 |
| GAB1 | -4.37E+00 | 1.24E-05 | 4.60E-05 |
| GABARAPL2 | -4.37E+00 | 1.24E-05 | 4.60E-05 |
| GABBR2 | -4.37E+00 | 1.24E-05 | 4.60E-05 |
| GABPA | -4.37E+00 | 1.24E-05 | 4.60E-05 |
| GAD1 | 4.45E+00 | 8.56E-06 | 4.60E-05 |
| GALNT11 | -4.37E+00 | 1.24E-05 | 4.60E-05 |
| GALNTL1 | -4.37E+00 | 1.24E-05 | 4.60E-05 |
| GALNTL2 | -4.37E+00 | 1.24E-05 | 4.60E-05 |
| GANC | -4.37E+00 | 1.24E-05 | 4.60E-05 |
| GARNL3 | -4.37E+00 | 1.24E-05 | 4.60E-05 |
| GAS6 | -4.37E+00 | 1.24E-05 | 4.60E-05 |
| GATA6 | -4.37E+00 | 1.24E-05 | 4.60E-05 |
| GATM | -4.37E+00 | 1.24E-05 | 4.60E-05 |
| GFRA1 | -4.37E+00 | 1.24E-05 | 4.60E-05 |
| GGCX | -4.37E+00 | 1.24E-05 | 4.60E-05 |
| GHR | -4.37E+00 | 1.24E-05 | 4.60E-05 |
| GIMAP5 | -4.37E+00 | 1.24E-05 | 4.60E-05 |
| GIMAP6 | -4.37E+00 | 1.24E-05 | 4.60E-05 |
| GIMAP7 | -4.37E+00 | 1.24E-05 | 4.60E-05 |
| GIN1 | -4.37E+00 | 1.24E-05 | 4.60E-05 |
| GJA5 | -4.37E+00 | 1.24E-05 | 4.60E-05 |
| GJB1 | -4.37E+00 | 1.24E-05 | 4.60E-05 |
| GK | -4.37E+00 | 1.24E-05 | 4.60E-05 |
| GLIPR1L2 | -4.37E+00 | 1.24E-05 | 4.60E-05 |
| GLS2 | -4.37E+00 | 1.24E-05 | 4.60E-05 |
| GMPR | -4.37E+00 | 1.24E-05 | 4.60E-05 |
| GMPR2 | -4.37E+00 | 1.24E-05 | 4.60E-05 |
| GNA14 | -4.37E+00 | 1.24E-05 | 4.60E-05 |
| GNAQ | -4.37E+00 | 1.24E-05 | 4.60E-05 |
| GNG10 | -4.37E+00 | 1.24E-05 | 4.60E-05 |
| GNG11 | -4.37E+00 | 1.24E-05 | 4.60E-05 |
| GNG12 | -4.37E+00 | 1.24E-05 | 4.60E-05 |
| GNG7 | -4.37E+00 | 1.24E-05 | 4.60E-05 |
| GNMT | -4.37E+00 | 1.24E-05 | 4.60E-05 |
| GNPDA1 | 4.42E+00 | 1.01E-05 | 4.60E-05 |
| GNPTG | -4.37E+00 | 1.24E-05 | 4.60E-05 |
| GPD1 | -4.37E+00 | 1.24E-05 | 4.60E-05 |
| GPIHBP1 | -4.37E+00 | 1.24E-05 | 4.60E-05 |
| GPM6A | -4.37E+00 | 1.24E-05 | 4.60E-05 |
| GPR116 | -4.37E+00 | 1.24E-05 | 4.60E-05 |
| GPR146 | -4.37E+00 | 1.24E-05 | 4.60E-05 |
| GPR152 | 4.45E+00 | 8.51E-06 | 4.60E-05 |
| GPR64 | -4.37E+00 | 1.24E-05 | 4.60E-05 |
| GPRASP2 | -4.37E+00 | 1.24E-05 | 4.60E-05 |
| GPRC5C | -4.37E+00 | 1.24E-05 | 4.60E-05 |
| GPX3 | -4.37E+00 | 1.24E-05 | 4.60E-05 |
| GRAMD3 | -4.37E+00 | 1.24E-05 | 4.60E-05 |
| GREM2 | -4.37E+00 | 1.24E-05 | 4.60E-05 |
| GRN | 4.37E+00 | 1.23E-05 | 4.60E-05 |
| GSR | 4.42E+00 | 9.72E-06 | 4.60E-05 |
| GSTA1 | -4.37E+00 | 1.24E-05 | 4.60E-05 |
| GSTA5 | -4.37E+00 | 1.24E-05 | 4.60E-05 |
| GSTK1 | -4.37E+00 | 1.24E-05 | 4.60E-05 |
| GSTM5 | -4.37E+00 | 1.24E-05 | 4.60E-05 |
| GSTO1 | -4.37E+00 | 1.24E-05 | 4.60E-05 |

| GTF2A2 | -4.37E+00 | 1.24E-05 | 4.60E-05 |
| --- | --- | --- | --- |
| GTF2H5 | -4.37E+00 | 1.24E-05 | 4.60E-05 |
| GTF2IRD2 | -4.37E+00 | 1.24E-05 | 4.60E-05 |
| GTF2IRD2B | -4.37E+00 | 1.24E-05 | 4.60E-05 |
| GTF2IRD2P1 | -4.37E+00 | 1.24E-05 | 4.60E-05 |
| GUCY1A2 | -4.37E+00 | 1.24E-05 | 4.60E-05 |
| GUCY2C | 4.37E+00 | 1.22E-05 | 4.60E-05 |
| GUSB | -4.37E+00 | 1.24E-05 | 4.60E-05 |
| GYPE | -4.37E+00 | 1.24E-05 | 4.60E-05 |
| H2AFY | 4.37E+00 | 1.23E-05 | 4.60E-05 |
| HACL1 | -4.37E+00 | 1.24E-05 | 4.60E-05 |
| HADH | -4.37E+00 | 1.24E-05 | 4.60E-05 |
| HADHB | -4.37E+00 | 1.24E-05 | 4.60E-05 |
| HAGH | -4.37E+00 | 1.24E-05 | 4.60E-05 |
| HARS | 4.40E+00 | 1.11E-05 | 4.60E-05 |
| HBB | -4.37E+00 | 1.24E-05 | 4.60E-05 |
| HBD | -4.37E+00 | 1.24E-05 | 4.60E-05 |
| HBP1 | -4.37E+00 | 1.24E-05 | 4.60E-05 |
| HCFC2 | -4.37E+00 | 1.24E-05 | 4.60E-05 |
| HDC | -4.37E+00 | 1.24E-05 | 4.60E-05 |
| HDHD2 | -4.37E+00 | 1.24E-05 | 4.60E-05 |
| HEBP1 | -4.37E+00 | 1.24E-05 | 4.60E-05 |
| HECW2 | -4.37E+00 | 1.24E-05 | 4.60E-05 |
| HELQ | -4.37E+00 | 1.24E-05 | 4.60E-05 |
| HERC3 | -4.37E+00 | 1.24E-05 | 4.60E-05 |
| HERC6 | -4.37E+00 | 1.24E-05 | 4.60E-05 |
| HERPUD1 | -4.37E+00 | 1.24E-05 | 4.60E-05 |
| HHAT | -4.37E+00 | 1.24E-05 | 4.60E-05 |
| HIBCH | -4.37E+00 | 1.24E-05 | 4.60E-05 |
| HIGD1A | -4.37E+00 | 1.24E-05 | 4.60E-05 |
| HINT2 | -4.37E+00 | 1.24E-05 | 4.60E-05 |
| HIST1H2AL | 4.43E+00 | 9.32E-06 | 4.60E-05 |
| HIST1H4E | 4.38E+00 | 1.18E-05 | 4.60E-05 |
| HLF | -4.37E+00 | 1.24E-05 | 4.60E-05 |
| HMGCL | -4.37E+00 | 1.24E-05 | 4.60E-05 |
| HMGCLL1 | -4.37E+00 | 1.24E-05 | 4.60E-05 |
| HMGCS2 | -4.37E+00 | 1.24E-05 | 4.60E-05 |
| HMOX2 | -4.37E+00 | 1.24E-05 | 4.60E-05 |
| HNMT | -4.37E+00 | 1.24E-05 | 4.60E-05 |
| HOMER2 | -4.37E+00 | 1.24E-05 | 4.60E-05 |
| HP | -4.37E+00 | 1.24E-05 | 4.60E-05 |
| HPR | -4.37E+00 | 1.24E-05 | 4.60E-05 |
| HSD11B1L | -4.37E+00 | 1.24E-05 | 4.60E-05 |
| HSD17B11 | -4.37E+00 | 1.24E-05 | 4.60E-05 |
| HSD17B12 | -4.37E+00 | 1.24E-05 | 4.60E-05 |
| HSD17B13 | -4.37E+00 | 1.24E-05 | 4.60E-05 |
| HSD17B2 | -4.37E+00 | 1.24E-05 | 4.60E-05 |
| HSD17B4 | -4.37E+00 | 1.24E-05 | 4.60E-05 |
| HSD17B6 | -4.37E+00 | 1.24E-05 | 4.60E-05 |
| HSD17B8 | -4.37E+00 | 1.24E-05 | 4.60E-05 |
| HSDL2 | -4.37E+00 | 1.24E-05 | 4.60E-05 |
| HSPB6 | -4.37E+00 | 1.24E-05 | 4.60E-05 |
| HTR2B | -4.37E+00 | 1.24E-05 | 4.60E-05 |
| HTRA1 | -4.37E+00 | 1.24E-05 | 4.60E-05 |
| IAH1 | -4.37E+00 | 1.24E-05 | 4.60E-05 |
| ICAM3 | -4.37E+00 | 1.24E-05 | 4.60E-05 |
| ID2 | -4.37E+00 | 1.24E-05 | 4.60E-05 |

| IDS | -4.37E+00 | 1.24E-05 | 4.60E-05 |
| --- | --- | --- | --- |
| IFI27 | -4.37E+00 | 1.24E-05 | 4.60E-05 |
| IFIT1 | -4.37E+00 | 1.24E-05 | 4.60E-05 |
| IFIT5 | -4.37E+00 | 1.24E-05 | 4.60E-05 |
| IFITM1 | -4.37E+00 | 1.24E-05 | 4.60E-05 |
| IFITM2 | -4.37E+00 | 1.24E-05 | 4.60E-05 |
| IFNAR1 | -4.37E+00 | 1.24E-05 | 4.60E-05 |
| IFRD2 | 4.41E+00 | 1.03E-05 | 4.60E-05 |
| IFT46 | -4.37E+00 | 1.24E-05 | 4.60E-05 |
| IFT88 | -4.37E+00 | 1.24E-05 | 4.60E-05 |
| IGF1 | -4.37E+00 | 1.24E-05 | 4.60E-05 |
| IGFBP4 | -4.37E+00 | 1.24E-05 | 4.60E-05 |
| IGFBP7 | -4.37E+00 | 1.24E-05 | 4.60E-05 |
| IKZF2 | -4.37E+00 | 1.24E-05 | 4.60E-05 |
| IKZF5 | -4.37E+00 | 1.24E-05 | 4.60E-05 |
| IL10RB | -4.37E+00 | 1.24E-05 | 4.60E-05 |
| IL13RA1 | -4.37E+00 | 1.24E-05 | 4.60E-05 |
| IL1R1 | -4.37E+00 | 1.24E-05 | 4.60E-05 |
| IL33 | -4.37E+00 | 1.24E-05 | 4.60E-05 |
| IL6R | -4.37E+00 | 1.24E-05 | 4.60E-05 |
| IMPACT | -4.37E+00 | 1.24E-05 | 4.60E-05 |
| INCA1 | -4.37E+00 | 1.24E-05 | 4.60E-05 |
| INMT | -4.37E+00 | 1.24E-05 | 4.60E-05 |
| INPP1 | -4.37E+00 | 1.24E-05 | 4.60E-05 |
| INSIG2 | -4.37E+00 | 1.24E-05 | 4.60E-05 |
| INTS10 | -4.37E+00 | 1.24E-05 | 4.60E-05 |
| IQCK | -4.37E+00 | 1.24E-05 | 4.60E-05 |
| IQGAP2 | -4.37E+00 | 1.24E-05 | 4.60E-05 |
| IRAK3 | -4.37E+00 | 1.24E-05 | 4.60E-05 |
| IRF2 | -4.37E+00 | 1.24E-05 | 4.60E-05 |
| IRF6 | -4.37E+00 | 1.24E-05 | 4.60E-05 |
| IRGC | 4.45E+00 | 8.68E-06 | 4.60E-05 |
| IRX3 | -4.37E+00 | 1.24E-05 | 4.60E-05 |
| ISCA1 | -4.37E+00 | 1.24E-05 | 4.60E-05 |
| ISCA1P1 | -4.37E+00 | 1.24E-05 | 4.60E-05 |
| ISCA2 | -4.37E+00 | 1.24E-05 | 4.60E-05 |
| ISCU | -4.37E+00 | 1.24E-05 | 4.60E-05 |
| ISOC1 | -4.37E+00 | 1.24E-05 | 4.60E-05 |
| ISPD | -4.37E+00 | 1.24E-05 | 4.60E-05 |
| ITFG1 | -4.37E+00 | 1.24E-05 | 4.60E-05 |
| ITGA1 | -4.37E+00 | 1.24E-05 | 4.60E-05 |
| ITGBL1 | -4.37E+00 | 1.24E-05 | 4.60E-05 |
| ITIH2 | -4.37E+00 | 1.24E-05 | 4.60E-05 |
| ITIH3 | -4.37E+00 | 1.24E-05 | 4.60E-05 |
| ITM2B | -4.37E+00 | 1.24E-05 | 4.60E-05 |
| ITPRIPL1 | 4.45E+00 | 8.43E-06 | 4.60E-05 |
| IVD | -4.37E+00 | 1.24E-05 | 4.60E-05 |
| IYD | -4.37E+00 | 1.24E-05 | 4.60E-05 |
| JAM2 | -4.37E+00 | 1.24E-05 | 4.60E-05 |
| JAZF1 | -4.37E+00 | 1.24E-05 | 4.60E-05 |
| JDP2 | -4.37E+00 | 1.24E-05 | 4.60E-05 |
| JKAMP | -4.37E+00 | 1.24E-05 | 4.60E-05 |
| JMY | -4.37E+00 | 1.24E-05 | 4.60E-05 |
| KANK2 | -4.37E+00 | 1.24E-05 | 4.60E-05 |
| KAT2B | -4.37E+00 | 1.24E-05 | 4.60E-05 |
| KAZ | -4.37E+00 | 1.24E-05 | 4.60E-05 |
| KBTBD11 | -4.37E+00 | 1.24E-05 | 4.60E-05 |

| KCNA5 | -4.37E+00 | 1.24E-05 | 4.60E-05 |
| --- | --- | --- | --- |
| KCNE2 | -4.37E+00 | 1.24E-05 | 4.60E-05 |
| KCNJ8 | -4.37E+00 | 1.24E-05 | 4.60E-05 |
| KCNK17 | -4.37E+00 | 1.24E-05 | 4.60E-05 |
| KCNK7 | 4.43E+00 | 9.37E-06 | 4.60E-05 |
| KCNMA1 | -4.37E+00 | 1.24E-05 | 4.60E-05 |
| KCNN2 | -4.37E+00 | 1.24E-05 | 4.60E-05 |
| KCNRG | -4.37E+00 | 1.24E-05 | 4.60E-05 |
| KCNS3 | -4.37E+00 | 1.24E-05 | 4.60E-05 |
| KCTD18 | -4.37E+00 | 1.24E-05 | 4.60E-05 |
| KDM3A | 4.40E+00 | 1.06E-05 | 4.60E-05 |
| KDR | -4.37E+00 | 1.24E-05 | 4.60E-05 |
| KDSR | -4.37E+00 | 1.24E-05 | 4.60E-05 |
| KIAA0040 | -4.37E+00 | 1.24E-05 | 4.60E-05 |
| KIAA0141 | -4.37E+00 | 1.24E-05 | 4.60E-05 |
| KIAA0247 | -4.37E+00 | 1.24E-05 | 4.60E-05 |
| KIAA0317 | 4.45E+00 | 8.69E-06 | 4.60E-05 |
| KIAA0408 | -4.37E+00 | 1.24E-05 | 4.60E-05 |
| KIAA0430 | -4.37E+00 | 1.24E-05 | 4.60E-05 |
| KIAA0494 | -4.37E+00 | 1.24E-05 | 4.60E-05 |
| KIAA1191 | -4.37E+00 | 1.24E-05 | 4.60E-05 |
| KIAA1370 | -4.37E+00 | 1.24E-05 | 4.60E-05 |
| KIAA1671 | -4.37E+00 | 1.24E-05 | 4.60E-05 |
| KIAA1712 | -4.37E+00 | 1.24E-05 | 4.60E-05 |
| KIAA1737 | -4.37E+00 | 1.24E-05 | 4.60E-05 |
| KIAA1841 | 4.39E+00 | 1.11E-05 | 4.60E-05 |
| KL | -4.37E+00 | 1.24E-05 | 4.60E-05 |
| KLC4 | -4.37E+00 | 1.24E-05 | 4.60E-05 |
| KLF12 | -4.37E+00 | 1.24E-05 | 4.60E-05 |
| KLF15 | -4.37E+00 | 1.24E-05 | 4.60E-05 |
| KLF2 | -4.37E+00 | 1.24E-05 | 4.60E-05 |
| KLF6 | -4.37E+00 | 1.24E-05 | 4.60E-05 |
| KLF8 | -4.37E+00 | 1.24E-05 | 4.60E-05 |
| KLF9 | -4.37E+00 | 1.24E-05 | 4.60E-05 |
| KLHDC1 | -4.37E+00 | 1.24E-05 | 4.60E-05 |
| KLHDC2 | -4.37E+00 | 1.24E-05 | 4.60E-05 |
| KLHDC9 | -4.37E+00 | 1.24E-05 | 4.60E-05 |
| KLHL2 | -4.37E+00 | 1.24E-05 | 4.60E-05 |
| KLHL20 | -4.37E+00 | 1.24E-05 | 4.60E-05 |
| KLHL24 | -4.37E+00 | 1.24E-05 | 4.60E-05 |
| KLHL28 | -4.37E+00 | 1.24E-05 | 4.60E-05 |
| KLHL8 | -4.37E+00 | 1.24E-05 | 4.60E-05 |
| KLRB1 | -4.37E+00 | 1.24E-05 | 4.60E-05 |
| KLRF1 | -4.37E+00 | 1.24E-05 | 4.60E-05 |
| KRCC1 | -4.37E+00 | 1.24E-05 | 4.60E-05 |
| KRT27 | -4.37E+00 | 1.24E-05 | 4.60E-05 |
| KRTAP5-1 | 4.43E+00 | 9.48E-06 | 4.60E-05 |
| KTELC1 | -4.37E+00 | 1.24E-05 | 4.60E-05 |
| LAMA2 | -4.37E+00 | 1.24E-05 | 4.60E-05 |
| LAMP2 | -4.37E+00 | 1.24E-05 | 4.60E-05 |
| LAPTM4A | -4.37E+00 | 1.24E-05 | 4.60E-05 |
| LAPTM4B | 4.40E+00 | 1.08E-05 | 4.60E-05 |
| LARGE | -4.37E+00 | 1.24E-05 | 4.60E-05 |
| LARP7 | -4.37E+00 | 1.24E-05 | 4.60E-05 |
| LASS2 | -4.37E+00 | 1.24E-05 | 4.60E-05 |
| LCA5L | -4.37E+00 | 1.24E-05 | 4.60E-05 |
| LCMT2 | -4.37E+00 | 1.24E-05 | 4.60E-05 |

| LDB2 | -4.37E+00 | 1.24E-05 | 4.60E-05 |
| --- | --- | --- | --- |
| LDB3 | -4.37E+00 | 1.24E-05 | 4.60E-05 |
| LDLRAD2 | -4.37E+00 | 1.24E-05 | 4.60E-05 |
| LDLRAP1 | -4.37E+00 | 1.24E-05 | 4.60E-05 |
| LEPR | -4.37E+00 | 1.24E-05 | 4.60E-05 |
| LHFP | -4.37E+00 | 1.24E-05 | 4.60E-05 |
| LHPP | -4.37E+00 | 1.24E-05 | 4.60E-05 |
| LIFR | -4.37E+00 | 1.24E-05 | 4.60E-05 |
| LIG4 | -4.37E+00 | 1.24E-05 | 4.60E-05 |
| LIMS2 | -4.37E+00 | 1.24E-05 | 4.60E-05 |
| LIN7C | -4.37E+00 | 1.24E-05 | 4.60E-05 |
| LINS1 | -4.37E+00 | 1.24E-05 | 4.60E-05 |
| LMBRD1 | -4.37E+00 | 1.24E-05 | 4.60E-05 |
| LMF1 | -4.37E+00 | 1.24E-05 | 4.60E-05 |
| LMO2 | -4.37E+00 | 1.24E-05 | 4.60E-05 |
| LMO7 | -4.37E+00 | 1.24E-05 | 4.60E-05 |
| LMOD1 | -4.37E+00 | 1.24E-05 | 4.60E-05 |
| LNX2 | -4.37E+00 | 1.24E-05 | 4.60E-05 |
| LOC100128542 | -4.37E+00 | 1.24E-05 | 4.60E-05 |
| LOC100128822 | -4.37E+00 | 1.24E-05 | 4.60E-05 |
| LOC100132707 | -4.37E+00 | 1.24E-05 | 4.60E-05 |
| LOC100303728 | -4.37E+00 | 1.24E-05 | 4.60E-05 |
| LOC113230 | -4.37E+00 | 1.24E-05 | 4.60E-05 |
| LOC121952 | -4.37E+00 | 1.24E-05 | 4.60E-05 |
| LOC144571 | -4.37E+00 | 1.24E-05 | 4.60E-05 |
| LOC145820 | -4.37E+00 | 1.24E-05 | 4.60E-05 |
| LOC148145 | -4.37E+00 | 1.24E-05 | 4.60E-05 |
| LOC157381 | -4.37E+00 | 1.24E-05 | 4.60E-05 |
| LOC158376 | -4.37E+00 | 1.24E-05 | 4.60E-05 |
| LOC283070 | -4.37E+00 | 1.24E-05 | 4.60E-05 |
| LOC283856 | -4.37E+00 | 1.24E-05 | 4.60E-05 |
| LOC284440 | -4.37E+00 | 1.24E-05 | 4.60E-05 |
| LOC285830 | -4.37E+00 | 1.24E-05 | 4.60E-05 |
| LOC399959 | -4.37E+00 | 1.24E-05 | 4.60E-05 |
| LOC401093 | -4.37E+00 | 1.24E-05 | 4.60E-05 |
| LOC572558 | -4.37E+00 | 1.24E-05 | 4.60E-05 |
| LOC648740 | -4.37E+00 | 1.24E-05 | 4.60E-05 |
| LOC653501 | -4.37E+00 | 1.24E-05 | 4.60E-05 |
| LOC90110 | -4.37E+00 | 1.24E-05 | 4.60E-05 |
| LOC92973 | -4.37E+00 | 1.24E-05 | 4.60E-05 |
| LOH12CR2 | -4.37E+00 | 1.24E-05 | 4.60E-05 |
| LPIN2 | -4.37E+00 | 1.24E-05 | 4.60E-05 |
| LRG1 | -4.37E+00 | 1.24E-05 | 4.60E-05 |
| LRIG1 | -4.37E+00 | 1.24E-05 | 4.60E-05 |
| LRRC2 | -4.37E+00 | 1.24E-05 | 4.60E-05 |
| LRRC50 | -4.37E+00 | 1.24E-05 | 4.60E-05 |
| LRRC70 | -4.37E+00 | 1.24E-05 | 4.60E-05 |
| LRRFIP1 | -4.37E+00 | 1.24E-05 | 4.60E-05 |
| LRTOMT | -4.37E+00 | 1.24E-05 | 4.60E-05 |
| LTC4S | -4.37E+00 | 1.24E-05 | 4.60E-05 |
| LTF | -4.37E+00 | 1.24E-05 | 4.60E-05 |
| LYPD2 | -4.37E+00 | 1.24E-05 | 4.60E-05 |
| LYRM1 | -4.37E+00 | 1.24E-05 | 4.60E-05 |
| LYRM5 | -4.37E+00 | 1.24E-05 | 4.60E-05 |
| LYRM7 | -4.37E+00 | 1.24E-05 | 4.60E-05 |
| LYSMD2 | -4.37E+00 | 1.24E-05 | 4.60E-05 |
| LYSMD3 | -4.37E+00 | 1.24E-05 | 4.60E-05 |

| MAF |  | -4.37E+00 | 1.24E-05 | 4.60E-05 |
| --- | --- | --- | --- | --- |
| MAGI2 |  | -4.37E+00 | 1.24E-05 | 4.60E-05 |
| MAGT1 |  | -4.37E+00 | 1.24E-05 | 4.60E-05 |
| MAMDC2 |  | -4.37E+00 | 1.24E-05 | 4.60E-05 |
| MAN1C1 |  | -4.37E+00 | 1.24E-05 | 4.60E-05 |
| MAOA |  | -4.37E+00 | 1.24E-05 | 4.60E-05 |
| MAOB |  | -4.37E+00 | 1.24E-05 | 4.60E-05 |
| MAP3K5 |  | -4.37E+00 | 1.24E-05 | 4.60E-05 |
| MAP6 |  | -4.37E+00 | 1.24E-05 | 4.60E-05 |
| MAPKSP1 |  | -4.37E+00 | 1.24E-05 | 4.60E-05 |
| MAPRE3 |  | -4.37E+00 | 1.24E-05 | 4.60E-05 |
|  | 2-Mar | -4.37E+00 | 1.24E-05 | 4.60E-05 |
| MASP1 |  | -4.37E+00 | 1.24E-05 | 4.60E-05 |
| MAT2B |  | -4.37E+00 | 1.24E-05 | 4.60E-05 |
| MATN2 |  | -4.37E+00 | 1.24E-05 | 4.60E-05 |
| MBIP |  | -4.37E+00 | 1.24E-05 | 4.60E-05 |
| MBLAC2 |  | -4.37E+00 | 1.24E-05 | 4.60E-05 |
| MBNL2 |  | -4.37E+00 | 1.24E-05 | 4.60E-05 |
| MCC |  | -4.37E+00 | 1.24E-05 | 4.60E-05 |
| MCEE |  | -4.37E+00 | 1.24E-05 | 4.60E-05 |
| MDP1 |  | -4.37E+00 | 1.24E-05 | 4.60E-05 |
| MED11 |  | -4.37E+00 | 1.24E-05 | 4.60E-05 |
| MED21 |  | -4.37E+00 | 1.24E-05 | 4.60E-05 |
| MEF2C |  | -4.37E+00 | 1.24E-05 | 4.60E-05 |
| MEGF9 |  | -4.37E+00 | 1.24E-05 | 4.60E-05 |
| MEIS3P1 |  | -4.37E+00 | 1.24E-05 | 4.60E-05 |
| METT5D1 |  | -4.37E+00 | 1.24E-05 | 4.60E-05 |
| METTL7A |  | -4.37E+00 | 1.24E-05 | 4.60E-05 |
| MFSD1 |  | -4.37E+00 | 1.24E-05 | 4.60E-05 |
| MFSD2A |  | -4.37E+00 | 1.24E-05 | 4.60E-05 |
| MFSD8 |  | -4.37E+00 | 1.24E-05 | 4.60E-05 |
| MGC27382 |  | -4.37E+00 | 1.24E-05 | 4.60E-05 |
| MGC3771 |  | -4.37E+00 | 1.24E-05 | 4.60E-05 |
| MGLL |  | -4.37E+00 | 1.24E-05 | 4.60E-05 |
| MGST2 |  | -4.37E+00 | 1.24E-05 | 4.60E-05 |
| MGST3 |  | -4.37E+00 | 1.24E-05 | 4.60E-05 |
| MICAL2 |  | -4.37E+00 | 1.24E-05 | 4.60E-05 |
| MICALCL |  | -4.37E+00 | 1.24E-05 | 4.60E-05 |
| MID2 |  | -4.37E+00 | 1.24E-05 | 4.60E-05 |
| MLPH |  | -4.37E+00 | 1.24E-05 | 4.60E-05 |
| MMAA |  | -4.37E+00 | 1.24E-05 | 4.60E-05 |
| MMP1 |  | 4.43E+00 | 9.27E-06 | 4.60E-05 |
| MMP17 |  | 4.45E+00 | 8.78E-06 | 4.60E-05 |
| MOAP1 |  | -4.37E+00 | 1.24E-05 | 4.60E-05 |
| MOCS1 |  | -4.37E+00 | 1.24E-05 | 4.60E-05 |
| MOCS2 |  | -4.37E+00 | 1.24E-05 | 4.60E-05 |
| MORC3 |  | -4.37E+00 | 1.24E-05 | 4.60E-05 |
| MOSC2 |  | -4.37E+00 | 1.24E-05 | 4.60E-05 |
| MPL |  | -4.37E+00 | 1.24E-05 | 4.60E-05 |
| MPP5 |  | -4.37E+00 | 1.24E-05 | 4.60E-05 |
| MPPE1 |  | -4.37E+00 | 1.24E-05 | 4.60E-05 |
| MRC1 |  | -4.37E+00 | 1.24E-05 | 4.60E-05 |
| MREG |  | -4.37E+00 | 1.24E-05 | 4.60E-05 |
| MRFAP1L1 |  | -4.37E+00 | 1.24E-05 | 4.60E-05 |
| MRPL32 |  | -4.37E+00 | 1.24E-05 | 4.60E-05 |
| MRPL34 |  | -4.37E+00 | 1.24E-05 | 4.60E-05 |
| MRPL39 |  | -4.37E+00 | 1.24E-05 | 4.60E-05 |

| MRPL42P5 | -4.37E+00 | 1.24E-05 | 4.60E-05 |
| --- | --- | --- | --- |
| MRPL54 | -4.37E+00 | 1.24E-05 | 4.60E-05 |
| MRPS36 | -4.37E+00 | 1.24E-05 | 4.60E-05 |
| MRVI1 | -4.37E+00 | 1.24E-05 | 4.60E-05 |
| MSRA | -4.37E+00 | 1.24E-05 | 4.60E-05 |
| MSRB2 | -4.37E+00 | 1.24E-05 | 4.60E-05 |
| MST1P9 | -4.37E+00 | 1.24E-05 | 4.60E-05 |
| MSTN | -4.37E+00 | 1.24E-05 | 4.60E-05 |
| MTCP1NB | -4.37E+00 | 1.24E-05 | 4.60E-05 |
| MTERFD1 | 4.43E+00 | 9.29E-06 | 4.60E-05 |
| MTERFD2 | -4.37E+00 | 1.24E-05 | 4.60E-05 |
| MTFMT | -4.37E+00 | 1.24E-05 | 4.60E-05 |
| MTHFS | -4.37E+00 | 1.24E-05 | 4.60E-05 |
| MTIF3 | -4.37E+00 | 1.24E-05 | 4.60E-05 |
| MTM1 | -4.37E+00 | 1.24E-05 | 4.60E-05 |
| MTMR10 | -4.37E+00 | 1.24E-05 | 4.60E-05 |
| MTMR6 | -4.37E+00 | 1.24E-05 | 4.60E-05 |
| MTSS1 | -4.37E+00 | 1.24E-05 | 4.60E-05 |
| MTUS1 | -4.37E+00 | 1.24E-05 | 4.60E-05 |
| MUDENG | -4.37E+00 | 1.24E-05 | 4.60E-05 |
| MUT | -4.37E+00 | 1.24E-05 | 4.60E-05 |
| MYCT1 | -4.37E+00 | 1.24E-05 | 4.60E-05 |
| MYH11 | -4.37E+00 | 1.24E-05 | 4.60E-05 |
| MYL12A | -4.37E+00 | 1.24E-05 | 4.60E-05 |
| MYL12B | -4.37E+00 | 1.24E-05 | 4.60E-05 |
| MYLK | -4.37E+00 | 1.24E-05 | 4.60E-05 |
| MYO1B | -4.37E+00 | 1.24E-05 | 4.60E-05 |
| MYOCD | -4.37E+00 | 1.24E-05 | 4.60E-05 |
| MYOM2 | -4.37E+00 | 1.24E-05 | 4.60E-05 |
| MYRIP | -4.37E+00 | 1.24E-05 | 4.60E-05 |
| N4BP2 | 4.45E+00 | 8.63E-06 | 4.60E-05 |
| N4BP2L1 | -4.37E+00 | 1.24E-05 | 4.60E-05 |
| NAAA | -4.37E+00 | 1.24E-05 | 4.60E-05 |
| NAALADL2 | -4.37E+00 | 1.24E-05 | 4.60E-05 |
| NAP1L5 | -4.37E+00 | 1.24E-05 | 4.60E-05 |
| NAPA | -4.37E+00 | 1.24E-05 | 4.60E-05 |
| NARG2 | -4.37E+00 | 1.24E-05 | 4.60E-05 |
| NAT1 | -4.37E+00 | 1.24E-05 | 4.60E-05 |
| NCAM2 | -4.37E+00 | 1.24E-05 | 4.60E-05 |
| NCOA4 | -4.37E+00 | 1.24E-05 | 4.60E-05 |
| NCRNA00093 | -4.37E+00 | 1.24E-05 | 4.60E-05 |
| NDFIP1 | -4.37E+00 | 1.24E-05 | 4.60E-05 |
| NDFIP2 | -4.37E+00 | 1.24E-05 | 4.60E-05 |
| NDNL2 | -4.37E+00 | 1.24E-05 | 4.60E-05 |
| NDRG2 | -4.37E+00 | 1.24E-05 | 4.60E-05 |
| NDUFA1 | -4.37E+00 | 1.24E-05 | 4.60E-05 |
| NDUFA2 | -4.37E+00 | 1.24E-05 | 4.60E-05 |
| NDUFA4 | -4.37E+00 | 1.24E-05 | 4.60E-05 |
| NDUFA5 | -4.37E+00 | 1.24E-05 | 4.60E-05 |
| NDUFA7 | -4.37E+00 | 1.24E-05 | 4.60E-05 |
| NDUFAF1 | -4.37E+00 | 1.24E-05 | 4.60E-05 |
| NDUFB1 | -4.37E+00 | 1.24E-05 | 4.60E-05 |
| NDUFB5 | -4.37E+00 | 1.24E-05 | 4.60E-05 |
| NDUFC1 | -4.37E+00 | 1.24E-05 | 4.60E-05 |
| NDUFV2 | -4.37E+00 | 1.24E-05 | 4.60E-05 |
| NEIL1 | -4.37E+00 | 1.24E-05 | 4.60E-05 |
| NFE2L2 | -4.37E+00 | 1.24E-05 | 4.60E-05 |

| NFIB | -4.37E+00 | 1.24E-05 | 4.60E-05 |
| --- | --- | --- | --- |
| NFIX | -4.37E+00 | 1.24E-05 | 4.60E-05 |
| NGFR | -4.37E+00 | 1.24E-05 | 4.60E-05 |
| NIPSNAP3A | -4.37E+00 | 1.24E-05 | 4.60E-05 |
| NKAPL | -4.37E+00 | 1.24E-05 | 4.60E-05 |
| NKIRAS1 | -4.37E+00 | 1.24E-05 | 4.60E-05 |
| NKX2-3 | 4.44E+00 | 8.92E-06 | 4.60E-05 |
| NMT2 | -4.37E+00 | 1.24E-05 | 4.60E-05 |
| NOSTRIN | -4.37E+00 | 1.24E-05 | 4.60E-05 |
| NPR1 | -4.37E+00 | 1.24E-05 | 4.60E-05 |
| NR1D2 | -4.37E+00 | 1.24E-05 | 4.60E-05 |
| NR2F6 | 4.43E+00 | 9.47E-06 | 4.60E-05 |
| NR3C1 | -4.37E+00 | 1.24E-05 | 4.60E-05 |
| NR3C2 | -4.37E+00 | 1.24E-05 | 4.60E-05 |
| NR4A3 | -4.37E+00 | 1.24E-05 | 4.60E-05 |
| NSUN5P2 | 4.39E+00 | 1.15E-05 | 4.60E-05 |
| NT5DC1 | -4.37E+00 | 1.24E-05 | 4.60E-05 |
| NTF3 | -4.37E+00 | 1.24E-05 | 4.60E-05 |
| NTS | 4.45E+00 | 8.52E-06 | 4.60E-05 |
| NUCB2 | -4.37E+00 | 1.24E-05 | 4.60E-05 |
| NUDCD2 | -4.37E+00 | 1.24E-05 | 4.60E-05 |
| NUDT12 | -4.37E+00 | 1.24E-05 | 4.60E-05 |
| NUDT16 | -4.37E+00 | 1.24E-05 | 4.60E-05 |
| NUDT6 | -4.37E+00 | 1.24E-05 | 4.60E-05 |
| NUDT7 | -4.37E+00 | 1.24E-05 | 4.60E-05 |
| NUMA1 | 4.40E+00 | 1.10E-05 | 4.60E-05 |
| NUMB | -4.37E+00 | 1.24E-05 | 4.60E-05 |
| NXF2 | 4.46E+00 | 8.37E-06 | 4.60E-05 |
| OAZ2 | -4.37E+00 | 1.24E-05 | 4.60E-05 |
| OCEL1 | -4.37E+00 | 1.24E-05 | 4.60E-05 |
| OCIAD1 | -4.37E+00 | 1.24E-05 | 4.60E-05 |
| OCIAD2 | -4.37E+00 | 1.24E-05 | 4.60E-05 |
| OGN | -4.37E+00 | 1.24E-05 | 4.60E-05 |
| OIT3 | -4.37E+00 | 1.24E-05 | 4.60E-05 |
| OLFM1 | -4.37E+00 | 1.24E-05 | 4.60E-05 |
| OLFML1 | -4.37E+00 | 1.24E-05 | 4.60E-05 |
| OMA1 | -4.37E+00 | 1.24E-05 | 4.60E-05 |
| OMD | -4.37E+00 | 1.24E-05 | 4.60E-05 |
| OR51B4 | 4.41E+00 | 1.05E-05 | 4.60E-05 |
| ORM1 | -4.37E+00 | 1.24E-05 | 4.60E-05 |
| ORM2 | -4.37E+00 | 1.24E-05 | 4.60E-05 |
| ORMDL3 | -4.37E+00 | 1.24E-05 | 4.60E-05 |
| OSBPL1A | -4.37E+00 | 1.24E-05 | 4.60E-05 |
| OSTF1 | -4.37E+00 | 1.24E-05 | 4.60E-05 |
| OTC | -4.37E+00 | 1.24E-05 | 4.60E-05 |
| P2RY14 | -4.37E+00 | 1.24E-05 | 4.60E-05 |
| P4HTM | -4.37E+00 | 1.24E-05 | 4.60E-05 |
| PACRG | -4.37E+00 | 1.24E-05 | 4.60E-05 |
| PAFAH2 | -4.37E+00 | 1.24E-05 | 4.60E-05 |
| PALMD | -4.37E+00 | 1.24E-05 | 4.60E-05 |
| PAPSS2 | -4.37E+00 | 1.24E-05 | 4.60E-05 |
| PARP16 | -4.37E+00 | 1.24E-05 | 4.60E-05 |
| PARP3 | -4.37E+00 | 1.24E-05 | 4.60E-05 |
| PARVA | -4.37E+00 | 1.24E-05 | 4.60E-05 |
| PBLD | -4.37E+00 | 1.24E-05 | 4.60E-05 |
| PCBD1 | -4.37E+00 | 1.24E-05 | 4.60E-05 |
| PCBD2 | -4.37E+00 | 1.24E-05 | 4.60E-05 |

| PCCA | -4.37E+00 | 1.24E-05 | 4.60E-05 |
| --- | --- | --- | --- |
| PCDH20 | -4.37E+00 | 1.24E-05 | 4.60E-05 |
| PCGF5 | -4.37E+00 | 1.24E-05 | 4.60E-05 |
| PCTP | -4.37E+00 | 1.24E-05 | 4.60E-05 |
| PCYOX1 | -4.37E+00 | 1.24E-05 | 4.60E-05 |
| PDE6D | -4.37E+00 | 1.24E-05 | 4.60E-05 |
| PDE7B | -4.37E+00 | 1.24E-05 | 4.60E-05 |
| PDGFC | -4.37E+00 | 1.24E-05 | 4.60E-05 |
| PDGFD | -4.37E+00 | 1.24E-05 | 4.60E-05 |
| PDIK1L | -4.37E+00 | 1.24E-05 | 4.60E-05 |
| PDK2 | -4.37E+00 | 1.24E-05 | 4.60E-05 |
| PDK4 | -4.37E+00 | 1.24E-05 | 4.60E-05 |
| PDLIM2 | -4.37E+00 | 1.24E-05 | 4.60E-05 |
| PDSS2 | -4.37E+00 | 1.24E-05 | 4.60E-05 |
| PDZK1IP1 | -4.37E+00 | 1.24E-05 | 4.60E-05 |
| PDZRN4 | -4.37E+00 | 1.24E-05 | 4.60E-05 |
| PEBP4 | -4.37E+00 | 1.24E-05 | 4.60E-05 |
| PECAM1 | -4.37E+00 | 1.24E-05 | 4.60E-05 |
| PECI | -4.37E+00 | 1.24E-05 | 4.60E-05 |
| PER3 | -4.37E+00 | 1.24E-05 | 4.60E-05 |
| PEX11A | -4.37E+00 | 1.24E-05 | 4.60E-05 |
| PEX11B | -4.37E+00 | 1.24E-05 | 4.60E-05 |
| PEX11G | -4.37E+00 | 1.24E-05 | 4.60E-05 |
| PEX12 | -4.37E+00 | 1.24E-05 | 4.60E-05 |
| PEX13 | -4.37E+00 | 1.24E-05 | 4.60E-05 |
| PEX19 | -4.37E+00 | 1.24E-05 | 4.60E-05 |
| PEX7 | -4.37E+00 | 1.24E-05 | 4.60E-05 |
| PGAP3 | -4.37E+00 | 1.24E-05 | 4.60E-05 |
| PGCP | -4.37E+00 | 1.24E-05 | 4.60E-05 |
| PGGT1B | -4.37E+00 | 1.24E-05 | 4.60E-05 |
| PGM5 | -4.37E+00 | 1.24E-05 | 4.60E-05 |
| PGR | -4.37E+00 | 1.24E-05 | 4.60E-05 |
| PGRMC1 | -4.37E+00 | 1.24E-05 | 4.60E-05 |
| PGRMC2 | -4.37E+00 | 1.24E-05 | 4.60E-05 |
| PHACTR3 | -4.37E+00 | 1.24E-05 | 4.60E-05 |
| PHKB | -4.37E+00 | 1.24E-05 | 4.60E-05 |
| PHYH | -4.37E+00 | 1.24E-05 | 4.60E-05 |
| PHYHD1 | -4.37E+00 | 1.24E-05 | 4.60E-05 |
| PI4K2B | -4.37E+00 | 1.24E-05 | 4.60E-05 |
| PID1 | -4.37E+00 | 1.24E-05 | 4.60E-05 |
| PIGB | -4.37E+00 | 1.24E-05 | 4.60E-05 |
| PIGK | -4.37E+00 | 1.24E-05 | 4.60E-05 |
| PIGP | -4.37E+00 | 1.24E-05 | 4.60E-05 |
| PIGV | -4.37E+00 | 1.24E-05 | 4.60E-05 |
| PIGY | -4.37E+00 | 1.24E-05 | 4.60E-05 |
| PIK3R1 | -4.37E+00 | 1.24E-05 | 4.60E-05 |
| PION | -4.37E+00 | 1.24E-05 | 4.60E-05 |
| PJA2 | -4.37E+00 | 1.24E-05 | 4.60E-05 |
| PLA2G12A | -4.37E+00 | 1.24E-05 | 4.60E-05 |
| PLA2G5 | -4.37E+00 | 1.24E-05 | 4.60E-05 |
| PLAC9 | -4.37E+00 | 1.24E-05 | 4.60E-05 |
| PLAT | -4.37E+00 | 1.24E-05 | 4.60E-05 |
| PLCXD3 | -4.37E+00 | 1.24E-05 | 4.60E-05 |
| PLDN | -4.37E+00 | 1.24E-05 | 4.60E-05 |
| PLEKHA3 | -4.37E+00 | 1.24E-05 | 4.60E-05 |
| PLEKHG1 | -4.37E+00 | 1.24E-05 | 4.60E-05 |
| PLIN5 | -4.37E+00 | 1.24E-05 | 4.60E-05 |

| PLS3 | -4.37E+00 | 1.24E-05 | 4.60E-05 |
| --- | --- | --- | --- |
| PLSCR4 | -4.37E+00 | 1.24E-05 | 4.60E-05 |
| PNMT | -4.37E+00 | 1.24E-05 | 4.60E-05 |
| PNPLA4 | -4.37E+00 | 1.24E-05 | 4.60E-05 |
| PNPLA8 | -4.37E+00 | 1.24E-05 | 4.60E-05 |
| PNRC1 | -4.37E+00 | 1.24E-05 | 4.60E-05 |
| POLK | -4.37E+00 | 1.24E-05 | 4.60E-05 |
| POLR3GL | -4.37E+00 | 1.24E-05 | 4.60E-05 |
| POM121L9P | -4.37E+00 | 1.24E-05 | 4.60E-05 |
| PON1 | -4.37E+00 | 1.24E-05 | 4.60E-05 |
| PON3 | -4.37E+00 | 1.24E-05 | 4.60E-05 |
| POPDC2 | -4.37E+00 | 1.24E-05 | 4.60E-05 |
| POU6F2 | 4.44E+00 | 9.02E-06 | 4.60E-05 |
| PPA2 | -4.37E+00 | 1.24E-05 | 4.60E-05 |
| PPAP2A | -4.37E+00 | 1.24E-05 | 4.60E-05 |
| PPAP2B | -4.37E+00 | 1.24E-05 | 4.60E-05 |
| PPCS | -4.37E+00 | 1.24E-05 | 4.60E-05 |
| PPFIBP2 | -4.37E+00 | 1.24E-05 | 4.60E-05 |
| PPIL3 | -4.37E+00 | 1.24E-05 | 4.60E-05 |
| PPM1A | -4.37E+00 | 1.24E-05 | 4.60E-05 |
| PPM1K | -4.37E+00 | 1.24E-05 | 4.60E-05 |
| PPP1CB | -4.37E+00 | 1.24E-05 | 4.60E-05 |
| PPP1R12B | -4.37E+00 | 1.24E-05 | 4.60E-05 |
| PPP2R5A | -4.37E+00 | 1.24E-05 | 4.60E-05 |
| PPP2R5C | -4.37E+00 | 1.24E-05 | 4.60E-05 |
| PPP3CA | -4.37E+00 | 1.24E-05 | 4.60E-05 |
| PPP4R4 | -4.37E+00 | 1.24E-05 | 4.60E-05 |
| PQLC3 | -4.37E+00 | 1.24E-05 | 4.60E-05 |
| PRDM7 | 4.39E+00 | 1.15E-05 | 4.60E-05 |
| PRELP | -4.37E+00 | 1.24E-05 | 4.60E-05 |
| PRICKLE4 | -4.37E+00 | 1.24E-05 | 4.60E-05 |
| PRKAG2 | -4.37E+00 | 1.24E-05 | 4.60E-05 |
| PRKCE | -4.37E+00 | 1.24E-05 | 4.60E-05 |
| PRKCH | -4.37E+00 | 1.24E-05 | 4.60E-05 |
| PRMT10 | -4.37E+00 | 1.24E-05 | 4.60E-05 |
| PRODH | -4.37E+00 | 1.24E-05 | 4.60E-05 |
| PROS1 | -4.37E+00 | 1.24E-05 | 4.60E-05 |
| PRPF18 | -4.37E+00 | 1.24E-05 | 4.60E-05 |
| PRRG4 | -4.37E+00 | 1.24E-05 | 4.60E-05 |
| PRTFDC1 | 4.39E+00 | 1.15E-05 | 4.60E-05 |
| PSEN1 | -4.37E+00 | 1.24E-05 | 4.60E-05 |
| PSME1 | -4.37E+00 | 1.24E-05 | 4.60E-05 |
| PTAR1 | -4.37E+00 | 1.24E-05 | 4.60E-05 |
| PTEN | -4.37E+00 | 1.24E-05 | 4.60E-05 |
| PTGER3 | -4.37E+00 | 1.24E-05 | 4.60E-05 |
| PTH1R | -4.37E+00 | 1.24E-05 | 4.60E-05 |
| PTK2B | -4.37E+00 | 1.24E-05 | 4.60E-05 |
| PTN | -4.37E+00 | 1.24E-05 | 4.60E-05 |
| PTPLAD1 | -4.37E+00 | 1.24E-05 | 4.60E-05 |
| PTPN21 | -4.37E+00 | 1.24E-05 | 4.60E-05 |
| PTPN3 | -4.37E+00 | 1.24E-05 | 4.60E-05 |
| PTPN4 | -4.37E+00 | 1.24E-05 | 4.60E-05 |
| PTPRB | -4.37E+00 | 1.24E-05 | 4.60E-05 |
| PTPRD | -4.37E+00 | 1.24E-05 | 4.60E-05 |
| PTPRM | -4.37E+00 | 1.24E-05 | 4.60E-05 |
| PTPRT | -4.37E+00 | 1.24E-05 | 4.60E-05 |
| PTPRU | -4.37E+00 | 1.24E-05 | 4.60E-05 |

| PUS10 | -4.37E+00 | 1.24E-05 | 4.60E-05 |
| --- | --- | --- | --- |
| PYROXD1 | -4.37E+00 | 1.24E-05 | 4.60E-05 |
| RAB12 | -4.37E+00 | 1.24E-05 | 4.60E-05 |
| RAB14 | -4.37E+00 | 1.24E-05 | 4.60E-05 |
| RAB17 | -4.37E+00 | 1.24E-05 | 4.60E-05 |
| RAB18 | -4.37E+00 | 1.24E-05 | 4.60E-05 |
| RAB20 | -4.37E+00 | 1.24E-05 | 4.60E-05 |
| RAB33B | -4.37E+00 | 1.24E-05 | 4.60E-05 |
| RAB4A | -4.37E+00 | 1.24E-05 | 4.60E-05 |
| RAB5A | -4.37E+00 | 1.24E-05 | 4.60E-05 |
| RAB9A | -4.37E+00 | 1.24E-05 | 4.60E-05 |
| RAD51C | 4.41E+00 | 1.05E-05 | 4.60E-05 |
| RAI2 | -4.37E+00 | 1.24E-05 | 4.60E-05 |
| RAMP2 | -4.37E+00 | 1.24E-05 | 4.60E-05 |
| RAMP3 | -4.37E+00 | 1.24E-05 | 4.60E-05 |
| RANBP3L | -4.37E+00 | 1.24E-05 | 4.60E-05 |
| RANBP6 | -4.37E+00 | 1.24E-05 | 4.60E-05 |
| RAP2C | -4.37E+00 | 1.24E-05 | 4.60E-05 |
| RAPGEF2 | -4.37E+00 | 1.24E-05 | 4.60E-05 |
| RAPGEF5 | -4.37E+00 | 1.24E-05 | 4.60E-05 |
| RAPH1 | -4.37E+00 | 1.24E-05 | 4.60E-05 |
| RASGEF1B | -4.37E+00 | 1.24E-05 | 4.60E-05 |
| RASL11A | -4.37E+00 | 1.24E-05 | 4.60E-05 |
| RBBP9 | -4.37E+00 | 1.24E-05 | 4.60E-05 |
| RBKS | -4.37E+00 | 1.24E-05 | 4.60E-05 |
| RBL2 | -4.37E+00 | 1.24E-05 | 4.60E-05 |
| RBM43 | -4.37E+00 | 1.24E-05 | 4.60E-05 |
| RBMS3 | -4.37E+00 | 1.24E-05 | 4.60E-05 |
| RBP5 | -4.37E+00 | 1.24E-05 | 4.60E-05 |
| RCAN1 | -4.37E+00 | 1.24E-05 | 4.60E-05 |
| RCAN2 | -4.37E+00 | 1.24E-05 | 4.60E-05 |
| RCBTB2 | -4.37E+00 | 1.24E-05 | 4.60E-05 |
| REEP3 | -4.37E+00 | 1.24E-05 | 4.60E-05 |
| REEP5 | -4.37E+00 | 1.24E-05 | 4.60E-05 |
| REM1 | -4.37E+00 | 1.24E-05 | 4.60E-05 |
| REN | -4.37E+00 | 1.24E-05 | 4.60E-05 |
| REPS2 | -4.37E+00 | 1.24E-05 | 4.60E-05 |
| RER1 | -4.37E+00 | 1.24E-05 | 4.60E-05 |
| RERG | -4.37E+00 | 1.24E-05 | 4.60E-05 |
| RERGL | -4.37E+00 | 1.24E-05 | 4.60E-05 |
| RETSAT | -4.37E+00 | 1.24E-05 | 4.60E-05 |
| RFESD | -4.37E+00 | 1.24E-05 | 4.60E-05 |
| RFTN1 | -4.37E+00 | 1.24E-05 | 4.60E-05 |
| RFX3 | 4.41E+00 | 1.05E-05 | 4.60E-05 |
| RGMA | -4.37E+00 | 1.24E-05 | 4.60E-05 |
| RGN | -4.37E+00 | 1.24E-05 | 4.60E-05 |
| RGS11 | -4.37E+00 | 1.24E-05 | 4.60E-05 |
| RGS5 | -4.37E+00 | 1.24E-05 | 4.60E-05 |
| RGS6 | -4.37E+00 | 1.24E-05 | 4.60E-05 |
| RGS9 | -4.37E+00 | 1.24E-05 | 4.60E-05 |
| RHCE | -4.37E+00 | 1.24E-05 | 4.60E-05 |
| RHD | -4.37E+00 | 1.24E-05 | 4.60E-05 |
| RHOJ | -4.37E+00 | 1.24E-05 | 4.60E-05 |
| RHOU | -4.37E+00 | 1.24E-05 | 4.60E-05 |
| RILP | -4.37E+00 | 1.24E-05 | 4.60E-05 |
| RIOK3 | -4.37E+00 | 1.24E-05 | 4.60E-05 |
| RNASE4 | -4.37E+00 | 1.24E-05 | 4.60E-05 |

| RNF11 | -4.37E+00 | 1.24E-05 | 4.60E-05 |
| --- | --- | --- | --- |
| RNF125 | -4.37E+00 | 1.24E-05 | 4.60E-05 |
| RNF128 | -4.37E+00 | 1.24E-05 | 4.60E-05 |
| RNF13 | -4.37E+00 | 1.24E-05 | 4.60E-05 |
| RNF130 | -4.37E+00 | 1.24E-05 | 4.60E-05 |
| RNF14 | -4.37E+00 | 1.24E-05 | 4.60E-05 |
| RNF141 | -4.37E+00 | 1.24E-05 | 4.60E-05 |
| RNF144B | -4.37E+00 | 1.24E-05 | 4.60E-05 |
| RNF167 | -4.37E+00 | 1.24E-05 | 4.60E-05 |
| RNF170 | -4.37E+00 | 1.24E-05 | 4.60E-05 |
| RNF180 | -4.37E+00 | 1.24E-05 | 4.60E-05 |
| RNF185 | -4.37E+00 | 1.24E-05 | 4.60E-05 |
| RNF187 | 4.46E+00 | 8.38E-06 | 4.60E-05 |
| RNF5 | -4.37E+00 | 1.24E-05 | 4.60E-05 |
| RNF5P1 | -4.37E+00 | 1.24E-05 | 4.60E-05 |
| RNLS | -4.37E+00 | 1.24E-05 | 4.60E-05 |
| ROGDI | -4.37E+00 | 1.24E-05 | 4.60E-05 |
| ROPN1B | -4.37E+00 | 1.24E-05 | 4.60E-05 |
| RORA | -4.37E+00 | 1.24E-05 | 4.60E-05 |
| RORC | -4.37E+00 | 1.24E-05 | 4.60E-05 |
| RPL36AL | -4.37E+00 | 1.24E-05 | 4.60E-05 |
| RPS27L | -4.37E+00 | 1.24E-05 | 4.60E-05 |
| RPS6KA2 | -4.37E+00 | 1.24E-05 | 4.60E-05 |
| RRH | -4.37E+00 | 1.24E-05 | 4.60E-05 |
| RSAD2 | -4.37E+00 | 1.24E-05 | 4.60E-05 |
| RTN4RL1 | -4.37E+00 | 1.24E-05 | 4.60E-05 |
| RUNDC3B | -4.37E+00 | 1.24E-05 | 4.60E-05 |
| RWDD2B | -4.37E+00 | 1.24E-05 | 4.60E-05 |
| RWDD4A | -4.37E+00 | 1.24E-05 | 4.60E-05 |
| S1PR1 | -4.37E+00 | 1.24E-05 | 4.60E-05 |
| SAP18 | -4.37E+00 | 1.24E-05 | 4.60E-05 |
| SAP30L | -4.37E+00 | 1.24E-05 | 4.60E-05 |
| SAR1B | -4.37E+00 | 1.24E-05 | 4.60E-05 |
| SAT1 | -4.37E+00 | 1.24E-05 | 4.60E-05 |
| SAT2 | -4.37E+00 | 1.24E-05 | 4.60E-05 |
| SAV1 | -4.37E+00 | 1.24E-05 | 4.60E-05 |
| SBDS | -4.37E+00 | 1.24E-05 | 4.60E-05 |
| SC4MOL | -4.37E+00 | 1.24E-05 | 4.60E-05 |
| SC5DL | -4.37E+00 | 1.24E-05 | 4.60E-05 |
| SCAI | -4.37E+00 | 1.24E-05 | 4.60E-05 |
| SCARB2 | -4.37E+00 | 1.24E-05 | 4.60E-05 |
| SCCPDH | -4.37E+00 | 1.24E-05 | 4.60E-05 |
| SCLY | 4.38E+00 | 1.20E-05 | 4.60E-05 |
| SCN4B | -4.37E+00 | 1.24E-05 | 4.60E-05 |
| SCOC | -4.37E+00 | 1.24E-05 | 4.60E-05 |
| SCP2 | -4.37E+00 | 1.24E-05 | 4.60E-05 |
| SCYL3 | -4.37E+00 | 1.24E-05 | 4.60E-05 |
| SDC1 | -4.37E+00 | 1.24E-05 | 4.60E-05 |
| SDHC | -4.37E+00 | 1.24E-05 | 4.60E-05 |
| SDHD | -4.37E+00 | 1.24E-05 | 4.60E-05 |
| SDPR | -4.37E+00 | 1.24E-05 | 4.60E-05 |
| SDR42E1 | -4.37E+00 | 1.24E-05 | 4.60E-05 |
| SEC14L3 | -4.37E+00 | 1.24E-05 | 4.60E-05 |
| SEC22C | -4.37E+00 | 1.24E-05 | 4.60E-05 |
| SEC62 | -4.37E+00 | 1.24E-05 | 4.60E-05 |
| SECISBP2L | -4.37E+00 | 1.24E-05 | 4.60E-05 |
| SELENBP1 | -4.37E+00 | 1.24E-05 | 4.60E-05 |

| SELP |  | -4.37E+00 | 1.24E-05 | 4.60E-05 |
| --- | --- | --- | --- | --- |
| SEMA5A |  | -4.37E+00 | 1.24E-05 | 4.60E-05 |
| SENP8 |  | -4.37E+00 | 1.24E-05 | 4.60E-05 |
| SEPP1 |  | -4.37E+00 | 1.24E-05 | 4.60E-05 |
| SEPSECS |  | -4.37E+00 | 1.24E-05 | 4.60E-05 |
|  | 4-Sep | -4.37E+00 | 1.24E-05 | 4.60E-05 |
| SERINC1 |  | -4.37E+00 | 1.24E-05 | 4.60E-05 |
| SERPIND1 |  | -4.37E+00 | 1.24E-05 | 4.60E-05 |
| SESN1 |  | -4.37E+00 | 1.24E-05 | 4.60E-05 |
| SETD3 |  | -4.37E+00 | 1.24E-05 | 4.60E-05 |
| SETDB2 |  | -4.37E+00 | 1.24E-05 | 4.60E-05 |
| SFRS2B |  | -4.37E+00 | 1.24E-05 | 4.60E-05 |
| SFRS5 |  | -4.37E+00 | 1.24E-05 | 4.60E-05 |
| SFTA1P |  | -4.37E+00 | 1.24E-05 | 4.60E-05 |
| SFTPD |  | -4.37E+00 | 1.24E-05 | 4.60E-05 |
| SFXN2 |  | -4.37E+00 | 1.24E-05 | 4.60E-05 |
| SGCB |  | -4.37E+00 | 1.24E-05 | 4.60E-05 |
| SGCD |  | -4.37E+00 | 1.24E-05 | 4.60E-05 |
| SGK1 |  | -4.37E+00 | 1.24E-05 | 4.60E-05 |
| SGMS2 |  | -4.37E+00 | 1.24E-05 | 4.60E-05 |
| SGPP1 |  | -4.37E+00 | 1.24E-05 | 4.60E-05 |
| SH2D4A |  | -4.37E+00 | 1.24E-05 | 4.60E-05 |
| SH3BGR |  | -4.37E+00 | 1.24E-05 | 4.60E-05 |
| SH3BGRL2 |  | -4.37E+00 | 1.24E-05 | 4.60E-05 |
| SH3D19 |  | -4.37E+00 | 1.24E-05 | 4.60E-05 |
| SH3YL1 |  | -4.37E+00 | 1.24E-05 | 4.60E-05 |
| SHE |  | -4.37E+00 | 1.24E-05 | 4.60E-05 |
| SHISA3 |  | -4.37E+00 | 1.24E-05 | 4.60E-05 |
| SHROOM3 |  | -4.37E+00 | 1.24E-05 | 4.60E-05 |
| SHROOM4 |  | -4.37E+00 | 1.24E-05 | 4.60E-05 |
| SIAE |  | -4.37E+00 | 1.24E-05 | 4.60E-05 |
| SIRT1 |  | -4.37E+00 | 1.24E-05 | 4.60E-05 |
| SIRT3 |  | -4.37E+00 | 1.24E-05 | 4.60E-05 |
| SKINTL |  | -4.37E+00 | 1.24E-05 | 4.60E-05 |
| SLC10A7 |  | -4.37E+00 | 1.24E-05 | 4.60E-05 |
| SLC14A1 |  | -4.37E+00 | 1.24E-05 | 4.60E-05 |
| SLC16A11 |  | -4.37E+00 | 1.24E-05 | 4.60E-05 |
| SLC16A4 |  | -4.37E+00 | 1.24E-05 | 4.60E-05 |
| SLC19A2 |  | -4.37E+00 | 1.24E-05 | 4.60E-05 |
| SLC1A1 |  | -4.37E+00 | 1.24E-05 | 4.60E-05 |
| SLC1A2 |  | -4.37E+00 | 1.24E-05 | 4.60E-05 |
| SLC22A3 |  | -4.37E+00 | 1.24E-05 | 4.60E-05 |
| SLC25A10 |  | 4.44E+00 | 8.89E-06 | 4.60E-05 |
| SLC25A17 |  | -4.37E+00 | 1.24E-05 | 4.60E-05 |
| SLC25A20 |  | -4.37E+00 | 1.24E-05 | 4.60E-05 |
| SLC25A23 |  | -4.37E+00 | 1.24E-05 | 4.60E-05 |
| SLC25A30 |  | -4.37E+00 | 1.24E-05 | 4.60E-05 |
| SLC25A4 |  | -4.37E+00 | 1.24E-05 | 4.60E-05 |
| SLC25A42 |  | -4.37E+00 | 1.24E-05 | 4.60E-05 |
| SLC26A8 |  | -4.37E+00 | 1.24E-05 | 4.60E-05 |
| SLC27A1 |  | -4.37E+00 | 1.24E-05 | 4.60E-05 |
| SLC2A12 |  | -4.37E+00 | 1.24E-05 | 4.60E-05 |
| SLC30A9 |  | -4.37E+00 | 1.24E-05 | 4.60E-05 |
| SLC31A1 |  | -4.37E+00 | 1.24E-05 | 4.60E-05 |
| SLC31A2 |  | -4.37E+00 | 1.24E-05 | 4.60E-05 |
| SLC35A1 |  | -4.37E+00 | 1.24E-05 | 4.60E-05 |
| SLC35A3 |  | -4.37E+00 | 1.24E-05 | 4.60E-05 |

| SLC35B3 | -4.37E+00 | 1.24E-05 | 4.60E-05 |
| --- | --- | --- | --- |
| SLC35F5 | -4.37E+00 | 1.24E-05 | 4.60E-05 |
| SLC38A4 | -4.37E+00 | 1.24E-05 | 4.60E-05 |
| SLC39A8 | -4.37E+00 | 1.24E-05 | 4.60E-05 |
| SLC39A9 | -4.37E+00 | 1.24E-05 | 4.60E-05 |
| SLC40A1 | -4.37E+00 | 1.24E-05 | 4.60E-05 |
| SLC46A3 | -4.37E+00 | 1.24E-05 | 4.60E-05 |
| SLC47A1 | -4.37E+00 | 1.24E-05 | 4.60E-05 |
| SLC4A4 | -4.37E+00 | 1.24E-05 | 4.60E-05 |
| SLCO2A1 | -4.37E+00 | 1.24E-05 | 4.60E-05 |
| SLPI | -4.37E+00 | 1.24E-05 | 4.60E-05 |
| SMAD6 | -4.37E+00 | 1.24E-05 | 4.60E-05 |
| SMAP2 | -4.37E+00 | 1.24E-05 | 4.60E-05 |
| SMARCA2 | -4.37E+00 | 1.24E-05 | 4.60E-05 |
| SMPD1 | -4.37E+00 | 1.24E-05 | 4.60E-05 |
| SMPDL3A | -4.37E+00 | 1.24E-05 | 4.60E-05 |
| SNED1 | -4.37E+00 | 1.24E-05 | 4.60E-05 |
| SNRK | -4.37E+00 | 1.24E-05 | 4.60E-05 |
| SNX1 | -4.37E+00 | 1.24E-05 | 4.60E-05 |
| SOCS2 | -4.37E+00 | 1.24E-05 | 4.60E-05 |
| SORBS1 | -4.37E+00 | 1.24E-05 | 4.60E-05 |
| SORBS2 | -4.37E+00 | 1.24E-05 | 4.60E-05 |
| SOS2 | -4.37E+00 | 1.24E-05 | 4.60E-05 |
| SOX30 | 4.43E+00 | 9.64E-06 | 4.60E-05 |
| SP100 | -4.37E+00 | 1.24E-05 | 4.60E-05 |
| SP6 | 4.43E+00 | 9.38E-06 | 4.60E-05 |
| SPAG7 | -4.37E+00 | 1.24E-05 | 4.60E-05 |
| SPARCL1 | -4.37E+00 | 1.24E-05 | 4.60E-05 |
| SPATA1 | -4.37E+00 | 1.24E-05 | 4.60E-05 |
| SPATA18 | -4.37E+00 | 1.24E-05 | 4.60E-05 |
| SPATA7 | -4.37E+00 | 1.24E-05 | 4.60E-05 |
| SPATA9 | -4.37E+00 | 1.24E-05 | 4.60E-05 |
| SPCS3 | -4.37E+00 | 1.24E-05 | 4.60E-05 |
| SPG21 | -4.37E+00 | 1.24E-05 | 4.60E-05 |
| SPHAR | -4.37E+00 | 1.24E-05 | 4.60E-05 |
| SPON1 | -4.37E+00 | 1.24E-05 | 4.60E-05 |
| SPPL2A | -4.37E+00 | 1.24E-05 | 4.60E-05 |
| SPRY1 | -4.37E+00 | 1.24E-05 | 4.60E-05 |
| SPSB2 | 4.45E+00 | 8.42E-06 | 4.60E-05 |
| SPZ1 | 4.42E+00 | 9.71E-06 | 4.60E-05 |
| SRCRB4D | 4.46E+00 | 8.35E-06 | 4.60E-05 |
| SRD5A2 | -4.37E+00 | 1.24E-05 | 4.60E-05 |
| SRD5A3 | -4.37E+00 | 1.24E-05 | 4.60E-05 |
| SRL | -4.37E+00 | 1.24E-05 | 4.60E-05 |
| SRP9 | -4.37E+00 | 1.24E-05 | 4.60E-05 |
| SRPX | -4.37E+00 | 1.24E-05 | 4.60E-05 |
| SRR | -4.37E+00 | 1.24E-05 | 4.60E-05 |
| SSTR1 | -4.37E+00 | 1.24E-05 | 4.60E-05 |
| SSX7 | 4.42E+00 | 1.01E-05 | 4.60E-05 |
| ST3GAL6 | -4.37E+00 | 1.24E-05 | 4.60E-05 |
| ST6GAL1 | -4.37E+00 | 1.24E-05 | 4.60E-05 |
| ST6GALNAC2 | -4.37E+00 | 1.24E-05 | 4.60E-05 |
| ST6GALNAC3 | -4.37E+00 | 1.24E-05 | 4.60E-05 |
| ST7 | -4.37E+00 | 1.24E-05 | 4.60E-05 |
| ST8SIA6 | -4.37E+00 | 1.24E-05 | 4.60E-05 |
| STARD10 | -4.37E+00 | 1.24E-05 | 4.60E-05 |
| STARD13 | -4.37E+00 | 1.24E-05 | 4.60E-05 |

| STEAP3 | -4.37E+00 | 1.24E-05 | 4.60E-05 |
| --- | --- | --- | --- |
| STEAP4 | -4.37E+00 | 1.24E-05 | 4.60E-05 |
| STK16 | -4.37E+00 | 1.24E-05 | 4.60E-05 |
| STK4 | 4.42E+00 | 1.01E-05 | 4.60E-05 |
| STOM | -4.37E+00 | 1.24E-05 | 4.60E-05 |
| STRADB | -4.37E+00 | 1.24E-05 | 4.60E-05 |
| STS | -4.37E+00 | 1.24E-05 | 4.60E-05 |
| STX12 | -4.37E+00 | 1.24E-05 | 4.60E-05 |
| STX17 | -4.37E+00 | 1.24E-05 | 4.60E-05 |
| STXBP2 | 4.44E+00 | 8.99E-06 | 4.60E-05 |
| SUCLG1 | -4.37E+00 | 1.24E-05 | 4.60E-05 |
| SUCLG2 | -4.37E+00 | 1.24E-05 | 4.60E-05 |
| SUGT1L1 | -4.37E+00 | 1.24E-05 | 4.60E-05 |
| SULT1A1 | -4.37E+00 | 1.24E-05 | 4.60E-05 |
| SULT1A2 | -4.37E+00 | 1.24E-05 | 4.60E-05 |
| SULT1B1 | -4.37E+00 | 1.24E-05 | 4.60E-05 |
| SUMF1 | -4.37E+00 | 1.24E-05 | 4.60E-05 |
| SUMO1 | -4.37E+00 | 1.24E-05 | 4.60E-05 |
| SUMO1P3 | -4.37E+00 | 1.24E-05 | 4.60E-05 |
| SURF1 | -4.37E+00 | 1.24E-05 | 4.60E-05 |
| SVEP1 | -4.37E+00 | 1.24E-05 | 4.60E-05 |
| SYBU | -4.37E+00 | 1.24E-05 | 4.60E-05 |
| SYNE1 | -4.37E+00 | 1.24E-05 | 4.60E-05 |
| SYNJ2BP | -4.37E+00 | 1.24E-05 | 4.60E-05 |
| SYNPO | -4.37E+00 | 1.24E-05 | 4.60E-05 |
| SYNPO2 | -4.37E+00 | 1.24E-05 | 4.60E-05 |
| SYPL1 | -4.37E+00 | 1.24E-05 | 4.60E-05 |
| SYTL2 | -4.37E+00 | 1.24E-05 | 4.60E-05 |
| TADA1 | -4.37E+00 | 1.24E-05 | 4.60E-05 |
| TAF3 | 4.38E+00 | 1.21E-05 | 4.60E-05 |
| TAF7 | -4.37E+00 | 1.24E-05 | 4.60E-05 |
| TAF9B | -4.37E+00 | 1.24E-05 | 4.60E-05 |
| TAL1 | -4.37E+00 | 1.24E-05 | 4.60E-05 |
| TAPT1 | -4.37E+00 | 1.24E-05 | 4.60E-05 |
| TATDN3 | -4.37E+00 | 1.24E-05 | 4.60E-05 |
| TBC1D23 | -4.37E+00 | 1.24E-05 | 4.60E-05 |
| TBC1D8B | -4.37E+00 | 1.24E-05 | 4.60E-05 |
| TBCK | -4.37E+00 | 1.24E-05 | 4.60E-05 |
| TCEAL1 | -4.37E+00 | 1.24E-05 | 4.60E-05 |
| TCEAL4 | -4.37E+00 | 1.24E-05 | 4.60E-05 |
| TCEAL7 | -4.37E+00 | 1.24E-05 | 4.60E-05 |
| TCEANC | -4.37E+00 | 1.24E-05 | 4.60E-05 |
| TCF21 | -4.37E+00 | 1.24E-05 | 4.60E-05 |
| TCF4 | -4.37E+00 | 1.24E-05 | 4.60E-05 |
| TCTA | -4.37E+00 | 1.24E-05 | 4.60E-05 |
| TCTEX1D4 | -4.37E+00 | 1.24E-05 | 4.60E-05 |
| TDRD10 | -4.37E+00 | 1.24E-05 | 4.60E-05 |
| TDRD3 | -4.37E+00 | 1.24E-05 | 4.60E-05 |
| TEF | -4.37E+00 | 1.24E-05 | 4.60E-05 |
| TEK | -4.37E+00 | 1.24E-05 | 4.60E-05 |
| TENC1 | -4.37E+00 | 1.24E-05 | 4.60E-05 |
| TERF2IP | -4.37E+00 | 1.24E-05 | 4.60E-05 |
| TESK2 | -4.37E+00 | 1.24E-05 | 4.60E-05 |
| TET2 | -4.37E+00 | 1.24E-05 | 4.60E-05 |
| TGDS | -4.37E+00 | 1.24E-05 | 4.60E-05 |
| TGFBR3 | -4.37E+00 | 1.24E-05 | 4.60E-05 |
| THAP1 | -4.37E+00 | 1.24E-05 | 4.60E-05 |

| THAP6 | -4.37E+00 | 1.24E-05 | 4.60E-05 |
| --- | --- | --- | --- |
| THAP9 | -4.37E+00 | 1.24E-05 | 4.60E-05 |
| THBD | -4.37E+00 | 1.24E-05 | 4.60E-05 |
| THRB | -4.37E+00 | 1.24E-05 | 4.60E-05 |
| THSD1 | -4.37E+00 | 1.24E-05 | 4.60E-05 |
| THSD7B | -4.37E+00 | 1.24E-05 | 4.60E-05 |
| TIGD2 | -4.37E+00 | 1.24E-05 | 4.60E-05 |
| TIMP3 | -4.37E+00 | 1.24E-05 | 4.60E-05 |
| TINF2 | -4.37E+00 | 1.24E-05 | 4.60E-05 |
| TIPARP | -4.37E+00 | 1.24E-05 | 4.60E-05 |
| TK2 | -4.37E+00 | 1.24E-05 | 4.60E-05 |
| TLE2 | -4.37E+00 | 1.24E-05 | 4.60E-05 |
| TLR3 | -4.37E+00 | 1.24E-05 | 4.60E-05 |
| TM2D3 | -4.37E+00 | 1.24E-05 | 4.60E-05 |
| TM4SF18 | -4.37E+00 | 1.24E-05 | 4.60E-05 |
| TM4SF20 | 4.38E+00 | 1.21E-05 | 4.60E-05 |
| TM7SF2 | -4.37E+00 | 1.24E-05 | 4.60E-05 |
| TM7SF3 | -4.37E+00 | 1.24E-05 | 4.60E-05 |
| TMBIM4 | -4.37E+00 | 1.24E-05 | 4.60E-05 |
| TMED10 | -4.37E+00 | 1.24E-05 | 4.60E-05 |
| TMED7 | -4.37E+00 | 1.24E-05 | 4.60E-05 |
| TMEM100 | -4.37E+00 | 1.24E-05 | 4.60E-05 |
| TMEM106B | -4.37E+00 | 1.24E-05 | 4.60E-05 |
| TMEM133 | -4.37E+00 | 1.24E-05 | 4.60E-05 |
| TMEM161B | -4.37E+00 | 1.24E-05 | 4.60E-05 |
| TMEM192 | -4.37E+00 | 1.24E-05 | 4.60E-05 |
| TMEM220 | -4.37E+00 | 1.24E-05 | 4.60E-05 |
| TMEM232 | -4.37E+00 | 1.24E-05 | 4.60E-05 |
| TMEM233 | -4.37E+00 | 1.24E-05 | 4.60E-05 |
| TMEM27 | -4.37E+00 | 1.24E-05 | 4.60E-05 |
| TMEM30B | -4.37E+00 | 1.24E-05 | 4.60E-05 |
| TMEM47 | -4.37E+00 | 1.24E-05 | 4.60E-05 |
| TMEM50B | -4.37E+00 | 1.24E-05 | 4.60E-05 |
| TMEM56 | -4.37E+00 | 1.24E-05 | 4.60E-05 |
| TMEM57 | -4.37E+00 | 1.24E-05 | 4.60E-05 |
| TMEM59 | -4.37E+00 | 1.24E-05 | 4.60E-05 |
| TMEM66 | -4.37E+00 | 1.24E-05 | 4.60E-05 |
| TMEM85 | -4.37E+00 | 1.24E-05 | 4.60E-05 |
| TMOD1 | -4.37E+00 | 1.24E-05 | 4.60E-05 |
| TMPRSS2 | -4.37E+00 | 1.24E-05 | 4.60E-05 |
| TMPRSS6 | -4.37E+00 | 1.24E-05 | 4.60E-05 |
| TMPRSS9 | 4.42E+00 | 1.01E-05 | 4.60E-05 |
| TMX4 | -4.37E+00 | 1.24E-05 | 4.60E-05 |
| TNFSF10 | -4.37E+00 | 1.24E-05 | 4.60E-05 |
| TNNI3K | -4.37E+00 | 1.24E-05 | 4.60E-05 |
| TNXB | -4.37E+00 | 1.24E-05 | 4.60E-05 |
| TOB1 | -4.37E+00 | 1.24E-05 | 4.60E-05 |
| TOM1L1 | -4.37E+00 | 1.24E-05 | 4.60E-05 |
| TOMM5 | 4.40E+00 | 1.11E-05 | 4.60E-05 |
| TOR1A | -4.37E+00 | 1.24E-05 | 4.60E-05 |
| TOR1AIP1 | -4.37E+00 | 1.24E-05 | 4.60E-05 |
| TP53TG1 | -4.37E+00 | 1.24E-05 | 4.60E-05 |
| TPCN2 | 4.45E+00 | 8.44E-06 | 4.60E-05 |
| TPMT | -4.37E+00 | 1.24E-05 | 4.60E-05 |
| TPPP | -4.37E+00 | 1.24E-05 | 4.60E-05 |
| TPPP2 | -4.37E+00 | 1.24E-05 | 4.60E-05 |
| TPRG1 | -4.37E+00 | 1.24E-05 | 4.60E-05 |

| TRAF6 | -4.37E+00 | 1.24E-05 | 4.60E-05 |
| --- | --- | --- | --- |
| TRAPPC6B | -4.37E+00 | 1.24E-05 | 4.60E-05 |
| TRIM13 | -4.37E+00 | 1.24E-05 | 4.60E-05 |
| TRIM2 | -4.37E+00 | 1.24E-05 | 4.60E-05 |
| TRIM22 | -4.37E+00 | 1.24E-05 | 4.60E-05 |
| TRIM23 | -4.37E+00 | 1.24E-05 | 4.60E-05 |
| TRIM38 | -4.37E+00 | 1.24E-05 | 4.60E-05 |
| TRIM9 | 4.39E+00 | 1.12E-05 | 4.60E-05 |
| TRPC6 | -4.37E+00 | 1.24E-05 | 4.60E-05 |
| TRPM7 | -4.37E+00 | 1.24E-05 | 4.60E-05 |
| TSC22D3 | -4.37E+00 | 1.24E-05 | 4.60E-05 |
| TSLP | -4.37E+00 | 1.24E-05 | 4.60E-05 |
| TSPAN1 | -4.37E+00 | 1.24E-05 | 4.60E-05 |
| TSPYL1 | -4.37E+00 | 1.24E-05 | 4.60E-05 |
| TST | -4.37E+00 | 1.24E-05 | 4.60E-05 |
| TSTD1 | -4.37E+00 | 1.24E-05 | 4.60E-05 |
| TTC19 | -4.37E+00 | 1.24E-05 | 4.60E-05 |
| TTC33 | -4.37E+00 | 1.24E-05 | 4.60E-05 |
| TTC38 | -4.37E+00 | 1.24E-05 | 4.60E-05 |
| TTC39B | -4.37E+00 | 1.24E-05 | 4.60E-05 |
| TXNDC11 | -4.37E+00 | 1.24E-05 | 4.60E-05 |
| TXNDC15 | -4.37E+00 | 1.24E-05 | 4.60E-05 |
| TXNDC16 | -4.37E+00 | 1.24E-05 | 4.60E-05 |
| TXNIP | -4.37E+00 | 1.24E-05 | 4.60E-05 |
| UBA7 | -4.37E+00 | 1.24E-05 | 4.60E-05 |
| UBB | -4.37E+00 | 1.24E-05 | 4.60E-05 |
| UBE2B | -4.37E+00 | 1.24E-05 | 4.60E-05 |
| UBE2D3 | -4.37E+00 | 1.24E-05 | 4.60E-05 |
| UBE2I | 4.42E+00 | 9.68E-06 | 4.60E-05 |
| UBL3 | -4.37E+00 | 1.24E-05 | 4.60E-05 |
| UBXN10 | -4.37E+00 | 1.24E-05 | 4.60E-05 |
| UFSP2 | -4.37E+00 | 1.24E-05 | 4.60E-05 |
| UGP2 | -4.37E+00 | 1.24E-05 | 4.60E-05 |
| UNC50 | -4.37E+00 | 1.24E-05 | 4.60E-05 |
| UPP2 | -4.37E+00 | 1.24E-05 | 4.60E-05 |
| UQCR11 | -4.37E+00 | 1.24E-05 | 4.60E-05 |
| UQCRC2 | -4.37E+00 | 1.24E-05 | 4.60E-05 |
| UQCRQ | -4.37E+00 | 1.24E-05 | 4.60E-05 |
| USP38 | -4.37E+00 | 1.24E-05 | 4.60E-05 |
| USP53 | -4.37E+00 | 1.24E-05 | 4.60E-05 |
| USP8 | -4.37E+00 | 1.24E-05 | 4.60E-05 |
| UST | -4.37E+00 | 1.24E-05 | 4.60E-05 |
| VAMP2 | -4.37E+00 | 1.24E-05 | 4.60E-05 |
| VAMP4 | -4.37E+00 | 1.24E-05 | 4.60E-05 |
| VAMP5 | -4.37E+00 | 1.24E-05 | 4.60E-05 |
| VIPR1 | -4.37E+00 | 1.24E-05 | 4.60E-05 |
| VKORC1 | -4.37E+00 | 1.24E-05 | 4.60E-05 |
| VSIG2 | -4.37E+00 | 1.24E-05 | 4.60E-05 |
| VTI1B | -4.37E+00 | 1.24E-05 | 4.60E-05 |
| VWA3B | -4.37E+00 | 1.24E-05 | 4.60E-05 |
| WAPAL | 4.41E+00 | 1.05E-05 | 4.60E-05 |
| WASH5P | 4.40E+00 | 1.11E-05 | 4.60E-05 |
| WBP1 | -4.37E+00 | 1.24E-05 | 4.60E-05 |
| WBSCR17 | -4.37E+00 | 1.24E-05 | 4.60E-05 |
| WDR20 | -4.37E+00 | 1.24E-05 | 4.60E-05 |
| WDR25 | -4.37E+00 | 1.24E-05 | 4.60E-05 |
| WDR65 | -4.37E+00 | 1.24E-05 | 4.60E-05 |

| WDR90 | 4.44E+00 | 8.84E-06 | 4.60E-05 |
| --- | --- | --- | --- |
| WFDC12 | -4.37E+00 | 1.24E-05 | 4.60E-05 |
| WFDC5 | -4.37E+00 | 1.24E-05 | 4.60E-05 |
| WRB | -4.37E+00 | 1.24E-05 | 4.60E-05 |
| WSCD2 | -4.37E+00 | 1.24E-05 | 4.60E-05 |
| WWC2 | -4.37E+00 | 1.24E-05 | 4.60E-05 |
| YAF2 | -4.37E+00 | 1.24E-05 | 4.60E-05 |
| YPEL1 | -4.37E+00 | 1.24E-05 | 4.60E-05 |
| YPEL2 | -4.37E+00 | 1.24E-05 | 4.60E-05 |
| YPEL3 | -4.37E+00 | 1.24E-05 | 4.60E-05 |
| YPEL5 | -4.37E+00 | 1.24E-05 | 4.60E-05 |
| YTHDC2 | -4.37E+00 | 1.24E-05 | 4.60E-05 |
| ZBTB16 | -4.37E+00 | 1.24E-05 | 4.60E-05 |
| ZBTB7C | -4.37E+00 | 1.24E-05 | 4.60E-05 |
| ZBTB8A | -4.37E+00 | 1.24E-05 | 4.60E-05 |
| ZC3H6 | -4.37E+00 | 1.24E-05 | 4.60E-05 |
| ZCCHC24 | -4.37E+00 | 1.24E-05 | 4.60E-05 |
| ZDHHC15 | -4.37E+00 | 1.24E-05 | 4.60E-05 |
| ZDHHC6 | -4.37E+00 | 1.24E-05 | 4.60E-05 |
| ZFAND3 | 4.44E+00 | 9.10E-06 | 4.60E-05 |
| ZFAND5 | -4.37E+00 | 1.24E-05 | 4.60E-05 |
| ZFAND6 | -4.37E+00 | 1.24E-05 | 4.60E-05 |
| ZFP1 | -4.37E+00 | 1.24E-05 | 4.60E-05 |
| ZFP161 | -4.37E+00 | 1.24E-05 | 4.60E-05 |
| ZFP2 | -4.37E+00 | 1.24E-05 | 4.60E-05 |
| ZFP36 | -4.37E+00 | 1.24E-05 | 4.60E-05 |
| ZFP36L1 | -4.37E+00 | 1.24E-05 | 4.60E-05 |
| ZFPM2 | -4.37E+00 | 1.24E-05 | 4.60E-05 |
| ZFYVE21 | -4.37E+00 | 1.24E-05 | 4.60E-05 |
| ZIC4 | 4.45E+00 | 8.76E-06 | 4.60E-05 |
| ZMAT1 | -4.37E+00 | 1.24E-05 | 4.60E-05 |
| ZMAT3 | -4.37E+00 | 1.24E-05 | 4.60E-05 |
| ZMPSTE24 | -4.37E+00 | 1.24E-05 | 4.60E-05 |
| ZMYND11 | -4.37E+00 | 1.24E-05 | 4.60E-05 |
| ZMYND12 | -4.37E+00 | 1.24E-05 | 4.60E-05 |
| ZNF136 | -4.37E+00 | 1.24E-05 | 4.60E-05 |
| ZNF17 | -4.37E+00 | 1.24E-05 | 4.60E-05 |
| ZNF175 | -4.37E+00 | 1.24E-05 | 4.60E-05 |
| ZNF181 | -4.37E+00 | 1.24E-05 | 4.60E-05 |
| ZNF236 | 4.43E+00 | 9.37E-06 | 4.60E-05 |
| ZNF238 | -4.37E+00 | 1.24E-05 | 4.60E-05 |
| ZNF25 | -4.37E+00 | 1.24E-05 | 4.60E-05 |
| ZNF280D | -4.37E+00 | 1.24E-05 | 4.60E-05 |
| ZNF295 | -4.37E+00 | 1.24E-05 | 4.60E-05 |
| ZNF33A | -4.37E+00 | 1.24E-05 | 4.60E-05 |
| ZNF385B | -4.37E+00 | 1.24E-05 | 4.60E-05 |
| ZNF425 | -4.37E+00 | 1.24E-05 | 4.60E-05 |
| ZNF429 | -4.37E+00 | 1.24E-05 | 4.60E-05 |
| ZNF436 | -4.37E+00 | 1.24E-05 | 4.60E-05 |
| ZNF441 | -4.37E+00 | 1.24E-05 | 4.60E-05 |
| ZNF442 | -4.37E+00 | 1.24E-05 | 4.60E-05 |
| ZNF451 | -4.37E+00 | 1.24E-05 | 4.60E-05 |
| ZNF487 | -4.37E+00 | 1.24E-05 | 4.60E-05 |
| ZNF510 | -4.37E+00 | 1.24E-05 | 4.60E-05 |
| ZNF521 | -4.37E+00 | 1.24E-05 | 4.60E-05 |
| ZNF540 | -4.37E+00 | 1.24E-05 | 4.60E-05 |
| ZNF546 | -4.37E+00 | 1.24E-05 | 4.60E-05 |

| ZNF563 | -4.37E+00 | 1.24E-05 | 4.60E-05 |
| --- | --- | --- | --- |
| ZNF564 | -4.37E+00 | 1.24E-05 | 4.60E-05 |
| ZNF596 | -4.37E+00 | 1.24E-05 | 4.60E-05 |
| ZNF627 | -4.37E+00 | 1.24E-05 | 4.60E-05 |
| ZNF654 | -4.37E+00 | 1.24E-05 | 4.60E-05 |
| ZNF658 | -4.37E+00 | 1.24E-05 | 4.60E-05 |
| ZNF662 | -4.37E+00 | 1.24E-05 | 4.60E-05 |
| ZNF671 | -4.37E+00 | 1.24E-05 | 4.60E-05 |
| ZNF684 | -4.37E+00 | 1.24E-05 | 4.60E-05 |
| ZNF688 | -4.37E+00 | 1.24E-05 | 4.60E-05 |
| ZNF711 | 4.38E+00 | 1.17E-05 | 4.60E-05 |
| ZNF75D | -4.37E+00 | 1.24E-05 | 4.60E-05 |
| ZNF763 | -4.37E+00 | 1.24E-05 | 4.60E-05 |
| ZNF776 | -4.37E+00 | 1.24E-05 | 4.60E-05 |
| ZNF790 | -4.37E+00 | 1.24E-05 | 4.60E-05 |
| ZNF791 | -4.37E+00 | 1.24E-05 | 4.60E-05 |
| ZNF799 | -4.37E+00 | 1.24E-05 | 4.60E-05 |
| ZNF823 | -4.37E+00 | 1.24E-05 | 4.60E-05 |
| ZNF846 | -4.37E+00 | 1.24E-05 | 4.60E-05 |
| ZNRF2 | -4.37E+00 | 1.24E-05 | 4.60E-05 |
| ZSWIM7 | -4.37E+00 | 1.24E-05 | 4.60E-05 |
| ZYG11B | -4.37E+00 | 1.24E-05 | 4.60E-05 |
| WBSCR22 | 4.37E+00 | 1.24E-05 | 4.60E-05 |
| HOXC4 | 4.37E+00 | 1.27E-05 | 4.72E-05 |
| IPO8 | 4.37E+00 | 1.27E-05 | 4.72E-05 |
| EDARADD | 4.36E+00 | 1.29E-05 | 4.78E-05 |
| SUPT7L | 4.36E+00 | 1.29E-05 | 4.78E-05 |
| RPL39L | 4.36E+00 | 1.29E-05 | 4.79E-05 |
| INSL3 | 4.36E+00 | 1.30E-05 | 4.82E-05 |
| KLK6 | 4.36E+00 | 1.31E-05 | 4.84E-05 |
| ZNF286B | 4.36E+00 | 1.31E-05 | 4.84E-05 |
| LOC341056 | 4.36E+00 | 1.31E-05 | 4.86E-05 |
| TAOK1 | 4.36E+00 | 1.32E-05 | 4.90E-05 |
| CEMP1 | 4.36E+00 | 1.33E-05 | 4.90E-05 |
| VPS18 | 4.36E+00 | 1.33E-05 | 4.93E-05 |
| UNC93B1 | 4.35E+00 | 1.34E-05 | 4.95E-05 |
| AKIRIN1 | 4.35E+00 | 1.34E-05 | 4.96E-05 |
| AGTRAP | 4.35E+00 | 1.35E-05 | 4.99E-05 |
| LOC285548 | 4.35E+00 | 1.35E-05 | 4.99E-05 |
| DSCR8 | 4.35E+00 | 1.35E-05 | 4.99E-05 |
| NKX3-2 | 4.35E+00 | 1.36E-05 | 5.02E-05 |
| IFNE | 4.35E+00 | 1.36E-05 | 5.02E-05 |
| SHANK1 | 4.35E+00 | 1.37E-05 | 5.04E-05 |
| MLLT1 | 4.35E+00 | 1.38E-05 | 5.10E-05 |
| LPO | 4.35E+00 | 1.38E-05 | 5.11E-05 |
| LHFPL5 | 4.35E+00 | 1.39E-05 | 5.14E-05 |
| GFPT2 | 4.35E+00 | 1.39E-05 | 5.14E-05 |
| OSBP2 | 4.34E+00 | 1.40E-05 | 5.16E-05 |
| PCSK7 | 4.34E+00 | 1.40E-05 | 5.17E-05 |
| SSX1 | 4.34E+00 | 1.41E-05 | 5.19E-05 |
| HIST1H2AD | 4.34E+00 | 1.41E-05 | 5.21E-05 |
| FOXR2 | 4.34E+00 | 1.41E-05 | 5.21E-05 |
| LOC387646 | 4.34E+00 | 1.42E-05 | 5.24E-05 |
| RNASE10 | 4.34E+00 | 1.43E-05 | 5.28E-05 |
| CCL7 | 4.34E+00 | 1.43E-05 | 5.28E-05 |
| COPB2 | 4.34E+00 | 1.44E-05 | 5.29E-05 |
| GZMB | 4.34E+00 | 1.45E-05 | 5.34E-05 |

| C15orf41 | 4.34E+00 | 1.46E-05 | 5.38E-05 |
| --- | --- | --- | --- |
| TRAM2 | 4.33E+00 | 1.48E-05 | 5.44E-05 |
| VCY | 4.33E+00 | 1.48E-05 | 5.44E-05 |
| C17orf99 | 4.33E+00 | 1.48E-05 | 5.46E-05 |
| EIF2C1 | 4.33E+00 | 1.48E-05 | 5.46E-05 |
| PALB2 | 4.33E+00 | 1.51E-05 | 5.54E-05 |
| GABRR1 | 4.33E+00 | 1.52E-05 | 5.60E-05 |
| VPS8 | 4.33E+00 | 1.52E-05 | 5.61E-05 |
| ERP29 | 4.33E+00 | 1.53E-05 | 5.62E-05 |
| ELF4 | 4.32E+00 | 1.55E-05 | 5.68E-05 |
| COPA | 4.32E+00 | 1.56E-05 | 5.73E-05 |
| ZNF257 | 4.32E+00 | 1.56E-05 | 5.74E-05 |
| B4GALT6 | 4.32E+00 | 1.57E-05 | 5.78E-05 |
| KIAA2013 | 4.32E+00 | 1.60E-05 | 5.86E-05 |
| ZNF283 | 4.31E+00 | 1.61E-05 | 5.89E-05 |
| CMIP | 4.31E+00 | 1.61E-05 | 5.91E-05 |
| DDX53 | 4.31E+00 | 1.61E-05 | 5.91E-05 |
| KRTAP20-4 | 4.31E+00 | 1.61E-05 | 5.91E-05 |
| MRPS17 | 4.31E+00 | 1.62E-05 | 5.96E-05 |
| NSFL1C | 4.31E+00 | 1.63E-05 | 5.96E-05 |
| RAPGEFL1 | 4.31E+00 | 1.63E-05 | 5.97E-05 |
| GPD2 | 4.31E+00 | 1.63E-05 | 5.98E-05 |
| NEUROD1 | 4.31E+00 | 1.65E-05 | 6.06E-05 |
| C17orf80 | 4.31E+00 | 1.66E-05 | 6.08E-05 |
| AHCY | 4.30E+00 | 1.68E-05 | 6.14E-05 |
| GRIA2 | 4.30E+00 | 1.68E-05 | 6.14E-05 |
| AAGAB | 4.30E+00 | 1.69E-05 | 6.17E-05 |
| SEPHS1 | 4.30E+00 | 1.69E-05 | 6.18E-05 |
| IL17RD | 4.30E+00 | 1.70E-05 | 6.21E-05 |
| GGN | 4.30E+00 | 1.71E-05 | 6.25E-05 |
| SNRNP40 | 4.30E+00 | 1.71E-05 | 6.26E-05 |
| C9orf30 | 4.30E+00 | 1.71E-05 | 6.27E-05 |
| SFXN4 | 4.30E+00 | 1.73E-05 | 6.32E-05 |
| DPPA2 | 4.30E+00 | 1.73E-05 | 6.33E-05 |
| IDH3G | 4.30E+00 | 1.73E-05 | 6.34E-05 |
| FKRP | 4.30E+00 | 1.74E-05 | 6.36E-05 |
| ARHGEF1 | 4.30E+00 | 1.75E-05 | 6.38E-05 |
| SPOCK1 | 4.30E+00 | 1.75E-05 | 6.38E-05 |
| HCN3 | 4.30E+00 | 1.75E-05 | 6.40E-05 |
| ALMS1 | 4.29E+00 | 1.75E-05 | 6.41E-05 |
| WT1 | 4.29E+00 | 1.76E-05 | 6.41E-05 |
| USP39 | 4.29E+00 | 1.78E-05 | 6.52E-05 |
| KRT18 | 4.29E+00 | 1.78E-05 | 6.52E-05 |
| SLC39A5 | 4.29E+00 | 1.80E-05 | 6.56E-05 |
| LOC100216001 | 4.29E+00 | 1.81E-05 | 6.60E-05 |
| LOC152217 | 4.29E+00 | 1.81E-05 | 6.61E-05 |
| PHF20 | 4.29E+00 | 1.82E-05 | 6.65E-05 |
| REP15 | 4.29E+00 | 1.82E-05 | 6.66E-05 |
| HIST1H2AG | 4.29E+00 | 1.83E-05 | 6.66E-05 |
| C6orf153 | 4.29E+00 | 1.83E-05 | 6.67E-05 |
| CLN6 | 4.29E+00 | 1.83E-05 | 6.68E-05 |
| FNDC7 | 4.28E+00 | 1.84E-05 | 6.72E-05 |
| CRYBA2 | 4.28E+00 | 1.85E-05 | 6.75E-05 |
| GREM1 | 4.28E+00 | 1.86E-05 | 6.79E-05 |
| KLK1 | 4.28E+00 | 1.86E-05 | 6.79E-05 |
| LHB | 4.28E+00 | 1.87E-05 | 6.81E-05 |
| SDAD1 | 4.28E+00 | 1.87E-05 | 6.82E-05 |

| LOC100302401 | 4.28E+00 | 1.89E-05 | 6.89E-05 |
| --- | --- | --- | --- |
| RPL8 | 4.28E+00 | 1.89E-05 | 6.89E-05 |
| KLK15 | 4.28E+00 | 1.90E-05 | 6.91E-05 |
| FLJ42627 | 4.28E+00 | 1.90E-05 | 6.93E-05 |
| GNGT1 | 4.28E+00 | 1.91E-05 | 6.95E-05 |
| MTG1 | 4.27E+00 | 1.92E-05 | 6.99E-05 |
| SQSTM1 | 4.27E+00 | 1.92E-05 | 6.99E-05 |
| LSM14A | 4.27E+00 | 1.93E-05 | 7.03E-05 |
| SP1 | 4.27E+00 | 1.94E-05 | 7.05E-05 |
| MAPK8IP3 | 4.27E+00 | 1.95E-05 | 7.08E-05 |
| P2RY4 | 4.27E+00 | 1.95E-05 | 7.09E-05 |
| CEND1 | 4.27E+00 | 1.97E-05 | 7.17E-05 |
| EMILIN2 | 4.27E+00 | 1.97E-05 | 7.17E-05 |
| C3orf66 | 4.27E+00 | 1.99E-05 | 7.23E-05 |
| LRP5 | 4.26E+00 | 2.01E-05 | 7.30E-05 |
| SPON2 | 4.26E+00 | 2.02E-05 | 7.32E-05 |
| IFNG | 4.26E+00 | 2.03E-05 | 7.36E-05 |
| CACNG8 | 4.26E+00 | 2.03E-05 | 7.37E-05 |
| C22orf23 | 4.26E+00 | 2.03E-05 | 7.38E-05 |
| PAQR4 | 4.26E+00 | 2.03E-05 | 7.38E-05 |
| IRGQ | 4.26E+00 | 2.05E-05 | 7.43E-05 |
| CRKL | 4.26E+00 | 2.05E-05 | 7.45E-05 |
| OTUB2 | 4.26E+00 | 2.06E-05 | 7.46E-05 |
| SRRM4 | 4.26E+00 | 2.07E-05 | 7.50E-05 |
| BDKRB1 | 4.26E+00 | 2.08E-05 | 7.53E-05 |
| GGCT | 4.26E+00 | 2.08E-05 | 7.55E-05 |
| PCLO | 4.26E+00 | 2.08E-05 | 7.55E-05 |
| KAAG1 | 4.25E+00 | 2.10E-05 | 7.61E-05 |
| TRPV3 | 4.25E+00 | 2.10E-05 | 7.62E-05 |
| GCSH | 4.25E+00 | 2.11E-05 | 7.65E-05 |
| TAF1D | 4.25E+00 | 2.12E-05 | 7.68E-05 |
| DGKB | 4.25E+00 | 2.13E-05 | 7.71E-05 |
| SRPK3 | 4.25E+00 | 2.13E-05 | 7.71E-05 |
| FHOD3 | 4.25E+00 | 2.13E-05 | 7.73E-05 |
| LOC654342 | 4.25E+00 | 2.14E-05 | 7.76E-05 |
| SEMA7A | 4.25E+00 | 2.15E-05 | 7.79E-05 |
| TBC1D1 | 4.25E+00 | 2.16E-05 | 7.82E-05 |
| SCXB | 4.25E+00 | 2.16E-05 | 7.82E-05 |
| ZNF229 | 4.25E+00 | 2.16E-05 | 7.82E-05 |
| RETNLB | 4.25E+00 | 2.18E-05 | 7.90E-05 |
| CWF19L1 | 4.25E+00 | 2.19E-05 | 7.91E-05 |
| ZSWIM4 | 4.25E+00 | 2.19E-05 | 7.92E-05 |
| TTC4 | 4.25E+00 | 2.19E-05 | 7.92E-05 |
| CLPS | 4.24E+00 | 2.19E-05 | 7.93E-05 |
| PHF6 | 4.24E+00 | 2.21E-05 | 7.98E-05 |
| QPCTL | 4.24E+00 | 2.21E-05 | 8.00E-05 |
| INTS7 | 4.24E+00 | 2.22E-05 | 8.01E-05 |
| PPARD | 4.24E+00 | 2.22E-05 | 8.01E-05 |
| RFC1 | 4.24E+00 | 2.22E-05 | 8.01E-05 |
| ANO7 | 4.24E+00 | 2.23E-05 | 8.04E-05 |
| TFIP11 | 4.24E+00 | 2.26E-05 | 8.15E-05 |
| SLC6A6 | 4.24E+00 | 2.28E-05 | 8.21E-05 |
| OR1J4 | 4.24E+00 | 2.29E-05 | 8.26E-05 |
| NEU3 | 4.23E+00 | 2.29E-05 | 8.27E-05 |
| PATE2 | 4.23E+00 | 2.30E-05 | 8.28E-05 |
| RNF144A | 4.23E+00 | 2.30E-05 | 8.31E-05 |
| GAD2 | 4.23E+00 | 2.31E-05 | 8.33E-05 |

| NPB | 4.23E+00 | 2.33E-05 | 8.39E-05 |
| --- | --- | --- | --- |
| C19orf71 | 4.23E+00 | 2.33E-05 | 8.40E-05 |
| DLX6AS | 4.23E+00 | 2.34E-05 | 8.41E-05 |
| PPP2R2C | 4.23E+00 | 2.34E-05 | 8.43E-05 |
| MSH4 | 4.23E+00 | 2.35E-05 | 8.47E-05 |
| ZNF251 | 4.23E+00 | 2.35E-05 | 8.47E-05 |
| UNC5D | 4.23E+00 | 2.36E-05 | 8.50E-05 |
| CNTD2 | 4.23E+00 | 2.37E-05 | 8.52E-05 |
| C20orf118 | 4.23E+00 | 2.39E-05 | 8.59E-05 |
| PYGB | 4.22E+00 | 2.40E-05 | 8.63E-05 |
| CROCC | 4.22E+00 | 2.40E-05 | 8.63E-05 |
| LAMA1 | 4.22E+00 | 2.42E-05 | 8.70E-05 |
| IKBIP | 4.22E+00 | 2.43E-05 | 8.74E-05 |
| FKBP8 | 4.22E+00 | 2.43E-05 | 8.75E-05 |
| SLC2A7 | 4.22E+00 | 2.44E-05 | 8.78E-05 |
| RBM4 | 4.22E+00 | 2.48E-05 | 8.91E-05 |
| SLC26A1 | 4.22E+00 | 2.49E-05 | 8.94E-05 |
| DOCK7 | 4.22E+00 | 2.50E-05 | 8.98E-05 |
| PCDHA5 | 4.22E+00 | 2.50E-05 | 8.98E-05 |
| HIST1H3G | 4.21E+00 | 2.51E-05 | 9.00E-05 |
| GNB1 | 4.21E+00 | 2.51E-05 | 9.02E-05 |
| C17orf51 | 4.21E+00 | 2.52E-05 | 9.06E-05 |
| PCSK1 | 4.21E+00 | 2.53E-05 | 9.07E-05 |
| WIT1 | 4.21E+00 | 2.53E-05 | 9.08E-05 |
| C3orf21 | 4.21E+00 | 2.54E-05 | 9.10E-05 |
| METTL12 | 4.21E+00 | 2.55E-05 | 9.15E-05 |
| NETO2 | 4.21E+00 | 2.57E-05 | 9.23E-05 |
| TRIM17 | 4.21E+00 | 2.58E-05 | 9.24E-05 |
| TLR6 | 4.21E+00 | 2.59E-05 | 9.27E-05 |
| AMBRA1 | 4.21E+00 | 2.59E-05 | 9.28E-05 |
| MADD | 4.20E+00 | 2.62E-05 | 9.39E-05 |
| XPO7 | 4.20E+00 | 2.62E-05 | 9.40E-05 |
| SFXN5 | 4.20E+00 | 2.63E-05 | 9.43E-05 |
| WDR72 | 4.20E+00 | 2.63E-05 | 9.43E-05 |
| LOC150776 | 4.20E+00 | 2.64E-05 | 9.46E-05 |
| LHX5 | 4.20E+00 | 2.65E-05 | 9.48E-05 |
| COL16A1 | 4.20E+00 | 2.66E-05 | 9.51E-05 |
| SLC35D3 | 4.20E+00 | 2.69E-05 | 9.62E-05 |
| TYSND1 | 4.20E+00 | 2.69E-05 | 9.63E-05 |
| FSD1 | 4.20E+00 | 2.71E-05 | 9.70E-05 |
| LILRB2 | 4.20E+00 | 2.72E-05 | 9.72E-05 |
| LOC144486 | 4.20E+00 | 2.73E-05 | 9.77E-05 |
| INO80E | 4.19E+00 | 2.74E-05 | 9.78E-05 |
| L3MBTL | 4.19E+00 | 2.75E-05 | 9.83E-05 |
| KRT34 | 4.19E+00 | 2.75E-05 | 9.84E-05 |
| SULT1A3 | 4.19E+00 | 2.76E-05 | 9.87E-05 |
| UCKL1AS | 4.19E+00 | 2.78E-05 | 9.92E-05 |
| NUDT19 | 4.19E+00 | 2.78E-05 | 9.93E-05 |
| PEG10 | 4.19E+00 | 2.79E-05 | 9.96E-05 |
| HIST2H2AB | 4.19E+00 | 2.80E-05 | 1.00E-04 |
| NKX6-3 | 4.19E+00 | 2.81E-05 | 1.00E-04 |
| ITIH5L | 4.19E+00 | 2.82E-05 | 1.01E-04 |
| IGSF11 | 4.19E+00 | 2.83E-05 | 1.01E-04 |
| CDK8 | 4.19E+00 | 2.85E-05 | 1.02E-04 |
| PMPCA | 4.19E+00 | 2.85E-05 | 1.02E-04 |
| SLC5A5 | 4.18E+00 | 2.87E-05 | 1.02E-04 |
| ARHGEF2 | 4.18E+00 | 2.87E-05 | 1.03E-04 |

| PTPN1 | 4.18E+00 | 2.91E-05 | 1.04E-04 |
| --- | --- | --- | --- |
| CCDC136 | 4.18E+00 | 2.93E-05 | 1.05E-04 |
| HSPA14 | 4.18E+00 | 2.93E-05 | 1.05E-04 |
| NPFFR2 | 4.18E+00 | 2.94E-05 | 1.05E-04 |
| EEF1D | 4.18E+00 | 2.95E-05 | 1.05E-04 |
| COL5A1 | 4.18E+00 | 2.95E-05 | 1.05E-04 |
| NDUFS6 | 4.18E+00 | 2.95E-05 | 1.05E-04 |
| TRNP1 | 4.18E+00 | 2.96E-05 | 1.06E-04 |
| SUN3 | 4.18E+00 | 2.96E-05 | 1.06E-04 |
| TPH1 | 4.18E+00 | 2.97E-05 | 1.06E-04 |
| FOXP4 | 4.18E+00 | 2.97E-05 | 1.06E-04 |
| ANGPT2 | 4.17E+00 | 3.00E-05 | 1.07E-04 |
| COL6A2 | 4.17E+00 | 3.06E-05 | 1.09E-04 |
| DPY19L2P1 | 4.17E+00 | 3.06E-05 | 1.09E-04 |
| NCKIPSD | 4.17E+00 | 3.08E-05 | 1.09E-04 |
| CRAMP1L | 4.17E+00 | 3.10E-05 | 1.10E-04 |
| NRSN2 | 4.17E+00 | 3.10E-05 | 1.10E-04 |
| CARD18 | 4.17E+00 | 3.11E-05 | 1.10E-04 |
| SYT14 | 4.17E+00 | 3.11E-05 | 1.11E-04 |
| STAT1 | 4.17E+00 | 3.12E-05 | 1.11E-04 |
| ATG4D | 4.16E+00 | 3.14E-05 | 1.12E-04 |
| CIAPIN1 | 4.16E+00 | 3.16E-05 | 1.12E-04 |
| DNTTIP2 | 4.16E+00 | 3.17E-05 | 1.13E-04 |
| KCNG1 | 4.16E+00 | 3.17E-05 | 1.13E-04 |
| LOC441601 | 4.16E+00 | 3.19E-05 | 1.13E-04 |
| IKBKAP | 4.16E+00 | 3.19E-05 | 1.13E-04 |
| TSPY2 | 4.16E+00 | 3.20E-05 | 1.13E-04 |
| LENEP | 4.16E+00 | 3.22E-05 | 1.14E-04 |
| SLC32A1 | 4.16E+00 | 3.22E-05 | 1.14E-04 |
| KIAA1609 | 4.16E+00 | 3.24E-05 | 1.15E-04 |
| NR5A1 | 4.15E+00 | 3.27E-05 | 1.16E-04 |
| UBE2V1 | 4.15E+00 | 3.27E-05 | 1.16E-04 |
| CHRM4 | 4.15E+00 | 3.28E-05 | 1.16E-04 |
| SIGLEC10 | 4.15E+00 | 3.29E-05 | 1.17E-04 |
| ELAVL3 | 4.15E+00 | 3.31E-05 | 1.17E-04 |
| PRPF8 | 4.15E+00 | 3.31E-05 | 1.17E-04 |
| EIF2S3 | 4.15E+00 | 3.34E-05 | 1.19E-04 |
| NEURL3 | 4.15E+00 | 3.36E-05 | 1.19E-04 |
| BAHD1 | 4.15E+00 | 3.38E-05 | 1.20E-04 |
| IER3 | 4.15E+00 | 3.37E-05 | 1.20E-04 |
| OSR2 | 4.15E+00 | 3.39E-05 | 1.20E-04 |
| C8orf44 | 4.15E+00 | 3.40E-05 | 1.20E-04 |
| TCEB1 | 4.15E+00 | 3.39E-05 | 1.20E-04 |
| APEX1 | 4.15E+00 | 3.41E-05 | 1.21E-04 |
| KIAA1875 | 4.15E+00 | 3.40E-05 | 1.21E-04 |
| RRBP1 | 4.15E+00 | 3.41E-05 | 1.21E-04 |
| ANKIB1 | 4.14E+00 | 3.43E-05 | 1.21E-04 |
| SLC25A13 | 4.14E+00 | 3.44E-05 | 1.22E-04 |
| OPLAH | 4.14E+00 | 3.45E-05 | 1.22E-04 |
| DBX1 | 4.14E+00 | 3.46E-05 | 1.22E-04 |
| ZAR1L | 4.14E+00 | 3.46E-05 | 1.22E-04 |
| TUG1 | 4.14E+00 | 3.47E-05 | 1.23E-04 |
| CDKN2D | 4.14E+00 | 3.49E-05 | 1.23E-04 |
| SVOP | 4.14E+00 | 3.50E-05 | 1.24E-04 |
| USP31 | 4.14E+00 | 3.52E-05 | 1.24E-04 |
| KIAA1949 | 4.14E+00 | 3.53E-05 | 1.25E-04 |
| CXCL5 | 4.14E+00 | 3.54E-05 | 1.25E-04 |

| SIX3 | 4.13E+00 | 3.58E-05 | 1.26E-04 |
| --- | --- | --- | --- |
| KIAA1310 | 4.13E+00 | 3.59E-05 | 1.27E-04 |
| TRIM42 | 4.13E+00 | 3.60E-05 | 1.27E-04 |
| CNTROB | 4.13E+00 | 3.60E-05 | 1.27E-04 |
| BUD13 | 4.13E+00 | 3.61E-05 | 1.27E-04 |
| NDUFAF4 | 4.13E+00 | 3.61E-05 | 1.27E-04 |
| NLRP5 | 4.13E+00 | 3.63E-05 | 1.28E-04 |
| GOT1L1 | 4.13E+00 | 3.66E-05 | 1.29E-04 |
| GATSL2 | 4.13E+00 | 3.67E-05 | 1.29E-04 |
| ADO | 4.13E+00 | 3.68E-05 | 1.30E-04 |
| TBC1D3C | 4.13E+00 | 3.71E-05 | 1.31E-04 |
| NIP7 | 4.13E+00 | 3.71E-05 | 1.31E-04 |
| BCR | 4.12E+00 | 3.72E-05 | 1.31E-04 |
| C10orf62 | 4.12E+00 | 3.74E-05 | 1.32E-04 |
| SEC14L1 | 4.12E+00 | 3.74E-05 | 1.32E-04 |
| C17orf74 | 4.12E+00 | 3.75E-05 | 1.32E-04 |
| FRG2B | 4.12E+00 | 3.75E-05 | 1.32E-04 |
| RASSF1 | 4.12E+00 | 3.79E-05 | 1.34E-04 |
| DYRK1B | 4.12E+00 | 3.80E-05 | 1.34E-04 |
| FAM199X | 4.12E+00 | 3.84E-05 | 1.35E-04 |
| C14orf33 | 4.12E+00 | 3.85E-05 | 1.35E-04 |
| MRPL47 | 4.12E+00 | 3.85E-05 | 1.36E-04 |
| UBE2V2 | 4.12E+00 | 3.86E-05 | 1.36E-04 |
| ABHD8 | 4.12E+00 | 3.87E-05 | 1.36E-04 |
| TLX1 | 4.12E+00 | 3.87E-05 | 1.36E-04 |
| APPL2 | 4.11E+00 | 3.89E-05 | 1.37E-04 |
| CTCF | 4.11E+00 | 3.89E-05 | 1.37E-04 |
| C5orf58 | 4.11E+00 | 3.89E-05 | 1.37E-04 |
| OCRL | 4.11E+00 | 3.90E-05 | 1.37E-04 |
| G2E3 | 4.11E+00 | 3.90E-05 | 1.37E-04 |
| YLPM1 | 4.11E+00 | 3.93E-05 | 1.38E-04 |
| GNAZ | 4.11E+00 | 3.94E-05 | 1.38E-04 |
| FLJ12825 | 4.11E+00 | 3.95E-05 | 1.39E-04 |
| PLBD1 | 4.11E+00 | 3.95E-05 | 1.39E-04 |
| KBTBD2 | 4.11E+00 | 3.96E-05 | 1.39E-04 |
| PMS2L11 | 4.11E+00 | 3.98E-05 | 1.40E-04 |
| C11orf30 | 4.11E+00 | 3.99E-05 | 1.40E-04 |
| DEPDC4 | 4.11E+00 | 4.01E-05 | 1.41E-04 |
| C1orf131 | 4.11E+00 | 4.04E-05 | 1.42E-04 |
| TXNDC2 | 4.11E+00 | 4.04E-05 | 1.42E-04 |
| GSDMD | 4.11E+00 | 4.04E-05 | 1.42E-04 |
| PGBD1 | 4.11E+00 | 4.04E-05 | 1.42E-04 |
| LHX1 | 4.10E+00 | 4.07E-05 | 1.43E-04 |
| TUBA3C | 4.10E+00 | 4.08E-05 | 1.43E-04 |
| CUL9 | 4.10E+00 | 4.08E-05 | 1.43E-04 |
| RHOXF2B | 4.10E+00 | 4.09E-05 | 1.43E-04 |
| PJA1 | 4.10E+00 | 4.14E-05 | 1.45E-04 |
| SNORA8 | 4.10E+00 | 4.17E-05 | 1.46E-04 |
| FAM91A1 | 4.10E+00 | 4.20E-05 | 1.47E-04 |
| DNASE1L2 | 4.10E+00 | 4.23E-05 | 1.48E-04 |
| MICAL3 | 4.09E+00 | 4.27E-05 | 1.49E-04 |
| ZUFSP | 4.09E+00 | 4.32E-05 | 1.51E-04 |
| CCNB3 | 4.09E+00 | 4.34E-05 | 1.52E-04 |
| FAM75A2 | 4.09E+00 | 4.35E-05 | 1.52E-04 |
| CKMT1B | 4.09E+00 | 4.37E-05 | 1.53E-04 |
| PTCD3 | 4.09E+00 | 4.38E-05 | 1.53E-04 |
| MICAL1 | 4.09E+00 | 4.38E-05 | 1.53E-04 |

| LYPLA2P1 | 4.09E+00 | 4.39E-05 | 1.53E-04 |
| --- | --- | --- | --- |
| ZAN | 4.09E+00 | 4.40E-05 | 1.54E-04 |
| GLG1 | 4.09E+00 | 4.40E-05 | 1.54E-04 |
| RFPL4B | 4.09E+00 | 4.40E-05 | 1.54E-04 |
| VCX3B | 4.09E+00 | 4.41E-05 | 1.54E-04 |
| M6PR | 4.09E+00 | 4.41E-05 | 1.54E-04 |
| ETV4 | 4.08E+00 | 4.43E-05 | 1.55E-04 |
| ACPT | 4.08E+00 | 4.44E-05 | 1.55E-04 |
| RAB6B | 4.08E+00 | 4.46E-05 | 1.56E-04 |
| DNAJB6 | 4.08E+00 | 4.52E-05 | 1.58E-04 |
| EIF4G3 | 4.08E+00 | 4.52E-05 | 1.58E-04 |
| AFG3L2 | 4.08E+00 | 4.56E-05 | 1.59E-04 |
| BSN | 4.08E+00 | 4.57E-05 | 1.59E-04 |
| RPRM | 4.08E+00 | 4.59E-05 | 1.60E-04 |
| XYLT2 | 4.08E+00 | 4.61E-05 | 1.61E-04 |
| OR51E1 | 4.07E+00 | 4.63E-05 | 1.61E-04 |
| LINGO1 | 4.07E+00 | 4.63E-05 | 1.61E-04 |
| CA12 | 4.07E+00 | 4.65E-05 | 1.62E-04 |
| WDR92 | 4.07E+00 | 4.65E-05 | 1.62E-04 |
| OR51B5 | 4.07E+00 | 4.69E-05 | 1.63E-04 |
| CTNND2 | 4.07E+00 | 4.72E-05 | 1.64E-04 |
| SOHLH2 | 4.07E+00 | 4.75E-05 | 1.65E-04 |
| ZNF37A | 4.07E+00 | 4.75E-05 | 1.65E-04 |
| COL9A3 | 4.07E+00 | 4.76E-05 | 1.66E-04 |
| SFRP2 | 4.06E+00 | 4.82E-05 | 1.68E-04 |
| ADCY6 | 4.06E+00 | 4.86E-05 | 1.69E-04 |
| GPR111 | 4.06E+00 | 4.87E-05 | 1.69E-04 |
| BBC3 | 4.06E+00 | 4.87E-05 | 1.70E-04 |
| GABRR3 | 4.06E+00 | 4.90E-05 | 1.70E-04 |
| HRH3 | 4.06E+00 | 4.90E-05 | 1.70E-04 |
| CT45A6 | 4.06E+00 | 4.91E-05 | 1.70E-04 |
| KIAA1267 | 4.06E+00 | 4.91E-05 | 1.70E-04 |
| C12orf42 | 4.06E+00 | 4.94E-05 | 1.72E-04 |
| CMTM1 | 4.06E+00 | 4.94E-05 | 1.72E-04 |
| C6orf129 | 4.06E+00 | 4.94E-05 | 1.72E-04 |
| TYK2 | 4.06E+00 | 4.94E-05 | 1.72E-04 |
| AP2A2 | 4.06E+00 | 4.94E-05 | 1.72E-04 |
| C22orf29 | 4.06E+00 | 4.95E-05 | 1.72E-04 |
| SLC35B2 | 4.06E+00 | 4.96E-05 | 1.72E-04 |
| ZW10 | 4.06E+00 | 4.98E-05 | 1.73E-04 |
| WASH2P | 4.06E+00 | 5.01E-05 | 1.74E-04 |
| CLK3 | 4.06E+00 | 5.01E-05 | 1.74E-04 |
| ORC2L | 4.06E+00 | 5.01E-05 | 1.74E-04 |
| BRD2 | 4.05E+00 | 5.03E-05 | 1.75E-04 |
| CDRT1 | 4.05E+00 | 5.04E-05 | 1.75E-04 |
| WRN | 4.05E+00 | 5.05E-05 | 1.75E-04 |
| INSL4 | 4.05E+00 | 5.06E-05 | 1.75E-04 |
| GEMIN7 | 4.05E+00 | 5.06E-05 | 1.75E-04 |
| KIAA0528 | 4.05E+00 | 5.06E-05 | 1.75E-04 |
| FAM71F2 | 4.05E+00 | 5.11E-05 | 1.77E-04 |
| FAM75A5 | 4.05E+00 | 5.12E-05 | 1.77E-04 |
| GGH | 4.05E+00 | 5.13E-05 | 1.78E-04 |
| INO80B | 4.05E+00 | 5.13E-05 | 1.78E-04 |
| TRIM72 | 4.05E+00 | 5.13E-05 | 1.78E-04 |
| DHX15 | 4.05E+00 | 5.13E-05 | 1.78E-04 |
| BIK | 4.05E+00 | 5.17E-05 | 1.79E-04 |
| ARID1B | 4.05E+00 | 5.20E-05 | 1.80E-04 |

| HIST1H3H | 4.04E+00 | 5.28E-05 | 1.83E-04 |
| --- | --- | --- | --- |
| SLC47A2 | 4.04E+00 | 5.31E-05 | 1.84E-04 |
| CERCAM | 4.04E+00 | 5.31E-05 | 1.84E-04 |
| RNF168 | 4.04E+00 | 5.31E-05 | 1.84E-04 |
| C5orf30 | 4.04E+00 | 5.33E-05 | 1.84E-04 |
| SPTBN5 | 4.04E+00 | 5.37E-05 | 1.85E-04 |
| PIH1D1 | 4.04E+00 | 5.40E-05 | 1.86E-04 |
| TFAP2A | 4.04E+00 | 5.40E-05 | 1.87E-04 |
| GEMIN8P4 | 4.04E+00 | 5.42E-05 | 1.87E-04 |
| HAT1 | 4.04E+00 | 5.44E-05 | 1.88E-04 |
| GTF2E1 | 4.03E+00 | 5.48E-05 | 1.89E-04 |
| VPS54 | 4.03E+00 | 5.49E-05 | 1.89E-04 |
| KIAA1543 | 4.03E+00 | 5.50E-05 | 1.90E-04 |
| DFFA | 4.03E+00 | 5.52E-05 | 1.90E-04 |
| RAD23A | 4.03E+00 | 5.54E-05 | 1.91E-04 |
| C7orf70 | 4.03E+00 | 5.57E-05 | 1.92E-04 |
| MAP3K9 | 4.03E+00 | 5.57E-05 | 1.92E-04 |
| MAD2L2 | 4.03E+00 | 5.60E-05 | 1.93E-04 |
| RERE | 4.03E+00 | 5.62E-05 | 1.94E-04 |
| FBXL14 | 4.03E+00 | 5.63E-05 | 1.94E-04 |
| CEP192 | 4.03E+00 | 5.65E-05 | 1.95E-04 |
| FATE1 | 4.03E+00 | 5.65E-05 | 1.95E-04 |
| SGSM3 | 4.03E+00 | 5.66E-05 | 1.95E-04 |
| ALPK2 | 4.03E+00 | 5.67E-05 | 1.95E-04 |
| NUTF2 | 4.03E+00 | 5.68E-05 | 1.96E-04 |
| GLDC | 4.03E+00 | 5.69E-05 | 1.96E-04 |
| C19orf6 | 4.03E+00 | 5.70E-05 | 1.96E-04 |
| OXSR1 | 4.03E+00 | 5.71E-05 | 1.97E-04 |
| EMG1 | 4.02E+00 | 5.72E-05 | 1.97E-04 |
| JUP | 4.02E+00 | 5.74E-05 | 1.98E-04 |
| UBXN11 | 4.02E+00 | 5.78E-05 | 1.99E-04 |
| UHRF1BP1L | 4.02E+00 | 5.78E-05 | 1.99E-04 |
| KCTD19 | 4.02E+00 | 5.80E-05 | 1.99E-04 |
| SLC35F2 | 4.02E+00 | 5.82E-05 | 2.00E-04 |
| LRRC37A3 | 4.02E+00 | 5.83E-05 | 2.00E-04 |
| ATP1B3 | 4.02E+00 | 5.85E-05 | 2.01E-04 |
| NCRNA00162 | 4.02E+00 | 5.87E-05 | 2.02E-04 |
| RTBDN | 4.02E+00 | 5.89E-05 | 2.02E-04 |
| SYCE2 | 4.02E+00 | 5.90E-05 | 2.03E-04 |
| HSPA2 | 4.02E+00 | 5.91E-05 | 2.03E-04 |
| OSM | 4.02E+00 | 5.91E-05 | 2.03E-04 |
| ANKRD23 | 4.02E+00 | 5.93E-05 | 2.04E-04 |
| CDC42BPB | 4.02E+00 | 5.95E-05 | 2.04E-04 |
| INTS3 | 4.02E+00 | 5.95E-05 | 2.04E-04 |
| PHF21B | 4.01E+00 | 5.97E-05 | 2.05E-04 |
| PANK4 | 4.01E+00 | 5.99E-05 | 2.06E-04 |
| ZIC1 | 4.01E+00 | 6.03E-05 | 2.07E-04 |
| ADCY3 | 4.01E+00 | 6.04E-05 | 2.07E-04 |
| MLNR | 4.01E+00 | 6.05E-05 | 2.07E-04 |
| BAIAP2L1 | 4.01E+00 | 6.06E-05 | 2.08E-04 |
| TOMM40L | 4.01E+00 | 6.06E-05 | 2.08E-04 |
| TPRXL | 4.01E+00 | 6.08E-05 | 2.08E-04 |
| GBP5 | 4.01E+00 | 6.20E-05 | 2.12E-04 |
| RAD52 | 4.01E+00 | 6.21E-05 | 2.13E-04 |
| LOC554202 | 4.01E+00 | 6.21E-05 | 2.13E-04 |
| RASGEF1A | 4.00E+00 | 6.22E-05 | 2.13E-04 |
| HSP90AA1 | 4.00E+00 | 6.26E-05 | 2.14E-04 |

| MAK16 | 4.00E+00 | 6.29E-05 | 2.15E-04 |
| --- | --- | --- | --- |
| TYRO3 | 4.00E+00 | 6.29E-05 | 2.15E-04 |
| ZSCAN29 | 4.00E+00 | 6.31E-05 | 2.16E-04 |
| NDE1 | 4.00E+00 | 6.38E-05 | 2.18E-04 |
| PCDHGA1 | 4.00E+00 | 6.38E-05 | 2.18E-04 |
| DNAJA1 | 4.00E+00 | 6.40E-05 | 2.19E-04 |
| OTOP2 | 4.00E+00 | 6.44E-05 | 2.20E-04 |
| CCKAR | 4.00E+00 | 6.44E-05 | 2.20E-04 |
| CLCF1 | 4.00E+00 | 6.45E-05 | 2.21E-04 |
| TLX2 | 4.00E+00 | 6.47E-05 | 2.21E-04 |
| SLC34A3 | 3.99E+00 | 6.52E-05 | 2.23E-04 |
| PPFIA1 | 3.99E+00 | 6.54E-05 | 2.24E-04 |
| LOC256880 | 3.99E+00 | 6.55E-05 | 2.24E-04 |
| MLF2 | 3.99E+00 | 6.55E-05 | 2.24E-04 |
| ITGAX | 3.99E+00 | 6.57E-05 | 2.25E-04 |
| STAG3L2 | 3.99E+00 | 6.60E-05 | 2.26E-04 |
| FRMD5 | 3.99E+00 | 6.64E-05 | 2.27E-04 |
| ZSCAN5B | 3.99E+00 | 6.68E-05 | 2.28E-04 |
| ZMAT4 | 3.99E+00 | 6.69E-05 | 2.28E-04 |
| XPO1 | 3.99E+00 | 6.70E-05 | 2.29E-04 |
| SNAPC2 | 3.99E+00 | 6.71E-05 | 2.29E-04 |
| ASPSCR1 | 3.99E+00 | 6.72E-05 | 2.29E-04 |
| FLYWCH1 | 3.99E+00 | 6.72E-05 | 2.29E-04 |
| WNK4 | 3.98E+00 | 6.87E-05 | 2.34E-04 |
| KLHL36 | 3.98E+00 | 6.90E-05 | 2.35E-04 |
| IL17C | 3.98E+00 | 6.90E-05 | 2.35E-04 |
| RFXANK | 3.98E+00 | 6.91E-05 | 2.36E-04 |
| ASPHD2 | 3.98E+00 | 6.92E-05 | 2.36E-04 |
| USP49 | 3.98E+00 | 6.93E-05 | 2.36E-04 |
| KCNK12 | 3.98E+00 | 6.98E-05 | 2.38E-04 |
| SNCG | 3.98E+00 | 7.02E-05 | 2.39E-04 |
| NCRNA00204B | 3.98E+00 | 7.04E-05 | 2.40E-04 |
| RAET1K | 3.97E+00 | 7.06E-05 | 2.40E-04 |
| TMPRSS15 | 3.97E+00 | 7.08E-05 | 2.41E-04 |
| C20orf151 | 3.97E+00 | 7.11E-05 | 2.42E-04 |
| INSL6 | 3.97E+00 | 7.11E-05 | 2.42E-04 |
| RFX4 | 3.97E+00 | 7.12E-05 | 2.42E-04 |
| TPD52 | 3.97E+00 | 7.13E-05 | 2.43E-04 |
| GMPPB | 3.97E+00 | 7.14E-05 | 2.43E-04 |
| PDDC1 | 3.97E+00 | 7.20E-05 | 2.45E-04 |
| PGM3 | 3.97E+00 | 7.24E-05 | 2.46E-04 |
| CNPY2 | 3.97E+00 | 7.31E-05 | 2.48E-04 |
| APC2 | 3.97E+00 | 7.32E-05 | 2.49E-04 |
| LOC100133545 | 3.97E+00 | 7.35E-05 | 2.50E-04 |
| TTLL5 | 3.96E+00 | 7.41E-05 | 2.52E-04 |
| FAM113A | 3.96E+00 | 7.47E-05 | 2.54E-04 |
| COL2A1 | 3.96E+00 | 7.48E-05 | 2.54E-04 |
| GCNT3 | 3.96E+00 | 7.54E-05 | 2.56E-04 |
| SNORA76 | 3.96E+00 | 7.56E-05 | 2.57E-04 |
| MED17 | 3.96E+00 | 7.58E-05 | 2.57E-04 |
| KIAA0802 | 3.96E+00 | 7.58E-05 | 2.57E-04 |
| DOM3Z | 3.96E+00 | 7.59E-05 | 2.58E-04 |
| USH1G | 3.96E+00 | 7.59E-05 | 2.58E-04 |
| C8orf45 | 3.96E+00 | 7.63E-05 | 2.59E-04 |
| DULLARD | 3.96E+00 | 7.65E-05 | 2.60E-04 |
| DNAJA3 | 3.96E+00 | 7.65E-05 | 2.60E-04 |
| ARMC7 | 3.96E+00 | 7.67E-05 | 2.60E-04 |

| UTP15 | 3.95E+00 | 7.76E-05 | 2.63E-04 |
| --- | --- | --- | --- |
| KCND2 | 3.95E+00 | 7.79E-05 | 2.64E-04 |
| IDH3B | 3.95E+00 | 7.81E-05 | 2.65E-04 |
| OR51E2 | 3.95E+00 | 7.83E-05 | 2.65E-04 |
| DDX1 | 3.95E+00 | 7.83E-05 | 2.65E-04 |
| KRT6C | 3.95E+00 | 7.84E-05 | 2.66E-04 |
| MYPN | 3.95E+00 | 7.87E-05 | 2.67E-04 |
| COX10 | 3.95E+00 | 7.98E-05 | 2.70E-04 |
| C9orf4 | 3.94E+00 | 8.03E-05 | 2.72E-04 |
| NME2 | 3.94E+00 | 8.06E-05 | 2.73E-04 |
| SPDYE3 | 3.94E+00 | 8.09E-05 | 2.74E-04 |
| TSPY3 | 3.94E+00 | 8.11E-05 | 2.75E-04 |
| ASB16 | 3.94E+00 | 8.12E-05 | 2.75E-04 |
| MAGEA5 | 3.94E+00 | 8.13E-05 | 2.75E-04 |
| KRTAP6-3 | 3.94E+00 | 8.13E-05 | 2.75E-04 |
| AOC2 | 3.94E+00 | 8.18E-05 | 2.77E-04 |
| DDX18 | 3.94E+00 | 8.20E-05 | 2.77E-04 |
| NCRNA00120 | 3.94E+00 | 8.21E-05 | 2.78E-04 |
| HEXIM2 | 3.94E+00 | 8.22E-05 | 2.78E-04 |
| PTGFRN | 3.94E+00 | 8.23E-05 | 2.78E-04 |
| SGK223 | 3.94E+00 | 8.26E-05 | 2.79E-04 |
| ATP2A3 | 3.94E+00 | 8.29E-05 | 2.80E-04 |
| ARR3 | 3.94E+00 | 8.33E-05 | 2.81E-04 |
| CDRT15P | 3.93E+00 | 8.34E-05 | 2.82E-04 |
| OTP | 3.93E+00 | 8.36E-05 | 2.82E-04 |
| GDAP1 | 3.93E+00 | 8.37E-05 | 2.82E-04 |
| FAM53A | 3.93E+00 | 8.40E-05 | 2.84E-04 |
| GPR78 | 3.93E+00 | 8.41E-05 | 2.84E-04 |
| FANCM | 3.93E+00 | 8.43E-05 | 2.84E-04 |
| H2BFM | 3.93E+00 | 8.49E-05 | 2.86E-04 |
| PMS2CL | 3.93E+00 | 8.49E-05 | 2.87E-04 |
| OR51B2 | 3.93E+00 | 8.51E-05 | 2.87E-04 |
| HTR3A | 3.93E+00 | 8.52E-05 | 2.87E-04 |
| GDF11 | 3.93E+00 | 8.62E-05 | 2.91E-04 |
| NFYA | 3.93E+00 | 8.63E-05 | 2.91E-04 |
| PAQR6 | 3.93E+00 | 8.66E-05 | 2.92E-04 |
| BANF1 | 3.93E+00 | 8.66E-05 | 2.92E-04 |
| PABPC1P2 | 3.93E+00 | 8.67E-05 | 2.92E-04 |
| YY1AP1 | 3.93E+00 | 8.67E-05 | 2.92E-04 |
| CRY1 | 3.92E+00 | 8.70E-05 | 2.93E-04 |
| GNASAS | 3.92E+00 | 8.73E-05 | 2.94E-04 |
| DARS | 3.92E+00 | 8.77E-05 | 2.95E-04 |
| SLC22A8 | 3.92E+00 | 8.80E-05 | 2.96E-04 |
| TAC1 | 3.92E+00 | 8.85E-05 | 2.98E-04 |
| TMEM194B | 3.92E+00 | 8.86E-05 | 2.98E-04 |
| C9orf163 | 3.92E+00 | 8.87E-05 | 2.98E-04 |
| ZNF273 | 3.92E+00 | 8.87E-05 | 2.98E-04 |
| ADAP1 | 3.92E+00 | 8.89E-05 | 2.99E-04 |
| PASD1 | 3.92E+00 | 8.89E-05 | 2.99E-04 |
| REM2 | 3.92E+00 | 8.95E-05 | 3.01E-04 |
| GBA2 | 3.92E+00 | 9.00E-05 | 3.02E-04 |
| PYY | 3.91E+00 | 9.07E-05 | 3.05E-04 |
| KCNH2 | 3.91E+00 | 9.12E-05 | 3.06E-04 |
| CECR5 | 3.91E+00 | 9.19E-05 | 3.09E-04 |
| PDK1 | 3.91E+00 | 9.25E-05 | 3.11E-04 |
| LOC144438 | 3.91E+00 | 9.28E-05 | 3.12E-04 |
| DUSP14 | 3.91E+00 | 9.30E-05 | 3.12E-04 |

| MESDC1 | 3.91E+00 | 9.36E-05 | 3.14E-04 |
| --- | --- | --- | --- |
| NISCH | 3.91E+00 | 9.41E-05 | 3.16E-04 |
| CTNNBL1 | 3.90E+00 | 9.48E-05 | 3.18E-04 |
| KRTAP5-5 | 3.90E+00 | 9.52E-05 | 3.20E-04 |
| NCRNA00175 | 3.90E+00 | 9.53E-05 | 3.20E-04 |
| PTK2 | 3.90E+00 | 9.63E-05 | 3.23E-04 |
| ADAMTS12 | 3.90E+00 | 9.67E-05 | 3.24E-04 |
| SEC16A | 3.90E+00 | 9.83E-05 | 3.29E-04 |
| DNLZ | 3.89E+00 | 9.87E-05 | 3.31E-04 |
| KLK14 | 3.89E+00 | 9.88E-05 | 3.31E-04 |
| DSCR10 | 3.89E+00 | 9.89E-05 | 3.31E-04 |
| FLJ37201 | 3.89E+00 | 9.92E-05 | 3.32E-04 |
| C12orf36 | 3.89E+00 | 1.00E-04 | 3.35E-04 |
| NCF1C | 3.89E+00 | 1.01E-04 | 3.37E-04 |
| POGZ | 3.89E+00 | 1.01E-04 | 3.37E-04 |
| FLJ45340 | 3.89E+00 | 1.01E-04 | 3.38E-04 |
| NARF | 3.89E+00 | 1.01E-04 | 3.38E-04 |
| RUNDC1 | 3.89E+00 | 1.01E-04 | 3.39E-04 |
| TFAM | 3.89E+00 | 1.02E-04 | 3.40E-04 |
| PLA2G6 | 3.89E+00 | 1.02E-04 | 3.42E-04 |
| RPL32P3 | 3.89E+00 | 1.03E-04 | 3.43E-04 |
| POU2F2 | 3.88E+00 | 1.04E-04 | 3.47E-04 |
| TNPO1 | 3.88E+00 | 1.04E-04 | 3.48E-04 |
| DIP2A | 3.88E+00 | 1.04E-04 | 3.48E-04 |
| EPHA6 | 3.88E+00 | 1.04E-04 | 3.48E-04 |
| SNX32 | 3.88E+00 | 1.06E-04 | 3.53E-04 |
| SLC37A1 | 3.88E+00 | 1.06E-04 | 3.55E-04 |
| INHA | 3.88E+00 | 1.07E-04 | 3.56E-04 |
| KIAA0319 | 3.88E+00 | 1.07E-04 | 3.57E-04 |
| TDRD12 | 3.88E+00 | 1.07E-04 | 3.57E-04 |
| OR7C1 | 3.87E+00 | 1.07E-04 | 3.57E-04 |
| MRPL28 | 3.87E+00 | 1.07E-04 | 3.57E-04 |
| STX4 | 3.87E+00 | 1.07E-04 | 3.58E-04 |
| NFKBIE | 3.87E+00 | 1.08E-04 | 3.60E-04 |
| METTL2A | 3.87E+00 | 1.08E-04 | 3.61E-04 |
| MEFV | 3.87E+00 | 1.08E-04 | 3.62E-04 |
| HOXB7 | 3.87E+00 | 1.09E-04 | 3.62E-04 |
| NUP50 | 3.87E+00 | 1.09E-04 | 3.63E-04 |
| DCP1A | 3.87E+00 | 1.09E-04 | 3.64E-04 |
| CHKA | 3.87E+00 | 1.09E-04 | 3.64E-04 |
| EYA4 | 3.87E+00 | 1.10E-04 | 3.66E-04 |
| TNFAIP8L1 | 3.87E+00 | 1.10E-04 | 3.67E-04 |
| C3orf32 | 3.87E+00 | 1.11E-04 | 3.69E-04 |
| POFUT1 | 3.87E+00 | 1.11E-04 | 3.69E-04 |
| BATF3 | 3.87E+00 | 1.11E-04 | 3.70E-04 |
| HIST1H2AM | 3.87E+00 | 1.11E-04 | 3.71E-04 |
| LOC100190940 | 3.86E+00 | 1.11E-04 | 3.71E-04 |
| NPC1L1 | 3.86E+00 | 1.11E-04 | 3.71E-04 |
| C14orf115 | 3.86E+00 | 1.12E-04 | 3.74E-04 |
| CSRNP3 | 3.86E+00 | 1.12E-04 | 3.74E-04 |
| NDUFV1 | 3.86E+00 | 1.13E-04 | 3.75E-04 |
| SLC25A6 | 3.86E+00 | 1.13E-04 | 3.75E-04 |
| SNORA65 | 3.86E+00 | 1.13E-04 | 3.76E-04 |
| GRPEL2 | 3.86E+00 | 1.14E-04 | 3.78E-04 |
| C12orf45 | 3.86E+00 | 1.14E-04 | 3.79E-04 |
| ZCCHC11 | 3.86E+00 | 1.14E-04 | 3.80E-04 |
| HOXC12 | 3.86E+00 | 1.14E-04 | 3.81E-04 |

| ACOT11 | 3.86E+00 | 1.16E-04 | 3.85E-04 |
| --- | --- | --- | --- |
| KYNU | 3.85E+00 | 1.16E-04 | 3.86E-04 |
| IL2RA | 3.85E+00 | 1.17E-04 | 3.89E-04 |
| GTSF1 | 3.85E+00 | 1.18E-04 | 3.91E-04 |
| STRN | 3.85E+00 | 1.19E-04 | 3.95E-04 |
| PTF1A | 3.85E+00 | 1.19E-04 | 3.95E-04 |
| FOXP2 | 3.85E+00 | 1.19E-04 | 3.96E-04 |
| ISYNA1 | 3.85E+00 | 1.20E-04 | 3.98E-04 |
| CALB2 | 3.85E+00 | 1.20E-04 | 3.98E-04 |
| SLC22A11 | 3.85E+00 | 1.20E-04 | 3.98E-04 |
| STK10 | 3.85E+00 | 1.20E-04 | 3.98E-04 |
| RCN3 | 3.85E+00 | 1.20E-04 | 3.99E-04 |
| IL17REL | 3.84E+00 | 1.21E-04 | 4.01E-04 |
| TNNI3 | 3.84E+00 | 1.21E-04 | 4.01E-04 |
| RGS14 | 3.84E+00 | 1.21E-04 | 4.02E-04 |
| CBS | 3.84E+00 | 1.22E-04 | 4.03E-04 |
| C16orf42 | 3.84E+00 | 1.23E-04 | 4.07E-04 |
| U2AF1 | 3.84E+00 | 1.23E-04 | 4.08E-04 |
| HIST1H2BM | 3.84E+00 | 1.24E-04 | 4.11E-04 |
| TNFRSF9 | 3.84E+00 | 1.24E-04 | 4.12E-04 |
| MTHFSD | 3.84E+00 | 1.25E-04 | 4.12E-04 |
| PLCXD2 | 3.84E+00 | 1.25E-04 | 4.14E-04 |
| PTTG2 | 3.84E+00 | 1.25E-04 | 4.14E-04 |
| TRIM47 | 3.83E+00 | 1.26E-04 | 4.17E-04 |
| PSMD4 | 3.83E+00 | 1.26E-04 | 4.18E-04 |
| DHX36 | 3.83E+00 | 1.27E-04 | 4.19E-04 |
| FAM115A | 3.83E+00 | 1.27E-04 | 4.20E-04 |
| ROCK2 | 3.83E+00 | 1.27E-04 | 4.20E-04 |
| ISX | 3.83E+00 | 1.27E-04 | 4.21E-04 |
| HBA2 | -3.83E+00 | 1.28E-04 | 4.24E-04 |
| NEU4 | 3.83E+00 | 1.29E-04 | 4.26E-04 |
| SERPINH1 | 3.83E+00 | 1.29E-04 | 4.27E-04 |
| OGG1 | 3.83E+00 | 1.29E-04 | 4.28E-04 |
| ATP13A4 | -3.83E+00 | 1.29E-04 | 4.28E-04 |
| ZNF883 | 3.83E+00 | 1.30E-04 | 4.29E-04 |
| FRZB | -3.83E+00 | 1.30E-04 | 4.30E-04 |
| PLEKHN1 | 3.83E+00 | 1.30E-04 | 4.30E-04 |
| DCAF5 | -3.83E+00 | 1.30E-04 | 4.31E-04 |
| RABGGTA | 3.83E+00 | 1.30E-04 | 4.31E-04 |
| PDIA6 | 3.83E+00 | 1.31E-04 | 4.31E-04 |
| ITGB3 | -3.83E+00 | 1.31E-04 | 4.32E-04 |
| ITGA10 | -3.83E+00 | 1.31E-04 | 4.32E-04 |
| TEX264 | -3.83E+00 | 1.31E-04 | 4.32E-04 |
| DFNB59 | -3.83E+00 | 1.31E-04 | 4.32E-04 |
| CAPN11 | -3.82E+00 | 1.31E-04 | 4.33E-04 |
| SEZ6L | 3.82E+00 | 1.31E-04 | 4.33E-04 |
| HS2ST1 | 3.82E+00 | 1.32E-04 | 4.36E-04 |
| CTAGE1 | -3.82E+00 | 1.32E-04 | 4.36E-04 |
| VTI1A | 3.82E+00 | 1.33E-04 | 4.37E-04 |
| SCGB3A1 | -3.82E+00 | 1.33E-04 | 4.38E-04 |
| DNAJC6 | 3.82E+00 | 1.33E-04 | 4.39E-04 |
| NXF5 | 3.82E+00 | 1.33E-04 | 4.39E-04 |
| SLC16A2 | -3.82E+00 | 1.33E-04 | 4.40E-04 |
| PRPH | 3.82E+00 | 1.34E-04 | 4.40E-04 |
| PHLDA3 | -3.82E+00 | 1.34E-04 | 4.40E-04 |
| FKBP11 | 3.82E+00 | 1.34E-04 | 4.40E-04 |
| LACTB | -3.82E+00 | 1.34E-04 | 4.40E-04 |

| GPR83 | 3.82E+00 | 1.34E-04 | 4.41E-04 |
| --- | --- | --- | --- |
| GFI1 | 3.82E+00 | 1.34E-04 | 4.41E-04 |
| ORAI3 | -3.82E+00 | 1.34E-04 | 4.41E-04 |
| SSX3 | 3.82E+00 | 1.34E-04 | 4.42E-04 |
| UBAC1 | 3.82E+00 | 1.34E-04 | 4.42E-04 |
| FTSJ2 | 3.82E+00 | 1.35E-04 | 4.45E-04 |
| TMEM102 | 3.82E+00 | 1.35E-04 | 4.45E-04 |
| FGFBP2 | -3.82E+00 | 1.36E-04 | 4.46E-04 |
| LILRP2 | 3.82E+00 | 1.36E-04 | 4.47E-04 |
| METRN | -3.82E+00 | 1.36E-04 | 4.47E-04 |
| C7orf69 | 3.82E+00 | 1.36E-04 | 4.48E-04 |
| CES4 | -3.81E+00 | 1.37E-04 | 4.49E-04 |
| HSD17B1 | 3.81E+00 | 1.37E-04 | 4.50E-04 |
| BST1 | -3.81E+00 | 1.37E-04 | 4.50E-04 |
| OLIG3 | 3.81E+00 | 1.37E-04 | 4.50E-04 |
| TUBD1 | 3.81E+00 | 1.37E-04 | 4.50E-04 |
| GSTM2 | -3.81E+00 | 1.38E-04 | 4.51E-04 |
| MEGF6 | -3.81E+00 | 1.38E-04 | 4.52E-04 |
| BMP8A | 3.81E+00 | 1.38E-04 | 4.53E-04 |
| MAPK10 | -3.81E+00 | 1.38E-04 | 4.54E-04 |
| LOC147727 | -3.81E+00 | 1.38E-04 | 4.54E-04 |
| PCMTD1 | -3.81E+00 | 1.39E-04 | 4.55E-04 |
| TPM1 | -3.81E+00 | 1.39E-04 | 4.55E-04 |
| KCNIP4 | -3.81E+00 | 1.39E-04 | 4.56E-04 |
| PNN | 3.81E+00 | 1.39E-04 | 4.56E-04 |
| UPK1A | 3.81E+00 | 1.40E-04 | 4.58E-04 |
| RAB27A | -3.81E+00 | 1.40E-04 | 4.59E-04 |
| TERC | 3.81E+00 | 1.40E-04 | 4.59E-04 |
| RASGEF1C | 3.81E+00 | 1.40E-04 | 4.60E-04 |
| ZNF571 | -3.81E+00 | 1.40E-04 | 4.60E-04 |
| RPP21 | 3.81E+00 | 1.41E-04 | 4.60E-04 |
| ARHGEF16 | 3.81E+00 | 1.41E-04 | 4.60E-04 |
| HMGB1 | -3.81E+00 | 1.41E-04 | 4.62E-04 |
| BSDC1 | -3.81E+00 | 1.41E-04 | 4.62E-04 |
| C14orf93 | 3.81E+00 | 1.41E-04 | 4.62E-04 |
| RPS2P32 | 3.81E+00 | 1.42E-04 | 4.64E-04 |
| LPCAT1 | 3.81E+00 | 1.42E-04 | 4.64E-04 |
| MCAM | 3.80E+00 | 1.42E-04 | 4.65E-04 |
| LRIT3 | -3.80E+00 | 1.43E-04 | 4.66E-04 |
| PRCP | -3.80E+00 | 1.43E-04 | 4.68E-04 |
| DCDC2B | -3.80E+00 | 1.44E-04 | 4.71E-04 |
| ROD1 | 3.80E+00 | 1.44E-04 | 4.71E-04 |
| DPPA5 | 3.80E+00 | 1.45E-04 | 4.73E-04 |
| NPTN | -3.80E+00 | 1.45E-04 | 4.73E-04 |
| SLC16A6 | 3.80E+00 | 1.45E-04 | 4.74E-04 |
| C6orf211 | -3.80E+00 | 1.45E-04 | 4.74E-04 |
| NCK2 | 3.80E+00 | 1.46E-04 | 4.75E-04 |
| WNT11 | -3.80E+00 | 1.46E-04 | 4.75E-04 |
| THOC2 | 3.80E+00 | 1.46E-04 | 4.76E-04 |
| COX4I2 | -3.80E+00 | 1.46E-04 | 4.76E-04 |
| ZNF81 | 3.80E+00 | 1.46E-04 | 4.76E-04 |
| ACE2 | -3.80E+00 | 1.46E-04 | 4.76E-04 |
| ATP1A3 | 3.80E+00 | 1.46E-04 | 4.76E-04 |
| IFIT2 | -3.80E+00 | 1.46E-04 | 4.77E-04 |
| SDHAP2 | 3.80E+00 | 1.46E-04 | 4.77E-04 |
| DDX19B | -3.80E+00 | 1.47E-04 | 4.79E-04 |
| MARVELD2 | -3.80E+00 | 1.47E-04 | 4.80E-04 |

| TBC1D25 | 3.80E+00 | 1.47E-04 | 4.80E-04 |
| --- | --- | --- | --- |
| LPA | -3.80E+00 | 1.47E-04 | 4.80E-04 |
| GRID1 | -3.80E+00 | 1.47E-04 | 4.80E-04 |
| ASXL2 | 3.80E+00 | 1.48E-04 | 4.81E-04 |
| MYOZ1 | -3.79E+00 | 1.48E-04 | 4.82E-04 |
| AES | -3.79E+00 | 1.48E-04 | 4.84E-04 |
| TCF7L2 | -3.79E+00 | 1.48E-04 | 4.84E-04 |
| ACSM3 | -3.79E+00 | 1.49E-04 | 4.85E-04 |
| INVS | 3.79E+00 | 1.49E-04 | 4.85E-04 |
| PMM1 | -3.79E+00 | 1.50E-04 | 4.87E-04 |
| C21orf129 | 3.79E+00 | 1.50E-04 | 4.88E-04 |
| IFNGR1 | -3.79E+00 | 1.50E-04 | 4.89E-04 |
| VIPAR | -3.79E+00 | 1.50E-04 | 4.90E-04 |
| C2orf63 | -3.79E+00 | 1.51E-04 | 4.91E-04 |
| KRTAP19-5 | 3.79E+00 | 1.52E-04 | 4.93E-04 |
| SH2D3A | 3.79E+00 | 1.52E-04 | 4.93E-04 |
| DEPDC5 | 3.79E+00 | 1.52E-04 | 4.94E-04 |
| KGFLP2 | -3.79E+00 | 1.52E-04 | 4.94E-04 |
| ADAMTS19 | 3.79E+00 | 1.53E-04 | 4.97E-04 |
| MS4A13 | 3.79E+00 | 1.53E-04 | 4.98E-04 |
| ST7OT4 | 3.79E+00 | 1.53E-04 | 4.98E-04 |
| C14orf106 | 3.79E+00 | 1.53E-04 | 4.98E-04 |
| HIST1H4B | 3.79E+00 | 1.53E-04 | 4.98E-04 |
| MMP11 | 3.79E+00 | 1.54E-04 | 4.99E-04 |
| AGAP2 | 3.79E+00 | 1.54E-04 | 4.99E-04 |
| CHMP4B | 3.79E+00 | 1.54E-04 | 4.99E-04 |
| MGMT | -3.79E+00 | 1.54E-04 | 4.99E-04 |
| PSMC6 | -3.79E+00 | 1.54E-04 | 4.99E-04 |
| ZNF324B | 3.78E+00 | 1.54E-04 | 5.00E-04 |
| ALG2 | -3.78E+00 | 1.54E-04 | 5.01E-04 |
| MGST1 | -3.78E+00 | 1.55E-04 | 5.01E-04 |
| RNPC3 | -3.78E+00 | 1.55E-04 | 5.01E-04 |
| C16orf58 | -3.78E+00 | 1.55E-04 | 5.02E-04 |
| SLC6A7 | 3.78E+00 | 1.56E-04 | 5.05E-04 |
| UTP23 | 3.78E+00 | 1.56E-04 | 5.05E-04 |
| PCDHB8 | 3.78E+00 | 1.56E-04 | 5.05E-04 |
| EEFSEC | 3.78E+00 | 1.56E-04 | 5.05E-04 |
| ALKBH3 | -3.78E+00 | 1.56E-04 | 5.06E-04 |
| RRN3P3 | 3.78E+00 | 1.57E-04 | 5.07E-04 |
| TUBGCP4 | 3.78E+00 | 1.57E-04 | 5.08E-04 |
| UTS2R | 3.78E+00 | 1.57E-04 | 5.08E-04 |
| LOC255167 | -3.78E+00 | 1.57E-04 | 5.08E-04 |
| ZNF544 | 3.78E+00 | 1.57E-04 | 5.09E-04 |
| SERPINA1 | -3.78E+00 | 1.57E-04 | 5.09E-04 |
| LRRC41 | 3.78E+00 | 1.58E-04 | 5.10E-04 |
| TAF4B | 3.78E+00 | 1.58E-04 | 5.10E-04 |
| CMTM5 | -3.78E+00 | 1.59E-04 | 5.14E-04 |
| CXorf40B | 3.78E+00 | 1.59E-04 | 5.16E-04 |
| EFEMP1 | -3.78E+00 | 1.60E-04 | 5.18E-04 |
| PNLIPRP3 | -3.78E+00 | 1.60E-04 | 5.18E-04 |
| GPRC6A | 3.78E+00 | 1.60E-04 | 5.18E-04 |
| PDZD2 | -3.77E+00 | 1.61E-04 | 5.20E-04 |
| KCND1 | 3.77E+00 | 1.61E-04 | 5.21E-04 |
| C19orf57 | 3.77E+00 | 1.61E-04 | 5.21E-04 |
| RASL11B | -3.77E+00 | 1.61E-04 | 5.22E-04 |
| CDC42BPA | -3.77E+00 | 1.62E-04 | 5.22E-04 |
| HSPA4L | 3.77E+00 | 1.62E-04 | 5.22E-04 |

| SEC24D |  | -3.77E+00 | 1.63E-04 | 5.25E-04 |
| --- | --- | --- | --- | --- |
| PTPN9 |  | -3.77E+00 | 1.63E-04 | 5.27E-04 |
| RANBP9 |  | -3.77E+00 | 1.63E-04 | 5.27E-04 |
| ANKRD26 |  | 3.77E+00 | 1.63E-04 | 5.27E-04 |
| LYPD4 |  | 3.77E+00 | 1.63E-04 | 5.28E-04 |
| SH3BP2 |  | 3.77E+00 | 1.64E-04 | 5.28E-04 |
| ALAS2 |  | -3.77E+00 | 1.64E-04 | 5.30E-04 |
| FAM179B |  | -3.77E+00 | 1.64E-04 | 5.30E-04 |
| HEPHL1 |  | 3.77E+00 | 1.64E-04 | 5.30E-04 |
| PRPF3 |  | 3.77E+00 | 1.65E-04 | 5.31E-04 |
| PDGFA |  | -3.77E+00 | 1.65E-04 | 5.32E-04 |
| RAD54L2 |  | 3.77E+00 | 1.65E-04 | 5.33E-04 |
| HSPB2 |  | -3.77E+00 | 1.66E-04 | 5.34E-04 |
| CPA4 |  | 3.77E+00 | 1.66E-04 | 5.34E-04 |
| TLX3 |  | 3.77E+00 | 1.66E-04 | 5.35E-04 |
| GPS2 |  | 3.77E+00 | 1.66E-04 | 5.35E-04 |
| NCRNA00052 |  | 3.77E+00 | 1.66E-04 | 5.36E-04 |
| RP1L1 |  | 3.77E+00 | 1.67E-04 | 5.38E-04 |
|  | 10-Sep | -3.76E+00 | 1.67E-04 | 5.39E-04 |
| ROBLD3 |  | -3.76E+00 | 1.67E-04 | 5.39E-04 |
| CFDP1 |  | -3.76E+00 | 1.68E-04 | 5.40E-04 |
| TNFRSF6B |  | 3.76E+00 | 1.69E-04 | 5.43E-04 |
| BTBD6 |  | -3.76E+00 | 1.69E-04 | 5.43E-04 |
| HOOK2 |  | 3.76E+00 | 1.69E-04 | 5.45E-04 |
| SCAMP1 |  | -3.76E+00 | 1.70E-04 | 5.46E-04 |
| TM4SF4 |  | -3.76E+00 | 1.70E-04 | 5.46E-04 |
| STAMBPL1 |  | 3.76E+00 | 1.70E-04 | 5.46E-04 |
| DNAJB3 |  | 3.76E+00 | 1.70E-04 | 5.47E-04 |
| FAM173B |  | -3.76E+00 | 1.70E-04 | 5.48E-04 |
| WWOX |  | -3.76E+00 | 1.70E-04 | 5.48E-04 |
| CSMD3 |  | 3.76E+00 | 1.71E-04 | 5.48E-04 |
| TTC12 |  | -3.76E+00 | 1.71E-04 | 5.48E-04 |
| OR2J3 |  | 3.76E+00 | 1.71E-04 | 5.51E-04 |
| AP1G2 |  | 3.76E+00 | 1.72E-04 | 5.51E-04 |
| ATP6AP1 |  | 3.76E+00 | 1.72E-04 | 5.51E-04 |
| ZNF318 |  | 3.76E+00 | 1.72E-04 | 5.52E-04 |
| C6orf163 |  | 3.76E+00 | 1.72E-04 | 5.52E-04 |
| ARL4C |  | 3.76E+00 | 1.72E-04 | 5.52E-04 |
| ZC3H15 |  | 3.76E+00 | 1.72E-04 | 5.53E-04 |
| ELFN2 |  | -3.76E+00 | 1.72E-04 | 5.53E-04 |
| PTPN18 |  | -3.76E+00 | 1.73E-04 | 5.54E-04 |
| AGPHD1 |  | -3.76E+00 | 1.73E-04 | 5.55E-04 |
| ZNF433 |  | -3.76E+00 | 1.73E-04 | 5.55E-04 |
| DLST |  | -3.76E+00 | 1.73E-04 | 5.56E-04 |
| CDC14B |  | -3.76E+00 | 1.74E-04 | 5.56E-04 |
| TMEM170B |  | -3.75E+00 | 1.75E-04 | 5.60E-04 |
| CALCA |  | 3.75E+00 | 1.76E-04 | 5.63E-04 |
| IL1RL1 |  | -3.75E+00 | 1.76E-04 | 5.63E-04 |
| HIST1H2AB |  | 3.75E+00 | 1.76E-04 | 5.64E-04 |
| CD300E |  | 3.75E+00 | 1.76E-04 | 5.64E-04 |
| EIF3H |  | 3.75E+00 | 1.78E-04 | 5.71E-04 |
| PCDP1 |  | -3.75E+00 | 1.78E-04 | 5.71E-04 |
| NINL |  | 3.75E+00 | 1.79E-04 | 5.74E-04 |
| TIMM8A |  | 3.75E+00 | 1.79E-04 | 5.75E-04 |
| NGF |  | -3.75E+00 | 1.80E-04 | 5.75E-04 |
| C1orf133 |  | -3.75E+00 | 1.80E-04 | 5.75E-04 |
| RPS19BP1 |  | 3.75E+00 | 1.80E-04 | 5.75E-04 |

| CCDC107 | -3.75E+00 | 1.80E-04 | 5.77E-04 |
| --- | --- | --- | --- |
| MGC12916 | 3.74E+00 | 1.81E-04 | 5.79E-04 |
| CYP4F12 | -3.74E+00 | 1.81E-04 | 5.79E-04 |
| LINGO2 | 3.74E+00 | 1.81E-04 | 5.80E-04 |
| WIPI2 | 3.74E+00 | 1.81E-04 | 5.80E-04 |
| WNT9A | 3.74E+00 | 1.82E-04 | 5.80E-04 |
| FAM22G | 3.74E+00 | 1.82E-04 | 5.82E-04 |
| C6orf62 | -3.74E+00 | 1.82E-04 | 5.83E-04 |
| SCRT1 | 3.74E+00 | 1.83E-04 | 5.83E-04 |
| C7orf10 | -3.74E+00 | 1.83E-04 | 5.84E-04 |
| DPAGT1 | 3.74E+00 | 1.83E-04 | 5.84E-04 |
| MGC21881 | -3.74E+00 | 1.86E-04 | 5.95E-04 |
| CLIC1 | 3.74E+00 | 1.86E-04 | 5.95E-04 |
| ANKRD12 | -3.74E+00 | 1.86E-04 | 5.95E-04 |
| HLA-C | -3.74E+00 | 1.87E-04 | 5.95E-04 |
| PM20D2 | 3.74E+00 | 1.87E-04 | 5.97E-04 |
| CLDN2 | -3.74E+00 | 1.87E-04 | 5.97E-04 |
| RC3H1 | -3.74E+00 | 1.87E-04 | 5.98E-04 |
| IFI44 | -3.74E+00 | 1.88E-04 | 5.98E-04 |
| UGT2B15 | -3.74E+00 | 1.88E-04 | 5.99E-04 |
| MGC34034 | 3.74E+00 | 1.88E-04 | 5.99E-04 |
| POLR3C | 3.74E+00 | 1.88E-04 | 5.99E-04 |
| SRA1 | -3.73E+00 | 1.88E-04 | 6.00E-04 |
| CDS2 | -3.73E+00 | 1.89E-04 | 6.01E-04 |
| REC8 | 3.73E+00 | 1.89E-04 | 6.01E-04 |
| SPATA21 | 3.73E+00 | 1.89E-04 | 6.01E-04 |
| JAKMIP3 | 3.73E+00 | 1.89E-04 | 6.02E-04 |
| ZNF254 | -3.73E+00 | 1.89E-04 | 6.02E-04 |
| TSC22D1 | -3.73E+00 | 1.89E-04 | 6.03E-04 |
| TXNL4A | 3.73E+00 | 1.91E-04 | 6.07E-04 |
| ZNF443 | -3.73E+00 | 1.91E-04 | 6.08E-04 |
| BBS4 | -3.73E+00 | 1.92E-04 | 6.11E-04 |
| CLCC1 | -3.73E+00 | 1.92E-04 | 6.12E-04 |
| FIGNL2 | 3.73E+00 | 1.92E-04 | 6.12E-04 |
| TPRKB | 3.73E+00 | 1.93E-04 | 6.13E-04 |
| GALNT4 | 3.73E+00 | 1.93E-04 | 6.14E-04 |
| CHAD | -3.73E+00 | 1.93E-04 | 6.14E-04 |
| MAGEC3 | 3.73E+00 | 1.93E-04 | 6.14E-04 |
| C10orf57 | -3.73E+00 | 1.93E-04 | 6.14E-04 |
| PAK1 | 3.73E+00 | 1.93E-04 | 6.15E-04 |
| KIAA1024 | 3.73E+00 | 1.94E-04 | 6.15E-04 |
| PDXK | 3.73E+00 | 1.94E-04 | 6.17E-04 |
| PPP2CB | -3.73E+00 | 1.94E-04 | 6.17E-04 |
| UBXN8 | -3.73E+00 | 1.94E-04 | 6.17E-04 |
| BMP1 | 3.73E+00 | 1.94E-04 | 6.18E-04 |
| GRINL1A | -3.73E+00 | 1.94E-04 | 6.18E-04 |
| PGK1 | 3.73E+00 | 1.95E-04 | 6.19E-04 |
| CHGB | 3.73E+00 | 1.95E-04 | 6.20E-04 |
| CYP20A1 | -3.73E+00 | 1.95E-04 | 6.20E-04 |
| C16orf63 | -3.72E+00 | 1.97E-04 | 6.25E-04 |
| FBXW5 | 3.72E+00 | 1.97E-04 | 6.26E-04 |
| PRDM6 | -3.72E+00 | 1.97E-04 | 6.27E-04 |
| TUBAL3 | 3.72E+00 | 1.98E-04 | 6.27E-04 |
| KCMF1 | 3.72E+00 | 1.98E-04 | 6.29E-04 |
| OR2AG2 | 3.72E+00 | 1.99E-04 | 6.30E-04 |
| C9orf9 | -3.72E+00 | 1.99E-04 | 6.30E-04 |
| THRA | -3.72E+00 | 1.99E-04 | 6.30E-04 |

| SYNRG |  | 3.72E+00 | 2.00E-04 | 6.33E-04 |
| --- | --- | --- | --- | --- |
| JMJD7-PLA2G4B |  | -3.72E+00 | 2.00E-04 | 6.35E-04 |
| LMF2 |  | 3.72E+00 | 2.00E-04 | 6.35E-04 |
| GP9 |  | -3.72E+00 | 2.01E-04 | 6.36E-04 |
| QDPR |  | -3.72E+00 | 2.01E-04 | 6.38E-04 |
| MICB |  | 3.72E+00 | 2.02E-04 | 6.39E-04 |
| POU4F1 |  | 3.72E+00 | 2.02E-04 | 6.39E-04 |
| STX3 |  | 3.72E+00 | 2.02E-04 | 6.40E-04 |
| NAGA |  | -3.72E+00 | 2.03E-04 | 6.41E-04 |
| CLASP1 |  | 3.72E+00 | 2.03E-04 | 6.41E-04 |
| FCHO2 |  | -3.72E+00 | 2.03E-04 | 6.41E-04 |
| DUS4L |  | 3.72E+00 | 2.03E-04 | 6.43E-04 |
| C8orf42 |  | -3.72E+00 | 2.03E-04 | 6.43E-04 |
| UPRT |  | -3.72E+00 | 2.04E-04 | 6.44E-04 |
| TMC2 |  | 3.71E+00 | 2.04E-04 | 6.44E-04 |
| FAM83F |  | 3.71E+00 | 2.04E-04 | 6.46E-04 |
| MEAF6 |  | -3.71E+00 | 2.05E-04 | 6.47E-04 |
| UPB1 |  | -3.71E+00 | 2.05E-04 | 6.48E-04 |
| TSPO2 |  | 3.71E+00 | 2.05E-04 | 6.49E-04 |
|  | 6-Mar | 3.71E+00 | 2.05E-04 | 6.49E-04 |
| KDELR1 |  | 3.71E+00 | 2.05E-04 | 6.49E-04 |
| PVRL2 |  | 3.71E+00 | 2.06E-04 | 6.50E-04 |
| ITM2A |  | -3.71E+00 | 2.06E-04 | 6.52E-04 |
| MRPS31 |  | -3.71E+00 | 2.06E-04 | 6.52E-04 |
| LOC126536 |  | 3.71E+00 | 2.07E-04 | 6.53E-04 |
| ST3GAL4 |  | 3.71E+00 | 2.07E-04 | 6.54E-04 |
| SLC25A46 |  | -3.71E+00 | 2.07E-04 | 6.54E-04 |
| NAA10 |  | 3.71E+00 | 2.08E-04 | 6.55E-04 |
| PPP3CC |  | -3.71E+00 | 2.08E-04 | 6.56E-04 |
| VCAN |  | 3.71E+00 | 2.08E-04 | 6.57E-04 |
| CROT |  | -3.71E+00 | 2.08E-04 | 6.58E-04 |
| HINT3 |  | -3.71E+00 | 2.08E-04 | 6.58E-04 |
| BET1 |  | -3.71E+00 | 2.09E-04 | 6.58E-04 |
| PUS7L |  | 3.71E+00 | 2.09E-04 | 6.58E-04 |
| ORC5L |  | 3.71E+00 | 2.09E-04 | 6.59E-04 |
| FAHD1 |  | -3.71E+00 | 2.10E-04 | 6.61E-04 |
| C6orf155 |  | -3.71E+00 | 2.10E-04 | 6.62E-04 |
| GIMAP8 |  | -3.71E+00 | 2.10E-04 | 6.63E-04 |
| ADH6 |  | -3.71E+00 | 2.11E-04 | 6.66E-04 |
| UGT1A10 |  | 3.71E+00 | 2.12E-04 | 6.67E-04 |
| COIL |  | 3.71E+00 | 2.12E-04 | 6.67E-04 |
| PGM2L1 |  | 3.70E+00 | 2.12E-04 | 6.69E-04 |
| BLCAP |  | -3.70E+00 | 2.12E-04 | 6.69E-04 |
| ZNF326 |  | 3.70E+00 | 2.13E-04 | 6.70E-04 |
| IRF7 |  | 3.70E+00 | 2.13E-04 | 6.71E-04 |
| PRDX3 |  | -3.70E+00 | 2.13E-04 | 6.71E-04 |
| ADM |  | 3.70E+00 | 2.14E-04 | 6.72E-04 |
| CYP2C18 |  | -3.70E+00 | 2.14E-04 | 6.74E-04 |
| ZNF446 |  | 3.70E+00 | 2.15E-04 | 6.75E-04 |
| CEBPG |  | 3.70E+00 | 2.15E-04 | 6.77E-04 |
| TMEM51 |  | 3.70E+00 | 2.16E-04 | 6.79E-04 |
| HSPA6 |  | 3.70E+00 | 2.16E-04 | 6.79E-04 |
| FAM66D |  | -3.70E+00 | 2.16E-04 | 6.80E-04 |
| ACBD7 |  | 3.70E+00 | 2.16E-04 | 6.80E-04 |
| ACTRT1 |  | 3.70E+00 | 2.16E-04 | 6.80E-04 |
| INO80 |  | 3.70E+00 | 2.17E-04 | 6.82E-04 |
| RAB5C |  | 3.70E+00 | 2.17E-04 | 6.83E-04 |

| ACER1 | -3.70E+00 | 2.18E-04 | 6.83E-04 |
| --- | --- | --- | --- |
| FAM46A | -3.70E+00 | 2.18E-04 | 6.85E-04 |
| GPR144 | 3.70E+00 | 2.18E-04 | 6.85E-04 |
| ACCN4 | 3.70E+00 | 2.19E-04 | 6.87E-04 |
| ZNF618 | 3.70E+00 | 2.19E-04 | 6.87E-04 |
| N4BP2L2 | -3.70E+00 | 2.19E-04 | 6.88E-04 |
| FAM184B | 3.70E+00 | 2.19E-04 | 6.88E-04 |
| PGK2 | 3.70E+00 | 2.19E-04 | 6.88E-04 |
| PPP1CA | 3.70E+00 | 2.19E-04 | 6.88E-04 |
| METTL14 | -3.70E+00 | 2.20E-04 | 6.89E-04 |
| TMEM160 | 3.70E+00 | 2.20E-04 | 6.89E-04 |
| PPP1R13L | 3.70E+00 | 2.20E-04 | 6.90E-04 |
| NVL | 3.70E+00 | 2.20E-04 | 6.90E-04 |
| JPH3 | 3.70E+00 | 2.20E-04 | 6.90E-04 |
| WDYHV1 | 3.69E+00 | 2.21E-04 | 6.91E-04 |
| SCAPER | -3.69E+00 | 2.21E-04 | 6.91E-04 |
| MPP4 | 3.69E+00 | 2.21E-04 | 6.92E-04 |
| PRKAB1 | 3.69E+00 | 2.21E-04 | 6.93E-04 |
| STX16 | 3.69E+00 | 2.22E-04 | 6.95E-04 |
| TAP1 | 3.69E+00 | 2.23E-04 | 6.98E-04 |
| ZKSCAN1 | 3.69E+00 | 2.23E-04 | 6.99E-04 |
| PPP2R4 | 3.69E+00 | 2.23E-04 | 7.00E-04 |
| SECTM1 | 3.69E+00 | 2.24E-04 | 7.01E-04 |
| GIMAP4 | -3.69E+00 | 2.24E-04 | 7.01E-04 |
| BICD2 | 3.69E+00 | 2.24E-04 | 7.02E-04 |
| CCDC69 | -3.69E+00 | 2.26E-04 | 7.06E-04 |
| SYT13 | 3.69E+00 | 2.26E-04 | 7.06E-04 |
| THEG | 3.69E+00 | 2.27E-04 | 7.08E-04 |
| RNF121 | 3.69E+00 | 2.27E-04 | 7.10E-04 |
| PCDHA1 | 3.69E+00 | 2.27E-04 | 7.10E-04 |
| LHX8 | 3.69E+00 | 2.28E-04 | 7.11E-04 |
| SUOX | -3.69E+00 | 2.28E-04 | 7.12E-04 |
| SLC27A3 | -3.69E+00 | 2.29E-04 | 7.16E-04 |
| NSMAF | 3.68E+00 | 2.30E-04 | 7.19E-04 |
| C3orf72 | 3.68E+00 | 2.30E-04 | 7.20E-04 |
| IL28B | 3.68E+00 | 2.31E-04 | 7.21E-04 |
| AIM2 | 3.68E+00 | 2.31E-04 | 7.21E-04 |
| ZBTB44 | -3.68E+00 | 2.31E-04 | 7.21E-04 |
| PRKCD | 3.68E+00 | 2.31E-04 | 7.22E-04 |
| SACM1L | -3.68E+00 | 2.32E-04 | 7.23E-04 |
| MANEA | -3.68E+00 | 2.32E-04 | 7.23E-04 |
| VPS36 | -3.68E+00 | 2.32E-04 | 7.24E-04 |
| LATS1 | 3.68E+00 | 2.32E-04 | 7.24E-04 |
| RELL1 | -3.68E+00 | 2.32E-04 | 7.25E-04 |
| NPY1R | -3.68E+00 | 2.32E-04 | 7.25E-04 |
| RPP14 | -3.68E+00 | 2.34E-04 | 7.29E-04 |
| RASA4 | 3.68E+00 | 2.34E-04 | 7.29E-04 |
| SYNGAP1 | 3.68E+00 | 2.34E-04 | 7.30E-04 |
| HYMAI | 3.68E+00 | 2.34E-04 | 7.30E-04 |
| MATN1 | 3.68E+00 | 2.34E-04 | 7.30E-04 |
| B3GNT1 | -3.68E+00 | 2.35E-04 | 7.31E-04 |
| LRRC52 | -3.68E+00 | 2.34E-04 | 7.31E-04 |
| ZNF649 | -3.68E+00 | 2.35E-04 | 7.31E-04 |
| ARHGEF12 | -3.68E+00 | 2.35E-04 | 7.31E-04 |
| TUBE1 | -3.68E+00 | 2.35E-04 | 7.31E-04 |
| SMCHD1 | 3.68E+00 | 2.35E-04 | 7.31E-04 |
| COL1A2 | 3.68E+00 | 2.35E-04 | 7.32E-04 |

| TRIM54 | 3.68E+00 | 2.37E-04 | 7.36E-04 |
| --- | --- | --- | --- |
| CEP97 | 3.68E+00 | 2.37E-04 | 7.37E-04 |
| ASRGL1 | 3.68E+00 | 2.37E-04 | 7.37E-04 |
| NUP210L | -3.68E+00 | 2.37E-04 | 7.37E-04 |
| LOC339290 | -3.68E+00 | 2.37E-04 | 7.39E-04 |
| KIAA0776 | -3.68E+00 | 2.38E-04 | 7.39E-04 |
| PAX9 | 3.68E+00 | 2.38E-04 | 7.41E-04 |
| SSFA2 | -3.68E+00 | 2.38E-04 | 7.41E-04 |
| PRKAA1 | -3.68E+00 | 2.38E-04 | 7.41E-04 |
| XPC | -3.67E+00 | 2.39E-04 | 7.43E-04 |
| NRBF2 | -3.67E+00 | 2.40E-04 | 7.45E-04 |
| MGC42105 | -3.67E+00 | 2.40E-04 | 7.46E-04 |
| CFB | -3.67E+00 | 2.40E-04 | 7.47E-04 |
| ZCWPW2 | -3.67E+00 | 2.41E-04 | 7.47E-04 |
| RELN | 3.67E+00 | 2.41E-04 | 7.48E-04 |
| OR52E2 | 3.67E+00 | 2.41E-04 | 7.48E-04 |
| EFHA1 | -3.67E+00 | 2.41E-04 | 7.49E-04 |
| LUZP2 | -3.67E+00 | 2.42E-04 | 7.51E-04 |
| SUCNR1 | -3.67E+00 | 2.43E-04 | 7.53E-04 |
| CYTH2 | 3.67E+00 | 2.43E-04 | 7.54E-04 |
| CYR61 | -3.67E+00 | 2.43E-04 | 7.54E-04 |
| HEMGN | -3.67E+00 | 2.43E-04 | 7.54E-04 |
| PCDHGB1 | 3.67E+00 | 2.43E-04 | 7.54E-04 |
| MFAP3L | -3.67E+00 | 2.44E-04 | 7.55E-04 |
| ERGIC1 | -3.67E+00 | 2.44E-04 | 7.56E-04 |
| SDC4 | -3.67E+00 | 2.44E-04 | 7.57E-04 |
| FOXI2 | -3.67E+00 | 2.46E-04 | 7.62E-04 |
| GPR45 | 3.67E+00 | 2.46E-04 | 7.63E-04 |
| UCP2 | 3.67E+00 | 2.47E-04 | 7.64E-04 |
| TAC3 | 3.67E+00 | 2.47E-04 | 7.65E-04 |
| C7orf13 | 3.67E+00 | 2.47E-04 | 7.65E-04 |
| GPR155 | -3.66E+00 | 2.48E-04 | 7.68E-04 |
| DMBX1 | 3.66E+00 | 2.48E-04 | 7.68E-04 |
| TMEM200C | 3.66E+00 | 2.48E-04 | 7.69E-04 |
| TULP2 | 3.66E+00 | 2.49E-04 | 7.69E-04 |
| CD109 | 3.66E+00 | 2.49E-04 | 7.71E-04 |
| DBC1 | -3.66E+00 | 2.49E-04 | 7.71E-04 |
| GHITM | -3.66E+00 | 2.50E-04 | 7.73E-04 |
| POLB | -3.66E+00 | 2.51E-04 | 7.76E-04 |
| COLEC10 | -3.66E+00 | 2.51E-04 | 7.76E-04 |
| CPT1C | 3.66E+00 | 2.52E-04 | 7.80E-04 |
| ITPR3 | 3.66E+00 | 2.53E-04 | 7.81E-04 |
| DAO | -3.66E+00 | 2.53E-04 | 7.82E-04 |
| CYB5RL | 3.66E+00 | 2.54E-04 | 7.85E-04 |
| CUL3 | -3.66E+00 | 2.54E-04 | 7.86E-04 |
| BTBD17 | 3.66E+00 | 2.55E-04 | 7.87E-04 |
| GTF3C4 | 3.66E+00 | 2.55E-04 | 7.88E-04 |
| BAG1 | -3.66E+00 | 2.55E-04 | 7.88E-04 |
| AZGP1 | -3.66E+00 | 2.55E-04 | 7.88E-04 |
| CHI3L1 | -3.66E+00 | 2.55E-04 | 7.88E-04 |
| CYP11A1 | -3.66E+00 | 2.55E-04 | 7.88E-04 |
| FAM19A1 | -3.66E+00 | 2.55E-04 | 7.88E-04 |
| LYPD1 | 3.66E+00 | 2.55E-04 | 7.88E-04 |
| XKR7 | 3.66E+00 | 2.55E-04 | 7.88E-04 |
| LOC220429 | -3.66E+00 | 2.57E-04 | 7.93E-04 |
| SHOC2 | -3.66E+00 | 2.57E-04 | 7.93E-04 |
| ATCAY | 3.66E+00 | 2.57E-04 | 7.93E-04 |

| PPP6C | -3.65E+00 | 2.59E-04 | 7.98E-04 |
| --- | --- | --- | --- |
| GADD45GIP1 | 3.65E+00 | 2.59E-04 | 8.00E-04 |
| C10orf90 | 3.65E+00 | 2.59E-04 | 8.00E-04 |
| ARHGEF9 | -3.65E+00 | 2.59E-04 | 8.00E-04 |
| ZNF197 | -3.65E+00 | 2.60E-04 | 8.00E-04 |
| LOC100270746 | -3.65E+00 | 2.60E-04 | 8.01E-04 |
| MRPL11 | 3.65E+00 | 2.60E-04 | 8.01E-04 |
| SLC12A5 | 3.65E+00 | 2.61E-04 | 8.05E-04 |
| AMZ1 | 3.65E+00 | 2.63E-04 | 8.11E-04 |
| GNRHR2 | -3.65E+00 | 2.64E-04 | 8.14E-04 |
| WIPF3 | 3.65E+00 | 2.65E-04 | 8.16E-04 |
| ADAM22 | 3.65E+00 | 2.65E-04 | 8.16E-04 |
| SLCO6A1 | 3.65E+00 | 2.66E-04 | 8.18E-04 |
| COG5 | 3.65E+00 | 2.66E-04 | 8.18E-04 |
| KDM4A | 3.65E+00 | 2.66E-04 | 8.19E-04 |
| ATXN7 | -3.65E+00 | 2.66E-04 | 8.20E-04 |
| MYLK4 | -3.65E+00 | 2.67E-04 | 8.21E-04 |
| SNX18 | -3.65E+00 | 2.67E-04 | 8.22E-04 |
| FLOT2 | 3.65E+00 | 2.67E-04 | 8.22E-04 |
| MMRN1 | -3.65E+00 | 2.68E-04 | 8.24E-04 |
| TAAR8 | 3.64E+00 | 2.68E-04 | 8.24E-04 |
| WFDC1 | -3.64E+00 | 2.68E-04 | 8.24E-04 |
| CPAMD8 | -3.64E+00 | 2.69E-04 | 8.25E-04 |
| DHX40 | -3.64E+00 | 2.69E-04 | 8.26E-04 |
| KIAA1797 | 3.64E+00 | 2.69E-04 | 8.26E-04 |
| C19orf45 | 3.64E+00 | 2.70E-04 | 8.29E-04 |
| WDR88 | 3.64E+00 | 2.70E-04 | 8.31E-04 |
| DKFZp686O24166 | 3.64E+00 | 2.71E-04 | 8.31E-04 |
| PINK1 | -3.64E+00 | 2.71E-04 | 8.33E-04 |
| SH2D7 | 3.64E+00 | 2.72E-04 | 8.34E-04 |
| C1orf109 | 3.64E+00 | 2.73E-04 | 8.38E-04 |
| NFKBIA | -3.64E+00 | 2.73E-04 | 8.38E-04 |
| KRT6B | 3.64E+00 | 2.75E-04 | 8.42E-04 |
| PLA2G12B | -3.64E+00 | 2.75E-04 | 8.44E-04 |
| ATP1A2 | -3.64E+00 | 2.75E-04 | 8.44E-04 |
| FAM108A1 | 3.64E+00 | 2.76E-04 | 8.45E-04 |
| C20orf24 | 3.64E+00 | 2.76E-04 | 8.46E-04 |
| PFN1 | 3.64E+00 | 2.76E-04 | 8.47E-04 |
| DOK5 | -3.64E+00 | 2.77E-04 | 8.50E-04 |
| NR2C1 | 3.64E+00 | 2.77E-04 | 8.50E-04 |
| TSPAN6 | -3.64E+00 | 2.78E-04 | 8.53E-04 |
| ITPR2 | -3.63E+00 | 2.79E-04 | 8.54E-04 |
| KLRC3 | 3.63E+00 | 2.79E-04 | 8.54E-04 |
| SRMS | 3.63E+00 | 2.79E-04 | 8.55E-04 |
| TACC1 | -3.63E+00 | 2.79E-04 | 8.55E-04 |
| UGT3A2 | 3.63E+00 | 2.79E-04 | 8.55E-04 |
| ABCA2 | 3.63E+00 | 2.79E-04 | 8.55E-04 |
| AKNA | 3.63E+00 | 2.79E-04 | 8.55E-04 |
| OR13A1 | 3.63E+00 | 2.79E-04 | 8.55E-04 |
| DARC | -3.63E+00 | 2.80E-04 | 8.57E-04 |
| C12orf53 | 3.63E+00 | 2.80E-04 | 8.57E-04 |
| LEUTX | 3.63E+00 | 2.80E-04 | 8.58E-04 |
| IGFALS | -3.63E+00 | 2.81E-04 | 8.60E-04 |
| LOC100286844 | 3.63E+00 | 2.81E-04 | 8.60E-04 |
| OSBPL3 | 3.63E+00 | 2.82E-04 | 8.61E-04 |
| BNIP2 | -3.63E+00 | 2.83E-04 | 8.66E-04 |
| ESRRA | 3.63E+00 | 2.83E-04 | 8.66E-04 |

| C6orf201 | -3.63E+00 | 2.84E-04 | 8.69E-04 |
| --- | --- | --- | --- |
| ZBTB1 | -3.63E+00 | 2.85E-04 | 8.70E-04 |
| XRN2 | 3.63E+00 | 2.85E-04 | 8.71E-04 |
| ADAM8 | 3.63E+00 | 2.85E-04 | 8.72E-04 |
| AP3M2 | 3.63E+00 | 2.85E-04 | 8.72E-04 |
| USP16 | -3.63E+00 | 2.85E-04 | 8.72E-04 |
| ARCN1 | 3.63E+00 | 2.86E-04 | 8.73E-04 |
| PTPRF | 3.63E+00 | 2.86E-04 | 8.74E-04 |
| C22orf41 | -3.63E+00 | 2.87E-04 | 8.75E-04 |
| MEG3 | 3.63E+00 | 2.87E-04 | 8.77E-04 |
| AMN1 | -3.63E+00 | 2.88E-04 | 8.78E-04 |
| ECE1 | 3.63E+00 | 2.88E-04 | 8.78E-04 |
| SPANXN3 | 3.63E+00 | 2.88E-04 | 8.80E-04 |
| ZIM2 | 3.63E+00 | 2.89E-04 | 8.81E-04 |
| EMILIN1 | 3.63E+00 | 2.89E-04 | 8.81E-04 |
| ARSF | -3.63E+00 | 2.89E-04 | 8.83E-04 |
| C14orf147 | -3.62E+00 | 2.91E-04 | 8.88E-04 |
| PTPN12 | 3.62E+00 | 2.91E-04 | 8.88E-04 |
| NRARP | 3.62E+00 | 2.92E-04 | 8.89E-04 |
| HSP90AB2P | 3.62E+00 | 2.92E-04 | 8.91E-04 |
| MBLAC1 | 3.62E+00 | 2.92E-04 | 8.91E-04 |
| UBE2D4 | -3.62E+00 | 2.92E-04 | 8.91E-04 |
| TRDN | 3.62E+00 | 2.93E-04 | 8.93E-04 |
| MAP1LC3A | -3.62E+00 | 2.94E-04 | 8.95E-04 |
| PLAU | 3.62E+00 | 2.94E-04 | 8.95E-04 |
| C1QTNF7 | -3.62E+00 | 2.97E-04 | 9.04E-04 |
| SNX13 | -3.62E+00 | 2.97E-04 | 9.04E-04 |
| ZSWIM1 | 3.62E+00 | 2.97E-04 | 9.04E-04 |
| C1orf103 | 3.62E+00 | 2.98E-04 | 9.07E-04 |
| NCRNA00087 | -3.62E+00 | 2.98E-04 | 9.07E-04 |
| FAM98C | -3.62E+00 | 2.99E-04 | 9.10E-04 |
| GPAM | -3.62E+00 | 2.99E-04 | 9.11E-04 |
| AASDH | -3.62E+00 | 3.00E-04 | 9.13E-04 |
| ZNF714 | 3.62E+00 | 3.01E-04 | 9.15E-04 |
| PAPOLA | 3.62E+00 | 3.01E-04 | 9.15E-04 |
| ST6GALNAC5 | 3.61E+00 | 3.01E-04 | 9.16E-04 |
| FLJ46111 | 3.61E+00 | 3.01E-04 | 9.16E-04 |
| ZBTB8B | 3.61E+00 | 3.02E-04 | 9.17E-04 |
| PPP1R14D | 3.61E+00 | 3.03E-04 | 9.21E-04 |
| LOC646627 | 3.61E+00 | 3.03E-04 | 9.21E-04 |
| GOLGA8DP | 3.61E+00 | 3.04E-04 | 9.22E-04 |
| AIRE | 3.61E+00 | 3.04E-04 | 9.23E-04 |
| ASPH | 3.61E+00 | 3.04E-04 | 9.24E-04 |
| ZNF785 | 3.61E+00 | 3.05E-04 | 9.26E-04 |
| DENND5A | 3.61E+00 | 3.05E-04 | 9.27E-04 |
| AP3M1 | 3.61E+00 | 3.06E-04 | 9.30E-04 |
| TMEM53 | -3.61E+00 | 3.07E-04 | 9.33E-04 |
| FAM100B | 3.61E+00 | 3.08E-04 | 9.35E-04 |
| PTRH2 | 3.61E+00 | 3.08E-04 | 9.35E-04 |
| CNPY4 | 3.61E+00 | 3.09E-04 | 9.36E-04 |
| POU2F1 | 3.61E+00 | 3.09E-04 | 9.36E-04 |
| ATP5I | -3.61E+00 | 3.10E-04 | 9.39E-04 |
| SNX27 | 3.61E+00 | 3.11E-04 | 9.43E-04 |
| ZNF800 | -3.61E+00 | 3.11E-04 | 9.43E-04 |
| RENBP | 3.61E+00 | 3.11E-04 | 9.44E-04 |
| RYBP | -3.61E+00 | 3.11E-04 | 9.44E-04 |
| SMAD1 | -3.61E+00 | 3.11E-04 | 9.44E-04 |

| FAM71F1 | 3.61E+00 | 3.12E-04 | 9.45E-04 |
| --- | --- | --- | --- |
| APLP2 | -3.60E+00 | 3.13E-04 | 9.49E-04 |
| TBC1D10A | -3.60E+00 | 3.14E-04 | 9.50E-04 |
| WNT9B | -3.60E+00 | 3.15E-04 | 9.53E-04 |
| COL14A1 | -3.60E+00 | 3.15E-04 | 9.53E-04 |
| DECR1 | -3.60E+00 | 3.15E-04 | 9.53E-04 |
| C5orf46 | 3.60E+00 | 3.16E-04 | 9.55E-04 |
| GXYLT1 | 3.60E+00 | 3.16E-04 | 9.56E-04 |
| C4orf26 | 3.60E+00 | 3.16E-04 | 9.57E-04 |
| DMRTA1 | -3.60E+00 | 3.18E-04 | 9.61E-04 |
| ZNF527 | -3.60E+00 | 3.18E-04 | 9.63E-04 |
| S100A9 | 3.60E+00 | 3.19E-04 | 9.66E-04 |
| HNRNPH2 | -3.60E+00 | 3.21E-04 | 9.70E-04 |
| SPOP | -3.60E+00 | 3.21E-04 | 9.70E-04 |
| AGAP1 | 3.60E+00 | 3.22E-04 | 9.73E-04 |
| MGC16384 | 3.60E+00 | 3.22E-04 | 9.73E-04 |
| BCHE | -3.60E+00 | 3.22E-04 | 9.74E-04 |
| C2orf42 | -3.60E+00 | 3.23E-04 | 9.75E-04 |
| MYLPF | -3.60E+00 | 3.23E-04 | 9.75E-04 |
| TMCO1 | -3.60E+00 | 3.23E-04 | 9.76E-04 |
| SERPINA7 | -3.60E+00 | 3.24E-04 | 9.79E-04 |
| CILP2 | 3.60E+00 | 3.25E-04 | 9.80E-04 |
| C5orf49 | -3.59E+00 | 3.25E-04 | 9.82E-04 |
| ANKRD37 | -3.59E+00 | 3.26E-04 | 9.83E-04 |
| COX6A1 | 3.59E+00 | 3.26E-04 | 9.83E-04 |
| JMJD1C | -3.59E+00 | 3.27E-04 | 9.86E-04 |
| CIB1 | -3.59E+00 | 3.27E-04 | 9.86E-04 |
| ALS2CR4 | 3.59E+00 | 3.28E-04 | 9.88E-04 |
| RPUSD3 | 3.59E+00 | 3.28E-04 | 9.89E-04 |
| ABCC3 | -3.59E+00 | 3.29E-04 | 9.91E-04 |
| BRAF | 3.59E+00 | 3.28E-04 | 9.91E-04 |
| REEP6 | -3.59E+00 | 3.29E-04 | 9.93E-04 |
| CRYGC | 3.59E+00 | 3.30E-04 | 9.93E-04 |
| PROCA1 | 3.59E+00 | 3.30E-04 | 9.96E-04 |
| CAPN13 | -3.59E+00 | 3.31E-04 | 9.97E-04 |
| CLRN3 | -3.59E+00 | 3.31E-04 | 9.97E-04 |
| MMP20 | 3.59E+00 | 3.31E-04 | 9.97E-04 |
| SYCP1 | 3.59E+00 | 3.32E-04 | 9.99E-04 |
| PRSS38 | 3.59E+00 | 3.32E-04 | 1.00E-03 |
| C6orf127 | 3.59E+00 | 3.32E-04 | 1.00E-03 |
| RNF146 | -3.59E+00 | 3.32E-04 | 1.00E-03 |
| NOS3 | 3.59E+00 | 3.33E-04 | 1.00E-03 |
| NMNAT3 | -3.59E+00 | 3.33E-04 | 1.00E-03 |
| LITAF | -3.59E+00 | 3.34E-04 | 1.01E-03 |
| ANTXR2 | -3.59E+00 | 3.34E-04 | 1.01E-03 |
| OXSM | -3.59E+00 | 3.35E-04 | 1.01E-03 |
| PHOX2A | 3.59E+00 | 3.35E-04 | 1.01E-03 |
| C20orf11 | 3.59E+00 | 3.36E-04 | 1.01E-03 |
| C1orf216 | 3.59E+00 | 3.36E-04 | 1.01E-03 |
| FPGT | -3.59E+00 | 3.37E-04 | 1.01E-03 |
| L3MBTL3 | 3.59E+00 | 3.37E-04 | 1.01E-03 |
| PRG2 | -3.59E+00 | 3.37E-04 | 1.01E-03 |
| HLA-E | -3.58E+00 | 3.38E-04 | 1.02E-03 |
| GGT5 | -3.58E+00 | 3.38E-04 | 1.02E-03 |
| CCDC90A | -3.58E+00 | 3.39E-04 | 1.02E-03 |
| C3orf37 | 3.58E+00 | 3.39E-04 | 1.02E-03 |
| EVI5L | 3.58E+00 | 3.39E-04 | 1.02E-03 |

| NCK1 | -3.58E+00 | 3.41E-04 | 1.02E-03 |
| --- | --- | --- | --- |
| TRIL | -3.58E+00 | 3.41E-04 | 1.02E-03 |
| SEC61G | 3.58E+00 | 3.42E-04 | 1.03E-03 |
| STRA8 | 3.58E+00 | 3.43E-04 | 1.03E-03 |
| OPCML | -3.58E+00 | 3.43E-04 | 1.03E-03 |
| APOB48R | 3.58E+00 | 3.44E-04 | 1.03E-03 |
| OTUD3 | 3.58E+00 | 3.44E-04 | 1.03E-03 |
| PSD3 | -3.58E+00 | 3.45E-04 | 1.04E-03 |
| MT1E | -3.58E+00 | 3.46E-04 | 1.04E-03 |
| C12orf23 | 3.58E+00 | 3.46E-04 | 1.04E-03 |
| C6orf223 | 3.58E+00 | 3.46E-04 | 1.04E-03 |
| ST6GAL2 | -3.58E+00 | 3.46E-04 | 1.04E-03 |
| DDT | -3.58E+00 | 3.46E-04 | 1.04E-03 |
| NENF | -3.58E+00 | 3.49E-04 | 1.05E-03 |
| ANAPC11 | 3.58E+00 | 3.49E-04 | 1.05E-03 |
| GP2 | 3.58E+00 | 3.49E-04 | 1.05E-03 |
| REV1 | -3.58E+00 | 3.49E-04 | 1.05E-03 |
| THSD4 | -3.58E+00 | 3.49E-04 | 1.05E-03 |
| GLOD5 | -3.58E+00 | 3.50E-04 | 1.05E-03 |
| DAAM1 | -3.58E+00 | 3.50E-04 | 1.05E-03 |
| CNTNAP3 | -3.58E+00 | 3.50E-04 | 1.05E-03 |
| C3orf18 | -3.58E+00 | 3.50E-04 | 1.05E-03 |
| RASSF3 | 3.58E+00 | 3.51E-04 | 1.05E-03 |
| GAGE10 | 3.57E+00 | 3.51E-04 | 1.05E-03 |
| C9orf82 | -3.57E+00 | 3.51E-04 | 1.05E-03 |
| DNAJB14 | -3.57E+00 | 3.51E-04 | 1.05E-03 |
| OSCP1 | -3.57E+00 | 3.52E-04 | 1.05E-03 |
| BRDT | 3.57E+00 | 3.52E-04 | 1.05E-03 |
| TNS1 | -3.57E+00 | 3.53E-04 | 1.06E-03 |
| NDUFS3 | -3.57E+00 | 3.53E-04 | 1.06E-03 |
| SLCO1C1 | -3.57E+00 | 3.53E-04 | 1.06E-03 |
| XPO4 | 3.57E+00 | 3.53E-04 | 1.06E-03 |
| FCRL6 | -3.57E+00 | 3.54E-04 | 1.06E-03 |
| MKNK1 | 3.57E+00 | 3.55E-04 | 1.06E-03 |
| IFI30 | 3.57E+00 | 3.55E-04 | 1.06E-03 |
| SHISA7 | 3.57E+00 | 3.55E-04 | 1.06E-03 |
| CCL20 | 3.57E+00 | 3.55E-04 | 1.06E-03 |
| C17orf28 | 3.57E+00 | 3.57E-04 | 1.07E-03 |
| ST6GALNAC4 | 3.57E+00 | 3.58E-04 | 1.07E-03 |
| ADAMTS9 | 3.57E+00 | 3.59E-04 | 1.07E-03 |
| IMP3 | -3.57E+00 | 3.59E-04 | 1.07E-03 |
| INF2 | 3.57E+00 | 3.59E-04 | 1.07E-03 |
| C10orf10 | -3.57E+00 | 3.59E-04 | 1.07E-03 |
| FCAMR | -3.57E+00 | 3.59E-04 | 1.07E-03 |
| GTF2F2 | 3.57E+00 | 3.59E-04 | 1.07E-03 |
| TCEA3 | -3.57E+00 | 3.59E-04 | 1.07E-03 |
| C6orf89 | -3.57E+00 | 3.59E-04 | 1.07E-03 |
| PIGA | -3.57E+00 | 3.60E-04 | 1.07E-03 |
| ELANE | -3.57E+00 | 3.60E-04 | 1.07E-03 |
| ALKBH6 | 3.57E+00 | 3.60E-04 | 1.07E-03 |
| C11orf52 | -3.57E+00 | 3.60E-04 | 1.07E-03 |
| JSRP1 | 3.57E+00 | 3.61E-04 | 1.07E-03 |
| KDM5B | 3.57E+00 | 3.61E-04 | 1.08E-03 |
| CALCR | 3.57E+00 | 3.61E-04 | 1.08E-03 |
| CDKN2AIPNL | 3.57E+00 | 3.61E-04 | 1.08E-03 |
| FNDC3A | -3.57E+00 | 3.62E-04 | 1.08E-03 |
| LOC388692 | 3.57E+00 | 3.62E-04 | 1.08E-03 |

| XYLB | 3.57E+00 | 3.62E-04 | 1.08E-03 |
| --- | --- | --- | --- |
| TSPAN10 | 3.57E+00 | 3.63E-04 | 1.08E-03 |
| SPR | -3.57E+00 | 3.63E-04 | 1.08E-03 |
| GPR143 | -3.57E+00 | 3.63E-04 | 1.08E-03 |
| SLCO4A1 | 3.57E+00 | 3.63E-04 | 1.08E-03 |
| TXK | -3.57E+00 | 3.64E-04 | 1.08E-03 |
| C11orf41 | 3.57E+00 | 3.64E-04 | 1.08E-03 |
| TTC30A | -3.57E+00 | 3.65E-04 | 1.08E-03 |
| THUMPD1 | -3.56E+00 | 3.65E-04 | 1.09E-03 |
| MRPS6 | -3.56E+00 | 3.66E-04 | 1.09E-03 |
| FAM47C | -3.56E+00 | 3.67E-04 | 1.09E-03 |
| C11orf94 | 3.56E+00 | 3.67E-04 | 1.09E-03 |
| DTWD1 | -3.56E+00 | 3.67E-04 | 1.09E-03 |
| ZSCAN10 | 3.56E+00 | 3.68E-04 | 1.09E-03 |
| NUBP1 | -3.56E+00 | 3.68E-04 | 1.09E-03 |
| OR8S1 | 3.56E+00 | 3.69E-04 | 1.10E-03 |
| ZNF580 | 3.56E+00 | 3.69E-04 | 1.10E-03 |
| LRRK2 | -3.56E+00 | 3.69E-04 | 1.10E-03 |
| ANKRD35 | -3.56E+00 | 3.70E-04 | 1.10E-03 |
| DNAJC17 | -3.56E+00 | 3.71E-04 | 1.10E-03 |
| PVRL3 | -3.56E+00 | 3.71E-04 | 1.10E-03 |
| ABI2 | 3.56E+00 | 3.73E-04 | 1.11E-03 |
| MAP3K13 | -3.56E+00 | 3.73E-04 | 1.11E-03 |
| GALNT1 | -3.56E+00 | 3.74E-04 | 1.11E-03 |
| GATSL3 | -3.56E+00 | 3.75E-04 | 1.11E-03 |
| SLC1A4 | 3.56E+00 | 3.76E-04 | 1.11E-03 |
| CLEC9A | -3.56E+00 | 3.76E-04 | 1.11E-03 |
| GAA | 3.56E+00 | 3.76E-04 | 1.11E-03 |
| TUT1 | 3.56E+00 | 3.76E-04 | 1.12E-03 |
| SOX7 | -3.56E+00 | 3.76E-04 | 1.12E-03 |
| SOX2 | 3.56E+00 | 3.78E-04 | 1.12E-03 |
| FERMT3 | 3.55E+00 | 3.79E-04 | 1.12E-03 |
| MPO | 3.55E+00 | 3.79E-04 | 1.12E-03 |
| RGS7BP | -3.55E+00 | 3.80E-04 | 1.12E-03 |
| HSPE1 | 3.55E+00 | 3.81E-04 | 1.13E-03 |
| SERPINE2 | 3.55E+00 | 3.81E-04 | 1.13E-03 |
| SEMA4B | 3.55E+00 | 3.81E-04 | 1.13E-03 |
| ZNF587 | 3.55E+00 | 3.81E-04 | 1.13E-03 |
| SPACA5 | -3.55E+00 | 3.81E-04 | 1.13E-03 |
| LOC100133612 | 3.55E+00 | 3.83E-04 | 1.13E-03 |
| PCGF2 | 3.55E+00 | 3.84E-04 | 1.14E-03 |
| SMEK2 | -3.55E+00 | 3.85E-04 | 1.14E-03 |
| C18orf45 | 3.55E+00 | 3.85E-04 | 1.14E-03 |
| EFHD1 | -3.55E+00 | 3.88E-04 | 1.15E-03 |
| CHRNA7 | 3.55E+00 | 3.88E-04 | 1.15E-03 |
| TPRG1L | -3.55E+00 | 3.88E-04 | 1.15E-03 |
| PPTC7 | 3.55E+00 | 3.88E-04 | 1.15E-03 |
| MFAP2 | 3.55E+00 | 3.89E-04 | 1.15E-03 |
| NBR1 | -3.55E+00 | 3.90E-04 | 1.15E-03 |
| CD274 | 3.55E+00 | 3.90E-04 | 1.15E-03 |
| UBXN1 | 3.55E+00 | 3.90E-04 | 1.15E-03 |
| TFPI | -3.55E+00 | 3.91E-04 | 1.16E-03 |
| MEF2A | -3.55E+00 | 3.92E-04 | 1.16E-03 |
| SEMA6D | -3.55E+00 | 3.92E-04 | 1.16E-03 |
| OSBPL10 | 3.55E+00 | 3.92E-04 | 1.16E-03 |
| SSNA1 | 3.55E+00 | 3.93E-04 | 1.16E-03 |
| BBX | 3.55E+00 | 3.93E-04 | 1.16E-03 |

| ZEB1 | -3.54E+00 | 3.95E-04 | 1.17E-03 |
| --- | --- | --- | --- |
| RASL10B | 3.54E+00 | 3.97E-04 | 1.17E-03 |
| KIN | 3.54E+00 | 3.98E-04 | 1.17E-03 |
| MIER1 | -3.54E+00 | 3.98E-04 | 1.17E-03 |
| TLX1NB | 3.54E+00 | 3.99E-04 | 1.18E-03 |
| ADAMTSL2 | -3.54E+00 | 3.99E-04 | 1.18E-03 |
| SLC43A1 | -3.54E+00 | 4.00E-04 | 1.18E-03 |
| RSPO2 | -3.54E+00 | 4.00E-04 | 1.18E-03 |
| MID1IP1 | 3.54E+00 | 4.01E-04 | 1.18E-03 |
| LOC344967 | 3.54E+00 | 4.01E-04 | 1.18E-03 |
| SIX4 | 3.54E+00 | 4.01E-04 | 1.18E-03 |
| GALC | -3.54E+00 | 4.02E-04 | 1.18E-03 |
| PLA2G4D | 3.54E+00 | 4.02E-04 | 1.18E-03 |
| TMBIM6 | -3.54E+00 | 4.02E-04 | 1.18E-03 |
| GRINA | 3.54E+00 | 4.02E-04 | 1.19E-03 |
| LOC201651 | -3.54E+00 | 4.03E-04 | 1.19E-03 |
| H2AFY2 | 3.54E+00 | 4.03E-04 | 1.19E-03 |
| TMEM205 | -3.54E+00 | 4.04E-04 | 1.19E-03 |
| ABCG2 | -3.54E+00 | 4.05E-04 | 1.19E-03 |
| ADAM21P1 | 3.54E+00 | 4.05E-04 | 1.19E-03 |
| LRCH4 | 3.54E+00 | 4.06E-04 | 1.19E-03 |
| TOP1 | 3.54E+00 | 4.06E-04 | 1.19E-03 |
| TMEM9B | -3.54E+00 | 4.06E-04 | 1.20E-03 |
| PCGF3 | 3.54E+00 | 4.07E-04 | 1.20E-03 |
| RTP4 | -3.54E+00 | 4.08E-04 | 1.20E-03 |
| DNAJC25 | -3.53E+00 | 4.09E-04 | 1.20E-03 |
| EIF4EBP3 | -3.53E+00 | 4.10E-04 | 1.20E-03 |
| MSH3 | -3.53E+00 | 4.10E-04 | 1.21E-03 |
| DNAH11 | -3.53E+00 | 4.10E-04 | 1.21E-03 |
| ACSS2 | -3.53E+00 | 4.11E-04 | 1.21E-03 |
| NDUFB10 | -3.53E+00 | 4.12E-04 | 1.21E-03 |
| ARSI | 3.53E+00 | 4.14E-04 | 1.22E-03 |
| ANKRD58 | 3.53E+00 | 4.14E-04 | 1.22E-03 |
| PPP4R2 | 3.53E+00 | 4.16E-04 | 1.22E-03 |
| CASP14 | 3.53E+00 | 4.16E-04 | 1.22E-03 |
| WASF3 | -3.53E+00 | 4.16E-04 | 1.22E-03 |
| TERF2 | 3.53E+00 | 4.16E-04 | 1.22E-03 |
| RBP7 | -3.53E+00 | 4.16E-04 | 1.22E-03 |
| RXFP3 | 3.53E+00 | 4.17E-04 | 1.23E-03 |
| MTDH | 3.53E+00 | 4.18E-04 | 1.23E-03 |
| DLEU2 | 3.53E+00 | 4.18E-04 | 1.23E-03 |
| KDM1B | 3.53E+00 | 4.20E-04 | 1.23E-03 |
| SNORA27 | 3.53E+00 | 4.20E-04 | 1.23E-03 |
| TIGD1 | 3.53E+00 | 4.21E-04 | 1.23E-03 |
| ALG10 | 3.53E+00 | 4.22E-04 | 1.24E-03 |
| C14orf43 | 3.53E+00 | 4.23E-04 | 1.24E-03 |
| KIFAP3 | -3.53E+00 | 4.24E-04 | 1.24E-03 |
| GLCE | -3.52E+00 | 4.24E-04 | 1.24E-03 |
| RALGDS | 3.52E+00 | 4.25E-04 | 1.25E-03 |
| ZDHHC24 | 3.52E+00 | 4.25E-04 | 1.25E-03 |
| CELP | 3.52E+00 | 4.25E-04 | 1.25E-03 |
| FAM95B1 | -3.52E+00 | 4.26E-04 | 1.25E-03 |
| PICK1 | 3.52E+00 | 4.26E-04 | 1.25E-03 |
| PTGES | 3.52E+00 | 4.27E-04 | 1.25E-03 |
| AP1M1 | 3.52E+00 | 4.28E-04 | 1.25E-03 |
| ZBTB22 | -3.52E+00 | 4.29E-04 | 1.26E-03 |
| STK36 | 3.52E+00 | 4.30E-04 | 1.26E-03 |

| EPB41L5 | -3.52E+00 | 4.31E-04 | 1.26E-03 |
| --- | --- | --- | --- |
| KLKB1 | -3.52E+00 | 4.31E-04 | 1.26E-03 |
| NYX | 3.52E+00 | 4.31E-04 | 1.26E-03 |
| CCNT1 | 3.52E+00 | 4.32E-04 | 1.26E-03 |
| DOCK3 | 3.52E+00 | 4.33E-04 | 1.27E-03 |
| ILKAP | 3.52E+00 | 4.34E-04 | 1.27E-03 |
| SEC22B | -3.52E+00 | 4.34E-04 | 1.27E-03 |
| WDR61 | -3.52E+00 | 4.35E-04 | 1.27E-03 |
| PTPRN2 | -3.52E+00 | 4.36E-04 | 1.27E-03 |
| GDF5 | -3.52E+00 | 4.36E-04 | 1.27E-03 |
| CH25H | -3.52E+00 | 4.36E-04 | 1.28E-03 |
| SLC38A5 | 3.52E+00 | 4.36E-04 | 1.28E-03 |
| ZNF330 | -3.52E+00 | 4.36E-04 | 1.28E-03 |
| HBM | -3.52E+00 | 4.39E-04 | 1.28E-03 |
| VAX2 | 3.52E+00 | 4.39E-04 | 1.28E-03 |
| ST6GALNAC6 | -3.52E+00 | 4.40E-04 | 1.28E-03 |
| RALGPS1 | -3.52E+00 | 4.41E-04 | 1.29E-03 |
| STARD5 | -3.51E+00 | 4.41E-04 | 1.29E-03 |
| CYBA | 3.51E+00 | 4.41E-04 | 1.29E-03 |
| ARPM1 | -3.51E+00 | 4.42E-04 | 1.29E-03 |
| GCC1 | 3.51E+00 | 4.43E-04 | 1.29E-03 |
| MAPKBP1 | 3.51E+00 | 4.43E-04 | 1.29E-03 |
| BIRC7 | 3.51E+00 | 4.44E-04 | 1.30E-03 |
| IL28RA | -3.51E+00 | 4.45E-04 | 1.30E-03 |
| ERI1 | 3.51E+00 | 4.46E-04 | 1.30E-03 |
| CES7 | -3.51E+00 | 4.46E-04 | 1.30E-03 |
| EPB41L2 | 3.51E+00 | 4.46E-04 | 1.30E-03 |
| MEP1A | 3.51E+00 | 4.47E-04 | 1.30E-03 |
| CA3 | -3.51E+00 | 4.48E-04 | 1.30E-03 |
| PAPL | 3.51E+00 | 4.48E-04 | 1.30E-03 |
| TSPAN31 | -3.51E+00 | 4.49E-04 | 1.31E-03 |
| CLDND1 | -3.51E+00 | 4.50E-04 | 1.31E-03 |
| C6orf26 | 3.51E+00 | 4.50E-04 | 1.31E-03 |
| PELI2 | -3.51E+00 | 4.52E-04 | 1.32E-03 |
| SLAIN2 | -3.51E+00 | 4.52E-04 | 1.32E-03 |
| KCNAB1 | -3.51E+00 | 4.54E-04 | 1.32E-03 |
| PCDHGC3 | -3.51E+00 | 4.55E-04 | 1.32E-03 |
| SIX6 | 3.51E+00 | 4.56E-04 | 1.33E-03 |
| CA13 | -3.50E+00 | 4.58E-04 | 1.33E-03 |
| POU3F3 | 3.50E+00 | 4.58E-04 | 1.33E-03 |
| CISD2 | -3.50E+00 | 4.59E-04 | 1.34E-03 |
| CYP21A2 | -3.50E+00 | 4.59E-04 | 1.34E-03 |
| TCTE3 | 3.50E+00 | 4.59E-04 | 1.34E-03 |
| BRPF3 | 3.50E+00 | 4.60E-04 | 1.34E-03 |
| HLA-F | -3.50E+00 | 4.61E-04 | 1.34E-03 |
| SDHB | -3.50E+00 | 4.62E-04 | 1.34E-03 |
| CA14 | -3.50E+00 | 4.62E-04 | 1.34E-03 |
| GSTCD | 3.50E+00 | 4.63E-04 | 1.34E-03 |
| RAB2B | -3.50E+00 | 4.63E-04 | 1.34E-03 |
| SLC8A2 | 3.50E+00 | 4.63E-04 | 1.35E-03 |
| RAB37 | -3.50E+00 | 4.64E-04 | 1.35E-03 |
| SELT | -3.50E+00 | 4.65E-04 | 1.35E-03 |
| CSNK2A2 | 3.50E+00 | 4.66E-04 | 1.35E-03 |
| KRTAP3-1 | 3.50E+00 | 4.67E-04 | 1.36E-03 |
| LOC100009676 | 3.50E+00 | 4.67E-04 | 1.36E-03 |
| GPN3 | 3.50E+00 | 4.68E-04 | 1.36E-03 |
| PPL | -3.50E+00 | 4.69E-04 | 1.36E-03 |

| C6orf136 | 3.50E+00 | 4.71E-04 | 1.37E-03 |
| --- | --- | --- | --- |
| ARL6IP6 | 3.50E+00 | 4.71E-04 | 1.37E-03 |
| EIF5B | 3.50E+00 | 4.71E-04 | 1.37E-03 |
| ALDH3B2 | 3.50E+00 | 4.72E-04 | 1.37E-03 |
| CSTL1 | 3.50E+00 | 4.72E-04 | 1.37E-03 |
| COX6B2 | 3.50E+00 | 4.73E-04 | 1.37E-03 |
| SPRY2 | -3.50E+00 | 4.73E-04 | 1.37E-03 |
| RP1 | -3.49E+00 | 4.77E-04 | 1.38E-03 |
| CCDC154 | 3.49E+00 | 4.77E-04 | 1.38E-03 |
| SIAH1 | -3.49E+00 | 4.77E-04 | 1.38E-03 |
| C3orf20 | 3.49E+00 | 4.78E-04 | 1.38E-03 |
| UNC5A | 3.49E+00 | 4.78E-04 | 1.39E-03 |
| NIPSNAP3B | -3.49E+00 | 4.78E-04 | 1.39E-03 |
| SCNM1 | 3.49E+00 | 4.81E-04 | 1.39E-03 |
| ZNF577 | -3.49E+00 | 4.82E-04 | 1.40E-03 |
| HSPA8 | 3.49E+00 | 4.83E-04 | 1.40E-03 |
| VDAC2 | 3.49E+00 | 4.83E-04 | 1.40E-03 |
| ZNF575 | -3.49E+00 | 4.86E-04 | 1.41E-03 |
| TATDN1 | 3.49E+00 | 4.87E-04 | 1.41E-03 |
| NCCRP1 | 3.49E+00 | 4.88E-04 | 1.41E-03 |
| HRH2 | 3.49E+00 | 4.88E-04 | 1.41E-03 |
| LDHA | 3.49E+00 | 4.88E-04 | 1.41E-03 |
| MGP | -3.49E+00 | 4.89E-04 | 1.41E-03 |
| ITGA9 | -3.49E+00 | 4.89E-04 | 1.41E-03 |
| SCAND1 | 3.49E+00 | 4.89E-04 | 1.42E-03 |
| CCNT2 | -3.49E+00 | 4.90E-04 | 1.42E-03 |
| ERBB2IP | -3.49E+00 | 4.91E-04 | 1.42E-03 |
| SPINK7 | -3.49E+00 | 4.91E-04 | 1.42E-03 |
| UNC5B | 3.49E+00 | 4.91E-04 | 1.42E-03 |
| IFI27L1 | -3.49E+00 | 4.92E-04 | 1.42E-03 |
| MYO1G | 3.49E+00 | 4.92E-04 | 1.42E-03 |
| PSMA7 | 3.49E+00 | 4.93E-04 | 1.42E-03 |
| RTKN2 | 3.49E+00 | 4.93E-04 | 1.42E-03 |
| SMAP1 | 3.49E+00 | 4.93E-04 | 1.42E-03 |
| ZNF750 | -3.48E+00 | 4.94E-04 | 1.43E-03 |
| DDX25 | 3.48E+00 | 4.95E-04 | 1.43E-03 |
| SLC4A11 | 3.48E+00 | 4.95E-04 | 1.43E-03 |
| ZBTB7B | 3.48E+00 | 4.95E-04 | 1.43E-03 |
| OR56A1 | 3.48E+00 | 4.96E-04 | 1.43E-03 |
| HCRT | 3.48E+00 | 4.97E-04 | 1.43E-03 |
| LY6G5C | -3.48E+00 | 4.99E-04 | 1.44E-03 |
| KIAA1683 | -3.48E+00 | 4.99E-04 | 1.44E-03 |
| RPP30 | 3.48E+00 | 5.01E-04 | 1.44E-03 |
| WASL | -3.48E+00 | 5.01E-04 | 1.44E-03 |
| APOM | -3.48E+00 | 5.02E-04 | 1.45E-03 |
| GLRX | -3.48E+00 | 5.03E-04 | 1.45E-03 |
| CMYA5 | -3.48E+00 | 5.03E-04 | 1.45E-03 |
| C13orf37 | 3.48E+00 | 5.03E-04 | 1.45E-03 |
| SOD1 | -3.48E+00 | 5.03E-04 | 1.45E-03 |
| WHAMML1 | -3.48E+00 | 5.04E-04 | 1.45E-03 |
| BEND5 | -3.48E+00 | 5.04E-04 | 1.45E-03 |
| LMNA | 3.48E+00 | 5.04E-04 | 1.45E-03 |
| C11orf36 | 3.48E+00 | 5.04E-04 | 1.45E-03 |
| BCO2 | -3.48E+00 | 5.05E-04 | 1.45E-03 |
| ABTB1 | -3.48E+00 | 5.06E-04 | 1.46E-03 |
| TBCEL | -3.48E+00 | 5.06E-04 | 1.46E-03 |
| PREB | 3.48E+00 | 5.08E-04 | 1.46E-03 |

| SLC24A2 | 3.48E+00 | 5.11E-04 | 1.47E-03 |
| --- | --- | --- | --- |
| MIER3 | -3.47E+00 | 5.13E-04 | 1.48E-03 |
| KRTAP3-2 | 3.47E+00 | 5.14E-04 | 1.48E-03 |
| SNAI1 | 3.47E+00 | 5.14E-04 | 1.48E-03 |
| DNAJC24 | -3.47E+00 | 5.14E-04 | 1.48E-03 |
| RECK | -3.47E+00 | 5.14E-04 | 1.48E-03 |
| ATF6B | 3.47E+00 | 5.15E-04 | 1.48E-03 |
| LSM11 | 3.47E+00 | 5.17E-04 | 1.49E-03 |
| SCARF1 | -3.47E+00 | 5.18E-04 | 1.49E-03 |
| ELAC1 | -3.47E+00 | 5.19E-04 | 1.49E-03 |
| MON1A | 3.47E+00 | 5.19E-04 | 1.49E-03 |
| TPBG | 3.47E+00 | 5.19E-04 | 1.49E-03 |
| FAM174B | -3.47E+00 | 5.20E-04 | 1.49E-03 |
| PKD1L2 | 3.47E+00 | 5.21E-04 | 1.50E-03 |
| GOLGA7 | -3.47E+00 | 5.21E-04 | 1.50E-03 |
| TSIX | -3.47E+00 | 5.23E-04 | 1.50E-03 |
| PRICKLE1 | -3.47E+00 | 5.24E-04 | 1.50E-03 |
| HPS5 | -3.47E+00 | 5.24E-04 | 1.50E-03 |
| TWIST1 | 3.47E+00 | 5.25E-04 | 1.51E-03 |
| C9orf68 | -3.47E+00 | 5.26E-04 | 1.51E-03 |
| ZNF207 | 3.47E+00 | 5.26E-04 | 1.51E-03 |
| LUZP1 | 3.47E+00 | 5.27E-04 | 1.51E-03 |
| CALHM3 | 3.47E+00 | 5.28E-04 | 1.51E-03 |
| RPL7 | 3.47E+00 | 5.29E-04 | 1.52E-03 |
| EP300 | 3.47E+00 | 5.30E-04 | 1.52E-03 |
| MYL3 | -3.47E+00 | 5.30E-04 | 1.52E-03 |
| SLC9A11 | -3.47E+00 | 5.30E-04 | 1.52E-03 |
| GLCCI1 | -3.47E+00 | 5.31E-04 | 1.52E-03 |
| TIAL1 | 3.46E+00 | 5.31E-04 | 1.52E-03 |
| IFT27 | -3.46E+00 | 5.34E-04 | 1.53E-03 |
| ZNF214 | -3.46E+00 | 5.35E-04 | 1.53E-03 |
| TDRD6 | -3.46E+00 | 5.35E-04 | 1.53E-03 |
| EPHA3 | -3.46E+00 | 5.36E-04 | 1.54E-03 |
| AOX1 | -3.46E+00 | 5.36E-04 | 1.54E-03 |
| PDE4DIP | -3.46E+00 | 5.37E-04 | 1.54E-03 |
| PVRL1 | 3.46E+00 | 5.37E-04 | 1.54E-03 |
| MICALL2 | 3.46E+00 | 5.37E-04 | 1.54E-03 |
| C3orf34 | 3.46E+00 | 5.39E-04 | 1.54E-03 |
| ATG10 | -3.46E+00 | 5.39E-04 | 1.54E-03 |
| C21orf71 | -3.46E+00 | 5.41E-04 | 1.55E-03 |
| AAK1 | 3.46E+00 | 5.41E-04 | 1.55E-03 |
| C9orf123 | -3.46E+00 | 5.42E-04 | 1.55E-03 |
| BEST2 | 3.46E+00 | 5.43E-04 | 1.55E-03 |
| WDR27 | 3.46E+00 | 5.45E-04 | 1.56E-03 |
| PAMR1 | -3.46E+00 | 5.45E-04 | 1.56E-03 |
| ANGEL1 | 3.46E+00 | 5.46E-04 | 1.56E-03 |
| ALG1L2 | 3.46E+00 | 5.46E-04 | 1.56E-03 |
| NTRK3 | -3.46E+00 | 5.46E-04 | 1.56E-03 |
| TMEM132E | -3.46E+00 | 5.47E-04 | 1.56E-03 |
| NCF1 | 3.46E+00 | 5.47E-04 | 1.56E-03 |
| SHC4 | -3.46E+00 | 5.48E-04 | 1.57E-03 |
| PCGF6 | 3.46E+00 | 5.50E-04 | 1.57E-03 |
| CSH1 | 3.46E+00 | 5.50E-04 | 1.57E-03 |
| RNASE13 | -3.46E+00 | 5.50E-04 | 1.57E-03 |
| SETBP1 | -3.46E+00 | 5.50E-04 | 1.57E-03 |
| TTLL7 | 3.46E+00 | 5.51E-04 | 1.57E-03 |
| BCOR | 3.45E+00 | 5.51E-04 | 1.57E-03 |

| MPPED2 | -3.45E+00 | 5.52E-04 | 1.58E-03 |
| --- | --- | --- | --- |
| H2BFWT | 3.45E+00 | 5.54E-04 | 1.58E-03 |
| ITLN2 | -3.45E+00 | 5.55E-04 | 1.58E-03 |
| FIS1 | -3.45E+00 | 5.56E-04 | 1.59E-03 |
| MYBPC3 | 3.45E+00 | 5.57E-04 | 1.59E-03 |
| GAB4 | 3.45E+00 | 5.58E-04 | 1.59E-03 |
| UNC45B | -3.45E+00 | 5.58E-04 | 1.59E-03 |
| EEF2 | 3.45E+00 | 5.59E-04 | 1.59E-03 |
| PATE4 | 3.45E+00 | 5.59E-04 | 1.59E-03 |
| SCG2 | 3.45E+00 | 5.61E-04 | 1.60E-03 |
| SLIT3 | -3.45E+00 | 5.61E-04 | 1.60E-03 |
| MGC2889 | 3.45E+00 | 5.62E-04 | 1.60E-03 |
| PCYT1A | 3.45E+00 | 5.62E-04 | 1.60E-03 |
| SLCO3A1 | -3.45E+00 | 5.62E-04 | 1.60E-03 |
| LRRFIP2 | -3.45E+00 | 5.63E-04 | 1.60E-03 |
| ARHGAP31 | -3.45E+00 | 5.63E-04 | 1.60E-03 |
| LMX1A | 3.45E+00 | 5.64E-04 | 1.61E-03 |
| SNX31 | -3.45E+00 | 5.66E-04 | 1.61E-03 |
| NAPEPLD | -3.45E+00 | 5.67E-04 | 1.62E-03 |
| LARS | 3.45E+00 | 5.68E-04 | 1.62E-03 |
| METTL5 | 3.45E+00 | 5.68E-04 | 1.62E-03 |
| TBC1D15 | -3.45E+00 | 5.68E-04 | 1.62E-03 |
| NUFIP2 | 3.45E+00 | 5.69E-04 | 1.62E-03 |
| PSMA8 | 3.45E+00 | 5.70E-04 | 1.62E-03 |
| GLTP | 3.45E+00 | 5.70E-04 | 1.62E-03 |
| PRSS53 | 3.45E+00 | 5.71E-04 | 1.62E-03 |
| ART5 | 3.45E+00 | 5.71E-04 | 1.62E-03 |
| ASPG | -3.44E+00 | 5.74E-04 | 1.63E-03 |
| HCG4P6 | -3.44E+00 | 5.74E-04 | 1.63E-03 |
| KLHL9 | -3.44E+00 | 5.75E-04 | 1.63E-03 |
| SERPINI1 | 3.44E+00 | 5.75E-04 | 1.63E-03 |
| CPLX3 | 3.44E+00 | 5.77E-04 | 1.64E-03 |
| FBXO33 | -3.44E+00 | 5.77E-04 | 1.64E-03 |
| PLCZ1 | 3.44E+00 | 5.77E-04 | 1.64E-03 |
| CRABP1 | 3.44E+00 | 5.78E-04 | 1.64E-03 |
| RAC3 | 3.44E+00 | 5.78E-04 | 1.64E-03 |
| CYP3A7 | -3.44E+00 | 5.79E-04 | 1.64E-03 |
| TACR1 | -3.44E+00 | 5.79E-04 | 1.64E-03 |
| LOC285780 | -3.44E+00 | 5.80E-04 | 1.65E-03 |
| ALG12 | 3.44E+00 | 5.80E-04 | 1.65E-03 |
| C5orf24 | -3.44E+00 | 5.81E-04 | 1.65E-03 |
| KCNE1 | -3.44E+00 | 5.81E-04 | 1.65E-03 |
| TAS1R2 | 3.44E+00 | 5.82E-04 | 1.65E-03 |
| NCRNA00092 | -3.44E+00 | 5.83E-04 | 1.65E-03 |
| L3MBTL4 | -3.44E+00 | 5.83E-04 | 1.65E-03 |
| MFRP | -3.44E+00 | 5.84E-04 | 1.66E-03 |
| RDH13 | 3.44E+00 | 5.84E-04 | 1.66E-03 |
| CABP4 | 3.44E+00 | 5.85E-04 | 1.66E-03 |
| RARRES3 | -3.44E+00 | 5.85E-04 | 1.66E-03 |
| MIOX | 3.44E+00 | 5.87E-04 | 1.66E-03 |
| STXBP3 | -3.44E+00 | 5.88E-04 | 1.67E-03 |
| SEC24B | -3.44E+00 | 5.88E-04 | 1.67E-03 |
| STAU1 | 3.44E+00 | 5.90E-04 | 1.67E-03 |
| LASS5 | 3.44E+00 | 5.91E-04 | 1.67E-03 |
| LILRB1 | 3.44E+00 | 5.91E-04 | 1.67E-03 |
| C1orf123 | -3.44E+00 | 5.91E-04 | 1.67E-03 |
| ERLIN1 | 3.44E+00 | 5.92E-04 | 1.68E-03 |

| TYMP | 3.43E+00 | 5.94E-04 | 1.68E-03 |
| --- | --- | --- | --- |
| NTRK2 | -3.43E+00 | 5.94E-04 | 1.68E-03 |
| TRHR | 3.43E+00 | 5.95E-04 | 1.68E-03 |
| TRIM63 | -3.43E+00 | 5.95E-04 | 1.68E-03 |
| EIF6 | 3.43E+00 | 5.96E-04 | 1.69E-03 |
| ALG5 | -3.43E+00 | 5.96E-04 | 1.69E-03 |
| POTEF | 3.43E+00 | 5.97E-04 | 1.69E-03 |
| STAB1 | 3.43E+00 | 5.98E-04 | 1.69E-03 |
| RHOB | -3.43E+00 | 6.00E-04 | 1.70E-03 |
| MFAP4 | -3.43E+00 | 6.01E-04 | 1.70E-03 |
| PCDHB6 | 3.43E+00 | 6.02E-04 | 1.70E-03 |
| CCDC28B | 3.43E+00 | 6.02E-04 | 1.70E-03 |
| LYPLAL1 | -3.43E+00 | 6.03E-04 | 1.70E-03 |
| IPMK | 3.43E+00 | 6.04E-04 | 1.71E-03 |
| ZNF541 | -3.43E+00 | 6.07E-04 | 1.71E-03 |
| OR1F2P | 3.43E+00 | 6.07E-04 | 1.72E-03 |
| CASP8AP2 | 3.43E+00 | 6.10E-04 | 1.72E-03 |
| HHATL | -3.43E+00 | 6.11E-04 | 1.73E-03 |
| C7orf50 | 3.43E+00 | 6.12E-04 | 1.73E-03 |
| TBC1D19 | -3.43E+00 | 6.12E-04 | 1.73E-03 |
| ZBTB10 | 3.43E+00 | 6.12E-04 | 1.73E-03 |
| TICAM1 | 3.43E+00 | 6.12E-04 | 1.73E-03 |
| TMEM14C | -3.43E+00 | 6.15E-04 | 1.74E-03 |
| PIAS3 | 3.42E+00 | 6.16E-04 | 1.74E-03 |
| FBXO30 | 3.42E+00 | 6.19E-04 | 1.75E-03 |
| CYB5D1 | -3.42E+00 | 6.19E-04 | 1.75E-03 |
| ZNF584 | -3.42E+00 | 6.20E-04 | 1.75E-03 |
| RPGRIP1L | 3.42E+00 | 6.21E-04 | 1.75E-03 |
| LOC100125556 | 3.42E+00 | 6.23E-04 | 1.76E-03 |
| FLJ33360 | -3.42E+00 | 6.23E-04 | 1.76E-03 |
| SOAT2 | 3.42E+00 | 6.24E-04 | 1.76E-03 |
| TRIT1 | 3.42E+00 | 6.25E-04 | 1.76E-03 |
| BAI1 | 3.42E+00 | 6.29E-04 | 1.77E-03 |
| PLA2G7 | 3.42E+00 | 6.29E-04 | 1.77E-03 |
| MAP2K6 | 3.42E+00 | 6.29E-04 | 1.77E-03 |
| RAB40B | -3.42E+00 | 6.30E-04 | 1.77E-03 |
| HOXC11 | 3.42E+00 | 6.30E-04 | 1.77E-03 |
| TARS2 | 3.42E+00 | 6.31E-04 | 1.78E-03 |
| GSG1L | -3.42E+00 | 6.31E-04 | 1.78E-03 |
| DKK3 | -3.42E+00 | 6.32E-04 | 1.78E-03 |
| DMRT1 | 3.42E+00 | 6.32E-04 | 1.78E-03 |
| LOC284100 | 3.42E+00 | 6.33E-04 | 1.78E-03 |
| ETFA | -3.42E+00 | 6.34E-04 | 1.78E-03 |
| HMG20B | 3.42E+00 | 6.35E-04 | 1.79E-03 |
| MRPS18C | -3.42E+00 | 6.35E-04 | 1.79E-03 |
| MIDN | 3.42E+00 | 6.35E-04 | 1.79E-03 |
| CREM | -3.42E+00 | 6.36E-04 | 1.79E-03 |
| LHCGR | -3.42E+00 | 6.36E-04 | 1.79E-03 |
| C2CD4A | 3.42E+00 | 6.36E-04 | 1.79E-03 |
| FOXJ1 | 3.42E+00 | 6.36E-04 | 1.79E-03 |
| KIAA1199 | 3.42E+00 | 6.36E-04 | 1.79E-03 |
| MTMR1 | 3.42E+00 | 6.37E-04 | 1.79E-03 |
| PPM1B | -3.42E+00 | 6.37E-04 | 1.79E-03 |
| CAPN2 | -3.41E+00 | 6.41E-04 | 1.80E-03 |
| GAL3ST1 | 3.41E+00 | 6.42E-04 | 1.80E-03 |
| LOC100144603 | 3.41E+00 | 6.42E-04 | 1.80E-03 |
| RPL21P44 | -3.41E+00 | 6.43E-04 | 1.81E-03 |

| SPATS1 | -3.41E+00 | 6.44E-04 | 1.81E-03 |
| --- | --- | --- | --- |
| INPP5E | 3.41E+00 | 6.45E-04 | 1.81E-03 |
| SGIP1 | -3.41E+00 | 6.47E-04 | 1.82E-03 |
| CDH19 | -3.41E+00 | 6.48E-04 | 1.82E-03 |
| CAPRIN2 | 3.41E+00 | 6.49E-04 | 1.82E-03 |
| PNMA5 | 3.41E+00 | 6.52E-04 | 1.83E-03 |
| TRIM45 | 3.41E+00 | 6.52E-04 | 1.83E-03 |
| OPHN1 | -3.41E+00 | 6.53E-04 | 1.83E-03 |
| VSX2 | 3.41E+00 | 6.52E-04 | 1.83E-03 |
| AP1S3 | 3.41E+00 | 6.54E-04 | 1.83E-03 |
| DUSP7 | 3.41E+00 | 6.54E-04 | 1.83E-03 |
| SDSL | 3.41E+00 | 6.56E-04 | 1.84E-03 |
| LOC729082 | -3.41E+00 | 6.56E-04 | 1.84E-03 |
| C21orf82 | -3.41E+00 | 6.56E-04 | 1.84E-03 |
| CDKL3 | -3.41E+00 | 6.56E-04 | 1.84E-03 |
| NCRNA00235 | 3.41E+00 | 6.57E-04 | 1.84E-03 |
| NRN1 | -3.41E+00 | 6.57E-04 | 1.84E-03 |
| GATA5 | -3.41E+00 | 6.59E-04 | 1.84E-03 |
| NUDT13 | -3.41E+00 | 6.60E-04 | 1.85E-03 |
| ANKRD24 | -3.41E+00 | 6.60E-04 | 1.85E-03 |
| CD70 | 3.41E+00 | 6.60E-04 | 1.85E-03 |
| DPYD | -3.41E+00 | 6.62E-04 | 1.85E-03 |
| OSBPL7 | 3.40E+00 | 6.64E-04 | 1.86E-03 |
| XAGE5 | 3.40E+00 | 6.66E-04 | 1.86E-03 |
| C16orf72 | -3.40E+00 | 6.66E-04 | 1.86E-03 |
| CCM2 | 3.40E+00 | 6.66E-04 | 1.86E-03 |
| LGR4 | -3.40E+00 | 6.67E-04 | 1.87E-03 |
| HYI | -3.40E+00 | 6.68E-04 | 1.87E-03 |
| TFAP2E | 3.40E+00 | 6.69E-04 | 1.87E-03 |
| OPRL1 | 3.40E+00 | 6.69E-04 | 1.87E-03 |
| CRYZ | -3.40E+00 | 6.70E-04 | 1.87E-03 |
| NEFL | 3.40E+00 | 6.72E-04 | 1.88E-03 |
| REEP1 | -3.40E+00 | 6.73E-04 | 1.88E-03 |
| C1orf114 | 3.40E+00 | 6.73E-04 | 1.88E-03 |
| TMEM231 | -3.40E+00 | 6.74E-04 | 1.88E-03 |
| DCAF12L1 | 3.40E+00 | 6.75E-04 | 1.89E-03 |
| LRAT | -3.40E+00 | 6.75E-04 | 1.89E-03 |
| TRIM35 | -3.40E+00 | 6.76E-04 | 1.89E-03 |
| DEPDC6 | -3.40E+00 | 6.76E-04 | 1.89E-03 |
| SERPINF2 | -3.40E+00 | 6.77E-04 | 1.89E-03 |
| ANKRD36B | 3.40E+00 | 6.78E-04 | 1.89E-03 |
| SYF2 | -3.40E+00 | 6.78E-04 | 1.89E-03 |
| SOX8 | -3.40E+00 | 6.80E-04 | 1.90E-03 |
| ENPP4 | -3.40E+00 | 6.81E-04 | 1.90E-03 |
| PRCD | -3.40E+00 | 6.81E-04 | 1.90E-03 |
| SYNJ1 | -3.40E+00 | 6.82E-04 | 1.90E-03 |
| GCAT | -3.40E+00 | 6.82E-04 | 1.90E-03 |
| COL6A6 | -3.40E+00 | 6.84E-04 | 1.91E-03 |
| CCDC82 | -3.40E+00 | 6.86E-04 | 1.91E-03 |
| FAM26D | 3.39E+00 | 6.88E-04 | 1.92E-03 |
| FAM118A | 3.39E+00 | 6.90E-04 | 1.92E-03 |
| UROC1 | -3.39E+00 | 6.90E-04 | 1.92E-03 |
| SPATA2L | 3.39E+00 | 6.92E-04 | 1.93E-03 |
| C4orf27 | -3.39E+00 | 6.93E-04 | 1.93E-03 |
| YIPF1 | -3.39E+00 | 6.94E-04 | 1.93E-03 |
| NTN4 | -3.39E+00 | 6.94E-04 | 1.93E-03 |
| MASP2 | -3.39E+00 | 6.95E-04 | 1.94E-03 |

| TEKT4 | -3.39E+00 | 6.96E-04 | 1.94E-03 |
| --- | --- | --- | --- |
| OST4 | -3.39E+00 | 6.97E-04 | 1.94E-03 |
| SEMA6A | 3.39E+00 | 6.97E-04 | 1.94E-03 |
| ANKRD16 | 3.39E+00 | 6.99E-04 | 1.94E-03 |
| RARRES2 | -3.39E+00 | 7.01E-04 | 1.95E-03 |
| GALK1 | 3.39E+00 | 7.01E-04 | 1.95E-03 |
| LCN1 | 3.39E+00 | 7.02E-04 | 1.95E-03 |
| ZNF154 | -3.39E+00 | 7.03E-04 | 1.96E-03 |
| YIPF4 | -3.39E+00 | 7.04E-04 | 1.96E-03 |
| IL12A | 3.39E+00 | 7.05E-04 | 1.96E-03 |
| DMP1 | 3.39E+00 | 7.10E-04 | 1.97E-03 |
| LOC643387 | 3.39E+00 | 7.11E-04 | 1.98E-03 |
| DEFA6 | 3.39E+00 | 7.12E-04 | 1.98E-03 |
| OMP | 3.38E+00 | 7.13E-04 | 1.98E-03 |
| SNX15 | 3.38E+00 | 7.14E-04 | 1.98E-03 |
| NTSR1 | 3.38E+00 | 7.14E-04 | 1.98E-03 |
| TM9SF2 | -3.38E+00 | 7.15E-04 | 1.99E-03 |
| GSK3B | 3.38E+00 | 7.17E-04 | 1.99E-03 |
| BBS9 | -3.38E+00 | 7.17E-04 | 1.99E-03 |
| MOBKL2B | -3.38E+00 | 7.18E-04 | 1.99E-03 |
| TEX101 | 3.38E+00 | 7.23E-04 | 2.01E-03 |
| TNMD | -3.38E+00 | 7.24E-04 | 2.01E-03 |
| INTS4L2 | 3.38E+00 | 7.25E-04 | 2.01E-03 |
| DENND4C | -3.38E+00 | 7.25E-04 | 2.01E-03 |
| RINT1 | 3.38E+00 | 7.26E-04 | 2.02E-03 |
| UBXN4 | -3.38E+00 | 7.27E-04 | 2.02E-03 |
| WDR5B | -3.38E+00 | 7.31E-04 | 2.03E-03 |
| SYT4 | 3.38E+00 | 7.32E-04 | 2.03E-03 |
| ZNF322B | -3.38E+00 | 7.32E-04 | 2.03E-03 |
| TMEM128 | -3.38E+00 | 7.33E-04 | 2.03E-03 |
| RHOBTB1 | 3.38E+00 | 7.36E-04 | 2.04E-03 |
| IRX4 | 3.38E+00 | 7.36E-04 | 2.04E-03 |
| C1orf97 | -3.38E+00 | 7.37E-04 | 2.04E-03 |
| ZNF428 | 3.38E+00 | 7.39E-04 | 2.05E-03 |
| ZBTB25 | -3.37E+00 | 7.40E-04 | 2.05E-03 |
| TULP4 | 3.37E+00 | 7.41E-04 | 2.05E-03 |
| WDR89 | -3.37E+00 | 7.41E-04 | 2.05E-03 |
| GRAMD1C | -3.37E+00 | 7.42E-04 | 2.05E-03 |
| CDH17 | 3.37E+00 | 7.42E-04 | 2.05E-03 |
| LOC441177 | 3.37E+00 | 7.44E-04 | 2.06E-03 |
| PTGR2 | -3.37E+00 | 7.45E-04 | 2.06E-03 |
| TMEM177 | 3.37E+00 | 7.47E-04 | 2.07E-03 |
| INSIG1 | -3.37E+00 | 7.48E-04 | 2.07E-03 |
| GOLGA6L1 | 3.37E+00 | 7.50E-04 | 2.08E-03 |
| ZNF132 | -3.37E+00 | 7.51E-04 | 2.08E-03 |
| ZNF169 | 3.37E+00 | 7.51E-04 | 2.08E-03 |
| NEK1 | -3.37E+00 | 7.52E-04 | 2.08E-03 |
| KDM5A | 3.37E+00 | 7.52E-04 | 2.08E-03 |
| AHCYL1 | -3.37E+00 | 7.52E-04 | 2.08E-03 |
| TGFBR2 | -3.37E+00 | 7.53E-04 | 2.08E-03 |
| LCLAT1 | 3.37E+00 | 7.56E-04 | 2.09E-03 |
| ETNK2 | -3.37E+00 | 7.56E-04 | 2.09E-03 |
| ZNF519 | 3.37E+00 | 7.56E-04 | 2.09E-03 |
| MYH1 | -3.37E+00 | 7.57E-04 | 2.09E-03 |
| CLEC18B | 3.37E+00 | 7.58E-04 | 2.09E-03 |
| ATF3 | -3.37E+00 | 7.59E-04 | 2.10E-03 |
| COL4A3BP | -3.37E+00 | 7.61E-04 | 2.10E-03 |

| TMEM217 | -3.37E+00 | 7.61E-04 | 2.10E-03 |
| --- | --- | --- | --- |
| COL29A1 | -3.37E+00 | 7.62E-04 | 2.10E-03 |
| INPP5K | -3.37E+00 | 7.66E-04 | 2.11E-03 |
| GDF2 | -3.37E+00 | 7.66E-04 | 2.11E-03 |
| SLC6A16 | -3.37E+00 | 7.67E-04 | 2.12E-03 |
| NDUFB6 | -3.37E+00 | 7.67E-04 | 2.12E-03 |
| PSMG2 | -3.36E+00 | 7.68E-04 | 2.12E-03 |
| GRIN2B | 3.36E+00 | 7.71E-04 | 2.13E-03 |
| CBLL1 | 3.36E+00 | 7.71E-04 | 2.13E-03 |
| KLC1 | 3.36E+00 | 7.73E-04 | 2.13E-03 |
| RNH1 | 3.36E+00 | 7.73E-04 | 2.13E-03 |
| SGK269 | -3.36E+00 | 7.73E-04 | 2.13E-03 |
| UBE2L3 | 3.36E+00 | 7.75E-04 | 2.14E-03 |
| LUZP6 | 3.36E+00 | 7.76E-04 | 2.14E-03 |
| ZNF396 | -3.36E+00 | 7.77E-04 | 2.14E-03 |
| RBM6 | 3.36E+00 | 7.79E-04 | 2.15E-03 |
| SAMD14 | 3.36E+00 | 7.81E-04 | 2.15E-03 |
| CLEC4F | -3.36E+00 | 7.81E-04 | 2.15E-03 |
| CMAS | 3.36E+00 | 7.83E-04 | 2.16E-03 |
| PSKH1 | 3.36E+00 | 7.84E-04 | 2.16E-03 |
| LIPJ | -3.36E+00 | 7.86E-04 | 2.16E-03 |
| ENGASE | 3.36E+00 | 7.87E-04 | 2.17E-03 |
| PEX5L | 3.36E+00 | 7.88E-04 | 2.17E-03 |
| C21orf67 | -3.36E+00 | 7.89E-04 | 2.17E-03 |
| STAP2 | -3.36E+00 | 7.89E-04 | 2.17E-03 |
| GABPB2 | -3.36E+00 | 7.90E-04 | 2.17E-03 |
| DEF6 | 3.36E+00 | 7.91E-04 | 2.18E-03 |
| PI16 | -3.36E+00 | 7.93E-04 | 2.18E-03 |
| PDZD8 | 3.36E+00 | 7.95E-04 | 2.19E-03 |
| TMEM30A | -3.35E+00 | 7.95E-04 | 2.19E-03 |
| LNX1 | -3.35E+00 | 7.99E-04 | 2.20E-03 |
| ZNF555 | -3.35E+00 | 7.99E-04 | 2.20E-03 |
| PTPDC1 | 3.35E+00 | 8.00E-04 | 2.20E-03 |
| TRMT12 | 3.35E+00 | 8.02E-04 | 2.21E-03 |
| TTC24 | 3.35E+00 | 8.04E-04 | 2.21E-03 |
| SORL1 | -3.35E+00 | 8.04E-04 | 2.21E-03 |
| KCND3 | -3.35E+00 | 8.05E-04 | 2.21E-03 |
| SLCO2B1 | -3.35E+00 | 8.05E-04 | 2.21E-03 |
| FGD5 | -3.35E+00 | 8.06E-04 | 2.21E-03 |
| NDUFS4 | -3.35E+00 | 8.06E-04 | 2.21E-03 |
| ZNF557 | -3.35E+00 | 8.06E-04 | 2.21E-03 |
| JAKMIP1 | 3.35E+00 | 8.07E-04 | 2.21E-03 |
| SHH | -3.35E+00 | 8.08E-04 | 2.22E-03 |
| C6orf27 | 3.35E+00 | 8.09E-04 | 2.22E-03 |
| LEAP2 | -3.35E+00 | 8.10E-04 | 2.22E-03 |
| COX4I1 | -3.35E+00 | 8.11E-04 | 2.23E-03 |
| PIM3 | 3.35E+00 | 8.13E-04 | 2.23E-03 |
| OR12D2 | 3.35E+00 | 8.13E-04 | 2.23E-03 |
| PXDNL | 3.35E+00 | 8.13E-04 | 2.23E-03 |
| SRGAP2 | 3.35E+00 | 8.15E-04 | 2.24E-03 |
| SNRPD2 | 3.35E+00 | 8.18E-04 | 2.24E-03 |
| ATG16L1 | 3.35E+00 | 8.19E-04 | 2.24E-03 |
| SLC44A2 | -3.35E+00 | 8.19E-04 | 2.24E-03 |
| CPD | 3.35E+00 | 8.20E-04 | 2.25E-03 |
| PLLP | -3.35E+00 | 8.23E-04 | 2.26E-03 |
| ERVFRDE1 | -3.35E+00 | 8.23E-04 | 2.26E-03 |
| RBBP6 | 3.35E+00 | 8.24E-04 | 2.26E-03 |

| TXLNG | 3.35E+00 | 8.24E-04 | 2.26E-03 |
| --- | --- | --- | --- |
| CAMK1D | -3.34E+00 | 8.24E-04 | 2.26E-03 |
| RBM7 | -3.34E+00 | 8.25E-04 | 2.26E-03 |
| SHISA6 | -3.34E+00 | 8.25E-04 | 2.26E-03 |
| IL2RB | 3.34E+00 | 8.25E-04 | 2.26E-03 |
| MTHFD2L | -3.34E+00 | 8.25E-04 | 2.26E-03 |
| TUBA4A | 3.34E+00 | 8.26E-04 | 2.26E-03 |
| OR2H1 | 3.34E+00 | 8.27E-04 | 2.26E-03 |
| GKAP1 | -3.34E+00 | 8.30E-04 | 2.27E-03 |
| CLK2 | 3.34E+00 | 8.30E-04 | 2.27E-03 |
| CDR2L | 3.34E+00 | 8.31E-04 | 2.27E-03 |
| ARL5A | -3.34E+00 | 8.34E-04 | 2.28E-03 |
| UBD | 3.34E+00 | 8.36E-04 | 2.29E-03 |
| PARP11 | -3.34E+00 | 8.38E-04 | 2.29E-03 |
| ZBTB4 | -3.34E+00 | 8.39E-04 | 2.29E-03 |
| C6orf225 | -3.34E+00 | 8.39E-04 | 2.29E-03 |
| CATSPERG | -3.34E+00 | 8.40E-04 | 2.29E-03 |
| CDYL2 | 3.34E+00 | 8.39E-04 | 2.29E-03 |
| IL15RA | 3.34E+00 | 8.39E-04 | 2.29E-03 |
| LDHD | -3.34E+00 | 8.40E-04 | 2.29E-03 |
| LRRC16B | 3.34E+00 | 8.40E-04 | 2.30E-03 |
| PHF16 | 3.34E+00 | 8.41E-04 | 2.30E-03 |
| SNAP25 | 3.34E+00 | 8.41E-04 | 2.30E-03 |
| DNAH9 | -3.34E+00 | 8.42E-04 | 2.30E-03 |
| ASB7 | -3.34E+00 | 8.44E-04 | 2.31E-03 |
| TTLL8 | 3.34E+00 | 8.45E-04 | 2.31E-03 |
| COPG2 | 3.34E+00 | 8.46E-04 | 2.31E-03 |
| BZRAP1 | -3.34E+00 | 8.48E-04 | 2.31E-03 |
| CXCL12 | -3.34E+00 | 8.50E-04 | 2.32E-03 |
| MFHAS1 | 3.34E+00 | 8.52E-04 | 2.32E-03 |
| UIMC1 | 3.34E+00 | 8.53E-04 | 2.33E-03 |
| WNT6 | 3.33E+00 | 8.55E-04 | 2.33E-03 |
| SLC20A2 | -3.33E+00 | 8.55E-04 | 2.33E-03 |
| ODF3L1 | -3.33E+00 | 8.56E-04 | 2.33E-03 |
| C4orf14 | 3.33E+00 | 8.57E-04 | 2.34E-03 |
| KIAA0467 | 3.33E+00 | 8.58E-04 | 2.34E-03 |
| ALPI | 3.33E+00 | 8.59E-04 | 2.34E-03 |
| PIK3R6 | 3.33E+00 | 8.63E-04 | 2.35E-03 |
| EXOSC8 | 3.33E+00 | 8.68E-04 | 2.36E-03 |
| CHAC1 | 3.33E+00 | 8.68E-04 | 2.37E-03 |
| ADC | -3.33E+00 | 8.71E-04 | 2.37E-03 |
| RUNX1T1 | -3.33E+00 | 8.73E-04 | 2.38E-03 |
| BBOX1 | -3.33E+00 | 8.74E-04 | 2.38E-03 |
| C3orf54 | -3.33E+00 | 8.74E-04 | 2.38E-03 |
| KIAA0146 | 3.33E+00 | 8.76E-04 | 2.39E-03 |
| TOB2 | -3.33E+00 | 8.77E-04 | 2.39E-03 |
| FAM81A | 3.33E+00 | 8.81E-04 | 2.40E-03 |
| PCDHA9 | 3.33E+00 | 8.83E-04 | 2.40E-03 |
| BOLA3 | 3.33E+00 | 8.83E-04 | 2.40E-03 |
| C9orf24 | -3.33E+00 | 8.84E-04 | 2.40E-03 |
| KMO | -3.33E+00 | 8.84E-04 | 2.40E-03 |
| ZDHHC5 | 3.33E+00 | 8.84E-04 | 2.40E-03 |
| AGL | -3.33E+00 | 8.85E-04 | 2.41E-03 |
| PPBP | -3.33E+00 | 8.85E-04 | 2.41E-03 |
| PIP4K2A | 3.33E+00 | 8.85E-04 | 2.41E-03 |
| MPV17L2 | 3.32E+00 | 8.89E-04 | 2.42E-03 |
| VPS53 | 3.32E+00 | 8.90E-04 | 2.42E-03 |

| ZNF581 | 3.32E+00 | 8.91E-04 | 2.42E-03 |
| --- | --- | --- | --- |
| ZDHHC11 | -3.32E+00 | 8.92E-04 | 2.42E-03 |
| LYPD6 | 3.32E+00 | 8.93E-04 | 2.43E-03 |
| HOTAIR | 3.32E+00 | 8.94E-04 | 2.43E-03 |
| FFAR1 | 3.32E+00 | 8.95E-04 | 2.43E-03 |
| MMADHC | -3.32E+00 | 8.96E-04 | 2.43E-03 |
| ATP1B2 | -3.32E+00 | 8.96E-04 | 2.43E-03 |
| TRIM4 | -3.32E+00 | 8.98E-04 | 2.44E-03 |
| DCAF4 | 3.32E+00 | 8.99E-04 | 2.44E-03 |
| TBRG1 | -3.32E+00 | 9.00E-04 | 2.44E-03 |
| FGF13 | -3.32E+00 | 9.01E-04 | 2.45E-03 |
| KDELR2 | -3.32E+00 | 9.03E-04 | 2.45E-03 |
| GNB5 | -3.32E+00 | 9.04E-04 | 2.45E-03 |
| KRT78 | 3.32E+00 | 9.04E-04 | 2.45E-03 |
| LOC283174 | -3.32E+00 | 9.04E-04 | 2.45E-03 |
| SPTA1 | 3.32E+00 | 9.04E-04 | 2.45E-03 |
| STAG3L4 | 3.32E+00 | 9.06E-04 | 2.46E-03 |
| C11orf86 | 3.32E+00 | 9.07E-04 | 2.46E-03 |
| OR52E6 | 3.32E+00 | 9.07E-04 | 2.46E-03 |
| HTR6 | 3.32E+00 | 9.10E-04 | 2.47E-03 |
| NDUFA6 | -3.32E+00 | 9.11E-04 | 2.47E-03 |
| ATP1B1 | -3.32E+00 | 9.12E-04 | 2.47E-03 |
| DPYSL2 | -3.32E+00 | 9.13E-04 | 2.47E-03 |
| UPK1B | 3.32E+00 | 9.14E-04 | 2.48E-03 |
| GIPC1 | 3.32E+00 | 9.14E-04 | 2.48E-03 |
| NTRK1 | 3.32E+00 | 9.15E-04 | 2.48E-03 |
| SERPINC1 | -3.32E+00 | 9.15E-04 | 2.48E-03 |
| NPHS2 | 3.31E+00 | 9.19E-04 | 2.49E-03 |
| KRT222 | -3.31E+00 | 9.21E-04 | 2.49E-03 |
| KLHL32 | -3.31E+00 | 9.22E-04 | 2.49E-03 |
| PMS1 | 3.31E+00 | 9.22E-04 | 2.49E-03 |
| ST7OT1 | -3.31E+00 | 9.22E-04 | 2.49E-03 |
| ASCC3 | 3.31E+00 | 9.23E-04 | 2.50E-03 |
| DCAF12L2 | 3.31E+00 | 9.25E-04 | 2.50E-03 |
| NPTX2 | 3.31E+00 | 9.25E-04 | 2.50E-03 |
| DKK1 | 3.31E+00 | 9.25E-04 | 2.50E-03 |
| C6orf114 | -3.31E+00 | 9.26E-04 | 2.51E-03 |
| CNNM4 | 3.31E+00 | 9.32E-04 | 2.52E-03 |
| PRKCZ | -3.31E+00 | 9.32E-04 | 2.52E-03 |
| RNASE7 | -3.31E+00 | 9.34E-04 | 2.53E-03 |
| MAGI1 | -3.31E+00 | 9.35E-04 | 2.53E-03 |
| ODZ3 | 3.31E+00 | 9.35E-04 | 2.53E-03 |
| SH3GLB2 | 3.31E+00 | 9.35E-04 | 2.53E-03 |
| OR4N4 | 3.31E+00 | 9.36E-04 | 2.53E-03 |
| RIC8A | 3.31E+00 | 9.36E-04 | 2.53E-03 |
| GALNTL5 | 3.31E+00 | 9.38E-04 | 2.53E-03 |
| HDX | -3.31E+00 | 9.42E-04 | 2.54E-03 |
| TIGD3 | 3.31E+00 | 9.42E-04 | 2.54E-03 |
| WISP1 | 3.31E+00 | 9.42E-04 | 2.54E-03 |
| C12orf4 | 3.31E+00 | 9.43E-04 | 2.55E-03 |
| SLC16A1 | 3.31E+00 | 9.44E-04 | 2.55E-03 |
| DLX5 | 3.31E+00 | 9.44E-04 | 2.55E-03 |
| TMEM131 | 3.31E+00 | 9.45E-04 | 2.55E-03 |
| FAM134C | -3.31E+00 | 9.45E-04 | 2.55E-03 |
| FGD3 | 3.31E+00 | 9.46E-04 | 2.55E-03 |
| TMEM179B | -3.31E+00 | 9.48E-04 | 2.56E-03 |
| LRP10 | 3.31E+00 | 9.51E-04 | 2.57E-03 |

| ZNF833 | -3.30E+00 | 9.55E-04 | 2.58E-03 |
| --- | --- | --- | --- |
| ZNF500 | 3.30E+00 | 9.56E-04 | 2.58E-03 |
| EFNB3 | -3.30E+00 | 9.58E-04 | 2.58E-03 |
| KCNB1 | -3.30E+00 | 9.61E-04 | 2.59E-03 |
| KDM3B | 3.30E+00 | 9.62E-04 | 2.59E-03 |
| RPS6KA5 | -3.30E+00 | 9.63E-04 | 2.60E-03 |
| SLC30A6 | 3.30E+00 | 9.64E-04 | 2.60E-03 |
| CD36 | -3.30E+00 | 9.66E-04 | 2.60E-03 |
| CSMD2 | 3.30E+00 | 9.69E-04 | 2.61E-03 |
| SNORA40 | 3.30E+00 | 9.73E-04 | 2.62E-03 |
| ANO2 | -3.30E+00 | 9.75E-04 | 2.63E-03 |
| IL8 | 3.30E+00 | 9.75E-04 | 2.63E-03 |
| SH3RF1 | -3.30E+00 | 9.75E-04 | 2.63E-03 |
| ARRDC3 | -3.30E+00 | 9.77E-04 | 2.63E-03 |
| OBP2A | 3.30E+00 | 9.78E-04 | 2.63E-03 |
| CRB3 | -3.30E+00 | 9.80E-04 | 2.64E-03 |
| C17orf85 | 3.30E+00 | 9.81E-04 | 2.64E-03 |
| TNFAIP3 | 3.30E+00 | 9.83E-04 | 2.64E-03 |
| SDF2L1 | 3.30E+00 | 9.85E-04 | 2.65E-03 |
| OAS1 | 3.30E+00 | 9.86E-04 | 2.65E-03 |
| SAP30 | 3.29E+00 | 9.86E-04 | 2.65E-03 |
| COL17A1 | -3.29E+00 | 9.87E-04 | 2.65E-03 |
| STYX | -3.29E+00 | 9.87E-04 | 2.65E-03 |
| PODN | -3.29E+00 | 9.90E-04 | 2.66E-03 |
| GPX4 | -3.29E+00 | 9.91E-04 | 2.66E-03 |
| HHIPL1 | 3.29E+00 | 9.91E-04 | 2.66E-03 |
| GCG | 3.29E+00 | 1.00E-03 | 2.69E-03 |
| ASPHD1 | 3.29E+00 | 1.00E-03 | 2.70E-03 |
| OR51G2 | 3.29E+00 | 1.00E-03 | 2.70E-03 |
| BCAS2 | -3.29E+00 | 1.01E-03 | 2.71E-03 |
| WDR87 | 3.29E+00 | 1.01E-03 | 2.71E-03 |
| APIP | -3.29E+00 | 1.01E-03 | 2.71E-03 |
| EVPL | 3.29E+00 | 1.01E-03 | 2.71E-03 |
| ELAVL4 | 3.29E+00 | 1.01E-03 | 2.72E-03 |
| PLEKHG3 | 3.29E+00 | 1.02E-03 | 2.73E-03 |
| BAALC | -3.29E+00 | 1.02E-03 | 2.73E-03 |
| EPS8 | -3.29E+00 | 1.02E-03 | 2.73E-03 |
| ZNF572 | 3.29E+00 | 1.02E-03 | 2.73E-03 |
| GTSF1L | 3.29E+00 | 1.02E-03 | 2.73E-03 |
| SFT2D3 | -3.29E+00 | 1.02E-03 | 2.73E-03 |
| VPS13B | 3.29E+00 | 1.02E-03 | 2.73E-03 |
| ADCY7 | 3.29E+00 | 1.02E-03 | 2.74E-03 |
| OTUD1 | -3.28E+00 | 1.03E-03 | 2.75E-03 |
| GSN | -3.28E+00 | 1.03E-03 | 2.76E-03 |
| PML | 3.28E+00 | 1.03E-03 | 2.76E-03 |
| TROVE2 | -3.28E+00 | 1.03E-03 | 2.77E-03 |
| METRNL | 3.28E+00 | 1.03E-03 | 2.77E-03 |
| SFMBT1 | 3.28E+00 | 1.04E-03 | 2.77E-03 |
| ARFGEF2 | 3.28E+00 | 1.04E-03 | 2.78E-03 |
| LIAS | -3.28E+00 | 1.04E-03 | 2.78E-03 |
| EVX1 | 3.28E+00 | 1.04E-03 | 2.78E-03 |
| SLFN13 | 3.28E+00 | 1.04E-03 | 2.79E-03 |
| SOX11 | 3.28E+00 | 1.05E-03 | 2.80E-03 |
| CYTH3 | -3.28E+00 | 1.05E-03 | 2.80E-03 |
| HOOK3 | -3.28E+00 | 1.05E-03 | 2.80E-03 |
| S100A7 | 3.28E+00 | 1.05E-03 | 2.80E-03 |
| TLCD1 | 3.28E+00 | 1.05E-03 | 2.80E-03 |

| SRP14 | -3.28E+00 | 1.05E-03 | 2.81E-03 |
| --- | --- | --- | --- |
| ZNF18 | -3.28E+00 | 1.05E-03 | 2.81E-03 |
| CST5 | -3.28E+00 | 1.05E-03 | 2.81E-03 |
| NOTCH2NL | -3.28E+00 | 1.05E-03 | 2.81E-03 |
| GPBP1L1 | -3.28E+00 | 1.05E-03 | 2.81E-03 |
| ALPK3 | 3.28E+00 | 1.05E-03 | 2.81E-03 |
| CTSG | -3.28E+00 | 1.05E-03 | 2.82E-03 |
| TTC16 | -3.28E+00 | 1.05E-03 | 2.82E-03 |
| FOXD3 | 3.28E+00 | 1.06E-03 | 2.82E-03 |
| CD248 | 3.28E+00 | 1.06E-03 | 2.83E-03 |
| OSBPL2 | 3.28E+00 | 1.06E-03 | 2.83E-03 |
| CDH1 | -3.27E+00 | 1.06E-03 | 2.83E-03 |
| TANK | -3.27E+00 | 1.06E-03 | 2.83E-03 |
| ERLEC1 | -3.27E+00 | 1.06E-03 | 2.83E-03 |
| MRGPRX4 | 3.27E+00 | 1.06E-03 | 2.83E-03 |
| CLIC4 | -3.27E+00 | 1.06E-03 | 2.83E-03 |
| LOC151162 | 3.27E+00 | 1.07E-03 | 2.84E-03 |
| NCRNA00173 | 3.27E+00 | 1.07E-03 | 2.85E-03 |
| ANGPT1 | -3.27E+00 | 1.07E-03 | 2.85E-03 |
| C4orf32 | -3.27E+00 | 1.07E-03 | 2.86E-03 |
| CD163L1 | 3.27E+00 | 1.07E-03 | 2.86E-03 |
| HS3ST3A1 | 3.27E+00 | 1.07E-03 | 2.86E-03 |
| PIWIL3 | 3.27E+00 | 1.07E-03 | 2.86E-03 |
| C13orf38 | 3.27E+00 | 1.08E-03 | 2.87E-03 |
| RBMS2 | -3.27E+00 | 1.08E-03 | 2.87E-03 |
| KLRC2 | 3.27E+00 | 1.08E-03 | 2.88E-03 |
| CCL3L1 | 3.27E+00 | 1.08E-03 | 2.88E-03 |
| LILRB3 | 3.27E+00 | 1.08E-03 | 2.89E-03 |
| ARHGEF5 | 3.27E+00 | 1.08E-03 | 2.89E-03 |
| GABRB1 | 3.27E+00 | 1.09E-03 | 2.89E-03 |
| URM1 | 3.27E+00 | 1.09E-03 | 2.89E-03 |
| BRD1 | 3.27E+00 | 1.09E-03 | 2.89E-03 |
| ZNF615 | -3.27E+00 | 1.09E-03 | 2.90E-03 |
| TGM4 | 3.27E+00 | 1.09E-03 | 2.90E-03 |
| PPIA | 3.27E+00 | 1.09E-03 | 2.90E-03 |
| GOSR1 | 3.27E+00 | 1.09E-03 | 2.91E-03 |
| HAUS1 | 3.27E+00 | 1.09E-03 | 2.91E-03 |
| MAP3K12 | 3.27E+00 | 1.09E-03 | 2.91E-03 |
| TMUB2 | 3.27E+00 | 1.09E-03 | 2.91E-03 |
| ST3GAL3 | -3.26E+00 | 1.10E-03 | 2.93E-03 |
| LBX1 | 3.26E+00 | 1.10E-03 | 2.93E-03 |
| ITPR1 | -3.26E+00 | 1.10E-03 | 2.93E-03 |
| HERC5 | -3.26E+00 | 1.11E-03 | 2.94E-03 |
| CNDP2 | -3.26E+00 | 1.11E-03 | 2.95E-03 |
| FAM155B | 3.26E+00 | 1.11E-03 | 2.96E-03 |
| NCRNA00152 | 3.26E+00 | 1.11E-03 | 2.96E-03 |
| C10orf55 | 3.26E+00 | 1.11E-03 | 2.96E-03 |
| GFER | 3.26E+00 | 1.11E-03 | 2.96E-03 |
| RSBN1 | -3.26E+00 | 1.11E-03 | 2.96E-03 |
| NMNAT1 | -3.26E+00 | 1.12E-03 | 2.96E-03 |
| MMP25 | 3.26E+00 | 1.12E-03 | 2.96E-03 |
| BRP44 | -3.26E+00 | 1.12E-03 | 2.97E-03 |
| HGSNAT | -3.26E+00 | 1.12E-03 | 2.98E-03 |
| ZNF277 | -3.26E+00 | 1.12E-03 | 2.98E-03 |
| BLVRB | -3.26E+00 | 1.12E-03 | 2.98E-03 |
| ENDOU | -3.26E+00 | 1.12E-03 | 2.98E-03 |
| C19orf36 | -3.26E+00 | 1.12E-03 | 2.98E-03 |

| RBMY1A1 | 3.26E+00 | 1.13E-03 | 3.00E-03 |
| --- | --- | --- | --- |
| ZNF33B | -3.26E+00 | 1.13E-03 | 3.00E-03 |
| SH3BP5 | -3.26E+00 | 1.13E-03 | 3.00E-03 |
| TMC7 | 3.26E+00 | 1.13E-03 | 3.01E-03 |
| EYA3 | 3.26E+00 | 1.14E-03 | 3.01E-03 |
| SPAG9 | -3.26E+00 | 1.14E-03 | 3.01E-03 |
| C21orf2 | -3.25E+00 | 1.14E-03 | 3.01E-03 |
| BHLHB9 | -3.25E+00 | 1.14E-03 | 3.02E-03 |
| RGAG4 | -3.25E+00 | 1.14E-03 | 3.03E-03 |
| VMAC | -3.25E+00 | 1.14E-03 | 3.03E-03 |
| LOC619207 | 3.25E+00 | 1.15E-03 | 3.04E-03 |
| CWC15 | -3.25E+00 | 1.15E-03 | 3.04E-03 |
| BCAR4 | 3.25E+00 | 1.15E-03 | 3.04E-03 |
| PAQR3 | -3.25E+00 | 1.15E-03 | 3.04E-03 |
| DDC | 3.25E+00 | 1.15E-03 | 3.05E-03 |
| TAS2R46 | 3.25E+00 | 1.15E-03 | 3.05E-03 |
| SNORA71A | 3.25E+00 | 1.15E-03 | 3.05E-03 |
| METTL4 | 3.25E+00 | 1.16E-03 | 3.07E-03 |
| DNAL1 | -3.25E+00 | 1.16E-03 | 3.07E-03 |
| SRRD | 3.25E+00 | 1.16E-03 | 3.07E-03 |
| TPSAB1 | -3.25E+00 | 1.16E-03 | 3.07E-03 |
| FIBCD1 | 3.25E+00 | 1.16E-03 | 3.07E-03 |
| NCRNA00115 | 3.25E+00 | 1.16E-03 | 3.08E-03 |
| FLNA | 3.25E+00 | 1.16E-03 | 3.08E-03 |
| NUP35 | 3.25E+00 | 1.16E-03 | 3.08E-03 |
| ALAS1 | -3.25E+00 | 1.17E-03 | 3.08E-03 |
| TRAPPC2P1 | -3.25E+00 | 1.17E-03 | 3.08E-03 |
| LSS | 3.25E+00 | 1.18E-03 | 3.11E-03 |
| VSIG10 | 3.25E+00 | 1.18E-03 | 3.11E-03 |
| PZP | -3.25E+00 | 1.18E-03 | 3.11E-03 |
| C3orf30 | 3.24E+00 | 1.18E-03 | 3.11E-03 |
| ADAM11 | 3.24E+00 | 1.18E-03 | 3.11E-03 |
| ZNF70 | 3.24E+00 | 1.18E-03 | 3.12E-03 |
| NRIP3 | 3.24E+00 | 1.18E-03 | 3.13E-03 |
| PIK3R3 | -3.24E+00 | 1.19E-03 | 3.13E-03 |
| TMED9 | 3.24E+00 | 1.19E-03 | 3.13E-03 |
| GCHFR | -3.24E+00 | 1.19E-03 | 3.14E-03 |
| B3GALTL | -3.24E+00 | 1.19E-03 | 3.14E-03 |
| TMEM150B | 3.24E+00 | 1.19E-03 | 3.14E-03 |
| GRRP1 | -3.24E+00 | 1.19E-03 | 3.15E-03 |
| PARVB | 3.24E+00 | 1.19E-03 | 3.15E-03 |
| PLEKHA4 | 3.24E+00 | 1.19E-03 | 3.15E-03 |
| C17orf59 | 3.24E+00 | 1.20E-03 | 3.15E-03 |
| HSF2 | 3.24E+00 | 1.20E-03 | 3.15E-03 |
| SUSD2 | -3.24E+00 | 1.20E-03 | 3.16E-03 |
| CD82 | -3.24E+00 | 1.20E-03 | 3.16E-03 |
| METTL6 | 3.24E+00 | 1.20E-03 | 3.16E-03 |
| RNASEL | -3.24E+00 | 1.20E-03 | 3.16E-03 |
| SDC2 | -3.24E+00 | 1.20E-03 | 3.16E-03 |
| FTSJD1 | -3.24E+00 | 1.20E-03 | 3.16E-03 |
| PLCXD1 | 3.24E+00 | 1.20E-03 | 3.16E-03 |
| FRG2 | 3.24E+00 | 1.20E-03 | 3.17E-03 |
| F8A1 | 3.24E+00 | 1.20E-03 | 3.17E-03 |
| CLLU1OS | 3.24E+00 | 1.21E-03 | 3.18E-03 |
| VPS13D | -3.24E+00 | 1.21E-03 | 3.18E-03 |
| CHST8 | -3.24E+00 | 1.21E-03 | 3.19E-03 |
| NBEAL1 | -3.24E+00 | 1.21E-03 | 3.19E-03 |

| LRRC8C | -3.24E+00 | 1.21E-03 | 3.20E-03 |
| --- | --- | --- | --- |
| ASTL | 3.24E+00 | 1.22E-03 | 3.20E-03 |
| PLCL2 | -3.24E+00 | 1.22E-03 | 3.20E-03 |
| POTEA | 3.24E+00 | 1.22E-03 | 3.21E-03 |
| MRPL13 | 3.23E+00 | 1.22E-03 | 3.21E-03 |
| CDH7 | 3.23E+00 | 1.22E-03 | 3.21E-03 |
| LOC342346 | -3.23E+00 | 1.22E-03 | 3.21E-03 |
| C1orf106 | 3.23E+00 | 1.22E-03 | 3.21E-03 |
| STAM2 | -3.23E+00 | 1.22E-03 | 3.21E-03 |
| C14orf118 | 3.23E+00 | 1.22E-03 | 3.22E-03 |
| CRB2 | 3.23E+00 | 1.23E-03 | 3.22E-03 |
| ATP6AP2 | -3.23E+00 | 1.23E-03 | 3.22E-03 |
| PDE8A | -3.23E+00 | 1.23E-03 | 3.23E-03 |
| IFFO2 | 3.23E+00 | 1.23E-03 | 3.23E-03 |
| SMG1 | 3.23E+00 | 1.23E-03 | 3.23E-03 |
| NCRNA00169 | 3.23E+00 | 1.24E-03 | 3.25E-03 |
| SP140L | 3.23E+00 | 1.24E-03 | 3.25E-03 |
| CFL2 | -3.23E+00 | 1.24E-03 | 3.25E-03 |
| LRRC16A | 3.23E+00 | 1.24E-03 | 3.25E-03 |
| LOC653566 | -3.23E+00 | 1.24E-03 | 3.26E-03 |
| RAP2B | 3.23E+00 | 1.24E-03 | 3.26E-03 |
| TSGA10IP | 3.23E+00 | 1.24E-03 | 3.26E-03 |
| HIST1H2AC | -3.23E+00 | 1.24E-03 | 3.26E-03 |
| MED14 | 3.23E+00 | 1.24E-03 | 3.26E-03 |
| KRTAP19-3 | 3.23E+00 | 1.24E-03 | 3.26E-03 |
| FBXW10 | 3.23E+00 | 1.24E-03 | 3.26E-03 |
| DNAJC22 | 3.23E+00 | 1.25E-03 | 3.27E-03 |
| S100P | 3.23E+00 | 1.25E-03 | 3.27E-03 |
| ESRRG | -3.23E+00 | 1.25E-03 | 3.27E-03 |
| C18orf16 | -3.23E+00 | 1.25E-03 | 3.27E-03 |
| INHBB | -3.23E+00 | 1.25E-03 | 3.27E-03 |
| APPBP2 | -3.23E+00 | 1.25E-03 | 3.28E-03 |
| CRISP1 | 3.23E+00 | 1.25E-03 | 3.28E-03 |
| C1S | -3.23E+00 | 1.25E-03 | 3.29E-03 |
| CRYGA | 3.23E+00 | 1.25E-03 | 3.29E-03 |
| C5 | -3.23E+00 | 1.26E-03 | 3.30E-03 |
| AP2S1 | 3.23E+00 | 1.26E-03 | 3.30E-03 |
| CD46 | -3.23E+00 | 1.26E-03 | 3.30E-03 |
| HTR3C | -3.23E+00 | 1.26E-03 | 3.30E-03 |
| UBOX5 | 3.23E+00 | 1.26E-03 | 3.30E-03 |
| MYST3 | 3.22E+00 | 1.26E-03 | 3.31E-03 |
| IL21 | 3.22E+00 | 1.27E-03 | 3.31E-03 |
| TICAM2 | -3.22E+00 | 1.27E-03 | 3.31E-03 |
| SLC2A9 | -3.22E+00 | 1.27E-03 | 3.32E-03 |
| ZNF23 | -3.22E+00 | 1.27E-03 | 3.32E-03 |
| MATN4 | 3.22E+00 | 1.27E-03 | 3.32E-03 |
| GPAT2 | 3.22E+00 | 1.27E-03 | 3.33E-03 |
| ZMYM2 | 3.22E+00 | 1.28E-03 | 3.34E-03 |
| KCNH3 | 3.22E+00 | 1.28E-03 | 3.34E-03 |
| C5orf22 | 3.22E+00 | 1.28E-03 | 3.35E-03 |
| AGAP4 | 3.22E+00 | 1.28E-03 | 3.35E-03 |
| CXorf23 | -3.22E+00 | 1.28E-03 | 3.36E-03 |
| TAS2R19 | 3.22E+00 | 1.29E-03 | 3.37E-03 |
| CNOT8 | -3.22E+00 | 1.29E-03 | 3.38E-03 |
| DCAF17 | 3.22E+00 | 1.29E-03 | 3.38E-03 |
| OLIG2 | 3.22E+00 | 1.29E-03 | 3.38E-03 |
| TTC18 | -3.22E+00 | 1.29E-03 | 3.38E-03 |

| EPB41L4B | -3.22E+00 | 1.29E-03 | 3.38E-03 |
| --- | --- | --- | --- |
| WBP2NL | -3.22E+00 | 1.30E-03 | 3.39E-03 |
| CRNKL1 | 3.22E+00 | 1.30E-03 | 3.41E-03 |
| CES8 | -3.21E+00 | 1.31E-03 | 3.41E-03 |
| CCNC | -3.21E+00 | 1.31E-03 | 3.42E-03 |
| TLR4 | -3.21E+00 | 1.31E-03 | 3.42E-03 |
| PMAIP1 | 3.21E+00 | 1.31E-03 | 3.43E-03 |
| CKMT1A | 3.21E+00 | 1.31E-03 | 3.43E-03 |
| TMEM219 | -3.21E+00 | 1.31E-03 | 3.43E-03 |
| MRPL46 | -3.21E+00 | 1.31E-03 | 3.43E-03 |
| DUSP3 | -3.21E+00 | 1.32E-03 | 3.44E-03 |
| GLT8D1 | -3.21E+00 | 1.32E-03 | 3.45E-03 |
| PTPN14 | 3.21E+00 | 1.32E-03 | 3.45E-03 |
| FBLIM1 | 3.21E+00 | 1.32E-03 | 3.45E-03 |
| ZNF22 | -3.21E+00 | 1.33E-03 | 3.46E-03 |
| TKTL1 | 3.21E+00 | 1.33E-03 | 3.47E-03 |
| C4orf31 | -3.21E+00 | 1.33E-03 | 3.48E-03 |
| CARD8 | -3.21E+00 | 1.33E-03 | 3.48E-03 |
| CDK5 | 3.21E+00 | 1.34E-03 | 3.49E-03 |
| MDH1 | -3.21E+00 | 1.34E-03 | 3.49E-03 |
| RBPJL | 3.21E+00 | 1.34E-03 | 3.49E-03 |
| NACA | 3.21E+00 | 1.34E-03 | 3.49E-03 |
| PRRG2 | -3.21E+00 | 1.34E-03 | 3.49E-03 |
| DLGAP2 | -3.21E+00 | 1.34E-03 | 3.50E-03 |
| VSIG10L | 3.21E+00 | 1.35E-03 | 3.52E-03 |
| PAF1 | 3.21E+00 | 1.35E-03 | 3.52E-03 |
| C3orf38 | -3.20E+00 | 1.36E-03 | 3.53E-03 |
| SMC6 | 3.20E+00 | 1.36E-03 | 3.53E-03 |
| RCHY1 | -3.20E+00 | 1.36E-03 | 3.53E-03 |
| IL11RA | -3.20E+00 | 1.37E-03 | 3.55E-03 |
| THBS4 | -3.20E+00 | 1.37E-03 | 3.56E-03 |
| CCDC130 | 3.20E+00 | 1.37E-03 | 3.56E-03 |
| CARD11 | 3.20E+00 | 1.37E-03 | 3.57E-03 |
| SCARA5 | -3.20E+00 | 1.37E-03 | 3.57E-03 |
| MAGED1 | 3.20E+00 | 1.37E-03 | 3.57E-03 |
| KCNAB3 | 3.20E+00 | 1.37E-03 | 3.57E-03 |
| VSTM2L | -3.20E+00 | 1.38E-03 | 3.58E-03 |
| VKORC1L1 | -3.20E+00 | 1.38E-03 | 3.59E-03 |
| HNRNPC | 3.20E+00 | 1.38E-03 | 3.59E-03 |
| HTR1B | 3.20E+00 | 1.38E-03 | 3.60E-03 |
| TWSG1 | -3.20E+00 | 1.38E-03 | 3.60E-03 |
| KRT36 | 3.20E+00 | 1.38E-03 | 3.60E-03 |
| TNNC2 | -3.20E+00 | 1.38E-03 | 3.60E-03 |
| ELMOD3 | -3.20E+00 | 1.39E-03 | 3.61E-03 |
| SP5 | 3.20E+00 | 1.39E-03 | 3.61E-03 |
| PHKA1 | 3.20E+00 | 1.39E-03 | 3.62E-03 |
| AK7 | -3.20E+00 | 1.39E-03 | 3.62E-03 |
| GLMN | 3.20E+00 | 1.39E-03 | 3.62E-03 |
| RAB24 | 3.20E+00 | 1.39E-03 | 3.62E-03 |
| SLC19A3 | -3.20E+00 | 1.40E-03 | 3.62E-03 |
| CXorf49B | 3.20E+00 | 1.40E-03 | 3.62E-03 |
| C15orf26 | -3.20E+00 | 1.40E-03 | 3.63E-03 |
| GPLD1 | -3.20E+00 | 1.40E-03 | 3.63E-03 |
| MAPK3 | 3.20E+00 | 1.40E-03 | 3.63E-03 |
| ZNF302 | -3.20E+00 | 1.40E-03 | 3.63E-03 |
| ACVRL1 | -3.20E+00 | 1.40E-03 | 3.63E-03 |
| TMEM62 | -3.19E+00 | 1.40E-03 | 3.64E-03 |

| ING4 | -3.19E+00 | 1.41E-03 | 3.65E-03 |
| --- | --- | --- | --- |
| ZNF16 | 3.19E+00 | 1.41E-03 | 3.65E-03 |
| SEC23IP | 3.19E+00 | 1.41E-03 | 3.65E-03 |
| CMA1 | -3.19E+00 | 1.41E-03 | 3.66E-03 |
| LEPROT | -3.19E+00 | 1.41E-03 | 3.66E-03 |
| SNX4 | -3.19E+00 | 1.41E-03 | 3.66E-03 |
| POF1B | 3.19E+00 | 1.41E-03 | 3.66E-03 |
| MAP2K3 | 3.19E+00 | 1.42E-03 | 3.68E-03 |
| MYL9 | -3.19E+00 | 1.42E-03 | 3.68E-03 |
| NCF1B | 3.19E+00 | 1.42E-03 | 3.68E-03 |
| PLSCR5 | 3.19E+00 | 1.42E-03 | 3.68E-03 |
| PCDHA3 | 3.19E+00 | 1.42E-03 | 3.68E-03 |
| LIN52 | -3.19E+00 | 1.42E-03 | 3.69E-03 |
| ZNF37B | 3.19E+00 | 1.42E-03 | 3.69E-03 |
| PPP3R1 | -3.19E+00 | 1.43E-03 | 3.69E-03 |
| ZNF720 | -3.19E+00 | 1.43E-03 | 3.69E-03 |
| FLJ35220 | -3.19E+00 | 1.43E-03 | 3.70E-03 |
| ST8SIA3 | 3.19E+00 | 1.43E-03 | 3.70E-03 |
| THEM5 | 3.19E+00 | 1.43E-03 | 3.70E-03 |
| LOC339047 | 3.19E+00 | 1.43E-03 | 3.70E-03 |
| ARHGAP20 | -3.19E+00 | 1.43E-03 | 3.70E-03 |
| PTBP2 | 3.19E+00 | 1.43E-03 | 3.71E-03 |
| FAM19A3 | 3.19E+00 | 1.44E-03 | 3.71E-03 |
| CXCR3 | 3.19E+00 | 1.44E-03 | 3.71E-03 |
| KIAA1109 | -3.19E+00 | 1.44E-03 | 3.71E-03 |
| ZNF462 | -3.19E+00 | 1.44E-03 | 3.73E-03 |
| DCPS | 3.19E+00 | 1.45E-03 | 3.74E-03 |
| KIF16B | -3.19E+00 | 1.45E-03 | 3.74E-03 |
| C1orf173 | -3.19E+00 | 1.45E-03 | 3.74E-03 |
| AKAP3 | -3.19E+00 | 1.45E-03 | 3.75E-03 |
| FAM13C | -3.18E+00 | 1.45E-03 | 3.75E-03 |
| GNAT1 | 3.18E+00 | 1.45E-03 | 3.76E-03 |
| TMEM203 | -3.18E+00 | 1.45E-03 | 3.76E-03 |
| HTR1A | 3.18E+00 | 1.46E-03 | 3.76E-03 |
| LONP2 | -3.18E+00 | 1.46E-03 | 3.76E-03 |
| SLC38A8 | 3.18E+00 | 1.46E-03 | 3.76E-03 |
| IGSF10 | -3.18E+00 | 1.46E-03 | 3.77E-03 |
| LOC727924 | 3.18E+00 | 1.46E-03 | 3.77E-03 |
| ZNF518A | 3.18E+00 | 1.46E-03 | 3.78E-03 |
| TBX1 | 3.18E+00 | 1.47E-03 | 3.78E-03 |
| C4orf49 | -3.18E+00 | 1.47E-03 | 3.79E-03 |
| TMEM209 | 3.18E+00 | 1.47E-03 | 3.79E-03 |
| CRIPAK | 3.18E+00 | 1.47E-03 | 3.79E-03 |
| POSTN | 3.18E+00 | 1.47E-03 | 3.80E-03 |
| C2orf52 | 3.18E+00 | 1.48E-03 | 3.81E-03 |
| DNAH3 | 3.18E+00 | 1.48E-03 | 3.81E-03 |
| IL1B | 3.18E+00 | 1.48E-03 | 3.82E-03 |
| SLC26A5 | -3.18E+00 | 1.48E-03 | 3.82E-03 |
| SIRPG | 3.18E+00 | 1.48E-03 | 3.82E-03 |
| FLJ42875 | -3.18E+00 | 1.48E-03 | 3.82E-03 |
| MAFA | 3.18E+00 | 1.49E-03 | 3.83E-03 |
| PLIN1 | -3.18E+00 | 1.49E-03 | 3.83E-03 |
| BAZ2B | -3.18E+00 | 1.49E-03 | 3.83E-03 |
| HEMK1 | -3.18E+00 | 1.49E-03 | 3.83E-03 |
| OR56A4 | 3.18E+00 | 1.49E-03 | 3.83E-03 |
| ODF3L2 | 3.18E+00 | 1.49E-03 | 3.84E-03 |
| MAP1LC3C | -3.18E+00 | 1.49E-03 | 3.84E-03 |

| SIL1 | -3.18E+00 | 1.49E-03 | 3.85E-03 |
| --- | --- | --- | --- |
| LRTM2 | 3.18E+00 | 1.50E-03 | 3.86E-03 |
| PGAM2 | 3.18E+00 | 1.50E-03 | 3.86E-03 |
| TBX18 | 3.18E+00 | 1.50E-03 | 3.86E-03 |
| PEX16 | 3.17E+00 | 1.50E-03 | 3.86E-03 |
| CAMLG | -3.17E+00 | 1.50E-03 | 3.87E-03 |
| CYP1A2 | -3.17E+00 | 1.51E-03 | 3.87E-03 |
| GPR123 | -3.17E+00 | 1.51E-03 | 3.87E-03 |
| SCNN1A | -3.17E+00 | 1.51E-03 | 3.87E-03 |
| IGLL3 | 3.17E+00 | 1.51E-03 | 3.88E-03 |
| FCHSD2 | -3.17E+00 | 1.51E-03 | 3.88E-03 |
| CSDA | 3.17E+00 | 1.51E-03 | 3.88E-03 |
| BPHL | -3.17E+00 | 1.51E-03 | 3.89E-03 |
| ZBTB3 | -3.17E+00 | 1.52E-03 | 3.89E-03 |
| C10orf78 | 3.17E+00 | 1.52E-03 | 3.90E-03 |
| PROSC | -3.17E+00 | 1.52E-03 | 3.90E-03 |
| RHOXF1 | -3.17E+00 | 1.52E-03 | 3.91E-03 |
| AHNAK | -3.17E+00 | 1.53E-03 | 3.92E-03 |
| RABL3 | -3.17E+00 | 1.53E-03 | 3.92E-03 |
| C5orf43 | -3.17E+00 | 1.53E-03 | 3.93E-03 |
| AGAP8 | 3.17E+00 | 1.53E-03 | 3.93E-03 |
| AKAP7 | -3.17E+00 | 1.54E-03 | 3.95E-03 |
| SERF2 | -3.17E+00 | 1.54E-03 | 3.96E-03 |
| EML3 | 3.17E+00 | 1.55E-03 | 3.97E-03 |
| PHKA2 | 3.17E+00 | 1.55E-03 | 3.97E-03 |
| TMEM99 | -3.17E+00 | 1.55E-03 | 3.97E-03 |
| ALKBH5 | 3.17E+00 | 1.55E-03 | 3.97E-03 |
| PRKY | 3.17E+00 | 1.55E-03 | 3.98E-03 |
| LOC285205 | 3.17E+00 | 1.55E-03 | 3.98E-03 |
| C9orf139 | 3.16E+00 | 1.56E-03 | 3.99E-03 |
| TPK1 | -3.16E+00 | 1.56E-03 | 3.99E-03 |
| RASA3 | 3.16E+00 | 1.56E-03 | 3.99E-03 |
| PIK3IP1 | -3.16E+00 | 1.56E-03 | 4.01E-03 |
| C20orf30 | -3.16E+00 | 1.56E-03 | 4.01E-03 |
| LRRC37A2 | 3.16E+00 | 1.57E-03 | 4.01E-03 |
| PYCR2 | 3.16E+00 | 1.57E-03 | 4.02E-03 |
| DMXL2 | 3.16E+00 | 1.57E-03 | 4.02E-03 |
| IL12RB1 | 3.16E+00 | 1.57E-03 | 4.02E-03 |
| PGF | 3.16E+00 | 1.57E-03 | 4.02E-03 |
| PMVK | -3.16E+00 | 1.57E-03 | 4.02E-03 |
| ANXA13 | 3.16E+00 | 1.57E-03 | 4.02E-03 |
| PODXL2 | 3.16E+00 | 1.57E-03 | 4.03E-03 |
| C11orf71 | -3.16E+00 | 1.58E-03 | 4.03E-03 |
| CST3 | -3.16E+00 | 1.58E-03 | 4.03E-03 |
| HPGD | -3.16E+00 | 1.58E-03 | 4.03E-03 |
| CCNI2 | 3.16E+00 | 1.58E-03 | 4.04E-03 |
| ARFGEF1 | 3.16E+00 | 1.58E-03 | 4.05E-03 |
| LUM | -3.16E+00 | 1.58E-03 | 4.05E-03 |
| BCS1L | 3.16E+00 | 1.58E-03 | 4.05E-03 |
| HDAC11 | 3.16E+00 | 1.58E-03 | 4.05E-03 |
| C18orf56 | 3.16E+00 | 1.59E-03 | 4.06E-03 |
| VTA1 | 3.16E+00 | 1.59E-03 | 4.07E-03 |
| FNIP1 | -3.16E+00 | 1.59E-03 | 4.08E-03 |
| TEPP | -3.16E+00 | 1.60E-03 | 4.08E-03 |
| BMP5 | -3.16E+00 | 1.60E-03 | 4.09E-03 |
| LGI2 | 3.16E+00 | 1.60E-03 | 4.10E-03 |
| IRF8 | -3.16E+00 | 1.61E-03 | 4.10E-03 |

| C16orf90 | 3.15E+00 | 1.62E-03 | 4.12E-03 |
| --- | --- | --- | --- |
| GABRG3 | 3.15E+00 | 1.61E-03 | 4.12E-03 |
| ZBTB6 | -3.15E+00 | 1.62E-03 | 4.12E-03 |
| PPIH | 3.15E+00 | 1.62E-03 | 4.13E-03 |
| ME1 | 3.15E+00 | 1.62E-03 | 4.13E-03 |
| MYNN | -3.15E+00 | 1.62E-03 | 4.13E-03 |
| TGM3 | 3.15E+00 | 1.62E-03 | 4.13E-03 |
| TPSB2 | -3.15E+00 | 1.62E-03 | 4.13E-03 |
| PEBP1 | -3.15E+00 | 1.62E-03 | 4.13E-03 |
| PIGT | 3.15E+00 | 1.62E-03 | 4.14E-03 |
| GUCY1A3 | -3.15E+00 | 1.62E-03 | 4.14E-03 |
| CAV1 | -3.15E+00 | 1.62E-03 | 4.14E-03 |
| CDH5 | -3.15E+00 | 1.63E-03 | 4.15E-03 |
| GPR142 | 3.15E+00 | 1.63E-03 | 4.16E-03 |
| C20orf111 | -3.15E+00 | 1.63E-03 | 4.16E-03 |
| CRELD1 | -3.15E+00 | 1.63E-03 | 4.17E-03 |
| AQP3 | -3.15E+00 | 1.64E-03 | 4.18E-03 |
| TADA2B | -3.15E+00 | 1.64E-03 | 4.18E-03 |
| EYS | -3.15E+00 | 1.64E-03 | 4.18E-03 |
| LEO1 | 3.15E+00 | 1.64E-03 | 4.18E-03 |
| KRT17 | 3.15E+00 | 1.64E-03 | 4.18E-03 |
| COX7C | -3.15E+00 | 1.64E-03 | 4.18E-03 |
| ARL17A | 3.15E+00 | 1.64E-03 | 4.19E-03 |
| SCARNA16 | 3.15E+00 | 1.64E-03 | 4.19E-03 |
| RWDD3 | -3.15E+00 | 1.64E-03 | 4.19E-03 |
| C4orf41 | -3.15E+00 | 1.65E-03 | 4.19E-03 |
| PTCH1 | -3.15E+00 | 1.65E-03 | 4.20E-03 |
| FNBP4 | 3.15E+00 | 1.65E-03 | 4.20E-03 |
| NUCKS1 | -3.15E+00 | 1.66E-03 | 4.21E-03 |
| ETS2 | -3.15E+00 | 1.66E-03 | 4.22E-03 |
| HSCB | -3.15E+00 | 1.66E-03 | 4.22E-03 |
| NXF3 | -3.14E+00 | 1.67E-03 | 4.24E-03 |
| OCLN | -3.14E+00 | 1.67E-03 | 4.24E-03 |
| CDKL4 | 3.14E+00 | 1.67E-03 | 4.25E-03 |
| PSG3 | 3.14E+00 | 1.67E-03 | 4.25E-03 |
| SLC22A14 | 3.14E+00 | 1.67E-03 | 4.25E-03 |
| KCNH6 | 3.14E+00 | 1.67E-03 | 4.25E-03 |
| MTAP | 3.14E+00 | 1.68E-03 | 4.27E-03 |
| LOC100271831 | 3.14E+00 | 1.68E-03 | 4.27E-03 |
| AKNAD1 | 3.14E+00 | 1.68E-03 | 4.27E-03 |
| PAEP | 3.14E+00 | 1.68E-03 | 4.27E-03 |
| GPR108 | -3.14E+00 | 1.68E-03 | 4.28E-03 |
| C15orf34 | -3.14E+00 | 1.68E-03 | 4.28E-03 |
| C1orf152 | -3.14E+00 | 1.69E-03 | 4.29E-03 |
| C20orf70 | 3.14E+00 | 1.69E-03 | 4.29E-03 |
| AGK | 3.14E+00 | 1.69E-03 | 4.29E-03 |
| DEAF1 | 3.14E+00 | 1.69E-03 | 4.30E-03 |
| SFMBT2 | 3.14E+00 | 1.69E-03 | 4.30E-03 |
| TMEM37 | -3.14E+00 | 1.69E-03 | 4.30E-03 |
| PDE7A | 3.14E+00 | 1.69E-03 | 4.30E-03 |
| PDE1A | -3.14E+00 | 1.70E-03 | 4.31E-03 |
| ZNF397OS | 3.14E+00 | 1.70E-03 | 4.32E-03 |
| SERP1 | -3.14E+00 | 1.70E-03 | 4.32E-03 |
| USP32 | 3.14E+00 | 1.71E-03 | 4.33E-03 |
| NDRG4 | 3.14E+00 | 1.71E-03 | 4.33E-03 |
| PARK7 | -3.14E+00 | 1.71E-03 | 4.34E-03 |
| ALDH1A1 | -3.14E+00 | 1.71E-03 | 4.35E-03 |

| MSTO2P | 3.14E+00 | 1.72E-03 | 4.36E-03 |
| --- | --- | --- | --- |
| KIAA0649 | 3.14E+00 | 1.72E-03 | 4.36E-03 |
| NIPAL4 | 3.14E+00 | 1.72E-03 | 4.36E-03 |
| FAM76B | -3.13E+00 | 1.72E-03 | 4.37E-03 |
| HHLA3 | -3.13E+00 | 1.73E-03 | 4.38E-03 |
| WDR55 | 3.13E+00 | 1.73E-03 | 4.39E-03 |
| PMS2L2 | -3.13E+00 | 1.73E-03 | 4.39E-03 |
| PRDX6 | -3.13E+00 | 1.73E-03 | 4.39E-03 |
| PRPH2 | 3.13E+00 | 1.73E-03 | 4.39E-03 |
| ANKHD1 | -3.13E+00 | 1.74E-03 | 4.40E-03 |
| C1orf96 | 3.13E+00 | 1.74E-03 | 4.40E-03 |
| GSTM1 | -3.13E+00 | 1.74E-03 | 4.40E-03 |
| DACT3 | -3.13E+00 | 1.74E-03 | 4.41E-03 |
| LOC100127888 | 3.13E+00 | 1.74E-03 | 4.41E-03 |
| NCOA7 | -3.13E+00 | 1.74E-03 | 4.41E-03 |
| IMMP2L | -3.13E+00 | 1.75E-03 | 4.42E-03 |
| RAPGEF3 | -3.13E+00 | 1.75E-03 | 4.42E-03 |
| SRRM3 | 3.13E+00 | 1.75E-03 | 4.42E-03 |
| PLA2G4C | -3.13E+00 | 1.75E-03 | 4.43E-03 |
| LGI3 | -3.13E+00 | 1.75E-03 | 4.43E-03 |
| INSRR | 3.13E+00 | 1.75E-03 | 4.44E-03 |
| C16orf53 | 3.13E+00 | 1.76E-03 | 4.44E-03 |
| PAIP2B | -3.13E+00 | 1.76E-03 | 4.44E-03 |
| PCDHB5 | 3.13E+00 | 1.76E-03 | 4.45E-03 |
| LOC728276 | -3.13E+00 | 1.76E-03 | 4.45E-03 |
| DRD2 | 3.13E+00 | 1.76E-03 | 4.45E-03 |
| GPR161 | 3.13E+00 | 1.77E-03 | 4.47E-03 |
| ARRDC2 | -3.13E+00 | 1.77E-03 | 4.47E-03 |
| NPR3 | -3.13E+00 | 1.77E-03 | 4.47E-03 |
| AS3MT | -3.13E+00 | 1.77E-03 | 4.47E-03 |
| KIAA0913 | 3.13E+00 | 1.77E-03 | 4.48E-03 |
| PHF8 | 3.13E+00 | 1.77E-03 | 4.48E-03 |
| FAM123B | 3.13E+00 | 1.78E-03 | 4.48E-03 |
| VASH2 | 3.13E+00 | 1.78E-03 | 4.49E-03 |
| NMB | 3.13E+00 | 1.78E-03 | 4.49E-03 |
| HBG1 | -3.13E+00 | 1.78E-03 | 4.49E-03 |
| SPDYE7P | 3.13E+00 | 1.78E-03 | 4.49E-03 |
| LRRC25 | 3.12E+00 | 1.78E-03 | 4.50E-03 |
| MR1 | -3.12E+00 | 1.79E-03 | 4.52E-03 |
| ADAMTS2 | 3.12E+00 | 1.79E-03 | 4.52E-03 |
| TMEM25 | -3.12E+00 | 1.79E-03 | 4.52E-03 |
| USP25 | -3.12E+00 | 1.79E-03 | 4.52E-03 |
| ZNF502 | -3.12E+00 | 1.79E-03 | 4.52E-03 |
| ALX4 | 3.12E+00 | 1.79E-03 | 4.52E-03 |
| CTSZ | -3.12E+00 | 1.79E-03 | 4.52E-03 |
| DISP2 | 3.12E+00 | 1.79E-03 | 4.52E-03 |
| GABRE | -3.12E+00 | 1.79E-03 | 4.52E-03 |
| SHANK3 | 3.12E+00 | 1.79E-03 | 4.52E-03 |
| IGFBP3 | 3.12E+00 | 1.80E-03 | 4.53E-03 |
| SPNS2 | -3.12E+00 | 1.80E-03 | 4.53E-03 |
| GTF2I | 3.12E+00 | 1.80E-03 | 4.54E-03 |
| TSPY1 | 3.12E+00 | 1.81E-03 | 4.55E-03 |
| OPA1 | 3.12E+00 | 1.81E-03 | 4.55E-03 |
| ARNTL | -3.12E+00 | 1.81E-03 | 4.55E-03 |
| ZNF609 | 3.12E+00 | 1.81E-03 | 4.55E-03 |
| IKZF4 | 3.12E+00 | 1.81E-03 | 4.56E-03 |
| LOC100188949 | -3.12E+00 | 1.81E-03 | 4.56E-03 |

| PIGR | -3.12E+00 | 1.82E-03 | 4.58E-03 |
| --- | --- | --- | --- |
| AGPAT4 | 3.12E+00 | 1.82E-03 | 4.59E-03 |
| FAM184A | -3.12E+00 | 1.82E-03 | 4.59E-03 |
| ZADH2 | -3.12E+00 | 1.83E-03 | 4.59E-03 |
| OSTC | -3.12E+00 | 1.83E-03 | 4.60E-03 |
| A1BG | -3.12E+00 | 1.83E-03 | 4.60E-03 |
| LOC647309 | -3.12E+00 | 1.83E-03 | 4.61E-03 |
| THRAP3 | 3.12E+00 | 1.83E-03 | 4.61E-03 |
| C10orf129 | 3.12E+00 | 1.84E-03 | 4.62E-03 |
| UHRF2 | 3.12E+00 | 1.84E-03 | 4.62E-03 |
| AVPI1 | -3.12E+00 | 1.84E-03 | 4.62E-03 |
| EGLN1 | -3.12E+00 | 1.84E-03 | 4.62E-03 |
| SMAD4 | -3.12E+00 | 1.84E-03 | 4.62E-03 |
| LOC100134259 | -3.12E+00 | 1.84E-03 | 4.63E-03 |
| C4orf45 | -3.12E+00 | 1.84E-03 | 4.63E-03 |
| CTNNA3 | -3.12E+00 | 1.84E-03 | 4.63E-03 |
| KPNA4 | 3.12E+00 | 1.84E-03 | 4.63E-03 |
| WDR53 | 3.11E+00 | 1.85E-03 | 4.65E-03 |
| OR52L1 | 3.11E+00 | 1.85E-03 | 4.65E-03 |
| DIAPH2 | -3.11E+00 | 1.85E-03 | 4.66E-03 |
| NRIP2 | -3.11E+00 | 1.85E-03 | 4.66E-03 |
| ENDOD1 | -3.11E+00 | 1.86E-03 | 4.66E-03 |
| DNASE2 | -3.11E+00 | 1.86E-03 | 4.67E-03 |
| ACTR8 | 3.11E+00 | 1.86E-03 | 4.67E-03 |
| ARVCF | 3.11E+00 | 1.87E-03 | 4.68E-03 |
| NPIP | 3.11E+00 | 1.87E-03 | 4.68E-03 |
| TSC1 | 3.11E+00 | 1.87E-03 | 4.68E-03 |
| LRRC4 | -3.11E+00 | 1.87E-03 | 4.68E-03 |
| GAL3ST4 | 3.11E+00 | 1.87E-03 | 4.69E-03 |
| LRRC17 | -3.11E+00 | 1.87E-03 | 4.69E-03 |
| CTH | -3.11E+00 | 1.87E-03 | 4.69E-03 |
| KIAA0368 | 3.11E+00 | 1.87E-03 | 4.69E-03 |
| GPRASP1 | -3.11E+00 | 1.87E-03 | 4.70E-03 |
| KLF7 | 3.11E+00 | 1.88E-03 | 4.70E-03 |
| SERPING1 | -3.11E+00 | 1.88E-03 | 4.71E-03 |
| LOC641298 | 3.11E+00 | 1.88E-03 | 4.71E-03 |
| CBR1 | -3.11E+00 | 1.88E-03 | 4.72E-03 |
| SUFU | 3.11E+00 | 1.89E-03 | 4.72E-03 |
| SNORA39 | 3.11E+00 | 1.89E-03 | 4.72E-03 |
| IL1RL2 | 3.11E+00 | 1.89E-03 | 4.73E-03 |
| MCCC1 | -3.11E+00 | 1.89E-03 | 4.73E-03 |
| MAGEB10 | 3.11E+00 | 1.89E-03 | 4.73E-03 |
| AVPR1B | 3.11E+00 | 1.89E-03 | 4.73E-03 |
| FYN | -3.11E+00 | 1.89E-03 | 4.73E-03 |
| ITPKA | 3.11E+00 | 1.89E-03 | 4.73E-03 |
| GGTA1 | -3.11E+00 | 1.89E-03 | 4.74E-03 |
| SLC9A7 | 3.11E+00 | 1.89E-03 | 4.74E-03 |
| MYH2 | -3.11E+00 | 1.90E-03 | 4.74E-03 |
| SOX9 | 3.11E+00 | 1.90E-03 | 4.74E-03 |
| DERL2 | -3.11E+00 | 1.90E-03 | 4.74E-03 |
| HIST1H2BB | 3.11E+00 | 1.90E-03 | 4.75E-03 |
| CYSLTR1 | -3.11E+00 | 1.90E-03 | 4.76E-03 |
| PHF11 | -3.11E+00 | 1.91E-03 | 4.76E-03 |
| EPX | -3.10E+00 | 1.91E-03 | 4.77E-03 |
| FLJ30679 | 3.10E+00 | 1.91E-03 | 4.77E-03 |
| PIGN | -3.10E+00 | 1.91E-03 | 4.77E-03 |
| TMEM147 | 3.10E+00 | 1.91E-03 | 4.77E-03 |

| LOC389634 | 3.10E+00 | 1.91E-03 | 4.78E-03 |
| --- | --- | --- | --- |
| MOGAT2 | -3.10E+00 | 1.91E-03 | 4.78E-03 |
| LRP6 | -3.10E+00 | 1.91E-03 | 4.78E-03 |
| DIS3 | -3.10E+00 | 1.92E-03 | 4.79E-03 |
| TAZ | 3.10E+00 | 1.92E-03 | 4.79E-03 |
| PPIL5 | 3.10E+00 | 1.92E-03 | 4.79E-03 |
| TMEM132C | -3.10E+00 | 1.92E-03 | 4.79E-03 |
| ZNF525 | 3.10E+00 | 1.92E-03 | 4.80E-03 |
| CWC22 | 3.10E+00 | 1.92E-03 | 4.80E-03 |
| TERF1 | 3.10E+00 | 1.92E-03 | 4.80E-03 |
| C1orf105 | 3.10E+00 | 1.92E-03 | 4.80E-03 |
| CDH12 | 3.10E+00 | 1.92E-03 | 4.80E-03 |
| FAM114A2 | -3.10E+00 | 1.93E-03 | 4.81E-03 |
| OR5AK2 | -3.10E+00 | 1.93E-03 | 4.81E-03 |
| TMEM214 | 3.10E+00 | 1.93E-03 | 4.81E-03 |
| SPCS2 | -3.10E+00 | 1.93E-03 | 4.82E-03 |
| IL18BP | 3.10E+00 | 1.93E-03 | 4.82E-03 |
| RNF219 | 3.10E+00 | 1.93E-03 | 4.82E-03 |
| CHMP5 | -3.10E+00 | 1.93E-03 | 4.82E-03 |
| ZNF616 | -3.10E+00 | 1.94E-03 | 4.83E-03 |
| ZNF554 | -3.10E+00 | 1.94E-03 | 4.83E-03 |
| SPINK5 | -3.10E+00 | 1.95E-03 | 4.85E-03 |
| C4orf6 | 3.10E+00 | 1.95E-03 | 4.85E-03 |
| SYCP2L | 3.10E+00 | 1.95E-03 | 4.87E-03 |
| AKR1C4 | 3.10E+00 | 1.96E-03 | 4.88E-03 |
| ACTN2 | -3.10E+00 | 1.96E-03 | 4.88E-03 |
| PRG4 | -3.10E+00 | 1.96E-03 | 4.88E-03 |
| ACYP1 | 3.10E+00 | 1.97E-03 | 4.89E-03 |
| MAGEB16 | 3.10E+00 | 1.97E-03 | 4.90E-03 |
| EIF5A2 | 3.10E+00 | 1.97E-03 | 4.90E-03 |
| ELFN1 | 3.10E+00 | 1.97E-03 | 4.90E-03 |
| DKK2 | -3.10E+00 | 1.97E-03 | 4.91E-03 |
| ZNF676 | 3.09E+00 | 1.98E-03 | 4.92E-03 |
| CMTM4 | 3.09E+00 | 1.98E-03 | 4.92E-03 |
| LOC440896 | 3.09E+00 | 1.98E-03 | 4.92E-03 |
| HAGHL | 3.09E+00 | 1.98E-03 | 4.93E-03 |
| HAR1A | -3.09E+00 | 1.98E-03 | 4.93E-03 |
| TCTE1 | -3.09E+00 | 1.99E-03 | 4.94E-03 |
| PLK2 | -3.09E+00 | 1.99E-03 | 4.94E-03 |
| KANK1 | -3.09E+00 | 1.99E-03 | 4.94E-03 |
| ODC1 | 3.09E+00 | 1.99E-03 | 4.95E-03 |
| SMARCD2 | 3.09E+00 | 1.99E-03 | 4.95E-03 |
| NFATC4 | 3.09E+00 | 2.00E-03 | 4.96E-03 |
| APOD | -3.09E+00 | 2.00E-03 | 4.96E-03 |
| KIAA1908 | -3.09E+00 | 2.00E-03 | 4.96E-03 |
| CTGF | -3.09E+00 | 2.00E-03 | 4.97E-03 |
| LIMS3 | 3.09E+00 | 2.00E-03 | 4.98E-03 |
| FAM129B | 3.09E+00 | 2.01E-03 | 4.99E-03 |
| TNFAIP6 | 3.09E+00 | 2.01E-03 | 4.99E-03 |
| TLL1 | -3.09E+00 | 2.01E-03 | 4.99E-03 |
| TPI1P3 | 3.09E+00 | 2.01E-03 | 4.99E-03 |
| C1orf126 | 3.09E+00 | 2.02E-03 | 5.00E-03 |
| ABI3BP | -3.09E+00 | 2.02E-03 | 5.01E-03 |
| LTBP2 | -3.09E+00 | 2.02E-03 | 5.01E-03 |
| RASD1 | -3.09E+00 | 2.02E-03 | 5.01E-03 |
| MYLK3 | 3.09E+00 | 2.03E-03 | 5.03E-03 |
| ANXA4 | -3.09E+00 | 2.03E-03 | 5.03E-03 |

| SIM1 | 3.09E+00 | 2.03E-03 | 5.04E-03 |
| --- | --- | --- | --- |
| ZNF383 | -3.09E+00 | 2.03E-03 | 5.04E-03 |
| HIPK4 | 3.09E+00 | 2.03E-03 | 5.04E-03 |
| NEK7 | -3.09E+00 | 2.04E-03 | 5.05E-03 |
| USO1 | -3.09E+00 | 2.04E-03 | 5.05E-03 |
| MEIS3 | 3.09E+00 | 2.04E-03 | 5.05E-03 |
| CCDC73 | -3.08E+00 | 2.04E-03 | 5.06E-03 |
| PPIC | -3.08E+00 | 2.04E-03 | 5.06E-03 |
| HTR3E | 3.08E+00 | 2.05E-03 | 5.08E-03 |
| SPANXN4 | 3.08E+00 | 2.05E-03 | 5.09E-03 |
| GSTA2 | -3.08E+00 | 2.05E-03 | 5.09E-03 |
| HIST1H2BJ | 3.08E+00 | 2.05E-03 | 5.09E-03 |
| CLDN16 | -3.08E+00 | 2.06E-03 | 5.09E-03 |
| PATZ1 | 3.08E+00 | 2.06E-03 | 5.09E-03 |
| LEPREL1 | -3.08E+00 | 2.06E-03 | 5.09E-03 |
| PIGH | -3.08E+00 | 2.06E-03 | 5.11E-03 |
| GRM6 | -3.08E+00 | 2.06E-03 | 5.11E-03 |
| ITM2C | 3.08E+00 | 2.06E-03 | 5.11E-03 |
| SCN8A | 3.08E+00 | 2.07E-03 | 5.11E-03 |
| GPR50 | 3.08E+00 | 2.07E-03 | 5.12E-03 |
| C6orf126 | 3.08E+00 | 2.08E-03 | 5.14E-03 |
| FOXL2 | 3.08E+00 | 2.08E-03 | 5.15E-03 |
| MRPL15 | 3.08E+00 | 2.08E-03 | 5.15E-03 |
| TMEM123 | -3.08E+00 | 2.08E-03 | 5.15E-03 |
| ASPRV1 | -3.08E+00 | 2.09E-03 | 5.16E-03 |
| FOXP1 | -3.08E+00 | 2.09E-03 | 5.17E-03 |
| THAP8 | 3.08E+00 | 2.09E-03 | 5.17E-03 |
| HRSP12 | -3.08E+00 | 2.09E-03 | 5.18E-03 |
| SNORD116-20 | 3.08E+00 | 2.10E-03 | 5.18E-03 |
| SCNN1B | -3.08E+00 | 2.10E-03 | 5.20E-03 |
| C17orf46 | 3.08E+00 | 2.11E-03 | 5.20E-03 |
| C1orf129 | -3.07E+00 | 2.11E-03 | 5.21E-03 |
| DPEP1 | 3.07E+00 | 2.11E-03 | 5.22E-03 |
| FRMD1 | 3.07E+00 | 2.11E-03 | 5.22E-03 |
| PIPSL | 3.07E+00 | 2.11E-03 | 5.22E-03 |
| SLC9A3R2 | -3.07E+00 | 2.11E-03 | 5.22E-03 |
| TMEM187 | -3.07E+00 | 2.12E-03 | 5.23E-03 |
| LOC440944 | 3.07E+00 | 2.13E-03 | 5.26E-03 |
| NWD1 | -3.07E+00 | 2.13E-03 | 5.26E-03 |
| FAM63A | -3.07E+00 | 2.13E-03 | 5.26E-03 |
| KPNA6 | 3.07E+00 | 2.14E-03 | 5.28E-03 |
| PIBF1 | -3.07E+00 | 2.14E-03 | 5.28E-03 |
| CTSA | 3.07E+00 | 2.14E-03 | 5.29E-03 |
| FAM161B | -3.07E+00 | 2.15E-03 | 5.30E-03 |
| SCMH1 | 3.07E+00 | 2.15E-03 | 5.30E-03 |
| TMEM11 | 3.07E+00 | 2.15E-03 | 5.30E-03 |
| TAB2 | -3.07E+00 | 2.15E-03 | 5.30E-03 |
| APLF | -3.07E+00 | 2.15E-03 | 5.30E-03 |
| ROBO3 | 3.07E+00 | 2.15E-03 | 5.31E-03 |
| TBX19 | 3.07E+00 | 2.15E-03 | 5.31E-03 |
| LOC340357 | 3.07E+00 | 2.16E-03 | 5.31E-03 |
| NT5C3L | 3.07E+00 | 2.16E-03 | 5.31E-03 |
| VWCE | 3.07E+00 | 2.16E-03 | 5.32E-03 |
| C16orf80 | -3.07E+00 | 2.17E-03 | 5.34E-03 |
| THTPA | -3.07E+00 | 2.17E-03 | 5.34E-03 |
| SGCA | -3.07E+00 | 2.17E-03 | 5.34E-03 |
| PFDN6 | 3.07E+00 | 2.17E-03 | 5.35E-03 |

| PAFAH1B2 | 3.07E+00 | 2.18E-03 | 5.36E-03 |
| --- | --- | --- | --- |
| CAPN5 | -3.07E+00 | 2.18E-03 | 5.36E-03 |
| TLR5 | -3.07E+00 | 2.18E-03 | 5.36E-03 |
| RBM25 | 3.06E+00 | 2.18E-03 | 5.37E-03 |
| BBS2 | -3.06E+00 | 2.18E-03 | 5.38E-03 |
| EIF4B | 3.06E+00 | 2.18E-03 | 5.38E-03 |
| RUFY4 | 3.06E+00 | 2.19E-03 | 5.38E-03 |
| CHRNA1 | 3.06E+00 | 2.19E-03 | 5.38E-03 |
| TMEM178 | -3.06E+00 | 2.19E-03 | 5.38E-03 |
| DNAJC30 | -3.06E+00 | 2.19E-03 | 5.38E-03 |
| IL1A | 3.06E+00 | 2.19E-03 | 5.38E-03 |
| NCOR1 | 3.06E+00 | 2.19E-03 | 5.39E-03 |
| IL17RE | -3.06E+00 | 2.20E-03 | 5.40E-03 |
| TIMM16 | 3.06E+00 | 2.20E-03 | 5.40E-03 |
| MAGEB3 | 3.06E+00 | 2.20E-03 | 5.40E-03 |
| FAM120C | -3.06E+00 | 2.20E-03 | 5.41E-03 |
| LOC285401 | 3.06E+00 | 2.20E-03 | 5.41E-03 |
| HMOX1 | 3.06E+00 | 2.20E-03 | 5.42E-03 |
| TTLL10 | -3.06E+00 | 2.21E-03 | 5.43E-03 |
| HNRNPK | 3.06E+00 | 2.21E-03 | 5.44E-03 |
| LIMCH1 | -3.06E+00 | 2.22E-03 | 5.44E-03 |
| CAPN1 | 3.06E+00 | 2.22E-03 | 5.46E-03 |
| AWAT1 | 3.06E+00 | 2.23E-03 | 5.48E-03 |
| HAUS4 | -3.06E+00 | 2.25E-03 | 5.51E-03 |
| ZNF606 | -3.06E+00 | 2.25E-03 | 5.51E-03 |
| FLJ13224 | 3.06E+00 | 2.25E-03 | 5.52E-03 |
| PMP22 | -3.06E+00 | 2.25E-03 | 5.52E-03 |
| C16orf45 | -3.06E+00 | 2.25E-03 | 5.52E-03 |
| CXorf36 | -3.06E+00 | 2.25E-03 | 5.53E-03 |
| ZFYVE26 | 3.06E+00 | 2.25E-03 | 5.53E-03 |
| SDHAP1 | 3.05E+00 | 2.26E-03 | 5.54E-03 |
| PRKAR1A | -3.05E+00 | 2.26E-03 | 5.54E-03 |
| CHRM5 | 3.05E+00 | 2.26E-03 | 5.54E-03 |
| TEAD3 | 3.05E+00 | 2.26E-03 | 5.55E-03 |
| BANF2 | 3.05E+00 | 2.26E-03 | 5.55E-03 |
| ZNF501 | -3.05E+00 | 2.26E-03 | 5.55E-03 |
| TRIM67 | 3.05E+00 | 2.27E-03 | 5.56E-03 |
| ZNF721 | -3.05E+00 | 2.28E-03 | 5.59E-03 |
| GPN1 | 3.05E+00 | 2.28E-03 | 5.60E-03 |
| ETV6 | 3.05E+00 | 2.29E-03 | 5.62E-03 |
| CCL15 | -3.05E+00 | 2.30E-03 | 5.63E-03 |
| TEX11 | 3.05E+00 | 2.30E-03 | 5.63E-03 |
| ZNF673 | -3.05E+00 | 2.30E-03 | 5.63E-03 |
| ACBD4 | -3.05E+00 | 2.30E-03 | 5.63E-03 |
| SPI1 | 3.05E+00 | 2.30E-03 | 5.64E-03 |
| GFM1 | 3.05E+00 | 2.30E-03 | 5.64E-03 |
| C17orf73 | 3.05E+00 | 2.30E-03 | 5.64E-03 |
| DLEU7 | 3.05E+00 | 2.30E-03 | 5.64E-03 |
| UBL5 | -3.05E+00 | 2.31E-03 | 5.66E-03 |
| DDX24 | -3.05E+00 | 2.32E-03 | 5.67E-03 |
| C9orf167 | 3.05E+00 | 2.32E-03 | 5.68E-03 |
| GSTM4 | -3.05E+00 | 2.32E-03 | 5.68E-03 |
| CYTH4 | 3.04E+00 | 2.33E-03 | 5.71E-03 |
| TTTY15 | 3.04E+00 | 2.33E-03 | 5.71E-03 |
| ANXA1 | -3.04E+00 | 2.34E-03 | 5.72E-03 |
| ARHGAP1 | 3.04E+00 | 2.34E-03 | 5.72E-03 |
| HIGD1B | -3.04E+00 | 2.34E-03 | 5.73E-03 |

| TAGLN | -3.04E+00 | 2.35E-03 | 5.74E-03 |
| --- | --- | --- | --- |
| KCNIP1 | -3.04E+00 | 2.35E-03 | 5.75E-03 |
| CDKAL1 | 3.04E+00 | 2.36E-03 | 5.77E-03 |
| LRP2BP | -3.04E+00 | 2.36E-03 | 5.77E-03 |
| PTPRR | -3.04E+00 | 2.37E-03 | 5.79E-03 |
| SYT2 | -3.04E+00 | 2.37E-03 | 5.79E-03 |
| SPG20 | -3.04E+00 | 2.37E-03 | 5.79E-03 |
| C1orf210 | -3.04E+00 | 2.37E-03 | 5.79E-03 |
| OR51Q1 | 3.04E+00 | 2.37E-03 | 5.80E-03 |
| LOC647979 | -3.04E+00 | 2.38E-03 | 5.81E-03 |
| CLDN9 | -3.04E+00 | 2.38E-03 | 5.81E-03 |
| SKP1 | -3.04E+00 | 2.38E-03 | 5.82E-03 |
| MAS1L | -3.04E+00 | 2.38E-03 | 5.82E-03 |
| ERGIC2 | 3.04E+00 | 2.38E-03 | 5.82E-03 |
| LMBR1L | 3.04E+00 | 2.39E-03 | 5.83E-03 |
| EBF3 | -3.04E+00 | 2.39E-03 | 5.83E-03 |
| FLJ42709 | -3.04E+00 | 2.39E-03 | 5.83E-03 |
| GSDMC | 3.04E+00 | 2.39E-03 | 5.83E-03 |
| NID1 | 3.04E+00 | 2.39E-03 | 5.84E-03 |
| C2orf28 | -3.04E+00 | 2.40E-03 | 5.84E-03 |
| UBE3A | -3.04E+00 | 2.40E-03 | 5.85E-03 |
| SLITRK6 | -3.04E+00 | 2.40E-03 | 5.86E-03 |
| SETD7 | -3.04E+00 | 2.40E-03 | 5.86E-03 |
| ST6GALNAC1 | -3.04E+00 | 2.40E-03 | 5.86E-03 |
| LCE1F | 3.04E+00 | 2.40E-03 | 5.86E-03 |
| C15orf43 | -3.04E+00 | 2.41E-03 | 5.87E-03 |
| CHSY3 | -3.04E+00 | 2.41E-03 | 5.87E-03 |
| SCD5 | -3.04E+00 | 2.41E-03 | 5.87E-03 |
| MBL1P | -3.03E+00 | 2.41E-03 | 5.88E-03 |
| NHP2 | 3.03E+00 | 2.42E-03 | 5.89E-03 |
| KRTCAP3 | -3.03E+00 | 2.42E-03 | 5.89E-03 |
| SLC4A5 | 3.03E+00 | 2.42E-03 | 5.89E-03 |
| ZFP90 | -3.03E+00 | 2.42E-03 | 5.89E-03 |
| IL6ST | -3.03E+00 | 2.42E-03 | 5.90E-03 |
| FLT3 | -3.03E+00 | 2.42E-03 | 5.90E-03 |
| C6orf35 | -3.03E+00 | 2.43E-03 | 5.90E-03 |
| AIDA | -3.03E+00 | 2.43E-03 | 5.91E-03 |
| RNF8 | 3.03E+00 | 2.43E-03 | 5.92E-03 |
| SNORA67 | 3.03E+00 | 2.43E-03 | 5.92E-03 |
| FOXB1 | 3.03E+00 | 2.43E-03 | 5.92E-03 |
| LOC221710 | 3.03E+00 | 2.43E-03 | 5.92E-03 |
| OSBPL11 | -3.03E+00 | 2.44E-03 | 5.93E-03 |
| BTBD19 | 3.03E+00 | 2.44E-03 | 5.93E-03 |
| ISM1 | -3.03E+00 | 2.44E-03 | 5.94E-03 |
| RGMB | -3.03E+00 | 2.44E-03 | 5.95E-03 |
| ZNF583 | -3.03E+00 | 2.44E-03 | 5.95E-03 |
| DDX60 | -3.03E+00 | 2.45E-03 | 5.95E-03 |
| LOC442459 | 3.03E+00 | 2.45E-03 | 5.96E-03 |
| FAM21C | 3.03E+00 | 2.46E-03 | 5.97E-03 |
| GLIS1 | 3.03E+00 | 2.46E-03 | 5.97E-03 |
| DSG4 | 3.03E+00 | 2.46E-03 | 5.98E-03 |
| LOC100129534 | -3.03E+00 | 2.46E-03 | 5.98E-03 |
| KIAA1715 | -3.03E+00 | 2.46E-03 | 5.98E-03 |
| LOC647288 | -3.03E+00 | 2.46E-03 | 5.98E-03 |
| MAGEB18 | 3.03E+00 | 2.47E-03 | 5.99E-03 |
| STX5 | -3.03E+00 | 2.47E-03 | 5.99E-03 |
| VENTXP7 | 3.03E+00 | 2.47E-03 | 5.99E-03 |

| LOC84931 | 3.03E+00 | 2.47E-03 | 6.00E-03 |
| --- | --- | --- | --- |
| EAF1 | 3.03E+00 | 2.48E-03 | 6.02E-03 |
| GUCA2A | 3.03E+00 | 2.49E-03 | 6.03E-03 |
| C9orf78 | -3.03E+00 | 2.49E-03 | 6.04E-03 |
| LOC283404 | 3.02E+00 | 2.49E-03 | 6.05E-03 |
| GPSM1 | 3.02E+00 | 2.50E-03 | 6.05E-03 |
| RABL2A | -3.02E+00 | 2.50E-03 | 6.06E-03 |
| NTAN1 | -3.02E+00 | 2.50E-03 | 6.06E-03 |
| AMH | 3.02E+00 | 2.50E-03 | 6.07E-03 |
| CD69 | -3.02E+00 | 2.50E-03 | 6.07E-03 |
| PLGLA | -3.02E+00 | 2.51E-03 | 6.08E-03 |
| PDZD4 | -3.02E+00 | 2.51E-03 | 6.08E-03 |
| TUBG2 | 3.02E+00 | 2.51E-03 | 6.10E-03 |
| CEACAM16 | 3.02E+00 | 2.52E-03 | 6.11E-03 |
| ARHGEF15 | -3.02E+00 | 2.52E-03 | 6.11E-03 |
| PKD2 | -3.02E+00 | 2.52E-03 | 6.11E-03 |
| ZNF560 | 3.02E+00 | 2.52E-03 | 6.11E-03 |
| ZNF582 | -3.02E+00 | 2.52E-03 | 6.11E-03 |
| NLRP7 | 3.02E+00 | 2.53E-03 | 6.13E-03 |
| SMAGP | 3.02E+00 | 2.53E-03 | 6.13E-03 |
| MRPS16 | 3.02E+00 | 2.54E-03 | 6.14E-03 |
| HRH4 | -3.02E+00 | 2.54E-03 | 6.14E-03 |
| OR2C1 | -3.02E+00 | 2.54E-03 | 6.15E-03 |
| COPS4 | -3.02E+00 | 2.54E-03 | 6.15E-03 |
| ZBTB26 | 3.02E+00 | 2.55E-03 | 6.16E-03 |
| ITGA2B | 3.02E+00 | 2.55E-03 | 6.17E-03 |
| TMEM115 | 3.02E+00 | 2.55E-03 | 6.17E-03 |
| MMP28 | -3.02E+00 | 2.55E-03 | 6.17E-03 |
| FOXC1 | -3.02E+00 | 2.55E-03 | 6.17E-03 |
| PLEKHB1 | 3.02E+00 | 2.56E-03 | 6.19E-03 |
| ANKRD17 | 3.02E+00 | 2.56E-03 | 6.19E-03 |
| ERI3 | 3.02E+00 | 2.56E-03 | 6.19E-03 |
| COL11A2 | 3.02E+00 | 2.56E-03 | 6.20E-03 |
| ZNF613 | -3.02E+00 | 2.57E-03 | 6.21E-03 |
| TMEM14B | -3.02E+00 | 2.57E-03 | 6.21E-03 |
| VGLL4 | 3.02E+00 | 2.57E-03 | 6.22E-03 |
| GUCY1B3 | -3.01E+00 | 2.57E-03 | 6.22E-03 |
| ZNF215 | 3.01E+00 | 2.58E-03 | 6.23E-03 |
| FAM167A | -3.01E+00 | 2.58E-03 | 6.23E-03 |
| SEC23B | 3.01E+00 | 2.59E-03 | 6.26E-03 |
| TMEM164 | 3.01E+00 | 2.59E-03 | 6.26E-03 |
| ANO1 | -3.01E+00 | 2.60E-03 | 6.28E-03 |
| SLC43A3 | -3.01E+00 | 2.60E-03 | 6.28E-03 |
| C14orf129 | -3.01E+00 | 2.60E-03 | 6.29E-03 |
| PLEKHO1 | 3.01E+00 | 2.61E-03 | 6.29E-03 |
| MARVELD1 | 3.01E+00 | 2.61E-03 | 6.31E-03 |
| SLC25A21 | 3.01E+00 | 2.61E-03 | 6.31E-03 |
| SNHG10 | 3.01E+00 | 2.62E-03 | 6.33E-03 |
| C6orf142 | -3.01E+00 | 2.62E-03 | 6.34E-03 |
| CSNK2B | 3.01E+00 | 2.64E-03 | 6.38E-03 |
| PRSS1 | 3.01E+00 | 2.65E-03 | 6.39E-03 |
| FAM187B | -3.01E+00 | 2.65E-03 | 6.39E-03 |
| CCDC7 | -3.01E+00 | 2.65E-03 | 6.40E-03 |
| SLC46A2 | -3.01E+00 | 2.66E-03 | 6.41E-03 |
| POLM | 3.01E+00 | 2.66E-03 | 6.42E-03 |
| CTDSP2 | 3.00E+00 | 2.66E-03 | 6.42E-03 |
| GADL1 | -3.00E+00 | 2.67E-03 | 6.43E-03 |

| C19orf77 | 3.00E+00 | 2.67E-03 | 6.43E-03 |
| --- | --- | --- | --- |
| GK3P | -3.00E+00 | 2.67E-03 | 6.43E-03 |
| CGGBP1 | -3.00E+00 | 2.68E-03 | 6.45E-03 |
| ATOH1 | 3.00E+00 | 2.68E-03 | 6.45E-03 |
| CETN2 | -3.00E+00 | 2.68E-03 | 6.46E-03 |
| ROM1 | -3.00E+00 | 2.68E-03 | 6.46E-03 |
| TTC8 | -3.00E+00 | 2.69E-03 | 6.47E-03 |
| ZKSCAN3 | 3.00E+00 | 2.69E-03 | 6.48E-03 |
| LOC286135 | 3.00E+00 | 2.70E-03 | 6.49E-03 |
| TIFA | -3.00E+00 | 2.70E-03 | 6.49E-03 |
| RGS22 | -3.00E+00 | 2.70E-03 | 6.50E-03 |
| CSNK1A1 | -3.00E+00 | 2.70E-03 | 6.51E-03 |
| CENPQ | 3.00E+00 | 2.71E-03 | 6.52E-03 |
| RSPH10B2 | -3.00E+00 | 2.71E-03 | 6.52E-03 |
| DIS3L2 | 3.00E+00 | 2.71E-03 | 6.52E-03 |
| C15orf5 | -3.00E+00 | 2.71E-03 | 6.53E-03 |
| CLDN6 | 3.00E+00 | 2.71E-03 | 6.53E-03 |
| HTR7P1 | -3.00E+00 | 2.71E-03 | 6.53E-03 |
| ZRANB3 | 3.00E+00 | 2.71E-03 | 6.53E-03 |
| GDPD3 | 3.00E+00 | 2.72E-03 | 6.53E-03 |
| IL31RA | 3.00E+00 | 2.72E-03 | 6.54E-03 |
| NEK6 | 3.00E+00 | 2.72E-03 | 6.54E-03 |
| ECEL1 | 3.00E+00 | 2.72E-03 | 6.54E-03 |
| TAS2R4 | 3.00E+00 | 2.73E-03 | 6.57E-03 |
| SLC6A13 | -3.00E+00 | 2.73E-03 | 6.57E-03 |
| SCN7A | -3.00E+00 | 2.74E-03 | 6.58E-03 |
| C19orf54 | 3.00E+00 | 2.74E-03 | 6.58E-03 |
| GLUL | -3.00E+00 | 2.75E-03 | 6.60E-03 |
| LOC100130987 | 3.00E+00 | 2.75E-03 | 6.60E-03 |
| HIST2H2AC | 2.99E+00 | 2.75E-03 | 6.61E-03 |
| RAP1GDS1 | -2.99E+00 | 2.76E-03 | 6.63E-03 |
| TMEM204 | -2.99E+00 | 2.76E-03 | 6.63E-03 |
| SESN3 | -2.99E+00 | 2.76E-03 | 6.63E-03 |
| SGMS1 | -2.99E+00 | 2.76E-03 | 6.64E-03 |
| UNC13B | -2.99E+00 | 2.77E-03 | 6.64E-03 |
| SERPINB4 | 2.99E+00 | 2.77E-03 | 6.64E-03 |
| NIF3L1 | 2.99E+00 | 2.77E-03 | 6.65E-03 |
| ALS2 | 2.99E+00 | 2.77E-03 | 6.65E-03 |
| ESYT1 | 2.99E+00 | 2.77E-03 | 6.65E-03 |
| AMD1 | 2.99E+00 | 2.78E-03 | 6.68E-03 |
| C2 | -2.99E+00 | 2.79E-03 | 6.68E-03 |
| PPAPDC1A | 2.99E+00 | 2.79E-03 | 6.69E-03 |
| ZMYM5 | -2.99E+00 | 2.79E-03 | 6.69E-03 |
| SNX24 | -2.99E+00 | 2.80E-03 | 6.71E-03 |
| TIGIT | 2.99E+00 | 2.80E-03 | 6.71E-03 |
| ERICH1 | 2.99E+00 | 2.80E-03 | 6.73E-03 |
| HHIP | -2.99E+00 | 2.81E-03 | 6.74E-03 |
| FLJ46321 | 2.99E+00 | 2.81E-03 | 6.75E-03 |
| ATF5 | 2.99E+00 | 2.82E-03 | 6.75E-03 |
| INTS12 | -2.99E+00 | 2.82E-03 | 6.75E-03 |
| C16orf79 | 2.99E+00 | 2.82E-03 | 6.76E-03 |
| DEFB103B | 2.99E+00 | 2.83E-03 | 6.77E-03 |
| LRRC55 | -2.99E+00 | 2.83E-03 | 6.78E-03 |
| TMEM111 | -2.99E+00 | 2.83E-03 | 6.78E-03 |
| ATP5G2 | 2.99E+00 | 2.83E-03 | 6.78E-03 |
| C5orf25 | -2.99E+00 | 2.83E-03 | 6.79E-03 |
| GPR98 | -2.99E+00 | 2.84E-03 | 6.79E-03 |

| ZCCHC2 | 2.99E+00 | 2.84E-03 | 6.80E-03 |
| --- | --- | --- | --- |
| EPB42 | -2.98E+00 | 2.84E-03 | 6.80E-03 |
| SERPINB5 | 2.98E+00 | 2.84E-03 | 6.80E-03 |
| TUBB8 | 2.98E+00 | 2.84E-03 | 6.80E-03 |
| RG9MTD2 | -2.98E+00 | 2.84E-03 | 6.80E-03 |
| PLGLB2 | -2.98E+00 | 2.84E-03 | 6.81E-03 |
| SCARNA7 | -2.98E+00 | 2.86E-03 | 6.83E-03 |
| SEC11A | -2.98E+00 | 2.86E-03 | 6.83E-03 |
| SLC9A1 | 2.98E+00 | 2.86E-03 | 6.83E-03 |
| FOXA2 | -2.98E+00 | 2.87E-03 | 6.86E-03 |
| RPAIN | -2.98E+00 | 2.87E-03 | 6.87E-03 |
| SIRT2 | -2.98E+00 | 2.87E-03 | 6.87E-03 |
| HECA | -2.98E+00 | 2.88E-03 | 6.88E-03 |
| ADAMTS1 | -2.98E+00 | 2.88E-03 | 6.89E-03 |
| SNHG11 | 2.98E+00 | 2.89E-03 | 6.91E-03 |
| MAG | -2.98E+00 | 2.90E-03 | 6.92E-03 |
| UMODL1 | 2.98E+00 | 2.90E-03 | 6.92E-03 |
| GRLF1 | 2.98E+00 | 2.90E-03 | 6.92E-03 |
| KCNK1 | -2.98E+00 | 2.90E-03 | 6.92E-03 |
| C11orf58 | -2.98E+00 | 2.90E-03 | 6.93E-03 |
| TMEM55B | 2.98E+00 | 2.91E-03 | 6.94E-03 |
| EPAS1 | -2.98E+00 | 2.91E-03 | 6.95E-03 |
| XG | 2.98E+00 | 2.91E-03 | 6.95E-03 |
| VPS13C | -2.98E+00 | 2.91E-03 | 6.95E-03 |
| PENK | -2.98E+00 | 2.91E-03 | 6.96E-03 |
| PAGE5 | 2.98E+00 | 2.92E-03 | 6.96E-03 |
| SHISA5 | 2.98E+00 | 2.92E-03 | 6.98E-03 |
| SCNN1G | -2.98E+00 | 2.93E-03 | 6.99E-03 |
| LOC90246 | -2.98E+00 | 2.93E-03 | 7.00E-03 |
| UBE2A | -2.97E+00 | 2.94E-03 | 7.01E-03 |
| GRIK1 | 2.97E+00 | 2.94E-03 | 7.01E-03 |
| LEF1 | 2.97E+00 | 2.94E-03 | 7.02E-03 |
| TESC | 2.97E+00 | 2.96E-03 | 7.05E-03 |
| FASTKD1 | 2.97E+00 | 2.96E-03 | 7.05E-03 |
| ZNF778 | -2.97E+00 | 2.97E-03 | 7.08E-03 |
| CCDC140 | 2.97E+00 | 2.97E-03 | 7.08E-03 |
| RALYL | 2.97E+00 | 2.97E-03 | 7.08E-03 |
| KCNK10 | 2.97E+00 | 2.99E-03 | 7.13E-03 |
| HMGXB4 | 2.97E+00 | 3.00E-03 | 7.14E-03 |
| CASS4 | -2.97E+00 | 3.00E-03 | 7.14E-03 |
| TOPORS | -2.97E+00 | 3.00E-03 | 7.16E-03 |
| DPCR1 | -2.97E+00 | 3.01E-03 | 7.17E-03 |
| SRY | 2.97E+00 | 3.02E-03 | 7.19E-03 |
| USP34 | 2.97E+00 | 3.02E-03 | 7.19E-03 |
| DTX4 | -2.97E+00 | 3.02E-03 | 7.19E-03 |
| ANXA3 | -2.97E+00 | 3.02E-03 | 7.20E-03 |
| PSTPIP2 | -2.97E+00 | 3.02E-03 | 7.20E-03 |
| MRPS23 | 2.97E+00 | 3.03E-03 | 7.20E-03 |
| ZNF644 | -2.97E+00 | 3.03E-03 | 7.21E-03 |
| AGAP6 | 2.96E+00 | 3.04E-03 | 7.24E-03 |
| GOLGA2B | 2.96E+00 | 3.04E-03 | 7.24E-03 |
| DPY30 | -2.96E+00 | 3.05E-03 | 7.25E-03 |
| TTC25 | -2.96E+00 | 3.05E-03 | 7.25E-03 |
| PIP5K1A | 2.96E+00 | 3.06E-03 | 7.27E-03 |
| FLVCR2 | -2.96E+00 | 3.06E-03 | 7.28E-03 |
| TBX3 | -2.96E+00 | 3.06E-03 | 7.28E-03 |
| ID4 | -2.96E+00 | 3.06E-03 | 7.29E-03 |

| MALL | -2.96E+00 | 3.07E-03 | 7.31E-03 |
| --- | --- | --- | --- |
| PLXND1 | 2.96E+00 | 3.08E-03 | 7.32E-03 |
| SOD3 | -2.96E+00 | 3.08E-03 | 7.32E-03 |
| COX11 | -2.96E+00 | 3.09E-03 | 7.34E-03 |
| BTNL2 | 2.96E+00 | 3.10E-03 | 7.35E-03 |
| FLNB | 2.96E+00 | 3.10E-03 | 7.36E-03 |
| AMPH | -2.96E+00 | 3.10E-03 | 7.37E-03 |
| TBC1D3H | 2.96E+00 | 3.11E-03 | 7.38E-03 |
| LYVE1 | -2.96E+00 | 3.11E-03 | 7.39E-03 |
| NLGN4Y | 2.96E+00 | 3.11E-03 | 7.39E-03 |
| PHC3 | -2.96E+00 | 3.11E-03 | 7.39E-03 |
| ZNF41 | -2.96E+00 | 3.11E-03 | 7.39E-03 |
| RFPL1S | 2.96E+00 | 3.12E-03 | 7.40E-03 |
| PLS1 | -2.96E+00 | 3.12E-03 | 7.40E-03 |
| NRSN1 | 2.95E+00 | 3.14E-03 | 7.44E-03 |
| PIAS2 | 2.95E+00 | 3.14E-03 | 7.44E-03 |
| ACSBG1 | -2.95E+00 | 3.14E-03 | 7.45E-03 |
| SYTL4 | -2.95E+00 | 3.14E-03 | 7.46E-03 |
| CABP7 | 2.95E+00 | 3.14E-03 | 7.46E-03 |
| CLDN1 | -2.95E+00 | 3.15E-03 | 7.48E-03 |
| CINP | -2.95E+00 | 3.16E-03 | 7.49E-03 |
| CMTM3 | 2.95E+00 | 3.17E-03 | 7.52E-03 |
| ANKRD56 | -2.95E+00 | 3.17E-03 | 7.52E-03 |
| ABHD10 | -2.95E+00 | 3.17E-03 | 7.52E-03 |
| TXN2 | -2.95E+00 | 3.17E-03 | 7.52E-03 |
| MYL5 | -2.95E+00 | 3.18E-03 | 7.53E-03 |
| ADRA2B | -2.95E+00 | 3.18E-03 | 7.54E-03 |
| TM2D2 | -2.95E+00 | 3.18E-03 | 7.54E-03 |
| ANKRD2 | 2.95E+00 | 3.19E-03 | 7.55E-03 |
| CATSPER3 | -2.95E+00 | 3.20E-03 | 7.57E-03 |
| MAPK6 | 2.95E+00 | 3.20E-03 | 7.58E-03 |
| PRAMEF20 | 2.95E+00 | 3.20E-03 | 7.58E-03 |
| CMTM7 | 2.95E+00 | 3.20E-03 | 7.58E-03 |
| OR9Q1 | -2.95E+00 | 3.20E-03 | 7.59E-03 |
| LRRC3 | -2.95E+00 | 3.21E-03 | 7.59E-03 |
| ATP8B5P | -2.95E+00 | 3.21E-03 | 7.60E-03 |
| KIAA0391 | -2.95E+00 | 3.21E-03 | 7.60E-03 |
| FAM35B2 | -2.95E+00 | 3.21E-03 | 7.61E-03 |
| FBXO36 | -2.95E+00 | 3.22E-03 | 7.61E-03 |
| WHAMML2 | -2.95E+00 | 3.22E-03 | 7.63E-03 |
| SRP72 | 2.95E+00 | 3.23E-03 | 7.63E-03 |
| LOC388387 | -2.95E+00 | 3.23E-03 | 7.65E-03 |
| SERPINE1 | 2.95E+00 | 3.23E-03 | 7.65E-03 |
| AMOT | -2.94E+00 | 3.24E-03 | 7.65E-03 |
| CYFIP2 | -2.94E+00 | 3.24E-03 | 7.65E-03 |
| ZNF221 | 2.94E+00 | 3.24E-03 | 7.66E-03 |
| OR2L2 | 2.94E+00 | 3.24E-03 | 7.66E-03 |
| AFAP1 | 2.94E+00 | 3.24E-03 | 7.66E-03 |
| POLR1E | 2.94E+00 | 3.24E-03 | 7.66E-03 |
| ATP5H | -2.94E+00 | 3.24E-03 | 7.67E-03 |
| GPR113 | 2.94E+00 | 3.25E-03 | 7.67E-03 |
| DOK4 | -2.94E+00 | 3.25E-03 | 7.68E-03 |
| LOC115110 | -2.94E+00 | 3.25E-03 | 7.68E-03 |
| CCL4L2 | 2.94E+00 | 3.25E-03 | 7.68E-03 |
| RAD17 | -2.94E+00 | 3.25E-03 | 7.68E-03 |
| FLJ43663 | -2.94E+00 | 3.25E-03 | 7.69E-03 |
| PBXIP1 | -2.94E+00 | 3.26E-03 | 7.69E-03 |

| FKBP14 | 2.94E+00 | 3.26E-03 | 7.70E-03 |
| --- | --- | --- | --- |
| TRPC7 | 2.94E+00 | 3.28E-03 | 7.73E-03 |
| HSD11B1 | -2.94E+00 | 3.28E-03 | 7.74E-03 |
| ROPN1 | -2.94E+00 | 3.28E-03 | 7.74E-03 |
| LYPD6B | 2.94E+00 | 3.28E-03 | 7.74E-03 |
| PSMD7 | 2.94E+00 | 3.29E-03 | 7.75E-03 |
| SCARNA5 | -2.94E+00 | 3.29E-03 | 7.76E-03 |
| C12orf41 | 2.94E+00 | 3.30E-03 | 7.78E-03 |
| SLC4A3 | 2.94E+00 | 3.30E-03 | 7.78E-03 |
| APRT | 2.94E+00 | 3.30E-03 | 7.78E-03 |
| FLJ40292 | 2.94E+00 | 3.30E-03 | 7.78E-03 |
| PRELID1 | 2.94E+00 | 3.30E-03 | 7.79E-03 |
| C6orf176 | 2.94E+00 | 3.31E-03 | 7.80E-03 |
| GPCPD1 | -2.94E+00 | 3.31E-03 | 7.80E-03 |
| PRR22 | 2.94E+00 | 3.31E-03 | 7.80E-03 |
| FAM19A2 | -2.94E+00 | 3.31E-03 | 7.81E-03 |
| COL3A1 | 2.94E+00 | 3.33E-03 | 7.84E-03 |
| MED30 | 2.94E+00 | 3.33E-03 | 7.84E-03 |
| GNAO1 | -2.94E+00 | 3.33E-03 | 7.84E-03 |
| MPND | -2.94E+00 | 3.33E-03 | 7.85E-03 |
| UBR3 | -2.94E+00 | 3.33E-03 | 7.85E-03 |
| UFM1 | -2.94E+00 | 3.34E-03 | 7.86E-03 |
| RBMY1J | 2.93E+00 | 3.35E-03 | 7.88E-03 |
| KLK9 | 2.93E+00 | 3.35E-03 | 7.88E-03 |
| SERINC4 | 2.93E+00 | 3.35E-03 | 7.89E-03 |
| CTNS | 2.93E+00 | 3.35E-03 | 7.89E-03 |
| MOG | -2.93E+00 | 3.35E-03 | 7.89E-03 |
| GEM | -2.93E+00 | 3.35E-03 | 7.89E-03 |
| GADD45B | -2.93E+00 | 3.36E-03 | 7.90E-03 |
| PLD2 | 2.93E+00 | 3.36E-03 | 7.91E-03 |
| C17orf72 | -2.93E+00 | 3.36E-03 | 7.91E-03 |
| TAAR9 | 2.93E+00 | 3.36E-03 | 7.91E-03 |
| JRKL | 2.93E+00 | 3.36E-03 | 7.91E-03 |
| C6orf57 | -2.93E+00 | 3.36E-03 | 7.91E-03 |
| GPR15 | 2.93E+00 | 3.38E-03 | 7.94E-03 |
| SPIN4 | 2.93E+00 | 3.38E-03 | 7.95E-03 |
| POLR3F | 2.93E+00 | 3.38E-03 | 7.95E-03 |
| ARHGAP5 | -2.93E+00 | 3.38E-03 | 7.95E-03 |
| LOC100128076 | 2.93E+00 | 3.39E-03 | 7.96E-03 |
| N6AMT2 | -2.93E+00 | 3.40E-03 | 7.98E-03 |
| CYTSB | 2.93E+00 | 3.40E-03 | 7.99E-03 |
| KRTAP5-7 | 2.93E+00 | 3.41E-03 | 8.01E-03 |
| C1QC | 2.93E+00 | 3.41E-03 | 8.02E-03 |
| CCL8 | 2.93E+00 | 3.41E-03 | 8.02E-03 |
| GOLPH3 | -2.93E+00 | 3.42E-03 | 8.02E-03 |
| CCDC101 | -2.93E+00 | 3.42E-03 | 8.03E-03 |
| MAGI3 | -2.93E+00 | 3.43E-03 | 8.05E-03 |
| TBL1X | 2.93E+00 | 3.43E-03 | 8.05E-03 |
| MDFIC | -2.93E+00 | 3.43E-03 | 8.05E-03 |
| PRAMEF8 | -2.93E+00 | 3.43E-03 | 8.06E-03 |
| NARFL | 2.93E+00 | 3.44E-03 | 8.07E-03 |
| UQCR10 | -2.93E+00 | 3.44E-03 | 8.07E-03 |
| SIX2 | 2.93E+00 | 3.44E-03 | 8.08E-03 |
| C19orf56 | -2.93E+00 | 3.45E-03 | 8.09E-03 |
| ACO2 | 2.93E+00 | 3.45E-03 | 8.09E-03 |
| C9orf130 | -2.92E+00 | 3.45E-03 | 8.10E-03 |
| SPINT1 | 2.92E+00 | 3.46E-03 | 8.11E-03 |

| HIST1H3J | 2.92E+00 | 3.47E-03 | 8.13E-03 |
| --- | --- | --- | --- |
| SLAMF8 | 2.92E+00 | 3.47E-03 | 8.14E-03 |
| FAM127A | 2.92E+00 | 3.47E-03 | 8.14E-03 |
| PRG1 | 2.92E+00 | 3.47E-03 | 8.14E-03 |
| RSAD1 | -2.92E+00 | 3.47E-03 | 8.14E-03 |
| TBC1D2 | -2.92E+00 | 3.48E-03 | 8.15E-03 |
| EPHA5 | 2.92E+00 | 3.49E-03 | 8.17E-03 |
| EFCAB4B | 2.92E+00 | 3.49E-03 | 8.18E-03 |
| CPLX1 | 2.92E+00 | 3.49E-03 | 8.18E-03 |
| FLRT3 | -2.92E+00 | 3.49E-03 | 8.18E-03 |
| PRKG1 | -2.92E+00 | 3.49E-03 | 8.18E-03 |
| RPS4Y1 | 2.92E+00 | 3.50E-03 | 8.19E-03 |
| TARBP1 | 2.92E+00 | 3.50E-03 | 8.19E-03 |
| C17orf75 | 2.92E+00 | 3.50E-03 | 8.19E-03 |
| NFASC | -2.92E+00 | 3.50E-03 | 8.19E-03 |
| ESM1 | 2.92E+00 | 3.50E-03 | 8.20E-03 |
| ZNF131 | 2.92E+00 | 3.50E-03 | 8.20E-03 |
| DEF8 | 2.92E+00 | 3.52E-03 | 8.23E-03 |
| TMEM196 | 2.92E+00 | 3.52E-03 | 8.23E-03 |
| AURKAIP1 | 2.92E+00 | 3.52E-03 | 8.23E-03 |
| IER5 | 2.92E+00 | 3.52E-03 | 8.24E-03 |
| GJA4 | -2.92E+00 | 3.52E-03 | 8.24E-03 |
| TREX1 | -2.92E+00 | 3.52E-03 | 8.24E-03 |
| GPR109A | 2.92E+00 | 3.53E-03 | 8.24E-03 |
| TCEAL6 | -2.92E+00 | 3.53E-03 | 8.24E-03 |
| UBAP1 | -2.92E+00 | 3.53E-03 | 8.24E-03 |
| ARPC5L | 2.92E+00 | 3.53E-03 | 8.25E-03 |
| C9orf96 | -2.92E+00 | 3.55E-03 | 8.29E-03 |
| C1QTNF3 | -2.92E+00 | 3.55E-03 | 8.29E-03 |
| CCDC115 | -2.92E+00 | 3.55E-03 | 8.30E-03 |
| COTL1 | 2.92E+00 | 3.56E-03 | 8.31E-03 |
| C21orf57 | -2.92E+00 | 3.56E-03 | 8.32E-03 |
| CYP46A1 | -2.91E+00 | 3.57E-03 | 8.34E-03 |
| MGAT2 | -2.91E+00 | 3.57E-03 | 8.34E-03 |
| DRAM1 | 2.91E+00 | 3.57E-03 | 8.35E-03 |
| ANKRD13C | -2.91E+00 | 3.57E-03 | 8.35E-03 |
| WDR73 | 2.91E+00 | 3.58E-03 | 8.35E-03 |
| TEX261 | 2.91E+00 | 3.58E-03 | 8.35E-03 |
| POU2AF1 | 2.91E+00 | 3.58E-03 | 8.35E-03 |
| GLUD1 | -2.91E+00 | 3.58E-03 | 8.36E-03 |
| TFAMP1 | 2.91E+00 | 3.59E-03 | 8.37E-03 |
| C12orf62 | -2.91E+00 | 3.59E-03 | 8.37E-03 |
| NFE2L3 | 2.91E+00 | 3.59E-03 | 8.38E-03 |
| ETS1 | -2.91E+00 | 3.60E-03 | 8.39E-03 |
| ZNF423 | -2.91E+00 | 3.60E-03 | 8.39E-03 |
| NFATC2IP | 2.91E+00 | 3.60E-03 | 8.40E-03 |
| ANKRD46 | -2.91E+00 | 3.60E-03 | 8.40E-03 |
| GOLGA8E | 2.91E+00 | 3.60E-03 | 8.41E-03 |
| GTF3C6 | 2.91E+00 | 3.61E-03 | 8.42E-03 |
| F2R | -2.91E+00 | 3.61E-03 | 8.42E-03 |
| PAR5 | 2.91E+00 | 3.61E-03 | 8.42E-03 |
| PCDH8 | 2.91E+00 | 3.61E-03 | 8.42E-03 |
| LOC441869 | -2.91E+00 | 3.62E-03 | 8.43E-03 |
| CLDN5 | -2.91E+00 | 3.63E-03 | 8.45E-03 |
| THADA | 2.91E+00 | 3.63E-03 | 8.46E-03 |
| C21orf121 | -2.91E+00 | 3.63E-03 | 8.47E-03 |
| FSCN2 | 2.91E+00 | 3.65E-03 | 8.49E-03 |

| OR52E4 | 2.91E+00 | 3.65E-03 | 8.51E-03 |
| --- | --- | --- | --- |
| HCP5 | -2.91E+00 | 3.65E-03 | 8.51E-03 |
| SPINT3 | -2.91E+00 | 3.66E-03 | 8.51E-03 |
| CAMK2N2 | 2.91E+00 | 3.66E-03 | 8.52E-03 |
| F10 | -2.91E+00 | 3.67E-03 | 8.54E-03 |
| MYOZ2 | -2.91E+00 | 3.67E-03 | 8.55E-03 |
| PTGFR | -2.91E+00 | 3.67E-03 | 8.55E-03 |
| GPR1 | 2.91E+00 | 3.67E-03 | 8.55E-03 |
| FAM73B | 2.90E+00 | 3.68E-03 | 8.56E-03 |
| MFSD4 | -2.90E+00 | 3.68E-03 | 8.57E-03 |
| SUMF2 | -2.90E+00 | 3.69E-03 | 8.58E-03 |
| C21orf91 | -2.90E+00 | 3.70E-03 | 8.60E-03 |
| CITED2 | -2.90E+00 | 3.70E-03 | 8.60E-03 |
| DEGS2 | -2.90E+00 | 3.70E-03 | 8.60E-03 |
| MGAT5 | 2.90E+00 | 3.70E-03 | 8.60E-03 |
| SGEF | -2.90E+00 | 3.70E-03 | 8.60E-03 |
| TMSB10 | 2.90E+00 | 3.70E-03 | 8.60E-03 |
| AK2 | -2.90E+00 | 3.70E-03 | 8.61E-03 |
| LASS4 | -2.90E+00 | 3.71E-03 | 8.62E-03 |
| NUP62CL | 2.90E+00 | 3.71E-03 | 8.63E-03 |
| AGA | -2.90E+00 | 3.72E-03 | 8.63E-03 |
| CDH13 | -2.90E+00 | 3.72E-03 | 8.63E-03 |
| OR2A14 | 2.90E+00 | 3.72E-03 | 8.63E-03 |
| FCAR | 2.90E+00 | 3.72E-03 | 8.64E-03 |
| TTC9B | 2.90E+00 | 3.72E-03 | 8.64E-03 |
| DHRS4 | -2.90E+00 | 3.73E-03 | 8.66E-03 |
| MST1P2 | -2.90E+00 | 3.74E-03 | 8.69E-03 |
| TOMM70A | 2.90E+00 | 3.75E-03 | 8.71E-03 |
| NCRNA00105 | 2.90E+00 | 3.75E-03 | 8.71E-03 |
| PTP4A1 | -2.90E+00 | 3.76E-03 | 8.72E-03 |
| DDX50 | 2.90E+00 | 3.76E-03 | 8.73E-03 |
| LOC613037 | 2.90E+00 | 3.77E-03 | 8.74E-03 |
| TMEM97 | 2.90E+00 | 3.77E-03 | 8.75E-03 |
| TMEM176A | -2.90E+00 | 3.78E-03 | 8.76E-03 |
| CEP170 | 2.90E+00 | 3.78E-03 | 8.77E-03 |
| ASTN2 | -2.90E+00 | 3.78E-03 | 8.77E-03 |
| C13orf1 | -2.90E+00 | 3.78E-03 | 8.78E-03 |
| KIR3DX1 | 2.90E+00 | 3.79E-03 | 8.78E-03 |
| IPO5 | 2.90E+00 | 3.79E-03 | 8.78E-03 |
| ARFIP2 | 2.90E+00 | 3.79E-03 | 8.78E-03 |
| MORN2 | -2.90E+00 | 3.80E-03 | 8.80E-03 |
| RPSAP58 | 2.89E+00 | 3.81E-03 | 8.83E-03 |
| ZNF35 | -2.89E+00 | 3.81E-03 | 8.83E-03 |
| MLX | -2.89E+00 | 3.81E-03 | 8.84E-03 |
| FEZ1 | -2.89E+00 | 3.82E-03 | 8.86E-03 |
| HMGCR | 2.89E+00 | 3.83E-03 | 8.86E-03 |
| GYLTL1B | 2.89E+00 | 3.83E-03 | 8.88E-03 |
| SGSM2 | 2.89E+00 | 3.83E-03 | 8.88E-03 |
| YES1 | 2.89E+00 | 3.83E-03 | 8.88E-03 |
| TWF1 | 2.89E+00 | 3.84E-03 | 8.88E-03 |
| ACTB | 2.89E+00 | 3.84E-03 | 8.89E-03 |
| NAT15 | 2.89E+00 | 3.85E-03 | 8.92E-03 |
| B3GNT5 | 2.89E+00 | 3.86E-03 | 8.94E-03 |
| TCEAL3 | -2.89E+00 | 3.86E-03 | 8.94E-03 |
| PEX3 | -2.89E+00 | 3.86E-03 | 8.94E-03 |
| HES5 | -2.89E+00 | 3.86E-03 | 8.94E-03 |
| C20orf3 | -2.89E+00 | 3.87E-03 | 8.95E-03 |

| DNM2 | 2.89E+00 | 3.87E-03 | 8.95E-03 |
| --- | --- | --- | --- |
| OR11H6 | 2.89E+00 | 3.87E-03 | 8.95E-03 |
| CYP26A1 | -2.89E+00 | 3.87E-03 | 8.95E-03 |
| C10orf118 | -2.89E+00 | 3.87E-03 | 8.95E-03 |
| CPA5 | 2.89E+00 | 3.87E-03 | 8.96E-03 |
| ATP6V1E2 | 2.89E+00 | 3.88E-03 | 8.96E-03 |
| MST4 | 2.89E+00 | 3.88E-03 | 8.96E-03 |
| EARS2 | 2.89E+00 | 3.88E-03 | 8.97E-03 |
| PCMTD2 | -2.89E+00 | 3.89E-03 | 8.99E-03 |
| ASAM | 2.89E+00 | 3.89E-03 | 9.00E-03 |
| ARC | -2.89E+00 | 3.90E-03 | 9.01E-03 |
| OR2AT4 | 2.89E+00 | 3.90E-03 | 9.02E-03 |
| RNF17 | 2.89E+00 | 3.91E-03 | 9.04E-03 |
| VEGFA | 2.88E+00 | 3.93E-03 | 9.07E-03 |
| GCNT4 | -2.88E+00 | 3.93E-03 | 9.07E-03 |
| LOC730101 | 2.88E+00 | 3.93E-03 | 9.07E-03 |
| DHRS4L2 | -2.88E+00 | 3.93E-03 | 9.08E-03 |
| MKL2 | -2.88E+00 | 3.94E-03 | 9.10E-03 |
| LRGUK | 2.88E+00 | 3.96E-03 | 9.14E-03 |
| TREH | -2.88E+00 | 3.97E-03 | 9.16E-03 |
| GOLPH3L | -2.88E+00 | 3.97E-03 | 9.16E-03 |
| RCN1 | 2.88E+00 | 3.97E-03 | 9.17E-03 |
| EHD3 | 2.88E+00 | 3.99E-03 | 9.20E-03 |
| MTMR9 | -2.88E+00 | 3.99E-03 | 9.20E-03 |
| EN1 | 2.88E+00 | 3.99E-03 | 9.21E-03 |
| SERPINE3 | 2.88E+00 | 3.99E-03 | 9.21E-03 |
| ABHD3 | 2.88E+00 | 4.00E-03 | 9.23E-03 |
| TAX1BP1 | -2.88E+00 | 4.00E-03 | 9.23E-03 |
| GPBP1 | -2.88E+00 | 4.02E-03 | 9.26E-03 |
| C1orf87 | -2.88E+00 | 4.02E-03 | 9.27E-03 |
| FBXL13 | 2.88E+00 | 4.02E-03 | 9.27E-03 |
| NME5 | -2.88E+00 | 4.02E-03 | 9.27E-03 |
| PLEKHG6 | 2.88E+00 | 4.02E-03 | 9.27E-03 |
| PHF17 | -2.88E+00 | 4.03E-03 | 9.28E-03 |
| CREB3L2 | 2.88E+00 | 4.03E-03 | 9.28E-03 |
| LOC644538 | -2.88E+00 | 4.03E-03 | 9.28E-03 |
| GPR39 | -2.88E+00 | 4.03E-03 | 9.28E-03 |
| COL9A2 | 2.88E+00 | 4.03E-03 | 9.29E-03 |
| HGC6.3 | 2.88E+00 | 4.04E-03 | 9.30E-03 |
| TMEM55A | 2.88E+00 | 4.04E-03 | 9.31E-03 |
| FAM131A | 2.88E+00 | 4.05E-03 | 9.31E-03 |
| HEXDC | 2.87E+00 | 4.05E-03 | 9.33E-03 |
| PPFIBP1 | 2.87E+00 | 4.06E-03 | 9.34E-03 |
| PNMAL2 | -2.87E+00 | 4.06E-03 | 9.34E-03 |
| A2M | -2.87E+00 | 4.06E-03 | 9.34E-03 |
| STX2 | 2.87E+00 | 4.06E-03 | 9.34E-03 |
| POLI | -2.87E+00 | 4.06E-03 | 9.35E-03 |
| ZNF304 | -2.87E+00 | 4.06E-03 | 9.35E-03 |
| SERTAD1 | -2.87E+00 | 4.07E-03 | 9.36E-03 |
| IBSP | 2.87E+00 | 4.07E-03 | 9.36E-03 |
| IPW | 2.87E+00 | 4.09E-03 | 9.39E-03 |
| TRIM21 | -2.87E+00 | 4.09E-03 | 9.40E-03 |
| BTBD3 | 2.87E+00 | 4.09E-03 | 9.41E-03 |
| WAC | 2.87E+00 | 4.10E-03 | 9.43E-03 |
| KCNQ4 | 2.87E+00 | 4.10E-03 | 9.44E-03 |
| SERINC3 | -2.87E+00 | 4.11E-03 | 9.44E-03 |
| SEC61A2 | 2.87E+00 | 4.11E-03 | 9.44E-03 |

| RFK | -2.87E+00 | 4.13E-03 | 9.48E-03 |
| --- | --- | --- | --- |
| TSPAN14 | 2.87E+00 | 4.13E-03 | 9.48E-03 |
| SNX25 | -2.87E+00 | 4.13E-03 | 9.49E-03 |
| HEBP2 | -2.87E+00 | 4.13E-03 | 9.49E-03 |
| IDO2 | -2.87E+00 | 4.13E-03 | 9.50E-03 |
| MMP24 | -2.87E+00 | 4.14E-03 | 9.50E-03 |
| LOC145837 | -2.87E+00 | 4.14E-03 | 9.51E-03 |
| SENP2 | 2.87E+00 | 4.15E-03 | 9.52E-03 |
| XBP1 | -2.87E+00 | 4.15E-03 | 9.53E-03 |
| TTC30B | -2.87E+00 | 4.17E-03 | 9.56E-03 |
| PARD6A | -2.87E+00 | 4.17E-03 | 9.57E-03 |
| RPA4 | 2.86E+00 | 4.19E-03 | 9.62E-03 |
| APOL2 | 2.86E+00 | 4.20E-03 | 9.63E-03 |
| FBXL16 | -2.86E+00 | 4.20E-03 | 9.65E-03 |
| KANK3 | -2.86E+00 | 4.21E-03 | 9.65E-03 |
| C6orf141 | 2.86E+00 | 4.21E-03 | 9.65E-03 |
| HBA1 | -2.86E+00 | 4.21E-03 | 9.65E-03 |
| SHROOM2 | -2.86E+00 | 4.21E-03 | 9.66E-03 |
| FOXD4L6 | 2.86E+00 | 4.22E-03 | 9.68E-03 |
| DTNB | 2.86E+00 | 4.23E-03 | 9.70E-03 |
| SDHAP3 | -2.86E+00 | 4.23E-03 | 9.70E-03 |
| COG3 | -2.86E+00 | 4.24E-03 | 9.71E-03 |
| RNF181 | -2.86E+00 | 4.25E-03 | 9.73E-03 |
| ZMYND15 | -2.86E+00 | 4.26E-03 | 9.75E-03 |
| SMOC2 | -2.86E+00 | 4.26E-03 | 9.76E-03 |
| COL4A1 | 2.86E+00 | 4.26E-03 | 9.77E-03 |
| TMBIM1 | -2.86E+00 | 4.26E-03 | 9.77E-03 |
| MZF1 | 2.86E+00 | 4.27E-03 | 9.78E-03 |
| CYP3A5 | -2.86E+00 | 4.27E-03 | 9.78E-03 |
| PRSS8 | -2.86E+00 | 4.27E-03 | 9.78E-03 |
| TBC1D28 | 2.86E+00 | 4.27E-03 | 9.78E-03 |
| DPPA3 | 2.86E+00 | 4.28E-03 | 9.80E-03 |
| HEPH | -2.86E+00 | 4.28E-03 | 9.80E-03 |
| ZFP3 | -2.86E+00 | 4.28E-03 | 9.80E-03 |
| MS4A10 | -2.86E+00 | 4.29E-03 | 9.81E-03 |
| WHSC1L1 | 2.86E+00 | 4.30E-03 | 9.84E-03 |
| MRPL1 | -2.85E+00 | 4.31E-03 | 9.87E-03 |
| C4orf22 | -2.85E+00 | 4.32E-03 | 9.87E-03 |
| C7orf71 | 2.85E+00 | 4.32E-03 | 9.88E-03 |
| FAM90A7 | 2.85E+00 | 4.32E-03 | 9.88E-03 |
| CCDC13 | -2.85E+00 | 4.33E-03 | 9.90E-03 |
| PLEKHM3 | -2.85E+00 | 4.33E-03 | 9.90E-03 |
| C19orf34 | 2.85E+00 | 4.33E-03 | 9.91E-03 |
| MFF | 2.85E+00 | 4.33E-03 | 9.91E-03 |
| XIAP | -2.85E+00 | 4.34E-03 | 9.91E-03 |
| USHBP1 | -2.85E+00 | 4.34E-03 | 9.92E-03 |
| SH2B1 | 2.85E+00 | 4.34E-03 | 9.93E-03 |
| OR51B6 | 2.85E+00 | 4.35E-03 | 9.94E-03 |
| SFN | 2.85E+00 | 4.35E-03 | 9.94E-03 |
| MAPK13 | 2.85E+00 | 4.35E-03 | 9.94E-03 |
| ZNF844 | -2.85E+00 | 4.35E-03 | 9.94E-03 |
| C14orf34 | 2.85E+00 | 4.36E-03 | 9.97E-03 |
| L3MBTL2 | 2.85E+00 | 4.37E-03 | 9.97E-03 |
| IL23A | 2.85E+00 | 4.37E-03 | 9.99E-03 |
| DNPEP | 2.85E+00 | 4.37E-03 | 9.99E-03 |
| CA5B | -2.85E+00 | 4.38E-03 | 9.99E-03 |
| CSDAP1 | 2.85E+00 | 4.38E-03 | 9.99E-03 |

| ZBP1 | 2.85E+00 | 4.39E-03 | 1.00E-02 |
| --- | --- | --- | --- |
| RPL22L1 | 2.85E+00 | 4.39E-03 | 1.00E-02 |
| FAM186A | 2.85E+00 | 4.40E-03 | 1.00E-02 |
| PAFAH1B1 | -2.85E+00 | 4.40E-03 | 1.00E-02 |
| STAG2 | -2.85E+00 | 4.40E-03 | 1.00E-02 |
| FOXI3 | 2.85E+00 | 4.41E-03 | 1.01E-02 |
| BAI3 | -2.85E+00 | 4.42E-03 | 1.01E-02 |
| HIBADH | -2.85E+00 | 4.42E-03 | 1.01E-02 |
| CATSPER1 | 2.85E+00 | 4.43E-03 | 1.01E-02 |
| TMEM176B | -2.85E+00 | 4.43E-03 | 1.01E-02 |
| LRRTM2 | -2.85E+00 | 4.43E-03 | 1.01E-02 |
| ZNF350 | -2.85E+00 | 4.44E-03 | 1.01E-02 |
| NHEDC1 | -2.85E+00 | 4.44E-03 | 1.01E-02 |
| PKIG | -2.85E+00 | 4.45E-03 | 1.01E-02 |
| FN1 | 2.85E+00 | 4.45E-03 | 1.01E-02 |
| PECR | -2.85E+00 | 4.45E-03 | 1.01E-02 |
| PLEKHB2 | 2.84E+00 | 4.45E-03 | 1.01E-02 |
| KRT38 | 2.84E+00 | 4.46E-03 | 1.02E-02 |
| BCAS3 | -2.84E+00 | 4.46E-03 | 1.02E-02 |
| CD164 | -2.84E+00 | 4.46E-03 | 1.02E-02 |
| DDHD2 | 2.84E+00 | 4.46E-03 | 1.02E-02 |
| LXN | -2.84E+00 | 4.47E-03 | 1.02E-02 |
| KRTAP2-1 | 2.84E+00 | 4.49E-03 | 1.02E-02 |
| WIBG | 2.84E+00 | 4.49E-03 | 1.02E-02 |
| JAK2 | -2.84E+00 | 4.49E-03 | 1.02E-02 |
| ZNF559 | -2.84E+00 | 4.50E-03 | 1.02E-02 |
| MECP2 | 2.84E+00 | 4.50E-03 | 1.02E-02 |
| PTPN7 | 2.84E+00 | 4.51E-03 | 1.03E-02 |
| NUB1 | 2.84E+00 | 4.51E-03 | 1.03E-02 |
| LHX2 | 2.84E+00 | 4.51E-03 | 1.03E-02 |
| RBMY2EP | 2.84E+00 | 4.51E-03 | 1.03E-02 |
| HIATL2 | 2.84E+00 | 4.51E-03 | 1.03E-02 |
| GRSF1 | 2.84E+00 | 4.52E-03 | 1.03E-02 |
| NFATC2 | 2.84E+00 | 4.52E-03 | 1.03E-02 |
| MAP9 | -2.84E+00 | 4.53E-03 | 1.03E-02 |
| BNIP3L | -2.84E+00 | 4.53E-03 | 1.03E-02 |
| SCN3A | 2.84E+00 | 4.53E-03 | 1.03E-02 |
| EGOT | -2.84E+00 | 4.53E-03 | 1.03E-02 |
| ALPK1 | -2.84E+00 | 4.53E-03 | 1.03E-02 |
| C18orf20 | -2.84E+00 | 4.53E-03 | 1.03E-02 |
| STARD4 | 2.84E+00 | 4.53E-03 | 1.03E-02 |
| VPS25 | 2.84E+00 | 4.54E-03 | 1.03E-02 |
| IGSF3 | 2.84E+00 | 4.54E-03 | 1.03E-02 |
| LGALS7 | 2.84E+00 | 4.54E-03 | 1.03E-02 |
| PSMG4 | 2.84E+00 | 4.54E-03 | 1.03E-02 |
| GOLM1 | 2.84E+00 | 4.55E-03 | 1.03E-02 |
| MYO1C | 2.84E+00 | 4.55E-03 | 1.03E-02 |
| HNRNPA1 | 2.84E+00 | 4.56E-03 | 1.04E-02 |
| C1orf125 | 2.84E+00 | 4.56E-03 | 1.04E-02 |
| RAB8B | -2.84E+00 | 4.57E-03 | 1.04E-02 |
| IL20 | -2.84E+00 | 4.57E-03 | 1.04E-02 |
| TRIM71 | 2.84E+00 | 4.57E-03 | 1.04E-02 |
| CHRNE | -2.84E+00 | 4.57E-03 | 1.04E-02 |
| TRIM24 | 2.84E+00 | 4.58E-03 | 1.04E-02 |
| MMP10 | 2.84E+00 | 4.58E-03 | 1.04E-02 |
| KBTBD12 | 2.83E+00 | 4.59E-03 | 1.04E-02 |
| ARPC4 | 2.83E+00 | 4.60E-03 | 1.04E-02 |

| PLAGL1 | 2.83E+00 | 4.60E-03 | 1.04E-02 |
| --- | --- | --- | --- |
| FLG | 2.83E+00 | 4.60E-03 | 1.04E-02 |
| C1orf198 | -2.83E+00 | 4.61E-03 | 1.04E-02 |
| FAM178B | 2.83E+00 | 4.61E-03 | 1.04E-02 |
| NODAL | 2.83E+00 | 4.61E-03 | 1.04E-02 |
| PHLDB3 | 2.83E+00 | 4.61E-03 | 1.04E-02 |
| NR0B2 | -2.83E+00 | 4.62E-03 | 1.05E-02 |
| TOE1 | 2.83E+00 | 4.62E-03 | 1.05E-02 |
| ITSN2 | -2.83E+00 | 4.63E-03 | 1.05E-02 |
| STRA13 | 2.83E+00 | 4.63E-03 | 1.05E-02 |
| ABCB4 | -2.83E+00 | 4.64E-03 | 1.05E-02 |
| KIF5C | 2.83E+00 | 4.65E-03 | 1.05E-02 |
| MRPL52 | 2.83E+00 | 4.66E-03 | 1.06E-02 |
| SNAP29 | -2.83E+00 | 4.67E-03 | 1.06E-02 |
| ADCY2 | -2.83E+00 | 4.69E-03 | 1.06E-02 |
| RRAS2 | -2.83E+00 | 4.69E-03 | 1.06E-02 |
| CDH2 | 2.83E+00 | 4.69E-03 | 1.06E-02 |
| PRDM2 | -2.83E+00 | 4.70E-03 | 1.06E-02 |
| COMMD10 | -2.83E+00 | 4.70E-03 | 1.06E-02 |
| FBXW7 | -2.83E+00 | 4.70E-03 | 1.06E-02 |
| TFF1 | 2.83E+00 | 4.70E-03 | 1.06E-02 |
| GPR176 | 2.83E+00 | 4.70E-03 | 1.06E-02 |
| FMO6P | -2.83E+00 | 4.71E-03 | 1.06E-02 |
| ZHX2 | 2.83E+00 | 4.71E-03 | 1.07E-02 |
| C10orf68 | -2.83E+00 | 4.72E-03 | 1.07E-02 |
| ZNF573 | -2.83E+00 | 4.72E-03 | 1.07E-02 |
| LIPK | 2.83E+00 | 4.72E-03 | 1.07E-02 |
| ANKRD34C | -2.83E+00 | 4.73E-03 | 1.07E-02 |
| C1orf86 | 2.82E+00 | 4.74E-03 | 1.07E-02 |
| DCAF11 | -2.82E+00 | 4.74E-03 | 1.07E-02 |
| CTLA4 | 2.82E+00 | 4.75E-03 | 1.07E-02 |
| SEC22A | -2.82E+00 | 4.75E-03 | 1.07E-02 |
| HORMAD2 | -2.82E+00 | 4.76E-03 | 1.07E-02 |
| GIGYF2 | 2.82E+00 | 4.76E-03 | 1.08E-02 |
| TRIM44 | 2.82E+00 | 4.76E-03 | 1.08E-02 |
| TPSD1 | -2.82E+00 | 4.76E-03 | 1.08E-02 |
| C14orf176 | -2.82E+00 | 4.77E-03 | 1.08E-02 |
| C5orf45 | -2.82E+00 | 4.77E-03 | 1.08E-02 |
| FAM104B | -2.82E+00 | 4.78E-03 | 1.08E-02 |
| FAHD2A | -2.82E+00 | 4.78E-03 | 1.08E-02 |
| GRM1 | 2.82E+00 | 4.79E-03 | 1.08E-02 |
| C12orf5 | 2.82E+00 | 4.80E-03 | 1.08E-02 |
| ZNF804B | 2.82E+00 | 4.80E-03 | 1.08E-02 |
| ENSA | -2.82E+00 | 4.81E-03 | 1.09E-02 |
| NAP1L6 | 2.82E+00 | 4.81E-03 | 1.09E-02 |
| ALKBH7 | -2.82E+00 | 4.82E-03 | 1.09E-02 |
| USP2 | -2.82E+00 | 4.83E-03 | 1.09E-02 |
| TSC2 | 2.82E+00 | 4.83E-03 | 1.09E-02 |
| PLAG1 | 2.82E+00 | 4.84E-03 | 1.09E-02 |
| C19orf44 | 2.82E+00 | 4.84E-03 | 1.09E-02 |
| KIAA1211 | 2.82E+00 | 4.85E-03 | 1.09E-02 |
| ATAD3C | -2.82E+00 | 4.88E-03 | 1.10E-02 |
| OR2AK2 | 2.82E+00 | 4.88E-03 | 1.10E-02 |
| LRP1 | 2.82E+00 | 4.88E-03 | 1.10E-02 |
| APBB2 | -2.82E+00 | 4.89E-03 | 1.10E-02 |
| LOC441046 | -2.82E+00 | 4.89E-03 | 1.10E-02 |
| OXNAD1 | -2.82E+00 | 4.89E-03 | 1.10E-02 |

| NEUROG2 | 2.81E+00 | 4.90E-03 | 1.10E-02 |
| --- | --- | --- | --- |
| CSF3R | 2.81E+00 | 4.90E-03 | 1.10E-02 |
| EDN1 | -2.81E+00 | 4.92E-03 | 1.11E-02 |
| ACTA1 | 2.81E+00 | 4.92E-03 | 1.11E-02 |
| C11orf88 | -2.81E+00 | 4.92E-03 | 1.11E-02 |
| NCRNA00099 | 2.81E+00 | 4.92E-03 | 1.11E-02 |
| C15orf48 | 2.81E+00 | 4.92E-03 | 1.11E-02 |
| DLG2 | -2.81E+00 | 4.93E-03 | 1.11E-02 |
| MUC13 | 2.81E+00 | 4.93E-03 | 1.11E-02 |
| SLC17A9 | 2.81E+00 | 4.93E-03 | 1.11E-02 |
| KCNS2 | -2.81E+00 | 4.94E-03 | 1.11E-02 |
| P704P | 2.81E+00 | 4.94E-03 | 1.11E-02 |
| C1QTNF9 | -2.81E+00 | 4.94E-03 | 1.11E-02 |
| PCDH17 | -2.81E+00 | 4.94E-03 | 1.11E-02 |
| LY6H | 2.81E+00 | 4.95E-03 | 1.11E-02 |
| C7orf33 | 2.81E+00 | 4.95E-03 | 1.11E-02 |
| ZMYM6 | -2.81E+00 | 4.96E-03 | 1.11E-02 |
| C1QBP | 2.81E+00 | 4.96E-03 | 1.12E-02 |
| RDH8 | 2.81E+00 | 4.96E-03 | 1.12E-02 |
| RWDD1 | -2.81E+00 | 4.97E-03 | 1.12E-02 |
| LOC643008 | -2.81E+00 | 4.98E-03 | 1.12E-02 |
| OR7A5 | 2.81E+00 | 4.98E-03 | 1.12E-02 |
| CYS1 | -2.81E+00 | 5.00E-03 | 1.12E-02 |
| RNF19A | -2.81E+00 | 5.00E-03 | 1.12E-02 |
| WBP11P1 | 2.81E+00 | 5.00E-03 | 1.12E-02 |
| MS4A7 | -2.81E+00 | 5.01E-03 | 1.12E-02 |
| NOD2 | 2.81E+00 | 5.01E-03 | 1.12E-02 |
| C5orf56 | -2.81E+00 | 5.01E-03 | 1.13E-02 |
| CLINT1 | -2.81E+00 | 5.02E-03 | 1.13E-02 |
| OR1L8 | -2.81E+00 | 5.02E-03 | 1.13E-02 |
| IL16 | -2.81E+00 | 5.03E-03 | 1.13E-02 |
| C7orf25 | -2.81E+00 | 5.04E-03 | 1.13E-02 |
| GATA1 | -2.80E+00 | 5.04E-03 | 1.13E-02 |
| WNT4 | -2.80E+00 | 5.05E-03 | 1.13E-02 |
| AAA1 | 2.80E+00 | 5.05E-03 | 1.13E-02 |
| CLTC | 2.80E+00 | 5.05E-03 | 1.13E-02 |
| ATG12 | -2.80E+00 | 5.05E-03 | 1.13E-02 |
| BCAT1 | 2.80E+00 | 5.05E-03 | 1.13E-02 |
| TBK1 | 2.80E+00 | 5.05E-03 | 1.13E-02 |
| AKR1B10 | 2.80E+00 | 5.06E-03 | 1.13E-02 |
| CHMP2A | -2.80E+00 | 5.06E-03 | 1.14E-02 |
| KDM5D | 2.80E+00 | 5.06E-03 | 1.14E-02 |
| FGF18 | -2.80E+00 | 5.06E-03 | 1.14E-02 |
| SPIRE2 | 2.80E+00 | 5.07E-03 | 1.14E-02 |
| MMP23B | -2.80E+00 | 5.08E-03 | 1.14E-02 |
| STX10 | 2.80E+00 | 5.08E-03 | 1.14E-02 |
| DBR1 | 2.80E+00 | 5.09E-03 | 1.14E-02 |
| ZC3H14 | -2.80E+00 | 5.09E-03 | 1.14E-02 |
| SFRS15 | 2.80E+00 | 5.10E-03 | 1.14E-02 |
| MYO1F | 2.80E+00 | 5.11E-03 | 1.14E-02 |
| NPEPL1 | 2.80E+00 | 5.11E-03 | 1.15E-02 |
| REST | 2.80E+00 | 5.11E-03 | 1.15E-02 |
| BRSK2 | 2.80E+00 | 5.12E-03 | 1.15E-02 |
| LOC401127 | -2.80E+00 | 5.12E-03 | 1.15E-02 |
| TCEA2 | -2.80E+00 | 5.14E-03 | 1.15E-02 |
| LOC285074 | -2.80E+00 | 5.15E-03 | 1.15E-02 |
| NR2F2 | -2.80E+00 | 5.15E-03 | 1.15E-02 |

| UBE2Q1 | 2.80E+00 | 5.16E-03 | 1.15E-02 |
| --- | --- | --- | --- |
| SPG11 | -2.80E+00 | 5.16E-03 | 1.15E-02 |
| NPHS1 | 2.80E+00 | 5.17E-03 | 1.16E-02 |
| RBM4B | 2.80E+00 | 5.17E-03 | 1.16E-02 |
| SQRDL | -2.80E+00 | 5.17E-03 | 1.16E-02 |
| GRIN3B | 2.80E+00 | 5.18E-03 | 1.16E-02 |
| CETN3 | -2.80E+00 | 5.18E-03 | 1.16E-02 |
| WNT16 | 2.80E+00 | 5.19E-03 | 1.16E-02 |
| ZC3H7B | 2.80E+00 | 5.20E-03 | 1.16E-02 |
| TMEM22 | 2.79E+00 | 5.20E-03 | 1.16E-02 |
| RAD9B | 2.79E+00 | 5.20E-03 | 1.16E-02 |
| OR4M2 | 2.79E+00 | 5.20E-03 | 1.16E-02 |
| ATP8B3 | 2.79E+00 | 5.21E-03 | 1.16E-02 |
| ZNF878 | 2.79E+00 | 5.21E-03 | 1.16E-02 |
| GIMAP2 | -2.79E+00 | 5.21E-03 | 1.17E-02 |
| CGB8 | 2.79E+00 | 5.22E-03 | 1.17E-02 |
| GSTA4 | -2.79E+00 | 5.22E-03 | 1.17E-02 |
| SYT9 | -2.79E+00 | 5.22E-03 | 1.17E-02 |
| GPR81 | 2.79E+00 | 5.23E-03 | 1.17E-02 |
| UTP11L | 2.79E+00 | 5.24E-03 | 1.17E-02 |
| MDM2 | -2.79E+00 | 5.25E-03 | 1.17E-02 |
| MTCH1 | 2.79E+00 | 5.25E-03 | 1.17E-02 |
| CPXM2 | -2.79E+00 | 5.27E-03 | 1.18E-02 |
| GTF2H2 | 2.79E+00 | 5.27E-03 | 1.18E-02 |
| PLSCR2 | 2.79E+00 | 5.27E-03 | 1.18E-02 |
| SEMA4G | 2.79E+00 | 5.27E-03 | 1.18E-02 |
| DRD4 | 2.79E+00 | 5.28E-03 | 1.18E-02 |
| KIAA1328 | -2.79E+00 | 5.29E-03 | 1.18E-02 |
| CCDC148 | -2.79E+00 | 5.30E-03 | 1.18E-02 |
| MT1G | 2.79E+00 | 5.30E-03 | 1.18E-02 |
| C21orf99 | 2.79E+00 | 5.31E-03 | 1.18E-02 |
| GBA3 | -2.79E+00 | 5.32E-03 | 1.19E-02 |
| RAB1B | 2.79E+00 | 5.33E-03 | 1.19E-02 |
| ZNF57 | -2.79E+00 | 5.33E-03 | 1.19E-02 |
| CBWD6 | 2.79E+00 | 5.36E-03 | 1.19E-02 |
| TCP11 | -2.79E+00 | 5.36E-03 | 1.19E-02 |
| TPM2 | 2.79E+00 | 5.36E-03 | 1.19E-02 |
| NIT1 | -2.78E+00 | 5.36E-03 | 1.20E-02 |
| NLGN3 | 2.78E+00 | 5.37E-03 | 1.20E-02 |
| HIST3H2BB | 2.78E+00 | 5.37E-03 | 1.20E-02 |
| DDX3Y | 2.78E+00 | 5.38E-03 | 1.20E-02 |
| SFRS12 | -2.78E+00 | 5.38E-03 | 1.20E-02 |
| PRH2 | -2.78E+00 | 5.39E-03 | 1.20E-02 |
| IFI35 | 2.78E+00 | 5.40E-03 | 1.20E-02 |
| RSPO1 | -2.78E+00 | 5.42E-03 | 1.21E-02 |
| IL2 | -2.78E+00 | 5.42E-03 | 1.21E-02 |
| IDI1 | -2.78E+00 | 5.43E-03 | 1.21E-02 |
| LOC148696 | -2.78E+00 | 5.43E-03 | 1.21E-02 |
| TRIM40 | 2.78E+00 | 5.43E-03 | 1.21E-02 |
| LOC150568 | 2.78E+00 | 5.44E-03 | 1.21E-02 |
| C1orf51 | -2.78E+00 | 5.46E-03 | 1.21E-02 |
| RPL23AP7 | 2.78E+00 | 5.47E-03 | 1.22E-02 |
| MBD3L2 | 2.78E+00 | 5.48E-03 | 1.22E-02 |
| IL1F9 | -2.78E+00 | 5.48E-03 | 1.22E-02 |
| ZBED2 | 2.78E+00 | 5.50E-03 | 1.22E-02 |
| C1orf69 | 2.78E+00 | 5.51E-03 | 1.22E-02 |
| CYP2J2 | -2.78E+00 | 5.51E-03 | 1.23E-02 |

| TRAPPC6A | -2.78E+00 | 5.52E-03 | 1.23E-02 |
| --- | --- | --- | --- |
| CMTM8 | -2.77E+00 | 5.53E-03 | 1.23E-02 |
| LOC100272216 | 2.77E+00 | 5.54E-03 | 1.23E-02 |
| MIA3 | -2.77E+00 | 5.55E-03 | 1.23E-02 |
| ZNF19 | -2.77E+00 | 5.56E-03 | 1.24E-02 |
| BBS10 | -2.77E+00 | 5.56E-03 | 1.24E-02 |
| FLJ39582 | -2.77E+00 | 5.57E-03 | 1.24E-02 |
| C2orf80 | 2.77E+00 | 5.57E-03 | 1.24E-02 |
| CSF1 | 2.77E+00 | 5.57E-03 | 1.24E-02 |
| RHOG | 2.77E+00 | 5.57E-03 | 1.24E-02 |
| PIRT | 2.77E+00 | 5.58E-03 | 1.24E-02 |
| LRRC31 | -2.77E+00 | 5.60E-03 | 1.24E-02 |
| MAGOH | 2.77E+00 | 5.61E-03 | 1.25E-02 |
| SMU1 | 2.77E+00 | 5.61E-03 | 1.25E-02 |
| C10orf58 | -2.77E+00 | 5.61E-03 | 1.25E-02 |
| MAPK15 | 2.77E+00 | 5.62E-03 | 1.25E-02 |
| LOC148824 | 2.77E+00 | 5.62E-03 | 1.25E-02 |
| FAM165B | -2.77E+00 | 5.64E-03 | 1.25E-02 |
| DZIP1L | 2.77E+00 | 5.64E-03 | 1.25E-02 |
| BID | 2.77E+00 | 5.64E-03 | 1.25E-02 |
| FAM48A | 2.77E+00 | 5.64E-03 | 1.25E-02 |
| RRAS | -2.77E+00 | 5.65E-03 | 1.25E-02 |
| LOC100130522 | -2.77E+00 | 5.65E-03 | 1.25E-02 |
| WFDC8 | 2.77E+00 | 5.65E-03 | 1.25E-02 |
| RRAGA | -2.77E+00 | 5.66E-03 | 1.26E-02 |
| FAM36A | -2.77E+00 | 5.66E-03 | 1.26E-02 |
| PIGG | 2.77E+00 | 5.66E-03 | 1.26E-02 |
| NFS1 | 2.77E+00 | 5.68E-03 | 1.26E-02 |
| ZNF516 | 2.77E+00 | 5.68E-03 | 1.26E-02 |
| GRID2 | 2.77E+00 | 5.69E-03 | 1.26E-02 |
| DKFZp434J0226 | 2.77E+00 | 5.69E-03 | 1.26E-02 |
| TEKT1 | -2.77E+00 | 5.69E-03 | 1.26E-02 |
| EYA1 | 2.77E+00 | 5.70E-03 | 1.26E-02 |
| FAM27C | 2.77E+00 | 5.70E-03 | 1.26E-02 |
| SALL3 | 2.76E+00 | 5.71E-03 | 1.26E-02 |
| AGTR1 | -2.76E+00 | 5.71E-03 | 1.27E-02 |
| C8orf86 | 2.76E+00 | 5.72E-03 | 1.27E-02 |
| SLC39A10 | 2.76E+00 | 5.72E-03 | 1.27E-02 |
| ATP8A1 | -2.76E+00 | 5.72E-03 | 1.27E-02 |
| CXCR2P1 | 2.76E+00 | 5.72E-03 | 1.27E-02 |
| FAM21A | 2.76E+00 | 5.73E-03 | 1.27E-02 |
| ZNF592 | 2.76E+00 | 5.73E-03 | 1.27E-02 |
| CRIP2 | -2.76E+00 | 5.74E-03 | 1.27E-02 |
| ICAM2 | -2.76E+00 | 5.75E-03 | 1.27E-02 |
| FKBPL | 2.76E+00 | 5.75E-03 | 1.27E-02 |
| EIF4A2 | -2.76E+00 | 5.75E-03 | 1.27E-02 |
| RNF2 | 2.76E+00 | 5.75E-03 | 1.27E-02 |
| C12orf27 | 2.76E+00 | 5.75E-03 | 1.27E-02 |
| CSNK1G3 | -2.76E+00 | 5.75E-03 | 1.27E-02 |
| CHMP4C | 2.76E+00 | 5.76E-03 | 1.27E-02 |
| NAT6 | -2.76E+00 | 5.76E-03 | 1.27E-02 |
| RASGRF2 | -2.76E+00 | 5.77E-03 | 1.28E-02 |
| CXADR | -2.76E+00 | 5.77E-03 | 1.28E-02 |
| C9orf128 | -2.76E+00 | 5.79E-03 | 1.28E-02 |
| CD68 | 2.76E+00 | 5.79E-03 | 1.28E-02 |
| SLC25A35 | 2.76E+00 | 5.79E-03 | 1.28E-02 |
| RBM18 | -2.76E+00 | 5.80E-03 | 1.28E-02 |

| CDK15 | -2.76E+00 | 5.82E-03 | 1.28E-02 |
| --- | --- | --- | --- |
| DCDC1 | 2.76E+00 | 5.82E-03 | 1.29E-02 |
| C9orf71 | -2.76E+00 | 5.82E-03 | 1.29E-02 |
| LOC284551 | 2.76E+00 | 5.82E-03 | 1.29E-02 |
| TRH | 2.76E+00 | 5.82E-03 | 1.29E-02 |
| GRB7 | 2.76E+00 | 5.83E-03 | 1.29E-02 |
| MYEF2 | 2.76E+00 | 5.83E-03 | 1.29E-02 |
| HCG27 | -2.76E+00 | 5.83E-03 | 1.29E-02 |
| HABP2 | -2.76E+00 | 5.83E-03 | 1.29E-02 |
| FKBP1A | 2.76E+00 | 5.84E-03 | 1.29E-02 |
| KIAA0232 | -2.76E+00 | 5.86E-03 | 1.29E-02 |
| FAM136B | 2.76E+00 | 5.87E-03 | 1.29E-02 |
| C15orf59 | -2.76E+00 | 5.87E-03 | 1.30E-02 |
| ANKRD43 | -2.75E+00 | 5.89E-03 | 1.30E-02 |
| GP6 | -2.75E+00 | 5.89E-03 | 1.30E-02 |
| HEXA | -2.75E+00 | 5.90E-03 | 1.30E-02 |
| TMEM50A | -2.75E+00 | 5.90E-03 | 1.30E-02 |
| C22orf46 | 2.75E+00 | 5.90E-03 | 1.30E-02 |
| C3orf1 | 2.75E+00 | 5.91E-03 | 1.30E-02 |
| HIST3H2A | 2.75E+00 | 5.91E-03 | 1.30E-02 |
| MCAT | 2.75E+00 | 5.92E-03 | 1.30E-02 |
| ETV2 | -2.75E+00 | 5.94E-03 | 1.31E-02 |
| LRRC27 | -2.75E+00 | 5.94E-03 | 1.31E-02 |
| FAM46C | -2.75E+00 | 5.94E-03 | 1.31E-02 |
| C19orf69 | 2.75E+00 | 5.95E-03 | 1.31E-02 |
| C9orf156 | -2.75E+00 | 5.95E-03 | 1.31E-02 |
| PHF7 | -2.75E+00 | 5.97E-03 | 1.32E-02 |
| HTRA2 | 2.75E+00 | 5.97E-03 | 1.32E-02 |
| DUT | -2.75E+00 | 5.98E-03 | 1.32E-02 |
| MYST1 | -2.75E+00 | 5.98E-03 | 1.32E-02 |
| LOC100101938 | 2.75E+00 | 5.99E-03 | 1.32E-02 |
| COX5B | -2.75E+00 | 6.00E-03 | 1.32E-02 |
| TRIM62 | 2.75E+00 | 6.00E-03 | 1.32E-02 |
| MAP4K3 | -2.75E+00 | 6.03E-03 | 1.33E-02 |
| SLC6A12 | -2.75E+00 | 6.04E-03 | 1.33E-02 |
| ARHGAP10 | -2.75E+00 | 6.04E-03 | 1.33E-02 |
| EIF3J | 2.75E+00 | 6.04E-03 | 1.33E-02 |
| PLAC2 | 2.75E+00 | 6.04E-03 | 1.33E-02 |
| PSMB3 | 2.75E+00 | 6.05E-03 | 1.33E-02 |
| SURF2 | 2.75E+00 | 6.05E-03 | 1.33E-02 |
| SLC18A3 | 2.75E+00 | 6.06E-03 | 1.33E-02 |
| BGLAP | 2.75E+00 | 6.06E-03 | 1.33E-02 |
| MICA | -2.74E+00 | 6.06E-03 | 1.33E-02 |
| HECW1 | 2.74E+00 | 6.06E-03 | 1.33E-02 |
| MMD2 | 2.74E+00 | 6.08E-03 | 1.34E-02 |
| EXTL1 | 2.74E+00 | 6.10E-03 | 1.34E-02 |
| PRAC | 2.74E+00 | 6.10E-03 | 1.34E-02 |
| DUSP2 | 2.74E+00 | 6.10E-03 | 1.34E-02 |
| MED20 | 2.74E+00 | 6.11E-03 | 1.34E-02 |
| FAM32A | -2.74E+00 | 6.11E-03 | 1.34E-02 |
| MED31 | -2.74E+00 | 6.11E-03 | 1.34E-02 |
| SLC9A3R1 | 2.74E+00 | 6.12E-03 | 1.34E-02 |
| FANCL | 2.74E+00 | 6.12E-03 | 1.35E-02 |
| C19orf70 | -2.74E+00 | 6.13E-03 | 1.35E-02 |
| C12orf66 | 2.74E+00 | 6.13E-03 | 1.35E-02 |
| CACNA2D2 | -2.74E+00 | 6.14E-03 | 1.35E-02 |
| PRSS50 | 2.74E+00 | 6.15E-03 | 1.35E-02 |

| PPP1R3A | 2.74E+00 | 6.15E-03 | 1.35E-02 |
| --- | --- | --- | --- |
| SLC29A1 | -2.74E+00 | 6.16E-03 | 1.35E-02 |
| KAL1 | -2.74E+00 | 6.16E-03 | 1.35E-02 |
| DNAJC21 | -2.74E+00 | 6.17E-03 | 1.35E-02 |
| TGFBR1 | -2.74E+00 | 6.17E-03 | 1.35E-02 |
| MRPL16 | -2.74E+00 | 6.18E-03 | 1.36E-02 |
| FAT2 | 2.74E+00 | 6.18E-03 | 1.36E-02 |
| SSX5 | 2.74E+00 | 6.19E-03 | 1.36E-02 |
| DAP3 | 2.74E+00 | 6.21E-03 | 1.36E-02 |
| KY | 2.74E+00 | 6.21E-03 | 1.36E-02 |
| CNOT2 | 2.74E+00 | 6.22E-03 | 1.36E-02 |
| ST13 | -2.74E+00 | 6.24E-03 | 1.37E-02 |
| HIST1H2BI | 2.74E+00 | 6.24E-03 | 1.37E-02 |
| TTC9 | -2.73E+00 | 6.26E-03 | 1.37E-02 |
| CCL4 | 2.73E+00 | 6.26E-03 | 1.37E-02 |
| TSPY4 | 2.73E+00 | 6.27E-03 | 1.37E-02 |
| CPA3 | -2.73E+00 | 6.28E-03 | 1.38E-02 |
| RBP3 | 2.73E+00 | 6.28E-03 | 1.38E-02 |
| EPHB4 | 2.73E+00 | 6.28E-03 | 1.38E-02 |
| GADD45A | -2.73E+00 | 6.29E-03 | 1.38E-02 |
| LOC200726 | 2.73E+00 | 6.30E-03 | 1.38E-02 |
[truncated: 477,400 more chars]
